# Supplementary material for: Influence of Primary Coordination Sphere on Anion Rebound Selectivity in Nonheme Fe Enzyme-Catalyzed C(sp3)–H Functionalization: A Comparative Experimental and Computational Study of EgtB and ACCO
Source: bioRxiv. 2026 Jul 13:2026.07.10.737789. Preprint. [Version 1] doi: 10.64898/2026.07.10.737789 (PMC13404739; doi:10.64898/2026.07.10.737789)

Supporting Information for

Influence of Primary Coordination Sphere on Anion Rebound Selectivity in Nonheme Fe Enzyme-Catalyzed C(sp^3)–H Functionalization: A Comparative Experimental and Computational Study of EgtB and ACCO

Liu-Peng Zhao,[1,#] Rui Guo,[1,#] Binh Khanh Mai,[2,#] Heyu Chen,[1] Peng Liu,[2,*] and Yang Yang[1,3,4,5,*]

[1] Department of Chemistry and Biochemistry, University of California Santa Barbara, Santa Barbara, California 93106, United States.

[2] Department of Chemistry, University of Pittsburgh, Pittsburgh, Pennsylvania 15260, United States.

[3] Department of Bioengineering, University of California Santa Barbara, Santa Barbara, California 93106, United States.

[4] Biomolecular Science and Engineering (BMSE) Program, University of California Santa Barbara, Santa Barbara, California 93106, United States.

[5] Howard Hughes Medical Institute, University of California Santa Barbara, Santa Barbara, California 93106, United States.

[#] These authors contributed equally.

*E-mail: pengliu@pitt.edu; yang@chem.ucsb.edu

Table of Contents

I. General methods 4

II. Evaluation of Fe-dependent proteins 11

III. Protein and DNA sequences 12

IV. Determination of nonheme enzyme concentrations 24

V. Mechanism studies. 28

VI. Primary coordination sphere effects on radical rebound activity and selectivity 36

VII. pH effects on radical rebound activity and selectivity 45

VIII. Synthesis and characterization of substrates and products. 51

IX. HPLC calibration curves 58

X. Chiral HPLC traces 60

XI. Computational Details 66

1. Classical Molecular Dynamics (MD) Simulations 66
2. Arginine/π interaction between R175 and the phenyl group of the *N*-fluoroamide 1 in $ACCO_{CHF}$ 70
3. Density Functional Theory Calculations 70
4. QM/MM Calculations 71
5. Bond Dissociation Enthalpy Calculations and Effect of Hydrogen Bond on N–F Bond . 72
6. Protonation State of Water Molecules Bound to the Fe Center 73
7. Protonation Abstraction from Fe(III)–F Species 77
8. Reaction Energy Profile of Nonheme Fe Enzyme-Catalyzed C–H Fluorination 80
9. Correlation Between Activation Barrier and Other Parameters for Radical Rebound 81

10. Alternative Regression Model for Fluorine Atom Abstraction 82

11. Alternative Regression Model for Radical Rebound 82

12. Substrate Reduction by Fe(II) Species 82

13. Benzyl Radical Oxidation Pathway by Fe(III)–F Species 84

14. Binding Free Energies of Azide Anion to Fe(II) and Fe(III)–F Species 86

15. Radical Rebound Transition States from QM/MM Calculations 87

16. Classical MD Simulations for $EgtB_{CHF1}$ H138D Variant 89

XII. References 90

XIII. Cartesian Coordinates and Energies 97

XIV. ^{1}H and ^{13}C NMR Spectra of Compounds 277

I. General methods

General. Unless otherwise noted, all chemicals and reagents were obtained from commercial suppliers (Sigma-Aldrich, VWR, Alfa Aesar, Combi-Blocks, AmBeed, Oakwood, and Enamine) and used without further purification. Silica gel chromatography was carried out using AMD Silica Gel 60, 230-400 mesh. ^{1}H and ^{13}C NMR spectra were recorded on Varian 500, Varian 600, Bruker 400, or Bruker 500 MHz instruments in $CDCl_3$ and are referenced to residual solvent signals. ^{19}F NMR spectra (where applicable) were recorded on a Bruker 400 MHz. Data for ^{1}H NMR are reported as follows: chemical shift (δ: ppm), multiplicity (s = singlet, d = doublet, t = triplet, q = quartet, p = pentet, sext = sextet, m = multiplet, dd = doublet of doublets, dt = doublet of triplets, dq = doublet of quartets, ddd = doublet of doublet of doublets, ddt = doublet of doublet of triplets, brs = broad singlet), coupling constant (Hz), integration. Sonication on a small scale was performed using a BioLogics ultrasonic homogenizer (model 150VT) equipped with a stepped microtip or a Qsonica Q500 sonicator with a 24-tip horn. All IR spectra were recorded on a Thermo Scientific Nicolet iS5 spectrometer (iD5 ATR, diamond). High-resolution mass spectrometry data were obtained at the Mass Spectral Facilities at the University of California Santa Barbara and the University of California Irvine. High-resolution accurate mass (HRAM) ESI data was analyzed on a Waters LCT Premier mass spectrometer with a LEAP PAL autosampler with isocratic MeOH flow (no column; direct injection). Molecular formulas (MF) were validated by lock mass calibration to sodiated polyethylene glycol polymer or monomethyl ether polyethylene glycol polymer standards. High-resolution accurate mass (HRAM) EI data was acquired using a Waters LCT Premier time-of-flight (TOF) mass spectrometer. Masses of positively charged ions were calibrated using a methanol solution of polyethylene glycol or polyethylene glycol monomethyl ether as an internal standard. All samples were dissolved in CH_2Cl_2 and were directly infused unless otherwise noted. Synthetic reactions were monitored by thin-layer chromatography (TLC, Silicycle TLG-R10014BK-323 gel plates) using a UV lamp or an appropriate TLC stain for visualization.

Cell cultures were grown in Luria-Bertani medium (LB) and Terrific Broth medium (TB) or Hyper Broth medium (AthenaES) (HB). M9-N minimal medium (M9-N buffer, pH = 7.4) was used as a buffering system for whole-cell and lysate biotransformation. The M9-N buffer contains 47.7 mM Na_2HPO_4, 22.0 mM KH_2PO_4, 8.6 mM NaCl, 2.0 mM $MgSO_4$, and 0.1 mM $CaCl_2$. The extraction solution was prepared by adding 0.5 mmol mesitylene to 500 mL diisopropyl ether (*i*-Pr_2O). Diisopropyl ether was used as the extraction solvent, as conventional extraction solvent such as EtOAc would co-elude with the *N*-fluoroamide substrate in our HPLC analysis. Our studies showed that the substrates and products are highly soluble in *i*-Pr_2O.

Cloning and site-saturation mutagenesis. pET-28b(+) was used as the cloning and expression vector for *Mycobacterium thermoresistible* EgtB (*Mth*EgtB) with an *N*-terminal 6×His-tag. The gene encoding *Mth*EgtB was codon-optimized for protein production using *E. coli* as the host organism and purchased as gBlocks from GeneralBiol.[1] The gene was cloned into pET-28b(+) between restriction sites Nde I and Hind III. Nonheme Fe enzymes with a C-terminal 6×His-tag were cloned into pET-22b(+) between Nde I and Xho I. Site-saturation mutagenesis was performed using the "22c-trick" method described by Kille *et al.*[2] PCR primers were ordered from IDT or Azenta (formerly Genewiz). The PCR products were gel purified and ligated using a Gibson mix prepared from 5X isothermal (ISO) reaction buffer (25% PEG-8000, 500 mM Tris-HCl pH 7.5, 50 mM $MgCl_2$, 50 mM DTT, 1 mM each of the dNTPs, and 5 mM NAD), T5 exonuclease, Phusion DNA polymerase, and Taq DNA ligase.[3] The ligation mixture was used directly to transform electrocompetent *E. coli* strain *E. cloni* BL21(DE3) cells (Lucigen).

Procedure for the directed evolution of EgtB$_{CHF}$ variants

General note. Initially, due to the relatively low total activity of the new-to-nature fluorinase, directed evolution was carried out using 24-well deep-well plates to ensure excellent reproducibility. When the total activity of an intermediate fluorinase variant is sufficiently high, directed evolution was carried out using 96-well deep-well plates to have higher throughput.

Expression of *Mth*EgtB variants in 24-well plates. For $EgtB_{CHF1}$, from wt *Mth*EgtB to *Mth*EgtB Y377W W415R R87D T141I, directed evolution was performed in 24-well plates. For $EgtB_{CHF2}$, from wt *Mth*EgtB to *Mth*EgtB Y377W W415R R87D T141M R379A, directed evolution of $EgtB_{CHF2}$ was performed in 24-well plates. Single colonies from LB_{kan} agar plates were picked using sterile toothpicks and cultured in deep-well 96-well plates containing LB_{kan} (400 µL/well) at 37 °C, 250 rpm shaking overnight. TB_{kan} (4.0 mL/well) in four 24-well plates was then inoculated with an aliquot (200 µL/well) of these overnight cultures and allowed to shake for 2.5 h at 37 °C and 230 rpm. The plates were cooled on ice for 20 min before induction with 0.5 mM isopropyl β-D-1-thiogalactopyranoside (IPTG, final concentration). Expression was conducted at 22 °C, 150 rpm for 22 h.

Whole-cell reaction screening in 24-well plates. *E. coli* cells in deep-well 24-well plates were then pelleted by centrifugation (4000 rpm, 5 min, 4 °C) using an Eppendorf 5910R tabletop centrifuge. After centrifugation, the supernatant was removed, and the cell pellet was resuspended in M9-N buffer (600 µL) by gentle shaking using a Fisher Scientific microplate shaker (800 rpm, 3 min). The 24-well plate was then transferred into a Coy anaerobic chamber. In the Coy chamber, the *N*-fluoroamide substrate **1** (10 µL, 0.4 M in EtOH) and ferrous ammonium sulfate (10 µL, 40 mM in ddH_2O) were added to each well in succession using an Eppendorf Xplorer multichannel pipette. The plates were sealed with an aluminum foil and shaken on a Corning microplate shaker at room temperature and 680 rpm. After 6–18 h, the 24-well plate was taken out of the anaerobic chamber, the seal was removed, and the analytical scale reactions were worked up following the appropriate method below.

Chiral HPLC analysis. The reaction mixtures were quenched by the addition of 600 µL extraction solution (0.5 mM mesitylene in diisopropyl ether, a non-UV active polar solvent) using an Eppendorf Xplorer plus, 12-channel, 50–1000 µL electronic pipette. The plate was tightly sealed with a reusable silicone mat, shaken vigorously for 30 times, and centrifuged (4500 rpm, 20 min)

to separate the organic and the aqueous layers. The organic layers (350 µL/well) were transferred to 500 µL vial inserts using an Eppendorf Xplorer, 12-channel, 15–300 µL electronic pipette. Chiral HPLC analysis was conducted using a Shimadzu i-series (66 MPa) HPLC with IPA/hexanes as the mobile phase. The Chiralpak IB-N5 (4.6 mm × 25 cm, 5 micron), Chiralpak IC (4.6 mm × 25 cm, 5 micron) and Chiralpak IG (4.6 mm × 25 cm, 5 micron) were used for this normal phase HPLC analysis (*vide infra*).

Expression of *Mth*EgtB variants in 96-well plates. For $EgtB_{CHF1}$, from *Mth*EgtB Y377W W415R R87D T141I to the final variant, directed evolution of was performed in 96-well plates. For $EgtB_{CHF2}$, from *Mth*EgtB Y377W W415R R87D T141M R379A to the final variant, directed evolution of was performed in 96-well plates. Single colonies from LB_{kan} agar plates were picked using sterile toothpicks and cultured in deep-well 96-well plates containing LB_{kan} (400 µL/well) at 37 °C, 250 rpm shaking overnight. TB_{kan} (1000 µL/well) in a deep-well 96-well plate was then inoculated with an aliquot (50 µL/well) of these overnight cultures and allowed to shake for 3 h at 37 °C and 250 rpm. The plates were cooled on ice for 20 min before induction with 0.5 mM IPTG (final concentration). Expression was conducted at 22 °C, 220 rpm for 22 h.

Whole-cell reaction screening in 96-well plates. *E. coli* cells were then pelleted by centrifugation (4000 rpm, 5 min, 4 °C) using an Eppendorf 5910R tabletop centrifuge. After centrifugation, the supernatant was removed, and the cell pellet was resuspended in M9-N buffer (400 µL) by gently shaking using a Fisher Scientific microplate shaker (800 rpm, 3 min). The 24-well plate was then transferred into a Coy anaerobic chamber. In the Coy chamber, the *N*-fluoroamide substrate **1** (10 µL, 0.4 M in EtOH) and ferrous ammonium sulfate (10 µL, 40 mM in ddH_2O) were added in succession using an Eppendorf Xplorer multichannel pipette. The plates were sealed with an aluminum foil and shaken on a Corning microplate shaker at room temperature and 680 rpm. After 6–18 h, the 24-well plate was taken out of the anaerobic chamber, the seal was removed, and the analytical scale reactions were worked up following the method described above.

Procedure for analytical scale biocatalytic C–H functionalization reactions

Expression of *Mth*EgtB variants and other nonheme Fe enzymes in 125 mL Erlenmeyer flasks. *E. coli* (*E. cloni* BL21(DE3)) cells carrying the *Mth*EgtB plasmid in a pET-28b(+) vector were grown overnight in 4 mL LB_{kan}. TB_{kan} (30 mL in a 125 mL Erlenmeyer flask) was then inoculated with 1.5 mL preculture and incubated at 37 °C, 230 rpm for 2.5 h until OD_{600} reached ca. 2.0. The cultures were cooled on ice for 20 min before induction with 0.5 mM IPTG (final concentration). Expression was conducted at 22 °C, 150 rpm for 22 h. Nonheme enzyme cloned into pET-22b(+) were expressed in TB_{amp} in an analogous manner.

Biocatalytic C–H fluorination using cell-free lysates. *E. coli* cells were pelleted by centrifugation (3000 rpm, 5 min, 4 °C) using an Eppendorf 5910R tabletop centrifuge. Supernatant was removed and the resulting cell pellet was resuspended in M9-N buffer to OD_{600} = 15–60 (typically 40). *E. coli* cells were lysed by sonication using a BioLogics ultrasonic homogenizer (model 150 V/T) equipped with a stepped microtip (6 min in total, 1 sec on, 1 sec off, 45% amplitude, 2 cycles), samples were carefully submerged in wet ice to avoid overheating. (Note: overheating in the sonication step will lead to a significant loss of enzyme activity.) The resulting lysed solutions were kept on ice until further use.

The cell-free lysate of the nonheme enzyme (500 µL) was added to a 2 mL vial. In a Coy anaerobic chamber, ferrous ammonium sulfate (10 µL, 40 mM in ddH_2O), sodium ascorbate (10 µL, 400 mM in M9-N buffer) and the *N*-fluoroamide substrate **1** (10 µL, 0.4 M in EtOH) were added to 500 µL cell-free lysate in a 2 mL vial in succession. The vials were sealed and shaken on a Corning microplate shaker at room temperature and 680 rpm for 20 h. All reactions were run in triplicates and averaged total turnover numbers along with standard deviation were reported.

Biocatalytic C–H azidation using cell-free lysates. Cell-free lysates were prepared using the procedure described above. In an anaerobic chamber, ferrous ammonium sulfate (10 µL, 40 mM in ddH_2O), sodium ascorbate (10 µL, 400 mM in M9-N buffer), NaN_3 (20 µL, 3.2 M in ddH_2O)

and the *N*-fluoroamide substrate **1** (10 μL, 0.4 M in EtOH) were added to 500 μL cell-free lysate in a 2 mL vial in succession. The vials were sealed and shaken on a Corning microplate shaker at room temperature and 680 rpm for 20 h. All reactions were run in triplicates.

Chiral HPLC analysis. The reaction mixtures were quenched by the addition of 600 μL extraction solution (0.5 mM mesitylene in diisopropyl ether). The vials were vortexed vigorously to ensure good mixing and extraction. The mixture in each vial was transferred to a 1.5 mL microcentrifuge tube, and the layers were separated by centrifugation (15000 rpm, 15 min) using an Eppendorf tabletop centrifuge 5425. The organic layer (350 μL) was transferred to a 500 μL vial insert placed in a 2 mL vial. Chiral HPLC analysis was conducted using a Shimadzu i-series (66 MPa) HPLC with IPA/hexanes as the mobile phase. Chiralpak IB-N5 (4.6 mm × 25 cm, 5 micron), Chiralpak IC (4.6 mm × 25 cm, 5 micron), Chiralpak IG (4.6 mm × 25 cm, 5 micron), and Chiralpak IH (4.6 mm × 25 cm, 5 micron) columns were used for analysis.

HPLC calibration curve. Stock solutions of authentic compounds (40 mM in EtOAc) were prepared. Aliquots (120 μL, 100 μL, 80 μL, 40 μL, 20 μL, 10 μL) were transferred to individual vials, and EtOAc was removed under vacuum. Note: In our HPLC analysis, EtOAc co-eluded with the *N*-fluoroamide substrate **1** and complicated the analysis. As a result, EtOAc was removed from samples used for calibration curve. To each vial were added 500 μL M9-N buffer and 600 μL extraction solution (0.5 mM mesitylene in diisopropyl ether). The mixture was vortexed and transferred to a microcentrifuge tube. After centrifugation (15000 rpm, 15 min) using an Eppendorf tabletop centrifuge 5425, the organic layer (350 μL) was transferred to a 500 μL vial insert placed in a 2 mL vial. Chiral HPLC analysis was conducted using a Shimadzu i-series (66 MPa) HPLC with IPA/hexanes as the mobile phase. Chiralpak IB-N5 (4.6 mm × 25 cm, 5 micron), Chiralpak IC (4.6 mm × 25 cm, 5 micron) and Chiralpak IG (4.6 mm × 25 cm, 5 micron) columns were used for analysis.

Determination of nonheme enzyme concentration by SDS-PAGE analysis. SDS-PAGE analysis was performed using a Bio-Rad Mini-PROTEAN Tetra cell powered by a Bio-Rad Power Pac Basic power supply. The lysate was diluted by 40 times in M9-N buffer and centrifuged (15,000 rpm, 10 min, 4 °C) using an Eppendorf tabletop centrifuge 5424R. The resulting supernatant was used for SDS-PAGE analysis. A stock solution of bovine serum albumin (BSA, 1.0 mg/mL in M9-N buffer) was freshly prepared. A series of aliquots (10 –100 μL) of the BSA stock solution was diluted to 1000 μL in M9-N buffer as the standard solution for SDS-PAGE analysis. The nonheme enzyme samples or BSA standards (30 μL) mixed with 10 μL Bio-Rad 4× Laemmli sample buffer were incubated at 90 °C for 10 min in a Bio-Rad T100 Thermal Cycler and then cooled to room temperature. (Note: incubation in a thermalcycler with a heated lid was found to be essential to ensure excellent reproducibility. Incubation in a thermal mixer without a heated lid will lead to water condensation on PCR tube caps, resulting in a change of protein concentration.)

10 μL denatured protein sample was loaded into the wells of a Bio-Rad Mini-PROTEAN TGX Stain-Free gel. 5 μL Bio-Rad Precision Plus Protein All Blue Standards was used as the protein marker in electrophoresis. Upon the completion, the SDS-PAGE gel was stained by Coomassie blue by microwaving the gel in Bio-Rad Coomassie Brilliant Blue R-250 Staining Solution for 30 s. The gel was then destained in a destaining solution (AcOH/MeOH/ddH_2O = 1:2:7) on a rocker overnight. The gel image was acquired using a Bio-Rad Gel Doc EZ Imager, and the protein bands were analyzed by the Bio-Rad Gel Doc™ EZ System. A calibration curve was generated from the BSA standards on the same gel to determine the concentrations of *Mth*EgtB and other nonheme Fe enzymes.

II. Evaluation of Fe-dependent proteins

Table S1. Detailed information of Fe-dependent proteins descripted in the manuscript.

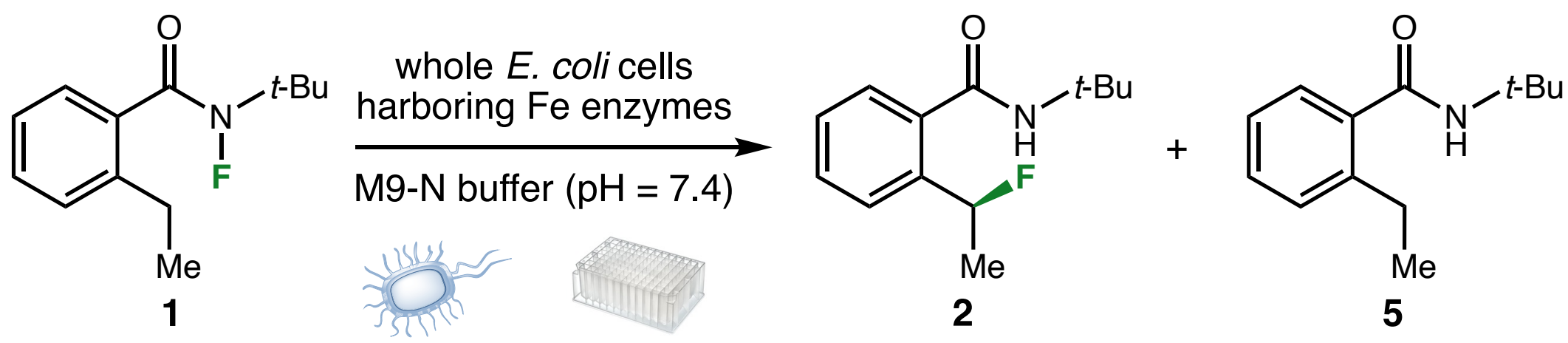

metallo protein	UniProt ID	organism	annotation	mutations
**BesD**	G8XHD5	*Streptantibioticus cattleyicolor*	2OG-dependent halogenase	none
**EvdO2**	A0A0M3KL01	*Micromonospora carbonacea*	everninomicin dioxygenase	none
**SadA D157G**	Q0B2N4	*Burkholderia ambifaria*	2OG-dependent halogenase	D157G
**WelO5**	A0A067YX61	*Hapalosiphon welwitschii*	2OG-dependent halogenase	none
**EgtB**	G7CFI3	*Mycobacterium thermoresistibile*	Ergothioneine-biosynthetic sulfoxide synthase	none
**ACCO**	Q08506	*Petunia hybrida*	1-aminocyclopropane-1-carboxylate oxidase	
**IPNS**	P05326	*Emericella nidulans*	isopenicillin N synthase	
**MPC**	P06622	*Pseudomonas putida*	metapyrocatechase	none

III. Protein and DNA sequences

Protein sequences

***Mth*EgtB**

MGSSHHHHHHSSGLVPRGSHMTGVAVPHRAELARQLIDARNRTLRLVDFDDAELRRQYDPLMSP
LVWDLAHIGQQEELWLLRGGDPRRPGLLEPAVEQLYDAFVHPRASRVHLPLLSPAQARRFCATV
RSAVLDALDRLPEDADTFAFGMVVSHEHQHDETMLQALNLRSGEPLLGSGTALPPGRPGVAGTS
VLVPGGPFVLGVDLADEPYALDNERPAHVVDVPAFRIGRVPVTNAEWRAFIDDGGYRQRRWWSD
AGWAYRCEAGLTAPQFWNPDGTRTRFGHVEDIPPDEPVQHVTYFEAEAYAAWAGARLPTEIEWE
KACAWDPATGRRRRYPWGDAAPTAALANLGGDALRPAPVGAYPAGASACGAEQMLGDVWEWTSS
PLRPWPGFTPMIYQRYSQPFFEGAGSGDYRVLRGGSWAVAADILRPSFRNWDHPIRRQIFAGVR
LAWDVDRQTARPGPVGGC*

***Mth*EgtB Y377W W415R R87D T141I F83Y R379E V84R H417L (EgtB$_{\mathrm{CHF1}}$)**

MGSSHHHHHHSSGLVPRGSHMTGVAVPHRAELARQLIDARNRTLRLVDFDDAELRRQYDPLMSP
LVWDLAHIGQQEELWLLRGGDPRRPGLLEPAVEQLYDA**YR**HP**D**ASRVHLPLLSPAQARRFCATV
RSAVLDALDRLPEDADTFAFGMVVSHEHQHDE**I**MLQALNLRSGEPLLGSGTALPPGRPGVAGTS
VLVPGGPFVLGVDLADEPYALDNERPAHVVDVPAFRIGRVPVTNAEWRAFIDDGGYRQRRWWSD
AGWAYRCEAGLTAPQFWNPDGTRTRFGHVEDIPPDEPVQHVTYFEAEAYAAWAGARLPTEIEWE
KACAWDPATGRRRRYPWGDAAPTAALANLGGDALRPAPVGAYPAGASACGAEQMLGDVWEWTSS
PLRPWPGFTPMI**W**Q**E**YSQPFFEGAGSGDYRVLRGGSWAVAADILRPSFRN**R**D**L**PIRRQIFAGVR
LAWDVDRQTARPGPVGGC*

***Mth*EgtB Y377W W415R R87D T141I F83Y R379E V84R H417L H138A (EgtB$_{\mathrm{CHF1}}$ H138A)**

MGSSHHHHHHSSGLVPRGSHMTGVAVPHRAELARQLIDARNRTLRLVDFDDAELRRQYDPLMSP
LVWDLAHIGQQEELWLLRGGDPRRPGLLEPAVEQLYDA**YR**HP**D**ASRVHLPLLSPAQARRFCATV
RSAVLDALDRLPEDADTFAFGMVVSHEHQ**A**DE**I**MLQALNLRSGEPLLGSGTALPPGRPGVAGTS

VLVPGGPFVLGVDLADEPYALDNERPAHVVDVPAFRIGRVPVTNAEWRAFIDDGGYRQRRWWSD
AGWAYRCEAGLTAPQFWNPDGTRTRFGHVEDIPPDEPVQHVTYFEAEAYAAWAGARLPTEIEWE
KACAWDPATGRRRRYPWGDAAPTAALANLGGDALRPAPVGAYPAGASACGAEQMLGDVWEWTSS
PLRPWPGFTPMI**W**Q**E**YSQPFFEGAGSGDYRVLRGGSWAVAADILRPSFRN**R**D**L**PIRRQIFAGVR
LAWDVDRQTARPGPVGGC*

***Mth*EgtB Y377W W415R R87D T141M R379A A145K Q422D (EgtB$_{CHF2}$)**

<u>MGSSHHHHHHSSGLVPRGSH</u>MTGVAVPHRAELARQLIDARNRTLRLVDFDDAELRRQYDPLMSP
LVWDLAHIGQQEELWLLRGGDPRRPGLLEPAVEQLYDAFVHP**D**ASRVHLPLLSPAQARRFCATV
RSAVLDALDRLPEDADTFAFGMVVSHEHQHDE**M**MLQ**K**LNLRSGEPLLGSGTALPPGRPGVAGTS
VLVPGGPFVLGVDLADEPYALDNERPAHVVDVPAFRIGRVPVTNAEWRAFIDDGGYRQRRWWSD
AGWAYRCEAGLTAPQFWNPDGTRTRFGHVEDIPPDEPVQHVTYFEAEAYAAWAGARLPTEIEWE
KACAWDPATGRRRRYPWGDAAPTAALANLGGDALRPAPVGAYPAGASACGAEQMLGDVWEWTSS
PLRPWPGFTPMI**W**Q**A**YSQPFFEGAGSGDYRVLRGGSWAVAADILRPSFRN**R**DHPIRR**D**IFAGVR
LAWDVDRQTARPGPVGGC*

***Mth*EgtB Y377W W415R R87D T141M R379A A145K Q422D H138A (EgtB$_{CHF2}$ H138A)**

<u>MGSSHHHHHHSSGLVPRGSH</u>MTGVAVPHRAELARQLIDARNRTLRLVDFDDAELRRQYDPLMSP
LVWDLAHIGQQEELWLLRGGDPRRPGLLEPAVEQLYDAFVHP**D**ASRVHLPLLSPAQARRFCATV
RSAVLDALDRLPEDADTFAFGMVVSHEHQ**A**DE**M**MLQ**K**LNLRSGEPLLGSGTALPPGRPGVAGTS
VLVPGGPFVLGVDLADEPYALDNERPAHVVDVPAFRIGRVPVTNAEWRAFIDDGGYRQRRWWSD
AGWAYRCEAGLTAPQFWNPDGTRTRFGHVEDIPPDEPVQHVTYFEAEAYAAWAGARLPTEIEWE
KACAWDPATGRRRRYPWGDAAPTAALANLGGDALRPAPVGAYPAGASACGAEQMLGDVWEWTSS
PLRPWPGFTPMI**W**Q**A**YSQPFFEGAGSGDYRVLRGGSWAVAADILRPSFRN**R**DHPIRR**D**IFAGVR
LAWDVDRQTARPGPVGGC*

ACCO I184A K158I F91L K172Y K93Q T89A (ACCO$_{CHF}$):

MGSSHHHHHHSSGLVPRGSHMENFPIISLDKVNGVERAATMEMIKDACENWGFFELVNHGIPRE

VMDTVEKMTKGHYKKCMEQRFKELVASKALEGVQAEVTDMDWES**A**F**L**L**Q**HLPISNISEVPDLDE

EYREVMRDFAKRLEKLAEELLDLLCENLGLEKGYLKNAFYGSKGPNFGT**I**VSNYPPCPKPDLI**Y**

GLRAHTDAGGI**A**LLFQDDKVSGLQLLKDGQWIDVPPMRHSIVVNLGDQLEVITNGKYKSVMHRV

IAQKDGARMSLASFYNPGSDAVIYPAPALVEKEAEENKQVYPKFVFDDYMKLYAGLKFQAKEPR

FEAMKAMETDVKMDPIATV*

DNA sequences

***Mth*EgtB**

ATGGGCAGCAGCCATCATCATCATCATCACAGCAGCGGCCTGGTGCCGCGCGGCAGCCATATGA

CTGGAGTAGCAGTGCCCCACCGTGCAGAATTGGCCCGTCAGTTAATTGACGCTCGCAATCGTAC

CCTGCGTCTTGTTGATTTCGATGACGCCGAGCTGCGCCGCCAGTATGACCCTCTTATGAGCCCC

TTGGTATGGGACTTGGCTCATATTGGACAGCAGGAGGAGTTATGGTTGCTTCGTGGAGGCGACC

CCCGTCGCCCAGGGCTGTTAGAGCCAGCAGTTGAGCAGTTATACGACGCATTCGTACATCCACG

TGCCTCCCGCGTCCACCTTCCGTTGTTATCTCCCGCTCAAGCCCGCCGCTTCTGTGCTACCGTG

CGTAGCGCGGTTCTGGACGCTTTAGACCGTCTTCCTGAAGATGCCGATACGTTCGCCTTCGGGA

TGGTCGTCTCGCACGAACACCAGCACGATGAGACGATGTTGCAAGCGCTGAATTTACGCTCAGG

TGAGCCCTTGCTTGGGTCTGGGACGGCGTTACCTCCGGGTCGCCCTGGCGTCGCGGGCACAAGC

GTTTTAGTACCTGGAGGTCCGTTTGTGCTGGGTGTCGATTTGGCTGACGAGCCATACGCATTAG

ATAACGAACGTCCGGCCCACGTAGTAGATGTGCCAGCGTTCCGTATCGGTCGCGTGCCAGTGAC

AAACGCTGAGTGGCGTGCATTTATCGACGATGGTGGATATCGCCAACGTCGTTGGTGGTCGGAT

GCTGGTTGGGCTTATCGCTGTGAAGCCGGGCTTACCGCCCCGCAGTTCTGGAATCCCGATGGCA

CTCGTACCCGCTTCGGACATGTAGAGGATATTCCCCCCGATGAGCCAGTTCAACACGTCACTTA

TTTTGAAGCAGAGGCTTATGCAGCATGGGCAGGGGCGCGCTTACCCACGGAAATTGAGTGGGAG

AAGGCTTGCGCCTGGGACCCTGCCACAGGTCGTCGCCGCCGCTACCCCTGGGGAGACGCGGCTC

CCACTGCGGCACTTGCGAATCTTGGTGGCGACGCGTTGCGTCCAGCGCCTGTAGGAGCCTATCC

TGCGGGGGCAAGCGCGTGTGGAGCTGAGCAGATGTTGGGGGATGTTTGGGAATGGACTTCAAGT

CCGCTGCGCCCATGGCCGGGGTTTACACCGATGATCTATCAGCGCTACTCTCAACCATTTTTTG

AGGGAGCAGGCAGCGGAGACTACCGTGTATTGCGTGGGGGCAGTTGGGCAGTCGCTGCTGACAT

CCTTCGTCCGTCATTCCGCAATTGGGACCACCCCATTCGTCGTCAAATTTTCGCAGGGGTCCGT

CTGGCGTGGGACGTTGACCGTCAGACGGCTCGTCCCGGACCAGTGGGCGGATGTTGA

*Mth*EgtB Y377W W415R R87D T141I F83Y R379E V84R H417L (EgtB$_{CHF1}$)

ATGGGCAGCAGCCATCATCATCATCATCACAGCAGCGGCCTGGTGCCGCGCGGCAGCCATATGA

CTGGAGTAGCAGTGCCCCACCGTGCAGAATTGGCCCGTCAGTTAATTGACGCTCGCAATCGTAC

CCTGCGTCTTGTTGATTTCGATGACGCCGAGCTGCGCCGCCAGTATGACCCTCTTATGAGCCCC

TTGGTATGGGACTTGGCTCATATTGGACAGCAGGAGGAGTTATGGTTGCTTCGTGGAGGCGACC

CCCGTCGCCCAGGGCTGTTAGAGCCAGCAGTTGAGCAGTTATACGACGCA**TATCGT**CATCCA**GA**

TGCCTCCCGCGTCCACCTTCCGTTGTTATCTCCCGCTCAAGCCCGCCGCTTCTGTGCTACCGTG

CGTAGCGCGGTTCTGGACGCTTTAGACCGTCTTCCTGAAGATGCCGATACGTTCGCCTTCGGGA

TGGTCGTCTCGCACGAACACCAGCACGATGAG**ATT**ATGTTGCAAGCGCTGAATTTACGCTCAGG

TGAGCCCTTGCTTGGGTCTGGGACGGCGTTACCTCCGGGTCGCCCTGGCGTCGCGGGCACAAGC

GTTTTAGTACCTGGAGGTCCGTTTGTGCTGGGTGTCGATTTGGCTGACGAGCCATACGCATTAG

ATAACGAACGTCCGGCCCACGTAGTAGATGTGCCAGCGTTCCGTATCGGTCGCGTGCCAGTGAC

AAACGCTGAGTGGCGTGCATTTATCGACGATGGTGGATATCGCCAACGTCGTTGGTGGTCGGAT

GCTGGTTGGGCTTATCGCTGTGAAGCCGGGCTTACCGCCCCGCAGTTCTGGAATCCCGATGGCA

CTCGTACCCGCTTCGGACATGTAGAGGATATTCCCCCCGATGAGCCAGTTCAACACGTCACTTA

TTTTGAAGCAGAGGCTTATGCAGCATGGGCAGGGGCGCGCTTACCCACGGAAATTGAGTGGGAG

AAGGCTTGCGCCTGGGACCCTGCCACAGGTCGTCGCCGCCGCTACCCCTGGGGAGACGCGGCTC

CCACTGCGGCACTTGCGAATCTTGGTGGCGACGCGTTGCGTCCAGCGCCTGTAGGAGCCTATCC

TGCGGGGGCAAGCGCGTGTGGAGCTGAGCAGATGTTGGGGGATGTTTGGGAATGGACTTCAAGT

CCGCTGCGCCCATGGCCGGGGTTTACACCGATGATC**TGG**CAG**GAG**TACTCTCAACCATTTTTTG

AGGGAGCAGGCAGCGGAGACTACCGTGTATTGCGTGGGGGCAGTTGGGCAGTCGCTGCTGACAT

CCTTCGTCCGTCATTCCGCAAT**CGT**GAC**CTG**CCCATTCGTCGTCAAATTTTCGCAGGGGTCCGT
CTGGCGTGGGACGTTGACCGTCAGACGGCTCGTCCCGGACCAGTGGGCGGATGTTGA

*Mth*EgtB Y377W W415R R87D T141M R379A A145K Q422D (EgtB$_{CHF2}$)

ATGGGCAGCAGCCATCATCATCATCATCACAGCAGCGGCCTGGTGCCGCGCGGCAGCCATATGA
CTGGAGTAGCAGTGCCCCACCGTGCAGAATTGGCCCGTCAGTTAATTGACGCTCGCAATCGTAC
CCTGCGTCTTGTTGATTTCGATGACGCCGAGCTGCGCCGCCAGTATGACCCTCTTATGAGCCCC
TTGGTATGGGACTTGGCTCATATTGGACAGCAGGAGGAGTTATGGTTGCTTCGTGGAGGCGACC
CCCGTCGCCCAGGGCTGTTAGAGCCAGCAGTTGAGCAGTTATACGACGCATTCGTACATCCA**GA**
TGCCTCCCGCGTCCACCTTCCGTTGTTATCTCCCGCTCAAGCCCGCCGCTTCTGTGCTACCGTG
CGTAGCGCGGTTCTGGACGCTTTAGACCGTCTTCCTGAAGATGCCGATACGTTCGCCTTCGGGA
TGGTCGTCTCGCACGAACACCAGCACGATGAG**ATG**ATGTTGCAA**AAG**CTGAATTTACGCTCAGG
TGAGCCCTTGCTTGGGTCTGGGACGGCGTTACCTCCGGGTCGCCCTGGCGTCGCGGGCACAAGC
GTTTTAGTACCTGGAGGTCCGTTTGTGCTGGGTGTCGATTTGGCTGACGAGCCATACGCATTAG
ATAACGAACGTCCGGCCCACGTAGTAGATGTGCCAGCGTTCCGTATCGGTCGCGTGCCAGTGAC
AAACGCTGAGTGGCGTGCATTTATCGACGATGGTGGATATCGCCAACGTCGTTGGTGGTCGGAT
GCTGGTTGGGCTTATCGCTGTGAAGCCGGGCTTACCGCCCCGCAGTTCTGGAATCCCGATGGCA
CTCGTACCCGCTTCGGACATGTAGAGGATATTCCCCCCGATGAGCCAGTTCAACACGTCACTTA
TTTTGAAGCAGAGGCTTATGCAGCATGGGCAGGGGCGCGCTTACCCACGGAAATTGAGTGGGAG
AAGGCTTGCGCCTGGGACCCTGCCACAGGTCGTCGCCGCCGCTACCCCTGGGGAGACGCGGCTC
CCACTGCGGCACTTGCGAATCTTGGTGGCGACGCGTTGCGTCCAGCGCCTGTAGGAGCCTATCC
TGCGGGGGCAAGCGCGTGTGGAGCTGAGCAGATGTTGGGGGATGTTTGGGAATGGACTTCAAGT
CCGCTGCGCCCATGGCCGGGGTTTACACCGATGATC**TGG**CAG**GCG**TACTCTCAACCATTTTTTG
AGGGAGCAGGCAGCGGAGACTACCGTGTATTGCGTGGGGGCAGTTGGGCAGTCGCTGCTGACAT
CCTTCGTCCGTCATTCCGCAAT**CGT**GACCACCCCATTCGTCGT**GAT**ATTTTCGCAGGGGTCCGT
CTGGCGTGGGACGTTGACCGTCAGACGGCTCGTCCCGGACCAGTGGGCGGATGTTGA

_Mth_EgtB Y377W W415R R87D T141I F83Y R379E V84R H417L H138A (EgtB$_{CHF1}$ H138A)

ATGGGCAGCAGCCATCATCATCATCATCACAGCAGCGGCCTGGTGCCGCGCGGCAGCCATATGA
CTGGAGTAGCAGTGCCCCACCGTGCAGAATTGGCCCGTCAGTTAATTGACGCTCGCAATCGTAC
CCTGCGTCTTGTTGATTTCGATGACGCCGAGCTGCGCCGCCAGTATGACCCTCTTATGAGCCCC
TTGGTATGGGACTTGGCTCATATTGGACAGCAGGAGGAGTTATGGTTGCTTCGTGGAGGCGACC
CCCGTCGCCCAGGGCTGTTAGAGCCAGCAGTTGAGCAGTTATACGACGCA**TATCGT**CATCCA**GA**
TGCCTCCCGCGTCCACCTTCCGTTGTTATCTCCCGCTCAAGCCCGCCGCTTCTGTGCTACCGTG
CGTAGCGCGGTTCTGGACGCTTTAGACCGTCTTCCTGAAGATGCCGATACGTTCGCCTTCGGGA
TGGTCGTCTCGCACGAACACCAG**GCG**GATGAG**ATT**ATGTTGCAAGCGCTGAATTTACGCTCAGG
TGAGCCCTTGCTTGGGTCTGGGACGGCGTTACCTCCGGGTCGCCCTGGCGTCGCGGGCACAAGC
GTTTTAGTACCTGGAGGTCCGTTTGTGCTGGGTGTCGATTTGGCTGACGAGCCATACGCATTAG
ATAACGAACGTCCGGCCCACGTAGTAGATGTGCCAGCGTTCCGTATCGGTCGCGTGCCAGTGAC
AAACGCTGAGTGGCGTGCATTTATCGACGATGGTGGATATCGCCAACGTCGTTGGTGGTCGGAT
GCTGGTTGGGCTTATCGCTGTGAAGCCGGGCTTACCGCCCCGCAGTTCTGGAATCCCGATGGCA
CTCGTACCCGCTTCGGACATGTAGAGGATATTCCCCCCGATGAGCCAGTTCAACACGTCACTTA
TTTTGAAGCAGAGGCTTATGCAGCATGGGCAGGGGCGCGCTTACCCACGGAAATTGAGTGGGAG
AAGGCTTGCGCCTGGGACCCTGCCACAGGTCGTCGCCGCCGCTACCCCTGGGGAGACGCGGCTC
CCACTGCGGCACTTGCGAATCTTGGTGGCGACGCGTTGCGTCCAGCGCCTGTAGGAGCCTATCC
TGCGGGGGCAAGCGCGTGTGGAGCTGAGCAGATGTTGGGGGATGTTTGGGAATGGACTTCAAGT
CCGCTGCGCCCATGGCCGGGGTTTACACCGATGATC**TGG**CAG**GAG**TACTCTCAACCATTTTTTG
AGGGAGCAGGCAGCGGAGACTACCGTGTATTGCGTGGGGGCAGTTGGGCAGTCGCTGCTGACAT
CCTTCGTCCGTCATTCCGCAAT**CGT**GAC**CTG**CCCATTCGTCGTCAAATTTTCGCAGGGGTCCGT
CTGGCGTGGGACGTTGACCGTCAGACGGCTCGTCCCGGACCAGTGGGCGGATGTTGA

_Mth_EgtB Y377W W415R R87D T141M R379A A145K Q422D H138A (EgtB$_{CHF2}$ H138A)

ATGGGCAGCAGCCATCATCATCATCATCACAGCAGCGGCCTGGTGCCGCGCGGCAGCCATATGA
CTGGAGTAGCAGTGCCCCACCGTGCAGAATTGGCCCGTCAGTTAATTGACGCTCGCAATCGTAC
CCTGCGTCTTGTTGATTTCGATGACGCCGAGCTGCGCCGCCAGTATGACCCTCTTATGAGCCCC
TTGGTATGGGACTTGGCTCATATTGGACAGCAGGAGGAGTTATGGTTGCTTCGTGGAGGCGACC
CCCGTCGCCCAGGGCTGTTAGAGCCAGCAGTTGAGCAGTTATACGACGCATTCGTACATCCA**GA**
TGCCTCCCGCGTCCACCTTCCGTTGTTATCTCCCGCTCAAGCCCGCCGCTTCTGTGCTACCGTG
CGTAGCGCGGTTCTGGACGCTTTAGACCGTCTTCCTGAAGATGCCGATACGTTCGCCTTCGGGA
TGGTCGTCTCGCACGAACACCAG**GCG**GATGAG**ATG**ATGTTGCAA**AAG**CTGAATTTACGCTCAGG
TGAGCCCTTGCTTGGGTCTGGGACGGCGTTACCTCCGGGTCGCCCTGGCGTCGCGGGCACAAGC
GTTTTAGTACCTGGAGGTCCGTTTGTGCTGGGTGTCGATTTGGCTGACGAGCCATACGCATTAG
ATAACGAACGTCCGGCCCACGTAGTAGATGTGCCAGCGTTCCGTATCGGTCGCGTGCCAGTGAC
AAACGCTGAGTGGCGTGCATTTATCGACGATGGTGGATATCGCCAACGTCGTTGGTGGTCGGAT
GCTGGTTGGGCTTATCGCTGTGAAGCCGGGCTTACCGCCCCGCAGTTCTGGAATCCCGATGGCA
CTCGTACCCGCTTCGGACATGTAGAGGATATTCCCCCCGATGAGCCAGTTCAACACGTCACTTA
TTTTGAAGCAGAGGCTTATGCAGCATGGGCAGGGGCGCGCTTACCCACGGAAATTGAGTGGGAG
AAGGCTTGCGCCTGGGACCCTGCCACAGGTCGTCGCCGCCGCTACCCCTGGGGAGACGCGGCTC
CCACTGCGGCACTTGCGAATCTTGGTGGCGACGCGTTGCGTCCAGCGCCTGTAGGAGCCTATCC
TGCGGGGGCAAGCGCGTGTGGAGCTGAGCAGATGTTGGGGGATGTTTGGGAATGGACTTCAAGT
CCGCTGCGCCCATGGCCGGGGTTTACACCGATGATC**TGG**CAG**GCG**TACTCTCAACCATTTTTTG
AGGGAGCAGGCAGCGGAGACTACCGTGTATTGCGTGGGGGCAGTTGGGCAGTCGCTGCTGACAT
CCTTCGTCCGTCATTCCGCAAT**CGT**GACCACCCCATTCGTCGT**GAT**ATTTTCGCAGGGGTCCGT
CTGGCGTGGGACGTTGACCGTCAGACGGCTCGTCCCGGACCAGTGGGCGGATGTTGA

ACCO I184A K158I F91L K172Y K93Q T89A (ACCO$_{CHF}$)

ATGGGCAGCAGCCATCATCATCATCATCACAGCAGCGGCCTGGTGCCGCGCGGCAGCCATATGG
AAAACTTCCCGATTATTAGTCTGGATAAAGTTAATGGTGTGGAACGCGCCGCCACAATGGAAAT
GATTAAGGATGCCTGTGAAAATTGGGGCTTTTTCGAACTGGTTAATCATGGCATTCCGCGTGAA

GTGATGGATACCGTGGAAAAAATGACCAAAGGCCATTATAAAAAGTGCATGGAACAGCGTTT**CT**

GAGAACTGGTTGCAAGTAAAGCCCTGGAAGGCGTGCAGGCAGAAGTTACCGATATGGATTGGGA

AAGC**GCG**TTTTTCCTG**CAG**CATCTGCCGATTAGTAATATTAGCGAAGTGCCGGATCTGGATGAA

GAATATCGTGAAGTGATGCGCGATTTTGCAAAACGTCTGGAAAAACTGGCCGAAGAACTGCTGG

ATCTGCTGTGTGAAAATCTGGGCCTGGAAAAAGGTTATCTGAAAAATGCATTTTACGGTAGTAA

GGGTCCGAATTTTGGTACA**ATT**GTTAGCAATTATCCGCCGTGTCCGAAACCGGATCTGATT**TAT**

GGTCTGCGTGCACATACCGATGCAGGTGGTATT**GCG**CTGCTGTTTCAGGATGATAAAGTGAGCG

GCCTGCAGCTGCTGAAAGATGGTCAGTGGATTGATGTTCCGCCGATGCGCCATAGTATTGTTGT

TAATCTGGGTGACCAGCTGGAAGTTATTACCAATGGCAAATATAAAAGCGTTATGCATCGTGTG

ATTGCCCAGAAAGATGGCGCACGCATGAGTCTGGCCAGTTTTTATAATCCGGGCAGCGATGCCG

TTATCTATCCGGCCCCGGCACTGGTTGAAAAAGAAGCCGAAGAAAATAAGCAGGTTTATCCGAA

ATTTGTGTTTGATGATTACATGAAACTGTACGCCGGCCTGAAATTTCAGGCAAAAGAACCGCGC

TTTGAAGCAATGAAAGCAATGGAAACCGATGTTAAAATGGACCCTATTGCAACCGTGTAA

Table S2. Primers used for the directed evolution of $EgtB_{CHF}$ and $ACCO_{CHF}$ via site-saturation mutagenesis.

DNA Template	name	5'-3' primer sequences
pET-28b(+)	HindIII_fwd	GAAAGCTTGCGGCCGCACTCGAG
	HindIII_rev	CTCGAGTGCGGCCGCAAGCTTTCAACATCCGCCCACTGGTCC
	NdeI_fwd	GTGCCGCGCGGCAGCCATATGACTGGAGTAGCAGTGCCCCAC
	NdeI_rev	CATATGGCTGCCGCGCGGCACCAG
*Mth*EgtB	Y377X_NDT	CATGGCCGGGGTTTACACCGATGATC**NDT**CAGCGCTACTC
	Y377X_VHG	CATGGCCGGGGTTTACACCGATGATC**VHG**CAGCGCTACTC
	Y377X_TGG	CATGGCCGGGGTTTACACCGATGATC**TGG**CAGCGCTACTC
	Y377X_rev	GATCATCGGTGTAAACCCCGGCCATG
*Mth*EgtB Y377W	W415X_NDT	CTTCGTCCGTCATTCCGCAAT**NDT**GACCACCCCATTC
	W415X_VHG	CTTCGTCCGTCATTCCGCAAT**VHG**GACCACCCCATTC
	W415X_rev	TTGCGGAATGACGGACGAAGGATGTCAG
*Mth*EgtB Y377W W415R	R87X_NDT	ATACGACGCATTCGTACATCCA**NDT**GCCTCCCGCGTC
	R87X_VHG	ATACGACGCATTCGTACATCCA**VHG**GCCTCCCGCGTC
	R87X_TGG	ATACGACGCATTCGTACATCCA**TGG**GCCTCCCGCGTC
	R87X_rev	GATGTACGAATGCGTCGTATAACTGCTCAAC
*Mth*EgtB Y377W W415R R87D	T141X_NDT	CACGAACACCAGCACGATGAG**NDT**ATGTTGCAAGC
	T141X_VHG	CACGAACACCAGCACGATGAG**VHG**ATGTTGCAAGC
	T141X_TGG	CACGAACACCAGCACGATGAG**TGG**ATGTTGCAAGC
	T141X_rev	CTCATCGTGCTGGTGTTCGTGCGAGACG
*Mth*EgtB Y377W W415R R87D T141I	F83X_NDT	CAGTTGAGCAGTTATACGACGCA**NDT**GTACATCCAC
	F83X_VHG	CAGTTGAGCAGTTATACGACGCA**VHG**GTACATCCAC
	F83X_TGG	CAGTTGAGCAGTTATACGACGCA**TGG**GTACATCCAC
	F83X_rev	GTCGTATAACTGCTCAACTGCTGGCTC

*Mth*EgtB Y377W W415R R87D T141I F83Y	R379X_NDT	GTTTACACCGATGATCTGGCAG**NDT**TACTCTCAAC
	R379X_VHG	GTTTACACCGATGATCTGGCAG**VHG**TACTCTCAAC
	R379X_TGG	GTTTACACCGATGATCTGGCAG**TGG**TACTCTCAAC
	R379X_rev	CAGATCATCGGTGTAAACCCCGGCCATG
*Mth*EgtB Y377W W415R R87D T141I F83Y R379E	V84X_NDT	CAGTTGAGCAGTTATACGACGCATAT**NDT**CATCCAGATG
	V84X_VHG	CAGTTGAGCAGTTATACGACGCATAT**VHG**CATCCAGATG
	V84X_TGG	CAGTTGAGCAGTTATACGACGCATAT**TGG**CATCCAGATG
	V84X_rev	CGTCGTATAACTGCTCAACTGCTG
*Mth*EgtB Y377W W415R R87D T141I F83Y R379E V84R	H417X_NDT	CGTCCGTCATTCCGCAATCGTGAC**NDT**CCCATTCG
	H417X_VHG	CGTCCGTCATTCCGCAATCGTGAC**VHG**CCCATTCG
	H417X_TGG	CGTCCGTCATTCCGCAATCGTGAC**TGG**CCCATTCG
	H417X_rev	GATTGCGGAATGACGGACGAAGGATGTC
*Mth*EgtB Y377W W415R R87D T141M	R379X_NDT	GTTTACACCGATGATCTGGCAG**NDT**TACTCTCAAC
	R379X_VHG	GTTTACACCGATGATCTGGCAG**VHG**TACTCTCAAC
	R379X_TGG	GTTTACACCGATGATCTGGCAG**TGG**TACTCTCAAC
	R379X_rev	CAGATCATCGGTGTAAACCCCGGCCATG
*Mth*EgtB Y377W W415R R87D T141M R379A	A145X_NDT	CGATGAGATGATGTTGCAA**NDT**CTGAATTTAC
	A145X_VHG	CGATGAGATGATGTTGCAA**VHG**CTGAATTTAC
	A145X_TGG	CGATGAGATGATGTTGCAA**TGG**CTGAATTTAC
	A145X_rev	TTGCAACATCATCTCATCGTGCTGGTG
*Mth*EgtB Y377W W415R R87D T141M R379A A145K	Q422X_NDT	CGTGACCACCCCATTCGTCGT**NDT**ATTTTCGCAG
	Q422X_VHG	CGTGACCACCCCATTCGTCGT**VHG**ATTTTCGCAG
	Q422X_TGG	CGTGACCACCCCATTCGTCGT**TGG**ATTTTCGCAG
	Q422X_rev	ACGACGAATGGGGTGGTCACGATTG

*Mth*EgtB Y377W W415R R87D T141I F83Y R379E V84R H417L (*Mth*EgtB$_{CHF1}$)	H51X_rev	AGCCAAGTCCCATACCAAGG
	H51D_fwd	CCTTGGTATGGGACTTGGCT**GAT**ATTGGACAGC
	H51E_fwd	CCTTGGTATGGGACTTGGCT**GAA**ATTGGACAGC
	H51A_fwd	CCTTGGTATGGGACTTGGCT**GCG**ATTGGACAGC
	H134X_rev	CGAGACGACCATCCCGAAGG
	H134D_fwd	CCTTCGGGATGGTCGTCTCG**GAT**GAACACCAGC
	H134E_fwd	CCTTCGGGATGGTCGTCTCG**GAA**GAACACCAGC
	H134A_fwd	CCTTCGGGATGGTCGTCTCG**GCG**GAACACCAGC
	H138X_rev	CTGGTGTTCGTGCGAGACGAC
	H138A_fwd	GTCGTCTCGCACGAACACCAG**GCG**GATGAGATTATG
	H138D_fwd	GTCGTCTCGCACGAACACCAG**GAT**GATGAGATTATG
	H138E_fwd	GTCGTCTCGCACGAACACCAG**GAA**GATGAGATTATG
*Mth*EgtB Y377W W415R R87D T141M R379A A145K H417L (*Mth*EgtB$_{CHF2}$)	H51X_rev	AGCCAAGTCCCATACCAAGG
	H51D_fwd	CCTTGGTATGGGACTTGGCT**GAT**ATTGGACAGC
	H51E_fwd	CCTTGGTATGGGACTTGGCT**GAA**ATTGGACAGC
	H51A_fwd	CCTTGGTATGGGACTTGGCT**GCG**ATTGGACAGC
	H134X_rev	CGAGACGACCATCCCGAAGG
	H134D_fwd	CCTTCGGGATGGTCGTCTCG**GAT**GAACACCAGC
	H134E_fwd	CCTTCGGGATGGTCGTCTCG**GAA**GAACACCAGC
	H134E_fwd	CCTTCGGGATGGTCGTCTCG**GCG**GAACACCAGC
	H138X_rev	CTGGTGTTCGTGCGAGACGAC
	H138A_fwd	GTCGTCTCGCACGAACACCAG**GCG**GATGAGATGATG
	H138D_fwd	GTCGTCTCGCACGAACACCAG**GAT**GATGAGATGATG
	H138E_fwd	GTCGTCTCGCACGAACACCAG**GAA**GATGAGATGATG
ACCO I184A K158I	D179X_rev	GGTATGTGCACGCAGACCATAAATCAGATCC
	D179E_fwd	ATGGTCTGCGTGCACATACC**GAA**GCAGGTGGTATTG

K172V K93Q T89A (ACCO$_{CHF}$)	D179A_fwd	ATGGTCTGCGTGCACATACC**GCG**GCAGGTGGTATTG
	D179H_fwd	ATGGTCTGCGTGCACATACC**CAT**GCAGGTGGTATTG

IV. Determination of nonheme enzyme concentrations

1. Determination of enzyme concentrations by SDS-PAGE analysis.

A representative example using SDS-PAGE analysis to determine the concentration of nonheme enzymes in the cell-free lysate is provided below. Typical concentrations of the entire $EgtB_{CHF}$ lineage are provided in Table **S3**.

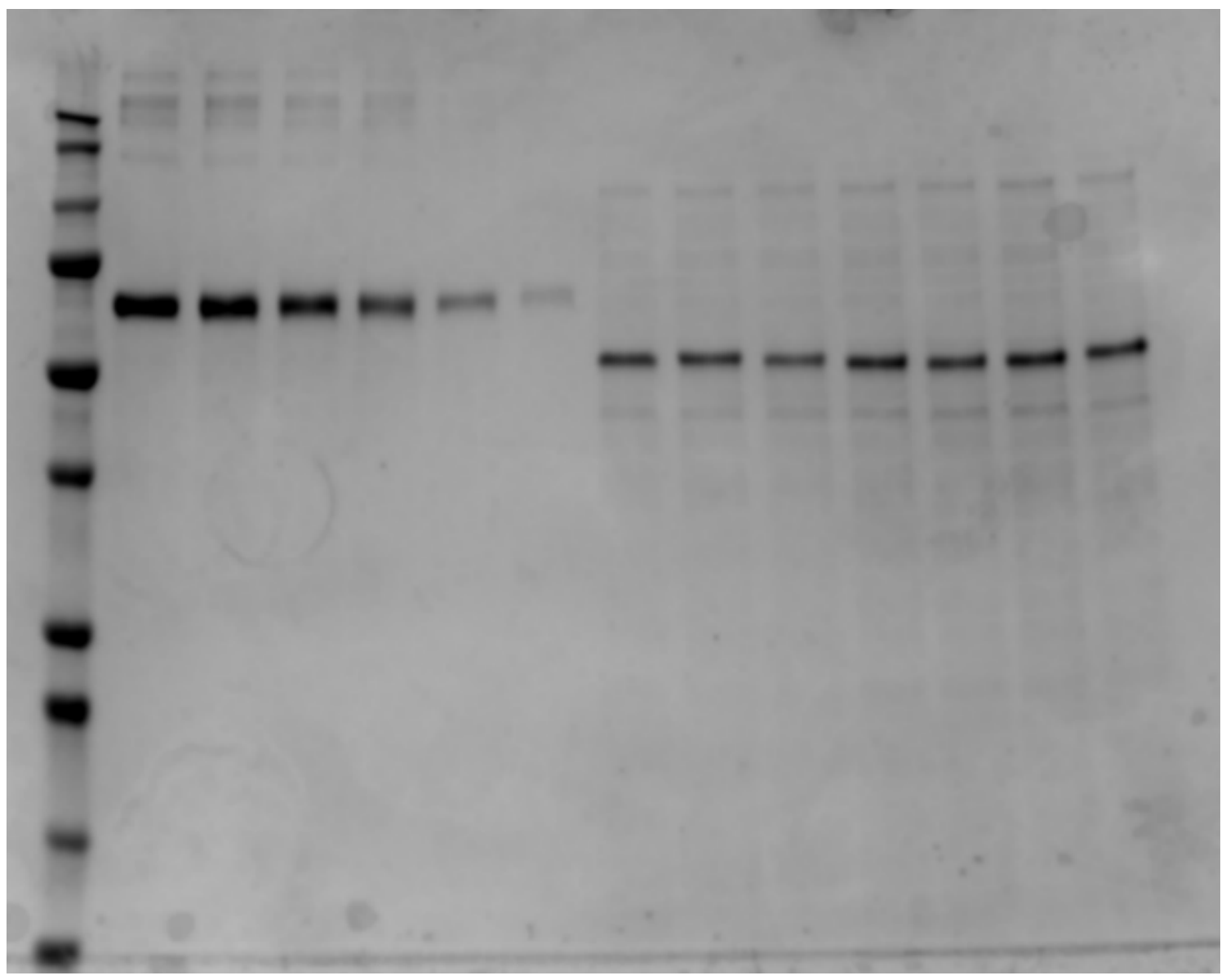

Figure S1. SDS-PAGE analysis for the determination of *Mth*EgtB concentration in cell-free lysates (Lane 1: protein ladder, Lanes 2-7: BSA standards, Lane 8-14: $EgtB_{CHF}$ variants).

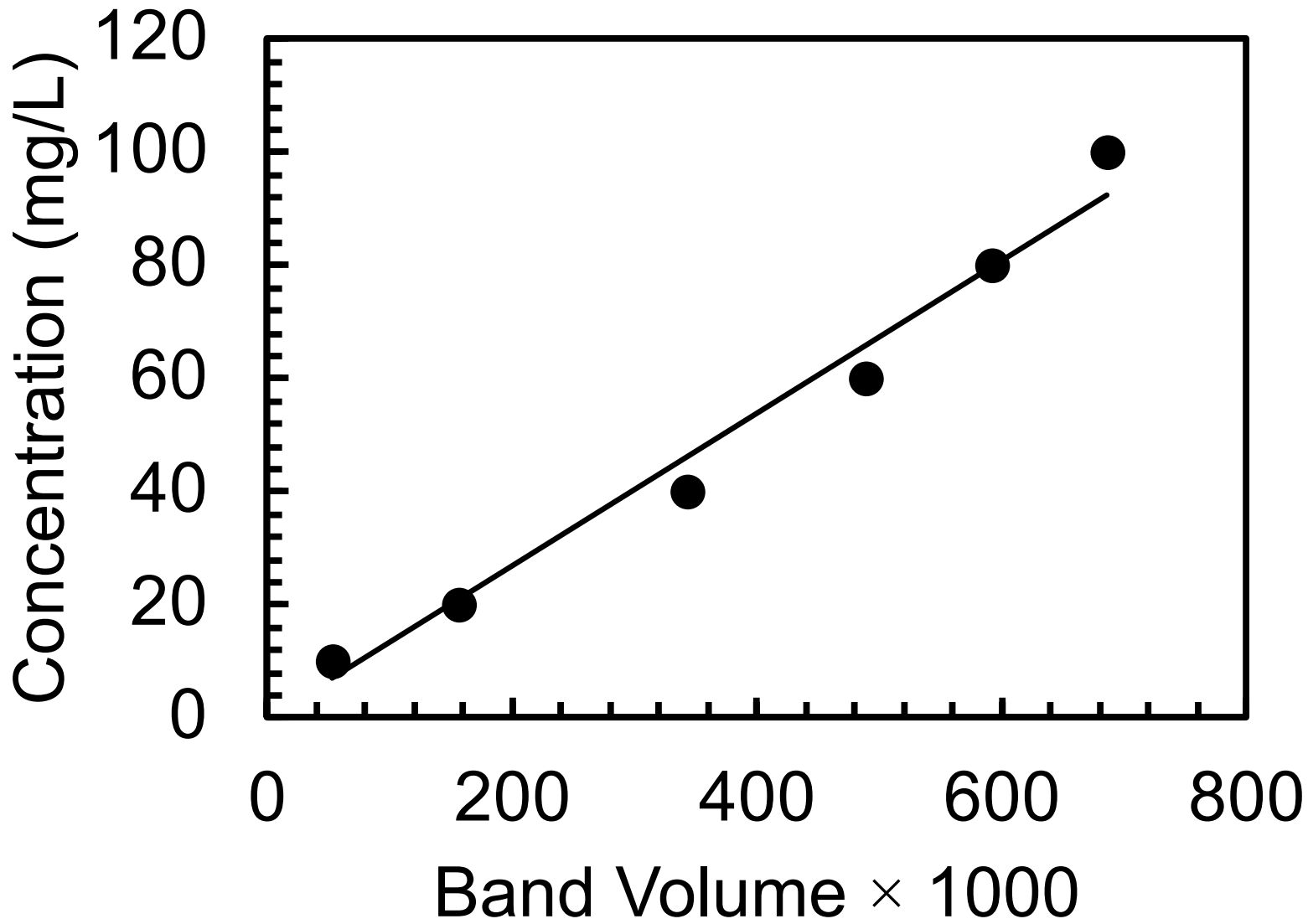

Figure S2. Calibration curve for nonheme enzyme concentration determination.

Table S3. Typical enzyme concentrations of EgtB$_{CHF}$ variants in cell-free lysate (CFL) determined using this method.

entry	EgtB$_{CHF}$ variant	Enzyme concentration (μM) in CFL
1	wt *Mth*EgtB	18.5
2	*Mth*EgtB Y377W W415R	13.9
3	*Mth*EgtB Y377W W415R R87D	21.4
4	*Mth*EgtB Y377W W415R R87D T141I	17.8
5	*Mth*EgtB Y377W W415R R87D T141I F83Y	22.3
6	*Mth*EgtB Y377W W415R R87D T141I F83Y R379E	20.3
7	*Mth*EgtB Y377W W415R R87D T141I F83Y R379E V84R	15.4
8	*Mth*EgtB Y377W W415R R87D T141I F83Y R379E V84R H417L (**EgtB$_{CHF1}$**)	10.7
9	*Mth*EgtB Y377W W415R R87D T141I F83Y R379E V84R H417L H138A (**EgtB$_{CHF1}$ H138A**)	15.6
10	*Mth*EgtB Y377W W415R R87D T141M	19.7
11	*Mth*EgtB Y377W W415R R87D T141M R379A	15.0
12	*Mth*EgtB Y377W W415R R87D T141M R379A A145K	21.2
13	*Mth*EgtB Y377W W415R R87D T141M R379A A145K Q422D (**EgtB$_{CHF2}$**)	21.6
14	*Mth*EgtB Y377W W415R R87D T141M R379A A145K Q422D H138A (**EgtB$_{CHF2}$ H138A**)	16.3

2. Determination of iron concentrations in purified nonheme Fe enzymes.

Iron in purified nonheme Fe enzyme was quantified using a modified ferrozine-based colorimetric method.[4] 500 µL purified nonheme Fe enzyme (100 µM) in KPi buffer (100 mM, pH = 7.4) was mixed with 250 µL of freshly prepared Reagent A (iron-releasing reagent; 1:1 mixture of 1.2 M HCl and 4.5% w/v $KMnO_4$) and incubated at 60 °C for 2 h in a sealed microcentrifuge tube to release enzyme bound iron. After cooling to room temperature, 50 µL of Reagent B (reducing/chelating reagent; 6.5 mM ferrozine, 13.1 mM neocuproine, 2.0 M ascorbic acid, and 5.0 M ammonium acetate in water) was added. The mixture was incubated at room temperature for 30 min to allow complete formation of the Fe(II)-ferrozine complex. Absorbance was measured at 562 nm using a UV–vis spectrophotometer. Iron concentrations were determined using a calibration curve developed using ferrous ethylenediammonium sulfate with 4 µg/mL, 2.4 µg/mL, 1.6 µg/mL, 0.8 µg/mL, 0.4 µg/mL, 0.2 µg/mL, and 0.1µg/mL.

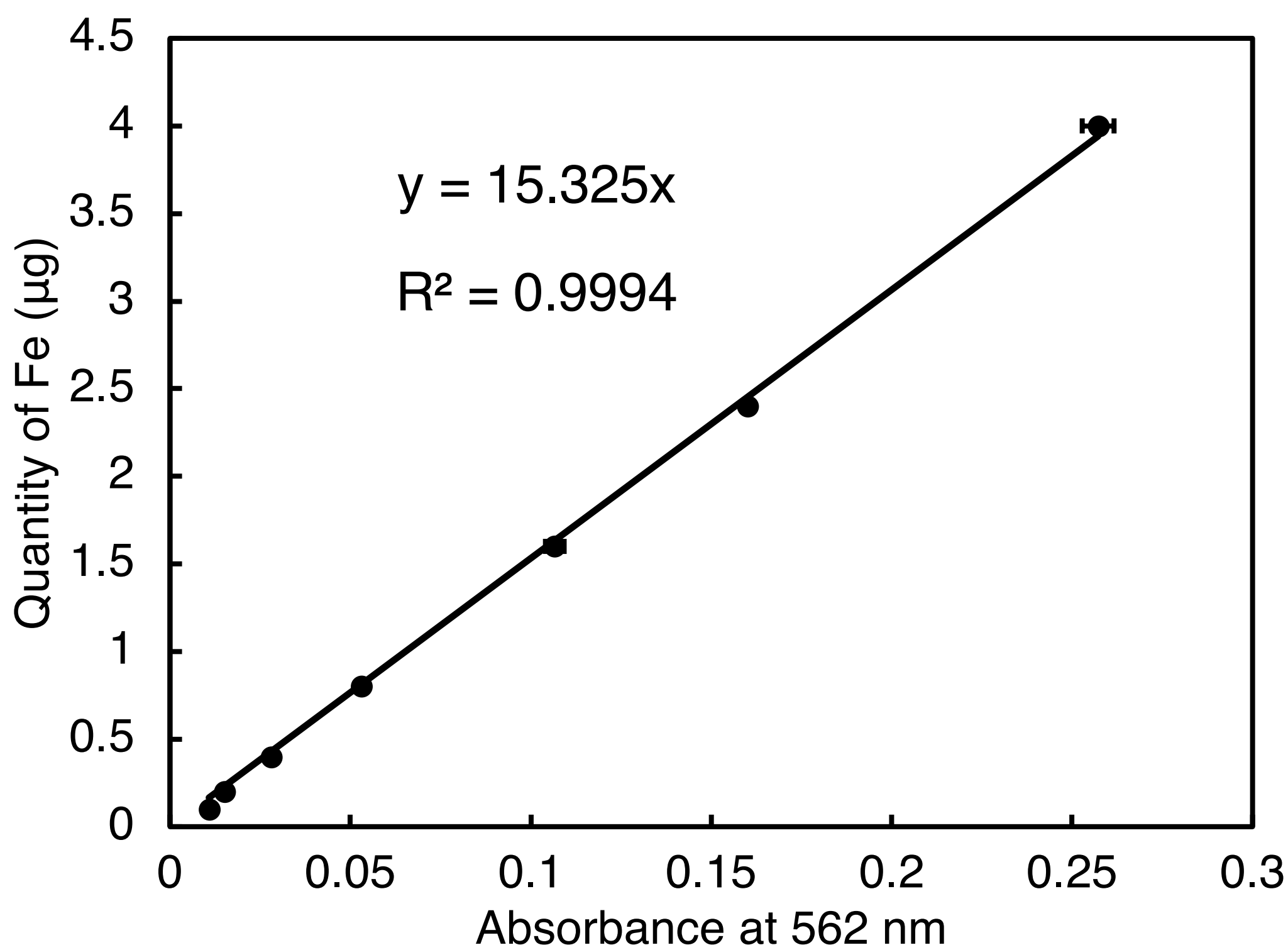

Figure S3. Ferrozine assay calibration curve for the determination of Fe concentration in purified nonheme Fe enzyme.

Table S4. Summary of Fe quantity in selected purified EgtB and ACCO variants determined by the ferrozine assay.

enzyme variant	absorbance at 562 nm[a]	Fe concentration (μg/mL)	Fe occupancy
$EgtB_{CHF1}$	0.153 ± 0.006	2.34 ± 0.09	42.5%
$EgtB_{CHF1}$ H51D	0.038 ± 0.001	0.58 ± 0.01	10.5%
$EgtB_{CHF1}$ H134E	0.019 ± 0.003	0.30 ± 0.05	5.4%
$EgtB_{CHF1}$ H138A	0.053 ± 0.002	0.81 ± 0.04	14.8%
$EgtB_{CHF1}$ H138D	0.097 ± 0.006	1.50 ± 0.09	27.2%
$EgtB_{CHF1}$ H138E	0.091 ± 0.005	1.41 ± 0.08	25.6%
$EgtB_{CHF2}$	0.180 ± 0.003	2.77 ± 0.05	50.4%
$EgtB_{CHF2}$ H138A	0.078 ± 0.007	1.20 ± 0.10	21.8%
$ACCO_{CHF}$	0.125 ± 0.004	1.92 ± 0.06	34.9%
$ACCO_{CHF}$ D179A	0.086 ± 0.004	1.32 ± 0.05	24.1%
$ACCO_{CHF}$ D179E	0.103 ± 0.007	1.58 ± 0.10	28.8%
$ACCO_{CHF}$ D179H	0.045 ± 0.005	0.69 ± 0.08	12.5%

[a]All the experiments were performed in technical triplicates, and the average absorbance values and standard deviations are reported.

Iron incorporation analysis revealed that primary sphere mutations altered Fe incorporation to varying degrees. Although excess Mohr's salt was supplemented during the biotransformations, the reduced iron occupancy observed for several mutants suggests that perturbation of the primary coordination sphere can compromise productive metal binding, thereby lowering the overall catalytic activity. Nevertheless, the changes in chemoselectivity and stereoselectivity observed for active mutants indicate that these mutations also modulate the intrinsic rebound selectivity, rather than merely affecting metal loading.

V. Mechanism studies.

1. Product analysis

Analysis of the reaction products obtained under the standard conditions revealed that no alkene side product, which would be expected from carbocation elimination pathways, were observed. In addition, no intramolecularly trapped lactam products arising from carbocation intermediates were detected. These observations argue against the involvement of a long-lived benzylic carbocation intermediate under the reaction conditions, consistent with previous experimental findings reported for Fe-catalyzed, fluoroamide-directed C–H fluorination reactions.[5]

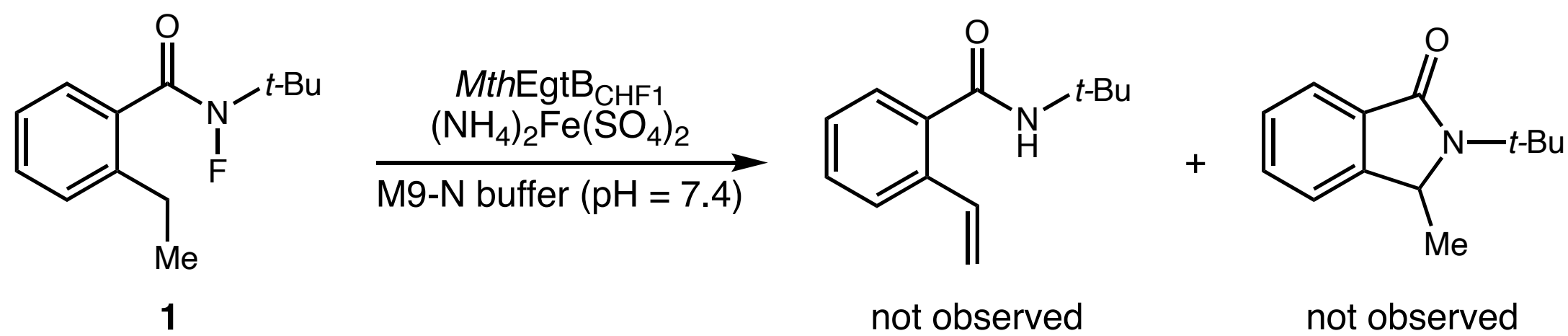

2. Radical clock experiments

Following the standard conditions, the cyclopropyl substrate **6** was subjected to reactions with cell-free lysate containing EgtB$_{CHF1}$ or EgtB$_{CHF2}$. GC-MS analysis revealed that neither the directed fluorinated product **S1** nor the ring-opened fluorinated product **S2** was detected. Instead, the ring-opened byproduct, (*E*)-2-(but-1-en-1-yl)-N-(tert-butyl)benzamide (**8**), was observed.

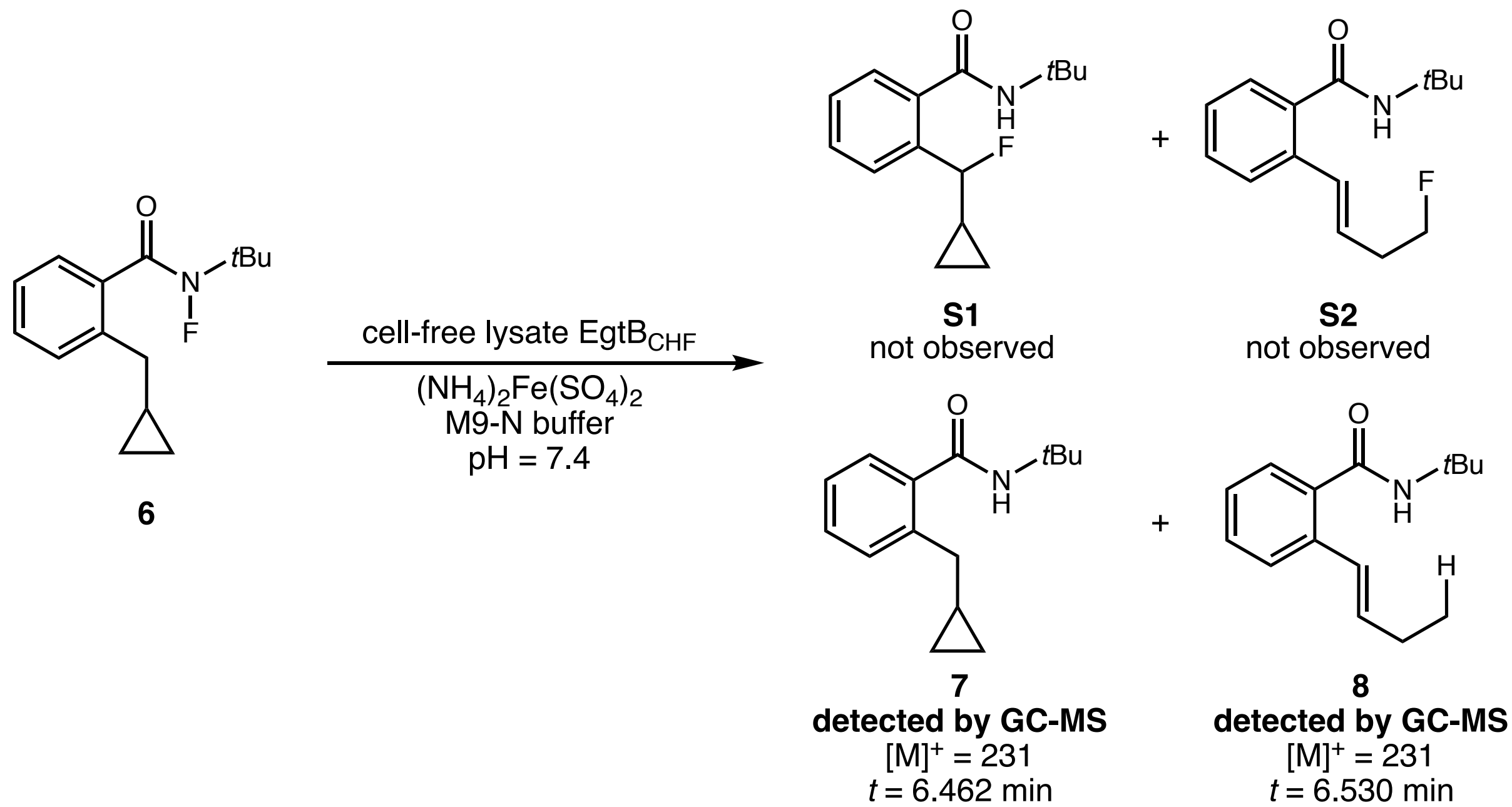

These results support the formation of intermediate **C** from intermediate **B** through a radical-mediated ring-opening process.

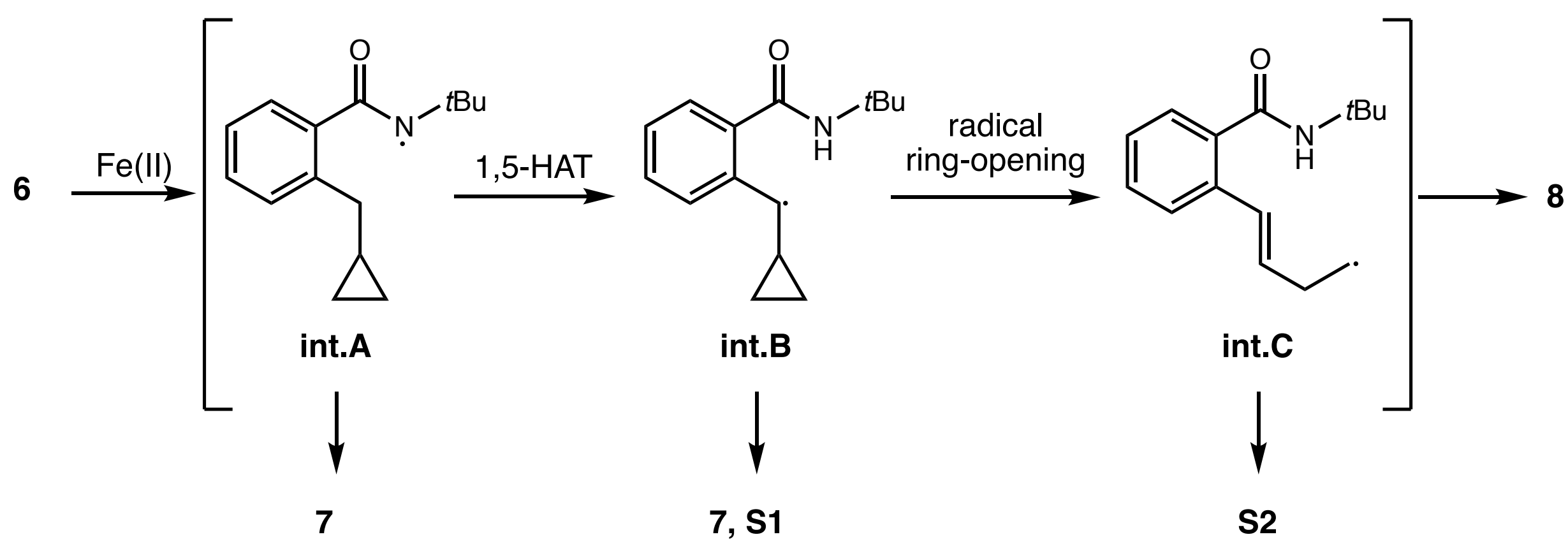

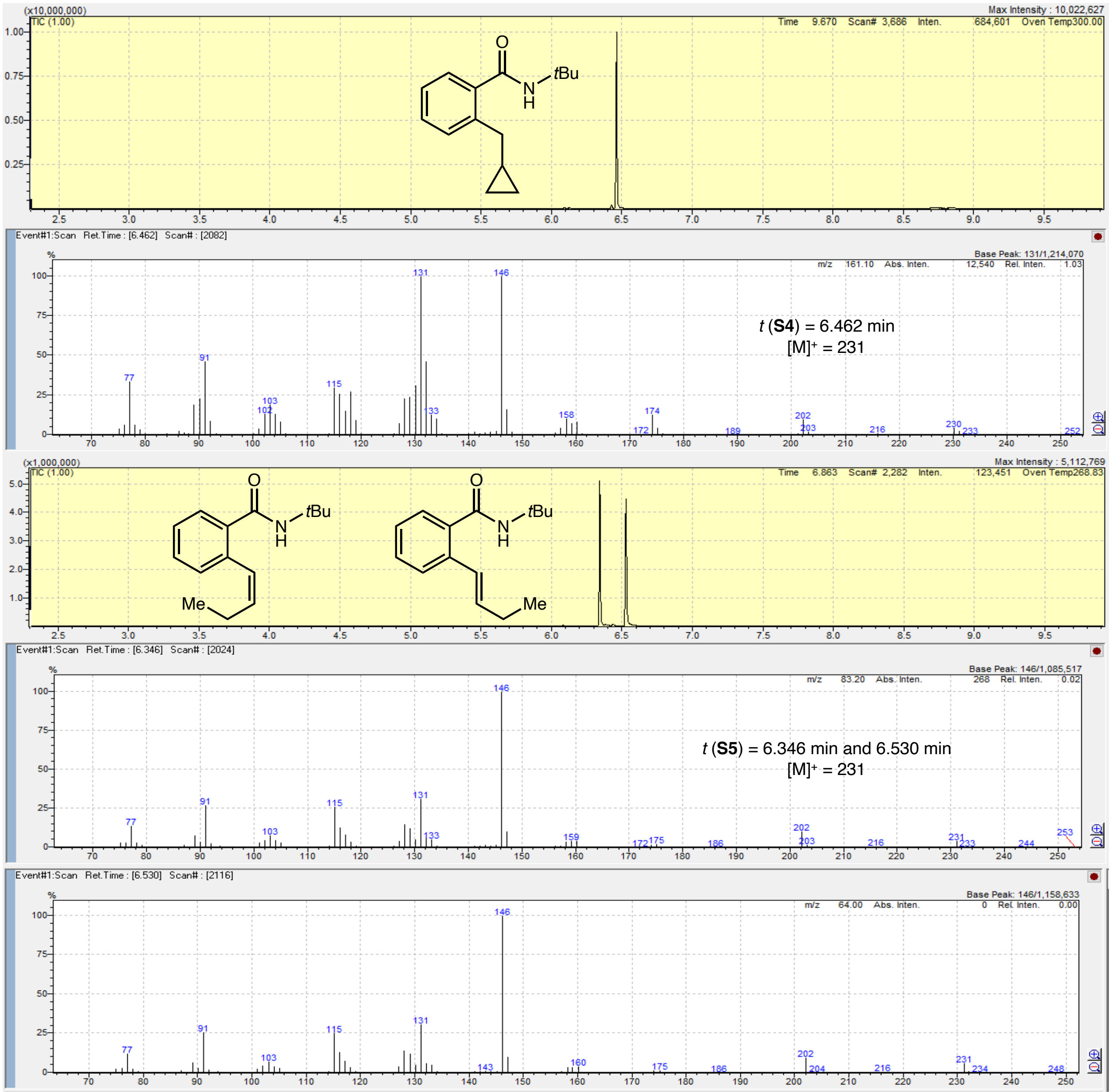

Figure S4. GC-MS analysis of authentic product **7** and (*Z*/*E*)-**8**.

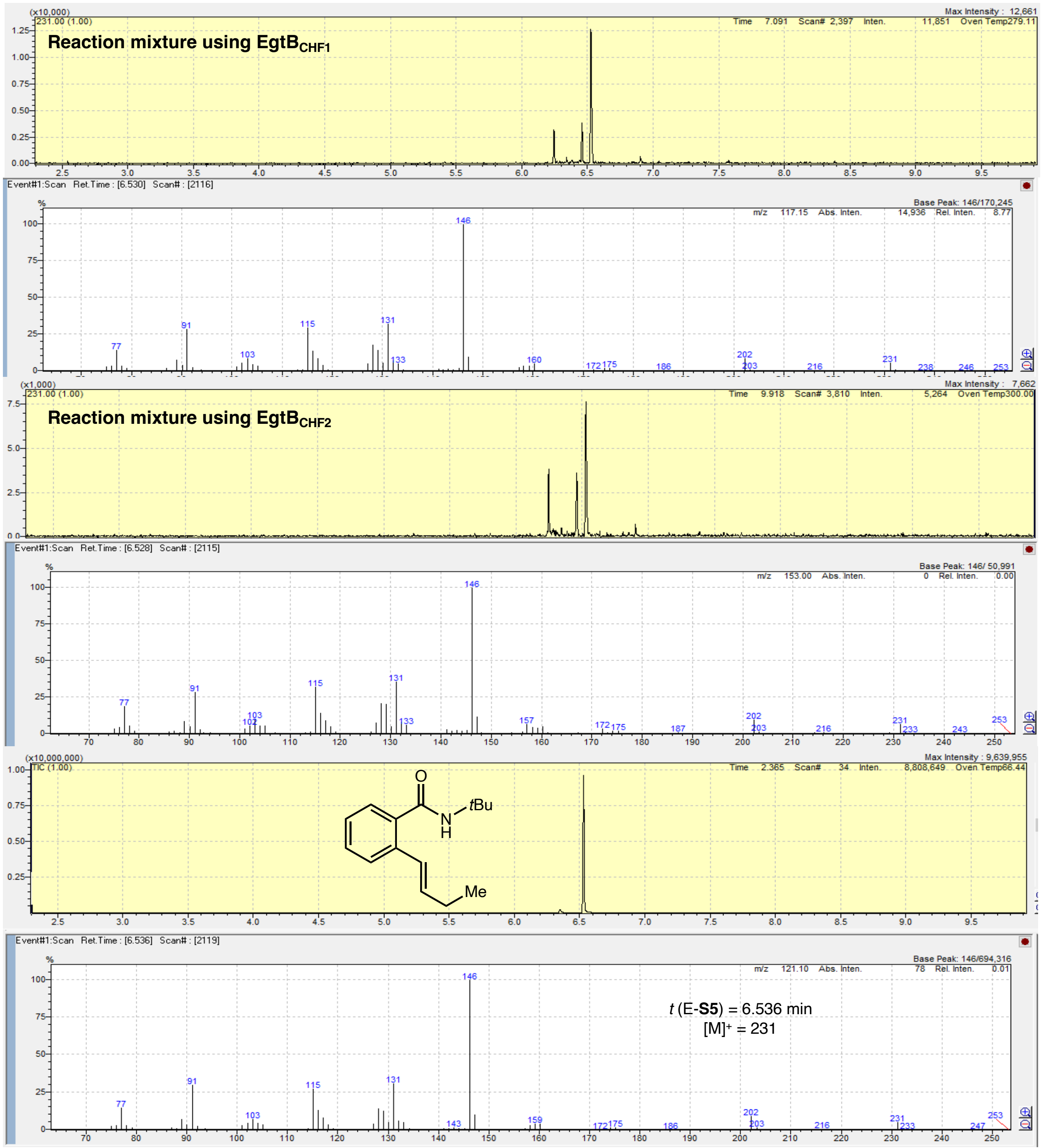

Figure S5. GC-MS analysis of reaction mixture.

3. TEMPO trapping experiments

Following the standard conditions, TEMPO trapping experiments was performed with $EgtB_{CHF1}$, $EgtB_{CHF2}$ and $ACCO_{CHF}$. Upon addition of 2.0 equiv of TEMPO to the reaction mixture, no desired fluorinated product was observed in any of the three enzymatic systems. Instead, the radical trapping adduct **9** was detected by LC-MS analysis. These results support the radical-mediated nature of this biocatalytic transformation.

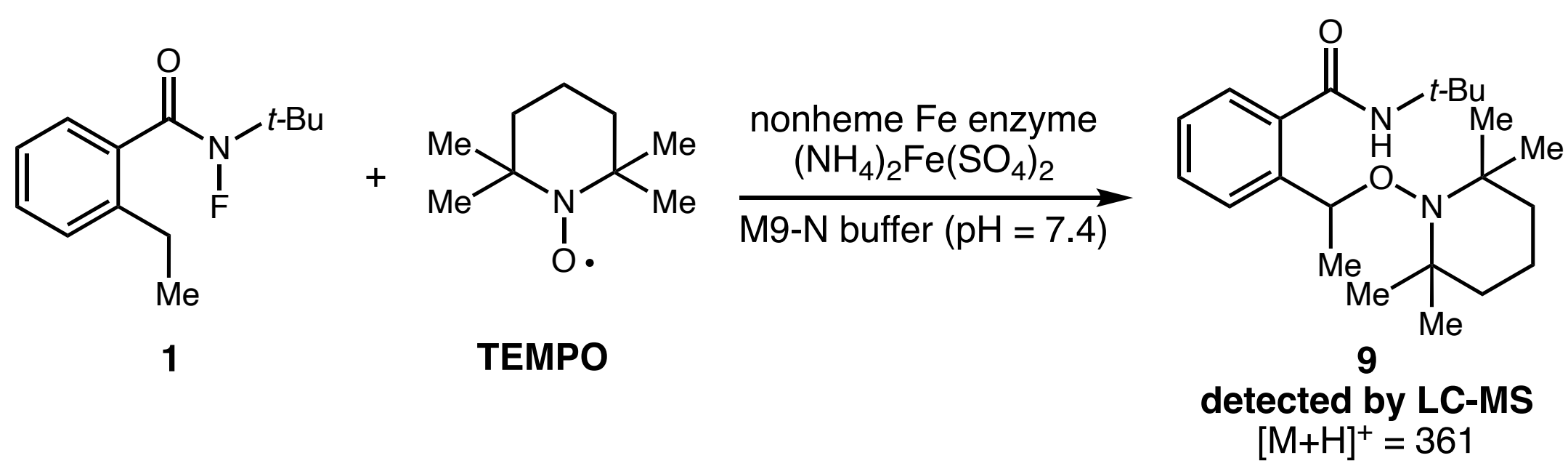

Together, the cyclopropyl radical clock and TEMPO trapping experiments provide strong evidence for the involvement of radical intermediates in this biocatalytic transformation.

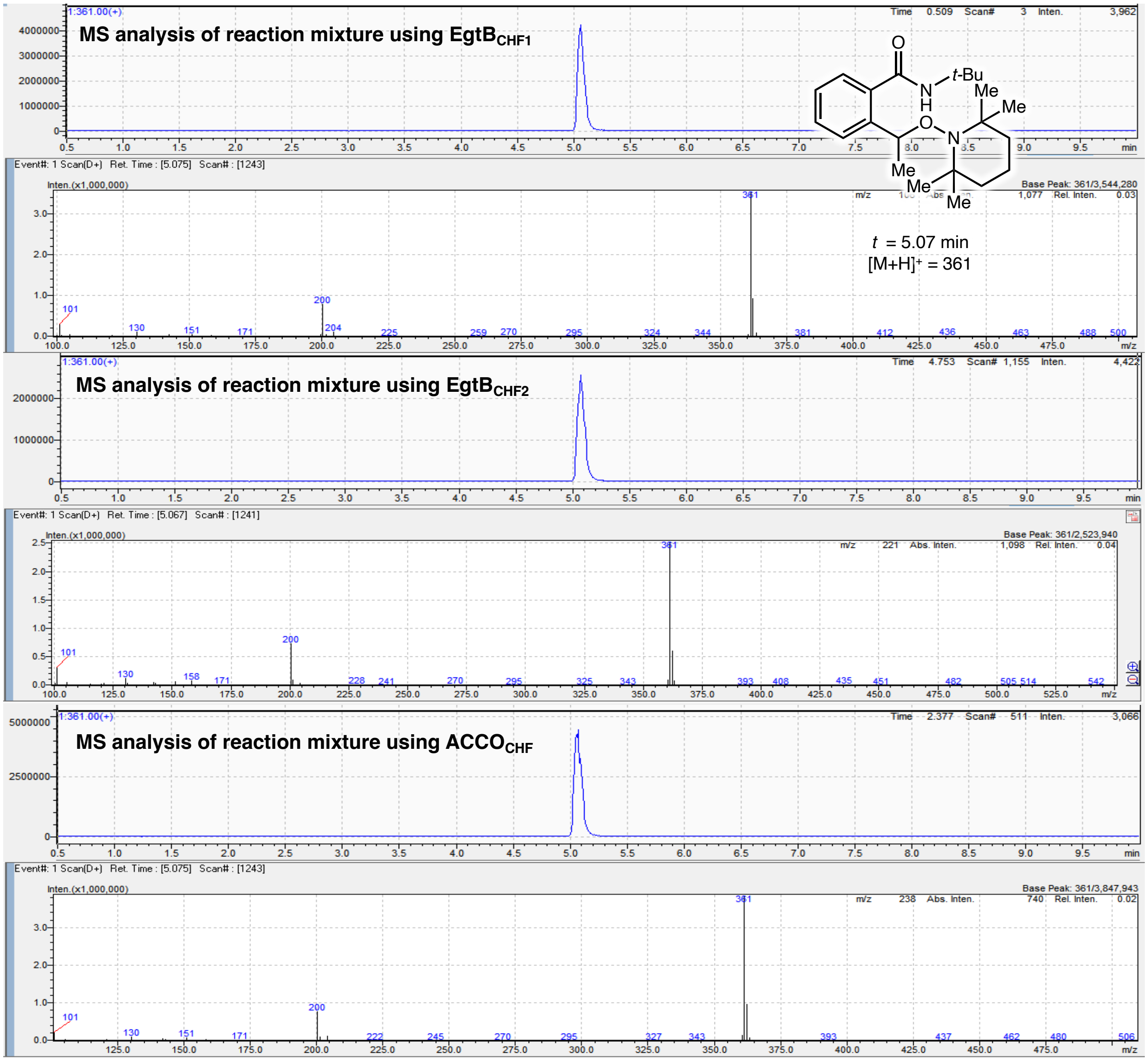

Figure S6. LC-MS analysis of reaction mixture.

4. ^{18}O-labeling experiments

^{18}O-labeling experiments was conducted using either $^{18}O_2$ or $H_2{}^{18}O$. When the reaction was performed under an atmosphere of $^{18}O_2$, no ^{18}O-incorporated hydroxylated product was detected, whereas clear ^{18}O incorporation was observed when the reaction was carried out in $H_2{}^{18}O$ containing buffer.

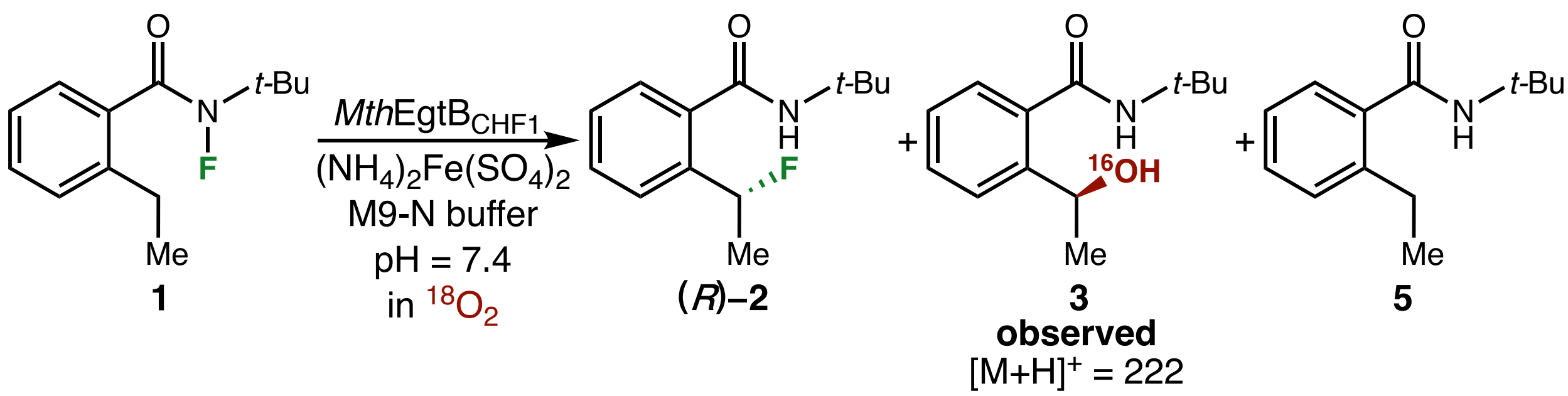

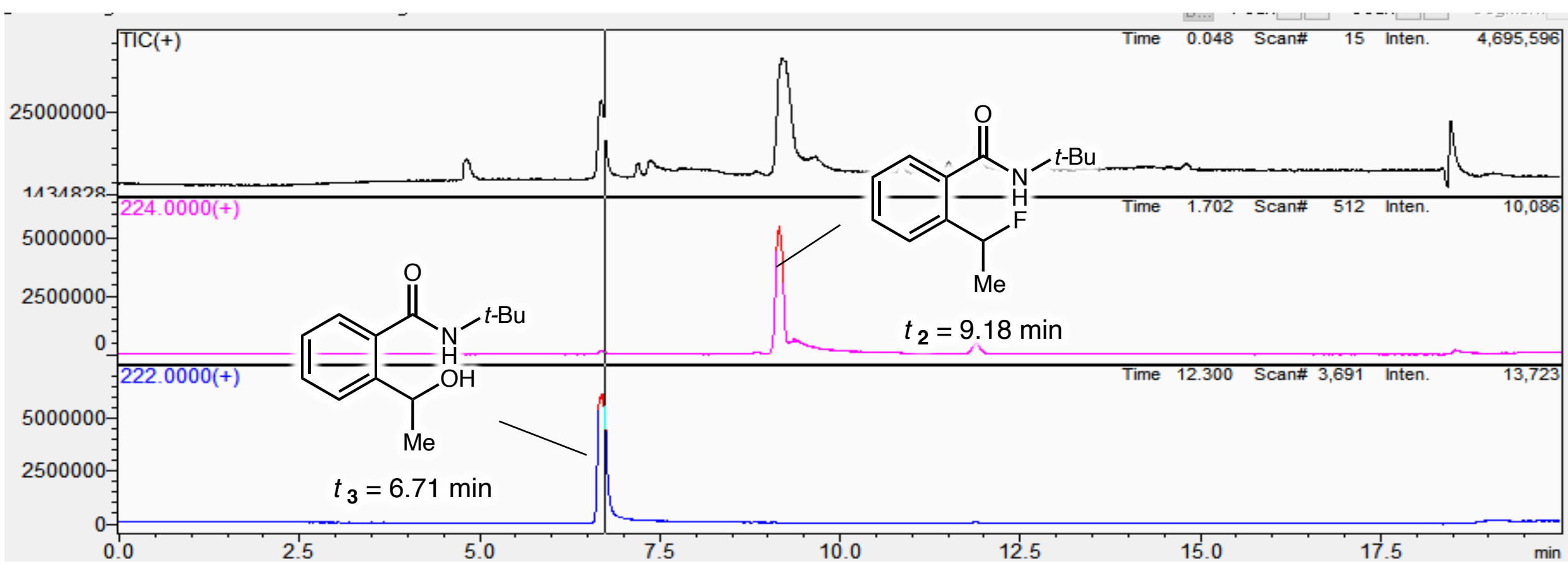

retention time (min)	extracted ion peak area ($\mathbf{3}_{m/z=222}$)	extracted ion peak area ($\mathbf{3}_{m/z=224}$)	area ratio ($\mathbf{3}_{m/z=222}$/$\mathbf{3}_{m/z=224}$)
6.71	$5.4*10^7$	$7.2*10^5$	99:1

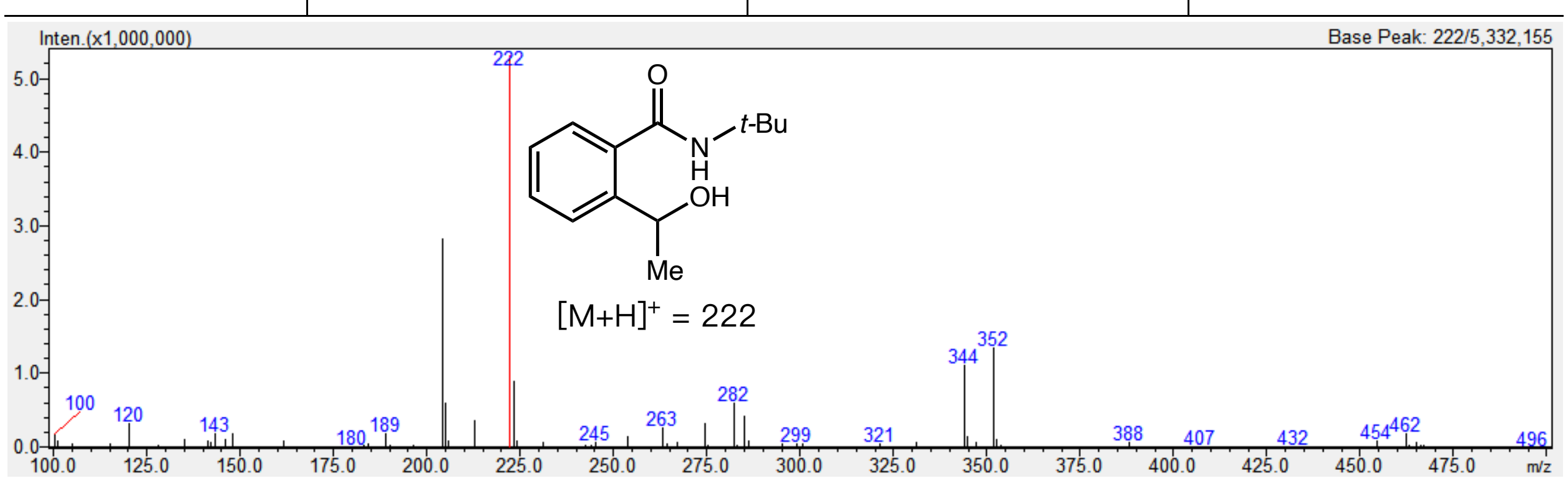

Figure S7. LC-MS analysis of reaction mixture.

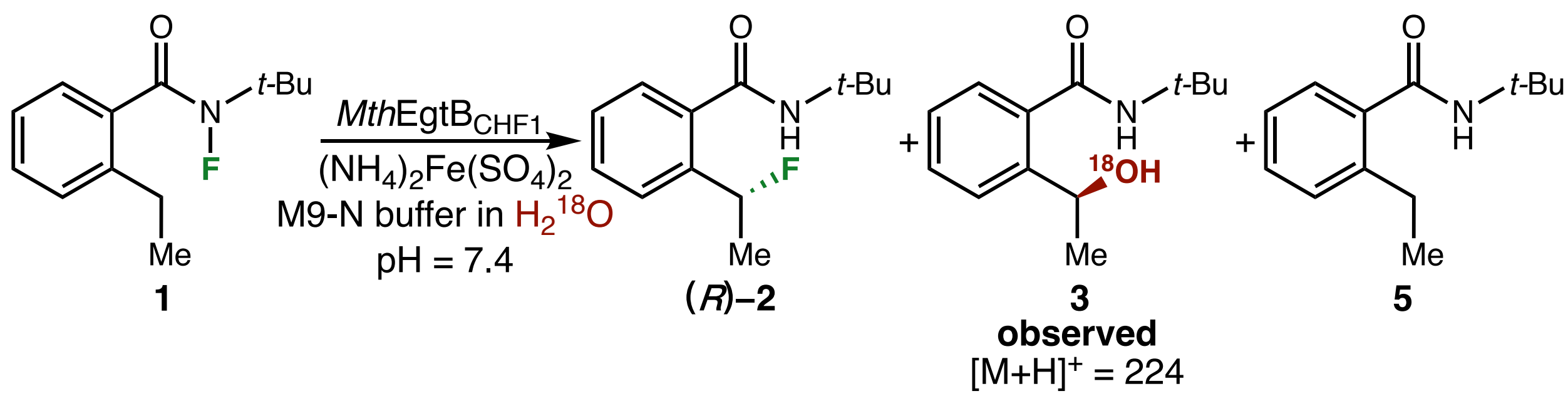

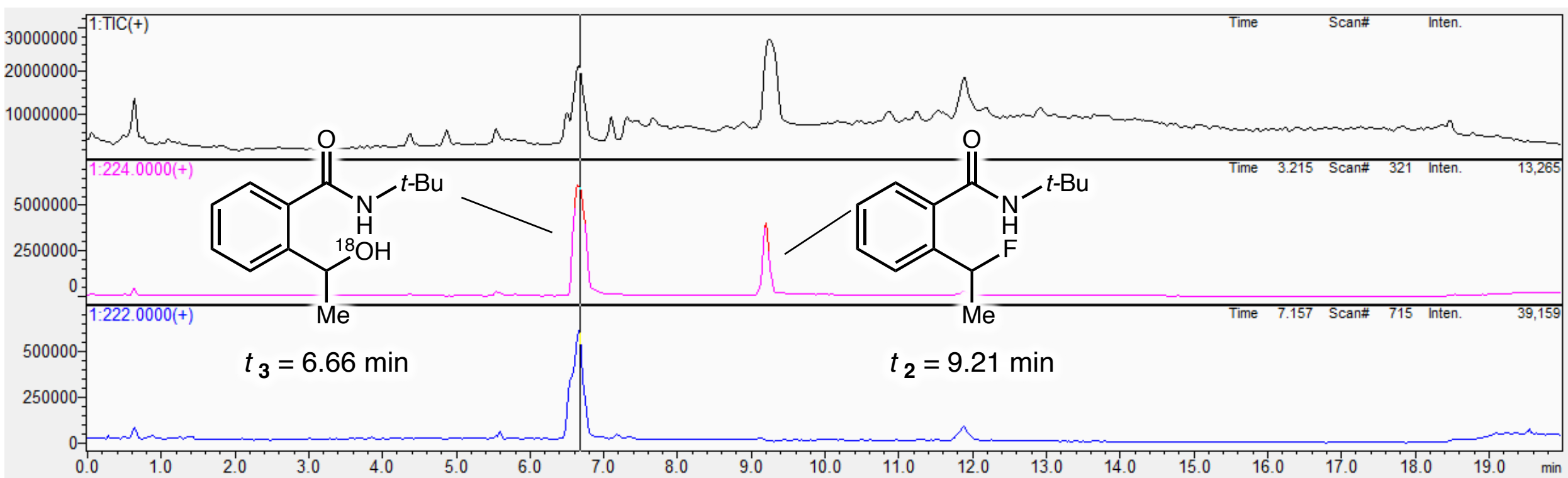

retention time (min)	extracted ion peak area ($\mathbf{3}_{m/z=222}$)	extracted ion peak area ($\mathbf{3}_{m/z=224}$)	area ratio ($\mathbf{3}_{m/z=224}/\mathbf{3}_{m/z=222}$)
6.66	6.4*10^6	6.8*10^7	91:9

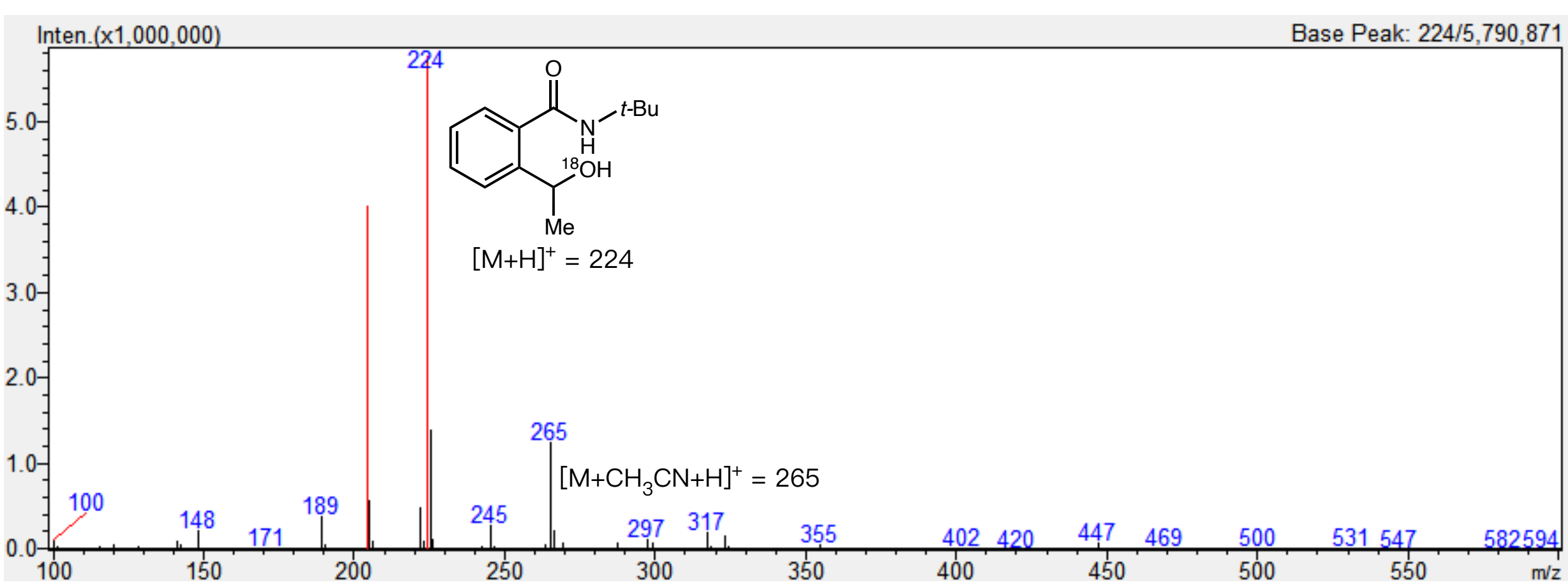

Figure S8. LC-MS analysis of reaction mixture.

VI. Primary coordination sphere effects on radical rebound activity and selectivity

To study the primary coordination sphere effect of the nonheme Fe enzyme on radical rebound activity and selectivity, we performed site-directed mutagenesis on evolved *Mth*EgtB to mutate the Fe-binding histidine (H) residues to aspartate (D), glutamate (E), and alanine (A) in the Fe center. A similar mutagenesis study was also carried out with our previously evolved C-H fluorinase $ACCO_{CHF}$ to mutate the Fe-binding aspartate (D) to E, H and A. A detailed discussion is provided in the main text.

Table S5. Primary coordination sphere effects on C–H fluorination with $EgtB_{CHF1}$

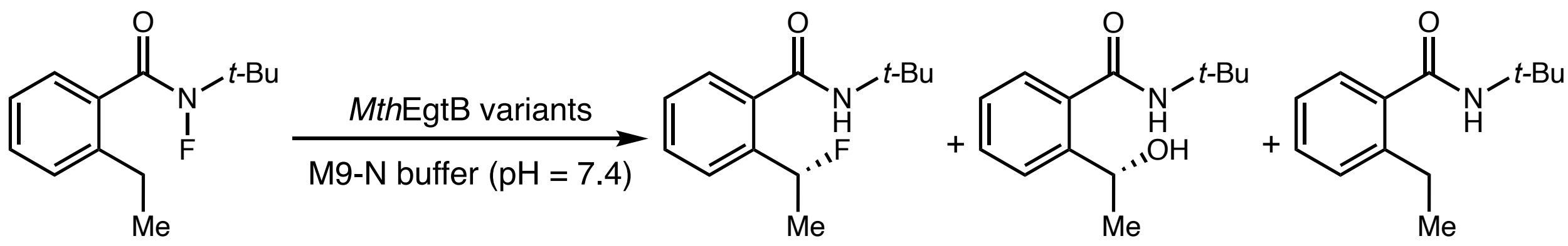

entry	variants	2		3		5
		yield (%)	e.r. ((*R*)-**2** : (*S*)-**2**)	yield (%)	e.r. ((*R*)-**3** : (*S*)-**3**)	yield (%)
1	$EgtB_{CHF1}$	30.7 ± 0.7	60:40	32 ± 1	23:77	14.7 ± 0.3
2	$EgtB_{CHF1}$ H51D	0	-	0	-	5
3	$EgtB_{CHF1}$ H51E	0	-	0	-	5
4	$EgtB_{CHF1}$ H51A	0	-	0	-	4
5	$EgtB_{CHF1}$ H134D	0	-	0	-	4
6	$EgtB_{CHF1}$ H134E	0	-	0	-	4
7	$EgtB_{CHF1}$ H134A	0	-	0	-	5
8	$EgtB_{CHF1}$ H138D	5.0 ± 0.1	36:64	0	-	6.3 ± 0.2
9	$EgtB_{CHF1}$ H138E	6.6 ± 0.1	35:65	0	-	4.4 ± 0.1

10	EgtB$_{CHF1}$ H138A	7.3 ± 0.4	59:41	13.5 ± 0.1	24:76	7.0 ± 0.2

Reaction conditions: 7.5 mM **1**, 0.75 mM $(NH_4)_2Fe(SO_4)_2$, 7.5 mM sodium ascorbate, 500 μL cell-free lysate of EgtB variant, M9-N buffer (pH = 7.4). **2**: fluorination product, **3**: hydroxylation product, **5**: reduction product.

Table S6. Primary coordination sphere effects on C–H fluorination with EgtB$_{CHF2}$

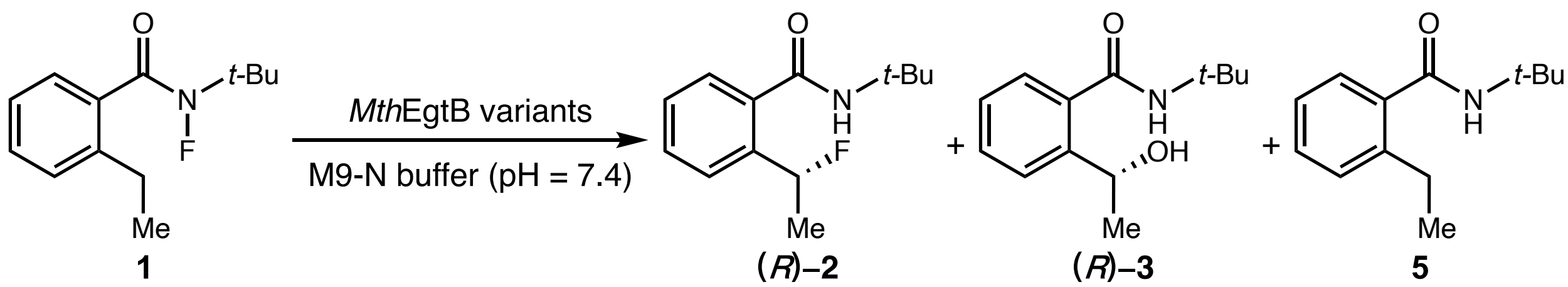

entry	variants	**2**		**3**		**5**
		yield (%)	e.r. ((*R*)-**2** : (*S*)-**2**)	yield (%)	e.r. ((*R*)-**3** : (*S*)-**3**)	yield (%)
1	EgtB$_{CHF2}$	35.5 ± 0.1	31:69	17.3 ± 0.5	34:66	16.9 ± 0.6
2	EgtB$_{CHF2}$ H51D	0	-	0	-	5
3	EgtB$_{CHF2}$ H51E	0	-	0	-	5
4	EgtB$_{CHF2}$ H51A	0	-	0	-	5
5	EgtB$_{CHF2}$ H134D	0	-	0	-	5
6	EgtB$_{CHF2}$ H134E	0	-	0	-	5
7	EgtB$_{CHF2}$ H134A	0	-	0	-	5
8	EgtB$_{CHF2}$ H138D	0	-	0	-	2.6 ± 0.2
9	EgtB$_{CHF2}$ H138E	1.3 ± 0.1	35:65	0	-	4.4 ± 0.1
10	EgtB$_{CHF2}$ H138A	0	-	0	-	2.1 ± 0.2

Reaction conditions: 7.5 mM **1**, 0.75 mM $(NH_4)_2Fe(SO_4)_2$, 7.5 mM sodium ascorbate, 500 µL cell-free lysate of EgtB variant, M9-N buffer (pH = 7.4). **2**: fluorination product, **3**: hydroxylation product, **5**: reduction product.

Table S7. Primary coordination sphere effects on C–H fluorination with $ACCO_{CHF}$ under whole-cell reaction conditions

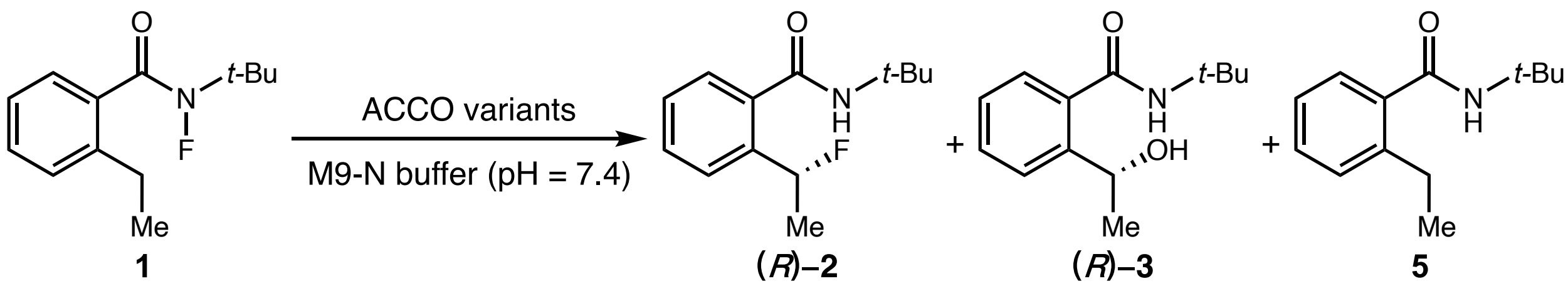

entry	variants	2		3		5
		yield (%)	e.r. ((*R*)-**2** : (*S*)-**2**)	yield (%)	e.r. ((*R*)-**3** : (*S*)-**3**)	yield (%)
1	$ACCO_{CHF}$	89 ± 3	5:95	0	-	1.5 ± 0.3
2	$ACCO_{CHF}$ D179E	0	-	0	-	9.7 ± 0.2
3	$ACCO_{CHF}$ D179H	0	-	0	-	2.6 ± 0.2
4	$ACCO_{CHF}$ D179A	0	-	0	-	4.9 ± 0.4

Reaction conditions: 7.5 mM **1**, 0.75 mM $(NH_4)_2Fe(SO_4)_2$, 500 µL whole-cell suspension of ACCO variant, M9-N buffer (pH = 7.4). **2**: fluorination product, **3**: hydroxylation product, **5**: reduction product.

Table S8. Primary coordination sphere effects on C–H fluorination with $ACCO_{CHF}$ under cell-free lysate reaction conditions

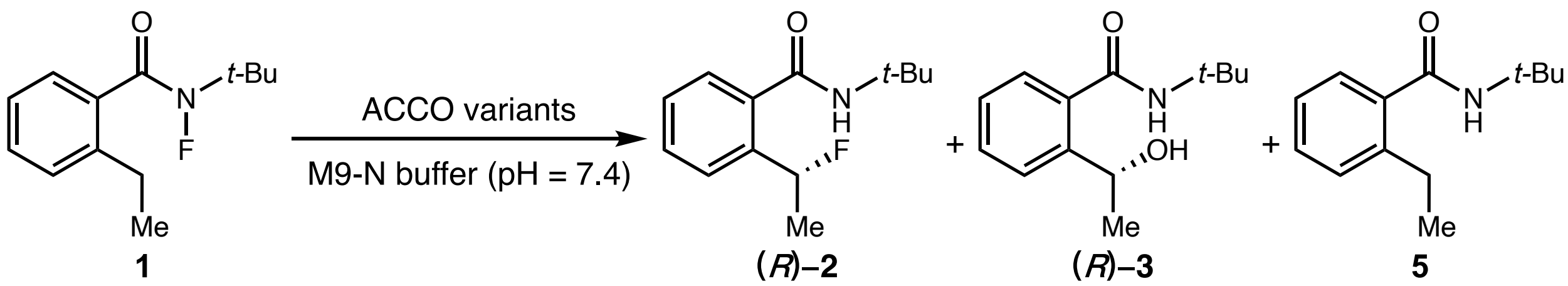

entry	variants	**2**		**3**		**5**
		yield (%)	e.r. ((*R*)-**2** : (*S*)-**2**)	yield (%)	e.r. ((*R*)-**3** : (*S*)-**3**)	yield (%)
1	$ACCO_{CHF}$	43 ± 2	11:89	0	-	3.0 ± 0.3
2	$ACCO_{CHF}$ D179E	0	-	0	-	2.7 ± 0.2
3	$ACCO_{CHF}$ D179H	0	-	0	-	1.2 ± 0.1
4	$ACCO_{CHF}$ D179A	0	-	0	-	2.9 ± 0.3

Reaction conditions: 7.5 mM **1**, 0.75 mM $(NH_4)_2Fe(SO_4)_2$, 7.5 mM sodium ascorbate, 500 µL cell-free lysate of ACCO variant, M9-N buffer (pH = 7.4). **2**: fluorination product, **3**: hydroxylation product, **5**: reduction product.

Primary coordination sphere effects on C–H azidation

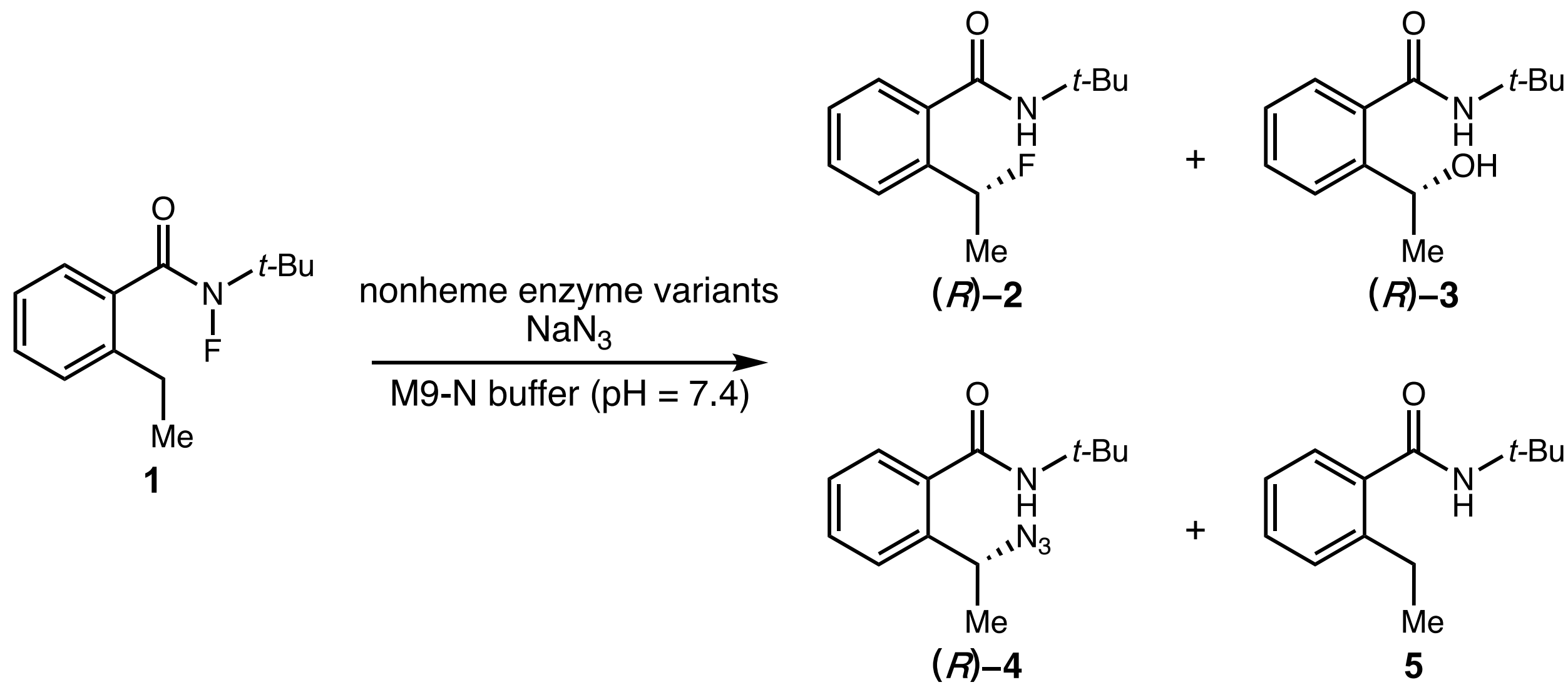

Table S9. Primary coordination sphere effects on C–H azidation with $EgtB_{CHF1}$

entry	variants	**2**		**3**		**4**		**5**
		yield (%)	e.r. ((*R*)-**2** : (*S*)-**2**)	yield (%)	e.r. ((*R*)-**3** : (*S*)-**3**)	yield (%)	e.r. ((*R*)-**4** : (*S*)-**4**)	yield (%)
1	$EgtB_{CHF1}$	15 ± 2	60:40	23 ± 1	23:77	50 ± 2	44:56	15.4 ± 0.9
2	$EgtB_{CHF1}$ H51D	0	-	0	-	0	-	5
3	$EgtB_{CHF1}$ H51E	0	-	0	-	0	-	5
4	$EgtB_{CHF1}$ H51A	0	-	0	-	0	-	5
5	$EgtB_{CHF1}$ H134D	0	-	0	-	0	-	5
6	$EgtB_{CHF1}$ H134E	0	-	0	-	0	-	5
7	$EgtB_{CHF1}$ H134A	0	-	0	-	0	-	5
8	$EgtB_{CHF1}$ H138D	2.3 ± 0.2	35:65	0	-	0	-	3.3 ± 0.1
9	$EgtB_{CHF1}$ H138E	6.7 ± 0.2	35:65	0	-	<1	-	5.1 ± 0.2
10	$EgtB_{CHF1}$ H138A	7.5 ± 0.3	50:50	9.0 ± 0.9	24:76	31.2 ± 0.8	45:55	9.1 ± 0.3

Reaction conditions: 7.5 mM **1**, 120 mM NaN_3, 0.75 mM $(NH_4)_2Fe(SO_4)_2$, 7.5 mM sodium ascorbate, 500 µL cell-free lysate of EgtB variant, M9-N buffer (pH = 7.4). **2**: fluorination product, **3**: hydroxylation product, **4**: azidation product, **5**: reduction product.

Table S10. Primary coordination sphere effects on C–H azidation with $EgtB_{CHF2}$

entry	variants	**2**		**3**		**4**		**5**
		yield (%)	e.r. ((*R*)-**2** : (*S*)-**2**)	yield (%)	e.r. ((*R*)-**3** : (*S*)-**3**)	yield (%)	e.r. ((*R*)-**4** : (*S*)-**4**)	yield (%)
1	$EgtB_{CHF2}$	14.9 ± 0.5	31:69	9.7 ± 0.4	33:67	40 ± 2	39:61	12.1 ± 0.5
2	$EgtB_{CHF2}$ H51D	0	-	0	-	<1	-	5
3	$EgtB_{CHF2}$ H51E	0	-	0	-	<1	-	5
4	$EgtB_{CHF2}$ H51A	0	-	0	-	<1	-	5
5	$EgtB_{CHF2}$ H134D	0	-	0	-	<1	-	5
6	$EgtB_{CHF2}$ H134E	0	-	0	-	<1	-	5
7	$EgtB_{CHF2}$ H134A	0	-	0	-	<1	-	5
8	$EgtB_{CHF2}$ H138D	0	-	0	-	<1	-	3
9	$EgtB_{CHF2}$ H138E	1.4 ± 0.1	-	0	-	1.0 ± 0.1	-	5.3 ± 0.2
10	$EgtB_{CHF2}$ H138A	1.0 ± 0.1	-	0	-	42 ± 2	27:73	7.6 ± 0.1

Reaction conditions: 7.5 mM **1**, 120 mM NaN_3, 0.75 mM $(NH_4)_2Fe(SO_4)_2$, 7.5 mM sodium ascorbate, 500 µL cell-free lysate of EgtB variant, M9-N buffer (pH = 7.4). **2**: fluorination product, **3**: hydroxylation product, **4**: azidation product, **5**: reduction product.

Table S11. Primary coordination sphere effects on C–H azidation with $ACCO_{CHF}$ under whole-cell reaction conditions

entry	variants	**2**		**3**		**4**		**5**
		yield (%)	e.r. ((*R*)-**2** : (*S*)-**2**)	yield (%)	e.r. ((*R*)-**3** : (*S*)-**3**)	yield (%)	e.r. ((*R*)-**4** : (*S*)-**4**)	yield (%)
1	$ACCO_{CHF}$	77 ± 3	6:94	0	-	12 ± 1	49:51	5 ± 1
2	$ACCO_{CHF}$ D179E	0	-	0	-	14 ± 2	45:55	9.7 ± 0.2
3	$ACCO_{CHF}$ D179H	0	-	0	-	0	-	2.6 ± 0.2
4	$ACCO_{CHF}$ D179A	0	-	0	-	10 ± 1	42:58	4.9 ± 0.4

Reaction conditions: 7.5 mM **1**, 120 mM NaN_3, 0.75 mM $(NH_4)_2Fe(SO_4)_2$, 500 µL whole-cell suspension of ACCO variant, M9-N buffer (pH = 7.4). **2**: fluorination product, **3**: hydroxylation product, **4**: azidation product, **5**: reduction product.

Table S12. Primary coordination sphere effects on C–H azidation with $ACCO_{CHF}$ under cell-free lysate reaction conditions

entry	variants	**2**		**3**		**4**		**5**
		yield (%)	e.r. ((*R*)-**2** : (*S*)-**2**)	yield (%)	e.r. ((*R*)-**3** : (*S*)-**3**)	yield (%)	e.r. ((*R*)-**4** : (*S*)-**4**)	yield (%)
1	$ACCO_{CHF}$	51 ± 2	18:82	0	-	22 ± 1	42:58	8 ± 1
2	$ACCO_{CHF}$ D179E	0	-	0	-	35 ± 2	45:55	15 ± 1
3	$ACCO_{CHF}$ D179H	0	-	0	-	0	-	2.1 ± 0.2
4	$ACCO_{CHF}$ D179A	0	-	0	-	35 ± 2	43:57	15 ± 1

Reaction conditions: 7.5 mM **1**, 120 mM NaN_3, 0.75 mM $(NH_4)_2Fe(SO_4)_2$, 7.5 mM sodium ascorbate, 500 µL cell-free lysate of ACCO variant, M9-N buffer (pH = 7.4). **2**: fluorination product, **3**: hydroxylation product, **4**: azidation product, **5**: reduction product.

VII. pH effects on radical rebound activity and selectivity

To further investigate the effect of pH on hydroxyl rebound activity and selectivity in nonheme Fe enzymes, we performed fluorination and azidation reactions using the evolved variants *Mth*EgtB$_{CHF1}$, *Mth*EgtB$_{CHF2}$ and ACCO$_{CHF}$ over a range of buffer pH values (6.5–9.0). The results revealed that higher pH conditions generally promoted both the activity and selectivity of hydroxylation. In particular, *Mth*EgtB$_{CHF1}$ exhibited the highest hydroxylation performance at pH 9.0, affording the hydroxylation product in 56% yield with a hydroxylation-to-fluorination ratio of 2:1.

Table S13. pH effects on C–H hydroxylation with EgtB$_{CHF1}$

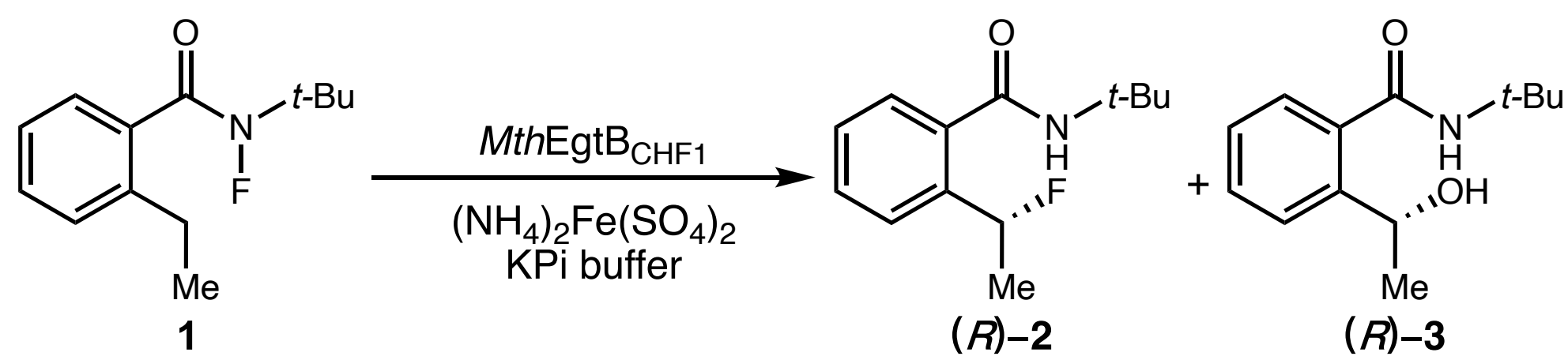

entry	buffer pH	2		3		5
		yield (%)	e.r. ((*R*)-**2** : (*S*)-**2**)	yield (%)	e.r. ((*R*)-**3** : (*S*)-**3**)	yield (%)
1	6.5	31 ± 2	58:42	36 ± 2	25:75	13 ± 1
2	7.0	31 ± 1	59:41	44 ± 1	24:76	14 ± 1
3	7.5	29 ± 1	60:40	48 ± 1	23:77	13 ± 1
4	8.0	26 ± 1	62:38	49 ± 1	20:80	13 ± 1
5	8.5	26 ± 1	62:38	49 ± 1	20:80	14 ± 1
6	9.0	28 ± 1	62:38	54 ± 1	20:80	15 ± 1

Reaction conditions: 7.5 mM **1**, 0.75 mM $(NH_4)_2Fe(SO_4)_2$, 7.5 mM sodium ascorbate, 500 μL cell-free lysate of EgtB$_{CHF1}$ variant, 100 mM KPi buffer. **2**: fluorination product, **3**: hydroxylation product, **5**: reduction product.

Table S14. pH effects on C–H hydroxylation with $EgtB_{CHF2}$

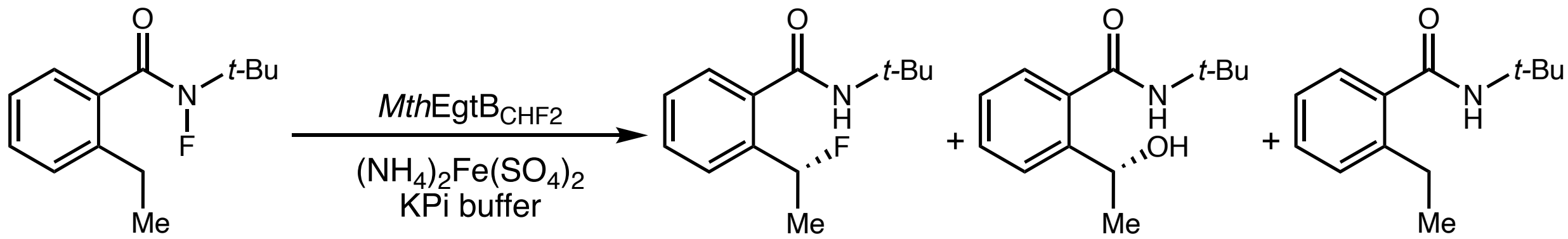

entry	buffer pH	2		3		5
		yield (%)	e.r. ((*R*)-**2** : (*S*)-**2**)	yield (%)	e.r. ((*R*)-**3** : (*S*)-**3**)	yield (%)
1	6.5	36 ± 2	30:70	17 ± 1	32:68	15 ± 1
2	7.0	32 ± 1	32:68	17 ± 1	30:70	14 ± 1
3	7.5	40 ± 1	31:69	18 ± 1	34:66	17 ± 1
4	8.0	38 ± 4	31:69	22 ± 1	29:71	17 ± 2
5	8.5	46 ± 2	31:69	25 ± 2	32:68	21 ± 1
6	9.0	43 ± 1	31:69	28 ± 2	31:69	21 ± 1

Reaction conditions: 7.5 mM **1**, 0.75 mM $(NH_4)_2Fe(SO_4)_2$, 7.5 mM sodium ascorbate, 500 µL cell-free lysate of $EgtB_{CHF2}$ variant, 100 mM KPi buffer. **2**: fluorination product, **3**: hydroxylation product, **5**: reduction product.

Table S15. pH effects on C–H hydroxylation with $ACCO_{CHF}$

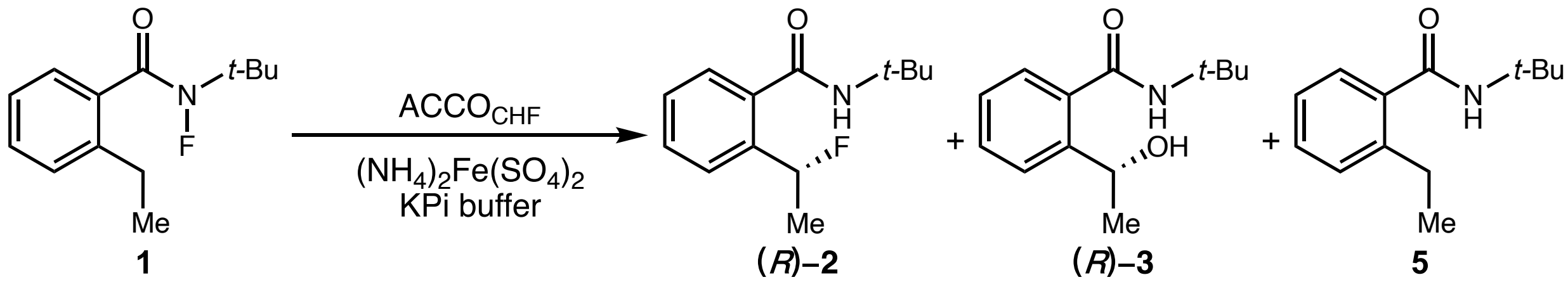

entry	buffer pH	**2**		**3**		**5**
		yield (%)	e.r. ((*R*)-**2** : (*S*)-**2**)	yield (%)	e.r. ((*R*)-**3** : (*S*)-**3**)	yield (%)
1	6.5	73 ± 3	6:94	0	-	3.9 ± 0.6
2	7.0	71 ± 1	6:94	0	-	4.1 ± 0.2
3	7.5	83 ± 5	5:95	0	-	5.1 ± 0.6
4	8.0	77 ± 4	5:95	0	-	5.7 ± 0.3
5	8.5	79 ± 4	6:94	0	-	6.8 ± 0.5
6	9.0	80 ± 6	6:94	0	-	8.1 ± 0.6

Reaction conditions: 7.5 mM **1**, 0.75 mM $(NH_4)_2Fe(SO_4)_2$, 500 µL whole-cell suspension of $ACCO_{CHF}$ variant, 100 mM KPi buffer. **2**: fluorination product, **3**: hydroxylation product, **5**: reduction product.

pH effects on C–H azidation

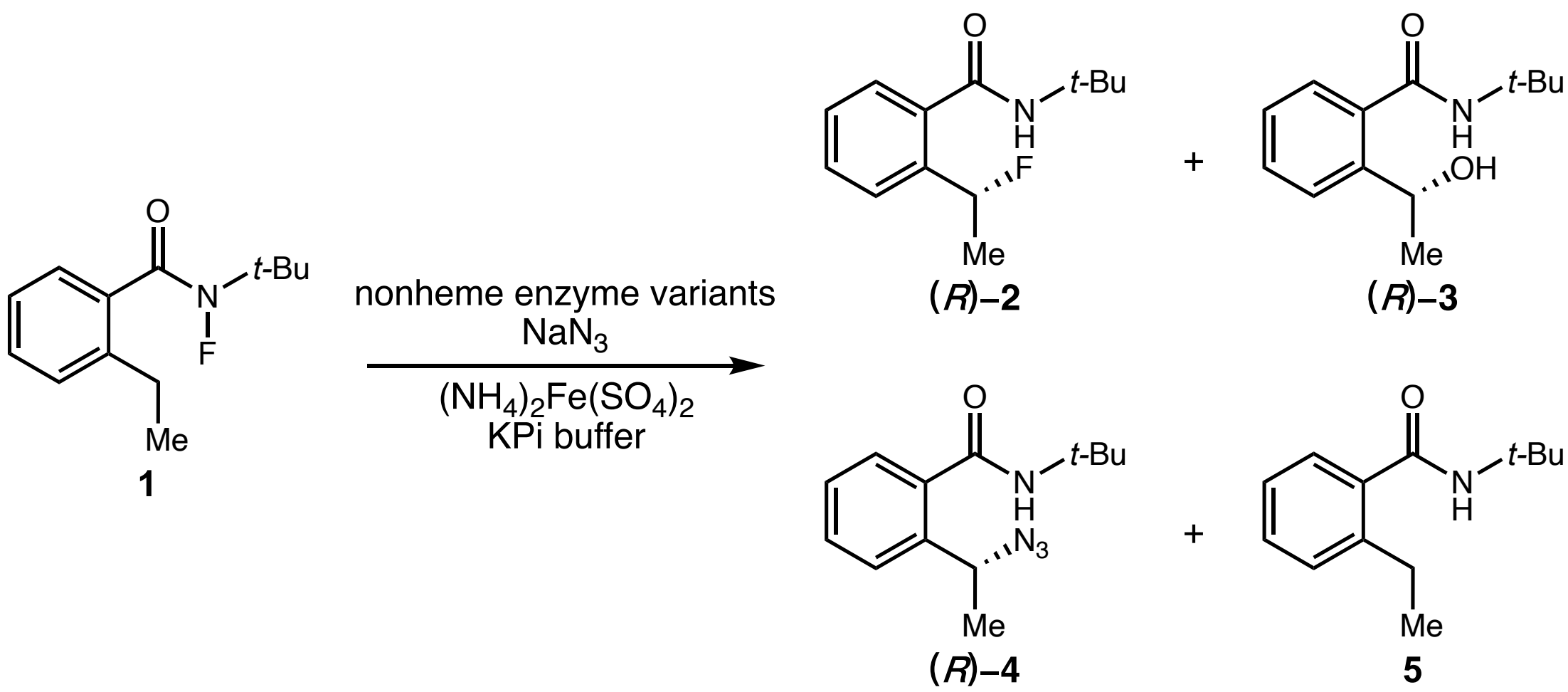

Table S16. pH effects on C–H azidation with $EgtB_{CHF1}$

entry	buffer pH	**2**		**3**		**4**		**5**
		yield (%)	e.r. ((*R*)-**2** : (*S*)-**2**)	yield (%)	e.r. ((*R*)-**3** : (*S*)-**3**)	yield (%)	e.r. ((*R*)-**4** : (*S*)-**4**)	yield (%)
1	6.5	13 ± 1	58:42	23 ± 2	24:76	52 ± 3	44:56	12 ± 1
2	7.0	14 ± 1	59:41	27 ± 1	23:77	55 ± 2	44:56	13 ± 1
3	7.5	14 ± 1	61:39	28 ± 1	21:79	53 ± 1	44:56	13 ± 1
4	8.0	14 ± 1	62:38	31 ± 1	19:81	47 ± 1	43:57	13 ± 1
5	8.5	14 ± 1	63:37	31 ± 1	18:82	49 ± 2	43:57	13 ± 1
6	9.0	14 ± 1	63:37	34 ± 1	19:81	50 ± 1	42:57	14 ± 1

Reaction conditions: 7.5 mM **1**, 120 mM NaN_3, 0.75 mM $(NH_4)_2Fe(SO_4)_2$, 7.5 mM sodium ascorbate, 500 µL cell-free lysate of $EgtB_{CHF1}$ variant, 100 mM KPi buffer. **2**: fluorination product, **3**: hydroxylation product, **4**: azidation product, **5**: reduction product.

Table S17. pH effects on C–H azidation with $EgtB_{CHF2}$

entry	buffer pH	**2**		**3**		**4**		**5**
		yield (%)	e.r. ((*R*)-**2** : (*S*)-**2**)	yield (%)	e.r. ((*R*)-**3** : (*S*)-**3**)	yield (%)	e.r. ((*R*)-**4** : (*S*)-**4**)	yield (%)
1	6.5	14 ± 1	25:75	10 ± 1	31:69	46 ± 3	38:62	18 ± 1
2	7.0	17 ± 1	28:72	15 ± 1	28:72	44 ± 1	38:62	13 ± 1
3	7.5	22 ± 4	25:75	11 ± 2	28:72	45 ± 3	38:62	17 ± 3
4	8.0	20 ± 1	29:71	16 ± 1	29:71	47 ± 1	37:63	15 ± 1
5	8.5	26 ± 3	26:74	14 ± 1	32:68	45 ± 1	38:62	18 ± 1
6	9.0	25 ± 2	28:72	17 ± 1	30:70	44 ± 1	38:62	19 ± 1

Reaction conditions: 7.5 mM **1**, 120 mM NaN_3, 0.75 mM $(NH_4)_2Fe(SO_4)_2$, 7.5 mM sodium ascorbate, 500 µL cell-free lysate of $EgtB_{CHF2}$ variant, 100 mM KPi buffer. **2**: fluorination product, **3**: hydroxylation product, **4**: azidation product, **5**: reduction product.

Table S18. pH effects on C–H azidation with $ACCO_{CHF}$

entry	buffer pH	**2**		**3**		**4**		**5**
		yield (%)	e.r. ((*R*)-**2** : (*S*)-**2**)	yield (%)	e.r. ((*R*)-**3** : (*S*)-**3**)	yield (%)	e.r. ((*R*)-**4** : (*S*)-**4**)	yield (%)
1	6.5	61 ± 4	10:90	0	-	15 ± 2	43:57	12 ± 2
2	7.0	63 ± 5	9:91	0	-	15 ± 2	42:58	13 ± 2
3	7.5	66 ± 1	7:93	0	-	15 ± 1	42:58	13 ± 3
4	8.0	74 ± 4	7:93	0	-	16 ± 2	42:58	14 ± 2
5	8.5	75 ± 3	9:91	0	-	16 ± 2	41:59	14 ± 2
6	9.0	79 ± 2	8:92	0	-	17 ± 1	41:59	15 ± 1

Reaction conditions: 7.5 mM **1**, 120 mM NaN_3, 0.75 mM $(NH_4)_2Fe(SO_4)_2$, 500 µL whole-cell suspension of $ACCO_{CHF}$ variant, 100 mM KPi buffer. **2**: fluorination product, **3**: hydroxylation product, **4**: azidation product, **5**: reduction product.

VIII. Synthesis and characterization of substrates and products.

Synthetic procedures to prepare substrates and racemic products.

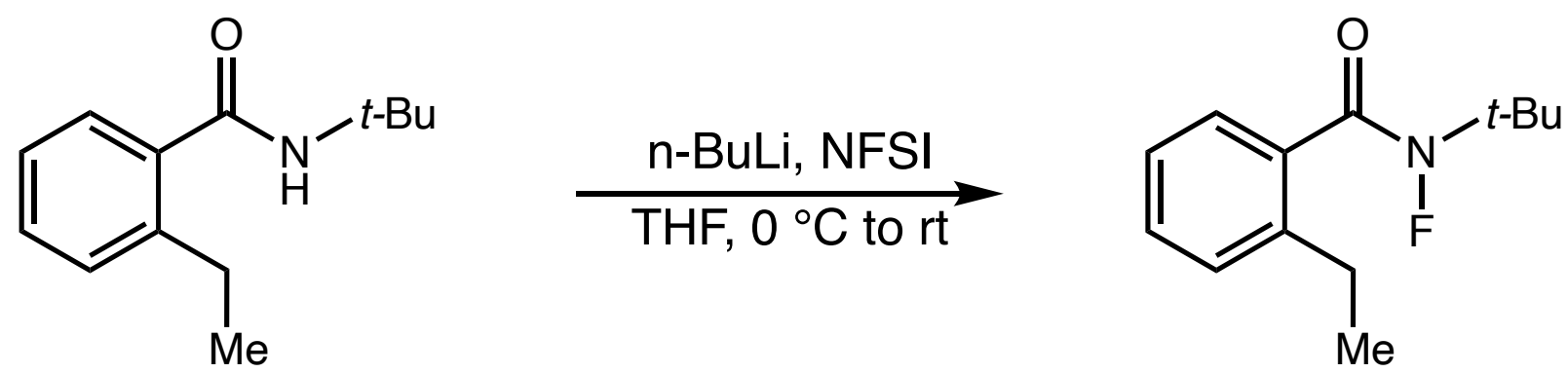

This procedure is modified from a previously described procedure.[5] A round bottom flask was dried in an oven (110 °C) and charged with a stir bar. This flask was evacuated and backfilled with N_2 and this procedure was repeated for a total of three times. To this flask were added the amide (17.8 g, 86.7 mmol, 1.0 equiv) and anhydrous THF (300 mL, 0.29 M). The mixture was cooled to 0 °C in an ice bath, and *n*-BuLi (38.2 mL, 2.5 M in hexanes, 1.1 equiv) was added dropwise. The reaction mixture was allowed to stir at 0 °C for 0.5 h before the addition of a solution of NFSI (41.0 g, 130 mmol, 1.5 equiv) in THF (180 mL). The reaction mixture was allowed to warm to room temperature slowly and stirred overnight. Saturated aq. $NaHCO_3$ (200 mL) was then added, and the reaction mixture was extracted with EtOAc (3 × 300 mL). The combined organic phases were washed with brine (200 mL), dried over anhydrous Na_2SO_4, and concentrated under vacuum with the aid of a rotary evaporator. The residue was purified by Biotage (100 g SNAP cartridge, 0-4% EtOAc/hexanes for 8 CV, then 4% EtOAc/hexanes for 10 CV) to afford the product as a pale-yellow oil (7.9 g, 45% yield).

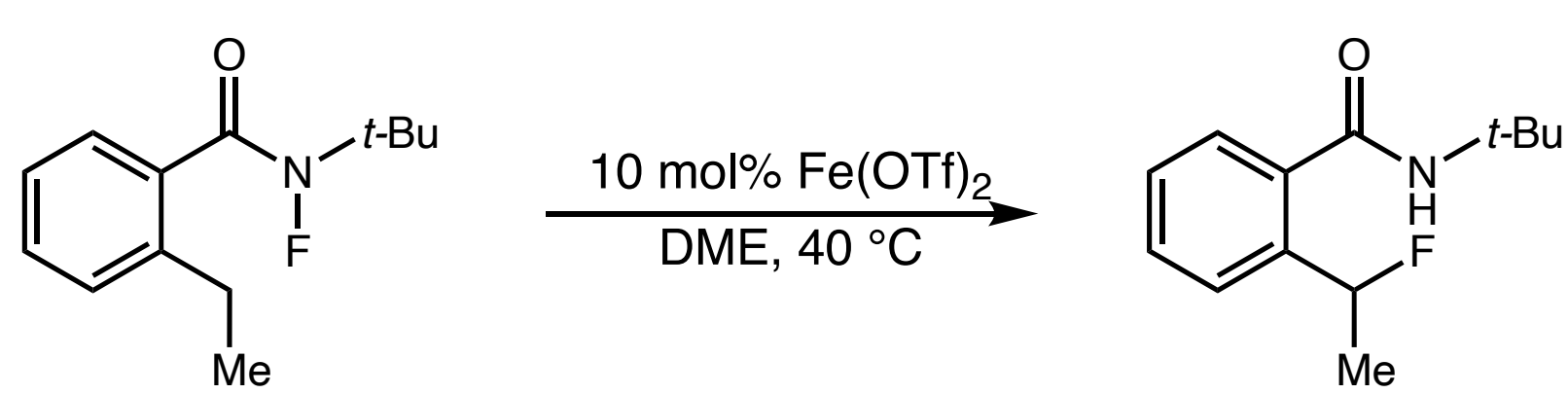

A round bottom flask was dried in an oven (110 °C) and charged with a stir bar. This flask was evacuated and backfilled with N_2 and this procedure was repeated for a total of three times. To this flask were added anhydrous $Fe(OTf)_2$ (318 mg, 0.898 mmol, 0.2 equiv) and a solution of fluoroamide (1.00 g, 4.49 mmol, 1.0 equiv) in anhydrous DME (22 mL, 0.2 M). The mixture was heated to 40 °C for 4 h. Then the mixture was diluted with 22 mL EtOAc and filtered through a

silica plug and eluded with EtOAc (3 × 20 mL). The filtrate was concentrated under vacuum with the aid of a rotary evaporator. The residue was purified by Biotage (50 g SNAP cartridge, 0-10% EtOAc/hexanes for 8 CV, then 10% EtOAc/hexanes for 5 CV) to afford the fluoride product as a white powder (831 mg, 83% yield).

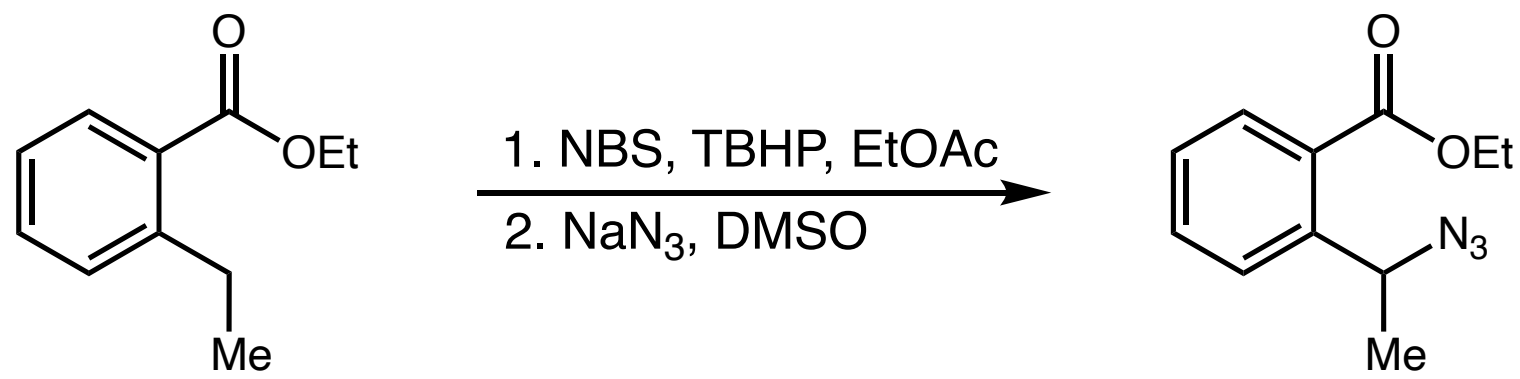

To a solution of methyl 2-ethylbenzoate (2.67 g, 15.0 mmol, 1.0 equiv) in ethyl acetate (30 mL, 0.2 M) was added *N*-bromosuccinimide (NBS, 3.20 g, 18.0 mmol, 1.2 equiv) and benzoyl peroxide (BPO, 0.440 g, 1.80 mmol, 0.12 eq). The mixture was purged with N_2 for 10 min, refluxed for 2 h, and then cooled to room temperature. 50 mL water was added, the organic layer was separated, and the reaction mixture was extracted with EtOAc (3 × 50 mL). The combined organic layers were dried over anhydrous Na_2SO_4 and concentrated under vacuum with the aid of a rotary evaporator. The crude product was dissolved in DMSO (30 mL, 0.2 M). NaN_3 (1.07 g, 16.5 mmol, 1.1 equiv) was then added. The mixture was allowed to stir at 22 °C for 12 h before saturated aq. $NaHCO_3$ (50 mL) was added, and the reaction mixture was extracted with EtOAc (50 mL) for a total of three times. The combined organic phases were washed with brine (50 mL), dried over anhydrous Na_2SO_4, and concentrated under vacuum with the aid of a rotary evaporator. The residue was purified by Biotage (50 g SNAP cartridge, 0-5% EtOAc/hexanes for 8 CV, then 5% EtOAc/hexanes for 10 CV) to afford the azide product as a colorless oil (2.46 g, 75% two steps). ^{1}H NMR (400 MHz, $CDCl_3$) δ: 7.91 (dd, J = 7.9, 1.2 Hz, 1H), 7.61 (dd, J = 7.9, 1.4 Hz, 1H), 7.55 (td, J = 7.7, 1.4 Hz, 1H), 7.35 (td, J = 7.8, 1.4 Hz, 1H), 5.68 (q, J = 6.7 Hz, 1H), 4.37 (q, J = 7.1 Hz, 2H), 1.53 (d, J = 6.7 Hz, 3H), 1.40 (t, J = 7.1 Hz, 3H) ppm. ^{13}C NMR (101 MHz, $CDCl_3$) δ: 167.1, 143.2, 132.7, 130.6, 128.9, 127.6, 126.9, 61.3, 57.4, 22.4, 14.3 ppm.

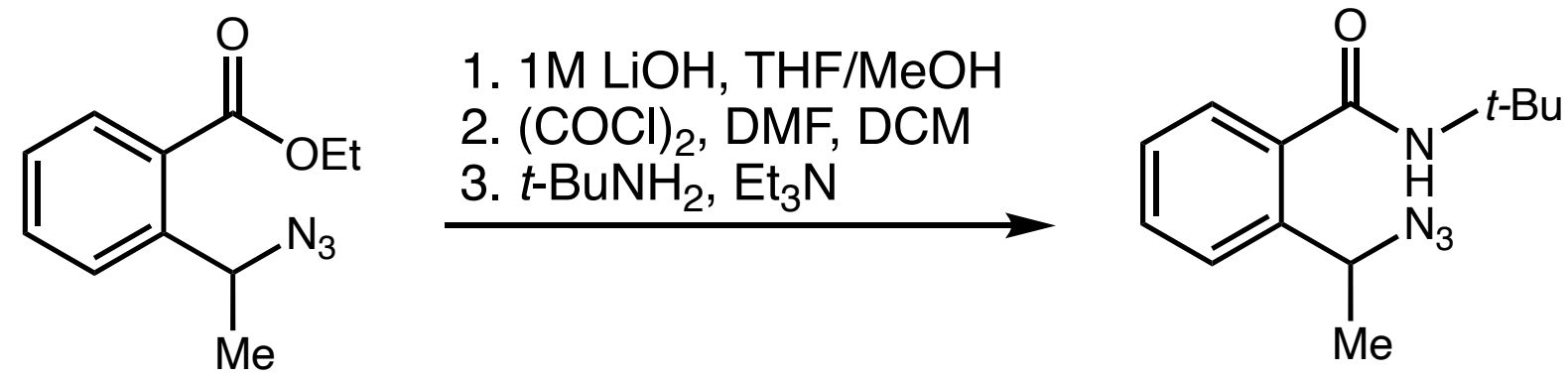

To a stirred solution of azide (500 mg, 2.28 mmol, 1.0 equiv) in THF/MeOH (20 mL, 1:1) was added aq. LiOH (12 mL, 1.0 M) slowly at 0 °C. The mixture was allowed to warm to room temperature for 2 h before aq. HCl (15 mL, 1.0 M) was added. The mixture was extracted with EtOAc (3 × 20 mL). The combined organic phases were washed with brine (20 mL), dried over anhydrous Na_2SO_4, and concentrated under vacuum with the aid of a rotary evaporator. The crude product was used directly for the next step without further purification.

To a stirred solution of the benzoic acid (330 mg, 1.72 mmol, 1.0 equiv) in CH_2Cl_2 (10 mL, 0.17 M) was added one drop *N,N*-dimethylformamide and oxalyl chloride (0.17 mL, 2.06 mmol, 1.50 equiv). The reaction was stirred at room temperature for 1 h. To another flask with a stir bar were added *tert*-butylamine (0.26 mL, 2.58mmol, 1.5 equiv), triethylamine (0.62 mL, 4.3 mmol, 2.5 equiv) and CH_2Cl_2 (5 mL, 0.3 M) at 0 ºC. The freshly prepared acid chloride was slowly added into this flask. The reaction mixture was allowed to warm to room temperature and stirred for another 1 h before aq. HCl (5 mL, 1.0 M) was added. The resultant mixture was extracted with EtOAc (3 × 10 mL). The combined organic phases were washed with brine (20 mL), dried over anhydrous Na_2SO_4, and concentrated under vacuum with the aid of a rotary evaporator. The residue was purified by Biotage (10 g SNAP cartridge, 0-10% EtOAc/hexanes for 8 CV, then 10% EtOAc/hexanes for 10 CV) to afford the amide product as a white solid (130 mg, 28% over two steps).

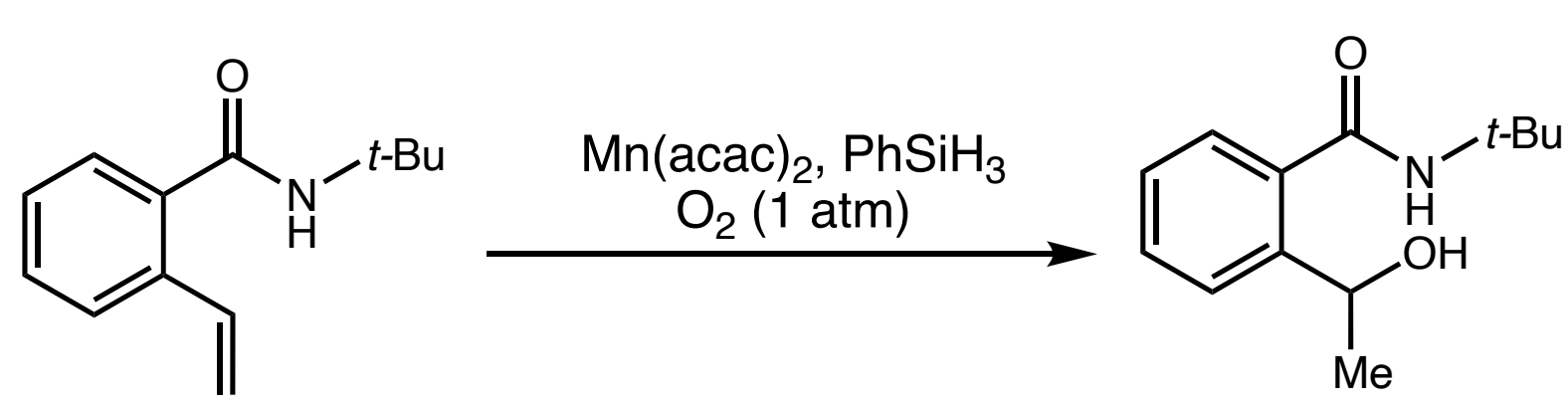

To a stirred solution of the styrene (1.11 g, 5.46 mmol, 1.0 equiv) and $PhSiH_3$ (3.4 mL, 27.3 mmol, 5 equiv) in EtOH (25 mL, 0.2 M) was added $Mn(acac)_2$ (138 mg, 0.546 mmol, 10 mol%)

at 22 °C. Oxygen was then bubbled into the mixture for 10 min. The mixture was then allowed to stir under an oxygen atmosphere at 22 °C for 5 h. The volatiles were removed under vacuum, and the residue was purified by Biotage (50 g SNAP cartridge, 0-30% EtOAc/hexanes for 8 CV, then 30% EtOAc/hexanes for 10 CV) to afford the alcohol as a colorless oil (800 mg, 66%).

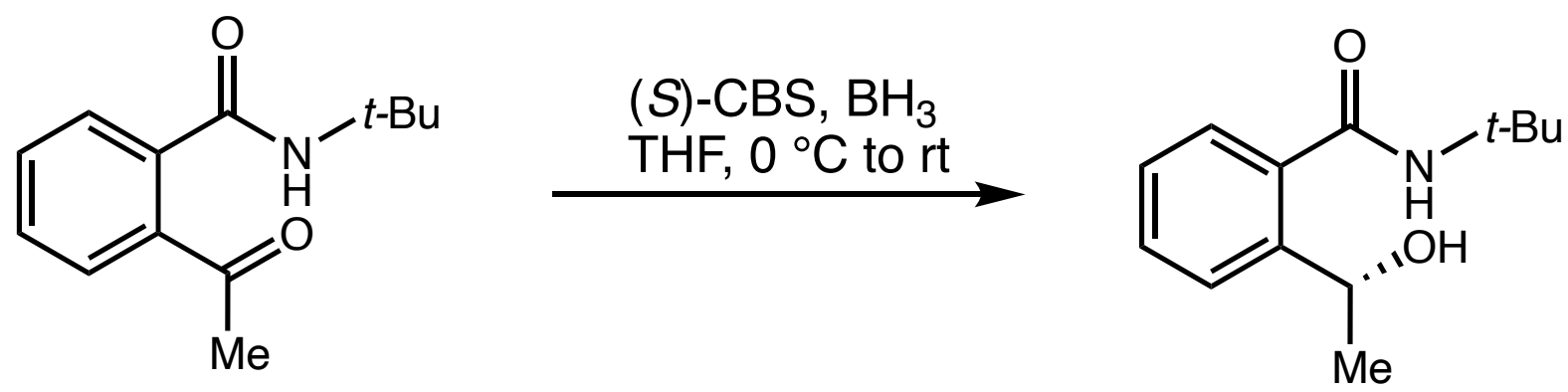

This procedure is modified from a previously described procedure, and the (*R*)-product was obtained in accordance with the Corey-Bakshi-Shibata reduction and its stereochemistry outcome.[6] To a stirred solution of (*S*)-1-Methyl-3,3-diphenylhexahydropyrrolo[1,2-c][1,3,2]oxazaborole (219 mg, 1.0 mmol, 1.0 equiv) and BH_3 in THF (1 M in THF, 1.5 mL, 1.5 mmol, 1.5 equiv) in THF (5 mL, 0.2 M) was added the ketone (219 mg, 1.0 mmol, 1.0 equiv) at 0 °C. The reaction mixture was allowed to warm to room temperature and stirred for another 6 h before 3 mL aq. HCl (1.0 M) was added. The resulting mixture was extracted with EtOAc (3 × 10 mL). The combined organic phases were washed with brine (20 mL), dried over anhydrous Na_2SO_4, and concentrated under vacuum with the aid of a rotary evaporator. The residue was purified by Biotage (10 g SNAP cartridge, 0-20% EtOAc/hexanes for 10 CV, then 20% EtOAc/hexanes for 5 CV) to afford the amide product as a white solid (88 mg, 40% yield, 60:40 e.r.). The absolute stereochemistry was established based on assignments in literature.[6]

Characterization data of substrates and products

***N*-(*Tert*-butyl)-2-ethyl-*N*-fluorobenzamide (1)**

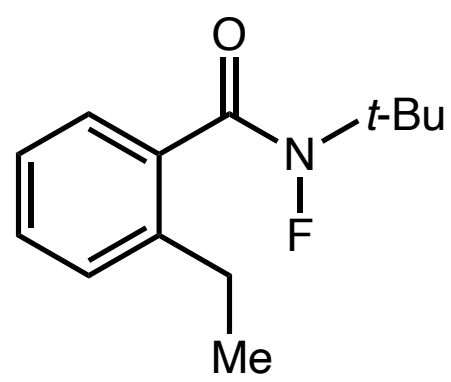

^{1}H NMR (400 MHz, $CDCl_3$) δ: 7.35 (ddd, *J* = 7.6, 7.6, 1.5 Hz, 1H), 7.33 – 7.25 (m, 1H), 7.26 (s, 1H), 7.21 (ddd, *J* = 7.4, 7.4, 1.3 Hz, 1H), 2.74 (q, *J* = 7.6 Hz, 1H), 1.56 (d, *J* = 2.0 Hz, 9H), 1.24 (t, *J* = 7.6 Hz, 3H) ppm. ^{13}C NMR (101 MHz, $CDCl_3$) δ: 175.2 (d, *J* = 11.0 Hz), 141.7 (d, *J* = 2.2 Hz), 134.8, 130.2 (d, *J* = 1.4 Hz), 129.0, 127.2 (d, *J* = 4.4 Hz), 125.5, 64.4 (d, *J* = 10.5 Hz), 27.3 (d, *J* = 5.7 Hz), 26.2, 15.8 ppm.^{19}F NMR (376 MHz, $CDCl_3$) δ: -63.4 (s) ppm. Spectral data match those previously reported.[5]

***N*-(*Tert*-butyl)-2-(1-fluoroethyl)benzamide (2)**

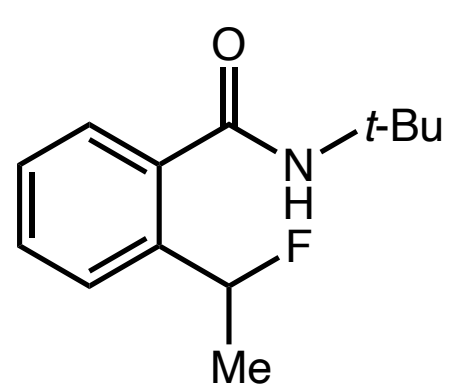

^{1}H NMR (400 MHz, $CDCl_3$) δ: 7.57 (d, *J* = 7.7 Hz, 1H), 7.45 (ddd, *J* = 7.5, 7.5, 1.6 Hz, 1H), 7.39 – 7.28 (m, *2*H), 6.03 (dq, *J* = 47.7, 6.3 Hz, 1H), 5.77 (s, 1H), 1.68 (dd, *J* = 24.1, 6.3 Hz, 3H), 1.45 (s, 9H) ppm. ^{13}C NMR (101 MHz, $CDCl_3$) δ: 168.7, 140.0 (d, *J* = 19.4 Hz), 135.7 (d, *J* = 5.1 Hz), 130.3, 128.1 (d, *J* = 1.9 Hz), 126.6, 125.9 (d, *J* = 8.9 Hz), 88.8 (d, *J* = 165.1 Hz), 52.1, 28.9, 23.2 (d, *J* = 25.3 Hz) ppm.^{19}F NMR (376 MHz, $CDCl_3$) δ: -166.58 (dq, *J* = 48.4, 24.1 Hz) ppm. Spectral data match those previously reported.[5]

For analytical scale biotransformation of substrate **1**, the HPLC analysis conditions are as follows: IC column, 97:3 hexanes: *i*-PrOH, 1.2 mL/min, 40 °C.

For biocatalytic reaction with $EgtB_{CHF1}$, HPLC analysis indicated 31% yield, 60:40 e.r.: t_R = 9.74 (*R*, major), 10.38 (*S*, minor) min.

For biocatalytic reaction with $EgtB_{CHF2}$, HPLC analysis indicated 36% yield, 31:69 e.r.: t_R = 9.75 (*R*, minor), 10.37 (*S*, major) min.

For biocatalytic reaction with $ACCO_{CHF}$, HPLC analysis indicated 89% yield, 5:95 e.r.: t_R = 9.56 (*R*, minor), 10.12 (*S*, major) min.

N-(tert-butyl)-2-(1-hydroxyethyl)benzamide (3)

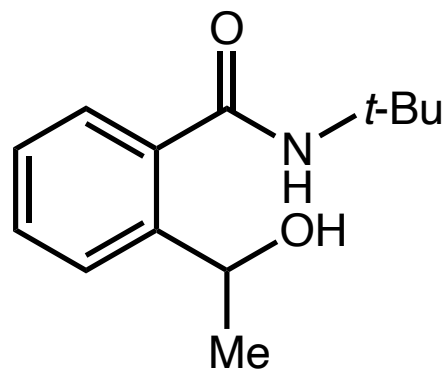

^{1}H NMR (400 MHz, $CDCl_3$) δ: 7.47 – 7.33 (m, 3H), 7.29 – 7.23 (m, 1H), 6.06 (s, 1H), 4.91 (q, J = 6.6 Hz, 1H), 1.53 (d, J = 6.6 Hz, 3H), 1.46 (s, 9H). ^{13}C NMR (126 MHz, $CDCl_3$) δ: 170.7, 143.4, 136.6, 130.4, 127.4, 127.4, 126.9, 68.1, 52.1, 28.7, 22.0 ppm. IR: ν: 3280, 2970, 1633, 1539, 1452, 1393, 1364, 1313, 1221, 1076, 761, 665 cm^{-1}. HRMS (*m/z*): $[M + Na]^+$ calcd for $C_{13}H_{19}N_1O_2Na^+$ 244.1313, found 244.1314.

For analytical scale biotransformation of substrate **1**, the HPLC analysis conditions are as follows: IB-N5 column, 98:2 hexanes: *i*-PrOH, 1.2 mL/min, 40 °C.

For biocatalytic reaction with $EgtB_{CHF1}$, HPLC analysis indicated 32% yield, 75:25 e.r.: t_R = 10.41 (major, *S*), 11.78 (minor, *R*) min.

For biocatalytic reaction with $EgtB_{CHF2}$, HPLC analysis indicated 17% yield, 66:34 e.r.: t_R = 10.28 (major, *S*), 11.57 (minor, *R*) min.

For CBS reduction, HPLC analysis indicated 40:60 e.r. and allowed the establishment of absolute configuration based on literature report[6]: t_R = 10.56 (minor, *S*), 11.588 (major, *R*) min.

2-(1-Azidoethyl)-N-(tert-butyl)benzamide (4)

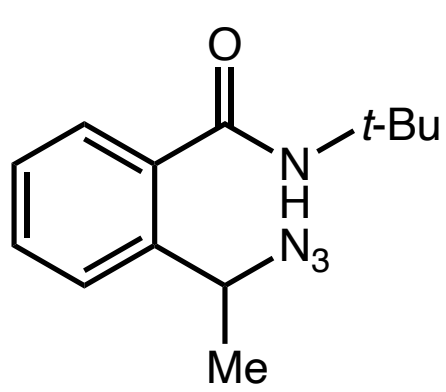

^{1}H NMR (400 MHz, $CDCl_3$) δ: 7.50 (dd, J = 7.8, 1.4 Hz, 1H), 7.44 (ddd, J = 7.9, 7.5, 1.6 Hz, 1H), 7.37 (dd, J = 7.6, 1.6 Hz, 1H), 7.31 (td, J = 7.4, 1.4 Hz, 1H), 5.76 (s, 1H), 5.15 (q, J = 6.8 Hz, 1H), 1.58 (d, J = 5.9 Hz, 3H), 1.47 (s, 9H) ppm. ^{13}C NMR (101 MHz, $CDCl_3$) δ: 168.9, 139.1, 136.9, 130.4, 128.0, 127.0, 126.7, 57.5, 52.2, 28.9, 21.5 ppm. Spectral data match those previously reported.[7]

For analytical scale biotransformation of substrate **1**, the HPLC analysis condition are as follows: IC column, 97:3 hexanes: *i*-PrOH, 1.2 mL/min, 40 °C.

For biocatalytic reaction with $EgtB_{CHF1}$, HPLC analysis indicated 50% yield, 56:44 e.r.: t_R = 7.05 (*S*, major), 7.62 (*R*, minor) min.

For biocatalytic reaction with $EgtB_{CHF2}$, HPLC analysis indicated 40% yield, 61:39 e.r.: t_R = 7.16 (*S*, major), 7.74 (*R*, minor) min.

For biocatalytic reaction with $EgtB_{CHF2}$ H138A, HPLC analysis indicated 42% yield, 73:27 e.r.: t_R = 7.17 (*S*, major), 7.76 (*R*, minor) min.

IX. HPLC calibration curves

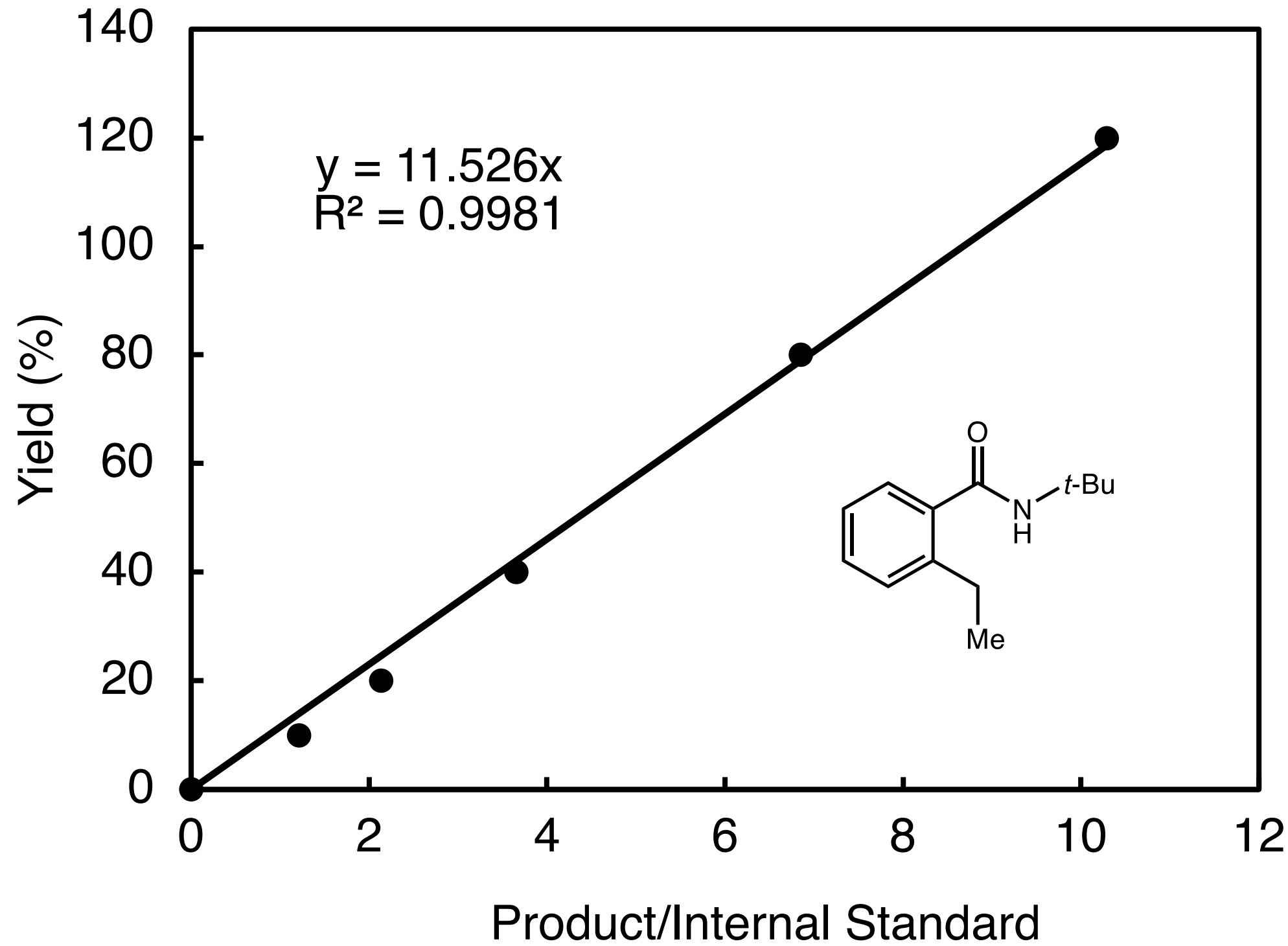

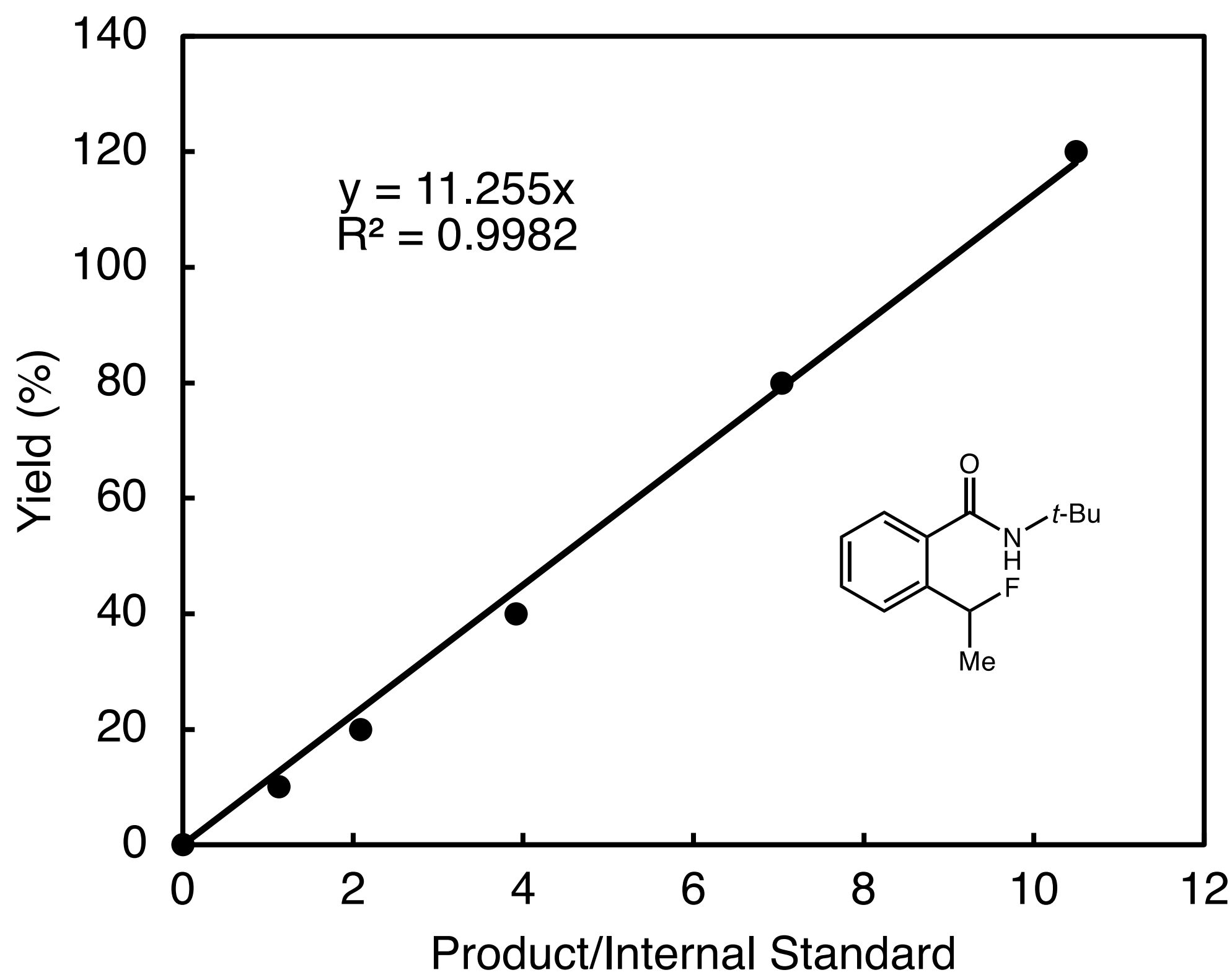

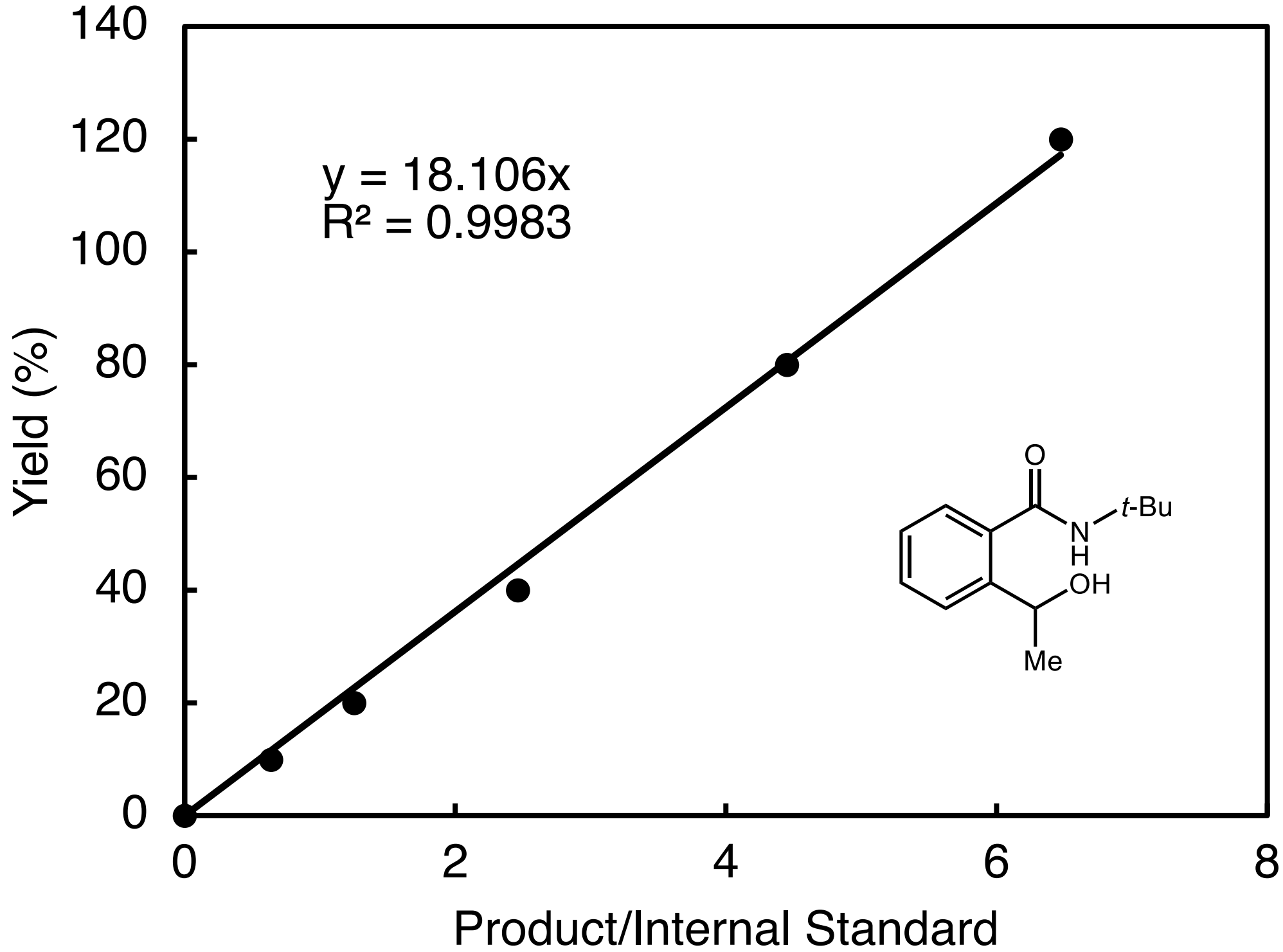
y = 18.106x
R² = 0.9983
Yield (%)
Product/Internal Standard
O
N
H
t-Bu
OH
Me

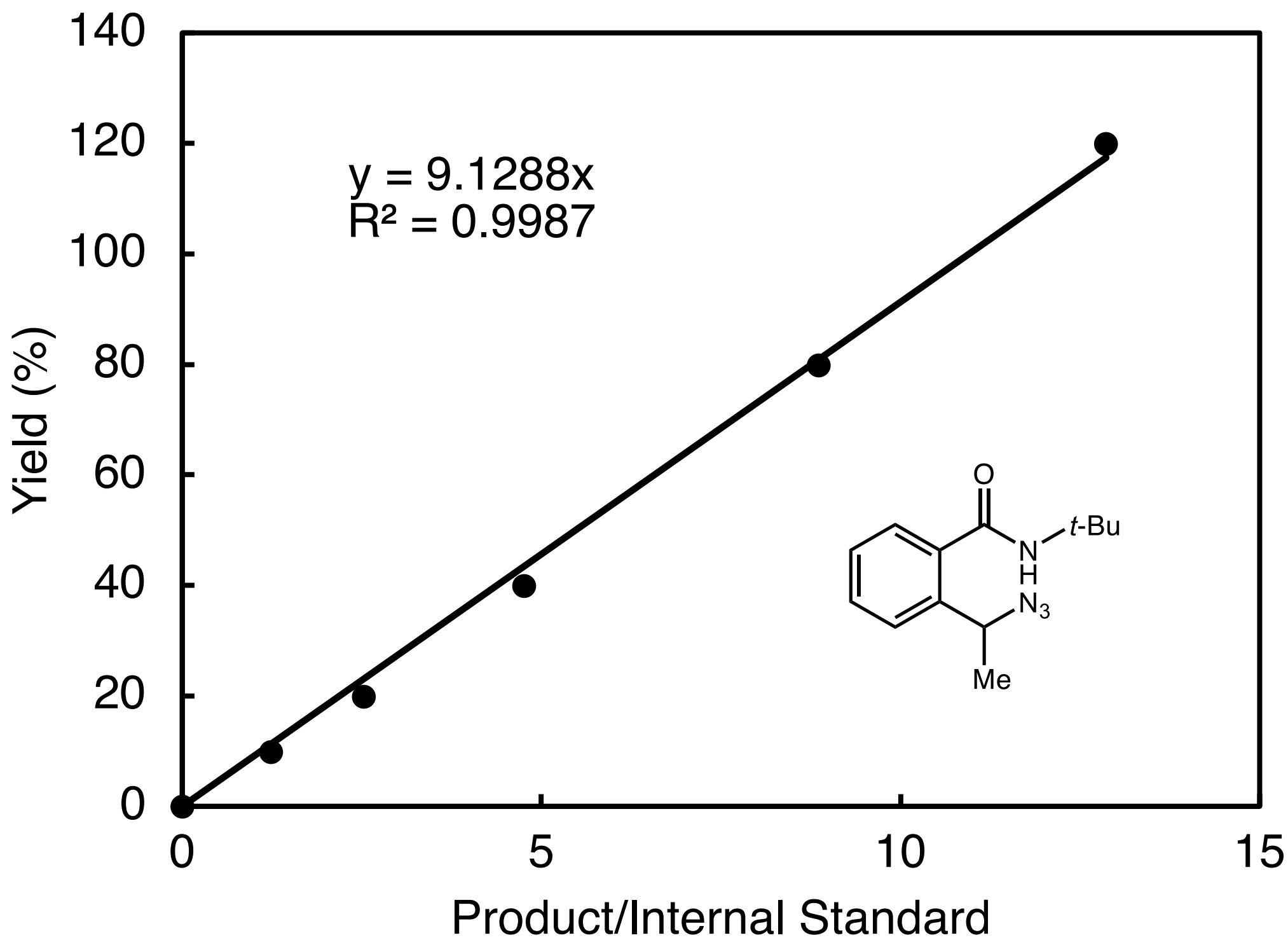
y = 9.1288x
R² = 0.9987
Yield (%)
Product/Internal Standard
O
N
H
t-Bu
N3
Me

X. Chiral HPLC traces

N-(*tert*-butyl)-2-(1-fluoroethyl)benzamide (2)

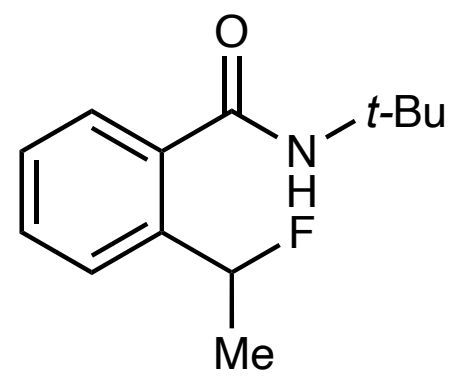

Racemic **2**:

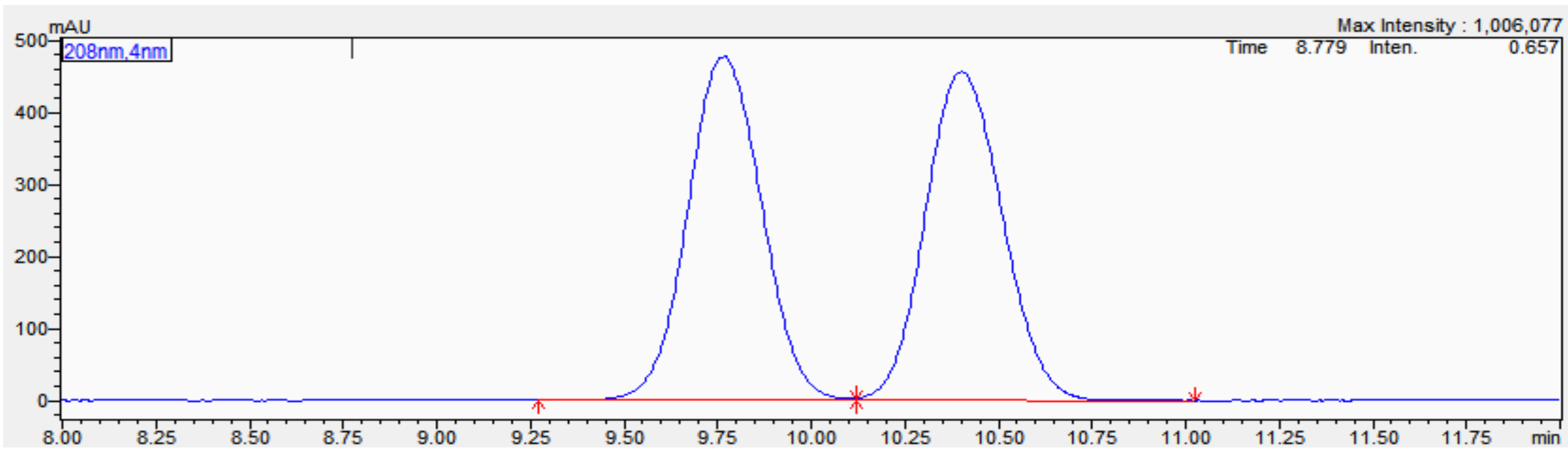

Peak#	Ret. Time	Height	Area	Area%
1	9.768	477341	6585286	50.151
2	10.403	456823	6545684	49.849
Total		934164	13130970	100.000

Enantioenriched **2** obtained using $EgtB_{CHF1}$ (HPLC analysis): 60:40 e.r.

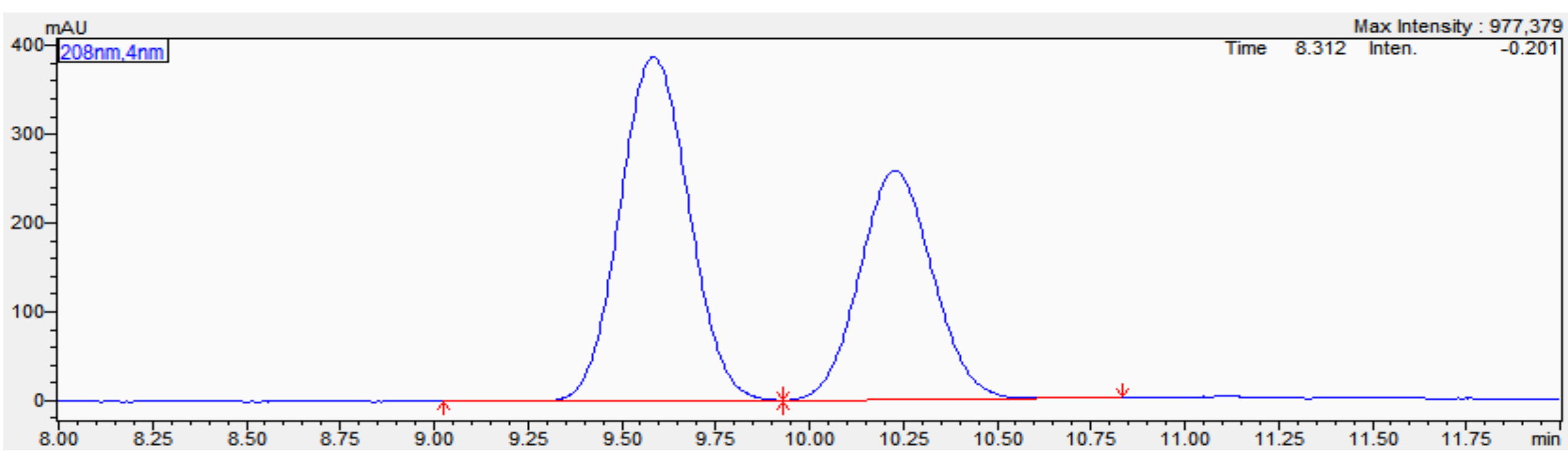

Peak#	Ret. Time	Height	Area	Area%
1	9.738	396542	5096452	59.984
2	10.384	255957	3399952	40.016
Total		652499	8496404	100.000

Enantioenriched **2** obtained using EgtB$_{CHF2}$ (HPLC analysis): 31:69 e.r.

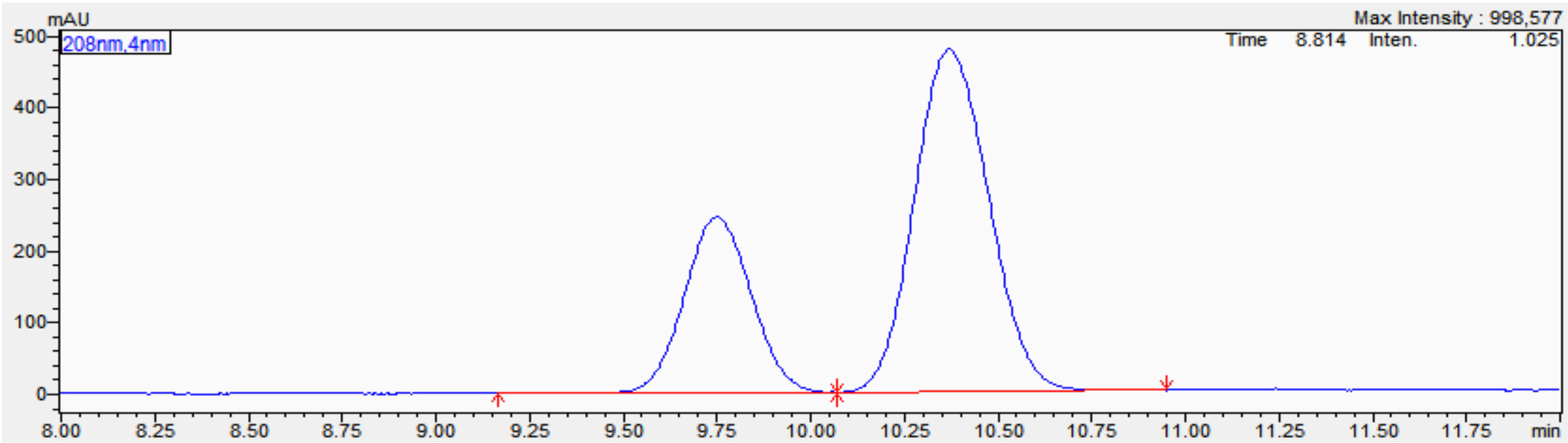

Peak#	Ret. Time	Height	Area	Area%
1	9.751	245367	3026199	31.425
2	10.370	477051	6603864	68.575
Total		722419	9630064	100.000

Enantioenriched **2** obtained using ACCO$_{CHF}$ (HPLC analysis): 5.5:94.5 e.r.

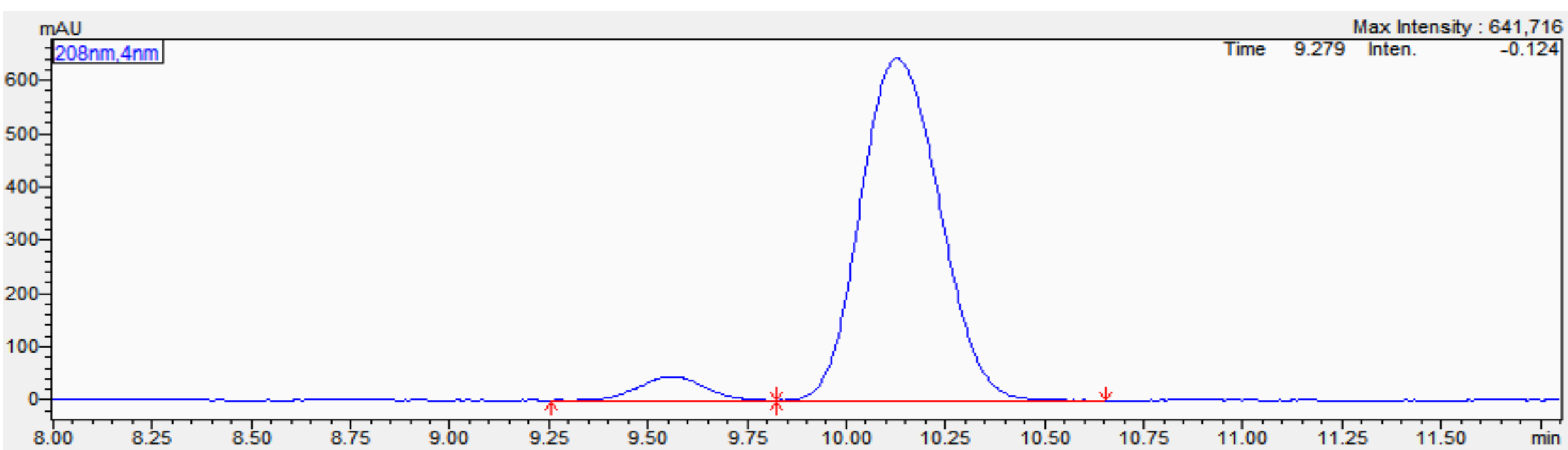

Peak#	Ret. Time	Height	Area	Area%
1	9.561	44496	523733	5.558
2	10.127	642184	8899936	94.442
Total		686681	9423669	100.000

***N*-(*tert*-butyl)-2-(1-hydroxyethyl)benzamide** (**3**)

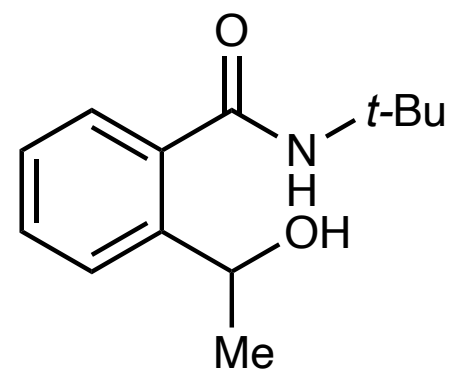

Racemic **3:**

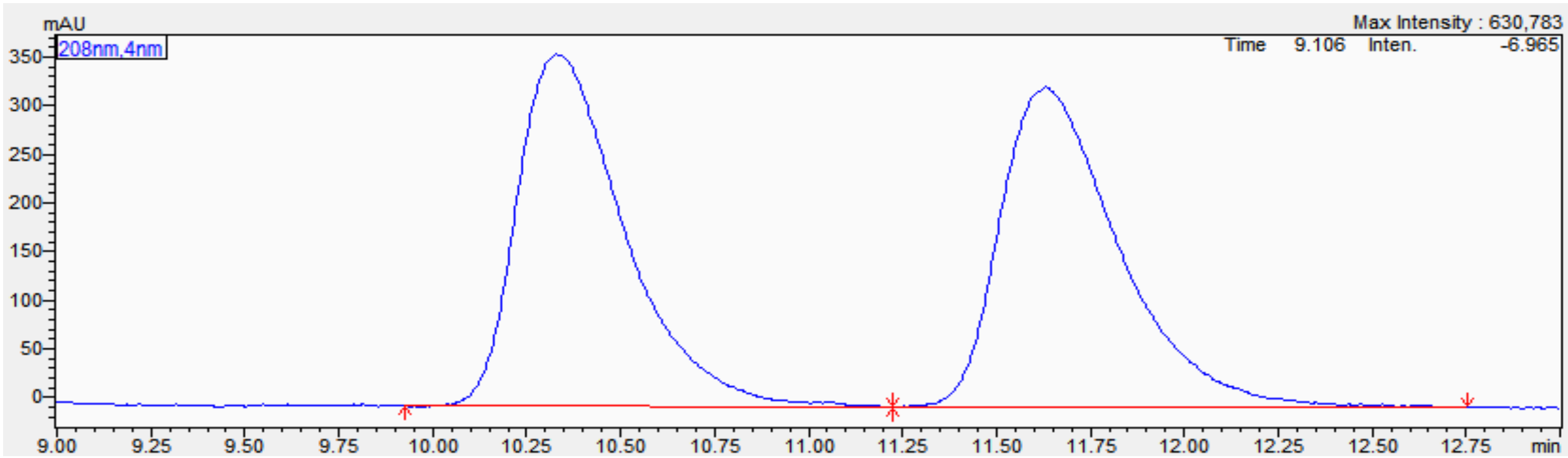

Peak#	Ret. Time	Height	Area	Area%
1	10.330	361986	7249855	49.904
2	11.631	329963	7277751	50.096
Total		691949	14527606	100.000

Enantioenriched **3** obtained using $EgtB_{CHF1}$ (HPLC analysis):75:25 e.r.

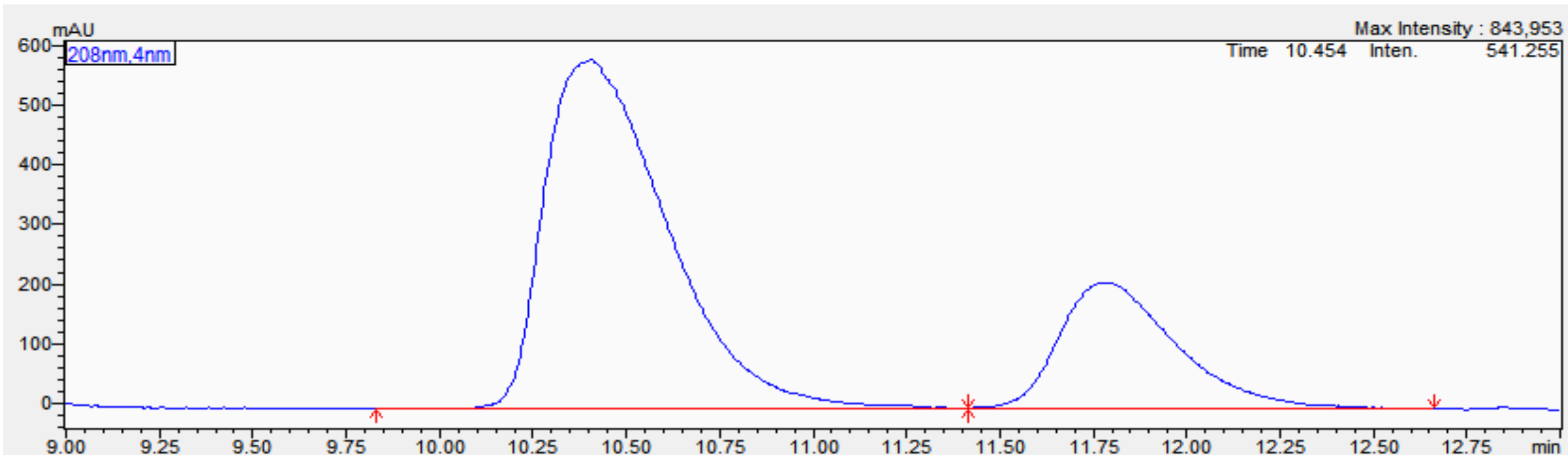

Peak#	Ret. Time	Height	Area	Area%
1	10.405	584949	13314664	74.564
2	11.780	210188	4542003	25.436
Total		795138	17856667	100.000

Enantioenriched **3** obtained using $EgtB_{CHF2}$ (HPLC analysis): 66:34 e.r.

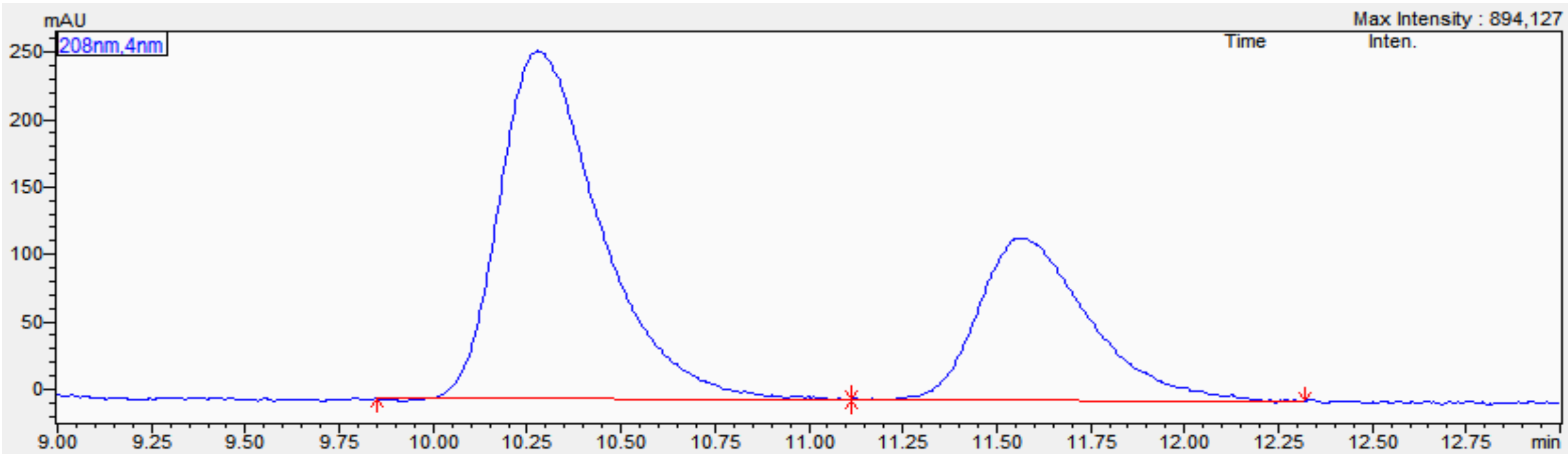

Peak#	Ret. Time	Height	Area	Area%
1	10.279	258023	4814053	66.358
2	11.573	120106	2440602	33.642
Total		378129	7254654	100.000

Enantioenriched **3** obtained via CBS reduction (HPLC analysis): 40:60 e.r.

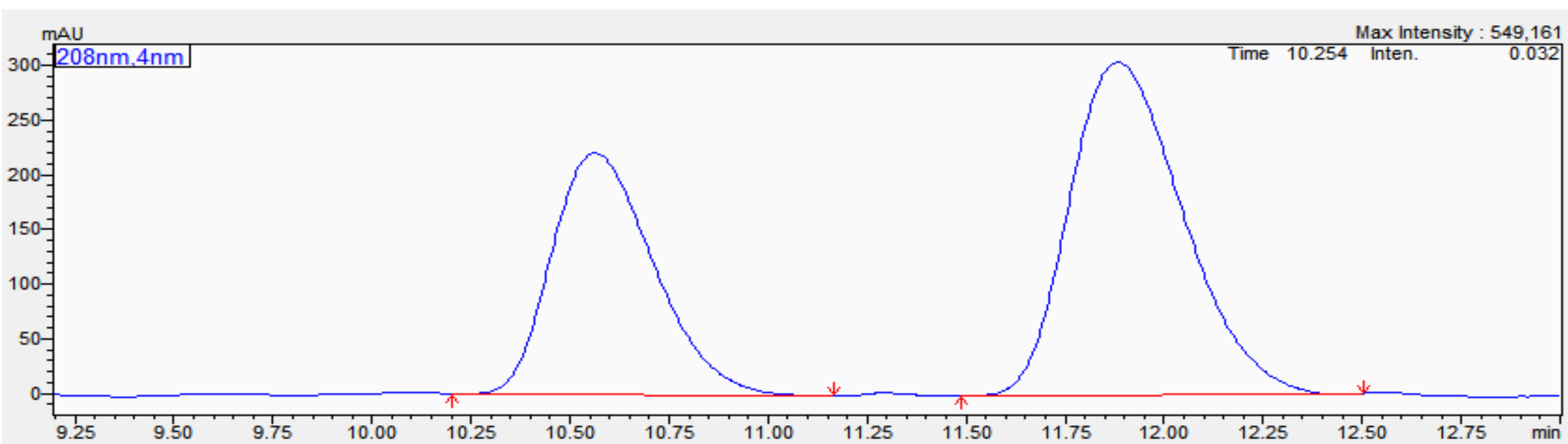

Peak#	Ret. Time	Height	Area	Area%
1	10.563	220395	3929951	39.411
2	11.884	303573	6041720	60.589
Total		523968	9971671	100.000

2-(1-azidoethyl)-*N*-(*tert*-butyl)benzamide (4)

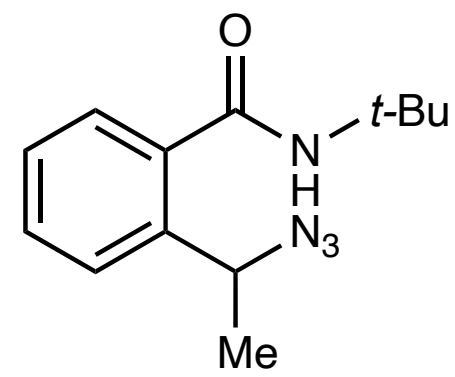

Racemic **4**:

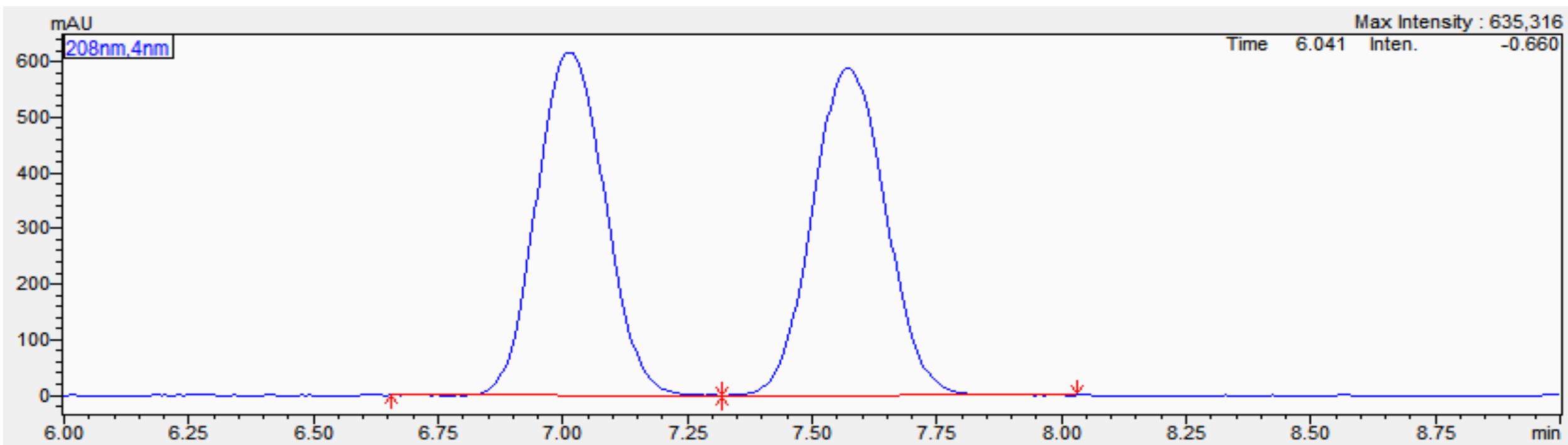

Peak#	Ret. Time	Height	Area	Area%
1	7.014	615700	5903252	49.427
2	7.572	589108	6040034	50.573
Total		1204808	11943286	100.000

Enantioenriched **4** obtained using $EgtB_{CHF1}$ (HPLC analysis): 56:44 e.r.

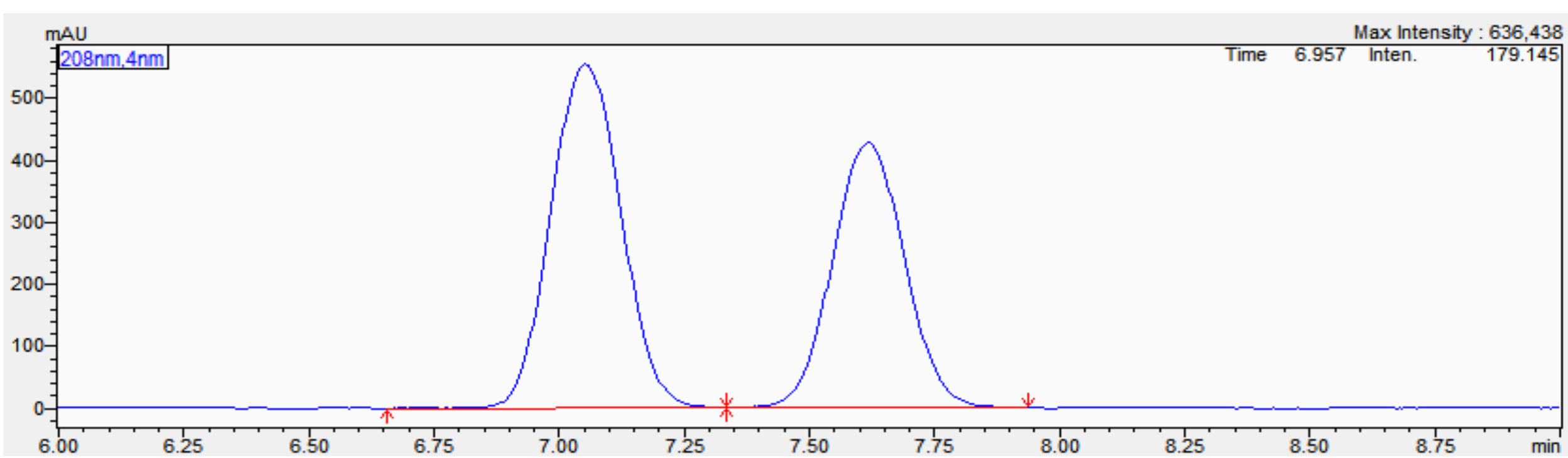

Peak#	Ret. Time	Height	Area	Area%
1	7.052	554920	5447961	55.841
2	7.617	427933	4308165	44.159
Total		982853	9756126	100.000

Enantioenriched **4** obtained using $EgtB_{CHF2}$ (HPLC analysis): 61:39 e.r.

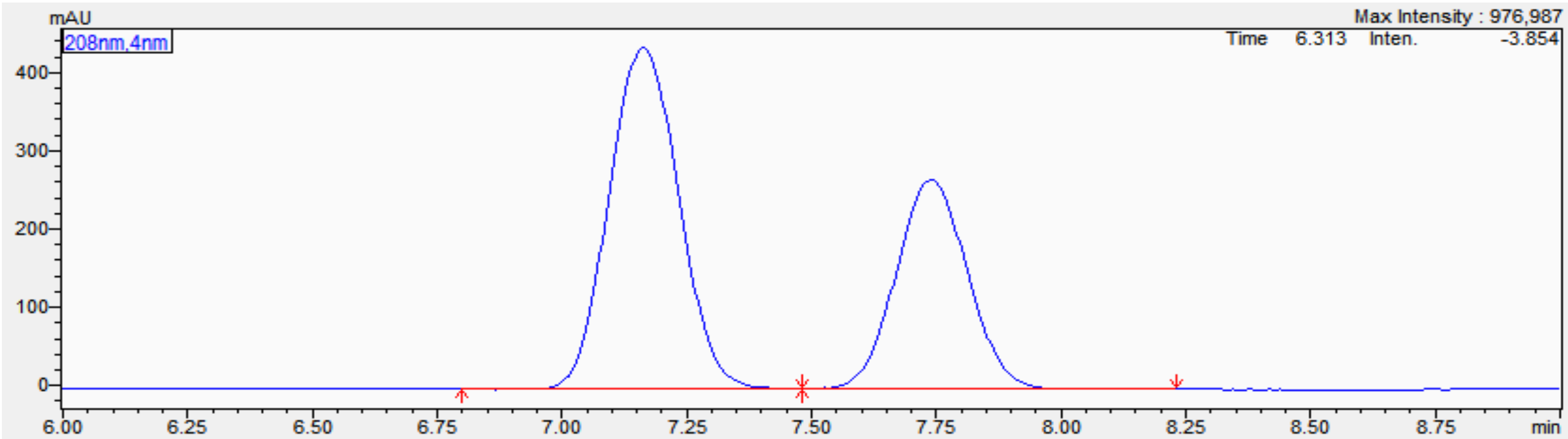

Peak#	Ret. Time	Height	Area	Area%
1	7.163	435750	4252681	61.494
2	7.740	267127	2662953	38.506
Total		702877	6915634	100.000

Enantioenriched **4** obtained using $EgtB_{CHF2}$ H138A (HPLC analysis): 73:27 e.r.

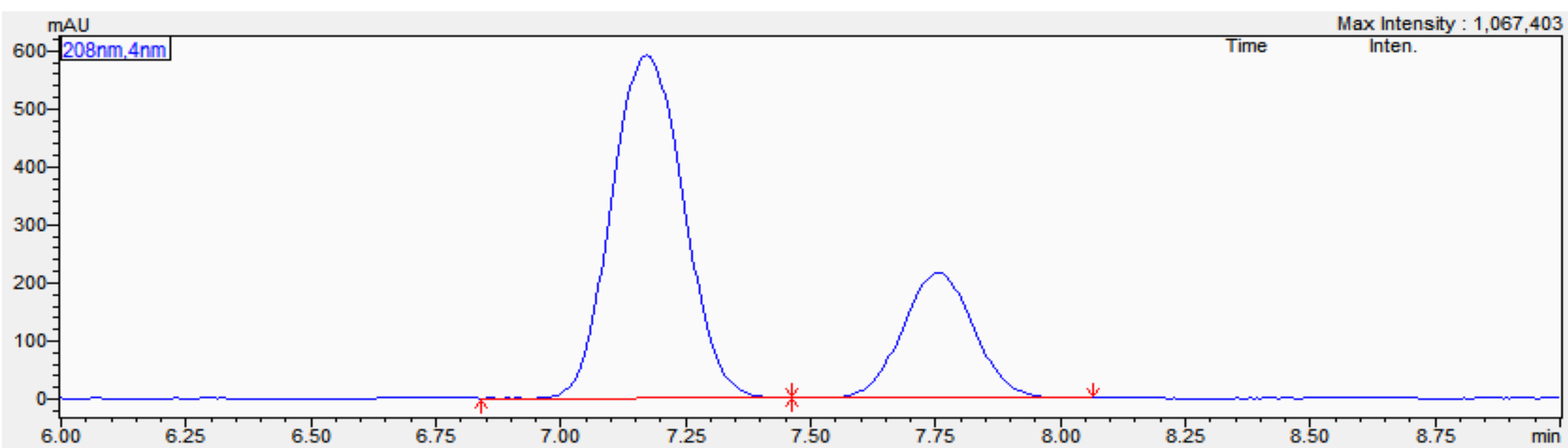

Peak#	Ret. Time	Height	Area	Area%
1	7.172	591130	5939553	73.479
2	7.755	216100	2143791	26.521
Total		807230	8083344	100.000

XI. Computational Details

1. Classical Molecular Dynamics (MD) Simulations

Classical MD simulations were performed to study the binding modes of *N*-fluoramide substrate (**1**) and active site residue–substrate interactions that promote the fluorine atom transfer from the substrate to the Fe(II) center. The starting structure of *Mth*EgtB was obtained from Protein Data Bank (PDB ID: 4X8B). To generate C–H fluorinase variants $EgtB_{CHF1}$ and $EgtB_{CHF2}$, appropriate residues were mutated using the Mutagenesis tool in PyMOL.[8] For variant $EgtB_{CHF1}$, mutated residues include Y377W W415R R87D, T141I, F83Y, R379E, V84R, H417L, and A145T. For variant $EgtB_{CHF2}$, mutated residues include Y377W, W415R, R87D, T141M, R379A, A145K, and Q422D (**Table 2** of the manuscript). The geometry of substrate *N*-fluoroamide **1** was optimized using the B3LYP functional[9] and 6-31G(d,p) basis set in *Gaussian 16*.[10] The optimized geometry of the substrate was then docked into $EgtB_{CHF1}$ and $EgtB_{CHF2}$ variants using AutoDock[11] with the Lamarckian genetic algorithm. A grid box with dimensions of 40 Å, 40 Å, and 40 Å was used, which center was set to be close to the Fe center. Docking parameters were set as follows: genetic algorithm run of 30, population size of 150, and 25 million energy evaluations. Because *N*-fluoroamide **1** may approach the Fe center at the coordination site *trans* to either residue H51 or H134, in our MD simulations, we considered these two binding modes **A** and **B**, where *N*-fluoroamide **1** is *trans* to H51 and H134, respectively (**Figure S10**). The best scored geometry for each binding mode from the docking calculations were then used to construct the initial input geometry for classical MD simulations. Because the potential substrate binding site *trans* to H138 is occupied by Q55 and W377 residues and our docking calculations did not locate docked substrate located proximal to that site, this binding mode was not considered in subsequent classical MD simulations.

Classical MD simulations were carried out using the pmemd module[12] of the GPU-accelerated Amber 16 software.[13] The Amber ff14SB force field[14] was used in all classical MD simulations. Force field parameters for the $Fe(II)(His)_3(H_2O)_2$(**1**) complexes in two binding modes were generated using the MCPB.py module[15] with the general Amber force field (gaff).[16] Using the

Merz-Singh-Kollman scheme,[17] RESP charge fitting[18] on electrostatic potential generated at the B3LYP/6-31G(d) level of theory was performed to generate partial charges at the high-spin quintet state, which was calculated to be the ground state of non-heme Fe(II) complexes.[19] Protonation states of enzyme residues were determined using the H++ server.[20] The enzyme was then put into a solvated cuboid box with the periodic boundary condition using the TIP3P water model.[21] The minimum distance between the enzyme surface and the edge of the water box was set to 10 Å. Water molecules were treated with the SHAKE algorithm.[22] The system was neutralized by adding Na^+ counterions. Long-range electrostatic was calculated using the particle-mesh-Ewald method.[23] Lennard-Jones and electrostatic interaction cut-offs were set to 12 Å.

We first performed energy minimization with positional restraints for the protein by applying a force constant of 500 kcal·mol^{-1}·Å^{-2} in 30,000 steps. Next, the system was gradually heated from 0 K to 300 K in 200 ps, which is followed by an equilibration using the isothermal–isobaric ensemble (NPT) in the next 1.5 ns. Finally, production MD simulations were run in 500 ns using the same conditions as the equilibration with a time step of 2 fs. In our MD simulations, to simulate the substrate near attack conformation in the fluorine atom transfer process and prevent undesired substrate dissociation events, the Fe–F distance was restrained in a range of 3.0 – 3.2 Å with a harmonic potential of 100 kcal·mol^{-1}·Å^{-2}. The restrained Fe–F distance was determined from DFT calculation with a small model, *i.e.*, an Fe(II)(Im)$_3$(H$_2$O)$_2$(**1**) complex, where the fluorine atom of substrate **1** form a weak dative bond with the Fe center with an Fe–F distance of 3.18 Å (**Figure S9**). After the MD simulations, clustering analysis was carried out using the cpptraj module[24] to identify the most populated structure in the last 400 ns of each MD simulation. The RMSD value was used as the distance metric for clustering analysis.

Our MD simulations demonstrate that in binding mode **A**, *N*-fluoroamide **1** forms hydrogen bonds with residue W415R in the simulations with both EgtB$_{CHF1}$ and EgtB$_{CHF2}$ variants. The N–H···O distance between the side chain of residue W415R and the amide carbonyl oxygen of **1** is less than 2.5 Å in most of the MD simulation times (61.2% and 81.9% for EgtB$_{CHF1}$ and EgtB$_{CHF2}$ variants, respectively; see **Figure S11**). On the other hand, in binding mode **B**, *N*-fluoroamide **1**

does not have any noticeable stabilizing interaction with residues in the active site. This result suggests that binding mode **B** is less favorable than binding mode **A**.

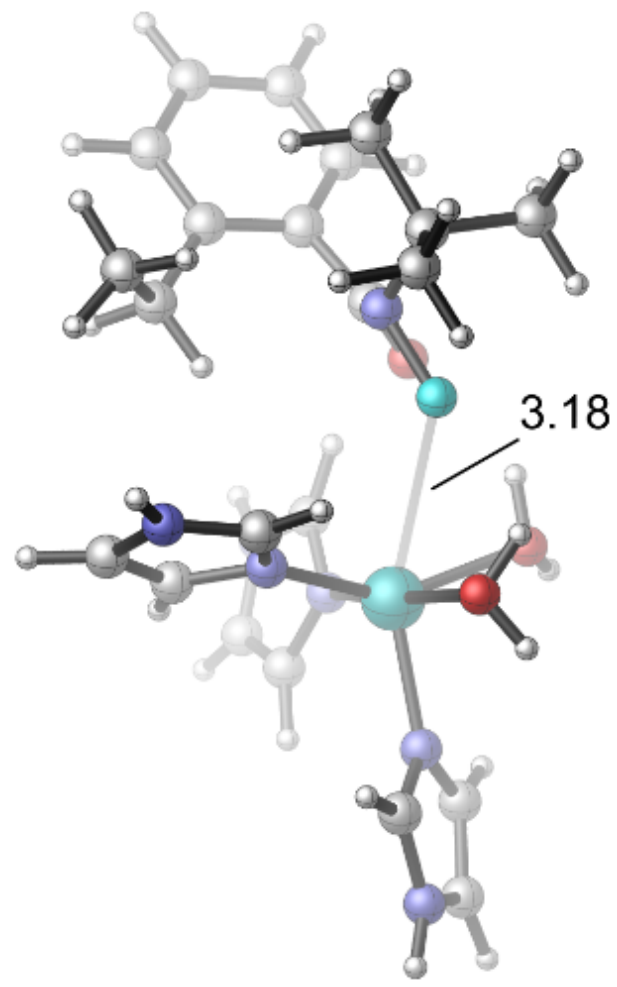

Figure S9. Optimized structure of the complex of *N*-fluoroamide (**1**) and a small model Fe(II)(Im)$_3$(H_2O)$_2$ where histidine residues are replaced by imidazole molecules at high-spin quintet state. The geometry optimization was carried out at the B3LYP-D3(BJ)/SDD(Fe)–6-31G(d) level of theory.

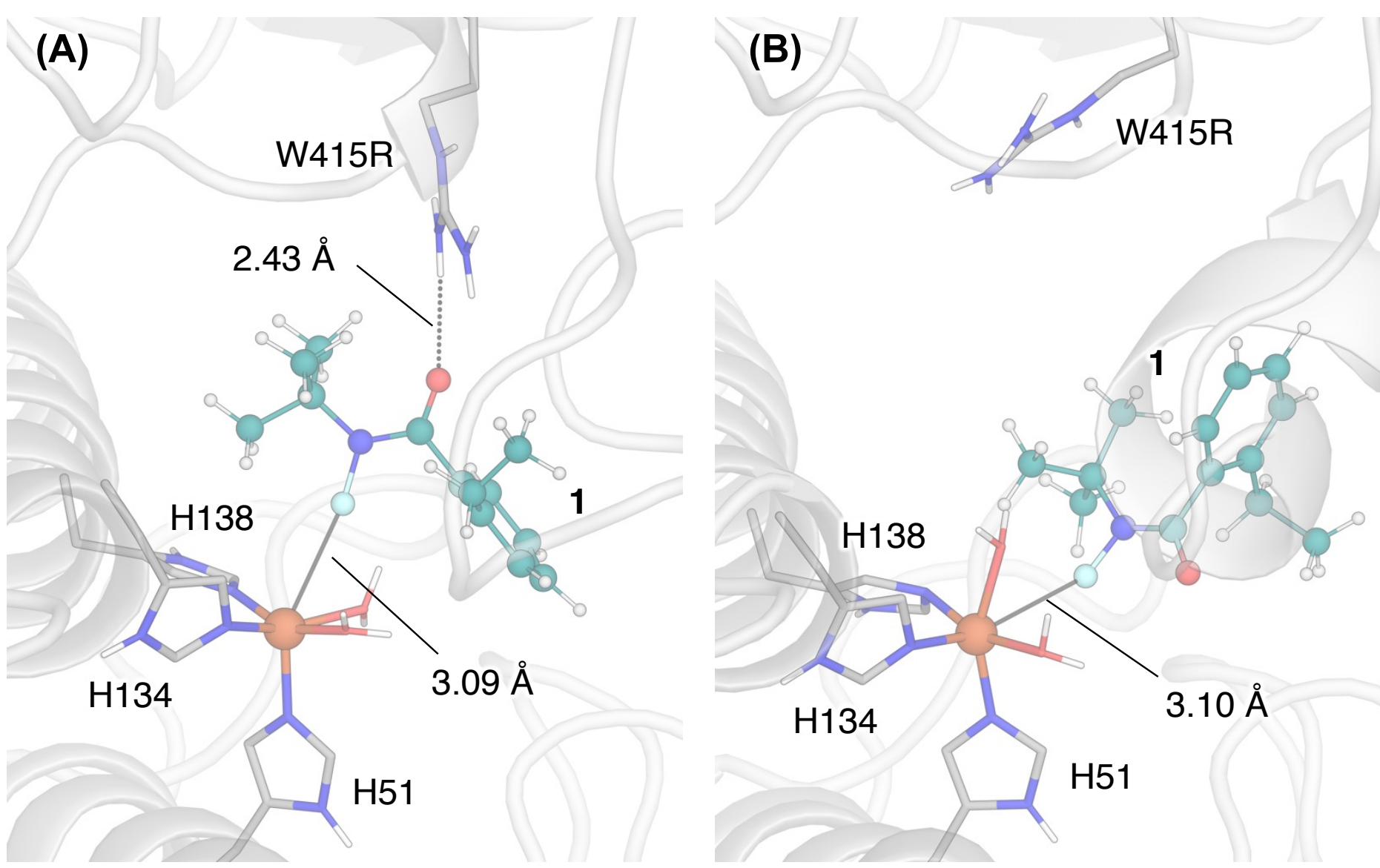

Binding mode **A** Binding mode **B**

Figure S10. The most populated structure of the EgtB$_{CHF1}$ variant in complex with *N*-fluoroamide **1** with in (**A**) binding mode **A** (**1** is *trans* to H51) and (**B**) binding mode **B** (**1** is *trans* to H134).

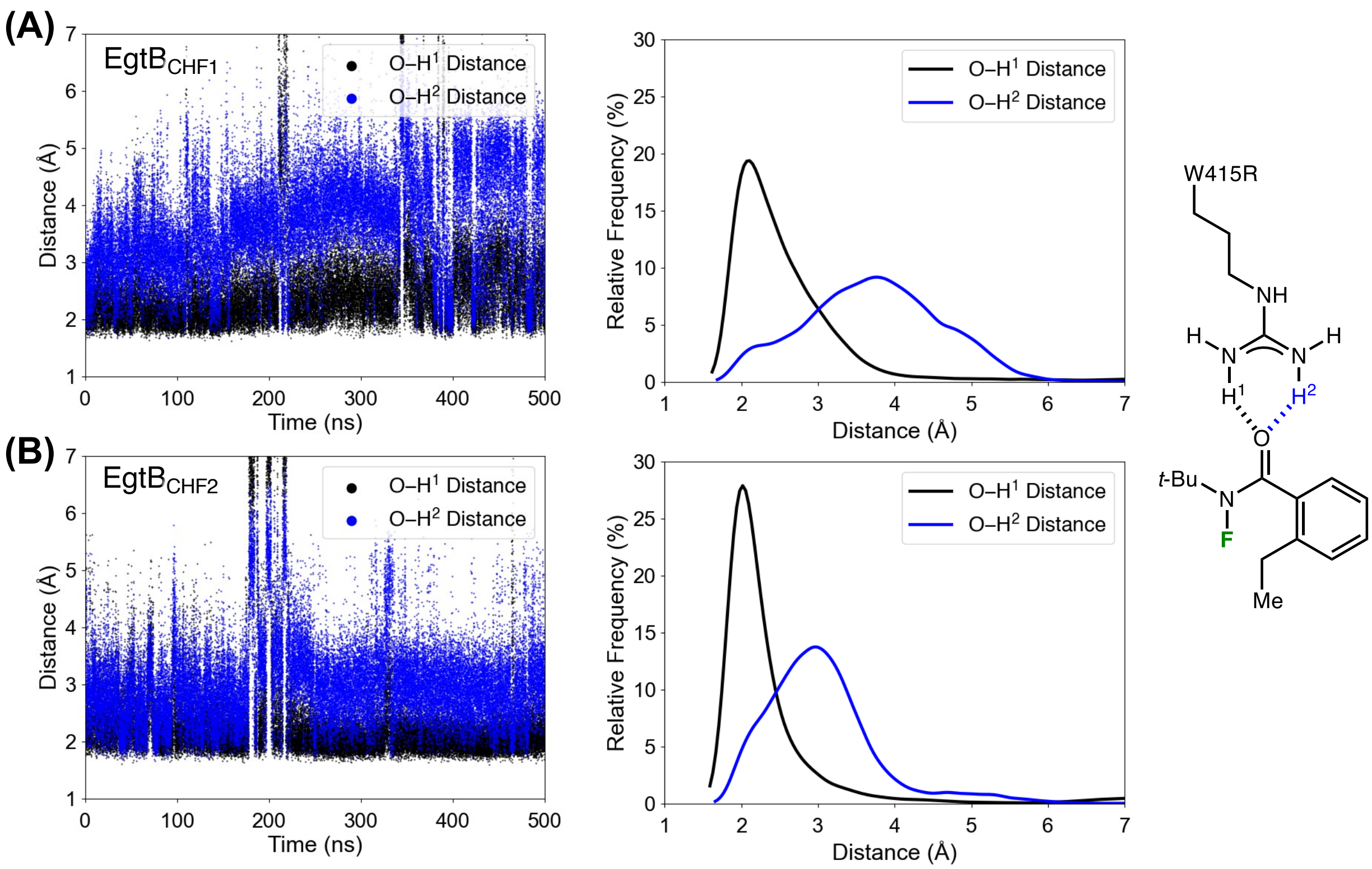

Figure S11. Time evolution and distribution of N–H···O distances between the side chain of residue W415R and the amide carbonyl oxygen of **1** in complex with (**A**) EgtB$_{CHF1}$ and (**B**) EgtB$_{CHF2}$ variants in binding mode **A** (**1** is *trans* to H51).

2. Arginine/π interaction between R175 and the phenyl group of the *N*-fluoroamide 1 in $ACCO_{CHF}$

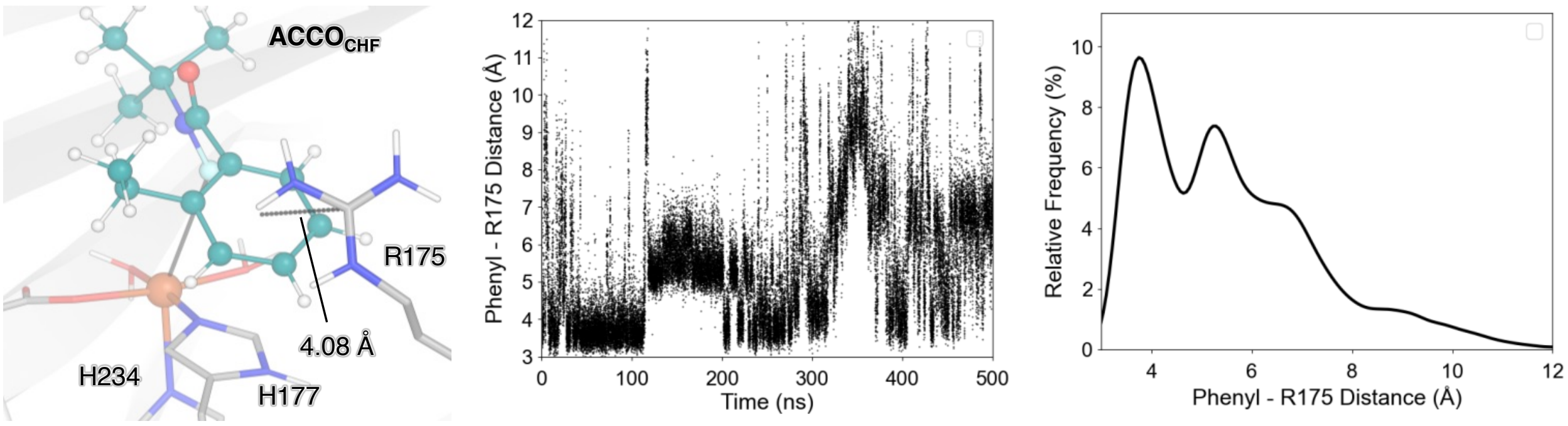

Figure S12. Time evolution and distribution of arginine/π interaction between R175 and the phenyl group of the *N*-fluoroamide **1** in $ACCO_{CHF}$.

3. Density Functional Theory Calculations

DFT calculations were performed using the Gaussian 16 software.[10] Geometries of intermediates and transition states were optimized using the dispersion-corrected[25] B3LYP-D3(BJ) functional[17c, 26] with the SDD basis set for Fe atom and 6-31G(d) basis set for other atoms. Vibrational frequency calculations were performed at the same level of theory as their respective geometry optimizations to confirm that the obtained transition states connect their respective minima. Quasi-harmonic approximation was performed with the Cramer-Truhlar approach[27] using the GoodVibes package[28], in which all vibrational frequencies below 100 cm^{-1} were shifted to 100 cm^{-1} in entropy calculations. Single-point energy calculations were carried out using the B3LYP-D3(BJ) functional with the def2-TZVP basis set.[29] Solvation energy corrections were calculated in the single-point energy calculations using the SMD solvation model.[30] Diethyl ether solvent (ε = 4.3) was used to mimic the actual dielectric constant within enzyme active site.[31] High and intermediate spin states for all species were calculated (*i.e.*, quintet and triplet states for Fe(II) species and sextet and quartet states for Fe(III) species).

Local force constants of Fe–F bond in all $Fe^{III}F$ species were calculated using the local modes method[32] at B3LYP-D3(BJ)/6-31G(d)–SDD(Fe) level of theory (i.e., optimization level).

4. QM/MM Calculations

The ONIOM algorithm[33] implemented in Gaussian 16 was used in QM/MM (hybrid quantum mechanics/classical mechanics) geometry optimizations of intermediates and transition states. The entire enzyme, substrate, water molecules, and counter ions within 5 Å from the enzyme were included in the QM/MM calculations. The QM region includes the Fe atom, H51, H134, H138, W415R residues, and the substrate **1**, which has 107 atoms and a total charge of +1. Only high-spin state was calculated because our DFT calculations (see later) indicated this state is the ground states of intermediates and TSs.

For the QM region, the B3LYP-D3(BJ)/6-31G(d)–SDD(Fe) level of theory was used in geometry optimization and vibrational frequency calculations, and the B3LYP-D3(BJ)/def2-TZVP level of theory was used in single-point energy calculations. For the MM region, the same force field parameters from the classical MD simulations were used. Residues, water molecules, and Na^+ counterions greater than 10 Å away from the QM region were fixed during geometry optimization. The quadratic coupled algorithm[34] and the mechanical embedding scheme were used in geometry optimization. Single-point energy calculations were performed with the electronic embedding scheme, which better describes electrostatic interactions between QM and MM regions.[35]

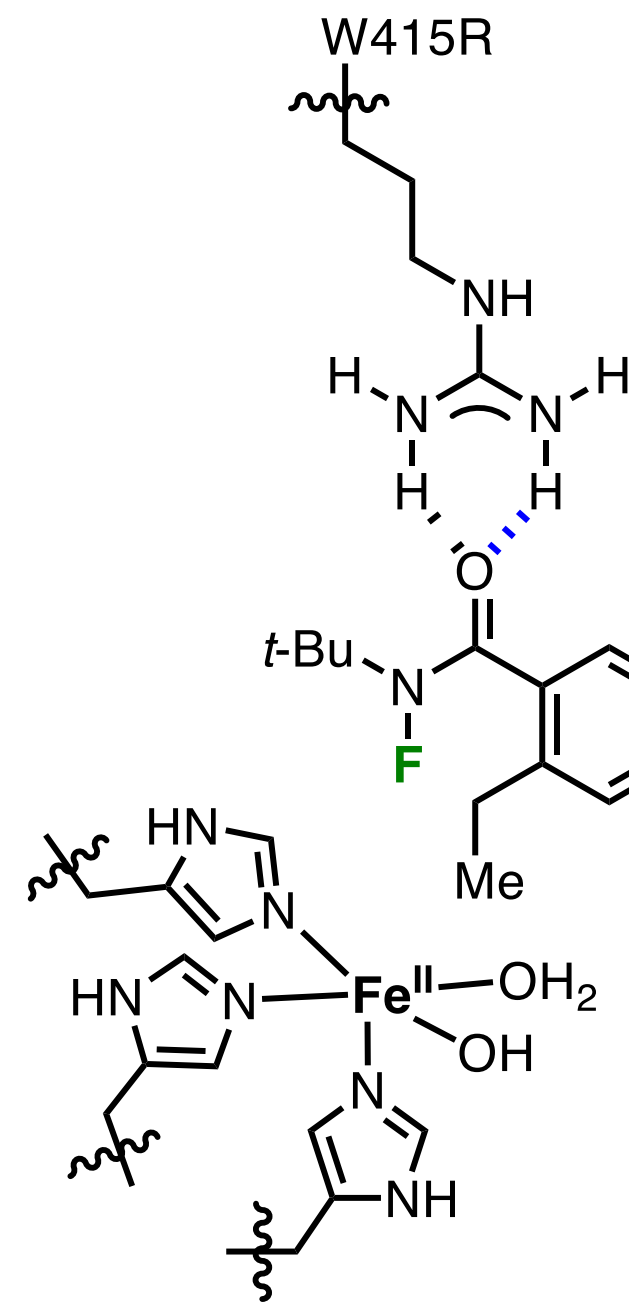

Scheme S1. QM region used in QM/MM Calculations. Wavy lines represent QM-MM boundary.

5. Bond Dissociation Enthalpy Calculations and Effect of Hydrogen Bond on N–F Bond

Bond dissociation enthalpies (BDEs) of N–F, Fe–F, and C–F bonds were calculated at the B3LYP-D3(BJ)/def2-TZVP/SMD(Et_2O)//B3LYP-D3(BJ)/6-31G(d)–SDD(Fe) level of theory (**Figure S13**).

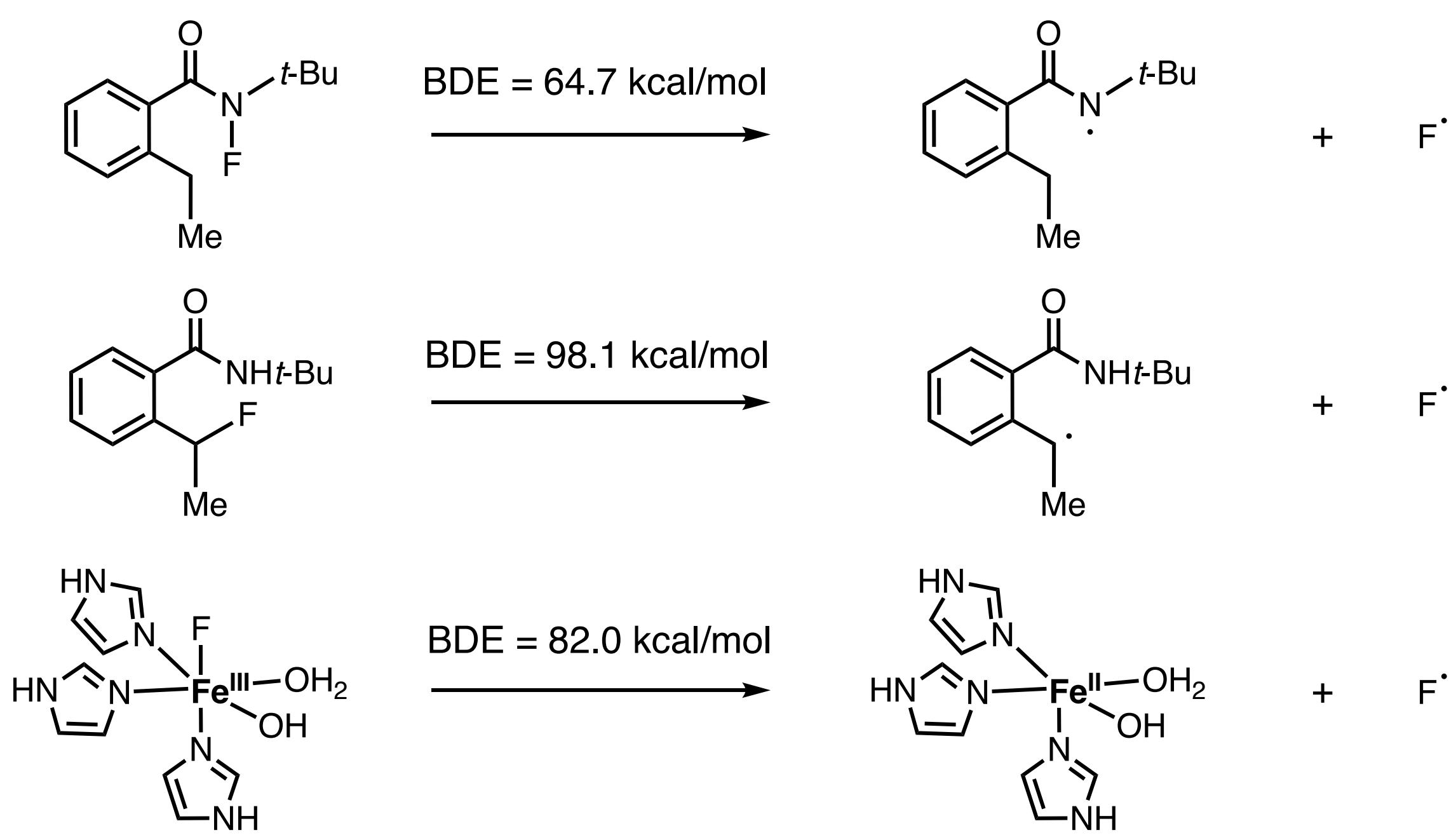

Figure S13. Bond dissociation enthalpy (BDE) calculations. Fe(II) and Fe(III)–F intermediates were calculated at high-spin quintet and sextet states, respectively.

Our calculations show that the N–F BDE decreases to 62.9 kcal/mol in the hydrogen bond complex with guanidium, compared to the N–F BDE of 64.7 kcal/mol in *N*-fluoroamide **1** itself (**Figure S14**). On the other hand, the N–F BDE in a hydrogen bond complex with a water molecule is 64.1 kcal/mol, which is only 0.6 kcal/mol weaker than the N–F BDE of **1**. These results indicate that the activation effect of hydrogen bond with the guanidium group in the W415R side chain is stronger than the hydrogen bond with water.

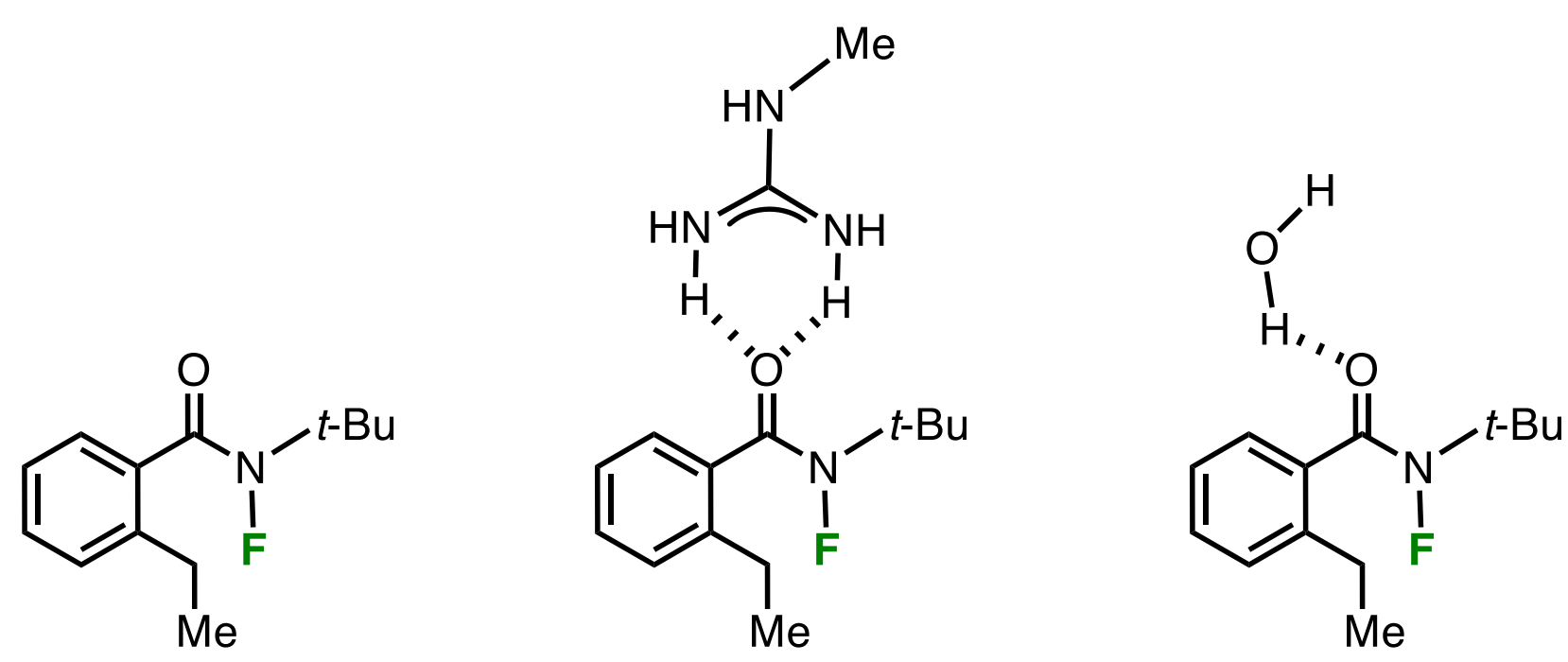

Figure S14. Effect of hydrogen bonds on N–F BDEs.

6. Protonation State of Water Molecules Bound to the Fe Center

Because the protonation states of water molecules bound to the Fe center are unknown, we calculated the pK_a values of several water-coordinated $(Im)_3$Fe(II) and $(Im)_3$Fe(III)–F species (Im = imidazole). We used the SMD_{sSAS} approach described by Smith *et al*.[36] to calculate the *p*Ka values in aqueous solution (**Scheme S2**). We used the same scaling factor ($\alpha = 0.485$) proposed by Smith to generate the solute cavity. In our own benchmark studies[37] using hexaaqua Fe(III) complex as a test compound, a better agreement with experimental *p*Ka value was obtained with the SMD_{sSAS} approach compared with the calculations using default SMD parameters.

We computed Gibbs free energies of the protonated form (*i.e.*, two water molecule bound) of the Fe(II) and Fe(III)–F species ($G^*_{\mathrm{aq}}(\mathrm{AH^+})$) and the corresponding conjugate bases ($G^*_{\mathrm{aq}}(\mathrm{A})$) using DFT at the B3LYP-D3(BJ)/def2-TZVP/SMD_{sSAS}(H_2O)//B3LYP-D3(BJ)/6-31G(d)–SDD(Fe) level of theory. The Gibbs free energy of a proton in aqueous solution ($G^*_{\mathrm{aq}}(\mathrm{H^+})$) was calculated from the gas-phase free energy of proton ($G^{\mathrm{o}}_{\mathrm{g}}(\mathrm{H^+}) = -6.28\ \mathrm{kcal/mol}$)[38] and its hydration free energy ($\Delta G_{\mathrm{aq,solv}}(\mathrm{H^+}) = -265.9\ \mathrm{kcal/mol}$) from literature.[39] $\Delta G^{\mathrm{o}\rightarrow *} = RT\ln(24.47) = 1.89\ \mathrm{kcal/mol}$ is the free energy change to convert from the standard state of ideal gas (1 atm) to the standard state in solution (1 M).[30]

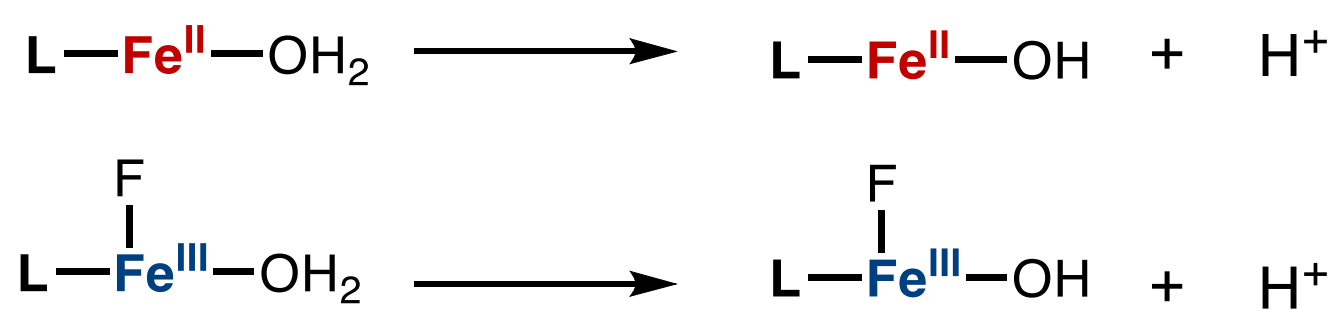

$$\mathrm{AH^+(aq)} \xrightarrow{\Delta G^*_{\mathrm{aq}}} \mathrm{A(aq)} + \mathrm{H^+(aq)} \quad \textbf{(S1)}$$

$$\mathrm{p}K_{\mathrm{a}} = \frac{\Delta G^*_{\mathrm{aq}}}{2.303RT} \quad \textbf{(S2)}$$

$$\Delta G^*_{\mathrm{aq}} = G^*_{\mathrm{aq}}(\mathrm{A}) + G^*_{\mathrm{aq}}(\mathrm{H^+}) - G^*_{\mathrm{aq}}(\mathrm{AH^+}) \quad \textbf{(S3)}$$

$$G^*_{\mathrm{aq}}(\mathrm{H^+}) = G^{\mathrm{o}}_{\mathrm{g}}(\mathrm{H^+}) + \Delta G_{\mathrm{aq,solv}}(\mathrm{H^+}) + \Delta G^{\mathrm{o}\rightarrow *} \quad \textbf{(S4)}$$

Scheme S2. Acid dissociation constant (*p*Ka) calculations.

The computed aqueous *p*Ka values indicate that the *p*Ka of water significantly decreases when bound to either an Fe(II) or Fe(III) center. H_2O bound to Fe(III) shows a lower *p*Ka relative to that bound to Fe(II). It should be noted that the enzyme active-sit environment further modulates the *p*Ka of Fe-bound water molecules. Therefore, these computational results alone cannot conclusively identify the protonation state of the Fe(II) species although Fe(III)–F compounds in $EgtB_{CHF}$ (three-histidine facial triad and two-histidine facial dyad) are likely deprotonated under the experimental conditions considering their low computed *p*Ka values.

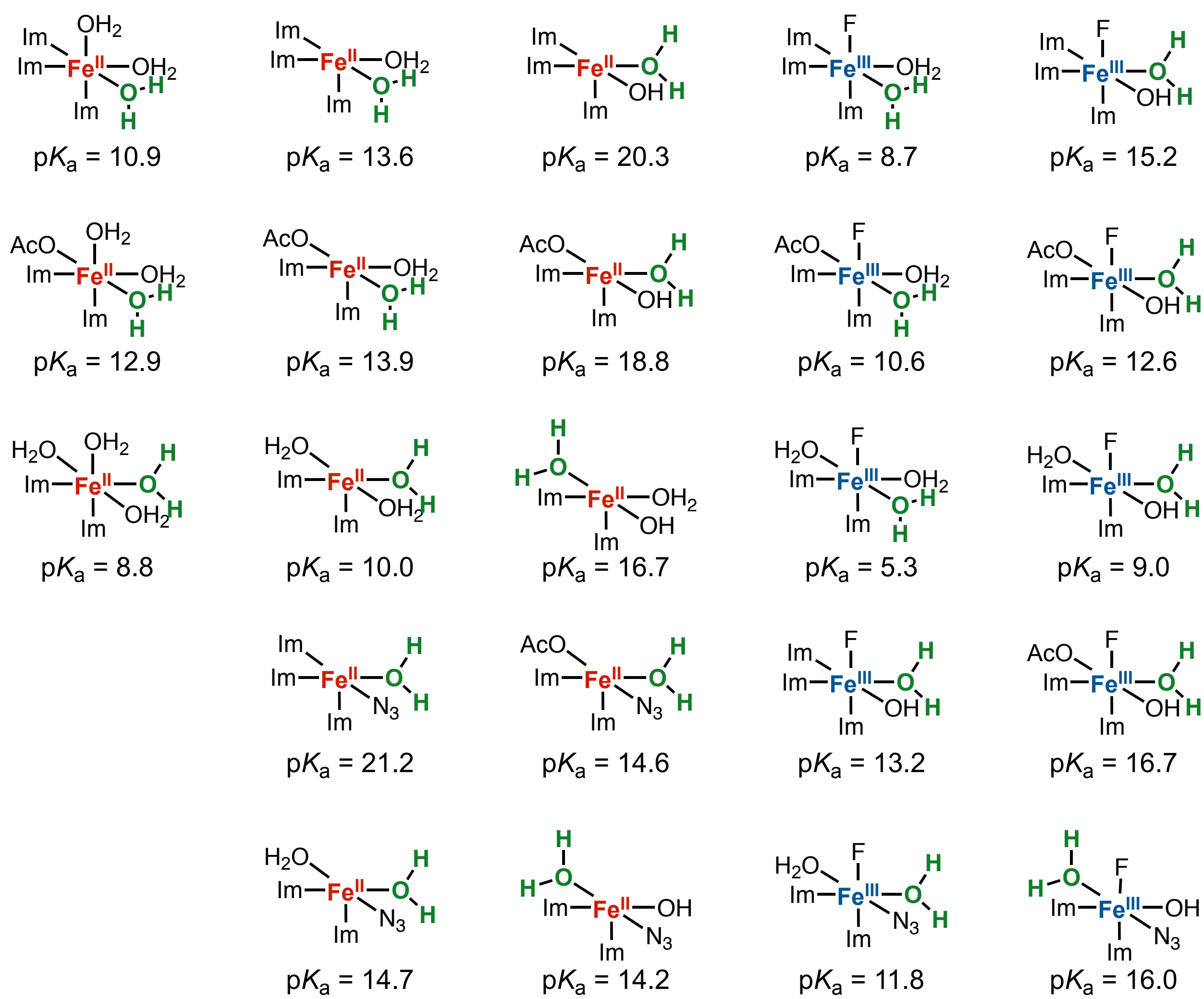

Figure S15. DFT-computed *p*Ka values of water-coordinated Fe(II) and Fe(III)–F compounds (the specific H_2O studied is highlighted in green). Fe(II) and Fe(III)–F intermediates were calculated at high-spin quintet and sextet states, respectively.

Considering that the enzyme environment can substantially modulate *p*Ka values through hydrogen-bonding interactions, active-site water organization, and substrate-induced effects, *p*Ka calculations were performed using theozyme models. The initial geometries of all theozyme models were extracted from the most populated structures obtained from classical MD simulations. Our theozyme model for $EgtB_{CHF1}$ enzyme consists of *N*-fluoroamide **1**, the Fe atom, two water molecules bound to the Fe atom, and five active site residues represented by their side chains and

Cα atoms: H51, H134, and H138, which coordinate the Fe center; W415R, which forms a hydrogen-bonding interaction with substrate **1**; and Q137, which is in close contact with the substrate. In addition, two second-shell water molecules that form hydrogen bond to the Fe-bound water ligands were included.

In addition, the theozyme model for $ACCO_{CHF}$ enzyme consists of *N*-fluoroamide **1**, the Fe atom, two water molecules bound to the Fe atom, and five active site residues represented by their side chains and Cα atoms: D179, H177, and H234, which coordinate the Fe center; R175, which forms a cation–π interaction with substrate **1**; N216, which hydrogen bonds with D179 and the Fe-bound water molecules; and T178, which is located between D179 and H177 in the active site.. Three additional second-shell water molecules that participate in hydrogen-bonding interactions with substrate **1**, the Fe-bound water ligands, and D179 were also included. Main chain carbonyl and amino groups of these residues were replaced with C–H bonds. To maintain the locations of amino acid residues as in the most populated structure of the enzyme, the positions of Cα and added H atoms of all residues included in the theozyme were fixed during geometry optimizations. DFT calculations were performed at the B3LYP-D3(BJ)/def2-TZVP/SMD_{sSAS}(H_2O)//B3LYP-D3(BJ)/6-31G(d)–SDD(Fe) level of theory. Similar active site cluster/theozyme approaches have previously been applied to investigate protonation equilibria and pKa shifts in enzyme active sites, including carbonic anhydrase[40] and papain.[41]

Interestingly, the *p*Ka values obtained from the theozyme calculations give a consistent trend with those derived from the truncated models. In both models, the water molecule bound to Fe(III) is predicted to be substantially more acidic than that bound to Fe(II). Notably, the enzyme active-site environment further modulates the acidity of the Fe-bound water ligands through local electrostatic and hydrogen-bonding interactions. Taken together, these results suggest that the Fe(III)–F intermediates in $EgtB_{CHF}$ (three-histidine facial triad and two-histidine facial dyad) are predominantly present in their deprotonated forms under the experimental conditions.

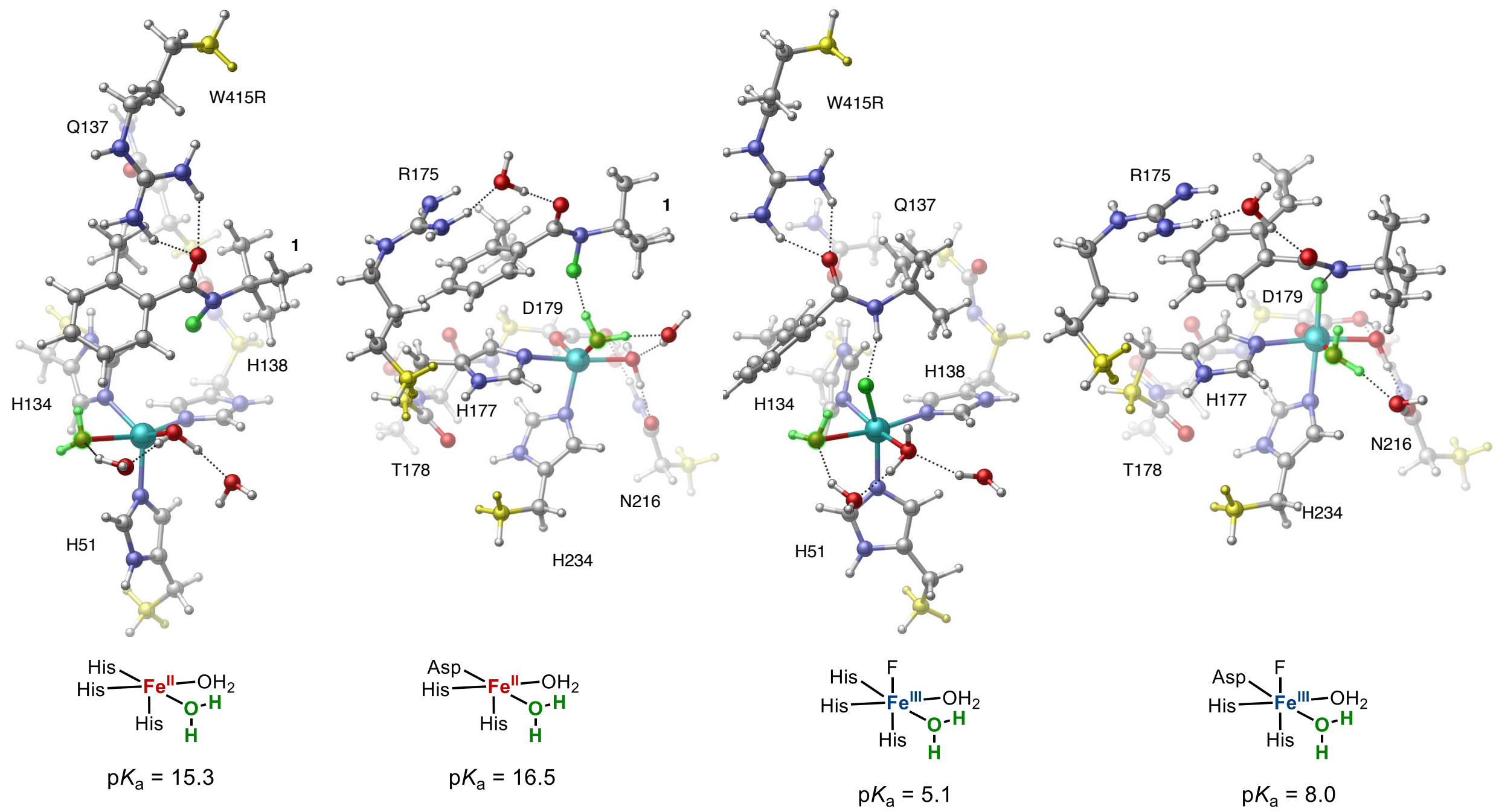

Figure S16. *p*Ka calculation using theozyme models. The specific H_2O studied is highlighted in green. Positions of the Cα and added H atoms (colored in yellow) of active site residues were constrained during geometry optimizations. DFT calculations were performed at high-spin quintet state.

7. Protonation Abstraction from Fe(III)–F Species

The proton abstraction step from Fe(III)–F species by acetate as a base was compute at the B3LYP-D3(BJ)/def2-TZVP/SMD$_{sSAS}$(H_2O)//B3LYP-D3(BJ)/6-31G(d)–SDD(Fe) level of theory.

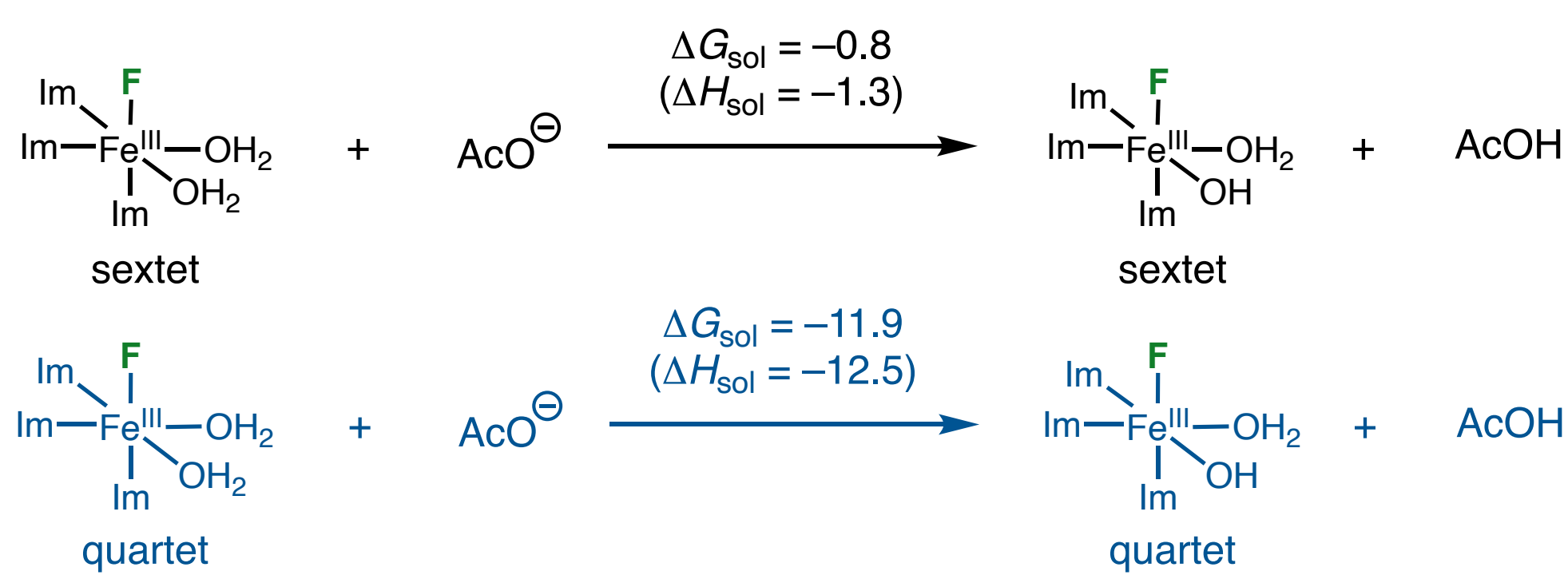

Figure S17. Proton abstraction from Fe(III)–F species. Fe(II) and Fe(III)–F intermediates were calculated at high-spin quintet and sextet states, respectively.

To identify potential proton acceptors involved in deprotonation of the Fe-bound water molecule, we further analyzed the classical MD simulation trajectories. In the most populated conformations of both $EgtB_{CHF1}$ and $EgtB_{CHF2}$ enzymes, we found that the D81 and D87 residues are located proximity to the non-heme iron center. The carboxylate groups of these residues are within ~7 Å of the Fe center and are connected to the active site through a network of water molecules. Such an arrangement could enable proton transfer from the Fe-bound water molecule through a water-wire network, implicating D81 and D87 as potential proton acceptors. (**Figure S18A**).

To further evaluate the feasibility of this process, we performed DFT calculations using a theozyme model that explicitly includes the Fe-bound water molecule, the bridging water molecules, and the D81 and D87 proton acceptors. The calculated activation enthalpies (**Figure S18B**) indicates that proton transfer through the proposed water-wire pathway is kinetically facile, with a sufficiently low barrier to compete with the subsequent 1,5-hydrogen atom transfer step. These results support the feasibility of Fe(III)–OH species formation prior to radical rebound, providing a mechanistic rationale for the experimentally observed hydroxylation pathway.

Taken together, the observed water organization provides a structural basis for proton transfer from the Fe-bound water molecule to D81/D87 through the hydrogen-bonded water molecules. Combined with the DFT calculations showing kinetically feasible proton transfer, these results support formation of the Fe(III)–OH species under the experimental conditions, consistent with the observed pH dependence of hydroxylation.

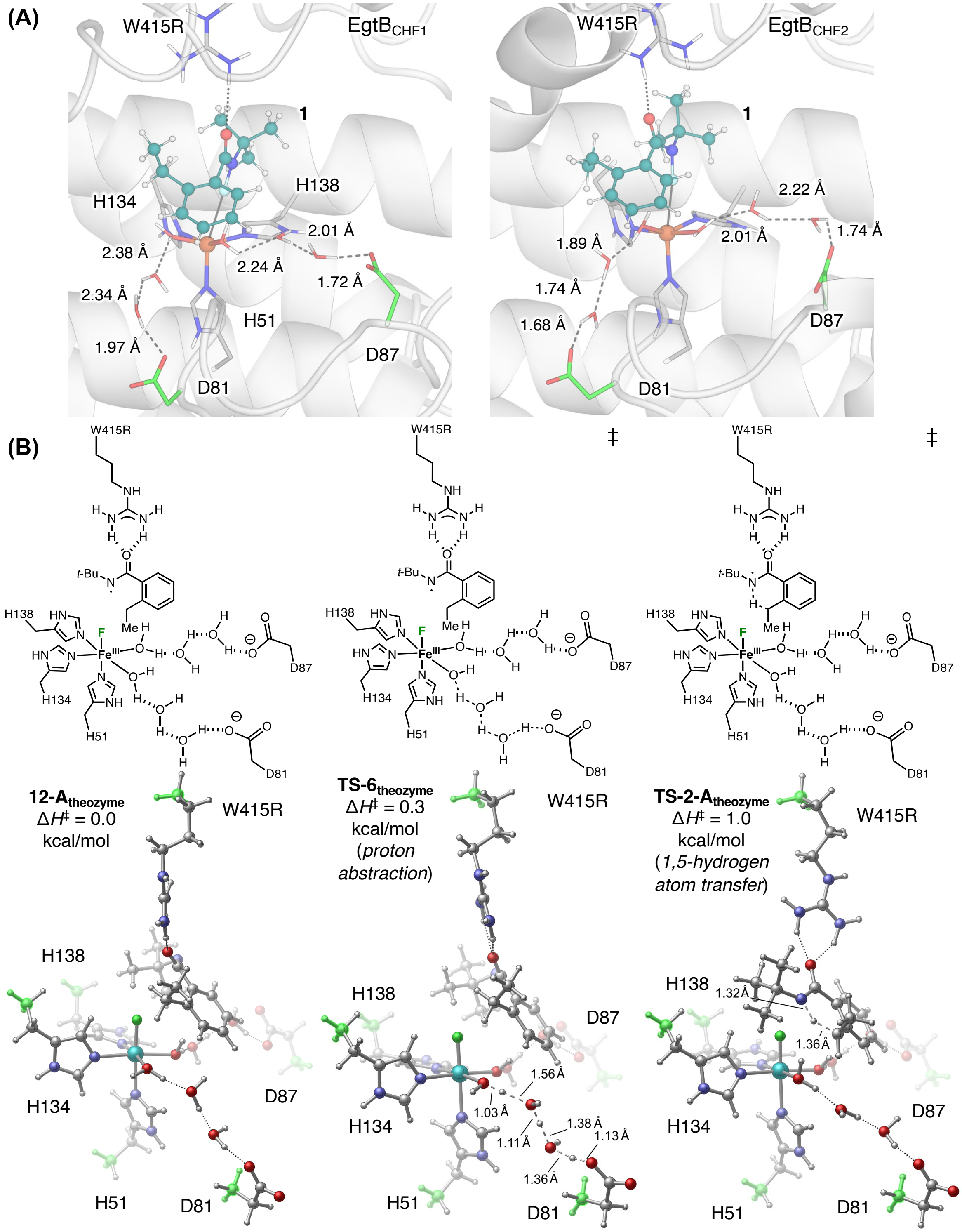

Figure S18. Computational studies for the protonation abstraction from Fe(III)–F species. (**A**) The most populated structure of the EgtB$_{CHF1}$ and EgtB$_{CHF2}$ variants obtained from classical MD simulations. Residues D81 and D87 are proximal to the non-heme iron center and connected to the

active site through water molecules, suggesting D81 and D87 as potential proton acceptors for the water deprotonation. (**B**) DFT calculations using theozyme model. Positions of the Cα and added H atoms (highlighted in green) of active site residues were constrained during geometry optimizations. DFT calculations were performed at high-spin quintet state. Enthalpies are relative to **12-A**$_{\textbf{theozyme}}$.

8. Reaction Energy Profile of Nonheme Fe Enzyme-Catalyzed C–H Fluorination

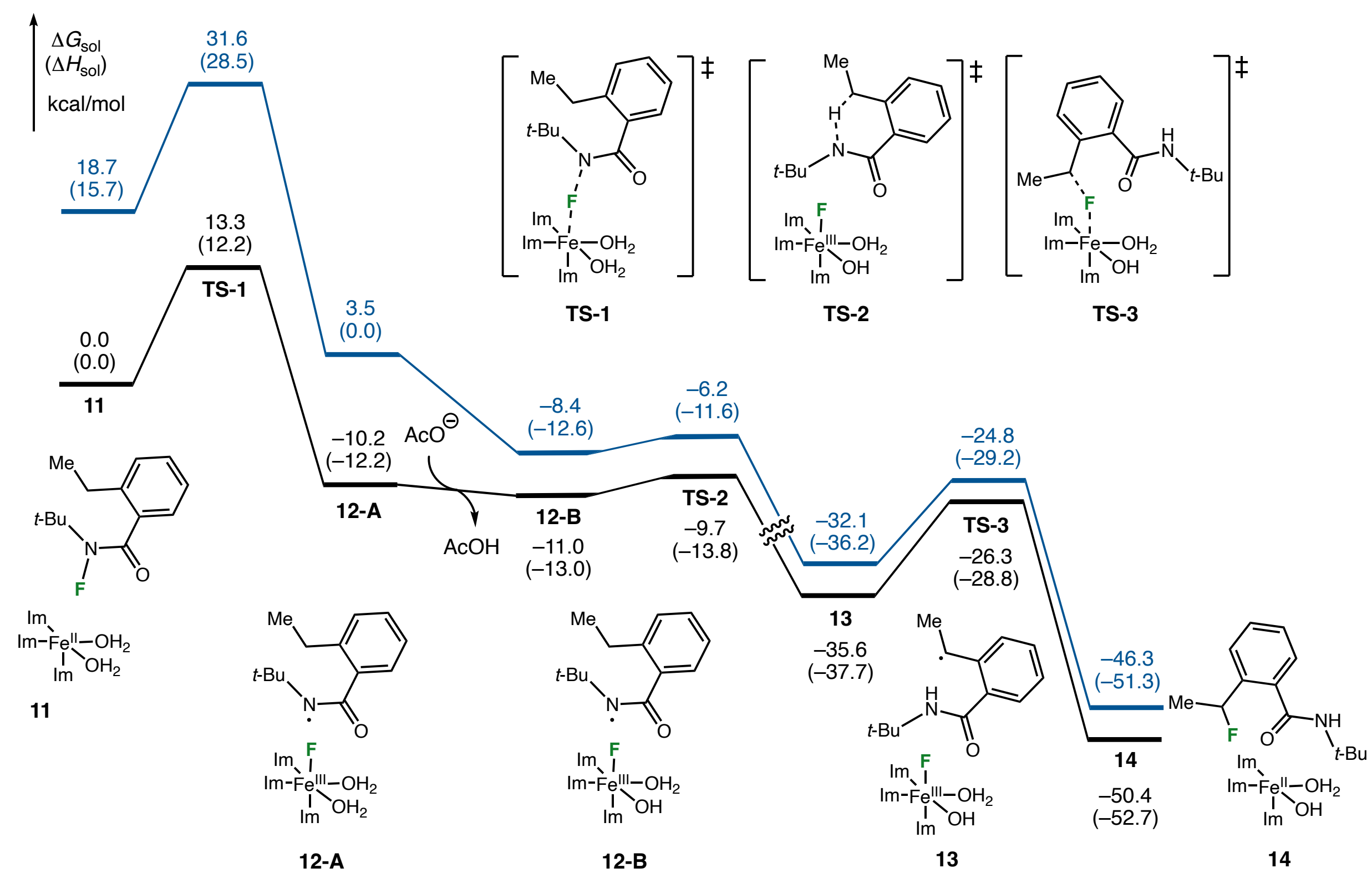

Figure S19. Reaction energy profile of the C(sp^3)–H fluorination at quintet (black) and triplet (dark blue) spin states computed using the truncated model at the B3LYP-D3(BJ)/def2-TZVP/SMD(Et_2O)//B3LYP-D3(BJ)/6-31G(d)–SDD(Fe) level of theory.

9. Correlation Between Activation Barrier and Other Parameters for Radical Rebound

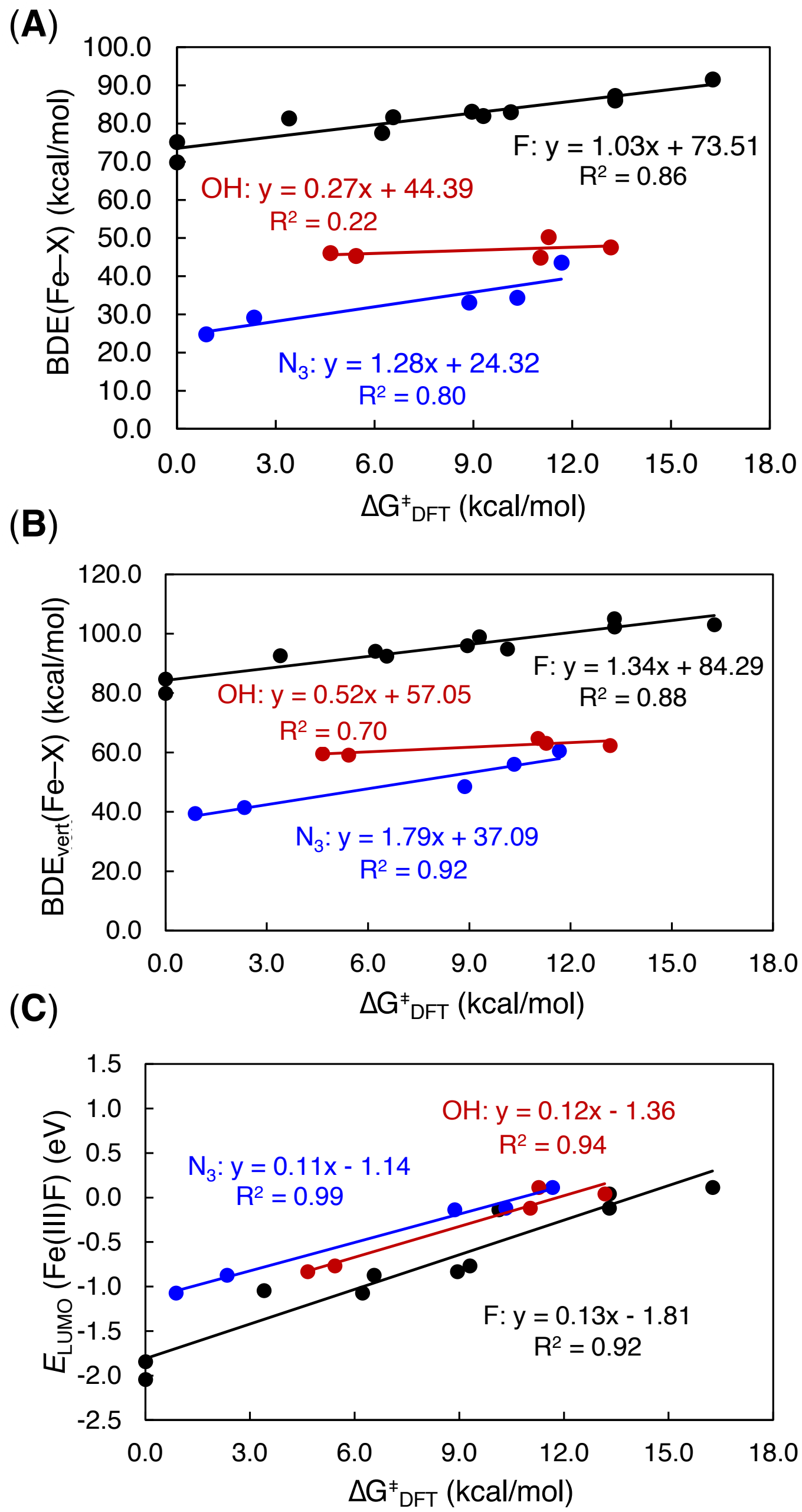

Figure S20. Correlation between activation barriers ($\Delta G^{\ddagger}$) for the radical rebound step with (**A**) bond dissociation enthalpies (BDE(Fe–X), X = F, N_3 or OH) (**B**) vertical bond dissociation energies (BDE_{vert}(Fe–X)), and (**C**) LUMO energies of $Fe^{III}F$ species ($E_{LUMO}(Fe^{III}F)$). The black, blue, and red colors represent the fluorine, azide, and hydroxyl radical rebound reactions, respectively.

10. Alternative Regression Model for Fluorine Atom Abstraction

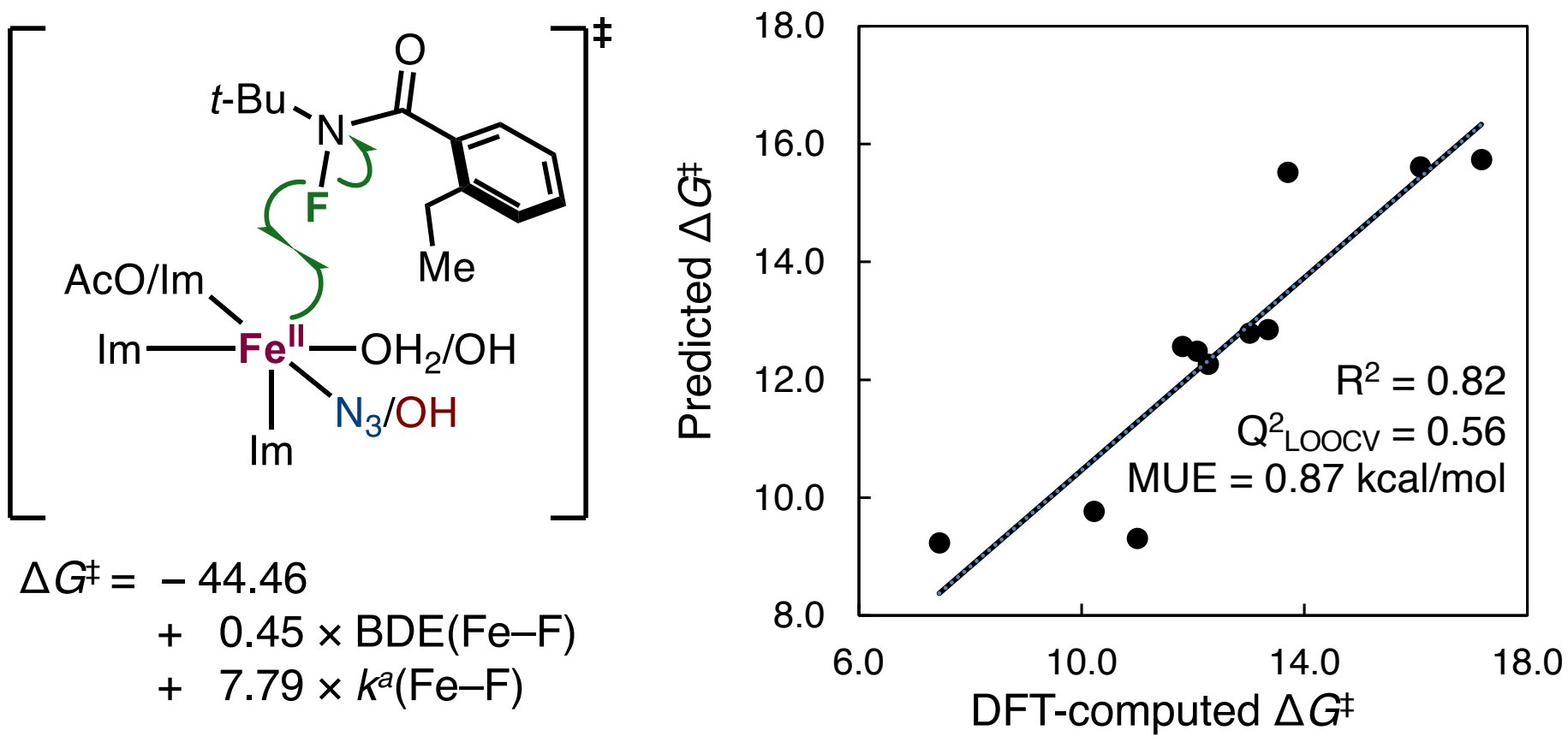

Figure S21. Alternative regression model for the fluorine atom abstraction step. BDE(Fe–F) is the bond dissociation enthalpy of Fe–F bond.

11. Alternative Regression Model for Radical Rebound

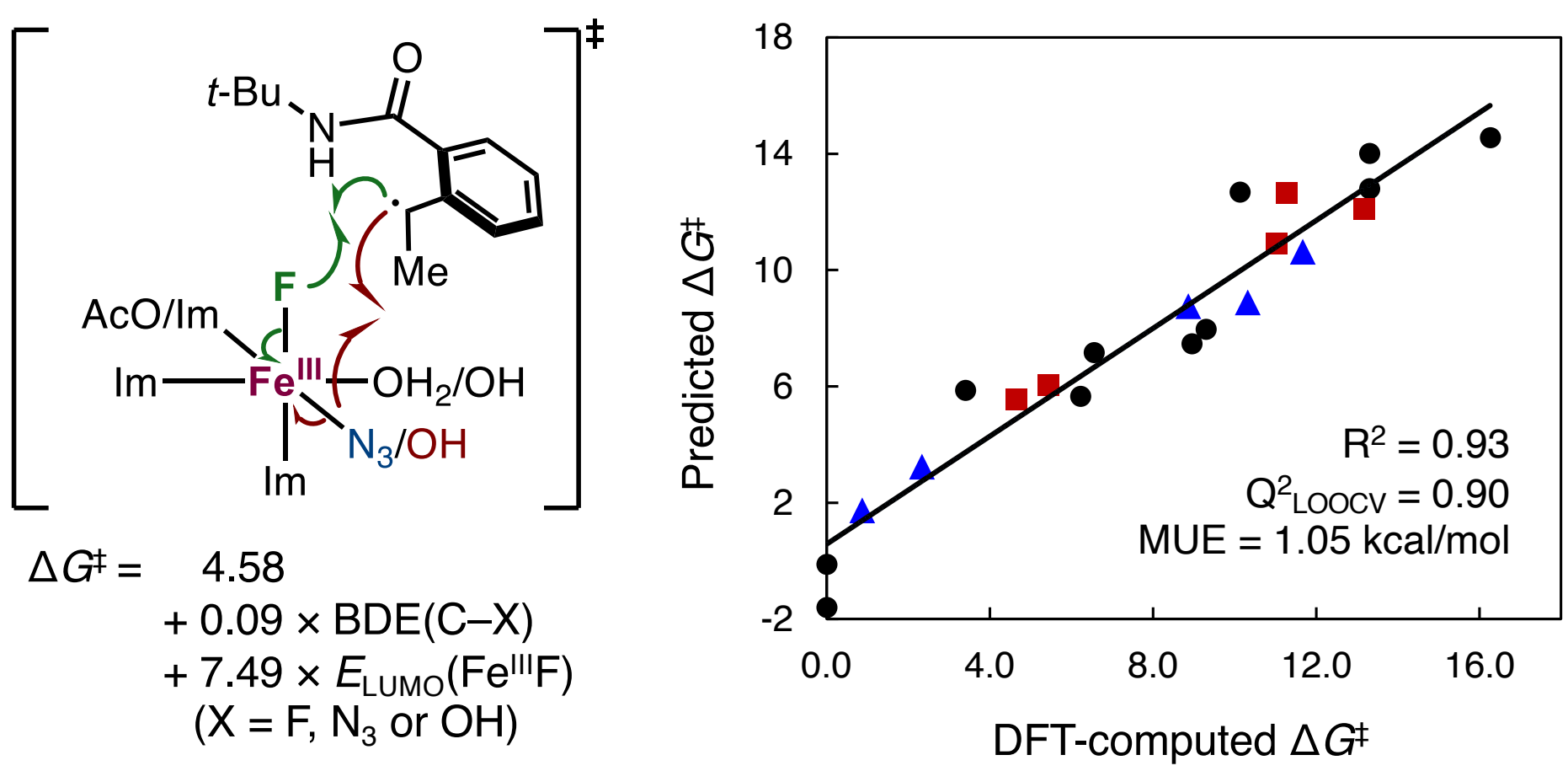

Figure S22.. Predictive models for the radical rebound step using the bond dissociation enthalpy of C–X bond, BDE(C–X), and LUMO energy of $Fe^{III}F$ species, $E_{LUMO}(Fe^{III}F)$, as descriptors.

12. Substrate Reduction by Fe(II) Species

The oxidation potentials of *N*-fluoroamide substrate **1** and Fe(II) intermediates were calculated from the reaction Gibbs free energies of the half-reactions at the B3LYP-D3(BJ)/def2-

TZVP/SMD(Et_2O)//B3LYP-D3(BJ)/6-31G(d)–SDD(Fe) level of theory. The Gibbs free energy of electron of −0.867 kcal/mol.[42]

Since

$$\Delta G_{rxn} = -n \times F \times E_{red}^{abs}$$

where *n* is the number of electron transferred (one), *F* is the Faraday constant. The absolute standard reduction potential can be calculated as

$$E_{red}^{abs} = \frac{\Delta G_{rxn}}{-n \times F}$$

Based on our DFT calculation, the electron transfers from Fe(II) to *N*-fluoroamide substrate **1** are highly endergonic (101.8 and 71.5 kcal/mol for $(Im)_3Fe^{II}(H_2O)_2$ (**11**) and $(Im)_2(AcO)Fe^{II}(H_2O)_2$ (**11'**), respectively). The highly unfavorable thermodynamics thus rule out the single electron transfer (SET) pathway.

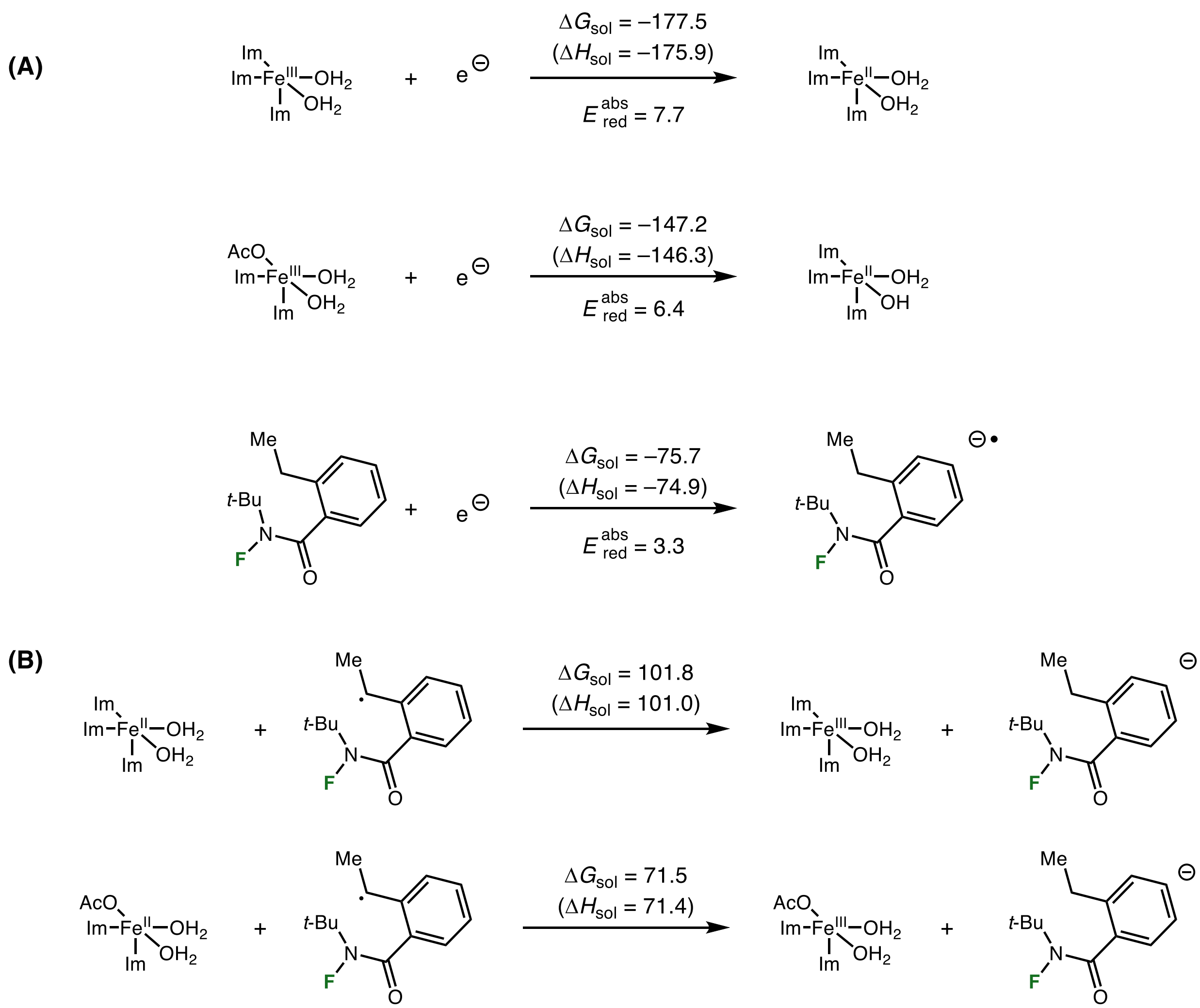

Figure S23. (**A**) Absolute standard reduction potential for half-reaction. (**B**) Electron transfer processes from Fe(II) to *N*-fluoroamide substrate **1**. Fe(II) and Fe(III)–F intermediates were calculated at high-spin quintet and sextet states, respectively.

13. Benzyl Radical Oxidation Pathway by Fe(III)–F Species

We further evaluated the oxidation potentials of the benzyl radical and Fe(III)–F intermediates at the B3LYP-D3(BJ)/def2-TZVP/SMD(Et_2O)//B3LYP-D3(BJ)/6-31G(d)–SDD(Fe) level of theory. Electron transfer from the benzyl radical to the Fe(III)–F intermediate is highly unfavorable, being uphill by 23.1 and 33.9 kcal/mol for $(Im)_3Fe^{III}F(H_2O)_2$ (**13**) and

$(Im)_2(AcO)Fe^{III}F(H_2O)_2$ (**13’**), respectively. Therefore, the formation of a carbocation intermediate via a single electron transfer mechanism can be ruled out.

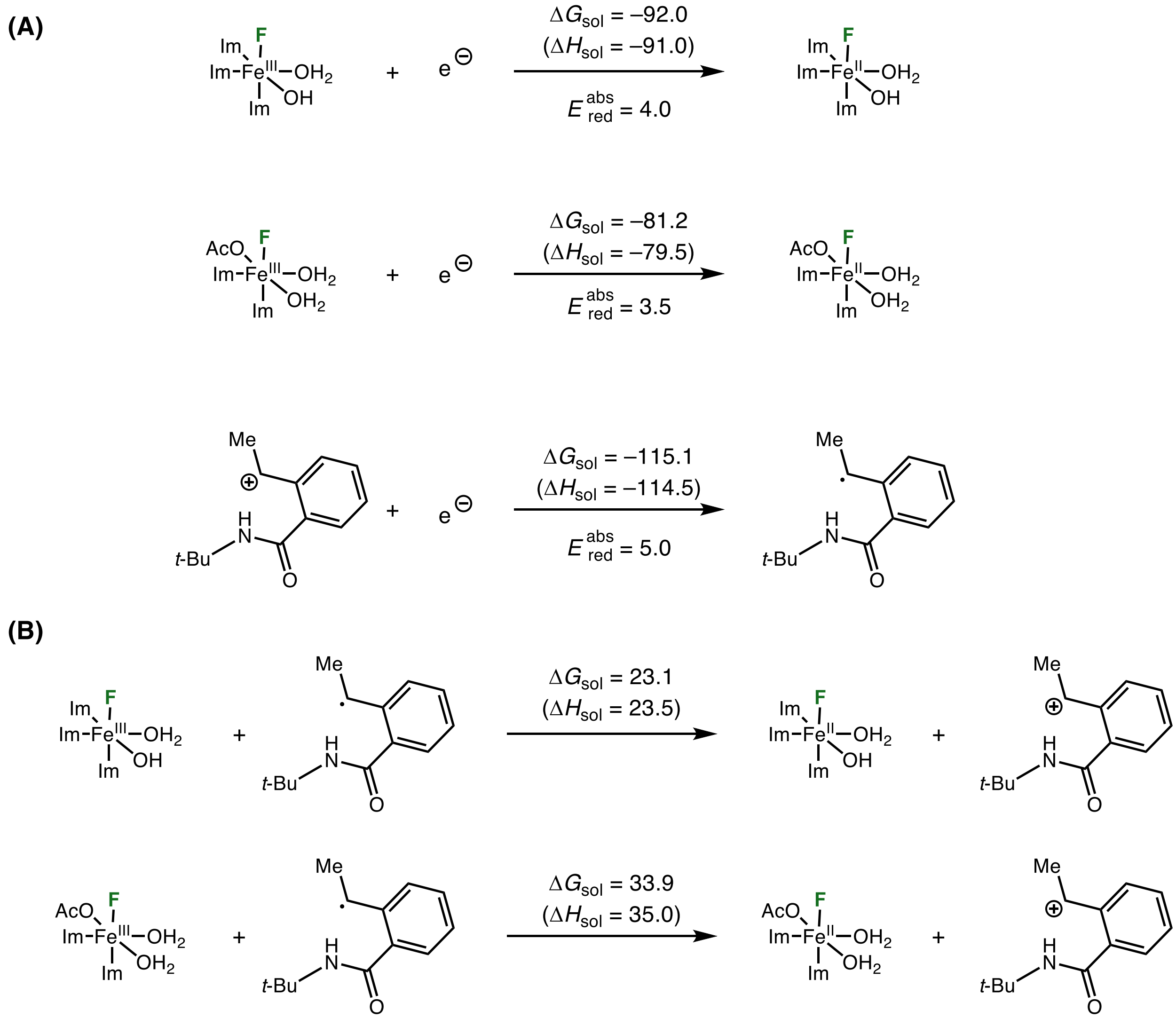

Figure S24. (**A**) Absolute standard reduction potential for half-reaction. (**B**) The formation of a carbocation intermediate via a single electron transfer mechanism. Fe(II) and Fe(III)–F intermediates were calculated at high-spin quintet and sextet states, respectively.

14. Binding Free Energies of Azide Anion to Fe(II) and Fe(III)–F Species

The free energies associated with azide binding to Fe(II) and Fe(III)–F species were calculated at the B3LYP-D3(BJ)/def2-TZVP/SMD(Et_2O)//B3LYP-D3(BJ)/6-31G(d)–SDD(Fe) level of theory. The calculated binding processes are uniformly exergonic ($\Delta G = -13.2$ to -62.6 kcal/mol), indicating a strong thermodynamic preference for azide coordination to the iron center.

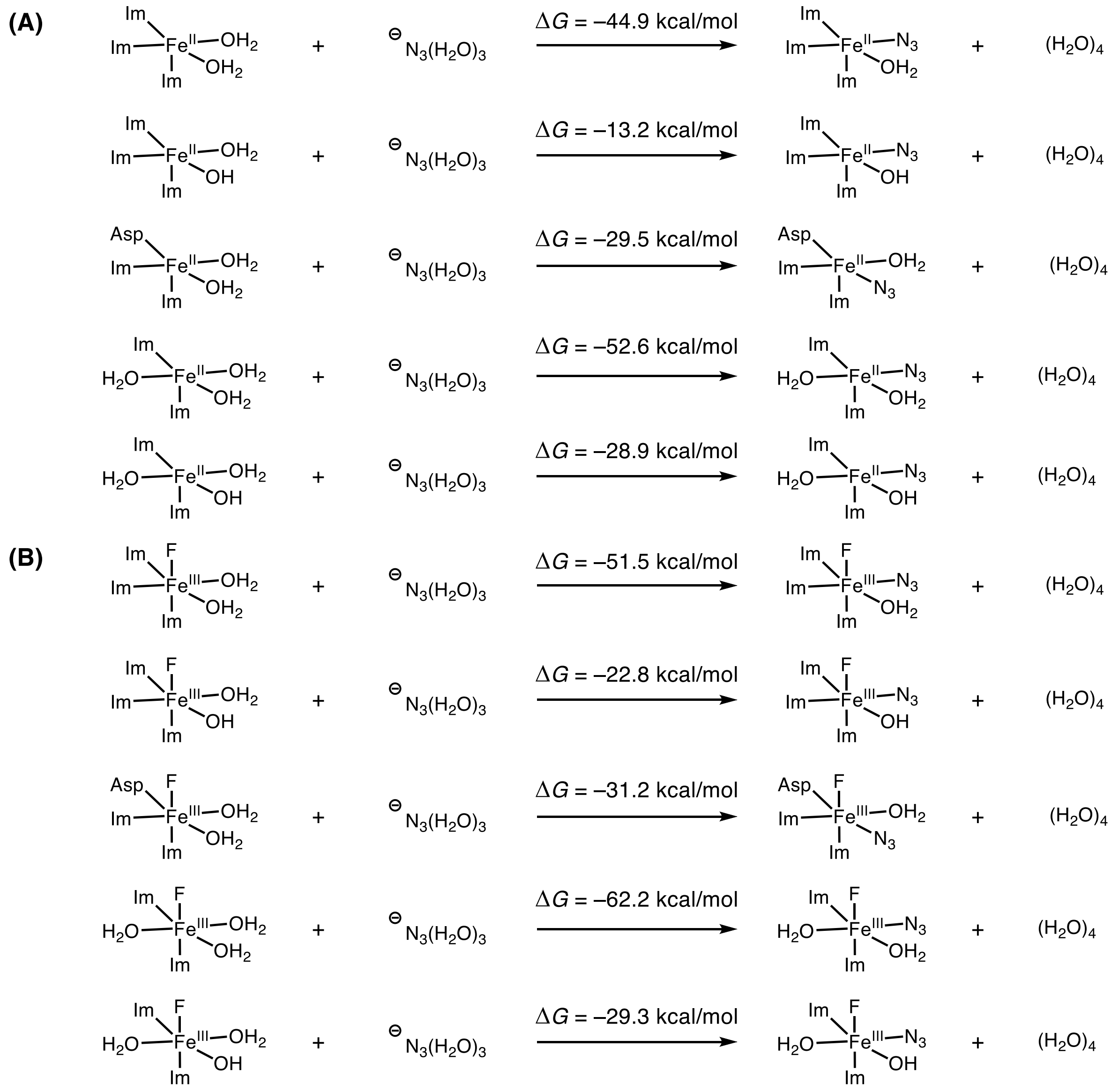

Figure S25. Calculated free energies for azide binding to Fe(II) and Fe(III)–F intermediates. Fe(II) and Fe(III)–F intermediates were calculated at high-spin quintet and sextet states, respectively.

15. Radical Rebound Transition States from QM/MM Calculations

Transition states of the fluorine and hydroxy rebound pathways in $EgtB_{CHF1}$ were investigated using QM/MM ONIOM calculations. The fluorine rebound transition states leading to (*R*)-**2** and (*S*)-**2** are nearly identical in energy, in agreement with the modest enantioselectivity observed experimentally. Interestingly, because of the steric repulsion between phenyl group on the benzyl radical and H134, the hydroxy rebound **TS-3-OH-S$_{\mathbf{QM/MM}}$** is calculated to be 1.0 kcal/mol higher in energy than the fluorine radical rebound **TS-3$_{\mathbf{QM/MM}}$**. Given the relatively small activation free energy difference, these calculations suggest that fluorine and hydroxy radical rebound are energetically competitive.

It is worth noting that the truncated model and the QM/MM calculations predict different trends in the relative fluorine and hydroxy rebound barriers. In the truncated model, hydroxy rebound from the Fe^{III}(F)(OH) intermediates are calculated to be more favorable than the corresponding fluorine radical rebounds (**Table 6**). In contrast, the QM/MM calculations predict fluorine rebound to be slightly lower in energy. This difference likely reflects the explicit protein environment included in the QM/MM calculations, which captures steric and noncovalent interactions absent from the truncated model. Nevertheless, both computational approaches consistently indicate that fluorine and hydroxy rebound pathways have similar activation barriers.

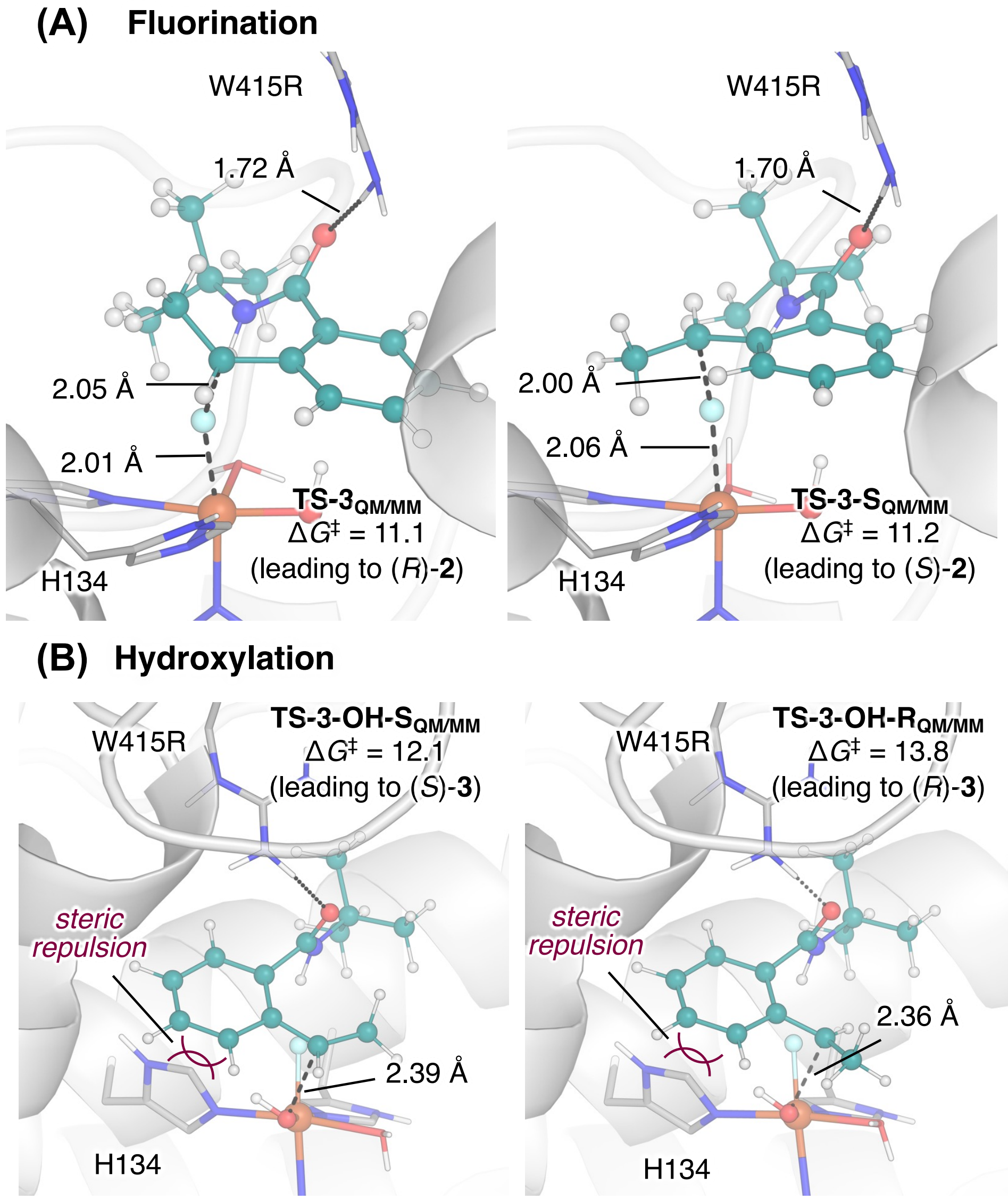

Figure S26. QM/MM-optimized geometries of the radical rebound transition states for the C–H fluorination and hydroxylation at high-spin quintet state. Gibbs free energy values are in kcal/mol with respect to the Fe(III)–F intermediates.

Furthermore, we have also considered the hydroxylation pathway involved in the equatorial-to-axial isomerization (**Figure S27**). However, the isomerization transition state **TS-4** is calculated to be 3.0 kcal/mol higher in energy than **TS-3-OH-S**$_{\mathbf{QM/MM}}$, which disfavors this scenario.

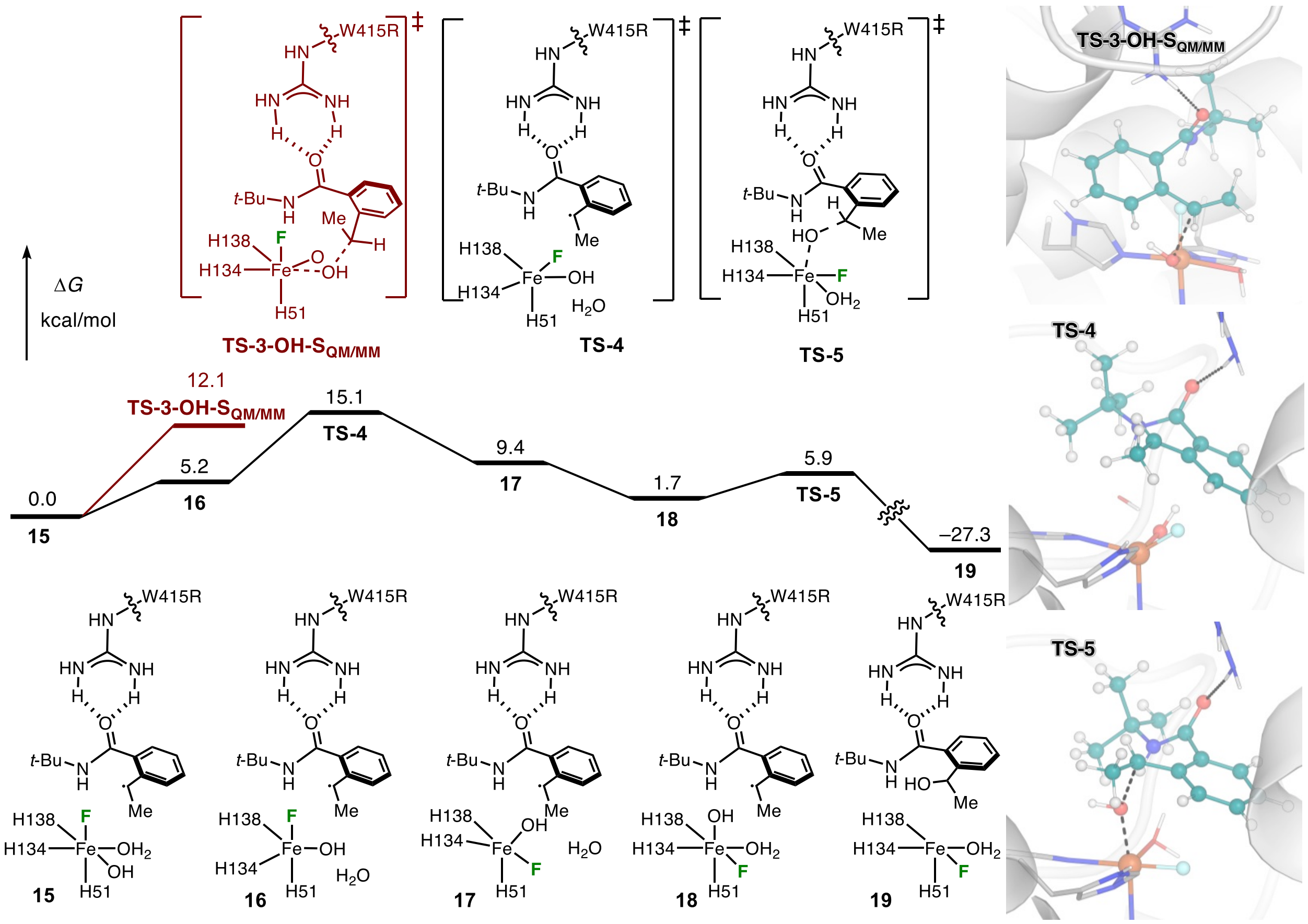

Figure S27. QM/MM-calculated free energy profile for the hydroxyl rebound pathway involving equatorial-to-axial isomerization of the hydroxyl ligand at high-spin quintet state. Gibbs free energy values are in kcal/mol with respect to the Fe(III)–F intermediates **15**.

16. Classical MD Simulations for EgtB$_{CHF1}$ H138D Variant

To gain qualitative insight into the origin of the enantioselectivity in EgtB$_{CHF1}$ and EgtB$_{CHF1}$ H138D variants, we analyzed the N···H^R/H^S distances in the near-attack-conformation (NACs). Interestingly, in EgtB$_{CHF1}$ enzyme, the H^R atom is closer to nitrogen atom in *N*-fluoroamide **1**, favoring the activation of the C–H^R bond. On the other hand, in EgtB$_{CHF1}$ H138D, the H138D mutation repositions the substrate toward the 375–385 loop and induces rotation of the ethyl group to relieve steric interactions. As a result, the H^S atom becomes preferentially oriented toward the nitrogen atom nitrogen atom in *N*-fluoroamide **1**, favoring the activation of C–H^S bond. These MD

simulations provide a qualitative structural rationale for the altered enantioselectivity observed in the H138D variant.

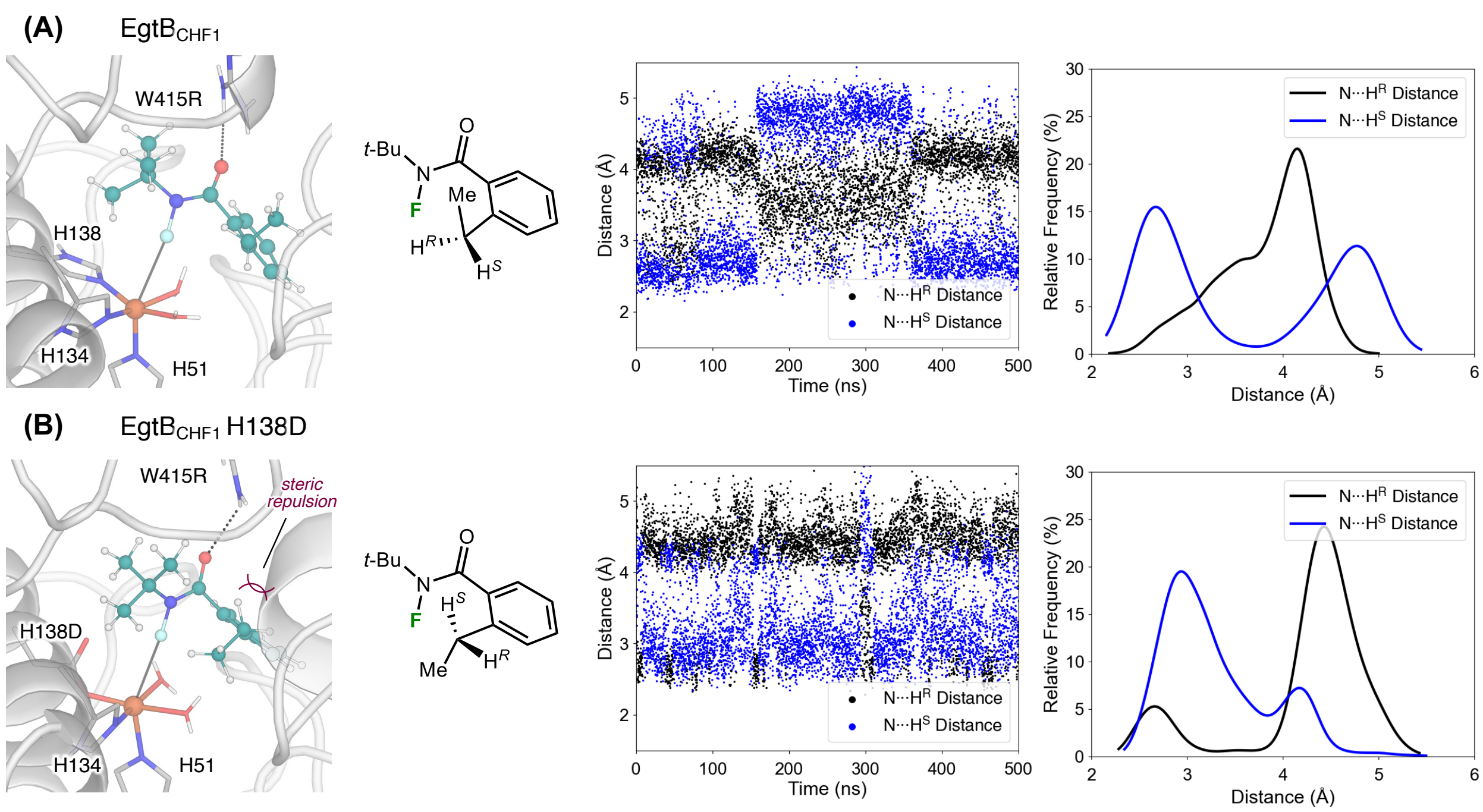

Figure S28. The most populated structures of EgtB$_{CHF1}$ and EgtB$_{CHF1}$ H138D; Time evolution and distribution of N···H^R/H^S distances in *N*-fluoroamide **1** obtained from classical MD simulation.

XII. References

(1) Goncharenko, K. V.; Vit, A.; Blankenfeldt, W.; Seebeck, F. P. Structure of the sulfoxide synthase EgtB from the ergothioneine biosynthetic pathway. *Angew. Chem. Int. Ed.* **2015**, *54* (9), 2821-2824.

(2) Kille, S.; Acevedo-Rocha, C. G.; Parra, L. P.; Zhang, Z.-G.; Opperman, D. J.; Reetz, M. T.; Acevedo, J. P. Reducing codon redundancy and screening effort of combinatorial protein libraries created by saturation mutagenesis. *ACS Synth. Biol.* **2013**, *2* (2), 83-92.

(3) Gibson, D. G.; Young, L.; Chuang, R.-Y.; Venter, J. C.; Hutchison III, C. A.; Smith, H. O. Enzymatic assembly of DNA molecules up to several hundred kilobases. *Nat. Methods* **2009**, *6* (5), 343-345.

(4) Wayne, W. [27] Rapid colorimetric micromethod for the quantitation of complexed iron in biological samples. In *Methods Enzymol.*, Vol. 158; Elsevier, 1988; pp 357-364.

(5) Groendyke, B. J.; AbuSalim, D. I.; Cook, S. P. Iron-catalyzed, fluoroamide-directed C–H fluorination. *J. Am. Chem. Soc.* **2016**, *138* (39), 12771-12774.

(6) Corey, E. J.; Helal, C. J. Reduction of carbonyl compounds with chiral oxazaborolidine catalysts: a new paradigm for enantioselective catalysis and a powerful new synthetic method. *Angew. Chem. Int. Ed.* **1998**, *37* (15), 1986-2012.

(7) Rui, J.; Zhao, Q.; Huls, A. J.; Soler, J.; Paris, J. C.; Chen, Z.; Reshetnikov, V.; Yang, Y.; Guo, Y.; Garcia-Borràs, M. Directed evolution of nonheme iron enzymes to access abiological radical-relay C(sp^3)-H azidation. *Science* **2022**, *376* (6595), 869-874.

(8) Schrodinger, L. The PyMOL molecular graphics system. **2021**.

(9) (a) Becke, A. D. Density-functional thermochemistry. III. The role of exact exchange. *J. Chem. Phys.* **1993**, *98* (7), 5648-5652; (b) Becke, A. D. Density-functional exchange-energy approximation with correct asymptotic behavior. *Phys. Rev. A* **1988**, *38* (6), 3098-3100.

(10) Frisch, M. J.; Trucks, G. W.; Schlegel, H. B.; Scuseria, G. E.; Robb, M. A.; Cheeseman, J. R.; Scalmani, G.; Barone, V.; Petersson, G. A.; Nakatsuji, H.; Li, X.; Caricato, M.; Marenich, A. V.; Bloino, J.; Janesko, B. G.; Gomperts, R.; Mennucci, B.; Hratchian, H. P.; Ortiz, J. V.; Izmaylov, A. F.; Sonnenberg, J. L.; Williams; Ding, F.; Lipparini, F.; Egidi, F.; Goings, J.; Peng, B.; Petrone, A.; Henderson, T.; Ranasinghe, D.; Zakrzewski, V. G.; Gao, J.; Rega, N.; Zheng, G.; Liang, W.; Hada, M.; Ehara, M.; Toyota, K.; Fukuda, R.; Hasegawa, J.; Ishida, M.; Nakajima, T.; Honda, Y.; Kitao, O.; Nakai, H.; Vreven, T.; Throssell, K.; Montgomery Jr., J. A.; Peralta, J. E.; Ogliaro, F.; Bearpark, M. J.; Heyd, J. J.; Brothers, E. N.; Kudin, K. N.; Staroverov, V. N.; Keith, T. A.; Kobayashi, R.; Normand, J.; Raghavachari, K.; Rendell, A. P.; Burant, J. C.; Iyengar, S. S.; Tomasi, J.; Cossi, M.; Millam, J. M.; Klene, M.; Adamo, C.; Cammi, R.; Ochterski, J. W.; Martin,

R. L.; Morokuma, K.; Farkas, O.; Foresman, J. B.; Fox, D. J. Gaussian 16 Rev. C.01, Wallingford, CT, **2016**.

(11) Morris, G. M.; Huey, R.; Lindstrom, W.; Sanner, M. F.; Belew, R. K.; Goodsell, D. S.; Olson, A. J. AutoDock4 and AutoDockTools4: Automated docking with selective receptor flexibility. *J. Comput. Chem.* **2009**, *30* (16), 2785-2791.

(12) Salomon-Ferrer, R.; Gotz, A. W.; Poole, D.; Le Grand, S.; Walker, R. C. Routine microsecond molecular dynamics simulations with AMBER on GPUs. 2. Explicit solvent particle mesh Ewald. *J. Chem. Theory Comput.* **2013**, *9* (9), 3878-3888.

(13) Case, D. A.; Aktulga, H. M.; Belfon, K.; Ben-Shalom, I. Y.; Berryman, J. T.; Brozell, S. R.; Cerutti, D. S.; T.E. Cheatham, I.; Cisneros, G. A.; Cruzeiro, V. W. D.; Darden, T. A.; Duke, R. E.; Giambasu, G.; Gilson, M. K.; Gohlke, H.; Goetz, A. W.; Harris, R.; Izadi, S.; Izmailov, S. A.; Kasavajhala, K.; Kaymak, M. C.; King, E.; Kovalenko, A.; Kurtzman, T.; Lee, T. S.; LeGrand, S.; Li, P.; Lin, C.; Liu, J.; Luchko, T.; Luo, R.; Machado, M.; Man, V.; Manathunga, M.; Merz, K. M.; Miao, Y.; Mikhailovskii, O.; Monard, G.; Nguyen, H.; O'Hearn, K. A.; Onufriev, A.; F. Pan, S. P.; Qi, R.; Rahnamoun, A.; Roe, D. R.; Roitberg, A.; Sagui, C.; Schott-Verdugo, S.; Shajan, A.; Shen, J.; Simmerling, C. L.; Skrynnikov, N. R.; Smith, J.; Swails, J.; Walker, R. C.; Wang, J.; Wang, J.; Wei, H.; Wolf, R. M.; Wu, X.; Xiong, Y.; Xue, Y.; York, D. M.; Zhao, S.; Kollman, P. A. Amber 20, University of California, San Francisco, CA, **2016**.

(14) Maier, J. A.; Martinez, C.; Kasavajhala, K.; Wickstrom, L.; Hauser, K. E.; Simmerling, C. ff14SB: improving the accuracy of protein side chain and backbone parameters from ff99SB. *J. Chem. Theory Comput.* **2015**, *11* (8), 3696-3713.

(15) Li, P.; Merz Jr, K. M. MCPB. py: A python based metal center parameter builder. *J. Chem. Inf. Model.* **2016**, *56*, 599-604.

(16) Wang, J.; Wolf, R. M.; Caldwell, J. W.; Kollman, P. A.; Case, D. A. Development and testing of a general amber force field. *J. Comput. Chem.* **2004**, *25* (9), 1157-1174.

(17) (a) Singh, U. C.; Kollman, P. A. An approach to computing electrostatic charges for molecules. *J. Comp. Chem.* **1984**, *5* (2), 129-145.; (b) Besler, B. H.; Merz Jr, K. M.; Kollman, P.

A. Atomic charges derived from semiempirical methods. *J. Comp. Chem.* **1990**, *11* (4), 431-439.; (c) Becke, A. D. Density-functional thermochemistry. III. The role of exact exchange. *J. Chem. Phys.* **1993**, *98* (7), 5648-5652.
(18) Bayly, C. I.; Cieplak, P.; Cornell, W.; Kollman, P. A. A well-behaved electrostatic potential based method using charge restraints for deriving atomic charges: the RESP model. *J. Phys. Chem.* **1993**, *97* (40), 10269-10280.
(19) (a) Huang, J.; Li, C.; Wang, B.; Sharon, D. A.; Wu, W.; Shaik, S. Selective Chlorination of Substrates by the Halogenase SyrB2 Is Controlled by the Protein According to a Combined Quantum Mechanics/Molecular Mechanics and Molecular Dynamics Study. *ACS Catal.* **2016**, *6* (4), 2694-2704; (b) Timmins, A.; Saint-André, M.; de Visser, S. P. Understanding How Prolyl-4-hydroxylase Structure Steers a Ferryl Oxidant toward Scission of a Strong C–H Bond. *J. Am. Chem. Soc.* **2017**, *139* (29), 9855-9866; (c) Kastner, D. W.; Nandy, A.; Mehmood, R.; Kulik, H. J. Mechanistic Insights into Substrate Positioning That Distinguish Non-heme Fe(II)/α-Ketoglutarate-Dependent Halogenases and Hydroxylases. *ACS Catal.* **2023**, *13* (4), 2489-2501.
(20) Anandakrishnan, R.; Aguilar, B.; Onufriev, A. V. H++ 3.0: automating p K prediction and the preparation of biomolecular structures for atomistic molecular modeling and simulations. *Nucleic Acids Res.* **2012**, *40* (W1), W537-W541.
(21) Jorgensen, W. L.; Chandrasekhar, J.; Madura, J. D.; Impey, R. W.; Klein, M. L. Comparison of simple potential functions for simulating liquid water. *J. Chem. Phys.* **1983**, *79* (2), 926-935.
(22) Ryckaert, J.-P.; Ciccotti, G.; Berendsen, H. J. Numerical integration of the cartesian equations of motion of a system with constraints: molecular dynamics of n-alkanes. *J. Comput. Phys.* **1977**, *23* (3), 327-341.
(23) Darden, T.; York, D.; Pedersen, L. Particle mesh Ewald: An N log (N) method for Ewald sums in large systems. *J. Chem. Phys.* **1993**, *98* (12), 10089-10092.
(24) Roe, D. R.; Cheatham III, T. E. PTRAJ and CPPTRAJ: software for processing and analysis of molecular dynamics trajectory data. *J. Chem. Theory Comput.* **2013**, *9* (7), 3084-3095.

(25) Grimme, S.; Ehrlich, S.; Goerigk, L. Effect of the damping function in dispersion corrected density functional theory. *J. Comput. Chem.* **2011**, *32* (7), 1456-1465.

(26) Lee, C.; Yang, W.; Parr, R. G. Development of the Colle-Salvetti correlation-energy formula into a functional of the electron density. *Phys. Rev. B* **1988**, *37* (2), 785-789.

(27) Ribeiro, R. F.; Marenich, A. V.; Cramer, C. J.; Truhlar, D. G. Use of solution-phase vibrational frequencies in continuum models for the free energy of solvation. *J. Phys. Chem. B* **2011**, *115* (49), 14556-14562.

(28) Luchini, G.; Alegre-Requena, J. V.; Funes-Ardoiz, I.; Paton, R. S. GoodVibes: automated thermochemistry for heterogeneous computational chemistry data. *F1000Research* **2020**, *9* (291), 291.

(29) Weigend, F.; Ahlrichs, R. Balanced basis sets of split valence, triple zeta valence and quadruple zeta valence quality for H to Rn: Design and assessment of accuracy. *Phys. Chem. Chem. Phys.* **2005**, *7* (18), 3297-3305.

(30) Marenich, A. V.; Cramer, C. J.; Truhlar, D. G. Universal solvation model based on solute electron density and on a continuum model of the solvent defined by the bulk dielectric constant and atomic surface tensions. *J. Phys. Chem. B* **2009**, *113* (18), 6378-6396.

(31) (a) Schutz, C. N.; Warshel, A. What are the dielectric "constants" of proteins and how to validate electrostatic models? *Proteins: Struct., Funct., Bioinf.* **2001**, *44* (4), 400-417; (b) Li, L.; Li, C.; Zhang, Z.; Alexov, E. On the dielectric "constant" of proteins: smooth dielectric function for macromolecular modeling and its implementation in DelPhi. *J. Chem. Theory Comput.* **2013**, *9* (4), 2126-2136.

(32) Konkoli, Z.; Cremer, D. A new way of analyzing vibrational spectra. I. Derivation of adiabatic internal modes. *Int. J. Quantum Chem.* **1998**, *67* (1), 1-9.

(33) Chung, L. W.; Sameera, W.; Ramozzi, R.; Page, A. J.; Hatanaka, M.; Petrova, G. P.; Harris, T. V.; Li, X.; Ke, Z.; Liu, F. The ONIOM method and its applications. *Chem. Rev.* **2015**, *115* (12), 5678-5796.

(34) Vreven, T.; Frisch, M.; Kudin, K.; Schlegel, H.; Morokuma, K. Geometry optimization with QM/MM methods II: Explicit quadratic coupling. *Mol. Phys.* **2006**, *104* (5-7), 701-714.
(35) Lin, H.; Truhlar, D. G. QM/MM: what have we learned, where are we, and where do we go from here? *Theor. Chem. Acc.* **2007**, *117* (2), 185-199.
(36) Lian, P.; Johnston, R. C.; Parks, J. M.; Smith, J. C. Quantum chemical calculation of p K as of environmentally relevant functional groups: Carboxylic acids, amines, and thiols in aqueous solution. *J. Phys. Chem. A* **2018**, *122* (17), 4366-4374.
(37) Zhao, L. P.; Mai, B. K.; Cheng, L.; Zhao, Y.; Guo, R.; Liu, P.; Yang, Y. Biocatalytic enantioselective C(sp^3)-H fluorination enabled by directed evolution of non-haem iron enzymes. *Nat. Synth.* **2024**, *3* (8), 967-975.
(38) Liptak, M. D.; Shields, G. C. Accurate p K a calculations for carboxylic acids using complete basis set and Gaussian-n models combined with CPCM continuum solvation methods. *J. Am. Chem. Soc.* **2001**, *123* (30), 7314-7319.
(39) (a) Kelly, C. P.; Cramer, C. J.; Truhlar, D. G. Aqueous solvation free energies of ions and ion− water clusters based on an accurate value for the absolute aqueous solvation free energy of the proton. *J. Phys. Chem. B* **2006**, *110* (32), 16066-16081; (b) Isse, A. A.; Gennaro, A. Absolute potential of the standard hydrogen electrode and the problem of interconversion of potentials in different solvents. *J. Phys. Chem. B* **2010**, *114* (23), 7894-7899; (c) Thapa, B.; Schlegel, H. B. Density functional theory calculation of p K a's of thiols in aqueous solution using explicit water molecules and the polarizable continuum model. *J. Phys. Chem. A* **2016**, *120* (28), 5726-5735.
(40) Jiao, D.; Rempe, S. B. Combined density functional theory (DFT) and continuum calculations of p K a in carbonic anhydrase. *Biochemistry* **2012**, *51* (30), 5979-5989.
(41) Shokhen, M.; Khazanov, N.; Albeck, A. Challenging a paradigm: Theoretical calculations of the protonation state of the Cys25-His159 catalytic diad in free papain. *Proteins: Struct., Funct., Bioinf.* **2009**, *77* (4), 916-926.
(42) Bartmess, J. E. Thermodynamics of the electron and the proton. *J. Phys. Chem.* **1994**, *98* (25), 6420-6424.

XIII. Cartesian Coordinates and Energies

1
Charge: 0
Multiplicity: 1
B3LYP-D3(BJ)/6-31G(d)-SDD SCF energy (au): -736.044094994
B3LYP-D3(BJ)/6-31G(d)-SDD enthalpy (au): -735.737527994
B3LYP-D3(BJ)/6-31G(d)-SDD free energy (au): -735.798616994
B3LYP-D3(BJ)/def2-TZVP/SMD SCF energy (au): -736.354925435
B3LYP-D3(BJ)/def2-TZVP/SMD enthalpy (au): -736.048358435
B3LYP-D3(BJ)/def2-TZVP/SMD free energy (au): -736.109447435
B3LYP-D3(BJ)/def2-TZVP/SMD free energy (quasi-harmonic) (au): -736.107411441

Cartesian coordinates

ATOM	X	Y	Z
C	-1.0265550000	-0.3727870000	-0.1814150000
C	-1.2114450000	-1.7075490000	-0.5763740000
C	-2.4573880000	-2.3142090000	-0.4723780000
C	-3.5295060000	-1.5916820000	0.0548480000
C	-3.3442360000	-0.2728520000	0.4609660000
C	-2.1045140000	0.3697300000	0.3433570000
H	-0.3686280000	-2.2612130000	-0.9745870000
H	-2.5901710000	-3.3428730000	-0.7938510000
H	-4.5067760000	-2.0562220000	0.1529220000
H	-4.1791180000	0.2831760000	0.8805380000
C	-1.9995290000	1.8170780000	0.7741850000
H	-0.9805570000	2.0554870000	1.0885850000
H	-2.6493150000	1.9640330000	1.6460830000
C	-2.4187110000	2.7867910000	-0.3431180000
H	-2.3715360000	3.8235390000	0.0089900000
H	-3.4442480000	2.5845510000	-0.6728330000
H	-1.7487040000	2.6793070000	-1.1991670000
C	0.2999870000	0.2778010000	-0.3985520000
O	0.4288820000	1.4113690000	-0.8444520000
N	1.4283510000	-0.5229330000	-0.1756700000
C	2.7973820000	0.0295320000	0.0265410000
C	3.7286180000	-1.1618490000	0.3000080000
C	3.2321710000	0.7043560000	-1.2851520000
C	2.8086710000	1.0135120000	1.2055670000
H	3.4960550000	-1.6490090000	1.2485120000
H	3.6524860000	-1.9037220000	-0.5013390000
H	4.7607210000	-0.8001930000	0.3398810000
H	2.6327340000	1.5870920000	-1.5006850000
H	4.2838110000	0.9961430000	-1.1923350000
H	3.1435440000	0.0036870000	-2.1218300000
H	3.8292560000	1.3618530000	1.3957390000
H	2.1805530000	1.8814520000	0.9901880000
H	2.4392490000	0.5211370000	2.1109130000
F	1.1594720000	-1.4509850000	0.8735580000

1_Arg
Charge: 1
Multiplicity: 1

B3LYP-D3(BJ)/6-31G(d)-SDD SCF energy (au): -981.175059921
B3LYP-D3(BJ)/6-31G(d)-SDD enthalpy (au): -980.740712921
B3LYP-D3(BJ)/6-31G(d)-SDD free energy (au): -980.822855921
B3LYP-D3(BJ)/def2-TZVP/SMD SCF energy (au): -981.653755640
B3LYP-D3(BJ)/def2-TZVP/SMD enthalpy (au): -981.219408640
B3LYP-D3(BJ)/def2-TZVP/SMD free energy (au): -981.301551640
B3LYP-D3(BJ)/def2-TZVP/SMD free energy (quasi-harmonic) (au): -981.295012719

Cartesian coordinates

ATOM	X	Y	Z
H	33.5365590000	40.6350500000	30.5518230000
C	34.5394700000	40.2054550000	30.4413190000
N	34.4948450000	38.7591470000	30.2435280000
C	34.1806730000	37.8774770000	31.2034570000
N	33.8266860000	38.2956400000	32.4244650000
N	34.2187650000	36.5619120000	30.9617780000
H	35.0128240000	40.6555860000	29.5685320000
H	35.1454120000	40.4455690000	31.3198370000
H	34.5695340000	38.4047800000	29.3003440000
H	33.5223080000	39.2436480000	32.5847590000
H	33.6606940000	37.5881400000	33.1400200000
H	34.7887590000	36.1884600000	30.2159660000
H	34.0087440000	35.9450150000	31.7511550000
N	34.7058890000	34.4889160000	35.2554620000
O	33.7461420000	35.7418380000	33.6438900000
C	35.3452010000	34.1303340000	32.9150280000
C	34.6113240000	33.3413410000	32.0194690000
C	35.2415060000	32.7480780000	30.9270140000
C	36.6053240000	32.9635460000	30.7231560000
C	37.3277680000	33.7618020000	31.6110100000
C	36.7213920000	34.3578640000	32.7229320000
C	34.5637140000	34.8512700000	33.9691190000
C	34.2575870000	35.1841480000	36.5055070000
C	34.9292110000	36.5643020000	36.5618970000
C	32.7252360000	35.2961880000	36.4609130000
C	34.6767740000	34.3265980000	37.7059780000
C	37.5194760000	35.2753060000	33.6232480000
C	37.2791280000	36.7628900000	33.3124540000
F	35.7103220000	33.5458850000	35.4589050000
H	33.5494840000	33.1873110000	32.1897910000
H	34.6742630000	32.1193680000	30.2480610000
H	37.1091140000	32.5054350000	29.8773950000
H	38.3890060000	33.9257620000	31.4442990000
H	37.2829610000	35.0721660000	34.6717610000
H	38.5841910000	35.0511730000	33.5007570000
H	37.8861030000	37.3981970000	33.9654320000
H	37.5464870000	36.9901040000	32.2741850000
H	36.2292460000	37.0371580000	33.4571740000
H	34.6231360000	37.0841300000	37.4747270000
H	36.0194950000	36.4669220000	36.5735830000
H	34.6384810000	37.1783570000	35.7052310000
H	32.3888870000	35.8888410000	35.6101380000
H	32.2698530000	34.3032720000	36.4009920000

H	32.3835520000	35.7755820000	37.3833500000
H	35.7622860000	34.2547140000	37.7982810000
H	34.2857400000	34.8015270000	38.6098510000
H	34.2608450000	33.3177890000	37.6407250000

1_water
Charge: 0
Multiplicity: 1
B3LYP-D3(BJ)/6-31G(d)-SDD SCF energy (au): -812.469508689
B3LYP-D3(BJ)/6-31G(d)-SDD enthalpy (au): -812.135146689
B3LYP-D3(BJ)/6-31G(d)-SDD free energy (au): -812.203476689
B3LYP-D3(BJ)/def2-TZVP/SMD SCF energy (au): -812.833279017
B3LYP-D3(BJ)/def2-TZVP/SMD enthalpy (au): -812.498917017
B3LYP-D3(BJ)/def2-TZVP/SMD free energy (au): -812.567247017
B3LYP-D3(BJ)/def2-TZVP/SMD free energy (quasi-harmonic) (au): -812.564434328

Cartesian coordinates

ATOM	X	Y	Z
C	-5.5517070000	-2.4789390000	-4.4590300000
C	-4.9199410000	-1.2405870000	-4.4499820000
C	-3.6473320000	-1.0835590000	-5.0185640000
C	-2.9770620000	-2.1835020000	-5.5896320000
C	-3.6254050000	-3.4253590000	-5.5643500000
C	-4.8969090000	-3.5783880000	-5.0173250000
H	-6.5425680000	-2.5872130000	-4.0283620000
H	-5.4123290000	-0.3809000000	-4.0082440000
H	-3.1131350000	-4.2873950000	-5.9846890000
H	-5.3736770000	-4.5545830000	-5.0207430000
C	-1.6058220000	-2.0915890000	-6.2253200000
C	-1.6824450000	-1.9033030000	-7.7498500000
H	-1.0610460000	-3.0174380000	-6.0029530000
H	-1.0215920000	-1.2720280000	-5.7999720000
H	-0.6763350000	-1.8754350000	-8.1862380000
H	-2.2292660000	-2.7265360000	-8.2229140000
H	-2.1972990000	-0.9690040000	-7.9879860000
C	-3.0381600000	0.2786480000	-5.0953740000
O	-2.6050960000	0.7454920000	-6.1503200000
N	-3.0779960000	1.0504360000	-3.9506300000
C	-2.2103300000	2.2160610000	-3.6100390000
C	-2.2626660000	3.2383990000	-4.7575000000
C	-0.7766650000	1.7316700000	-3.3471950000
H	-1.8153650000	4.1697640000	-4.3945060000
H	-1.7102030000	2.9060850000	-5.6337340000
H	-3.2966740000	3.4459160000	-5.0496610000
H	-0.7722980000	0.9759220000	-2.5558130000
H	-0.3243370000	1.3114150000	-4.2497240000
H	-0.1588940000	2.5743030000	-3.0189840000
C	-2.8163950000	2.8573910000	-2.3504470000
H	-3.8536930000	3.1580260000	-2.5304990000
H	-2.7929510000	2.1753090000	-1.4988030000
H	-2.2365790000	3.7493070000	-2.0950270000
O	0.1573460000	0.8986120000	-6.5151620000
H	-0.8178580000	0.9313660000	-6.5188880000

H	0.3781170000	0.3485070000	-7.2810040000
F	-3.2162990000	0.2416240000	-2.7953080000

2
Charge: 0
Multiplicity: 1
B3LYP-D3(BJ)/6-31G(d)-SDD SCF energy (au): -736.115155606
B3LYP-D3(BJ)/6-31G(d)-SDD enthalpy (au): -735.807546606
B3LYP-D3(BJ)/6-31G(d)-SDD free energy (au): -735.868788606
B3LYP-D3(BJ)/def2-TZVP/SMD SCF energy (au): -736.438269050
B3LYP-D3(BJ)/def2-TZVP/SMD enthalpy (au): -736.130660050
B3LYP-D3(BJ)/def2-TZVP/SMD free energy (au): -736.191902050
B3LYP-D3(BJ)/def2-TZVP/SMD free energy (quasi-harmonic) (au): -736.189814444

Cartesian coordinates

ATOM	X	Y	Z
C	3.0339090000	-0.0772010000	-0.9129220000
O	2.4149420000	-0.5693690000	-1.8504540000
N	3.9812500000	-0.7334360000	-0.1880280000
H	4.4345370000	-0.2100210000	0.5460560000
C	4.2484810000	-2.1859690000	-0.2439180000
C	5.3115810000	-2.4705610000	0.8243040000
C	2.9603390000	-2.9635460000	0.0732370000
C	4.7839220000	-2.5608520000	-1.6351110000
H	6.2280180000	-1.8995130000	0.6316740000
H	4.9431610000	-2.2155920000	1.8258820000
H	5.5701250000	-3.5337190000	0.8234570000
H	2.1962540000	-2.7504270000	-0.6769160000
H	3.1627240000	-4.0405150000	0.0778150000
H	2.5699090000	-2.6705550000	1.0521850000
H	4.9961020000	-3.6351160000	-1.6806360000
H	4.0463010000	-2.3115370000	-2.4000220000
H	5.7100540000	-2.0156780000	-1.8489950000
C	2.8634270000	1.3792800000	-0.5373490000
C	3.2503230000	2.3179010000	-1.5028660000
C	2.2981970000	1.8228520000	0.6700220000
C	3.1330350000	3.6840310000	-1.2655280000
H	3.6457520000	1.9555860000	-2.4469150000
C	2.1676180000	3.2006160000	0.8892720000
C	2.5932680000	4.1284480000	-0.0571310000
H	3.4494380000	4.3970880000	-2.0211080000
H	1.7101970000	3.5481510000	1.8132510000
H	2.4843630000	5.1912650000	0.1384930000
C	1.7948680000	0.8975070000	1.7616730000
C	2.7975910000	0.6638100000	2.8862340000
H	3.0670820000	1.6126080000	3.3605600000
H	2.3654850000	0.0005220000	3.6420450000
H	3.7103030000	0.1993330000	2.5026450000
F	1.4311920000	-0.3441570000	1.2326300000
H	0.8791850000	1.3313290000	2.1828270000

3
Charge: 0

Multiplicity: 1
B3LYP-D3(BJ)/6-31G(d)-SDD SCF energy (au): -712.103839381
B3LYP-D3(BJ)/6-31G(d)-SDD enthalpy (au): -711.782950381
B3LYP-D3(BJ)/6-31G(d)-SDD free energy (au): -711.843650381
B3LYP-D3(BJ)/def2-TZVP/SMD SCF energy (au): -712.415375784
B3LYP-D3(BJ)/def2-TZVP/SMD enthalpy (au): -712.094486784
B3LYP-D3(BJ)/def2-TZVP/SMD free energy (au): -712.155186784
B3LYP-D3(BJ)/def2-TZVP/SMD free energy (quasi-harmonic) (au): -712.153135403

Cartesian coordinates

ATOM	X	Y	Z
C	-0.2702010000	0.3631770000	-3.6333980000
O	-0.7789540000	0.0901640000	-2.5363520000
N	-0.5874100000	-0.2979630000	-4.7754110000
H	-0.2298230000	0.0917840000	-5.6361090000
C	-1.5790390000	-1.3899070000	-4.8968930000
C	-1.5837340000	-1.7989340000	-6.3753850000
C	-1.1472800000	-2.5785700000	-4.0239130000
C	-2.9722500000	-0.8865580000	-4.4838510000
H	-1.8860320000	-0.9617720000	-7.0167360000
H	-0.5919300000	-2.1428260000	-6.6921320000
H	-2.2928030000	-2.6163240000	-6.5359830000
H	-1.1005360000	-2.2838420000	-2.9741910000
H	-1.8656250000	-3.3989130000	-4.1300830000
H	-0.1605490000	-2.9413340000	-4.3320290000
H	-3.7144680000	-1.6800410000	-4.6251990000
H	-2.9739860000	-0.5887350000	-3.4339160000
H	-3.2662270000	-0.0245600000	-5.0923030000
C	0.7766110000	1.4304510000	-3.8011530000
C	1.8971390000	1.1341240000	-4.5945900000
C	0.6836620000	2.6854460000	-3.1647990000
C	2.9273340000	2.0542570000	-4.7668950000
H	1.9729990000	0.1521020000	-5.0527520000
C	1.7232730000	3.6013270000	-3.3660520000
C	2.8358670000	3.3004180000	-4.1488010000
H	3.7907930000	1.7987230000	-5.3738700000
H	1.6502490000	4.5795610000	-2.8962840000
H	3.6239140000	4.0370090000	-4.2777110000
C	-0.4612050000	3.1231380000	-2.2481640000
C	-0.1365660000	2.7819820000	-0.7856600000
H	0.8076440000	3.2400940000	-0.4711570000
H	-0.9405020000	3.1488640000	-0.1389300000
H	-0.0518430000	1.6973940000	-0.6712550000
H	-0.5340090000	4.2132260000	-2.3425290000
O	-1.7369820000	2.6435460000	-2.6288990000
H	-1.7535790000	1.7007920000	-2.3800440000

4
Charge: 0
Multiplicity: 1
B3LYP-D3(BJ)/6-31G(d)-SDD SCF energy (au): -800.474281268
B3LYP-D3(BJ)/6-31G(d)-SDD enthalpy (au): -800.153471268
B3LYP-D3(BJ)/6-31G(d)-SDD free energy (au): -800.218264268

B3LYP-D3(BJ)/def2-TZVP/SMD SCF energy (au): -800.810997620
B3LYP-D3(BJ)/def2-TZVP/SMD enthalpy (au): -800.490187620
B3LYP-D3(BJ)/def2-TZVP/SMD free energy (au): -800.554980620
B3LYP-D3(BJ)/def2-TZVP/SMD free energy (quasi-harmonic) (au): -800.552547352

Cartesian coordinates

ATOM	X	Y	Z
C	-0.1638520000	0.3334340000	-3.6034810000
O	-0.5963860000	0.0340990000	-2.4903130000
N	-0.5322340000	-0.2922260000	-4.7546340000
H	-0.2288500000	0.1428600000	-5.6148030000
C	-1.5592650000	-1.3506150000	-4.8669030000
C	-1.6301630000	-1.7223570000	-6.3534700000
C	-1.1304980000	-2.5724010000	-4.0393920000
C	-2.9207580000	-0.8203560000	-4.3879550000
H	-1.9330060000	-0.8612460000	-6.9623130000
H	-0.6605910000	-2.0837900000	-6.7160460000
H	-2.3675250000	-2.5158680000	-6.5070190000
H	-1.0392580000	-2.3042860000	-2.9854990000
H	-1.8745030000	-3.3704190000	-4.1395380000
H	-0.1652040000	-2.9532370000	-4.3909930000
H	-3.6912790000	-1.5884560000	-4.5187020000
H	-2.8766200000	-0.5455490000	-3.3331160000
H	-3.2128470000	0.0628540000	-4.9667840000
C	0.8969750000	1.3886750000	-3.8038700000
C	2.0803900000	1.0162100000	-4.4544900000
C	0.7515600000	2.7041750000	-3.3257130000
C	3.1281120000	1.9212230000	-4.6195480000
H	2.1816770000	-0.0038650000	-4.8136920000
C	1.8036260000	3.6049670000	-3.5146830000
C	2.9879780000	3.2237010000	-4.1451290000
H	4.0427010000	1.6102110000	-5.1157930000
H	1.6870070000	4.6284660000	-3.1663590000
H	3.7897870000	3.9450580000	-4.2733870000
C	-0.4909780000	3.2002550000	-2.6042490000
C	-0.4657110000	2.8546800000	-1.1101660000
H	0.4371340000	3.2803080000	-0.6614850000
H	-1.3345810000	3.2795840000	-0.5951580000
H	-0.4517660000	1.7711880000	-0.9780810000
H	-0.5049700000	4.2925850000	-2.7084870000
N	-1.7025520000	2.7221530000	-3.3300830000
N	-2.6196560000	2.2580010000	-2.6511550000
N	-3.5390110000	1.8103100000	-2.1420590000

^{5}11’

Charge: 1
Multiplicity: 5
B3LYP-D3(BJ)/6-31G(d)-SDD SCF energy (au): -1693.80364091
B3LYP-D3(BJ)/6-31G(d)-SDD enthalpy (au): -1693.22260791
B3LYP-D3(BJ)/6-31G(d)-SDD free energy (au): -1693.33763091
B3LYP-D3(BJ)/def2-TZVP/SMD SCF energy (au): -2834.36043901
B3LYP-D3(BJ)/def2-TZVP/SMD enthalpy (au): -2833.77940601
B3LYP-D3(BJ)/def2-TZVP/SMD free energy (au): -2833.89442901

B3LYP-D3(BJ)/def2-TZVP/SMD free energy (quasi-harmonic) (au): -2833.88358210

Cartesian coordinates

ATOM	X	Y	Z
Fe	-0.0347160000	-0.8276320000	-1.1589560000
C	-0.5051100000	-1.3740230000	3.1461640000
N	0.7100660000	-0.7207370000	3.0584800000
H	1.2905520000	-0.4404400000	3.8366910000
C	0.9888260000	-0.5129900000	1.7479060000
H	1.8663040000	-0.0107320000	1.3705120000
N	0.0147880000	-1.0023750000	0.9941390000
C	-0.9262280000	-1.5405800000	1.8534400000
H	-1.8330310000	-1.9856330000	1.4744750000
H	-0.9449600000	-1.6462560000	4.0926150000
C	1.4609840000	-4.5345150000	-2.7561190000
N	1.5247000000	-4.7947280000	-1.3986490000
H	1.8807620000	-5.6395860000	-0.9733070000
C	1.0335090000	-3.7219820000	-0.7365260000
H	0.9633850000	-3.6476640000	0.3379800000
N	0.6563390000	-2.7886000000	-1.6000550000
C	0.9190180000	-3.2833340000	-2.8670470000
H	0.6986510000	-2.6919210000	-3.7418650000
H	1.8012820000	-5.2448870000	-3.4928100000
O	-2.1297290000	-1.3998930000	-1.1387150000
H	-2.5666910000	-0.6033580000	-1.5464830000
H	-2.2797780000	-2.1329050000	-1.7581540000
C	-1.2400670000	4.2256280000	-5.2158520000
C	-1.2399630000	3.3772770000	-4.1110590000
C	-2.4527640000	2.8756290000	-3.6132430000
C	-3.6843340000	3.1857920000	-4.2296970000
C	-3.6517850000	4.0609410000	-5.3188120000
C	-2.4512180000	4.5792150000	-5.8091060000
H	-0.3012280000	4.6023810000	-5.6089080000
H	-0.3037210000	3.0729000000	-3.6524790000
H	-4.5878930000	4.3320270000	-5.7987700000
H	-2.4648430000	5.2480770000	-6.6644380000
C	-5.0064090000	2.6344320000	-3.7394210000
C	-5.4312570000	3.1739030000	-2.3644850000
H	-4.9533750000	1.5422550000	-3.6973400000
H	-5.7773240000	2.8867480000	-4.4747520000
H	-6.4201810000	2.7926330000	-2.0910300000
H	-4.7337660000	2.8636550000	-1.5784760000
H	-5.4770360000	4.2683720000	-2.3653460000
C	-2.3841720000	1.9057830000	-2.4925480000
O	-2.8293950000	0.7479350000	-2.5979820000
N	-1.6414370000	2.2931700000	-1.4106360000
C	-1.5443370000	3.5215020000	-0.5524860000
C	-1.9671850000	4.7757460000	-1.3270220000
C	-0.0768960000	3.6399560000	-0.1203960000
H	-1.9042760000	5.6123620000	-0.6247220000
H	-1.3101430000	4.9873070000	-2.1701080000
H	-2.9948620000	4.7140550000	-1.6891380000
H	0.2556970000	2.7388630000	0.4007800000

H	0.5627470000	3.7949980000	-0.9934520000
H	0.0371300000	4.4950790000	0.5524120000
F	-1.4290170000	1.1723660000	-0.5553930000
C	-2.4773250000	3.3338880000	0.6578640000
H	-3.5143380000	3.2002420000	0.3349970000
H	-2.1844660000	2.4767440000	1.2665360000
H	-2.4285890000	4.2309720000	1.2816510000
C	1.8683210000	1.3393320000	-1.9124600000
O	1.3042760000	1.5508690000	-3.0059370000
O	1.5084350000	0.4290650000	-1.0653980000
C	3.0685200000	2.1690860000	-1.5062410000
H	2.9278310000	2.5694250000	-0.4973850000
H	3.9561920000	1.5271270000	-1.4811410000
H	3.2342310000	2.9823290000	-2.2140690000
O	-0.3290320000	-0.4651380000	-3.3028720000
H	0.2925140000	0.3308930000	-3.3646100000
H	-1.2222140000	-0.1106290000	-3.4687760000

[3]11'

Charge: 1
Multiplicity: 3
B3LYP-D3(BJ)/6-31G(d)-SDD SCF energy (au): -1693.77431645
B3LYP-D3(BJ)/6-31G(d)-SDD enthalpy (au): -1693.19253245
B3LYP-D3(BJ)/6-31G(d)-SDD free energy (au): -1693.30447145
B3LYP-D3(BJ)/def2-TZVP/SMD SCF energy (au): -2834.33412287
B3LYP-D3(BJ)/def2-TZVP/SMD enthalpy (au): -2833.75233887
B3LYP-D3(BJ)/def2-TZVP/SMD free energy (au): -2833.86427787
B3LYP-D3(BJ)/def2-TZVP/SMD free energy (quasi-harmonic) (au): -2833.85420442

Cartesian coordinates

ATOM	X	Y	Z
Fe	0.0133060000	-0.7957500000	-1.0716660000
C	-0.2725360000	-1.6762200000	2.9992700000
N	0.9375620000	-1.0111420000	2.9302900000
H	1.5530910000	-0.8156440000	3.7073000000
C	1.1572120000	-0.6493020000	1.6441870000
H	2.0124360000	-0.1027620000	1.2808740000
N	0.1458570000	-1.0546550000	0.8863420000
C	-0.7565960000	-1.6966480000	1.7192000000
H	-1.6819760000	-2.0912040000	1.3319550000
H	-0.6706380000	-2.0536430000	3.9277990000
C	1.2799490000	-4.5403640000	-2.7674370000
N	1.2632910000	-4.8637840000	-1.4227040000
H	1.5311990000	-5.7525820000	-1.0231840000
C	0.8305590000	-3.7828130000	-0.7318320000
H	0.7188290000	-3.7533720000	0.3412600000
N	0.5669800000	-2.7845860000	-1.5644760000
C	0.8454100000	-3.2450840000	-2.8406340000
H	0.7110730000	-2.6019380000	-3.6955550000
H	1.5938290000	-5.2432950000	-3.5228280000
O	-2.0609270000	-1.3385170000	-1.0117090000
H	-2.5001770000	-0.5479440000	-1.4270660000
H	-2.1791520000	-2.0637910000	-1.6474400000

C	-1.2268210000	4.1857320000	-5.2504930000
C	-1.2236980000	3.3748300000	-4.1176940000
C	-2.4316120000	2.8628570000	-3.6208680000
C	-3.6600990000	3.1229720000	-4.2639070000
C	-3.6323390000	3.9616390000	-5.3817190000
C	-2.4369170000	4.4910750000	-5.8723010000
H	-0.2913110000	4.5719140000	-5.6424050000
H	-0.2880980000	3.1093300000	-3.6341160000
H	-4.5674010000	4.1943230000	-5.8834470000
H	-2.4530020000	5.1307960000	-6.7495920000
C	-4.9740600000	2.5537630000	-3.7723030000
C	-5.4353550000	3.1313270000	-2.4246440000
H	-4.8908230000	1.4656700000	-3.6884840000
H	-5.7405230000	2.7559200000	-4.5275430000
H	-6.4166870000	2.7309910000	-2.1510110000
H	-4.7411870000	2.8729470000	-1.6171080000
H	-5.5138030000	4.2230890000	-2.4681210000
C	-2.3593820000	1.9301870000	-2.4681410000
O	-2.7367130000	0.7442050000	-2.5651170000
N	-1.7124410000	2.3796300000	-1.3587890000
C	-1.6052240000	3.6554480000	-0.5774140000
C	-2.0535230000	4.8638180000	-1.4085360000
C	-0.1322570000	3.8063380000	-0.1746710000
H	-2.0068440000	5.7327860000	-0.7453520000
H	-1.4026190000	5.0507120000	-2.2617780000
H	-3.0804210000	4.7645640000	-1.7645600000
H	0.2123760000	2.9370320000	0.3909440000
H	0.4941890000	3.9157250000	-1.0640140000
H	-0.0136390000	4.6977110000	0.4484240000
F	-1.4878720000	1.3063650000	-0.4595790000
C	-2.5186390000	3.5264690000	0.6557240000
H	-3.5594330000	3.3713640000	0.3551540000
H	-2.2122020000	2.7035280000	1.3033770000
H	-2.4648120000	4.4549020000	1.2311800000
C	1.8519490000	1.4131220000	-1.7583990000
O	1.3058900000	1.6151410000	-2.8667580000
O	1.4890840000	0.5081470000	-0.9130410000
C	3.0336700000	2.2615600000	-1.3373290000
H	2.8754850000	2.6591310000	-0.3301680000
H	3.9318740000	1.6346760000	-1.3049860000
H	3.1931710000	3.0777100000	-2.0432180000
O	-0.2128690000	-0.4269190000	-3.0460750000
H	0.3735510000	0.4057130000	-3.1457600000
H	-1.1272250000	-0.1227810000	-3.2153380000

^{5}11

Charge: 2
Multiplicity: 5

B3LYP-D3(BJ)/6-31G(d)-SDD SCF energy (au):	-1691.25400454
B3LYP-D3(BJ)/6-31G(d)-SDD enthalpy (au):	-1690.65150554
B3LYP-D3(BJ)/6-31G(d)-SDD free energy (au):	-1690.76988654
B3LYP-D3(BJ)/def2-TZVP/SMD SCF energy (au):	-2831.91639985
B3LYP-D3(BJ)/def2-TZVP/SMD enthalpy (au):	-2831.31390085

B3LYP-D3(BJ)/def2-TZVP/SMD free energy (au): -2831.43228185
B3LYP-D3(BJ)/def2-TZVP/SMD free energy (quasi-harmonic) (au): -2831.42071131

Cartesian coordinates

ATOM	X	Y	Z
Fe	0.0398830000	-1.1210000000	-0.7156970000
C	1.3301560000	2.1531860000	1.8256930000
N	1.7837640000	2.5930540000	0.5971800000
H	2.2921800000	3.4511590000	0.4269640000
C	1.4448800000	1.6761920000	-0.3383750000
H	1.6655910000	1.7749350000	-1.3891240000
N	0.7975950000	0.6635190000	0.2286010000
C	0.7204330000	0.9522560000	1.5819740000
H	0.2269750000	0.2867180000	2.2727170000
C	-3.0064880000	-1.7330810000	2.2780750000
N	-3.7572060000	-1.1914580000	1.2529330000
H	-4.7522480000	-1.0090620000	1.2797450000
C	-2.9436700000	-0.9529320000	0.2006640000
H	-3.2669540000	-0.5142540000	-0.7299270000
N	-1.6995010000	-1.3151480000	0.4990890000
C	-1.7276920000	-1.8055550000	1.7973490000
H	-0.8376350000	-2.1801770000	2.2783960000
O	1.6087730000	-0.9950130000	-2.2360680000
H	1.7222580000	-1.8732760000	-2.6396220000
H	1.2152380000	-0.4273410000	-2.9249350000
H	-3.4400950000	-2.0130240000	3.2254290000
H	1.4843760000	2.7169270000	2.7325060000
C	2.1797410000	-4.2227730000	1.4548350000
N	3.1118680000	-3.2059040000	1.3958760000
H	4.0533810000	-3.2450870000	1.7656370000
C	2.5584390000	-2.1520250000	0.7560940000
H	3.0669460000	-1.2190960000	0.5675970000
N	1.3118040000	-2.4391070000	0.3951440000
C	1.0663450000	-3.7337070000	0.8274800000
H	0.1111220000	-4.2127880000	0.6750630000
H	2.3894650000	-5.1714330000	1.9238250000
O	-0.5977120000	-2.7852400000	-2.0850330000
H	-1.3205190000	-2.4326420000	-2.6319510000
H	-0.9030610000	-3.6413780000	-1.7430170000
C	-5.2150830000	1.5195840000	-3.1873420000
C	-3.8789190000	1.1835380000	-3.3888330000
C	-2.9170890000	1.5283170000	-2.4291280000
C	-3.2746250000	2.1692030000	-1.2278010000
C	-4.6249880000	2.5008590000	-1.0552850000
C	-5.5836290000	2.1939690000	-2.0206450000
H	-5.9590480000	1.2629940000	-3.9342200000
H	-3.5770190000	0.6688580000	-4.2970200000
H	-4.9257180000	3.0075040000	-0.1420160000
H	-6.6205000000	2.4712290000	-1.8585460000
C	-2.2723610000	2.4788280000	-0.1349880000
C	-1.5895850000	3.8501640000	-0.2624700000
H	-1.5060960000	1.6999050000	-0.0973880000
H	-2.7954880000	2.4365190000	0.8271510000

H	-0.9686830000	4.0430850000	0.6180040000
H	-0.9441810000	3.8910290000	-1.1450710000
H	-2.3254760000	4.6564960000	-0.3405320000
C	-1.5108250000	1.0860040000	-2.6604220000
O	-1.1214310000	-0.0351490000	-2.2671160000
N	-0.6697130000	1.9526040000	-3.2676940000
C	-0.7819820000	3.0783390000	-4.2790130000
C	-2.0471010000	3.9131090000	-4.0383630000
C	-0.8020200000	2.4451610000	-5.6765100000
H	-1.9742380000	4.7813130000	-4.6996400000
H	-2.9623300000	3.3797710000	-4.2889470000
H	-2.1153910000	4.2781390000	-3.0127390000
H	0.0926880000	1.8443460000	-5.8572330000
H	-1.6885360000	1.8163250000	-5.8071400000
H	-0.8359520000	3.2375010000	-6.4295440000
F	0.6088850000	1.3523630000	-3.4399620000
C	0.4537050000	3.9692240000	-4.0753390000
H	0.4789960000	4.3750220000	-3.0588430000
H	1.3841650000	3.4347240000	-4.2733480000
H	0.3939050000	4.8090500000	-4.7720020000

^{3}11

Charge: 2
Multiplicity: 3
B3LYP-D3(BJ)/6-31G(d)-SDD SCF energy (au): -1691.21920689
B3LYP-D3(BJ)/6-31G(d)-SDD enthalpy (au): -1690.61588889
B3LYP-D3(BJ)/6-31G(d)-SDD free energy (au): -1690.72999089
B3LYP-D3(BJ)/def2-TZVP/SMD SCF energy (au): -2831.89220848
B3LYP-D3(BJ)/def2-TZVP/SMD enthalpy (au): -2831.28889048
B3LYP-D3(BJ)/def2-TZVP/SMD free energy (au): -2831.40299248
B3LYP-D3(BJ)/def2-TZVP/SMD free energy (quasi-harmonic) (au): -2831.39095059

Cartesian coordinates

ATOM	X	Y	Z
Fe	0.6459010000	-1.0644500000	-0.1404990000
C	-0.4712530000	2.2903930000	2.0963950000
N	0.3580980000	2.8640600000	1.1499070000
H	0.6158330000	3.8414830000	1.1036690000
C	0.7629010000	1.9101410000	0.2897300000
H	1.4134690000	2.0824430000	-0.5503620000
N	0.2280470000	0.7380310000	0.6335610000
C	-0.5493350000	0.9682470000	1.7636900000
H	-1.0892890000	0.1727980000	2.2509030000
C	-2.5924020000	-2.8926500000	1.7013590000
N	-3.2046820000	-2.1534460000	0.7085850000
H	-4.2004800000	-2.1099090000	0.5325190000
C	-2.2588260000	-1.5122770000	-0.0104910000
H	-2.4538030000	-0.8536690000	-0.8473190000
N	-1.0550280000	-1.8118880000	0.4801540000
C	-1.2508180000	-2.6746680000	1.5505080000
H	-0.4246130000	-3.0714800000	2.1186180000
O	2.3652090000	-0.2374630000	-0.9524160000
H	3.1891370000	-0.7420890000	-0.8439870000

H	2.0951690000	-0.3559800000	-1.8897750000
H	-3.1497710000	-3.4944940000	2.4019500000
H	-0.9126840000	2.8607950000	2.8982050000
C	2.0076560000	-5.0506640000	-0.0525780000
N	3.0108160000	-4.4168900000	0.6495720000
H	3.8159670000	-4.8666690000	1.0682790000
C	2.7344930000	-3.0957510000	0.7001260000
H	3.3474520000	-2.3722520000	1.2177350000
N	1.5925140000	-2.8470460000	0.0593160000
C	1.1276900000	-4.0663630000	-0.4115400000
H	0.1991130000	-4.1479140000	-0.9542440000
H	2.0100340000	-6.1150360000	-0.2289750000
O	0.3166570000	-1.0252580000	-2.3267380000
H	-0.4427380000	-0.3903090000	-2.5103950000
H	0.1092030000	-1.8514230000	-2.7921100000
C	-5.5916060000	2.3978030000	-3.6178110000
C	-4.3071290000	1.8657800000	-3.6061350000
C	-3.4242470000	2.1898810000	-2.5647430000
C	-3.8334910000	2.9839390000	-1.4784800000
C	-5.1365750000	3.4973340000	-1.5101390000
C	-6.0001020000	3.2286070000	-2.5700510000
H	-6.2697130000	2.1662690000	-4.4325100000
H	-3.9720140000	1.2201910000	-4.4126800000
H	-5.4818140000	4.1069140000	-0.6792540000
H	-7.0008430000	3.6489430000	-2.5695630000
C	-2.9621210000	3.2314020000	-0.2623180000
C	-2.3716600000	4.6466610000	-0.1768620000
H	-2.1535570000	2.4946400000	-0.2196100000
H	-3.5782190000	3.0559160000	0.6280270000
H	-1.9074860000	4.8131020000	0.8015930000
H	-1.6120970000	4.8058470000	-0.9479910000
H	-3.1458980000	5.4094360000	-0.3043330000
C	-2.0583260000	1.6099530000	-2.6215010000
O	-1.8648800000	0.3949820000	-2.4498830000
N	-1.0223180000	2.5013980000	-2.7779130000
C	-0.8227000000	3.5407530000	-3.8704470000
C	-2.0405620000	4.4684610000	-3.9732440000
C	-0.5959980000	2.8021490000	-5.1965030000
H	-1.7700400000	5.2661060000	-4.6713170000
H	-2.9234310000	3.9663170000	-4.3663150000
H	-2.2932260000	4.9289330000	-3.0173910000
H	0.2560580000	2.1210420000	-5.1335620000
H	-1.4873710000	2.2334470000	-5.4822730000
H	-0.3934050000	3.5289090000	-5.9882590000
F	0.2038950000	1.7717150000	-2.6895500000
C	0.4064240000	4.3640110000	-3.4615160000
H	0.2459410000	4.8462410000	-2.4917400000
H	1.3095110000	3.7529060000	-3.4123280000
H	0.5696110000	5.1481700000	-4.2051950000

^{5}12'
Charge: 1
Multiplicity: 5

B3LYP-D3(BJ)/6-31G(d)-SDD SCF energy (au): -1693.82554952
B3LYP-D3(BJ)/6-31G(d)-SDD enthalpy (au): -1693.24635252
B3LYP-D3(BJ)/6-31G(d)-SDD free energy (au): -1693.36048152
B3LYP-D3(BJ)/def2-TZVP/SMD SCF energy (au): -2834.38194382
B3LYP-D3(BJ)/def2-TZVP/SMD enthalpy (au): -2833.80274682
B3LYP-D3(BJ)/def2-TZVP/SMD free energy (au): -2833.91687582
B3LYP-D3(BJ)/def2-TZVP/SMD free energy (quasi-harmonic) (au): -2833.90619230

Cartesian coordinates

ATOM	X	Y	Z
Fe	-0.1333820000	0.1252450000	-0.4657430000
C	-3.7528320000	-2.0747990000	0.5303000000
N	-4.1529380000	-0.7502900000	0.5665800000
H	-5.0812470000	-0.4163510000	0.7865270000
C	-3.0930510000	0.0297750000	0.2654700000
H	-3.1056750000	1.1060500000	0.2044410000
N	-2.0297110000	-0.7340260000	0.0341700000
C	-2.4272540000	-2.0496200000	0.1957340000
H	-1.7363880000	-2.8651510000	0.0563370000
H	-4.4299230000	-2.8869150000	0.7426200000
C	1.3209460000	-3.7875320000	-1.6711370000
N	1.8173520000	-3.6713620000	-0.3845620000
H	2.4150610000	-4.3386700000	0.0837920000
C	1.3942790000	-2.4973950000	0.1332250000
H	1.6326640000	-2.1314740000	1.1191190000
N	0.6445700000	-1.8582360000	-0.7565560000
C	0.5928700000	-2.6508320000	-1.8901910000
H	0.0557580000	-2.3274250000	-2.7665720000
H	1.5346020000	-4.6408910000	-2.2949780000
O	-0.8268660000	-0.0527370000	-2.5243920000
H	-0.5442620000	0.8085600000	-2.9429110000
H	-1.7975950000	0.0118580000	-2.4975800000
F	-0.9312190000	1.7718530000	-0.3103940000
C	-0.9328470000	3.2850440000	-2.7666480000
O	-0.0698420000	2.4477870000	-3.1461210000
N	-0.6127350000	4.5796430000	-2.6114160000
H	-3.3490090000	4.1818620000	0.2911210000
C	0.5661710000	5.1867290000	-2.0172910000
C	0.2320700000	6.6649520000	-1.7458660000
C	1.0447840000	4.4932600000	-0.7358630000
C	1.6595030000	5.1149410000	-3.1262670000
H	-0.1363390000	7.1531550000	-2.6521540000
H	-0.5295690000	6.7550540000	-0.9664570000
H	1.1335980000	7.1835410000	-1.4066850000
H	1.2804960000	3.4444010000	-0.9023890000
H	1.9489360000	4.9874090000	-0.3679690000
H	0.2867980000	4.5439370000	0.0507420000
H	2.5433360000	5.6470760000	-2.7593340000
H	1.9219240000	4.0790150000	-3.3458860000
H	1.3102220000	5.5958470000	-4.0433170000
C	-2.3745640000	2.8982830000	-2.6820980000
C	-2.8280440000	1.9668400000	-3.6317310000
C	-3.2445550000	3.3654630000	-1.6777200000

C	-4.1263800000	1.4671520000	-3.5832360000
H	-2.1545810000	1.6556190000	-4.4244160000
C	-4.5441350000	2.8389760000	-1.6410270000
C	-4.9862450000	1.8997840000	-2.5704820000
H	-4.4680790000	0.7633500000	-4.3358280000
H	-5.2223800000	3.1890860000	-0.8663500000
H	-6.0034840000	1.5222900000	-2.5197490000
C	-2.8620830000	4.4166630000	-0.6631610000
C	-3.2871640000	5.8241490000	-1.1162350000
H	-4.3720670000	5.8774650000	-1.2548080000
H	-3.0039070000	6.5728840000	-0.3691280000
H	-2.8121500000	6.0870360000	-2.0660190000
H	-1.7878630000	4.3838370000	-0.4697000000
C	1.6606070000	1.0395550000	1.6508080000
O	2.3723460000	1.6988290000	0.8811390000
O	0.7824320000	0.1508820000	1.2444030000
C	1.7505760000	1.2059080000	3.1474380000
H	0.8165460000	1.6478490000	3.5124690000
H	1.8571860000	0.2301370000	3.6310700000
H	2.5887840000	1.8523820000	3.4092590000
O	1.5400670000	0.9196640000	-1.4448600000
H	2.0225710000	1.3165560000	-0.6435790000
H	1.2576800000	1.6557150000	-2.0300300000

^{3}12'

Charge: 1
Multiplicity: 3
B3LYP-D3(BJ)/6-31G(d)-SDD SCF energy (au): -1693.79805708
B3LYP-D3(BJ)/6-31G(d)-SDD enthalpy (au): -1693.21993008
B3LYP-D3(BJ)/6-31G(d)-SDD free energy (au): -1693.33406008
B3LYP-D3(BJ)/def2-TZVP/SMD SCF energy (au): -2834.36237751
B3LYP-D3(BJ)/def2-TZVP/SMD enthalpy (au): -2833.78425051
B3LYP-D3(BJ)/def2-TZVP/SMD free energy (au): -2833.89838051
B3LYP-D3(BJ)/def2-TZVP/SMD free energy (quasi-harmonic) (au): -2833.88684843

Cartesian coordinates

ATOM	X	Y	Z
Fe	-0.2734510000	-0.1775650000	-0.1454740000
C	-3.0219730000	-2.9070000000	1.3598440000
N	-2.5284660000	-2.2971190000	2.5001310000
H	-2.8227640000	-2.4864870000	3.4486140000
C	-1.5874050000	-1.3990950000	2.1415220000
H	-1.0125600000	-0.7770570000	2.8085440000
N	-1.4607200000	-1.4089870000	0.8212210000
C	-2.3458030000	-2.3425290000	0.3144800000
H	-2.4139200000	-2.5390950000	-0.7435610000
H	-3.7858170000	-3.6671850000	1.3971630000
C	1.4705170000	-3.4067930000	-2.2008040000
N	2.2125790000	-3.3547580000	-1.0343390000
H	3.0039240000	-3.9415500000	-0.8078770000
C	1.7190110000	-2.3726510000	-0.2503360000
H	2.0962540000	-2.0839300000	0.7179210000
N	0.6919530000	-1.8003210000	-0.8640850000

C	0.5222830000	-2.4285210000	-2.0838300000
H	-0.2523340000	-2.1187270000	-2.7661780000
H	1.6833530000	-4.1127550000	-2.9876490000
O	-1.6289460000	-0.2352270000	-1.9857820000
H	-1.3416930000	0.6037660000	-2.4335580000
H	-2.4884900000	0.0066050000	-1.6005640000
F	-1.2637180000	1.2024780000	0.3508650000
C	-1.3155390000	3.2429070000	-2.8297550000
O	-0.6600390000	2.1647840000	-2.7764400000
N	-0.6740790000	4.4227990000	-2.6955250000
H	-2.7350910000	5.2455540000	-1.2265040000
C	0.2838280000	4.8161240000	-1.6690380000
C	0.3302240000	6.3553990000	-1.6536260000
C	-0.0635010000	4.2679000000	-0.2766770000
C	1.6676830000	4.2984350000	-2.1589200000
H	0.5552160000	6.7483120000	-2.6490700000
H	-0.6241570000	6.7723290000	-1.3197240000
H	1.1084450000	6.6863340000	-0.9594110000
H	-0.2649710000	3.1967000000	-0.2862350000
H	0.7662730000	4.4468790000	0.4133690000
H	-0.9504500000	4.7725230000	0.1211920000
H	2.4307230000	4.6728440000	-1.4692730000
H	1.7117610000	3.2098500000	-2.1729480000
H	1.8815540000	4.6701180000	-3.1641790000
C	-2.7514530000	3.2342390000	-3.2084380000
C	-3.2103350000	2.0908250000	-3.8851990000
C	-3.6445310000	4.2923530000	-2.9086030000
C	-4.5411260000	1.9709380000	-4.2659750000
H	-2.5010530000	1.3133030000	-4.1443270000
C	-4.9810990000	4.1409740000	-3.2930680000
C	-5.4299440000	3.0013000000	-3.9601660000
H	-4.8774790000	1.0918880000	-4.8066660000
H	-5.6901310000	4.9290990000	-3.0700530000
H	-6.4744040000	2.9251180000	-4.2476660000
C	-3.1783870000	5.5402310000	-2.1864310000
C	-4.2363040000	6.6153810000	-1.9280010000
H	-5.0479220000	6.2419850000	-1.2943540000
H	-3.7791790000	7.4662020000	-1.4137730000
H	-4.6715650000	6.9872020000	-2.8612850000
H	-2.3593510000	5.9816140000	-2.7709190000
C	1.7471770000	0.8752090000	1.7973040000
O	2.0838840000	1.7245930000	0.9307850000
O	0.9812780000	-0.1329160000	1.5693880000
C	2.2454860000	1.0486580000	3.2150180000
H	1.4497840000	1.5111120000	3.8118300000
H	2.4762930000	0.0787880000	3.6634820000
H	3.1200830000	1.7004020000	3.2397630000
O	0.9887160000	0.8926190000	-1.1041810000
H	1.5146480000	1.3733880000	-0.2920300000
H	0.5222950000	1.5410820000	-1.6880850000

[5]12-A

Charge: 2

Multiplicity: 5
B3LYP-D3(BJ)/6-31G(d)-SDD SCF energy (au): -1691.26926371
B3LYP-D3(BJ)/6-31G(d)-SDD enthalpy (au): -1690.66828371
B3LYP-D3(BJ)/6-31G(d)-SDD free energy (au): -1690.78412971
B3LYP-D3(BJ)/def2-TZVP/SMD SCF energy (au): -2831.93432570
B3LYP-D3(BJ)/def2-TZVP/SMD enthalpy (au): -2831.33334570
B3LYP-D3(BJ)/def2-TZVP/SMD free energy (au): -2831.44919170
B3LYP-D3(BJ)/def2-TZVP/SMD free energy (quasi-harmonic) (au): -2831.43688999

Cartesian coordinates

ATOM	X	Y	Z
Fe	-0.1814540000	-0.0992020000	0.2184810000
C	2.6499270000	-1.1884790000	3.2656830000
N	3.0896060000	0.0628880000	2.8760470000
H	3.8522230000	0.5793900000	3.2974490000
C	2.3276030000	0.4918890000	1.8508060000
H	2.4387530000	1.4419670000	1.3516950000
N	1.4158040000	-0.4325540000	1.5559670000
C	1.6030150000	-1.4870430000	2.4385530000
H	0.9731060000	-2.3626860000	2.4159670000
C	-2.6011560000	-3.1778910000	1.9981840000
N	-2.8453210000	-2.0693830000	2.7891780000
H	-3.4505370000	-2.0438540000	3.6012630000
C	-2.1557300000	-1.0230020000	2.2985250000
H	-2.1531810000	-0.0263170000	2.7129510000
N	-1.4714000000	-1.4092910000	1.2191200000
C	-1.7421540000	-2.7559660000	1.0222290000
H	-1.3160980000	-3.3037250000	0.1970600000
O	1.0659310000	1.1956590000	-0.9138350000
H	1.5213590000	0.7529890000	-1.6504830000
H	0.4924340000	1.9157630000	-1.3248360000
H	-3.0549750000	-4.1368410000	2.1945020000
H	3.1081440000	-1.7339680000	4.0759370000
C	0.6380680000	-3.2118550000	-2.6936610000
N	1.7393990000	-3.3340210000	-1.8681850000
H	2.4903580000	-4.0046370000	-1.9792230000
C	1.6554690000	-2.4054540000	-0.8941290000
H	2.3830830000	-2.2826680000	-0.1075950000
N	0.5448960000	-1.6842870000	-1.0516950000
C	-0.0995390000	-2.1822290000	-2.1781720000
H	-1.0269410000	-1.7543330000	-2.5215200000
H	0.4871470000	-3.8489730000	-3.5512580000
O	-1.6778240000	0.2026640000	-1.2848250000
H	-1.5206170000	1.1201840000	-1.6824260000
H	-2.5543050000	0.2382250000	-0.8639380000
F	-0.8116500000	1.3310480000	1.1238820000
C	-1.4318430000	3.5997350000	-2.3699120000
O	-0.8221690000	2.5204940000	-2.0597280000
N	-0.7993670000	4.7677940000	-2.2028030000
H	-4.3683760000	6.3536510000	-2.0948510000
C	-0.1003040000	5.2967470000	-1.0371140000
C	-0.1475500000	6.8362280000	-1.1280370000
C	-0.6943480000	4.7990370000	0.2875580000

C	1.3818110000	4.8555780000	-1.1955930000
H	0.2290930000	7.1814250000	-2.0940000000
H	-1.1654570000	7.2097120000	-0.9955100000
H	0.4764090000	7.2572680000	-0.3346490000
H	-0.6665200000	3.7086700000	0.3655390000
H	-0.1266210000	5.2173590000	1.1244510000
H	-1.7329340000	5.1298400000	0.3935120000
H	1.9680650000	5.3542000000	-0.4171660000
H	1.4917740000	3.7759930000	-1.0760700000
H	1.7723530000	5.1501340000	-2.1725750000
C	-2.7394550000	3.5337720000	-3.0524160000
C	-3.0461770000	2.3156890000	-3.6957990000
C	-3.6666560000	4.6020610000	-3.0827250000
C	-4.2505270000	2.1417670000	-4.3603030000
H	-2.3066310000	1.5231460000	-3.7057780000
C	-4.8818250000	4.3920840000	-3.7468800000
C	-5.1777530000	3.1879650000	-4.3794680000
H	-4.4636310000	1.2079740000	-4.8704450000
H	-5.6083590000	5.1993020000	-3.7661330000
H	-6.1272890000	3.0665270000	-4.8914980000
C	-3.4183350000	5.9607580000	-2.4741490000
C	-2.8311020000	6.9512160000	-3.4959930000
H	-3.5178070000	7.0866280000	-4.3370930000
H	-2.6667860000	7.9289000000	-3.0324660000
H	-1.8766010000	6.5910320000	-3.8907910000
H	-2.7589890000	5.8794420000	-1.6043730000

^{3}12-A

Charge: 2
Multiplicity: 3
B3LYP-D3(BJ)/6-31G(d)-SDD SCF energy (au): -1691.24777634
B3LYP-D3(BJ)/6-31G(d)-SDD enthalpy (au): -1690.64601234
B3LYP-D3(BJ)/6-31G(d)-SDD free energy (au): -1690.75890734
B3LYP-D3(BJ)/def2-TZVP/SMD SCF energy (au): -2831.91574111
B3LYP-D3(BJ)/def2-TZVP/SMD enthalpy (au): -2831.31397711
B3LYP-D3(BJ)/def2-TZVP/SMD free energy (au): -2831.42687211
B3LYP-D3(BJ)/def2-TZVP/SMD free energy (quasi-harmonic) (au): -2831.41509377

Cartesian coordinates

ATOM	X	Y	Z
Fe	-0.2956380000	-0.2590180000	-0.6342280000
C	1.7281990000	1.3621860000	2.8167810000
N	2.4207780000	1.9466940000	1.7740430000
H	3.1504370000	2.6427830000	1.8659690000
C	1.9582070000	1.4430120000	0.6103690000
H	2.3076300000	1.7327540000	-0.3682980000
N	1.0020660000	0.5526330000	0.8568010000
C	0.8428760000	0.4978880000	2.2330110000
H	0.1072390000	-0.1432810000	2.6935300000
C	-3.3117790000	-1.4932070000	2.0026680000
N	-3.7784980000	-0.2875530000	1.5092720000
H	-4.6541850000	0.1545560000	1.7612240000
C	-2.8817840000	0.2117350000	0.6405810000

H	-2.9825170000	1.1363200000	0.0939710000
N	-1.8485310000	-0.6308410000	0.5494120000
C	-2.1061350000	-1.7016640000	1.3951210000
H	-1.4236470000	-2.5298320000	1.4903900000
O	1.0729580000	0.1219290000	-2.0980650000
H	1.5173640000	-0.6667520000	-2.4557920000
H	0.5742150000	0.5528820000	-2.8572620000
H	-3.8669790000	-2.0775710000	2.7198180000
H	1.9219890000	1.6103100000	3.8487190000
C	0.7981390000	-4.2984040000	-0.5076020000
N	1.7257800000	-3.7422330000	0.3502930000
H	2.4566450000	-4.2453360000	0.8391360000
C	1.5141160000	-2.4132700000	0.4231330000
H	2.0896050000	-1.7308780000	1.0262590000
N	0.4799190000	-2.0890020000	-0.3549490000
C	0.0217000000	-3.2614620000	-0.9446680000
H	-0.8015490000	-3.2524490000	-1.6387190000
H	0.7765220000	-5.3537350000	-0.7311510000
O	-1.4474780000	-1.1067770000	-2.2954980000
H	-1.2690220000	-0.4689450000	-3.0495290000
H	-2.4000060000	-0.9950920000	-2.1244460000
C	-4.7995770000	0.3404960000	-3.1790490000
C	-3.5348120000	0.6052270000	-3.6959810000
C	-2.8247700000	1.7569320000	-3.3100310000
C	-3.3984770000	2.6819910000	-2.4157620000
C	-4.6668780000	2.3832780000	-1.8938720000
C	-5.3618400000	1.2322090000	-2.2598440000
H	-5.3513370000	-0.5342810000	-3.5085420000
H	-3.0990080000	-0.0630570000	-4.4330840000
H	-5.1221020000	3.0887860000	-1.2031150000
H	-6.3517660000	1.0443750000	-1.8546680000
C	-2.7428360000	3.9854790000	-2.0261840000
C	-3.2361010000	5.1492340000	-2.9048000000
H	-1.6543230000	3.8985770000	-2.0867000000
H	-2.9745830000	4.2023440000	-0.9768110000
H	-2.7499770000	6.0859650000	-2.6143740000
H	-3.0232900000	4.9610290000	-3.9616040000
H	-4.3171740000	5.2838760000	-2.8006290000
C	-1.4322340000	1.8849640000	-3.8269510000
O	-0.6889510000	0.8503400000	-3.9190400000
N	-1.0300490000	3.0407830000	-4.3276760000
C	0.2359270000	3.7375610000	-4.4078710000
C	-0.0651300000	5.2152840000	-4.7339160000
C	0.9788610000	3.0960970000	-5.6176060000
H	0.8774590000	5.7358560000	-4.9240300000
H	-0.6965460000	5.2960480000	-5.6220880000
H	-0.5697610000	5.7047500000	-3.8971300000
H	1.1850900000	2.0404220000	-5.4301510000
H	0.3823750000	3.1866750000	-6.5279930000
H	1.9218780000	3.6348820000	-5.7557920000
F	-0.9501190000	1.3619400000	-0.9465900000
C	1.0662920000	3.6215690000	-3.1213300000
H	0.5221820000	4.0268320000	-2.2629220000

H	1.3331320000	2.5835550000	-2.9083630000
H	1.9924270000	4.1924590000	-3.2384080000

[5]12-B
Charge: 1
Multiplicity: 5
B3LYP-D3(BJ)/6-31G(d)-SDD SCF energy (au): -1690.94778462
B3LYP-D3(BJ)/6-31G(d)-SDD enthalpy (au): -1690.35964562
B3LYP-D3(BJ)/6-31G(d)-SDD free energy (au): -1690.47692462
B3LYP-D3(BJ)/def2-TZVP/SMD SCF energy (au): -2831.50199626
B3LYP-D3(BJ)/def2-TZVP/SMD enthalpy (au): -2830.91385726
B3LYP-D3(BJ)/def2-TZVP/SMD free energy (au): -2831.03113626
B3LYP-D3(BJ)/def2-TZVP/SMD free energy (quasi-harmonic) (au): -2831.01777864

Cartesian coordinates

ATOM	X	Y	Z
Fe	-0.0769010000	0.0505360000	0.0890980000
C	2.6005710000	-1.0796200000	3.3471840000
N	3.2390520000	-0.0210880000	2.7244280000
H	4.1052050000	0.4071230000	3.0206570000
C	2.4979230000	0.3596340000	1.6590630000
H	2.7348110000	1.1587370000	0.9732860000
N	1.4181130000	-0.4060400000	1.5686270000
C	1.4668720000	-1.3068030000	2.6159250000
H	0.6917500000	-2.0428240000	2.7594340000
C	-2.4486480000	-3.0781440000	2.2475980000
N	-2.8462860000	-1.8844430000	2.8218140000
H	-3.4939210000	-1.7814650000	3.5908120000
C	-2.2255490000	-0.8713690000	2.1717400000
H	-2.3259700000	0.1765420000	2.4069020000
N	-1.4512870000	-1.3536810000	1.2088080000
C	-1.5810580000	-2.7305630000	1.2466400000
H	-1.0495930000	-3.3634000000	0.5539920000
O	1.1821470000	1.0291140000	-0.8464580000
H	0.7793330000	1.7484100000	-1.3687490000
H	-2.8153320000	-4.0327360000	2.5908650000
H	3.0024010000	-1.5562450000	4.2272820000
C	0.4577810000	-3.3719550000	-2.5984700000
N	1.7429450000	-3.1445110000	-2.1399920000
H	2.5786280000	-3.6155590000	-2.4572100000
C	1.6939780000	-2.1574320000	-1.2141710000
H	2.5557850000	-1.7538050000	-0.7068570000
N	0.4464150000	-1.7498090000	-1.0505510000
C	-0.3372220000	-2.4934510000	-1.9129560000
H	-1.3987900000	-2.3209410000	-1.9866340000
H	0.2387460000	-4.1121740000	-3.3515850000
O	-1.6749300000	0.2344920000	-1.4065930000
H	-1.3978600000	1.0646240000	-1.8839380000
H	-2.4192250000	0.5178990000	-0.8484740000
F	-1.0206080000	1.3575390000	1.0075800000
C	-1.3640780000	3.6174910000	-2.4837600000
O	-0.7067990000	2.5677810000	-2.2788180000
N	-0.7916980000	4.8179710000	-2.2146580000

H	-4.4073530000	6.2863770000	-2.0839050000
C	-0.1699370000	5.2112530000	-0.9523930000
C	-0.1577820000	6.7509250000	-0.9222750000
C	-0.8820820000	4.6354080000	0.2807080000
C	1.3004200000	4.7100690000	-1.0131940000
H	0.3277290000	7.1517770000	-1.8163730000
H	-1.1727610000	7.1545760000	-0.8698910000
H	0.3902230000	7.0929650000	-0.0390640000
H	-0.8774460000	3.5424990000	0.2891160000
H	-0.3743690000	4.9792730000	1.1880750000
H	-1.9187300000	4.9878510000	0.3295630000
H	1.8447280000	5.1630010000	-0.1773750000
H	1.3497680000	3.6255470000	-0.9173550000
H	1.7766540000	5.0142920000	-1.9489560000
C	-2.6824470000	3.5648180000	-3.1714360000
C	-2.9569880000	2.3768010000	-3.8757280000
C	-3.6426430000	4.6016190000	-3.1391290000
C	-4.1607000000	2.1993650000	-4.5435130000
H	-2.1968950000	1.6052050000	-3.9129160000
C	-4.8542640000	4.3919520000	-3.8106600000
C	-5.1184350000	3.2150520000	-4.5058960000
H	-4.3491520000	1.2824270000	-5.0929700000
H	-5.6046680000	5.1774740000	-3.7826300000
H	-6.0680650000	3.0915400000	-5.0181120000
C	-3.4381800000	5.9342740000	-2.4564370000
C	-2.8468030000	6.9887240000	-3.4079140000
H	-3.5087780000	7.1475210000	-4.2654390000
H	-2.7218350000	7.9464810000	-2.8918160000
H	-1.8702530000	6.6690650000	-3.7815270000
H	-2.7974810000	5.8251840000	-1.5775000000

^{3}12-B

Charge: 1
Multiplicity: 3
B3LYP-D3(BJ)/6-31G(d)-SDD SCF energy (au): -1690.93631724
B3LYP-D3(BJ)/6-31G(d)-SDD enthalpy (au): -1690.34751024
B3LYP-D3(BJ)/6-31G(d)-SDD free energy (au): -1690.46082024
B3LYP-D3(BJ)/def2-TZVP/SMD SCF energy (au): -2831.47842479
B3LYP-D3(BJ)/def2-TZVP/SMD enthalpy (au): -2830.88961779
B3LYP-D3(BJ)/def2-TZVP/SMD free energy (au): -2831.00292779
B3LYP-D3(BJ)/def2-TZVP/SMD free energy (quasi-harmonic) (au): -2830.99173955

Cartesian coordinates

ATOM	X	Y	Z
Fe	-0.2510690000	-0.1707470000	-0.7686630000
C	1.7436040000	1.5526530000	2.7532960000
N	2.5226050000	1.9594470000	1.6848900000
H	3.3113950000	2.5892190000	1.7318140000
C	2.0345100000	1.3849210000	0.5584400000
H	2.4249880000	1.5237750000	-0.4379970000
N	0.9887250000	0.6281720000	0.8535720000
C	0.7925390000	0.7261180000	2.2169980000
H	-0.0146970000	0.2041160000	2.7075810000

C	-3.2533350000	-1.2852890000	2.0346120000
N	-3.7883530000	-0.1824840000	1.3943910000
H	-4.6895060000	0.2350300000	1.5808440000
C	-2.9025710000	0.2557500000	0.4721140000
H	-3.0397000000	1.1086710000	-0.1737550000
N	-1.8249700000	-0.5205040000	0.4885850000
C	-2.0279500000	-1.4856080000	1.4590460000
H	-1.2911430000	-2.2467810000	1.6571620000
O	1.1232930000	0.0670760000	-1.9073560000
H	0.7414620000	0.3627160000	-2.7560580000
H	-3.7790350000	-1.8109080000	2.8159290000
H	1.9346340000	1.8815690000	3.7626460000
C	0.7699490000	-4.3112380000	-0.4573120000
N	1.9148090000	-3.6837000000	0.0002300000
H	2.7806310000	-4.1388120000	0.2535630000
C	1.6906990000	-2.3500010000	0.0230050000
H	2.4168600000	-1.6084010000	0.3130910000
N	0.4582430000	-2.0955980000	-0.3867650000
C	-0.1284670000	-3.3082340000	-0.6984940000
H	-1.1283940000	-3.3569690000	-1.0962370000
H	0.7069890000	-5.3815160000	-0.5730340000
O	-1.4836240000	-1.1497410000	-2.3556420000
H	-1.2308170000	-0.5950200000	-3.1378180000
H	-2.4021870000	-0.8796910000	-2.1871840000
C	-4.8778310000	0.3018590000	-3.2629140000
C	-3.6011950000	0.5674310000	-3.7517300000
C	-2.8980880000	1.7175870000	-3.3579080000
C	-3.4937540000	2.6410990000	-2.4761480000
C	-4.7747020000	2.3474790000	-1.9860100000
C	-5.4632460000	1.1956200000	-2.3633490000
H	-5.4140250000	-0.5830000000	-3.5917430000
H	-3.1387370000	-0.1091040000	-4.4634700000
H	-5.2400380000	3.0513470000	-1.2997730000
H	-6.4595870000	1.0059690000	-1.9741710000
C	-2.8386050000	3.9340370000	-2.0517860000
C	-3.2814390000	5.1135120000	-2.9342760000
H	-1.7522820000	3.8282900000	-2.0667780000
H	-3.1085740000	4.1404150000	-1.0088450000
H	-2.7990450000	6.0418770000	-2.6102640000
H	-3.0175830000	4.9345790000	-3.9805720000
H	-4.3656300000	5.2575220000	-2.8773820000
C	-1.4974620000	1.8490440000	-3.8708890000
O	-0.7939540000	0.8337990000	-4.0868780000
N	-1.0919840000	3.0704450000	-4.2810780000
C	0.2340800000	3.6604600000	-4.2048890000
C	0.0707640000	5.1681250000	-4.4696090000
C	1.0354350000	3.0267700000	-5.3809960000
H	1.0569900000	5.6378560000	-4.5343600000
H	-0.4644080000	5.3413110000	-5.4073170000
H	-0.4854850000	5.6476380000	-3.6589650000
H	1.1766890000	1.9576930000	-5.2179950000
H	0.5110100000	3.1768100000	-6.3282690000
H	2.0095020000	3.5249240000	-5.4328080000

F	-1.0102640000	1.4504810000	-1.1136430000
C	0.9563830000	3.4225230000	-2.8703670000
H	0.3996160000	3.8642080000	-2.0387850000
H	1.0903220000	2.3624570000	-2.6566640000
H	1.9422640000	3.8985550000	-2.9116840000

⁵13'

Charge: 1
Multiplicity: 5
B3LYP-D3(BJ)/6-31G(d)-SDD SCF energy (au): -1693.85868757
B3LYP-D3(BJ)/6-31G(d)-SDD enthalpy (au): -1693.27916557
B3LYP-D3(BJ)/6-31G(d)-SDD free energy (au): -1693.39509557
B3LYP-D3(BJ)/def2-TZVP/SMD SCF energy (au): -2834.42177427
B3LYP-D3(BJ)/def2-TZVP/SMD enthalpy (au): -2833.84225227
B3LYP-D3(BJ)/def2-TZVP/SMD free energy (au): -2833.95818227
B3LYP-D3(BJ)/def2-TZVP/SMD free energy (quasi-harmonic) (au): -2833.94602970

Cartesian coordinates

ATOM	X	Y	Z
Fe	-0.0366110000	0.0610980000	-0.5321600000
C	-3.6348650000	-2.1100640000	0.6179660000
N	-3.9494630000	-0.7885340000	0.8833270000
H	-4.8185600000	-0.4541260000	1.2762120000
C	-2.8951920000	-0.0166910000	0.5405490000
H	-2.8458800000	1.0572240000	0.6291220000
N	-1.9185010000	-0.7826260000	0.0655440000
C	-2.3661030000	-2.0907470000	0.1080950000
H	-1.7476390000	-2.9056190000	-0.2318420000
H	-4.3242940000	-2.9161020000	0.8130620000
C	1.1596150000	-3.7584740000	-2.2221640000
N	1.7577560000	-3.7863670000	-0.9744130000
H	2.3633000000	-4.5151480000	-0.6223470000
C	1.4219950000	-2.6566950000	-0.3134350000
H	1.7495940000	-2.3971530000	0.6806030000
N	0.6317820000	-1.9094680000	-1.0740850000
C	0.4621200000	-2.5830740000	-2.2711920000
H	-0.1269180000	-2.1558710000	-3.0660650000
H	1.2912570000	-4.5519220000	-2.9405960000
O	-0.9008090000	0.1110770000	-2.5290050000
H	-0.6028200000	1.0079950000	-2.8732840000
H	-1.8629410000	0.2047380000	-2.4277330000
F	-0.7586510000	1.7049770000	-0.1490660000
C	-0.9315450000	3.3833780000	-2.5402040000
O	-0.0589360000	2.5789140000	-2.9600530000
N	-0.6466880000	4.6225610000	-2.1277410000
H	-1.8523700000	3.9097570000	-0.0516430000
C	0.6905290000	5.2590390000	-1.9697590000
C	0.4084260000	6.7138160000	-1.5707570000
C	1.4681720000	4.5528840000	-0.8495390000
C	1.4571110000	5.2185060000	-3.2996730000
H	-0.1630020000	7.2337080000	-2.3480910000
H	-0.1490200000	6.7658740000	-0.6276170000
H	1.3508210000	7.2497910000	-1.4309430000

H	1.6778650000	3.5131950000	-1.0965820000
H	2.4250650000	5.0586360000	-0.6871790000
H	0.9052610000	4.5676160000	0.0888030000
H	2.4152890000	5.7345920000	-3.1826080000
H	1.6493860000	4.1921350000	-3.6157510000
H	0.8889320000	5.7247220000	-4.0869040000
C	-2.3785070000	2.9974540000	-2.5728480000
C	-2.8102180000	2.3573320000	-3.7363600000
C	-3.3026310000	3.2538310000	-1.5003980000
C	-4.1422510000	1.9735570000	-3.9061710000
H	-2.0902410000	2.1817670000	-4.5300160000
C	-4.6578250000	2.8645340000	-1.7191590000
C	-5.0656920000	2.2399220000	-2.8860700000
H	-4.4580030000	1.4942510000	-4.8269210000
H	-5.3851440000	3.0511690000	-0.9364890000
H	-6.1073240000	1.9587170000	-3.0101410000
C	-2.9094890000	3.8031930000	-0.2547120000
C	-3.8609160000	4.1272040000	0.8539800000
H	-4.2822430000	3.2211870000	1.3204960000
H	-3.3573440000	4.6870220000	1.6460510000
H	-4.7167470000	4.7223080000	0.5108030000
H	-1.4430290000	5.1916610000	-1.8715010000
C	1.9070710000	0.7222920000	1.5397440000
O	2.5623730000	1.4675620000	0.8001940000
O	0.9974160000	-0.1199190000	1.1028300000
C	2.1082380000	0.7180890000	3.0355560000
H	1.2031990000	1.1046060000	3.5176360000
H	2.2541350000	-0.3052740000	3.3946860000
H	2.9621110000	1.3397400000	3.3067880000
O	1.5847140000	0.9054870000	-1.5378410000
H	2.1143940000	1.2330520000	-0.7371370000
H	1.2558180000	1.6910920000	-2.0317870000

^{3}13'

Charge: 1
Multiplicity: 3
B3LYP-D3(BJ)/6-31G(d)-SDD SCF energy (au): -1693.83571627
B3LYP-D3(BJ)/6-31G(d)-SDD enthalpy (au): -1693.25546427
B3LYP-D3(BJ)/6-31G(d)-SDD free energy (au): -1693.36866727
B3LYP-D3(BJ)/def2-TZVP/SMD SCF energy (au): -2834.40136335
B3LYP-D3(BJ)/def2-TZVP/SMD enthalpy (au): -2833.82111135
B3LYP-D3(BJ)/def2-TZVP/SMD free energy (au): -2833.93431435
B3LYP-D3(BJ)/def2-TZVP/SMD free energy (quasi-harmonic) (au): -2833.92314408

Cartesian coordinates

ATOM	X	Y	Z
Fe	-0.0292070000	-0.0405310000	-0.6187560000
C	-3.7314380000	-2.3274390000	0.1179860000
N	-4.0988140000	-1.0205320000	0.3829950000
H	-5.0120170000	-0.7106210000	0.6849080000
C	-3.0270810000	-0.2217050000	0.1722200000
H	-3.0155460000	0.8499310000	0.2914770000
N	-1.9915700000	-0.9538200000	-0.2175830000

C	-2.4160080000	-2.2687840000	-0.2556620000
H	-1.7510950000	-3.0655820000	-0.5500490000
H	-4.4204980000	-3.1510260000	0.2188820000
C	1.4166810000	-3.6526460000	-2.0970060000
N	1.8440640000	-3.6838930000	-0.7797840000
H	2.3985550000	-4.4136020000	-0.3529150000
C	1.4245200000	-2.5624690000	-0.1607310000
H	1.6118780000	-2.2992380000	0.8674840000
N	0.7393050000	-1.8178400000	-1.0220330000
C	0.7283260000	-2.4808310000	-2.2376830000
H	0.2446960000	-2.0508880000	-3.0988490000
H	1.6442650000	-4.4426570000	-2.7947740000
O	-0.7454170000	0.0748050000	-2.5660950000
H	-0.5216040000	1.0185240000	-2.8578040000
H	-1.7137990000	0.0696220000	-2.4724420000
F	-0.8127430000	1.5172800000	-0.2171910000
C	-0.8819790000	3.3611570000	-2.4785210000
O	-0.0498200000	2.5611810000	-2.9833440000
N	-0.5498900000	4.5982820000	-2.0968830000
H	-1.5687220000	3.9672250000	0.0687300000
C	0.7830990000	5.2580310000	-2.1737340000
C	0.5981310000	6.6245040000	-1.4999060000
C	1.8411010000	4.4442160000	-1.4121100000
C	1.1803650000	5.4316270000	-3.6472290000
H	-0.1817750000	7.2118420000	-1.9995640000
H	0.3321450000	6.5111240000	-0.4425850000
H	1.5290990000	7.1945840000	-1.5545770000
H	2.1526530000	3.5657550000	-1.9783720000
H	2.7296650000	5.0650320000	-1.2602790000
H	1.4758470000	4.1233360000	-0.4318520000
H	2.1574200000	5.9212450000	-3.7124710000
H	1.2457630000	4.4607970000	-4.1443270000
H	0.4497410000	6.0512310000	-4.1776650000
C	-2.3231020000	2.9745760000	-2.3592040000
C	-2.8569280000	2.2826550000	-3.4477180000
C	-3.1468260000	3.2787870000	-1.2193180000
C	-4.1958570000	1.8856560000	-3.4761750000
H	-2.2146430000	2.0794750000	-4.2994920000
C	-4.5160280000	2.8825360000	-1.2975200000
C	-5.0244100000	2.1998750000	-2.3900450000
H	-4.5920290000	1.3655760000	-4.3421710000
H	-5.1700980000	3.1079040000	-0.4620820000
H	-6.0723440000	1.9145680000	-2.4068280000
C	-2.6414610000	3.8691590000	-0.0354910000
C	-3.4788450000	4.2329590000	1.1489070000
H	-3.7740280000	3.3444690000	1.7309860000
H	-2.9234020000	4.8839370000	1.8292930000
H	-4.4051900000	4.7487070000	0.8687210000
H	-1.3168880000	5.1840710000	-1.7925690000
C	1.3833770000	0.8624050000	1.6520760000
O	2.0043640000	1.7199330000	1.0181400000
O	0.7476140000	-0.1543010000	1.1034190000
C	1.3085430000	0.8875130000	3.1609510000

H	0.2885920000	1.1582760000	3.4574030000
H	1.5144700000	-0.1046840000	3.5727550000
H	2.0114280000	1.6185480000	3.5621980000
O	1.6732330000	0.9462280000	-1.4688230000
H	1.9703530000	1.3664190000	-0.6051810000
H	1.3071490000	1.6769170000	-2.0120710000

[5]13

Charge: 1
Multiplicity: 5
B3LYP-D3(BJ)/6-31G(d)-SDD SCF energy (au): -1690.98271601
B3LYP-D3(BJ)/6-31G(d)-SDD enthalpy (au): -1690.39465201
B3LYP-D3(BJ)/6-31G(d)-SDD free energy (au): -1690.51169001
B3LYP-D3(BJ)/def2-TZVP/SMD SCF energy (au): -2831.54132052
B3LYP-D3(BJ)/def2-TZVP/SMD enthalpy (au): -2830.95325652
B3LYP-D3(BJ)/def2-TZVP/SMD free energy (au): -2831.07029452
B3LYP-D3(BJ)/def2-TZVP/SMD free energy (quasi-harmonic) (au): -2831.05707651

Cartesian coordinates

ATOM	X	Y	Z
Fe	0.0509230000	0.2135410000	-0.5651570000
C	1.3436160000	-0.2875560000	3.5697400000
N	2.4075600000	0.3411550000	2.9468170000
H	3.2578120000	0.6531540000	3.3953330000
C	2.1109690000	0.4873750000	1.6343680000
H	2.7424030000	0.9462970000	0.8882020000
N	0.9118620000	-0.0207830000	1.3888700000
C	0.4175440000	-0.5043700000	2.5848300000
H	-0.5586630000	-0.9601260000	2.6430420000
C	-3.6759560000	-2.0072740000	0.5697930000
N	-3.9963610000	-0.6771720000	0.7766790000
H	-4.8918630000	-0.3233350000	1.0827660000
C	-2.9084300000	0.0755680000	0.4850070000
H	-2.8565640000	1.1515100000	0.5401290000
N	-1.9062440000	-0.7058370000	0.1027780000
C	-2.3727850000	-2.0068840000	0.1494250000
H	-1.7433340000	-2.8359150000	-0.1322760000
O	1.6141640000	0.8643820000	-1.3063450000
H	1.4305500000	1.6433490000	-1.8633200000
H	-4.3861250000	-2.8025310000	0.7332660000
H	1.3441890000	-0.5084690000	4.6254430000
C	1.1974520000	-3.6940140000	-2.1501570000
N	1.6170610000	-3.8418170000	-0.8405830000
H	2.1180290000	-4.6339530000	-0.4633840000
C	1.2674140000	-2.7258730000	-0.1557850000
H	1.4760150000	-2.5678890000	0.8905840000
N	0.6400250000	-1.8787240000	-0.9570230000
C	0.5926770000	-2.4678330000	-2.2073160000
H	0.1367240000	-1.9541930000	-3.0378070000
H	1.3710870000	-4.4515220000	-2.8979270000
O	-0.8962550000	0.1442170000	-2.5114530000
H	-0.6027800000	1.0417420000	-2.8630240000
H	-1.8548400000	0.2347530000	-2.3817930000

F	-0.8110930000	1.7961700000	-0.1246390000
C	-0.9991620000	3.3943160000	-2.5586710000
O	-0.1135050000	2.6123910000	-2.9763820000
N	-0.7346980000	4.6374780000	-2.1301690000
H	-1.9701800000	3.8997760000	-0.0764640000
C	0.5970570000	5.2613520000	-1.9201220000
C	0.3147090000	6.7163490000	-1.5216790000
C	1.3282790000	4.5395880000	-0.7766400000
C	1.4153100000	5.2254640000	-3.2193900000
H	-0.2196480000	7.2472350000	-2.3176660000
H	-0.2816320000	6.7674100000	-0.6023330000
H	1.2557510000	7.2421320000	-1.3388050000
H	1.5044250000	3.4888720000	-1.0094140000
H	2.2951550000	5.0220300000	-0.5971180000
H	0.7405550000	4.5835020000	0.1460260000
H	2.3680100000	5.7424480000	-3.0646410000
H	1.6173320000	4.1996020000	-3.5297840000
H	0.8764900000	5.7336120000	-4.0259090000
C	-2.4445370000	2.9957460000	-2.6086770000
C	-2.8519010000	2.3459350000	-3.7750350000
C	-3.3911990000	3.2542950000	-1.5566650000
C	-4.1789310000	1.9560910000	-3.9688410000
H	-2.1144070000	2.1661410000	-4.5512310000
C	-4.7408140000	2.8607910000	-1.7996630000
C	-5.1237360000	2.2271680000	-2.9703100000
H	-4.4740080000	1.4670200000	-4.8913960000
H	-5.4838560000	3.0488970000	-1.0320860000
H	-6.1618260000	1.9412710000	-3.1129400000
C	-3.0232860000	3.8093020000	-0.3054260000
C	-3.9980890000	4.1378350000	0.7815450000
H	-4.4153040000	3.2331210000	1.2549430000
H	-3.5148960000	4.7153360000	1.5740110000
H	-4.8553680000	4.7170320000	0.4158800000
H	-1.5369420000	5.1937160000	-1.8664520000

^{3}13

Charge: 1
Multiplicity: 3
B3LYP-D3(BJ)/6-31G(d)-SDD SCF energy (au): -1690.96728770
B3LYP-D3(BJ)/6-31G(d)-SDD enthalpy (au): -1690.37816770
B3LYP-D3(BJ)/6-31G(d)-SDD free energy (au): -1690.49342170
B3LYP-D3(BJ)/def2-TZVP/SMD SCF energy (au): -2831.51638883
B3LYP-D3(BJ)/def2-TZVP/SMD enthalpy (au): -2830.92726883
B3LYP-D3(BJ)/def2-TZVP/SMD free energy (au): -2831.04252283
B3LYP-D3(BJ)/def2-TZVP/SMD free energy (quasi-harmonic) (au): -2831.02950413

Cartesian coordinates

ATOM	X	Y	Z
Fe	-0.1226750000	0.1006100000	-0.5524550000
C	1.2883620000	-0.1837360000	3.6074020000
N	2.2064690000	0.6428840000	2.9840020000
H	2.9663030000	1.1326270000	3.4354290000
C	1.9020350000	0.6999790000	1.6644450000

H	2.4309510000	1.2738050000	0.9188370000
N	0.8411420000	-0.0510010000	1.4128170000
C	0.4444060000	-0.6047760000	2.6144590000
H	-0.4125880000	-1.2582580000	2.6756520000
C	-3.4586050000	-2.1023400000	0.9265000000
N	-3.8662680000	-0.7840320000	1.0193520000
H	-4.7552120000	-0.4617260000	1.3754340000
C	-2.8729570000	0.0094470000	0.5571990000
H	-2.8890090000	1.0872760000	0.5137100000
N	-1.8487080000	-0.7413110000	0.1706560000
C	-2.1978630000	-2.0606300000	0.3954910000
H	-1.5282550000	-2.8679580000	0.1482890000
O	1.3900650000	0.8023900000	-1.2166780000
H	1.1154650000	1.5017410000	-1.8433870000
H	-4.0867170000	-2.9235500000	1.2334500000
H	1.3226030000	-0.3858750000	4.6664120000
C	1.0337480000	-3.6654650000	-2.2244030000
N	1.9129500000	-3.5419440000	-1.1640320000
H	2.6696040000	-4.1738880000	-0.9429340000
C	1.6090180000	-2.4097260000	-0.4876140000
H	2.1426060000	-2.0486530000	0.3761930000
N	0.5752130000	-1.8105830000	-1.0571490000
C	0.2075300000	-2.5775150000	-2.1480170000
H	-0.5931840000	-2.2640840000	-2.7969090000
H	1.0825410000	-4.4884880000	-2.9195250000
O	-1.0927450000	0.0841530000	-2.5585240000
H	-0.7162600000	0.9386650000	-2.9149500000
H	-2.0287010000	0.2963940000	-2.4115310000
F	-0.8732400000	1.7195050000	-0.1609790000
C	-0.9814690000	3.3407200000	-2.5730830000
O	-0.1239730000	2.5287300000	-2.9908460000
N	-0.6732610000	4.5544730000	-2.0917620000
H	-2.0171060000	3.7903290000	-0.1055050000
C	0.6761940000	5.1178280000	-1.8301750000
C	0.4411900000	6.5779540000	-1.4192980000
C	1.3414400000	4.3508300000	-0.6758650000
C	1.5338500000	5.0661070000	-3.1032020000
H	-0.0451680000	7.1413710000	-2.2236240000
H	-0.1818620000	6.6412110000	-0.5185330000
H	1.3966380000	7.0616920000	-1.1988350000
H	1.4888800000	3.2989040000	-0.9219870000
H	2.3170000000	4.7980660000	-0.4551220000
H	0.7232130000	4.4002310000	0.2265020000
H	2.5007080000	5.5423870000	-2.9101850000
H	1.7049280000	4.0372660000	-3.4219200000
H	1.0423110000	5.6064050000	-3.9190020000
C	-2.4433130000	3.0225520000	-2.6898250000
C	-2.8406200000	2.4485700000	-3.8986180000
C	-3.4148390000	3.2927050000	-1.6633810000
C	-4.1803020000	2.1544040000	-4.1614900000
H	-2.0831360000	2.2529610000	-4.6511840000
C	-4.7752820000	2.9984860000	-1.9766210000
C	-5.1476090000	2.4439090000	-3.1900650000

H	-4.4663270000	1.7237130000	-5.1154510000
H	-5.5365190000	3.1991720000	-1.2304480000
H	-6.1947170000	2.2325130000	-3.3862810000
C	-3.0645090000	3.7677050000	-0.3746870000
C	-4.0621650000	4.1105150000	0.6874590000
H	-4.5845940000	3.2196400000	1.0741480000
H	-3.5727440000	4.5891110000	1.5396770000
H	-4.8448750000	4.7885690000	0.3233320000
H	-1.4580550000	5.1374270000	-1.8331840000

[5]14'

Charge: 1
Multiplicity: 5
B3LYP-D3(BJ)/6-31G(d)-SDD SCF energy (au): -1693.88859309
B3LYP-D3(BJ)/6-31G(d)-SDD enthalpy (au): -1693.30724009
B3LYP-D3(BJ)/6-31G(d)-SDD free energy (au): -1693.42526509
B3LYP-D3(BJ)/def2-TZVP/SMD SCF energy (au): -2834.45284941
B3LYP-D3(BJ)/def2-TZVP/SMD enthalpy (au): -2833.87149641
B3LYP-D3(BJ)/def2-TZVP/SMD free energy (au): -2833.98952141
B3LYP-D3(BJ)/def2-TZVP/SMD free energy (quasi-harmonic) (au): -2833.97637932

Cartesian coordinates

ATOM	X	Y	Z
Fe	0.0051450000	-0.0115060000	-0.8166370000
C	-2.2193880000	-3.5365940000	0.4498370000
N	-2.9276340000	-2.5230320000	1.0692790000
H	-3.6971170000	-2.6451140000	1.7128150000
C	-2.4001250000	-1.3377960000	0.6795120000
H	-2.7522850000	-0.3745060000	1.0145290000
N	-1.3932570000	-1.5379060000	-0.1575920000
C	-1.2686400000	-2.9077740000	-0.3075740000
H	-0.5037350000	-3.3315550000	-0.9400550000
H	-2.4534650000	-4.5780160000	0.6042540000
C	3.0089260000	-2.9135960000	-2.0683470000
N	3.3932070000	-2.6285760000	-0.7719480000
H	4.1793520000	-3.0337410000	-0.2831700000
C	2.5381390000	-1.7039130000	-0.2680970000
H	2.5872570000	-1.2934390000	0.7291740000
N	1.6288280000	-1.3847350000	-1.1761780000
C	1.9092940000	-2.1314620000	-2.3059670000
H	1.3022190000	-2.0432390000	-3.1940800000
H	3.5413090000	-3.6219720000	-2.6832720000
O	-0.8574530000	-0.2712550000	-2.7498470000
H	-0.9159810000	0.5652480000	-3.3066870000
H	-1.7589820000	-0.6186940000	-2.6636850000
F	-1.7389280000	1.4661110000	-0.4674080000
C	-1.8612520000	2.7657390000	-4.2526910000
O	-0.9455910000	1.9239410000	-4.1900600000
N	-1.7890740000	3.8644260000	-5.0217880000
H	-2.5613030000	4.5141590000	-4.9543380000
C	-0.6314490000	4.2736680000	-5.8642090000
C	-1.0429620000	5.5876730000	-6.5388880000
C	0.5961150000	4.4959620000	-4.9656560000

C	-0.3567960000	3.1971350000	-6.9246420000
H	-1.9262110000	5.4483750000	-7.1730270000
H	-1.2601080000	6.3650600000	-5.7964660000
H	-0.2289750000	5.9504890000	-7.1722370000
H	0.8607450000	3.5794900000	-4.4337390000
H	1.4497990000	4.8069390000	-5.5764700000
H	0.3976150000	5.2835940000	-4.2306150000
H	0.4636630000	3.5223250000	-7.5723370000
H	-0.0786160000	2.2508510000	-6.4573630000
H	-1.2422350000	3.0365260000	-7.5488250000
C	-3.1410060000	2.6061630000	-3.4783460000
C	-4.3462620000	2.5168350000	-4.1880200000
C	-3.1432650000	2.5099060000	-2.0748180000
C	-5.5480230000	2.2986460000	-3.5176600000
H	-4.3320920000	2.5924590000	-5.2714080000
C	-4.3599710000	2.2987310000	-1.4157680000
C	-5.5528590000	2.1838050000	-2.1263480000
H	-6.4740840000	2.2165730000	-4.0778910000
H	-4.3689950000	2.2293550000	-0.3322150000
H	-6.4853210000	2.0157440000	-1.5963840000
C	-1.8829170000	2.6771170000	-1.2675470000
C	-1.8730880000	3.8376130000	-0.2968560000
H	-2.7239890000	3.7930000000	0.3897030000
H	-0.9396030000	3.8282270000	0.2726090000
H	-1.9351670000	4.7772680000	-0.8562140000
H	-0.9970880000	2.6888750000	-1.8971520000
C	0.9857960000	1.6426650000	1.4443940000
O	1.2202660000	2.6189910000	0.6956540000
O	0.6450900000	0.4671040000	1.0315660000
C	1.0813820000	1.8135150000	2.9462210000
H	0.0745360000	1.7644430000	3.3770870000
H	1.6581180000	0.9926860000	3.3828590000
H	1.5350350000	2.7724770000	3.1997000000
O	1.0244640000	1.6974560000	-1.6922530000
H	1.1999150000	2.1753300000	-0.8042720000
H	1.8866510000	1.4522440000	-2.0623800000

^{3}14'

Charge: 1
Multiplicity: 3
B3LYP-D3(BJ)/6-31G(d)-SDD SCF energy (au): -1693.86284553
B3LYP-D3(BJ)/6-31G(d)-SDD enthalpy (au): -1693.27937853
B3LYP-D3(BJ)/6-31G(d)-SDD free energy (au): -1693.39012353
B3LYP-D3(BJ)/def2-TZVP/SMD SCF energy (au): -2834.42976954
B3LYP-D3(BJ)/def2-TZVP/SMD enthalpy (au): -2833.84630254
B3LYP-D3(BJ)/def2-TZVP/SMD free energy (au): -2833.95704754
B3LYP-D3(BJ)/def2-TZVP/SMD free energy (quasi-harmonic) (au): -2833.94738301

Cartesian coordinates

ATOM	X	Y	Z
Fe	0.0454930000	-0.3201330000	-1.6373750000
C	-2.8420940000	-2.5568880000	0.3946550000
N	-2.8621220000	-1.3544810000	1.0757620000

H	-3.4646070000	-1.1228550000	1.8528940000
C	-1.9213380000	-0.5375120000	0.5445570000
H	-1.7218530000	0.4763260000	0.8520050000
N	-1.2985280000	-1.1623840000	-0.4477360000
C	-1.8650190000	-2.4235580000	-0.5550060000
H	-1.5193000000	-3.1321640000	-1.2923860000
H	-3.4982410000	-3.3748410000	0.6467870000
C	3.2953800000	-3.0764710000	-1.8443160000
N	2.6799770000	-3.4964220000	-0.6786170000
H	3.0087110000	-4.2288470000	-0.0650760000
C	1.5707620000	-2.7438710000	-0.4899540000
H	0.9013370000	-2.8491570000	0.3496660000
N	1.4432390000	-1.8660370000	-1.4753690000
C	2.5165640000	-2.0608230000	-2.3269760000
H	2.6445760000	-1.4421650000	-3.2004330000
H	4.2055320000	-3.5269970000	-2.2071730000
O	-1.1490930000	-0.8051760000	-3.2497240000
H	-1.1481090000	-0.0309370000	-3.8801680000
H	-2.0768500000	-0.9317440000	-2.9904470000
F	-2.0449650000	2.7498050000	0.3554560000
C	-1.6349890000	2.3867480000	-3.9057510000
O	-0.9198020000	1.5412960000	-4.4973970000
N	-1.4269460000	3.7045040000	-3.9787550000
H	-2.1506570000	4.2863920000	-3.5791900000
C	-0.3858720000	4.4231030000	-4.7736270000
C	-0.5524410000	5.9048210000	-4.4126140000
C	1.0176800000	3.9453520000	-4.3772530000
C	-0.6496260000	4.1942150000	-6.2690260000
H	-1.5601560000	6.2646240000	-4.6553140000
H	-0.3609480000	6.0753780000	-3.3471110000
H	0.1590440000	6.5069560000	-4.9833910000
H	1.2080440000	2.9323800000	-4.7362050000
H	1.7594320000	4.6047680000	-4.8387710000
H	1.1579720000	3.9703630000	-3.2932130000
H	0.0994430000	4.7286830000	-6.8617670000
H	-0.5876890000	3.1308750000	-6.5131820000
H	-1.6398570000	4.5656540000	-6.5537860000
C	-2.8052650000	1.9159860000	-3.0888900000
C	-3.6613160000	0.9969020000	-3.7208480000
C	-3.0229510000	2.2640920000	-1.7432470000
C	-4.7270130000	0.4245520000	-3.0347450000
H	-3.4787490000	0.7421980000	-4.7606220000
C	-4.0956190000	1.6685970000	-1.0635180000
C	-4.9392000000	0.7598370000	-1.6940450000
H	-5.3895060000	-0.2706690000	-3.5407520000
H	-4.2495020000	1.9187740000	-0.0197230000
H	-5.7649940000	0.3171050000	-1.1455290000
C	-2.1494550000	3.2320920000	-0.9699230000
C	-2.7337300000	4.6348500000	-0.8623580000
H	-3.7284580000	4.5959330000	-0.4085110000
H	-2.0841080000	5.2598010000	-0.2430840000
H	-2.8317170000	5.1124880000	-1.8423240000
H	-1.1344200000	3.2571200000	-1.3709980000

C	0.9691570000	2.1154460000	-0.2452330000
O	1.4464400000	2.6177430000	-1.2885120000
O	0.3219280000	0.9987040000	-0.1999880000
C	1.1040820000	2.8439070000	1.0741330000
H	0.1082890000	3.1467340000	1.4118590000
H	1.5171520000	2.1691320000	1.8302010000
H	1.7436240000	3.7207900000	0.9661060000
O	1.2440510000	0.6596600000	-2.9262520000
H	1.4513300000	1.4956910000	-2.3778600000
H	0.7154450000	0.9902700000	-3.6802460000

^{5}14

Charge: 1
Multiplicity: 5
B3LYP-D3(BJ)/6-31G(d)-SDD SCF energy (au): -1691.01125007
B3LYP-D3(BJ)/6-31G(d)-SDD enthalpy (au): -1690.42029407
B3LYP-D3(BJ)/6-31G(d)-SDD free energy (au): -1690.53443707
B3LYP-D3(BJ)/def2-TZVP/SMD SCF energy (au): -2831.56803864
B3LYP-D3(BJ)/def2-TZVP/SMD enthalpy (au): -2830.97708264
B3LYP-D3(BJ)/def2-TZVP/SMD free energy (au): -2831.09122564
B3LYP-D3(BJ)/def2-TZVP/SMD free energy (quasi-harmonic) (au): -2831.08057757

Cartesian coordinates

ATOM	X	Y	Z
Fe	-0.1607970000	0.2837470000	-1.3770050000
C	0.7973150000	1.4659940000	2.8027270000
N	1.1949110000	2.5348920000	2.0210340000
H	1.5709500000	3.4094200000	2.3589400000
C	0.9870220000	2.2121910000	0.7193890000
H	1.1859340000	2.8465970000	-0.1316020000
N	0.4787050000	0.9934650000	0.6276270000
C	0.3528390000	0.5169720000	1.9194050000
H	-0.0474470000	-0.4659770000	2.1192120000
C	-3.5953420000	-1.9362440000	0.1232020000
N	-3.7308270000	-0.7307070000	0.7878210000
H	-4.4647250000	-0.4925740000	1.4399190000
C	-2.7161100000	0.0835690000	0.4089390000
H	-2.5870440000	1.1017280000	0.7385090000
N	-1.9375660000	-0.5383440000	-0.4633700000
C	-2.4739830000	-1.8016490000	-0.6500520000
H	-2.0058340000	-2.5166570000	-1.3096530000
O	0.3657200000	1.9398190000	-2.1173980000
H	0.5832370000	1.8823660000	-3.0588310000
H	-4.2828020000	-2.7544780000	0.2683250000
H	0.8693200000	1.4813440000	3.8789430000
C	2.3279150000	-3.1932160000	-2.0314180000
N	2.8803500000	-2.6754650000	-0.8735150000
H	3.6888190000	-3.0382190000	-0.3881340000
C	2.1607600000	-1.5870230000	-0.5103240000
H	2.3691470000	-0.9791040000	0.3565430000
N	1.1764730000	-1.3821910000	-1.3735340000
C	1.2700830000	-2.3784110000	-2.3305160000
H	0.5741710000	-2.4131140000	-3.1536340000

H	2.7330810000	-4.0650120000	-2.5203150000
O	-1.0713750000	-0.6104330000	-3.2694520000
H	-0.9496570000	0.1020850000	-3.9556490000
H	-2.0285010000	-0.5939410000	-3.1051270000
F	-2.0980730000	3.0829650000	-0.1501190000
C	-1.6552430000	2.4456680000	-4.3368390000
O	-0.9247790000	1.5745760000	-4.8470130000
N	-1.4695360000	3.7642760000	-4.5217170000
H	-2.2119290000	4.3690790000	-4.1993480000
C	-0.4048300000	4.4092440000	-5.3352400000
C	-0.5633360000	5.9173910000	-5.1035600000
C	0.9799780000	3.9496260000	-4.8550350000
C	-0.6138820000	4.0645120000	-6.8176460000
H	-1.5573760000	6.2652150000	-5.4113770000
H	-0.4118210000	6.1723660000	-4.0482850000
H	0.1757930000	6.4652640000	-5.6941700000
H	1.1492420000	2.8996990000	-5.1013670000
H	1.7513060000	4.5468620000	-5.3517930000
H	1.0797130000	4.0783400000	-3.7729480000
H	0.1615580000	4.5419410000	-7.4256940000
H	-0.5580950000	2.9840030000	-6.9695330000
H	-1.5895480000	4.4202610000	-7.1660230000
C	-2.8492450000	2.0585690000	-3.5079180000
C	-3.7002100000	1.0941470000	-4.0719250000
C	-3.1206740000	2.5690160000	-2.2253270000
C	-4.8282020000	0.6499560000	-3.3886280000
H	-3.4647080000	0.7051950000	-5.0579490000
C	-4.2672430000	2.1181030000	-1.5568900000
C	-5.1157210000	1.1728690000	-2.1251270000
H	-5.4838500000	-0.0870790000	-3.8418240000
H	-4.4753360000	2.5052280000	-0.5646950000
H	-5.9981830000	0.8415990000	-1.5858440000
C	-2.1988860000	3.5289350000	-1.5011280000
C	-2.7101470000	4.9597630000	-1.4378010000
H	-3.7083140000	4.9974320000	-0.9900690000
H	-2.0309110000	5.5735120000	-0.8386010000
H	-2.7740270000	5.4037080000	-2.4366540000
H	-1.1877230000	3.4519030000	-1.9015800000

^{3}14

Charge: 1
Multiplicity: 3
B3LYP-D3(BJ)/6-31G(d)-SDD SCF energy (au): -1690.98300497
B3LYP-D3(BJ)/6-31G(d)-SDD enthalpy (au): -1690.39092497
B3LYP-D3(BJ)/6-31G(d)-SDD free energy (au): -1690.50190697
B3LYP-D3(BJ)/def2-TZVP/SMD SCF energy (au): -2831.54336111
B3LYP-D3(BJ)/def2-TZVP/SMD enthalpy (au): -2830.95128111
B3LYP-D3(BJ)/def2-TZVP/SMD free energy (au): -2831.06226311
B3LYP-D3(BJ)/def2-TZVP/SMD free energy (quasi-harmonic) (au): -2831.05217414

Cartesian coordinates

ATOM	X	Y	Z
Fe	-0.3246680000	0.3578660000	-1.2683070000

C	0.7671980000	1.5300930000	2.6064100000
N	1.2239090000	2.5710460000	1.8191550000
H	1.6988450000	3.3994190000	2.1479550000
C	0.9274370000	2.2965690000	0.5259780000
H	1.1596750000	2.9050080000	-0.3342450000
N	0.3004290000	1.1315350000	0.4476640000
C	0.1925850000	0.6408220000	1.7379640000
H	-0.2824710000	-0.3060780000	1.9407260000
C	-3.3045630000	-2.2594950000	0.2755910000
N	-3.7155270000	-1.0367230000	0.7753310000
H	-4.5347960000	-0.8737080000	1.3434400000
C	-2.8346480000	-0.0901350000	0.3699960000
H	-2.9069840000	0.9626830000	0.5915510000
N	-1.8823130000	-0.6466420000	-0.3663420000
C	-2.1627790000	-2.0010780000	-0.4339370000
H	-1.5143240000	-2.6756760000	-0.9711380000
O	0.4115320000	1.8521820000	-2.1223180000
H	0.5374870000	1.7072080000	-3.0711280000
H	-3.8444320000	-3.1721580000	0.4724190000
H	0.8900470000	1.5196060000	3.6778060000
C	2.2821850000	-3.0939360000	-2.0135910000
N	2.8484710000	-2.5614160000	-0.8702440000
H	3.6833780000	-2.8958200000	-0.4102100000
C	2.0974040000	-1.4978790000	-0.4856750000
H	2.3135090000	-0.8862300000	0.3772020000
N	1.0827510000	-1.3252210000	-1.3161470000
C	1.1873610000	-2.3141980000	-2.2770010000
H	0.4766820000	-2.3696820000	-3.0863280000
H	2.7049330000	-3.9481690000	-2.5187020000
O	-1.0745490000	-0.4182160000	-3.0466390000
H	-0.9729480000	0.2436460000	-3.7896090000
H	-2.0355930000	-0.4967850000	-2.9322890000
F	-2.2149460000	2.9663730000	-0.1431940000
C	-1.6651880000	2.4709920000	-4.3319350000
O	-0.9293900000	1.5836220000	-4.8080490000
N	-1.4497530000	3.7843740000	-4.5072690000
H	-2.1975550000	4.4000000000	-4.2185220000
C	-0.3428030000	4.4137340000	-5.2772080000
C	-0.4687300000	5.9212620000	-5.0221990000
C	1.0151520000	3.9083070000	-4.7673870000
C	-0.5200140000	4.0996450000	-6.7704780000
H	-1.4460500000	6.3006140000	-5.3462640000
H	-0.3350160000	6.1537070000	-3.9593900000
H	0.2976390000	6.4597450000	-5.5860600000
H	1.1783720000	2.8682420000	-5.0543310000
H	1.8123320000	4.5158050000	-5.2078430000
H	1.0759340000	3.9802910000	-3.6776020000
H	0.2871940000	4.5618570000	-7.3481380000
H	-0.4918080000	3.0202400000	-6.9386050000
H	-1.4737210000	4.4904550000	-7.1415470000
C	-2.8989160000	2.1022000000	-3.5552030000
C	-3.7652580000	1.1869190000	-4.1737050000
C	-3.1869770000	2.5805370000	-2.2644250000

C	-4.9331760000	0.7719490000	-3.5402780000
H	-3.5108490000	0.8136210000	-5.1609960000
C	-4.3724530000	2.1601040000	-1.6457850000
C	-5.2417850000	1.2714420000	-2.2721710000
H	-5.6022520000	0.0750280000	-4.0352820000
H	-4.5975490000	2.5257160000	-0.6490560000
H	-6.1565720000	0.9651620000	-1.7733680000
C	-2.2377940000	3.4632330000	-1.4810100000
C	-2.6629500000	4.9196820000	-1.3869150000
H	-3.6747440000	5.0050970000	-0.9781680000
H	-1.9729100000	5.4694610000	-0.7398280000
H	-2.6547460000	5.3971970000	-2.3718400000
H	-1.2174360000	3.3405170000	-1.8499450000

F□

Charge: 0
Multiplicity: 2
B3LYP-D3(BJ)/6-31G(d)-SDD SCF energy (au): -99.7145779299
B3LYP-D3(BJ)/6-31G(d)-SDD enthalpy (au): -99.7122179299
B3LYP-D3(BJ)/6-31G(d)-SDD free energy (au): -99.7293909299
B3LYP-D3(BJ)/def2-TZVP/SMD SCF energy (au): -99.7699421456
B3LYP-D3(BJ)/def2-TZVP/SMD enthalpy (au): -99.7675821456
B3LYP-D3(BJ)/def2-TZVP/SMD free energy (au): -99.7847551456
B3LYP-D3(BJ)/def2-TZVP/SMD free energy (quasi-harmonic) (au): -99.7847552157

Cartesian coordinates

ATOM	X	Y	Z
F	-0.0987430000	0.1885100000	0.0000000000

^{5}Im$_2$AcOWat$_2$_FeF_remove-F

Charge: 1
Multiplicity: 5
B3LYP-D3(BJ)/6-31G(d)-SDD SCF energy (au): -957.706375357
B3LYP-D3(BJ)/6-31G(d)-SDD enthalpy (au): -957.435398357
B3LYP-D3(BJ)/6-31G(d)-SDD free energy (au): -957.513740357
B3LYP-D3(BJ)/def2-TZVP/SMD SCF energy (au): -2097.97855970
B3LYP-D3(BJ)/def2-TZVP/SMD enthalpy (au): -2097.70758270
B3LYP-D3(BJ)/def2-TZVP/SMD free energy (au): -2097.78592470
B3LYP-D3(BJ)/def2-TZVP/SMD free energy (quasi-harmonic) (au): -2097.778447343

Cartesian coordinates

ATOM	X	Y	Z
Fe	-0.2809980000	-1.2035720000	-0.4138570000
C	-0.7585230000	-1.4340070000	3.8928220000
N	0.2520610000	-0.5067370000	3.7238310000
H	0.7527390000	-0.0340860000	4.4642850000
C	0.4608910000	-0.3357460000	2.3988680000
H	1.2003750000	0.3202500000	1.9652210000
N	-0.3674220000	-1.1097500000	1.7064310000
C	-1.1332190000	-1.8022700000	2.6295460000
H	-1.8856980000	-2.5121010000	2.3210440000
H	-1.1048390000	-1.7375940000	4.8680780000
C	0.7917530000	-5.2893520000	-1.3199320000

N	2.0163500000	-4.6533700000	-1.2773900000
H	2.9159930000	-5.0879990000	-1.4326010000
C	1.8106710000	-3.3455610000	-0.9969080000
H	2.5923570000	-2.6065570000	-0.9052130000
N	0.5116280000	-3.1110240000	-0.8579180000
C	-0.1365130000	-4.3182670000	-1.0578350000
H	-1.2111870000	-4.4010470000	-0.9961810000
H	0.6962950000	-6.3435000000	-1.5268630000
O	-2.4448340000	-1.1088610000	-0.3920100000
H	-2.5825700000	-0.7951300000	-1.3087550000
H	-2.9132390000	-0.5063540000	0.2090970000
C	1.6666340000	0.7127780000	-1.5390390000
O	1.0238240000	0.8100530000	-2.6008410000
O	1.3299570000	-0.0607950000	-0.5446560000
C	2.9195130000	1.5326430000	-1.3314760000
H	2.7418750000	2.2698270000	-0.5401410000
H	3.7395650000	0.8896670000	-0.9968980000
H	3.1957020000	2.0506970000	-2.2501560000
O	-0.9064690000	-0.8450350000	-2.4849470000
H	-0.1892750000	-0.1270500000	-2.6722630000
H	-0.7357590000	-1.5748630000	-3.1019030000

^{6}Im$_2$AcOWat$_2$_FeF
Charge: 1
Multiplicity: 6
B3LYP-D3(BJ)/6-31G(d)-SDD SCF energy (au): -1057.56353877
B3LYP-D3(BJ)/6-31G(d)-SDD enthalpy (au): -1057.28793377
B3LYP-D3(BJ)/6-31G(d)-SDD free energy (au): -1057.36405277
B3LYP-D3(BJ)/def2-TZVP/SMD SCF energy (au): -2197.88044536
B3LYP-D3(BJ)/def2-TZVP/SMD enthalpy (au): -2197.60484036
B3LYP-D3(BJ)/def2-TZVP/SMD free energy (au): -2197.68095936
B3LYP-D3(BJ)/def2-TZVP/SMD free energy (quasi-harmonic) (au): -2197.67730759

Cartesian coordinates

ATOM	X	Y	Z
Fe	-0.3190570000	0.2412120000	-0.5632920000
C	-3.6684990000	-2.0299570000	0.8986390000
N	-4.2245340000	-0.8077070000	0.5650270000
H	-5.1882350000	-0.5353070000	0.7069260000
C	-3.2643710000	-0.0287510000	0.0281360000
H	-3.4001070000	0.9877020000	-0.3071010000
N	-2.1147260000	-0.6977390000	-0.0058960000
C	-2.3524440000	-1.9474980000	0.5401050000
H	-1.5679730000	-2.6806270000	0.6344520000
H	-4.2459530000	-2.8214910000	1.3492190000
C	1.0998000000	-3.7481230000	-1.5065920000
N	2.1300920000	-3.2700390000	-0.7179830000
H	2.9766510000	-3.7696320000	-0.4792840000
C	1.8298300000	-2.0132080000	-0.3284840000
H	2.4682130000	-1.3872690000	0.2782130000
N	0.6499040000	-1.6588440000	-0.8300780000
C	0.1845980000	-2.7346190000	-1.5716490000
H	-0.7598170000	-2.6908830000	-2.0902090000

H	1.1147010000	-4.7366520000	-1.9375890000
O	-0.6940040000	-0.0928780000	-2.7405880000
H	0.1765710000	-0.0360940000	-3.1736250000
H	-1.1546600000	0.7390980000	-2.9559110000
F	-1.2058640000	1.7880880000	-0.9451810000
C	1.5094970000	0.7042900000	1.7251490000
O	2.5289490000	0.7746760000	1.0117640000
O	0.3170370000	0.4776340000	1.2424770000
C	1.5791500000	0.8690340000	3.2201570000
H	0.9792460000	1.7370360000	3.5139070000
H	1.1408830000	-0.0057780000	3.7111250000
H	2.6119970000	1.0068230000	3.5408130000
O	1.4835330000	1.1164630000	-1.3247840000
H	2.0797330000	1.0160990000	-0.5037240000
H	1.3144140000	2.0720510000	-1.4127240000

$^5Im_2AcOWatN_3$_11

Charge: 0
Multiplicity: 5
B3LYP-D3(BJ)/6-31G(d)-SDD SCF energy (au): -1781.73725776
B3LYP-D3(BJ)/6-31G(d)-SDD enthalpy (au): -1781.16923076
B3LYP-D3(BJ)/6-31G(d)-SDD free energy (au): -1781.29071876
B3LYP-D3(BJ)/def2-TZVP/SMD SCF energy (au): -2922.29646504
B3LYP-D3(BJ)/def2-TZVP/SMD enthalpy (au): -2921.72843804
B3LYP-D3(BJ)/def2-TZVP/SMD free energy (au): -2921.84992604
B3LYP-D3(BJ)/def2-TZVP/SMD free energy (quasi-harmonic) (au): -2921.83704328

Cartesian coordinates

ATOM	X	Y	Z
Fe	0.6756350000	-1.2910560000	-1.4525910000
C	1.7152430000	1.7022870000	1.5824320000
N	2.3579000000	2.2332060000	0.4752900000
H	2.9805660000	3.0272790000	0.4700380000
C	2.0784470000	1.4411480000	-0.5925320000
H	2.4478750000	1.6116080000	-1.5907900000
N	1.2835940000	0.4503790000	-0.2301840000
C	1.0509840000	0.5962920000	1.1225550000
H	0.4062900000	-0.0965540000	1.6396630000
H	1.7898050000	2.1543850000	2.5590230000
C	1.5790280000	-4.6032210000	1.2469260000
N	2.8026930000	-3.9561120000	1.2260140000
H	3.6482360000	-4.2628270000	1.6832880000
C	2.6805740000	-2.8449120000	0.4520070000
H	3.4926190000	-2.1635730000	0.2340930000
N	1.4476450000	-2.7526830000	-0.0162330000
C	0.7488970000	-3.8411510000	0.4689380000
H	-0.2863860000	-3.9973690000	0.2039560000
H	1.4238230000	-5.5199790000	1.7935840000
O	-0.2268420000	-2.8497240000	-2.6773420000
H	-0.4501540000	-2.3906840000	-3.5019590000
H	-1.0956250000	-2.9474530000	-2.1644450000
C	-4.5664240000	0.2305080000	-2.0786470000
C	-3.2862780000	0.2869340000	-2.6155320000

```
C     -2.3160180000    1.1326600000   -2.0579460000
C     -2.5867920000    1.8700780000   -0.8895060000
C     -3.8853410000    1.7955930000   -0.3676440000
C     -4.8705090000    1.0078560000   -0.9584130000
H     -5.3145930000   -0.4201980000   -2.5197280000
H     -3.0226120000   -0.3158920000   -3.4785650000
H     -4.1148870000    2.3472550000    0.5407850000
H     -5.8658360000    0.9724070000   -0.5248320000
C     -1.5301070000    2.6318620000   -0.1148960000
C     -1.7181360000    4.1543970000   -0.1048340000
H     -0.5375400000    2.3913150000   -0.4930950000
H     -1.5552750000    2.2614060000    0.9168520000
H     -0.9969880000    4.6227360000    0.5742210000
H     -1.5592790000    4.5760410000   -1.1019490000
H     -2.7240660000    4.4377570000    0.2251740000
C     -0.9968860000    1.1567840000   -2.7413740000
O     -0.3501120000    0.1468710000   -2.9871230000
N     -0.5060710000    2.4427320000   -3.0449400000
C     -1.1226610000    3.2942370000   -4.1289880000
C     -2.5996690000    3.5760840000   -3.8228530000
C     -0.9911520000    2.5679340000   -5.4768030000
H     -2.9513350000    4.3059580000   -4.5592110000
H     -3.2232070000    2.6853530000   -3.9064940000
H     -2.7384320000    4.0008230000   -2.8272230000
H      0.0512890000    2.3224240000   -5.6886900000
H     -1.5718610000    1.6392070000   -5.4759490000
H     -1.3718370000    3.2066010000   -6.2802870000
F      0.8565790000    2.2865510000   -3.4221590000
C     -0.3457100000    4.6180860000   -4.1316490000
H     -0.4252920000    5.1113350000   -3.1576480000
H      0.7101550000    4.4575330000   -4.3555080000
H     -0.7653190000    5.2834580000   -4.8924920000
C     -2.0920580000   -1.9266290000   -0.3074930000
O     -2.3054120000   -2.8740850000   -1.1030390000
O     -1.0701250000   -1.1574730000   -0.3190400000
C     -3.0949430000   -1.6732730000    0.8089660000
H     -4.0548500000   -2.1419780000    0.5829930000
H     -3.2239530000   -0.6003310000    0.9663290000
H     -2.7025950000   -2.1051770000    1.7382050000
N      2.4307990000   -1.1357920000   -2.5236190000
N      3.4623450000   -0.8991260000   -1.9543990000
N      4.4615730000   -0.6578890000   -1.4022410000
```

$^{5}Im_2AcOWatN_3$_13

Charge: 0
Multiplicity: 5
B3LYP-D3(BJ)/6-31G(d)-SDD SCF energy (au):   -1781.80719873
B3LYP-D3(BJ)/6-31G(d)-SDD enthalpy (au):   -1781.24039373
B3LYP-D3(BJ)/6-31G(d)-SDD free energy (au):   -1781.35941773
B3LYP-D3(BJ)/def2-TZVP/SMD SCF energy (au):   -2922.36776995
B3LYP-D3(BJ)/def2-TZVP/SMD enthalpy (au):   -2921.80096495
B3LYP-D3(BJ)/def2-TZVP/SMD free energy (au):   -2921.91998895
B3LYP-D3(BJ)/def2-TZVP/SMD free energy (quasi-harmonic) (au): -2921.90842522

Cartesian coordinates

ATOM	X	Y	Z
H	35.7177770000	28.1774830000	39.2753610000
C	35.2410310000	28.7404770000	38.4886380000
C	35.3300980000	28.6769800000	37.1242000000
N	34.3419480000	29.7594130000	38.7515440000
C	33.9219390000	30.2688630000	37.5677010000
N	34.5038000000	29.6320580000	36.5665950000
H	34.0333990000	30.0642150000	39.6626890000
H	35.9265880000	28.0267310000	36.5041140000
H	33.2221460000	31.0815910000	37.4615360000
H	37.1577050000	25.8546840000	33.0025250000
C	36.3474030000	26.5049160000	33.2924100000
C	36.3134060000	27.8141990000	33.6921820000
N	35.0295320000	26.0770990000	33.3071430000
C	34.2521740000	27.1149150000	33.7062700000
N	35.0056380000	28.1715400000	33.9492000000
H	34.6984380000	25.1580670000	33.0528890000
H	37.1023820000	28.5391650000	33.8129490000
H	33.1770160000	27.0936870000	33.8006120000
Fe	34.2191720000	30.1581100000	34.3990440000
O	33.6691210000	31.9942750000	35.2199970000
H	34.5693230000	32.3288940000	35.5494710000
H	33.3473600000	32.6185210000	34.5111980000
F	34.1264680000	30.5672830000	32.6184590000
N	33.8382080000	33.4331710000	31.0541420000
O	32.9634870000	33.5545430000	33.1512380000
C	35.3176790000	33.8952600000	32.9152830000
C	35.3589710000	34.8982330000	33.8821790000
C	36.5689170000	35.3697970000	34.3912050000
C	37.7677070000	34.8208310000	33.9211850000
C	37.7505580000	33.7995450000	32.9862790000
C	36.5322250000	33.2714970000	32.4673650000
C	33.9500950000	33.5768750000	32.3890320000
C	32.5939260000	33.1468170000	30.3044820000
C	31.9312630000	31.8598550000	30.8200140000
C	31.6364130000	34.3427380000	30.4357070000
C	33.0192500000	32.9664250000	28.8413760000
C	36.5307440000	32.1293600000	31.6316030000
C	37.7658630000	31.4024210000	31.2085340000
H	34.4179190000	35.3177420000	34.2226940000
H	36.5769570000	36.1540560000	35.1413810000
H	38.7184210000	35.1822750000	34.3040470000
H	38.6881860000	33.3568930000	32.6681720000
H	34.6971490000	33.4832600000	30.5238110000
H	35.5878540000	31.6210660000	31.4766690000
H	38.1581570000	30.7926770000	32.0391560000
H	37.5481050000	30.7152520000	30.3846940000
H	38.5744730000	32.0708080000	30.8881380000
H	31.0538220000	31.6340630000	30.2025520000
H	32.6290760000	31.0211470000	30.7809310000
H	31.6157440000	31.9750370000	31.8573580000

H	31.3743430000	34.5005440000	31.4839740000
H	32.1010250000	35.2559540000	30.0466830000
H	30.7185030000	34.1525930000	29.8681490000
H	33.7058880000	32.1180150000	28.7360750000
H	32.1417650000	32.7706780000	28.2180400000
H	33.5125080000	33.8678640000	28.4563830000
N	32.3683800000	29.3772920000	34.5473980000
N	31.7316100000	29.3115600000	35.5733290000
N	31.1022940000	29.2372160000	36.5412160000
C	36.7320270000	31.5440150000	35.3594000000
O	36.1045010000	32.4136080000	35.9975700000
O	36.2067910000	30.6675780000	34.5749280000
C	38.2406450000	31.4478640000	35.5185510000
H	38.7161070000	31.2728020000	34.5496440000
H	38.4732970000	30.5924790000	36.1651510000
H	38.6373100000	32.3576040000	35.9718570000

$^5Im_2AcOWatN_3$_FeF_remove-F

Charge: 0
Multiplicity: 5
B3LYP-D3(BJ)/6-31G(d)-SDD SCF energy (au): -1045.67205188
B3LYP-D3(BJ)/6-31G(d)-SDD enthalpy (au): -1045.41223888
B3LYP-D3(BJ)/6-31G(d)-SDD free energy (au): -1045.49315488
B3LYP-D3(BJ)/def2-TZVP/SMD SCF energy (au): -2185.93257811
B3LYP-D3(BJ)/def2-TZVP/SMD enthalpy (au): -2185.67276511
B3LYP-D3(BJ)/def2-TZVP/SMD free energy (au): -2185.75368111
B3LYP-D3(BJ)/def2-TZVP/SMD free energy (quasi-harmonic) (au): -2185.74648723

Cartesian coordinates

ATOM	X	Y	Z
Fe	0.4551210000	-0.8536570000	-0.8867460000
C	1.0781460000	1.6595090000	2.6632990000
N	2.1001530000	2.1481830000	1.8690370000
H	2.7222240000	2.9099870000	2.0966060000
C	2.0955950000	1.4577330000	0.6984240000
H	2.7748050000	1.6552300000	-0.1201810000
N	1.1283840000	0.5520860000	0.7078420000
C	0.4845530000	0.6690420000	1.9274300000
H	-0.3693840000	0.0487860000	2.1497860000
H	0.8719760000	2.0586410000	3.6439120000
C	2.6344130000	-4.4803100000	-0.0372150000
N	3.0900070000	-3.6703290000	0.9900560000
H	3.7577060000	-3.9307620000	1.7008770000
C	2.4861290000	-2.4615550000	0.8713190000
H	2.6500060000	-1.6291280000	1.5383050000
N	1.6735480000	-2.4586860000	-0.1715280000
C	1.7542640000	-3.7118000000	-0.7496960000
H	1.1678020000	-3.9400550000	-1.6259790000
H	2.9731200000	-5.4971980000	-0.1570440000
O	-0.3167420000	-2.2106700000	-2.4898620000
H	-0.1196110000	-1.6249460000	-3.2404270000
H	-1.2936220000	-2.0905940000	-2.2714700000
C	-2.4658650000	-1.2846190000	-0.3748670000

O	-2.7002200000	-1.7605480000	-1.5008070000
O	-1.3004080000	-0.9356370000	0.0671130000
C	-3.6078520000	-1.0715920000	0.6058110000
H	-3.6676990000	-0.0109580000	0.8734800000
H	-3.4095330000	-1.6285770000	1.5281560000
H	-4.5550840000	-1.3976780000	0.1733810000
N	1.3398670000	0.0974900000	-2.4294420000
N	2.2883090000	0.8410230000	-2.4062850000
N	3.1996490000	1.5605970000	-2.4046810000

^{5}Im$_2$AcOWatN$_3$_FeF_remove-N$_3$

Charge: 0
Multiplicity: 5
B3LYP-D3(BJ)/6-31G(d)-SDD SCF energy (au): -981.331328730
B3LYP-D3(BJ)/6-31G(d)-SDD enthalpy (au): -981.085036730
B3LYP-D3(BJ)/6-31G(d)-SDD free energy (au): -981.161505730
B3LYP-D3(BJ)/def2-TZVP/SMD SCF energy (au): -2121.57381139
B3LYP-D3(BJ)/def2-TZVP/SMD enthalpy (au): -2121.32751939
B3LYP-D3(BJ)/def2-TZVP/SMD free energy (au): -2121.40398839
B3LYP-D3(BJ)/def2-TZVP/SMD free energy (quasi-harmonic) (au): -2121.397489660

Cartesian coordinates

ATOM	X	Y	Z
Fe	0.2661520000	-0.6255950000	-0.8586760000
C	1.1482660000	1.8995050000	2.6817740000
N	1.8371070000	2.5061720000	1.6452630000
H	2.3600120000	3.3675290000	1.7021380000
C	1.6547640000	1.7590240000	0.5237640000
H	2.0441030000	1.9861130000	-0.4574480000
N	0.8965630000	0.7115310000	0.7930920000
C	0.5681900000	0.7860750000	2.1308460000
H	-0.0801420000	0.0493700000	2.5804540000
H	1.1307430000	2.3124900000	3.6782620000
C	2.5348640000	-4.2719110000	-0.3003770000
N	2.9504350000	-3.5456940000	0.8037690000
H	3.6044350000	-3.8553980000	1.5073060000
C	2.3231340000	-2.3418860000	0.7734080000
H	2.4534540000	-1.5674940000	1.5142160000
N	1.5358670000	-2.2638950000	-0.2841220000
C	1.6555180000	-3.4599160000	-0.9649140000
H	1.0937400000	-3.6227930000	-1.8716220000
H	2.8971310000	-5.2682480000	-0.4988120000
O	-0.4681930000	-1.8563860000	-2.5843760000
H	-0.3967880000	-1.1801020000	-3.2774340000
H	-1.4336940000	-1.8665210000	-2.2825210000
F	1.0862720000	0.5352480000	-2.0543760000
C	-2.5598460000	-1.4343290000	-0.2474570000
O	-2.8006000000	-1.8135050000	-1.4124870000
O	-1.4323880000	-0.9798640000	0.1799430000
C	-3.6667280000	-1.4923630000	0.7956920000
H	-3.8511920000	-0.4873830000	1.1908300000
H	-3.3463770000	-2.1168660000	1.6369400000
H	-4.5853000000	-1.8948580000	0.3652310000

$^{6}Im_2AcOWatN_3$_FeF

Charge: 0
Multiplicity: 6
B3LYP-D3(BJ)/6-31G(d)-SDD SCF energy (au): -1145.52769527
B3LYP-D3(BJ)/6-31G(d)-SDD enthalpy (au): -1145.26437227
B3LYP-D3(BJ)/6-31G(d)-SDD free energy (au): -1145.34602827
B3LYP-D3(BJ)/def2-TZVP/SMD SCF energy (au): -2285.83596984
B3LYP-D3(BJ)/def2-TZVP/SMD enthalpy (au): -2285.57264684
B3LYP-D3(BJ)/def2-TZVP/SMD free energy (au): -2285.65430284
B3LYP-D3(BJ)/def2-TZVP/SMD free energy (quasi-harmonic) (au): -2285.64799357

Cartesian coordinates

ATOM	X	Y	Z
Fe	0.3240480000	-0.3175630000	-1.2057030000
C	0.9471070000	2.2135530000	2.2682710000
N	2.2490880000	2.1354900000	1.8022130000
H	3.0489690000	2.6221750000	2.1797230000
C	2.2689060000	1.3051440000	0.7316470000
H	3.1637320000	1.0586560000	0.1791870000
N	1.0494420000	0.8459650000	0.4972120000
C	0.2134450000	1.4037830000	1.4456210000
H	-0.8373440000	1.1653350000	1.4477940000
H	0.6758870000	2.8257700000	3.1135600000
C	2.6110180000	-3.9833720000	-0.3540250000
N	2.0701840000	-3.7032350000	0.8901530000
H	2.1873810000	-4.2553250000	1.7269090000
C	1.3376080000	-2.5667240000	0.7852450000
H	0.7740680000	-2.1183590000	1.5880830000
N	1.3884870000	-2.1092270000	-0.4528310000
C	2.1767880000	-2.9800110000	-1.1764510000
H	2.3580670000	-2.8187730000	-2.2270510000
H	3.2362350000	-4.8445490000	-0.5277680000
O	-0.4375060000	-1.6972080000	-2.6380730000
H	-0.8440110000	-1.0906080000	-3.2814410000
H	-1.2149970000	-2.0775070000	-2.0856970000
F	-0.7683900000	0.9493080000	-1.9133890000
C	-2.0868230000	-1.7052670000	0.0057120000
O	-2.2911700000	-2.4017000000	-1.0110140000
O	-1.0831580000	-0.9196420000	0.1872490000
C	-3.0893080000	-1.7487710000	1.1472470000
H	-3.6497970000	-0.8067190000	1.1619460000
H	-2.5707270000	-1.8354460000	2.1072520000
H	-3.7877290000	-2.5771240000	1.0167230000
N	1.9767910000	0.0913920000	-2.2654230000
N	3.1201730000	-0.0933020000	-1.9330190000
N	4.2274780000	-0.2431430000	-1.6195090000

$^{5}Im_2AcOWatN_3$_TS-1

Charge: 0
Multiplicity: 5
B3LYP-D3(BJ)/6-31G(d)-SDD SCF energy (au): -1781.71572522
B3LYP-D3(BJ)/6-31G(d)-SDD enthalpy (au): -1781.15005022

```
B3LYP-D3(BJ)/6-31G(d)-SDD free energy (au):            -1781.27044122
B3LYP-D3(BJ)/def2-TZVP/SMD SCF energy (au):            -2922.26972268
B3LYP-D3(BJ)/def2-TZVP/SMD enthalpy (au):              -2921.70404768
B3LYP-D3(BJ)/def2-TZVP/SMD free energy (au):           -2921.82443868
B3LYP-D3(BJ)/def2-TZVP/SMD free energy (quasi-harmonic) (au): -2921.81141646
```

Cartesian coordinates

ATOM	X	Y	Z
Fe	0.4281060000	-0.4922090000	-1.0939980000
C	0.7199030000	2.3160760000	2.3004230000
N	2.0313520000	2.3860150000	1.8620290000
H	2.7365460000	3.0308760000	2.1868510000
C	2.2064990000	1.4351810000	0.9056820000
H	3.1460660000	1.2552500000	0.4017380000
N	1.0825680000	0.7631830000	0.7162100000
C	0.1468290000	1.3029900000	1.5785440000
H	-0.8589130000	0.9153140000	1.5875480000
H	0.3330490000	2.9772400000	3.0598770000
C	2.2985640000	-4.2657680000	-0.4736200000
N	2.7166430000	-3.5818960000	0.6562360000
H	3.3731260000	-3.9169060000	1.3454200000
C	2.1435680000	-2.3542140000	0.6427140000
H	2.3013770000	-1.5934610000	1.3901760000
N	1.3741080000	-2.2282100000	-0.4245990000
C	1.4623620000	-3.4096630000	-1.1355440000
H	0.9276100000	-3.5314030000	-2.0638810000
H	2.6278290000	-5.2694090000	-0.6904910000
O	-0.4471110000	-1.5862660000	-2.7482940000
H	-0.9298660000	-0.8990360000	-3.2515580000
H	-1.1636820000	-2.0576610000	-2.2208140000
C	-6.0150800000	2.5094870000	-3.6650090000
C	-4.7320980000	2.0013540000	-3.8280830000
C	-3.8340270000	1.9751780000	-2.7496800000
C	-4.2308840000	2.4066570000	-1.4651240000
C	-5.5362580000	2.8944790000	-1.3251140000
C	-6.4142440000	2.9644470000	-2.4053140000
H	-6.7010700000	2.5430210000	-4.5059460000
H	-4.4016800000	1.6237820000	-4.7908890000
H	-5.8707350000	3.2121760000	-0.3404240000
H	-7.4170820000	3.3567700000	-2.2609990000
C	-3.3474200000	2.2992620000	-0.2374390000
C	-2.7139910000	3.6382760000	0.1682770000
H	-2.5608440000	1.5563960000	-0.3850350000
H	-3.9709640000	1.9348870000	0.5903710000
H	-2.0879500000	3.5094000000	1.0580480000
H	-2.0796420000	4.0095130000	-0.6399620000
H	-3.4790030000	4.3921630000	0.3885100000
C	-2.4700860000	1.4394520000	-3.0175240000
O	-2.2782350000	0.3764290000	-3.5920470000
N	-1.4771000000	2.3190110000	-2.5329240000
C	-0.5773450000	2.9804140000	-3.4928610000
C	-1.4799010000	4.0085790000	-4.2204400000
C	0.0798160000	2.0308550000	-4.5072760000

H	-0.8480180000	4.6263310000	-4.8667260000
H	-2.2300580000	3.5144980000	-4.8454970000
H	-1.9944290000	4.6584970000	-3.5066370000
H	0.7080900000	1.3000000000	-3.9933450000
H	-0.6609180000	1.5017340000	-5.1112230000
H	0.7123580000	2.6219720000	-5.1790880000
F	-0.3865000000	1.1791150000	-1.7161720000
C	0.4944460000	3.7144850000	-2.6753790000
H	0.0299220000	4.3645180000	-1.9270480000
H	1.1352240000	2.9914920000	-2.1671660000
H	1.1097580000	4.3282910000	-3.3419080000
C	-2.1471150000	-1.6954060000	-0.1196340000
O	-2.2106160000	-2.5264180000	-1.0450830000
O	-1.2396810000	-0.7884780000	0.0198520000
C	-3.2169430000	-1.6893010000	0.9630050000
H	-3.7491350000	-0.7315180000	0.9388210000
H	-2.7500110000	-1.7775920000	1.9498250000
H	-3.9257470000	-2.5050340000	0.8110850000
N	2.1135510000	0.0305990000	-2.1243560000
N	3.1783200000	-0.1682810000	-1.5979450000
N	4.2106020000	-0.3355850000	-1.0876140000

^{5}Im$_2$AcOWatN$_3$_TS-3-F
Charge: 0
Multiplicity: 5
B3LYP-D3(BJ)/6-31G(d)-SDD SCF energy (au): -1781.78835167
B3LYP-D3(BJ)/6-31G(d)-SDD enthalpy (au): -1781.22199867
B3LYP-D3(BJ)/6-31G(d)-SDD free energy (au): -1781.33894667
B3LYP-D3(BJ)/def2-TZVP/SMD SCF energy (au): -2922.35248229
B3LYP-D3(BJ)/def2-TZVP/SMD enthalpy (au): -2921.78612929
B3LYP-D3(BJ)/def2-TZVP/SMD free energy (au): -2921.90307729
B3LYP-D3(BJ)/def2-TZVP/SMD free energy (quasi-harmonic) (au): -2921.89227862

Cartesian coordinates

ATOM	X	Y	Z
H	34.4670740000	27.0762500000	39.1972890000
C	34.3514060000	27.7949540000	38.4013440000
C	35.1034860000	28.0738000000	37.2917910000
N	33.2910030000	28.6836950000	38.3586070000
C	33.4266630000	29.4503690000	37.2477790000
N	34.5173860000	29.1049810000	36.5852470000
H	32.5201100000	28.7267020000	39.0081280000
H	36.0198600000	27.6172140000	36.9517780000
H	32.7422900000	30.2258250000	36.9448330000
H	39.0250400000	26.3455830000	34.2889080000
C	38.0776920000	26.8578320000	34.2263600000
C	37.6600650000	28.0874090000	34.6653820000
N	36.9739040000	26.2910460000	33.6100020000
C	35.9440470000	27.1746660000	33.6930920000
N	36.3349740000	28.2626790000	34.3275930000
H	36.9376790000	25.3831640000	33.1707150000
H	38.1896680000	28.8658670000	35.1920910000
H	34.9502180000	27.0242870000	33.2976160000

Fe	35.0426130000	30.0260210000	34.6185950000
O	33.7911650000	31.5887530000	35.2665650000
H	34.4387640000	32.1117270000	35.8415190000
H	33.4676210000	32.1485620000	34.5182810000
F	35.7334360000	30.6313610000	32.9161350000
N	34.2307490000	33.1200090000	31.0549950000
O	33.0649040000	32.8766300000	32.9941080000
C	35.2519600000	33.7908260000	33.1613420000
C	34.9469440000	34.8179920000	34.0556770000
C	35.9196300000	35.3546470000	34.8955380000
C	37.2126880000	34.8233300000	34.8742890000
C	37.5195890000	33.7716680000	34.0255040000
C	36.5620950000	33.2302550000	33.1339340000
C	34.0976630000	33.2160170000	32.3948490000
C	33.3300910000	32.3623950000	30.1503730000
C	33.2509560000	30.8990470000	30.6146330000
C	31.9389940000	33.0144160000	30.1319850000
C	33.9680730000	32.4419790000	28.7576800000
C	36.9005750000	32.1021080000	32.3074690000
C	38.1950840000	31.3742720000	32.4525150000
H	33.9257440000	35.1840550000	34.0937530000
H	35.6663370000	36.1588920000	35.5787830000
H	37.9748330000	35.2171020000	35.5401330000
H	38.5185180000	33.3512990000	34.0418640000
H	35.0699510000	33.5119680000	30.6522160000
H	36.3895280000	31.9932390000	31.3629560000
H	38.3695470000	31.1229970000	33.5023800000
H	38.1679190000	30.4463550000	31.8773530000
H	39.0400660000	31.9789460000	32.0887580000
H	32.6230890000	30.3276850000	29.9214250000
H	34.2468890000	30.4480330000	30.6457890000
H	32.8296370000	30.8174790000	31.6174790000
H	31.4972270000	32.9902960000	31.1294430000
H	32.0049560000	34.0567980000	29.7998620000
H	31.2835270000	32.4723900000	29.4412790000
H	34.9629750000	31.9794680000	28.7520670000
H	33.3475220000	31.9076550000	28.0325010000
H	34.0612670000	33.4820720000	28.4210110000
N	33.4733780000	29.0232350000	33.7512370000
N	32.4615910000	28.7725150000	34.3632450000
N	31.4838860000	28.5165080000	34.9313940000
C	36.6868370000	31.7974990000	36.4581160000
O	35.7028340000	32.5022570000	36.7687140000
O	36.7010680000	30.8691050000	35.5705780000
C	38.0110870000	32.0265210000	37.1754010000
H	38.8157300000	32.1501680000	36.4430120000
H	38.2539070000	31.1449750000	37.7802380000
H	37.9569840000	32.9048020000	37.8214430000

^{5}Im$_2$AcOWatN$_3$_TS-3-N$_3$
Charge: 0
Multiplicity: 5
B3LYP-D3(BJ)/6-31G(d)-SDD SCF energy (au): -1781.78462992

B3LYP-D3(BJ)/6-31G(d)-SDD enthalpy (au): -1781.21879692
B3LYP-D3(BJ)/6-31G(d)-SDD free energy (au): -1781.33891292
B3LYP-D3(BJ)/def2-TZVP/SMD SCF energy (au): -2922.35361664
B3LYP-D3(BJ)/def2-TZVP/SMD enthalpy (au): -2921.78778364
B3LYP-D3(BJ)/def2-TZVP/SMD free energy (au): -2921.90789964
B3LYP-D3(BJ)/def2-TZVP/SMD free energy (quasi-harmonic) (au): -2921.89429772

Cartesian coordinates

ATOM	X	Y	Z
H	32.2000520000	26.7204960000	36.1669550000
C	32.7631780000	27.6235580000	35.9917100000
C	34.1052570000	27.8657290000	35.8660500000
N	32.1531370000	28.8556330000	35.8398180000
C	33.1226840000	29.7840430000	35.6318870000
N	34.3140620000	29.2116120000	35.6442890000
H	31.1609680000	29.0373880000	35.8649940000
H	34.9323740000	27.1761720000	35.9207990000
H	32.9527090000	30.8388310000	35.4866160000
H	38.9340250000	25.9722320000	36.6753400000
C	38.3869520000	26.6814230000	36.0742410000
C	37.7175840000	27.8353110000	36.3864020000
N	38.2574200000	26.5362040000	34.7022920000
C	37.5289100000	27.5853700000	34.2378730000
N	37.1869350000	28.3782820000	35.2343680000
H	38.6495180000	25.7939360000	34.1422210000
H	37.5808690000	28.3436190000	37.3277790000
H	37.2828580000	27.7465900000	33.1999230000
Fe	36.2018280000	30.3625620000	35.0657860000
O	34.9298460000	32.0009700000	35.3829000000
H	35.4441280000	32.5058600000	36.1029340000
H	34.5745480000	32.5859540000	34.6802830000
F	37.7501330000	31.0857880000	34.3933370000
N	33.4313060000	32.3506840000	31.1671620000
O	33.4171980000	33.0042950000	33.3498050000
C	35.4990470000	33.1133410000	32.2123060000
C	35.8814370000	34.3343610000	32.7716700000
C	37.2202110000	34.7140120000	32.8275200000
C	38.2004600000	33.8382090000	32.3505320000
C	37.8403710000	32.6057130000	31.8325840000
C	36.4868070000	32.2039320000	31.7278990000
C	34.0359060000	32.8077700000	32.2903980000
C	31.9940360000	32.0201080000	31.0262790000
C	31.5889210000	30.9643360000	32.0675500000
C	31.1522750000	33.2966960000	31.1879970000
C	31.8280600000	31.4434230000	29.6146460000
C	36.1593600000	30.8879690000	31.2629420000
C	37.2010330000	29.9166030000	30.8015710000
H	35.1097510000	34.9842640000	33.1717790000
H	37.4969660000	35.6716100000	33.2572190000
H	39.2501710000	34.1090320000	32.4120370000
H	38.6172240000	31.9213010000	31.5151700000
H	33.9755980000	32.3927630000	30.3165410000
H	35.1531980000	30.7118180000	30.9074330000

H	37.9854360000	29.8002790000	31.5556060000
H	36.7496840000	28.9380410000	30.6116530000
H	37.6762300000	30.2489380000	29.8659570000
H	30.5361920000	30.6971670000	31.9204890000
H	32.1938880000	30.0584970000	31.9656440000
H	31.7154680000	31.3566380000	33.0775810000
H	31.3106540000	33.7289570000	32.1785470000
H	31.4280290000	34.0404610000	30.4318650000
H	30.0876720000	33.0655190000	31.0702740000
H	32.4255020000	30.5328350000	29.4914080000
H	30.7795760000	31.1878880000	29.4352990000
H	32.1304210000	32.1702660000	28.8498990000
N	35.6146810000	29.8424150000	33.0837200000
N	34.7536450000	29.0556070000	32.7532730000
N	33.9545200000	28.3043720000	32.3824020000
C	36.8650570000	31.6697300000	37.6769580000
O	36.3924330000	32.7762650000	37.3332960000
O	36.6742530000	30.5510650000	37.0723270000
C	37.7526840000	31.5968530000	38.9111210000
H	38.7717090000	31.3384260000	38.6007220000
H	37.4021320000	30.8026340000	39.5786970000
H	37.7654220000	32.5518950000	39.4397320000

^{5}Im$_2$AcOWatOH_11
Charge: 0
Multiplicity: 5
B3LYP-D3(BJ)/6-31G(d)-SDD SCF energy (au): -1693.35290801
B3LYP-D3(BJ)/6-31G(d)-SDD enthalpy (au): -1692.78523101
B3LYP-D3(BJ)/6-31G(d)-SDD free energy (au): -1692.90166501
B3LYP-D3(BJ)/def2-TZVP/SMD SCF energy (au): -2833.88358650
B3LYP-D3(BJ)/def2-TZVP/SMD enthalpy (au): -2833.31590950
B3LYP-D3(BJ)/def2-TZVP/SMD free energy (au): -2833.43234350
B3LYP-D3(BJ)/def2-TZVP/SMD free energy (quasi-harmonic) (au): -2833.41974149

Cartesian coordinates

ATOM	X	Y	Z
Fe	0.1870350000	-0.4717890000	-0.4695470000
C	0.7393360000	2.3855580000	2.8844300000
N	1.3476160000	3.0191740000	1.8130080000
H	1.7226240000	3.9560070000	1.8111260000
C	1.3208300000	2.1688510000	0.7506710000
H	1.6806920000	2.3861300000	-0.2440970000
N	0.7365720000	1.0343030000	1.0906000000
C	0.3649520000	1.1534770000	2.4141800000
H	-0.1676780000	0.3527130000	2.9032020000
O	0.6841640000	0.8638960000	-1.7509460000
H	0.6341580000	0.5236660000	-2.6562560000
H	0.6296160000	2.8631270000	3.8455950000
C	3.1158260000	-3.6564710000	-0.1689210000
N	3.5389300000	-2.8284010000	0.8583540000
H	4.3230090000	-2.9946830000	1.4715100000
C	2.7088100000	-1.7535900000	0.8973280000
H	2.7953630000	-0.9403260000	1.6020600000

N	1.7883970000	-1.8530510000	-0.0432940000
C	2.0265390000	-3.0344580000	-0.7180310000
H	1.3846850000	-3.3259970000	-1.5352040000
H	3.6185150000	-4.5822390000	-0.4005830000
O	-0.5959120000	-1.9297030000	-1.9552590000
H	-0.9970150000	-1.3890950000	-2.6671330000
H	-1.3762780000	-2.2430730000	-1.4058240000
C	-5.2175530000	3.0862390000	-4.1180830000
C	-4.0213930000	2.4285740000	-4.4041680000
C	-3.2946190000	1.8064960000	-3.3852300000
C	-3.7608050000	1.8042260000	-2.0528180000
C	-4.9521120000	2.4873050000	-1.7887470000
C	-5.6747230000	3.1207990000	-2.8027090000
H	-5.7819620000	3.5634790000	-4.9136520000
H	-3.6510300000	2.3823220000	-5.4233270000
H	-5.3269250000	2.5242880000	-0.7715460000
H	-6.6001680000	3.6362420000	-2.5605710000
C	-2.9846280000	1.0910760000	-0.9636060000
C	-3.2372090000	1.5825940000	0.4632920000
H	-1.9139880000	1.1785240000	-1.1761390000
H	-3.1990490000	0.0171110000	-1.0264410000
H	-2.5512160000	1.0676430000	1.1403900000
H	-3.0654150000	2.6624900000	0.5498940000
H	-4.2602940000	1.3762800000	0.7987730000
C	-2.0629950000	1.0319010000	-3.7370520000
O	-2.0158540000	-0.1849900000	-3.5953150000
N	-1.0535140000	1.7511170000	-4.3434320000
C	-0.5336890000	3.1465010000	-4.0578860000
C	-0.8269320000	3.5637120000	-2.6078640000
C	-1.1778510000	4.1123400000	-5.0627870000
H	-0.3314120000	4.5231880000	-2.4193070000
H	-1.8940560000	3.6993920000	-2.4247940000
H	-0.4181110000	2.8060520000	-1.9328210000
H	-1.0008370000	3.7787970000	-6.0905230000
H	-2.2543030000	4.1980810000	-4.9007800000
H	-0.7370180000	5.1077370000	-4.9408930000
F	-0.0105780000	0.8667380000	-4.6815170000
C	0.9913420000	3.1366250000	-4.2566120000
H	1.4701260000	2.4855810000	-3.5226090000
H	1.2717970000	2.8238000000	-5.2648630000
H	1.3458910000	4.1608480000	-4.1035160000
C	-2.1883440000	-1.8788880000	0.8093170000
O	-2.5103110000	-2.5044850000	-0.2206940000
O	-1.1918790000	-1.0703460000	0.9201160000
C	-3.0379100000	-2.0276740000	2.0651080000
H	-3.7770780000	-2.8213390000	1.9419540000
H	-3.5537290000	-1.0810820000	2.2648110000
H	-2.3991850000	-2.2387070000	2.9290680000

^{5}Im$_2$AcOWatOH_13
Charge: 0
Multiplicity: 5
B3LYP-D3(BJ)/6-31G(d)-SDD SCF energy (au): -1693.41879127

B3LYP-D3(BJ)/6-31G(d)-SDD enthalpy (au): -1692.85339927
B3LYP-D3(BJ)/6-31G(d)-SDD free energy (au): -1692.96934927
B3LYP-D3(BJ)/def2-TZVP/SMD SCF energy (au): -2833.95216894
B3LYP-D3(BJ)/def2-TZVP/SMD enthalpy (au): -2833.38677694
B3LYP-D3(BJ)/def2-TZVP/SMD free energy (au): -2833.50272694
B3LYP-D3(BJ)/def2-TZVP/SMD free energy (quasi-harmonic) (au): -2833.49074067

Cartesian coordinates

ATOM	X	Y	Z
H	35.1091280000	29.9996210000	39.1938020000
C	34.8646290000	29.9573800000	38.1440420000
C	35.6374920000	29.8440270000	37.0191920000
N	33.5563860000	30.0315640000	37.6888600000
C	33.5761010000	29.9657950000	36.3329160000
N	34.8208350000	29.8447610000	35.9084280000
H	32.7334710000	30.1512060000	38.2606740000
H	36.7082040000	29.7830100000	36.9040910000
H	32.7204850000	30.0243170000	35.6765320000
H	36.5845240000	25.0391510000	32.3821530000
C	36.2284100000	25.8860480000	32.9471440000
C	36.2092330000	27.2291630000	32.6830350000
N	35.6508130000	25.7451520000	34.1983870000
C	35.3070490000	26.9826740000	34.6422270000
N	35.6349470000	27.8922920000	33.7468040000
H	35.5059950000	24.8779350000	34.6933540000
H	36.5491620000	27.7828740000	31.8237600000
H	34.8359450000	27.1804200000	35.5919460000
Fe	35.3007610000	30.0702200000	33.7553090000
O	35.2905800000	32.1704800000	34.1019390000
H	36.2693190000	32.3966440000	33.9316940000
H	34.7529530000	32.5732010000	33.4007570000
F	35.9354740000	30.0892120000	32.0113270000
N	34.6806350000	30.9696650000	29.1003800000
O	33.1380220000	30.4218060000	30.6813360000
C	34.2523730000	32.5264870000	30.9110710000
C	33.1272490000	33.1155280000	31.4892590000
C	33.1821470000	34.3821110000	32.0746920000
C	34.3981470000	35.0798340000	32.0793750000
C	35.5366150000	34.5036210000	31.5413280000
C	35.5259950000	33.1972200000	30.9664840000
C	33.9998320000	31.2011200000	30.2489900000
C	34.6789080000	29.7023930000	28.3422890000
C	35.1410110000	28.5479780000	29.2473200000
C	33.2726110000	29.4288020000	27.7845560000
C	35.6725120000	29.8946050000	27.1891680000
C	36.7419600000	32.5768020000	30.5856080000
C	38.0845740000	33.2248530000	30.6702960000
H	32.1989370000	32.5551540000	31.4682440000
H	32.2917960000	34.8189080000	32.5160000000
H	34.4580940000	36.0664590000	32.5305120000
H	36.4802990000	35.0320710000	31.6110980000
H	35.3599080000	31.6666370000	28.8288680000
H	36.7262510000	31.5049410000	30.4423090000

H	38.4443840000	33.1831430000	31.7104390000
H	38.8110080000	32.6885470000	30.0500960000
H	38.0794340000	34.2744480000	30.3535910000
H	35.1607480000	27.6124830000	28.6757340000
H	36.1434250000	28.7503630000	29.6354390000
H	34.4672450000	28.4353280000	30.0976620000
H	32.5567470000	29.3305590000	28.6022270000
H	32.9525800000	30.2504890000	27.1341730000
H	33.2751280000	28.5033100000	27.1971630000
H	36.6815490000	30.0965900000	27.5690720000
H	35.7178440000	28.9890060000	26.5765880000
H	35.3698660000	30.7269660000	26.5424210000
C	38.1284630000	31.1046800000	34.1405030000
O	37.8432120000	32.2811490000	33.8052720000
O	37.2863500000	30.2043040000	34.4873530000
C	39.5880420000	30.6757390000	34.1198970000
H	39.7629240000	30.0840670000	33.2130190000
H	39.8110310000	30.0343190000	34.9773810000
H	40.2508870000	31.5436520000	34.1091530000
O	33.4637580000	30.0271650000	33.4838140000
H	33.2488910000	30.0956730000	32.5283980000

^{5}Im$_2$AcOWatOH_FeF_remove-F

Charge: 0
Multiplicity: 5
B3LYP-D3(BJ)/6-31G(d)-SDD SCF energy (au): -957.283078025
B3LYP-D3(BJ)/6-31G(d)-SDD enthalpy (au): -957.025412025
B3LYP-D3(BJ)/6-31G(d)-SDD free energy (au): -957.101774025
B3LYP-D3(BJ)/def2-TZVP/SMD SCF energy (au): -2097.51413418
B3LYP-D3(BJ)/def2-TZVP/SMD enthalpy (au): -2097.25646818
B3LYP-D3(BJ)/def2-TZVP/SMD free energy (au): -2097.33283018
B3LYP-D3(BJ)/def2-TZVP/SMD free energy (quasi-harmonic) (au): -2097.327514155

Cartesian coordinates

ATOM	X	Y	Z
Fe	0.2859980000	-0.8585110000	1.2009830000
C	-2.8767280000	-1.4591000000	-1.7529960000
N	-2.0110160000	-0.6738630000	-2.4968770000
H	-2.1645910000	-0.3473360000	-3.4391270000
C	-0.9293710000	-0.3933670000	-1.7200970000
H	-0.0957160000	0.2225710000	-2.0198490000
N	-1.0591610000	-0.9598120000	-0.5362230000
C	-2.2685460000	-1.6254040000	-0.5367570000
H	-2.5966530000	-2.1406740000	0.3532130000
H	-3.8172800000	-1.8073220000	-2.1502360000
C	2.1987700000	-4.6078250000	0.0518360000
N	3.2886200000	-3.7582850000	-0.0289250000
H	4.2277350000	-4.0176360000	-0.2923790000
C	2.8783480000	-2.5089180000	0.3176850000
H	3.5120680000	-1.6325080000	0.3577940000
N	1.5877310000	-2.5206860000	0.6096130000
C	1.1510710000	-3.8206280000	0.4501150000
H	0.1216150000	-4.0813500000	0.6429950000

H	2.2734910000	-5.6602180000	-0.1726320000
O	-1.0317520000	-1.6143980000	2.3092580000
H	-0.9383930000	-1.3478270000	3.2355510000
C	2.4940860000	0.9445740000	0.1465570000
O	3.3416220000	0.5057950000	0.9640790000
O	1.2529010000	0.6323130000	0.1231480000
C	2.9506490000	1.9111950000	-0.9381890000
H	2.2901910000	2.7837590000	-0.9595050000
H	2.8752820000	1.4223630000	-1.9172470000
H	3.9823750000	2.2260940000	-0.7718810000
O	1.7571740000	-0.4288900000	2.8814710000
H	2.0544030000	-1.3236770000	3.1085990000
H	2.4682000000	-0.0618150000	2.2762240000

[5]Im$_2$AcOWatOH_FeF_remove-OH

Charge: 0
Multiplicity: 5
B3LYP-D3(BJ)/6-31G(d)-SDD SCF energy (au): -981.331484369
B3LYP-D3(BJ)/6-31G(d)-SDD enthalpy (au): -981.085064369
B3LYP-D3(BJ)/6-31G(d)-SDD free energy (au): -981.160461369
B3LYP-D3(BJ)/def2-TZVP/SMD SCF energy (au): -2121.57189667
B3LYP-D3(BJ)/def2-TZVP/SMD enthalpy (au): -2121.32547667
B3LYP-D3(BJ)/def2-TZVP/SMD free energy (au): -2121.40087367
B3LYP-D3(BJ)/def2-TZVP/SMD free energy (quasi-harmonic) (au): -2121.395374301

Cartesian coordinates

ATOM	X	Y	Z
Fe	0.4281050000	-0.4134470000	0.9861720000
C	-2.0488830000	-0.6275320000	-2.6623630000
N	-2.9217210000	-1.0031010000	-1.6547990000
H	-3.8983190000	-1.2307290000	-1.7676500000
C	-2.2391190000	-0.9858490000	-0.4791260000
H	-2.6517590000	-1.2149540000	0.4917640000
N	-0.9851840000	-0.6264850000	-0.6868020000
C	-0.8504190000	-0.3966370000	-2.0395510000
H	0.0935470000	-0.0681450000	-2.4454300000
H	-2.3588920000	-0.5590930000	-3.6933440000
C	1.6554640000	-4.5877680000	0.8586660000
N	2.8752830000	-3.9789280000	0.6203900000
H	3.7556790000	-4.4483130000	0.4682800000
C	2.6875960000	-2.6325580000	0.6349810000
H	3.4633290000	-1.8917110000	0.4936590000
N	1.4143700000	-2.3558050000	0.8648490000
C	0.7596110000	-3.5628360000	1.0081380000
H	-0.2983710000	-3.5917470000	1.2190530000
H	1.5473430000	-5.6601300000	0.9006770000
F	-0.9533500000	-0.8439780000	2.1441220000
C	2.8905310000	0.7058590000	-0.3139180000
O	3.6280190000	0.3099410000	0.6232560000
O	1.6119260000	0.6219060000	-0.3377410000
C	3.5304430000	1.3248300000	-1.5478510000
H	3.0800940000	2.3032570000	-1.7436770000
H	3.3258580000	0.6948110000	-2.4214330000

H	4.6089490000	1.4299320000	-1.4176090000
O	1.8263920000	0.2728090000	2.5722790000
H	1.9023980000	-0.4815380000	3.1765400000
H	2.6427770000	0.2644030000	1.9911990000

^{6}Im$_2$AcOWatOH_FeF

Charge: 0
Multiplicity: 6
B3LYP-D3(BJ)/6-31G(d)-SDD SCF energy (au): -1057.14588096
B3LYP-D3(BJ)/6-31G(d)-SDD enthalpy (au): -1056.88392196
B3LYP-D3(BJ)/6-31G(d)-SDD free energy (au): -1056.96007596
B3LYP-D3(BJ)/def2-TZVP/SMD SCF energy (au): -2197.42509744
B3LYP-D3(BJ)/def2-TZVP/SMD enthalpy (au): -2197.16313844
B3LYP-D3(BJ)/def2-TZVP/SMD free energy (au): -2197.23929244
B3LYP-D3(BJ)/def2-TZVP/SMD free energy (quasi-harmonic) (au): -2197.23498448

Cartesian coordinates

ATOM	X	Y	Z
Fe	0.2658740000	-0.5227400000	1.3283950000
C	-1.9988520000	-1.5957990000	-2.3126840000
N	-2.5461600000	-0.3969390000	-1.8881530000
H	-3.2832140000	0.1176860000	-2.3468030000
C	-1.9164140000	-0.0255840000	-0.7456350000
H	-2.1155500000	0.8690750000	-0.1765310000
N	-1.0017910000	-0.9226150000	-0.4223940000
C	-1.0383300000	-1.9060980000	-1.3874870000
H	-0.3798540000	-2.7590020000	-1.3415910000
H	-2.3371350000	-2.0983000000	-3.2050580000
C	1.7889490000	-4.5968040000	0.4167090000
N	2.8803940000	-3.8638690000	-0.0210910000
H	3.7331850000	-4.2384070000	-0.4102130000
C	2.6217740000	-2.5484900000	0.1962120000
H	3.3184120000	-1.7457570000	0.0041110000
N	1.4269180000	-2.4063350000	0.7442890000
C	0.8969950000	-3.6740700000	0.8947530000
H	-0.0700840000	-3.7995910000	1.3554060000
H	1.7584580000	-5.6731970000	0.3539330000
O	-0.5374880000	-1.7277370000	2.5249580000
H	-1.2523240000	-1.2444110000	2.9709150000
F	-0.7896350000	0.9376340000	1.6583040000
C	2.7242960000	0.7516280000	-0.0418980000
O	3.5568660000	0.3447460000	0.8106110000
O	1.4797550000	0.4620650000	-0.0504360000
C	3.2019300000	1.6473040000	-1.1734110000
H	2.7062650000	2.6206950000	-1.0915680000
H	2.9108330000	1.2173720000	-2.1376140000
H	4.2841520000	1.7832340000	-1.1321780000
O	1.8545950000	-0.1592810000	2.7087020000
H	1.9362810000	-1.0137370000	3.1617940000
H	2.6527840000	-0.0383590000	2.0942570000

^{5}Im$_2$AcOWatOH_TS-1

Charge: 0

Multiplicity: 5
B3LYP-D3(BJ)/6-31G(d)-SDD SCF energy (au): -1693.32360708
B3LYP-D3(BJ)/6-31G(d)-SDD enthalpy (au): -1692.75945808
B3LYP-D3(BJ)/6-31G(d)-SDD free energy (au): -1692.87577008
B3LYP-D3(BJ)/def2-TZVP/SMD SCF energy (au): -2833.85173138
B3LYP-D3(BJ)/def2-TZVP/SMD enthalpy (au): -2833.28758238
B3LYP-D3(BJ)/def2-TZVP/SMD free energy (au): -2833.40389438
B3LYP-D3(BJ)/def2-TZVP/SMD free energy (quasi-harmonic) (au): -2833.39236730

Cartesian coordinates

ATOM	X	Y	Z
Fe	0.3588150000	-0.6547090000	1.2343600000
C	-2.1014670000	-1.7021270000	-2.3720780000
N	-2.5279740000	-0.4243930000	-2.0536860000
H	-3.2271950000	0.1106680000	-2.5466120000
C	-1.8417050000	-0.0159930000	-0.9520970000
H	-1.9650980000	0.9433730000	-0.4753060000
N	-1.0060340000	-0.9568270000	-0.5561050000
C	-1.1552250000	-2.0121280000	-1.4298710000
H	-0.5773540000	-2.9164890000	-1.3124520000
H	-2.5030030000	-2.2493710000	-3.2105770000
C	1.7958820000	-4.6335480000	0.4540750000
N	2.8906560000	-3.9308670000	-0.0228350000
H	3.7312510000	-4.3296150000	-0.4136400000
C	2.6568280000	-2.6048910000	0.1625390000
H	3.3540520000	-1.8117010000	-0.0653790000
N	1.4725540000	-2.4312760000	0.7230920000
C	0.9237730000	-3.6850610000	0.9176770000
H	-0.0376840000	-3.7782670000	1.3990360000
H	1.7492250000	-5.7108230000	0.4252360000
O	-0.5082430000	-1.6588920000	2.5299650000
H	-1.1320930000	-1.0713790000	2.9937170000
C	-5.2552050000	3.3534900000	5.1947570000
C	-4.1252080000	2.6269100000	4.8370190000
C	-3.8742250000	2.3144680000	3.4928140000
C	-4.7935360000	2.6739740000	2.4882540000
C	-5.9331190000	3.3918990000	2.8757400000
C	-6.1590760000	3.7457920000	4.2040310000
H	-5.4356320000	3.6048730000	6.2356090000
H	-3.4159250000	2.2970200000	5.5899440000
H	-6.6629400000	3.6633190000	2.1164330000
H	-7.0492200000	4.3091480000	4.4695690000
C	-4.6449830000	2.2634230000	1.0359860000
C	-4.1473510000	3.3948040000	0.1251560000
H	-3.9706150000	1.4071220000	0.9515970000
H	-5.6274630000	1.9255900000	0.6813500000
H	-4.1396230000	3.0744730000	-0.9239860000
H	-3.1305670000	3.6835920000	0.4019310000
H	-4.7914790000	4.2781150000	0.2009740000
C	-2.6137280000	1.5537050000	3.2004410000
O	-2.3509730000	0.4987830000	3.7497240000
N	-1.8485560000	2.1924050000	2.2081340000
C	-0.8117140000	3.1829970000	2.5863520000

C	-1.5570220000	4.4487350000	3.0722680000
C	0.1166110000	2.6594290000	3.6914940000
H	-0.8147870000	5.2402340000	3.2189760000
H	-2.0731970000	4.2828540000	4.0201620000
H	-2.2891260000	4.7913710000	2.3358030000
H	0.6477050000	1.7605980000	3.3734210000
H	-0.4461240000	2.4272420000	4.6021100000
H	0.8529860000	3.4327100000	3.9377280000
F	-0.9205640000	0.8723260000	1.5888780000
C	-0.0227150000	3.5254780000	1.3145400000
H	-0.6961670000	3.8975880000	0.5343720000
H	0.5035970000	2.6498820000	0.9320880000
H	0.7056950000	4.3112210000	1.5428550000
C	2.6741750000	0.8052530000	-0.0255560000
O	3.5297650000	0.2488000000	0.7020800000
O	1.4079400000	0.6041900000	0.0202940000
C	3.1259460000	1.8164550000	-1.0706430000
H	2.6947470000	2.7959120000	-0.8345190000
H	2.7479330000	1.5279980000	-2.0571740000
H	4.2146050000	1.8933670000	-1.0928180000
O	1.9273510000	-0.0894660000	2.8215810000
H	1.8430110000	-0.9692770000	3.2232020000
H	2.6876890000	-0.1103040000	2.1806940000

^{5}Im$_2$AcOWatOH_TS-3-F

Charge: 0
Multiplicity: 5
B3LYP-D3(BJ)/6-31G(d)-SDD SCF energy (au): -1693.39728376
B3LYP-D3(BJ)/6-31G(d)-SDD enthalpy (au): -1692.83216776
B3LYP-D3(BJ)/6-31G(d)-SDD free energy (au): -1692.94522376
B3LYP-D3(BJ)/def2-TZVP/SMD SCF energy (au): -2833.93164307
B3LYP-D3(BJ)/def2-TZVP/SMD enthalpy (au): -2833.36652707
B3LYP-D3(BJ)/def2-TZVP/SMD free energy (au): -2833.47958307
B3LYP-D3(BJ)/def2-TZVP/SMD free energy (quasi-harmonic) (au): -2833.46954231

Cartesian coordinates

ATOM	X	Y	Z
H	33.5512180000	27.8926570000	39.6626750000
C	33.5856310000	28.2645230000	38.6505030000
C	34.5843660000	28.8382130000	37.9082490000
N	32.4791870000	28.2317320000	37.8161910000
C	32.8362920000	28.7777680000	36.6227130000
N	34.1020140000	29.1469230000	36.6554840000
H	31.5633840000	27.8827560000	38.0554860000
H	35.6064890000	29.0663700000	38.1666930000
H	32.1973430000	28.9070990000	35.7616250000
H	35.9290360000	25.4554440000	32.5874320000
C	36.0220490000	26.3298930000	33.2123730000
C	35.2426390000	27.4471990000	33.3630160000
N	37.0857500000	26.4991390000	34.0849860000
C	36.9177690000	27.6902240000	34.7232340000
N	35.8156540000	28.2758230000	34.3026550000
H	37.8484680000	25.8540160000	34.2255690000

H	34.3195300000	27.7379540000	32.8853000000
H	37.5827030000	28.1028270000	35.4664810000
Fe	35.0015370000	30.2201570000	34.9397070000
O	34.4341850000	32.1543550000	35.7004420000
H	35.3505260000	32.5148380000	35.8958790000
H	34.0571610000	32.6216350000	34.9276540000
F	36.1732160000	30.9898220000	33.5141040000
N	34.3953740000	32.9533030000	31.1144920000
O	33.2912510000	32.7256840000	33.1124760000
C	35.2364180000	34.0755210000	33.0915560000
C	34.7554870000	35.2373660000	33.6937990000
C	35.5984810000	36.0526990000	34.4488070000
C	36.9278070000	35.6667670000	34.6456480000
C	37.4055130000	34.4898820000	34.0885600000
C	36.5887240000	33.6696850000	33.2763220000
C	34.2256640000	33.1805910000	32.4357590000
C	33.6944540000	31.9531110000	30.2716000000
C	34.1009200000	30.5344310000	30.7037470000
C	32.1738860000	32.1481010000	30.3663650000
C	34.1602820000	32.2215740000	28.8336700000
C	37.0975740000	32.4427900000	32.7095140000
C	38.4887400000	31.9740320000	32.9837270000
H	33.7062770000	35.4917710000	33.5762820000
H	35.2164460000	36.9642860000	34.8980250000
H	37.5870730000	36.2759580000	35.2568440000
H	38.4266540000	34.1892570000	34.2859160000
H	35.1847110000	33.4217130000	30.6911740000
H	36.6733070000	32.1279850000	31.7664100000
H	38.6980810000	31.9963880000	34.0560940000
H	38.6125720000	30.9493200000	32.6269370000
H	39.2310560000	32.6088780000	32.4765200000
H	33.6079090000	29.7991190000	30.0562330000
H	35.1844120000	30.4005840000	30.6103600000
H	33.8283330000	30.3245510000	31.7416230000
H	31.8225130000	31.9730480000	31.3833260000
H	31.8979370000	33.1671580000	30.0730450000
H	31.6737400000	31.4460620000	29.6901920000
H	35.2472120000	32.0998980000	28.7408710000
H	33.6877400000	31.5105340000	28.1497340000
H	33.8922790000	33.2349530000	28.5123250000
C	37.3725350000	31.3530710000	36.4786210000
O	36.9817440000	32.5310380000	36.3270120000
O	36.6971840000	30.2943260000	36.2214700000
C	38.7889390000	31.1115980000	36.9960480000
H	39.4032460000	30.7017010000	36.1844120000
H	38.7767690000	30.3685830000	37.8002980000
H	39.2400290000	32.0400800000	37.3523380000
O	33.4004180000	29.9192800000	33.9515380000
H	33.0616650000	30.7735750000	33.6366260000

^{5}Im$_2$AcOWatOH_TS-3-OH
Charge: 0
Multiplicity: 5

B3LYP-D3(BJ)/6-31G(d)-SDD SCF energy (au): -1693.37592699
B3LYP-D3(BJ)/6-31G(d)-SDD enthalpy (au): -1692.81174199
B3LYP-D3(BJ)/6-31G(d)-SDD free energy (au): -1692.92628199
B3LYP-D3(BJ)/def2-TZVP/SMD SCF energy (au): -2833.93101328
B3LYP-D3(BJ)/def2-TZVP/SMD enthalpy (au): -2833.36682828
B3LYP-D3(BJ)/def2-TZVP/SMD free energy (au): -2833.48136828
B3LYP-D3(BJ)/def2-TZVP/SMD free energy (quasi-harmonic) (au): -2833.46975229

Cartesian coordinates

ATOM	X	Y	Z
H	31.9962890000	26.9758330000	36.7271170000
C	32.5868840000	27.8439290000	36.4800170000
C	33.7937810000	27.9895800000	35.8500070000
N	32.1823260000	29.1285150000	36.8007570000
C	33.1339130000	29.9942310000	36.3673830000
N	34.1185710000	29.3299210000	35.7864770000
H	31.3351850000	29.3839720000	37.2859710000
H	34.4608400000	27.2413390000	35.4543220000
H	33.1099270000	31.0676910000	36.4702820000
H	39.0131370000	26.0222830000	36.4121470000
C	38.3234500000	26.6645700000	35.8870990000
C	37.5205840000	27.6957060000	36.2994650000
N	38.1075870000	26.5509530000	34.5227030000
C	37.2031840000	27.5024500000	34.1638990000
N	36.8286140000	28.1975890000	35.2186290000
H	38.5694340000	25.9057630000	33.8993370000
H	37.4040150000	28.1466810000	37.2725100000
H	36.8717570000	27.6868360000	33.1537230000
Fe	35.9281710000	30.2778000000	35.0716060000
O	34.7571320000	32.0808380000	35.4126410000
H	35.4845790000	32.5993940000	35.8764930000
H	34.4072270000	32.5487010000	34.6250870000
F	37.5678390000	30.7764070000	34.3887710000
N	33.4893110000	32.4696990000	30.9920390000
O	33.2528820000	32.9016810000	33.2174480000
C	35.4163040000	33.2006020000	32.2796960000
C	35.7179290000	34.4192040000	32.8868980000
C	37.0393040000	34.7977300000	33.1195700000
C	38.0707660000	33.9169150000	32.7786070000
C	37.7836190000	32.6849360000	32.2138040000
C	36.4536350000	32.2857990000	31.9259390000
C	33.9690340000	32.8393330000	32.2055960000
C	32.1467600000	31.9111950000	30.7278990000
C	31.9353570000	30.6514850000	31.5863040000
C	31.0703580000	32.9647390000	31.0352900000
C	32.1163210000	31.5430890000	29.2394700000
C	36.1864040000	30.9781000000	31.4189860000
C	37.2667520000	29.9650780000	31.2347150000
H	34.9000210000	35.0660590000	33.1901620000
H	37.2596590000	35.7529110000	33.5857390000
H	39.1031200000	34.1834370000	32.9854090000
H	38.5938020000	31.9983070000	32.0054360000
H	34.1389700000	32.5106800000	30.2194630000

H	35.2160530000	30.7671100000	30.9960700000
H	37.8135220000	29.8539310000	32.1777940000
H	36.8429850000	28.9978660000	30.9490840000
H	37.9841900000	30.2607700000	30.4530180000
H	30.9486850000	30.2197370000	31.3847060000
H	32.6962980000	29.8981740000	31.3527200000
H	32.0023760000	30.9000220000	32.6473720000
H	31.1196680000	33.2574190000	32.0856030000
H	31.2201950000	33.8557300000	30.4158670000
H	30.0744540000	32.5595190000	30.8228280000
H	32.8813690000	30.7937730000	29.0018900000
H	31.1406420000	31.1241840000	28.9755410000
H	32.2855700000	32.4262340000	28.6118140000
C	37.0937320000	31.6823340000	37.2999610000
O	36.7681050000	32.8100680000	36.8816560000
O	36.5545880000	30.5602690000	36.9496260000
C	38.2249330000	31.5543890000	38.3087580000
H	39.0966940000	31.1321590000	37.7949410000
H	37.9469520000	30.8621370000	39.1099510000
H	38.4890580000	32.5287570000	38.7248690000
O	35.3292570000	30.0237300000	33.2801490000
H	34.4271450000	29.6553640000	33.2677480000

[5]$Im_2WatOHN_3$_11
Charge: 0
Multiplicity: 5
B3LYP-D3(BJ)/6-31G(d)-SDD SCF energy (au): -1629.03798869
B3LYP-D3(BJ)/6-31G(d)-SDD enthalpy (au): -1628.51129569
B3LYP-D3(BJ)/6-31G(d)-SDD free energy (au): -1628.62336469
B3LYP-D3(BJ)/def2-TZVP/SMD SCF energy (au): -2769.53627779
B3LYP-D3(BJ)/def2-TZVP/SMD enthalpy (au): -2769.00958479
B3LYP-D3(BJ)/def2-TZVP/SMD free energy (au): -2769.12165379
B3LYP-D3(BJ)/def2-TZVP/SMD free energy (quasi-harmonic) (au): -2769.11101110

Cartesian coordinates

ATOM	X	Y	Z
Fe	0.1193830000	-0.8928110000	-0.7224550000
C	-1.6638260000	-0.2101600000	3.2900070000
N	-1.2303740000	1.0410470000	2.8846010000
H	-1.3745640000	1.9092220000	3.3781920000
C	-0.5997170000	0.9013830000	1.6874670000
H	-0.1882000000	1.7041610000	1.0942310000
N	-0.6065640000	-0.3655630000	1.3145690000
C	-1.2672380000	-1.0706580000	2.3000320000
H	-1.4176780000	-2.1357880000	2.2117450000
H	-2.2019230000	-0.3602360000	4.2127360000
C	3.6649010000	-2.0715110000	1.5521050000
N	4.0946040000	-2.2682090000	0.2515880000
H	5.0069980000	-2.5913140000	-0.0349750000
C	3.0751110000	-1.9539300000	-0.5869620000
H	3.1438450000	-2.0231580000	-1.6650360000
N	2.0166290000	-1.5650970000	0.1101920000
C	2.3728050000	-1.6338490000	1.4444700000

H	1.6699870000	-1.3675020000	2.2178970000
H	4.3014220000	-2.2584980000	2.4024990000
O	-0.0168270000	0.9498360000	-1.1749580000
H	0.3356350000	1.0998600000	-2.0649400000
C	-7.1893460000	1.5607970000	-1.4726440000
C	-6.2560240000	0.5554080000	-1.7040570000
C	-4.9158680000	0.7295440000	-1.3297430000
C	-4.4848500000	1.9207100000	-0.7161960000
C	-5.4426320000	2.9212400000	-0.5044610000
C	-6.7762410000	2.7508980000	-0.8709260000
H	-8.2276610000	1.4166230000	-1.7556890000
H	-6.5555490000	-0.3786120000	-2.1686370000
H	-5.1288280000	3.8535950000	-0.0418160000
H	-7.4934890000	3.5455620000	-0.6851690000
C	-3.0387270000	2.1791450000	-0.3556560000
C	-2.3959130000	3.2736580000	-1.2219230000
H	-2.4374570000	1.2760350000	-0.4512250000
H	-2.9867700000	2.4751150000	0.7008900000
H	-1.3298390000	3.3438370000	-0.9908620000
H	-2.4800640000	3.0228320000	-2.2847940000
H	-2.8697980000	4.2503920000	-1.0698200000
C	-3.9893860000	-0.4295060000	-1.4985430000
O	-3.4675920000	-0.9759440000	-0.5302010000
N	-3.9105470000	-0.9468190000	-2.7871950000
C	-3.4652720000	-0.2215540000	-4.0329430000
C	-4.3337980000	1.0211990000	-4.2683380000
C	-3.6779390000	-1.2002930000	-5.1976820000
H	-4.0495140000	1.4305980000	-5.2428790000
H	-5.3987500000	0.7776740000	-4.2952800000
H	-4.1732510000	1.7966950000	-3.5190860000
H	-3.0566780000	-2.0901390000	-5.0875320000
H	-4.7276000000	-1.5061020000	-5.2603240000
H	-3.4039840000	-0.7034560000	-6.1333130000
F	-3.1524680000	-2.1421340000	-2.7272800000
C	-1.9875160000	0.1656180000	-3.8964290000
H	-1.8247170000	0.8289680000	-3.0413800000
H	-1.3506510000	-0.7109460000	-3.7529670000
H	-1.6656180000	0.6849000000	-4.8062530000
O	-1.2566540000	-2.5781640000	-0.5447840000
H	-1.0383850000	-2.8911200000	-1.4461180000
H	-2.1445220000	-2.1503500000	-0.6108060000
N	2.3803370000	-2.1026390000	-3.8283300000
N	1.3848920000	-2.0105920000	-3.2294410000
N	0.3580120000	-1.9217880000	-2.6085160000

$^5Im_2WatOHN_3$_13
Charge: 0
Multiplicity: 5
B3LYP-D3(BJ)/6-31G(d)-SDD SCF energy (au): -1629.10744341
B3LYP-D3(BJ)/6-31G(d)-SDD enthalpy (au): -1628.58240141
B3LYP-D3(BJ)/6-31G(d)-SDD free energy (au): -1628.69489141
B3LYP-D3(BJ)/def2-TZVP/SMD SCF energy (au): -2769.61332194
B3LYP-D3(BJ)/def2-TZVP/SMD enthalpy (au): -2769.08827994

B3LYP-D3(BJ)/def2-TZVP/SMD free energy (au): -2769.20076994
B3LYP-D3(BJ)/def2-TZVP/SMD free energy (quasi-harmonic) (au): -2769.19001753

Cartesian coordinates

ATOM	X	Y	Z
H	32.4424500000	29.0907250000	38.1211620000
C	33.0201550000	29.1979380000	37.2168630000
C	34.2930280000	29.6449420000	36.9840180000
N	32.5080990000	28.8731390000	35.9713890000
C	33.4623250000	29.1303660000	35.0414070000
N	34.5506070000	29.5949930000	35.6299630000
H	31.5734920000	28.5466140000	35.7768000000
H	35.0326700000	30.0108350000	37.6777480000
H	33.3568150000	28.9772910000	33.9791290000
H	39.1430870000	26.8079350000	37.5028400000
C	38.5462240000	27.2695560000	36.7319700000
C	37.9957420000	28.5181370000	36.6137240000
N	38.1896360000	26.5968180000	35.5743660000
C	37.4520950000	27.4406080000	34.8068320000
N	37.3207750000	28.6041020000	35.4151340000
H	38.4430420000	25.6502500000	35.3337210000
H	38.0314360000	29.3638410000	37.2818750000
H	37.0273620000	27.2132550000	33.8407490000
Fe	36.2663040000	30.2772370000	34.4206630000
O	35.7572240000	29.0868780000	33.0456160000
H	36.0258150000	29.4786460000	32.1985310000
F	37.8687440000	31.0359420000	33.9089780000
N	33.4843700000	34.5014870000	36.0338270000
O	34.8953850000	33.0335750000	37.0425830000
C	35.8534800000	34.5894250000	35.5022390000
C	37.0369090000	34.6711150000	36.2371240000
C	38.1835070000	35.2708770000	35.7159820000
C	38.1455590000	35.8038810000	34.4214620000
C	36.9877380000	35.7221590000	33.6675220000
C	35.8027260000	35.0997370000	34.1587650000
C	34.7055430000	33.9605300000	36.2296080000
C	32.2253090000	34.0988690000	36.7008050000
C	31.9394340000	32.6095610000	36.4563610000
C	32.3265190000	34.3955140000	38.2062970000
C	31.1214330000	34.9502250000	36.0601380000
C	34.6829060000	34.9446910000	33.3062700000
C	34.6348310000	35.4262310000	31.8910830000
H	37.0423550000	34.2642100000	37.2430980000
H	39.0891160000	35.3275140000	36.3118870000
H	39.0296780000	36.2736650000	33.9995920000
H	36.9892390000	36.1093490000	32.6548370000
H	33.4434440000	35.3128640000	35.4327290000
H	33.8568760000	34.3270180000	33.6315500000
H	35.3035200000	34.8338380000	31.2471690000
H	33.6243710000	35.3269590000	31.4850790000
H	34.9419150000	36.4753430000	31.7880160000
H	30.9742960000	32.3497220000	36.9079320000
H	31.8986420000	32.3928770000	35.3850260000

H	32.7146440000	31.9881090000	36.9048060000
H	33.1463080000	33.8242930000	38.6475700000
H	32.5074120000	35.4622800000	38.3804350000
H	31.3927300000	34.1181720000	38.7083810000
H	31.0453610000	34.7439840000	34.9865310000
H	30.1560830000	34.7184070000	36.5198250000
H	31.3118260000	36.0217320000	36.2018140000
N	35.1184790000	31.8834780000	33.8878090000
N	33.9679310000	31.8020900000	33.5519390000
N	32.8508100000	31.7771830000	33.2273840000
O	36.8584620000	31.3443350000	36.3268810000
H	36.1594410000	32.0083830000	36.5774400000
H	37.5241200000	31.8285170000	35.8032180000

$^5Im_2WatOHN_3$_FeF_remove-F

Charge: 0
Multiplicity: 5
B3LYP-D3(BJ)/6-31G(d)-SDD SCF energy (au): -892.967172174
B3LYP-D3(BJ)/6-31G(d)-SDD enthalpy (au): -892.750210174
B3LYP-D3(BJ)/6-31G(d)-SDD free energy (au): -892.824324174
B3LYP-D3(BJ)/def2-TZVP/SMD SCF energy (au): -2033.17228999
B3LYP-D3(BJ)/def2-TZVP/SMD enthalpy (au): -2032.95532799
B3LYP-D3(BJ)/def2-TZVP/SMD free energy (au): -2033.02944199
B3LYP-D3(BJ)/def2-TZVP/SMD free energy (quasi-harmonic) (au): -2033.024075816

Cartesian coordinates

ATOM	X	Y	Z
Fe	0.0865390000	-0.9413600000	1.4322710000
C	-1.0592410000	-1.1035090000	-2.7777900000
N	-2.2177710000	-1.4324260000	-2.0940420000
H	-3.1090350000	-1.6624280000	-2.5074600000
C	-1.9487350000	-1.3877460000	-0.7639540000
H	-2.6513910000	-1.6034680000	0.0275640000
N	-0.6852150000	-1.0498000000	-0.5689000000
C	-0.1136340000	-0.8690470000	-1.8150480000
H	0.9284470000	-0.5923280000	-1.9074000000
H	-1.0209520000	-1.0674710000	-3.8551530000
C	2.1820590000	-4.6865220000	1.8794830000
N	3.0831850000	-4.0079660000	1.0774870000
H	3.9642620000	-4.3643220000	0.7381720000
C	2.5836260000	-2.7705250000	0.8235610000
H	3.0648270000	-2.0212690000	0.2047590000
N	1.4116120000	-2.6295650000	1.4236840000
C	1.1475060000	-3.8141390000	2.0851110000
H	0.2341720000	-3.9332440000	2.6480410000
H	2.3603510000	-5.6946240000	2.2188520000
O	-1.4345420000	-1.5589730000	2.3226920000
H	-1.7132690000	-1.0747450000	3.1127900000
O	2.9220790000	-0.2263590000	-0.9169400000
H	2.4121240000	0.1543500000	-0.1562910000
H	3.5191540000	0.4866240000	-1.1882750000
N	1.3885230000	0.5753580000	1.3008880000
N	1.9113850000	1.2234870000	2.1878120000

N   2.4286430000   1.8658910000   2.9928020000

[5]Im2WatOHN3_FeF_remove-N3

Charge: 0
Multiplicity: 5
B3LYP-D3(BJ)/6-31G(d)-SDD SCF energy (au): -828.633665982
B3LYP-D3(BJ)/6-31G(d)-SDD enthalpy (au): -828.429699982
B3LYP-D3(BJ)/6-31G(d)-SDD free energy (au): -828.497182982
B3LYP-D3(BJ)/def2-TZVP/SMD SCF energy (au): -1968.81692155
B3LYP-D3(BJ)/def2-TZVP/SMD enthalpy (au): -1968.61295555
B3LYP-D3(BJ)/def2-TZVP/SMD free energy (au): -1968.68043855
B3LYP-D3(BJ)/def2-TZVP/SMD free energy (quasi-harmonic) (au): -1968.677087568

Cartesian coordinates

ATOM	X	Y	Z
Fe	-0.0458780000	-0.9294730000	1.3099980000
C	-2.3589010000	-2.0961380000	-2.1534760000
N	-1.3710130000	-1.3530350000	-2.7771460000
H	-1.2901750000	-1.1837310000	-3.7685530000
C	-0.5251710000	-0.8816210000	-1.8225560000
H	0.3551780000	-0.2771350000	-2.0111860000
N	-0.9265660000	-1.2868430000	-0.6288140000
C	-2.0663090000	-2.0442740000	-0.8169740000
H	-2.5711320000	-2.4848500000	0.0295050000
H	-3.1524590000	-2.5743110000	-2.7057690000
C	3.0227890000	-3.9716010000	1.4785060000
N	3.6179460000	-3.0958770000	0.5862940000
H	4.5195360000	-3.2134130000	0.1488040000
C	2.7806840000	-2.0404950000	0.4017720000
H	2.9717390000	-1.1982530000	-0.2540890000
N	1.6884830000	-2.2026140000	1.1305150000
C	1.8228370000	-3.3996860000	1.8065410000
H	1.0403770000	-3.7405930000	2.4675920000
H	3.4987890000	-4.8893430000	1.7860420000
F	0.7649800000	0.7379930000	0.9722920000
O	-1.1460480000	-1.9427020000	2.4262910000
H	-1.5369190000	-1.5060080000	3.1956740000
O	2.1701250000	0.5586280000	-1.2066800000
H	2.5854000000	1.4178590000	-1.3704690000
H	1.6500100000	0.6915720000	-0.3653290000

[5]Im2WatOHN3_FeF_remove-OH

Charge: 0
Multiplicity: 5
B3LYP-D3(BJ)/6-31G(d)-SDD SCF energy (au): -917.015831410
B3LYP-D3(BJ)/6-31G(d)-SDD enthalpy (au): -916.809594410
B3LYP-D3(BJ)/6-31G(d)-SDD free energy (au): -916.882425410
B3LYP-D3(BJ)/def2-TZVP/SMD SCF energy (au): -2057.23153675
B3LYP-D3(BJ)/def2-TZVP/SMD enthalpy (au): -2057.02529975
B3LYP-D3(BJ)/def2-TZVP/SMD free energy (au): -2057.09813075
B3LYP-D3(BJ)/def2-TZVP/SMD free energy (quasi-harmonic) (au): -2057.092275340

Cartesian coordinates

```
ATOM     X                Y                Z
Fe     0.7042400000   -0.1967990000    0.8762950000
C     -2.6147420000   -2.1907250000   -1.1141540000
N     -2.1082350000   -1.3712630000   -2.1071650000
H     -2.4678520000   -1.2815530000   -3.0459810000
C     -1.0477740000   -0.6951900000   -1.5967120000
H     -0.4545370000    0.0265550000   -2.1376860000
N     -0.8527010000   -1.0434480000   -0.3360170000
C     -1.8230660000   -1.9757730000   -0.0177740000
H     -1.8833540000   -2.4094870000    0.9700180000
H     -3.4685690000   -2.8292860000   -1.2761740000
C      3.0130470000   -3.8173580000    1.3140800000
N      3.8278350000   -3.1076230000    0.4500760000
H      4.7042820000   -3.4238130000    0.0622520000
C      3.2547110000   -1.9012000000    0.2094390000
H      3.6668660000   -1.1212500000   -0.4288140000
N      2.1124900000   -1.8098370000    0.8776430000
C      1.9488790000   -2.9974410000    1.5719760000
H      1.0907320000   -3.1715220000    2.2046910000
H      3.2615350000   -4.8090250000    1.6574510000
F      1.2849180000    1.0383840000   -0.3929250000
O      3.7501460000    0.6837180000   -1.3227120000
H      4.2402680000    1.3134990000   -0.7730470000
H      2.8084580000    0.8621000000   -1.0856330000
N     -0.0445340000   -0.3473790000    2.6928470000
N     -0.5709800000   -1.3646960000    3.0796350000
N     -1.0767390000   -2.3320800000    3.4728450000
```

$^{6}Im_2WatOHN_3$_FeF

```
Charge: 0
Multiplicity: 6
B3LYP-D3(BJ)/6-31G(d)-SDD SCF energy (au):              -992.832574410
B3LYP-D3(BJ)/6-31G(d)-SDD enthalpy (au):                -992.611118410
B3LYP-D3(BJ)/6-31G(d)-SDD free energy (au):             -992.685312410
B3LYP-D3(BJ)/def2-TZVP/SMD SCF energy (au):             -2133.08149357
B3LYP-D3(BJ)/def2-TZVP/SMD enthalpy (au):               -2132.86003757
B3LYP-D3(BJ)/def2-TZVP/SMD free energy (au):            -2132.93423157
B3LYP-D3(BJ)/def2-TZVP/SMD free energy (quasi-harmonic) (au): -2132.929378160

Cartesian coordinates
ATOM     X                Y                Z
Fe     0.2441180000   -0.3154020000    1.0676530000
C     -1.5058930000   -2.5584460000   -2.3150320000
N     -1.7954970000   -1.2376550000   -2.6134210000
H     -2.3372290000   -0.9092510000   -3.3993020000
C     -1.2325030000   -0.4551930000   -1.6605110000
H     -1.2929320000    0.6205010000   -1.6150620000
N     -0.5970070000   -1.2053860000   -0.7760320000
C     -0.7598270000   -2.5180640000   -1.1683680000
H     -0.3369940000   -3.3280370000   -0.5952530000
H     -1.8514420000   -3.3753620000   -2.9285780000
C      2.2770370000   -4.1029110000    1.5827830000
N      3.1307050000   -3.4879590000    0.6830600000
```

H	4.0054180000	-3.8610890000	0.3442890000
C	2.6188250000	-2.2725590000	0.3693310000
H	3.0607280000	-1.5543290000	-0.3050000000
N	1.4810430000	-2.0896570000	1.0179080000
C	1.2530170000	-3.2166960000	1.7827180000
H	0.3791820000	-3.2801120000	2.4121060000
H	2.4818990000	-5.0784880000	1.9939210000
F	-0.1635710000	1.3024140000	0.2287750000
O	-1.0604540000	-0.9942100000	2.1827560000
H	-1.0681180000	-0.5627280000	3.0525100000
O	2.3323260000	0.6437480000	-0.4047520000
H	2.5658820000	0.8553600000	0.5202400000
H	1.5088840000	1.1634920000	-0.5165790000
N	1.5763590000	0.4700550000	2.3761720000
N	2.1803300000	-0.2327010000	3.1621070000
N	2.7743910000	-0.8777520000	3.9125620000

$^5Im_2WatOHN_3$_TS-1
Charge: 0
Multiplicity: 5
B3LYP-D3(BJ)/6-31G(d)-SDD SCF energy (au): -1629.01692323
B3LYP-D3(BJ)/6-31G(d)-SDD enthalpy (au): -1628.49323323
B3LYP-D3(BJ)/6-31G(d)-SDD free energy (au): -1628.60409323
B3LYP-D3(BJ)/def2-TZVP/SMD SCF energy (au): -2769.51613764
B3LYP-D3(BJ)/def2-TZVP/SMD enthalpy (au): -2768.99244764
B3LYP-D3(BJ)/def2-TZVP/SMD free energy (au): -2769.10330764
B3LYP-D3(BJ)/def2-TZVP/SMD free energy (quasi-harmonic) (au): -2769.09345541

Cartesian coordinates

ATOM	X	Y	Z
Fe	-0.2740400000	-0.3815460000	0.8826810000
C	0.8902540000	-1.6643240000	-3.2748880000
N	-0.4362860000	-1.2809170000	-3.3672020000
H	-1.0204500000	-1.3522220000	-4.1871800000
C	-0.8255700000	-0.8080750000	-2.1532750000
H	-1.8215960000	-0.4794550000	-1.8974470000
N	0.1784520000	-0.8649340000	-1.2976360000
C	1.2525840000	-1.4006140000	-1.9796510000
H	2.1969400000	-1.5799380000	-1.4867300000
H	1.4295400000	-2.0779200000	-4.1125270000
C	0.8402240000	-4.5945780000	1.4039860000
N	1.6112720000	-3.9775430000	2.3742550000
H	2.2112880000	-4.4375230000	3.0427950000
C	1.4057370000	-2.6368150000	2.2899940000
H	1.8667010000	-1.9106180000	2.9448430000
N	0.5491010000	-2.3711790000	1.3191180000
C	0.1869930000	-3.5780290000	0.7586150000
H	-0.5206010000	-3.6151350000	-0.0552070000
H	0.8301830000	-5.6652430000	1.2737140000
C	0.7227760000	7.1795560000	1.8879460000
C	0.5627830000	5.8850920000	1.3980600000
C	0.6233210000	4.7843690000	2.2596150000
C	0.8825100000	4.9550700000	3.6352200000

C	1.0158230000	6.2667460000	4.1052470000
C	0.9361940000	7.3687220000	3.2526390000
H	0.6804940000	8.0280560000	1.2114210000
H	0.3930020000	5.7157880000	0.3388340000
H	1.2001540000	6.4212250000	5.1653680000
H	1.0545350000	8.3714690000	3.6538370000
C	0.9972870000	3.7914890000	4.5963300000
C	-0.3489430000	3.1235520000	4.9168040000
H	1.6734950000	3.0360400000	4.1882400000
H	1.4474560000	4.1589830000	5.5257200000
H	-0.2047640000	2.3336280000	5.6599960000
H	-0.7757690000	2.6493120000	4.0289810000
H	-1.0714750000	3.8491130000	5.3093930000
C	0.5449000000	3.4061260000	1.6647490000
O	1.5319890000	2.6687840000	1.7630210000
N	-0.5554910000	3.1433450000	0.9184020000
C	-2.0138460000	3.3832280000	0.9789850000
C	-2.3365710000	4.6560400000	1.7873750000
C	-2.4920620000	3.5606000000	-0.4706090000
H	-3.4268040000	4.7538160000	1.7957420000
H	-1.9142500000	5.5569210000	1.3420040000
H	-1.9922170000	4.5819470000	2.8206030000
H	-2.2608170000	2.6678300000	-1.0558550000
H	-2.0072350000	4.4252050000	-0.9366100000
H	-3.5756330000	3.7170210000	-0.4857820000
F	-0.3593090000	1.6844020000	0.2227640000
C	-2.7305860000	2.1918310000	1.6384260000
H	-2.3024910000	1.9679850000	2.6176020000
H	-2.6569910000	1.2915230000	1.0254190000
H	-3.7882880000	2.4507520000	1.7623060000
O	-2.0714570000	-0.8005780000	0.4284790000
H	-2.5634940000	-0.9392180000	1.2527900000
O	1.8855360000	0.2197730000	0.8285920000
H	1.7908330000	1.1265630000	1.2354320000
H	1.9964500000	0.4059500000	-0.1171120000
N	1.4259350000	0.1139470000	4.4163780000
N	0.5641000000	0.1622260000	3.6355480000
N	-0.3450270000	0.2193550000	2.8489850000

[5]Im$_2$WatOHN$_3$_TS-3-F
Charge: 0
Multiplicity: 5
B3LYP-D3(BJ)/6-31G(d)-SDD SCF energy (au): -1629.07821112
B3LYP-D3(BJ)/6-31G(d)-SDD enthalpy (au): -1628.55356812
B3LYP-D3(BJ)/6-31G(d)-SDD free energy (au): -1628.66590712
B3LYP-D3(BJ)/def2-TZVP/SMD SCF energy (au): -2769.59243396
B3LYP-D3(BJ)/def2-TZVP/SMD enthalpy (au): -2769.06779096
B3LYP-D3(BJ)/def2-TZVP/SMD free energy (au): -2769.18012996
B3LYP-D3(BJ)/def2-TZVP/SMD free energy (quasi-harmonic) (au): -2769.16876084

Cartesian coordinates

ATOM	X	Y	Z
H	34.5516760000	26.9842370000	39.2252420000

C	34.3047120000	27.5905760000	38.3681030000
C	34.7204640000	27.5580830000	37.0642830000
N	33.3902090000	28.6285040000	38.4359190000
C	33.2857320000	29.1803250000	37.2007210000
N	34.0782770000	28.5509360000	36.3503290000
H	32.9017710000	28.9414290000	39.2613950000
H	35.4070160000	26.8770520000	36.5872620000
H	32.6703630000	30.0274330000	36.9422620000
H	39.1377580000	30.2797810000	36.7022730000
C	38.3607320000	29.9237710000	36.0442270000
C	37.0610940000	30.3143290000	35.8610350000
N	38.5689660000	28.8687170000	35.1674060000
C	37.4109190000	28.6478680000	34.4949200000
N	36.4920400000	29.5137040000	34.8875000000
H	39.4177280000	28.3312110000	35.0708520000
H	36.4640410000	31.0829320000	36.3433530000
H	37.2534760000	27.8695050000	33.7630160000
Fe	34.4389190000	29.3806530000	34.2626490000
O	33.3181210000	30.7671190000	34.8716070000
H	32.9983820000	31.3862870000	34.1875040000
F	34.6567560000	30.0659130000	32.4558550000
N	33.4631360000	33.1028500000	30.8315420000
O	32.5252450000	32.7007390000	32.8715190000
C	34.8585230000	33.1881810000	32.8143500000
C	34.9373010000	34.1743420000	33.7954930000
C	36.1266690000	34.3999630000	34.4928030000
C	37.2372810000	33.5872340000	34.2364760000
C	37.1606750000	32.5785080000	33.2872910000
C	35.9849510000	32.3611880000	32.5305010000
C	33.5123260000	32.9659040000	32.1792180000
C	32.2955490000	32.7870330000	29.9753580000
C	31.8644850000	31.3304340000	30.2131100000
C	31.1445640000	33.7569280000	30.2862190000
C	32.7665470000	32.9682680000	28.5271740000
C	35.8968160000	31.2848240000	31.5799120000
C	37.0251810000	30.3555180000	31.2834520000
H	34.0511340000	34.7614550000	34.0163850000
H	36.1820590000	35.1897890000	35.2361620000
H	38.1596930000	33.7354160000	34.7902030000
H	38.0234880000	31.9443600000	33.1191600000
H	34.3048640000	33.4219930000	30.3725150000
H	35.1576970000	31.3804850000	30.7982890000
H	37.5444680000	30.0424270000	32.1915210000
H	36.6424020000	29.4618860000	30.7845940000
H	37.7603890000	30.8312690000	30.6159630000
H	31.0386620000	31.0741200000	29.5404100000
H	32.6961460000	30.6439780000	30.0233860000
H	31.5369990000	31.1882130000	31.2440750000
H	30.8416650000	33.6592430000	31.3304880000
H	31.4528010000	34.7925480000	30.1035450000
H	30.2835580000	33.5351830000	29.6459270000
H	33.5872570000	32.2804980000	28.2896400000
H	31.9436010000	32.7595660000	27.8374690000

H	33.1052920000	33.9959360000	28.3455400000
N	34.5633160000	27.5086060000	33.5333090000
N	34.3921720000	26.4498480000	34.0914720000
N	34.2554510000	25.4109630000	34.5847760000
O	34.7320820000	32.2045770000	36.6781000000
H	35.0185510000	32.9527020000	36.1322270000
H	34.1632420000	31.6837510000	36.0469750000

[5]Im$_2$WatOHN$_3$_TS-3-N$_3$

Charge: 0
Multiplicity: 5
B3LYP-D3(BJ)/6-31G(d)-SDD SCF energy (au): -1629.09315762
B3LYP-D3(BJ)/6-31G(d)-SDD enthalpy (au): -1628.56910662
B3LYP-D3(BJ)/6-31G(d)-SDD free energy (au): -1628.67823962
B3LYP-D3(BJ)/def2-TZVP/SMD SCF energy (au): -2769.59759527
B3LYP-D3(BJ)/def2-TZVP/SMD enthalpy (au): -2769.07354427
B3LYP-D3(BJ)/def2-TZVP/SMD free energy (au): -2769.18267727
B3LYP-D3(BJ)/def2-TZVP/SMD free energy (quasi-harmonic) (au): -2769.17356065

Cartesian coordinates

ATOM	X	Y	Z
H	32.2485150000	29.0967350000	38.5298990000
C	32.8013170000	29.1882470000	37.6082990000
C	33.9894600000	29.7980780000	37.3027230000
N	32.3505650000	28.6383080000	36.4201610000
C	33.2536930000	28.9344080000	35.4490090000
N	34.2559770000	29.6280910000	35.9599270000
H	31.4798820000	28.1474090000	36.2834560000
H	34.6551840000	30.3616450000	37.9367650000
H	33.1707840000	28.6689750000	34.4056640000
H	39.3626430000	26.8577750000	36.4035540000
C	38.6036030000	27.3915180000	35.8533660000
C	37.9746790000	28.5888400000	36.0711200000
N	38.0642730000	26.8987290000	34.6758670000
C	37.1468880000	27.7955670000	34.2266920000
N	37.0750290000	28.8219450000	35.0528560000
H	38.3145000000	26.0326870000	34.2229080000
H	38.0923570000	29.3068060000	36.8672690000
H	36.5524320000	27.7003030000	33.3304980000
Fe	35.7528110000	30.5254250000	34.6103930000
O	35.0065850000	29.5762020000	33.1367840000
H	35.2157630000	30.0589240000	32.3219280000
F	37.1737480000	31.6156840000	33.9625790000
N	33.7241630000	34.7434180000	36.7731860000
O	35.0237450000	33.1339850000	37.7353860000
C	36.0304420000	34.4816420000	36.0496130000
C	37.2193780000	34.7546620000	36.7332190000
C	38.3880480000	35.0738360000	36.0467340000
C	38.3772040000	35.0718860000	34.6472120000
C	37.2159180000	34.7598390000	33.9574110000
C	36.0026630000	34.4782770000	34.6262630000
C	34.8786220000	34.0514010000	36.9080900000
C	32.4935690000	34.5555670000	37.5768290000

C   31.9738600000   33.1178530000   37.4280040000
C   32.7904130000   34.8773670000   39.0512870000
C   31.4632900000   35.5417690000   37.0127270000
C   34.8222540000   34.1014610000   33.8787810000
C   34.8787380000   33.8524680000   32.4030630000
H   37.2172400000   34.7007870000   37.8177200000
H   39.2986010000   35.3000910000   36.5930510000
H   39.2853820000   35.2951940000   34.0946590000
H   37.2382890000   34.7238440000   32.8748020000
H   33.7646680000   35.5640510000   36.1845170000
H   33.8639270000   34.4263030000   34.2686590000
H   35.6984690000   33.1673890000   32.1668780000
H   33.9401940000   33.4089270000   32.0563920000
H   35.0307350000   34.7844310000   31.8366030000
H   31.0702340000   32.9948910000   38.0363700000
H   31.7209690000   32.8973190000   36.3874740000
H   32.7217610000   32.3978090000   37.7611490000
H   33.5519350000   34.1973990000   39.4393630000
H   33.1511860000   35.9067720000   39.1577070000
H   31.8805150000   34.7679390000   39.6518830000
H   31.2555750000   35.3285360000   35.9578340000
H   30.5233740000   35.4583180000   37.5663090000
H   31.8134500000   36.5781890000   37.1010660000
N   34.4478700000   32.1943680000   34.5982810000
N   33.2727800000   31.9731500000   34.3782190000
N   32.1487430000   31.8089510000   34.1691060000
O   36.7579500000   31.3497360000   36.5136680000
H   36.1984360000   32.0128040000   36.9848270000
H   37.2660570000   31.8471840000   35.8367670000

$^5Im_2WatOHN_3$_TS-3-OH
Charge: 0
Multiplicity: 5
B3LYP-D3(BJ)/6-31G(d)-SDD SCF energy (au): -1629.09196123
B3LYP-D3(BJ)/6-31G(d)-SDD enthalpy (au): -1628.56780423
B3LYP-D3(BJ)/6-31G(d)-SDD free energy (au): -1628.67675223
B3LYP-D3(BJ)/def2-TZVP/SMD SCF energy (au): -2769.59671465
B3LYP-D3(BJ)/def2-TZVP/SMD enthalpy (au): -2769.07255765
B3LYP-D3(BJ)/def2-TZVP/SMD free energy (au): -2769.18150565
B3LYP-D3(BJ)/def2-TZVP/SMD free energy (quasi-harmonic) (au): -2769.17243342

Cartesian coordinates
ATOM   X   Y   Z
H   32.9095350000   28.9017090000   39.1798000000
C   33.2167940000   29.0994710000   38.1650650000
C   34.4104880000   29.4980910000   37.6230630000
N   32.3501580000   28.9723350000   37.0945380000
C   33.0310610000   29.2909580000   35.9611030000
N   34.2792540000   29.6114900000   36.2544670000
H   31.3796590000   28.6994760000   37.1406190000
H   35.3466620000   29.7243670000   38.1075360000
H   32.5812520000   29.3070710000   34.9783670000
H   39.1100670000   26.5652860000   36.4185160000

C	38.3713220000	27.1497520000	35.8931210000
C	37.8087320000	28.3717360000	36.1486790000
N	37.7890800000	26.7139760000	34.7145190000
C	36.9125300000	27.6653630000	34.3004060000
N	36.9044020000	28.6738280000	35.1516200000
H	37.9905570000	25.8497140000	34.2342860000
H	37.9811490000	29.0642300000	36.9574320000
H	36.3116920000	27.6142200000	33.4050150000
Fe	35.7982790000	30.5469480000	34.8275600000
O	34.6225600000	32.0490560000	34.8498110000
H	33.7004660000	31.8201700000	34.6486110000
F	37.3575400000	31.3974800000	34.2431270000
N	33.7425540000	34.5880980000	36.7138580000
O	35.1215880000	33.1159690000	37.7633270000
C	36.0619170000	34.4183910000	35.9996270000
C	37.2578540000	34.7167880000	36.6585340000
C	38.4125460000	35.0438150000	35.9510500000
C	38.3795810000	35.0273100000	34.5516180000
C	37.2127060000	34.6925970000	33.8832190000
C	36.0117400000	34.3942170000	34.5722970000
C	34.9354390000	33.9724570000	36.8785990000
C	32.4755580000	34.2133890000	37.3783180000
C	32.1306760000	32.7478710000	37.0614260000
C	32.5977800000	34.4254330000	38.8963310000
C	31.3983080000	35.1413530000	36.8030260000
C	34.8486540000	33.9744440000	33.8429370000
C	34.8961290000	33.5725310000	32.4049160000
H	37.2722670000	34.6771510000	37.7437110000
H	39.3276880000	35.2904580000	36.4807770000
H	39.2755250000	35.2602330000	33.9828900000
H	37.2159710000	34.6504110000	32.7999010000
H	33.7080200000	35.3332880000	36.0331450000
H	33.8724370000	34.1655020000	34.2655120000
H	35.7465810000	32.9084610000	32.2190100000
H	33.9830310000	33.0360210000	32.1308960000
H	34.9876080000	34.4402410000	31.7311820000
H	31.2025560000	32.4686890000	37.5736100000
H	31.9817570000	32.6138920000	35.9851740000
H	32.9312900000	32.0830660000	37.3887560000
H	33.3831580000	33.7868570000	39.3039830000
H	32.8446870000	35.4695950000	39.1177130000
H	31.6480350000	34.1839090000	39.3872060000
H	31.3028380000	35.0105160000	35.7182240000
H	30.4278730000	34.9164730000	37.2555370000
H	31.6314500000	36.1928890000	37.0097920000
N	34.9073940000	29.6146830000	33.2004690000
N	33.7958160000	29.9901410000	32.9297330000
N	32.7128720000	30.3471240000	32.6939430000
O	36.7916040000	31.2220030000	36.7591780000
H	36.2268370000	31.9354930000	37.1610350000
H	37.4110070000	31.6792230000	36.1532460000

$^5Im_2Wat_2N_3$_11

Charge: 1
Multiplicity: 5
B3LYP-D3(BJ)/6-31G(d)-SDD SCF energy (au): -1629.47073885
B3LYP-D3(BJ)/6-31G(d)-SDD enthalpy (au): -1628.93062285
B3LYP-D3(BJ)/6-31G(d)-SDD free energy (au): -1629.04444785
B3LYP-D3(BJ)/def2-TZVP/SMD SCF energy (au): -2770.00736913
B3LYP-D3(BJ)/def2-TZVP/SMD enthalpy (au): -2769.46725313
B3LYP-D3(BJ)/def2-TZVP/SMD free energy (au): -2769.58107813
B3LYP-D3(BJ)/def2-TZVP/SMD free energy (quasi-harmonic) (au): -2769.56939928

Cartesian coordinates

ATOM	X	Y	Z
Fe	0.4164240000	-0.5069930000	-0.4839440000
C	-0.4352850000	1.4290570000	3.3657690000
N	0.6445410000	2.1345630000	2.8699130000
H	1.0789520000	2.9378140000	3.3027840000
C	1.0186800000	1.5716840000	1.6939990000
H	1.8152700000	1.9296630000	1.0592760000
N	0.2342060000	0.5412810000	1.4136520000
C	-0.6791830000	0.4416860000	2.4475470000
H	-1.4537960000	-0.3092370000	2.4392520000
H	-0.9164100000	1.6902190000	4.2951410000
C	3.2052290000	-3.6712660000	0.3192830000
N	2.8007430000	-3.3537210000	1.6026290000
H	3.1599780000	-3.7617960000	2.4548440000
C	1.8495140000	-2.3946570000	1.5250750000
H	1.3669610000	-1.9441610000	2.3791020000
N	1.6155500000	-2.0869170000	0.2558150000
C	2.4595680000	-2.8769100000	-0.5079400000
H	2.4829160000	-2.7900210000	-1.5832790000
H	3.9691140000	-4.4067120000	0.1224990000
O	0.3022350000	-1.5457560000	-2.4505490000
H	-0.4024980000	-1.0156840000	-2.8968620000
H	0.0753120000	-2.4792240000	-2.5809000000
C	-5.5685140000	2.5709640000	-2.7543270000
C	-4.4574390000	1.9180270000	-3.2787190000
C	-3.2513620000	1.9068240000	-2.5670520000
C	-3.1338250000	2.5303590000	-1.3131790000
C	-4.2675640000	3.1800490000	-0.8079390000
C	-5.4685680000	3.2053840000	-1.5141950000
H	-6.5031770000	2.5828210000	-3.3056410000
H	-4.5174310000	1.4160440000	-4.2399580000
H	-4.2058820000	3.6737210000	0.1577490000
H	-6.3294810000	3.7172360000	-1.0950150000
C	-1.8380460000	2.5286630000	-0.5284240000
C	-1.3054610000	3.9341130000	-0.2130010000
H	-1.0581090000	1.9831770000	-1.0678060000
H	-1.9902660000	1.9848220000	0.4118850000
H	-0.3606330000	3.8612000000	0.3351910000
H	-1.1187290000	4.5027520000	-1.1300540000
H	-2.0086450000	4.5095810000	0.3974120000
C	-2.1195620000	1.1019600000	-3.1114980000
O	-1.9119190000	-0.0572760000	-2.6834630000

N	-1.3836680000	1.6013070000	-4.1205460000
C	-1.0304780000	2.9572550000	-4.6641010000
C	-2.1475900000	3.9732730000	-4.4027660000
C	-0.8456170000	2.7857530000	-6.1801410000
H	-1.8423930000	4.9036620000	-4.8905910000
H	-3.1005920000	3.6612030000	-4.8340630000
H	-2.2926680000	4.1805460000	-3.3423510000
H	-0.0484170000	2.0769740000	-6.4093770000
H	-1.7733120000	2.4416910000	-6.6480680000
H	-0.5789670000	3.7537000000	-6.6135100000
F	-0.3743640000	0.7019470000	-4.4842920000
C	0.2745590000	3.4046480000	-3.9891150000
H	0.1496310000	3.4660730000	-2.9037870000
H	1.0930890000	2.7164610000	-4.2069390000
H	0.5478890000	4.3958010000	-4.3634690000
O	-1.6911850000	-1.0033380000	-0.2381920000
H	-1.9030700000	-1.9504570000	-0.2643090000
H	-2.0565980000	-0.6218530000	-1.0843170000
N	2.6547410000	0.9853620000	-3.2828160000
N	2.0206010000	0.9657710000	-2.3224490000
N	1.3554580000	1.0058190000	-1.3036040000

$^{5}Im_2Wat_2N_3$_13

Charge: 1
Multiplicity: 5
B3LYP-D3(BJ)/6-31G(d)-SDD SCF energy (au): -1629.53845012
B3LYP-D3(BJ)/6-31G(d)-SDD enthalpy (au): -1628.99961012
B3LYP-D3(BJ)/6-31G(d)-SDD free energy (au): -1629.11278012
B3LYP-D3(BJ)/def2-TZVP/SMD SCF energy (au): -2770.07343488
B3LYP-D3(BJ)/def2-TZVP/SMD enthalpy (au): -2769.53459488
B3LYP-D3(BJ)/def2-TZVP/SMD free energy (au): -2769.64776488
B3LYP-D3(BJ)/def2-TZVP/SMD free energy (quasi-harmonic) (au): -2769.63701376

Cartesian coordinates

ATOM	X	Y	Z
H	33.4033180000	26.6164660000	38.0126420000
C	33.5899820000	27.4377980000	37.3392870000
C	34.7455910000	28.0402430000	36.9266870000
N	32.5695630000	28.1147060000	36.6981990000
C	33.1103750000	29.0845780000	35.9289530000
N	34.4348910000	29.0665840000	36.0493780000
H	31.5821900000	27.9123920000	36.7736990000
H	35.7661990000	27.8156830000	37.1899110000
H	32.5377040000	29.7619180000	35.3109760000
H	39.9780710000	27.2139440000	36.4704520000
C	39.1554590000	27.6459140000	35.9229140000
C	38.2259880000	28.5909190000	36.2600600000
N	38.8618960000	27.2807700000	34.6221060000
C	37.7893720000	27.9911110000	34.2089480000
N	37.3782930000	28.7917740000	35.1836960000
H	39.3684370000	26.6050470000	34.0663660000
H	38.1011470000	29.1414790000	37.1787210000
H	37.3472250000	27.9259810000	33.2270740000

Fe	35.8517670000	30.2552010000	34.9147350000
O	34.4666400000	31.8707970000	35.2310040000
H	34.8795040000	32.5657880000	34.6838090000
H	33.5512140000	31.7385230000	34.8235110000
F	37.0018320000	31.5944780000	34.4365990000
N	31.2205380000	31.3983230000	31.9753870000
O	32.1556110000	31.4194870000	34.0545670000
C	33.3256090000	32.5433750000	32.3018430000
C	33.5723110000	33.7622860000	32.9367490000
C	34.6531440000	34.5636470000	32.5629060000
C	35.5086740000	34.1246880000	31.5415680000
C	35.2799130000	32.9175780000	30.9042670000
C	34.1840750000	32.0754010000	31.2537890000
C	32.1892810000	31.7304310000	32.8372830000
C	29.9619110000	30.6570630000	32.2637580000
C	30.2871920000	29.2484460000	32.7803260000
C	29.1278810000	31.4439120000	33.2862540000
C	29.2169920000	30.5635810000	30.9261820000
C	34.0073280000	30.8164290000	30.6314130000
C	34.9355700000	30.2530430000	29.6053400000
H	32.9002380000	34.0894430000	33.7257470000
H	34.8208520000	35.5173920000	33.0524460000
H	36.3575740000	34.7343050000	31.2478130000
H	35.9616550000	32.5900670000	30.1278010000
H	31.3330930000	31.7512280000	31.0335000000
H	33.1930370000	30.1839770000	30.9657720000
H	35.9308430000	30.0634790000	30.0352460000
H	34.5566220000	29.3053670000	29.2147240000
H	35.0828850000	30.9318990000	28.7547860000
H	29.3566710000	28.6888470000	32.9209620000
H	30.9159800000	28.7023850000	32.0716180000
H	30.8124050000	29.2920440000	33.7359560000
H	29.6693420000	31.5516210000	34.2286510000
H	28.8880120000	32.4414630000	32.9040010000
H	28.1879470000	30.9171400000	33.4802710000
H	29.8053590000	30.0127120000	30.1832330000
H	28.2699510000	30.0350790000	31.0640140000
H	28.9893930000	31.5596410000	30.5288000000
N	35.2356920000	29.5375280000	33.2229470000
N	34.3460770000	28.7367240000	33.0251240000
N	33.5019030000	27.9885760000	32.7911770000
O	36.4636160000	30.9706750000	36.9759860000
H	35.6539710000	31.4476320000	37.2327780000
H	37.0573750000	31.6572330000	36.6149180000

$^5Im_2Wat_2N_3$_FeF_remove-F

Charge: 1
Multiplicity: 5
B3LYP-D3(BJ)/6-31G(d)-SDD SCF energy (au): -893.385801270
B3LYP-D3(BJ)/6-31G(d)-SDD enthalpy (au): -893.154772270
B3LYP-D3(BJ)/6-31G(d)-SDD free energy (au): -893.229899270
B3LYP-D3(BJ)/def2-TZVP/SMD SCF energy (au): -2033.63568239
B3LYP-D3(BJ)/def2-TZVP/SMD enthalpy (au): -2033.40465339

B3LYP-D3(BJ)/def2-TZVP/SMD free energy (au): -2033.47978039
B3LYP-D3(BJ)/def2-TZVP/SMD free energy (quasi-harmonic) (au): -2033.472277120

Cartesian coordinates

ATOM	X	Y	Z
Fe	0.1271510000	-1.2015290000	0.2586320000
C	-1.3820120000	-2.2231640000	-3.6200950000
N	-2.4984590000	-1.8281660000	-2.9069170000
H	-3.4470200000	-1.8008350000	-3.2573070000
C	-2.1155460000	-1.4843960000	-1.6600660000
H	-2.7786460000	-1.1319070000	-0.8833900000
N	-0.7981470000	-1.6367850000	-1.5365300000
C	-0.3297930000	-2.0993810000	-2.7562070000
H	0.7163700000	-2.3084000000	-2.9187580000
H	-1.4392410000	-2.5466100000	-4.6472820000
C	2.4894410000	-4.4346460000	1.7166770000
N	3.4515630000	-3.4934660000	1.3995610000
H	4.4475690000	-3.5928320000	1.5445460000
C	2.8401500000	-2.4109360000	0.8764960000
H	3.3484570000	-1.5180460000	0.5466930000
N	1.5245360000	-2.6094680000	0.8387310000
C	1.2949810000	-3.8731540000	1.3630720000
H	0.2976120000	-4.2755230000	1.4528420000
H	2.7384670000	-5.3879760000	2.1549450000
O	2.0076080000	1.0143520000	2.6111760000
H	1.1654790000	1.0660950000	3.1132710000
H	2.5257750000	1.7890870000	2.8794500000
O	1.5376780000	0.3707140000	0.1117980000
H	1.7628220000	0.7111320000	1.0364000000
H	1.2411010000	1.1374550000	-0.4045760000
N	-1.2222630000	-0.4487890000	1.4286090000
N	-0.9946790000	0.1878100000	2.4322080000
N	-0.7902300000	0.8124560000	3.3830540000

$^5Im_2Wat_2N_3$_FeF_remove-N_3
Charge: 1
Multiplicity: 5
B3LYP-D3(BJ)/6-31G(d)-SDD SCF energy (au): -829.055172156
B3LYP-D3(BJ)/6-31G(d)-SDD enthalpy (au): -828.836857156
B3LYP-D3(BJ)/6-31G(d)-SDD free energy (au): -828.905716156
B3LYP-D3(BJ)/def2-TZVP/SMD SCF energy (au): -1969.28221291
B3LYP-D3(BJ)/def2-TZVP/SMD enthalpy (au): -1969.06389791
B3LYP-D3(BJ)/def2-TZVP/SMD free energy (au): -1969.13275691
B3LYP-D3(BJ)/def2-TZVP/SMD free energy (quasi-harmonic) (au): -1969.126740754

Cartesian coordinates

ATOM	X	Y	Z
Fe	0.6350380000	-0.9463530000	0.4958530000
C	-1.5548860000	-2.1070060000	-3.0232960000
N	-2.3472680000	-1.1324680000	-2.4486240000
H	-3.2341410000	-0.8020770000	-2.8055300000
C	-1.7421950000	-0.6916470000	-1.3245100000
H	-2.1358120000	0.0731180000	-0.6712620000

N	-0.5919080000	-1.3367530000	-1.1498880000
C	-0.4648750000	-2.2249470000	-2.2053940000
H	0.3891870000	-2.8786750000	-2.2953600000
H	-1.8322020000	-2.6120100000	-3.9349520000
C	2.3624700000	-4.6685870000	1.6620500000
N	3.4664670000	-3.9149430000	1.3085500000
H	4.4307540000	-4.2161030000	1.3613910000
C	3.0476310000	-2.7035850000	0.8896120000
H	3.6917520000	-1.9047240000	0.5550820000
N	1.7188680000	-2.6400550000	0.9564790000
C	1.2803990000	-3.8646120000	1.4391400000
H	0.2323930000	-4.0723120000	1.5919970000
H	2.4478930000	-5.6780830000	2.0316870000
F	-0.2457570000	0.3855130000	1.4029430000
O	1.6918800000	2.1715570000	1.3661230000
H	0.8312480000	1.7311240000	1.5633790000
H	2.0951670000	2.3905140000	2.2200910000
O	2.3526800000	0.1688460000	-0.1216050000
H	2.2916290000	1.0039670000	0.4579660000
H	2.3160460000	0.4863410000	-1.0388620000

$^{6}Im_2Wat_2N_3$_FeF

Charge: 1
Multiplicity: 6
B3LYP-D3(BJ)/6-31G(d)-SDD SCF energy (au): -993.242139814
B3LYP-D3(BJ)/6-31G(d)-SDD enthalpy (au): -993.007251814
B3LYP-D3(BJ)/6-31G(d)-SDD free energy (au): -993.083283814
B3LYP-D3(BJ)/def2-TZVP/SMD SCF energy (au): -2133.53069231
B3LYP-D3(BJ)/def2-TZVP/SMD enthalpy (au): -2133.29580431
B3LYP-D3(BJ)/def2-TZVP/SMD free energy (au): -2133.37183631
B3LYP-D3(BJ)/def2-TZVP/SMD free energy (quasi-harmonic) (au): -2133.366455496

Cartesian coordinates

ATOM	X	Y	Z
Fe	0.3704350000	-0.4675560000	0.7344300000
C	-1.3784400000	-2.5370990000	-2.6119120000
N	-1.9516250000	-1.2804510000	-2.6794160000
H	-2.6610550000	-0.9864250000	-3.3377040000
C	-1.4186450000	-0.5099220000	-1.7082070000
H	-1.6855640000	0.5164180000	-1.5078480000
N	-0.5233810000	-1.2145080000	-1.0220090000
C	-0.4910550000	-2.4830760000	-1.5730950000
H	0.1527880000	-3.2523630000	-1.1778380000
H	-1.6491070000	-3.3303930000	-3.2905100000
C	2.3441880000	-4.1860760000	1.6156170000
N	3.2309110000	-3.6545610000	0.6959740000
H	4.0751070000	-4.0995590000	0.3605130000
C	2.7888230000	-2.4341750000	0.3256080000
H	3.2775740000	-1.7914470000	-0.3891440000
N	1.6565550000	-2.1539620000	0.9652650000
C	1.3668720000	-3.2436870000	1.7741940000
H	0.4824190000	-3.2520660000	2.3912610000
H	2.4890330000	-5.1582010000	2.0597030000

F	-0.2875800000	1.2092980000	0.3985200000
O	1.6517000000	0.6473280000	2.2164980000
H	1.0790760000	1.4154340000	2.3992830000
H	1.7348000000	0.1641760000	3.0567380000
O	2.1567220000	0.3053270000	-0.4978200000
H	2.5914790000	0.8514210000	0.1825640000
H	1.7304280000	0.9421660000	-1.0976070000
N	-0.8413930000	-1.1317530000	2.0594440000
N	-1.9735720000	-0.7274640000	2.2806730000
N	-3.0428760000	-0.3878450000	2.5148350000

$^5Im_2Wat_2N_3$_TS-1
Charge: 1
Multiplicity: 5
B3LYP-D3(BJ)/6-31G(d)-SDD SCF energy (au): -1629.44760838
B3LYP-D3(BJ)/6-31G(d)-SDD enthalpy (au): -1628.91108338
B3LYP-D3(BJ)/6-31G(d)-SDD free energy (au): -1629.02372938
B3LYP-D3(BJ)/def2-TZVP/SMD SCF energy (au): -2769.99195039
B3LYP-D3(BJ)/def2-TZVP/SMD enthalpy (au): -2769.45542539
B3LYP-D3(BJ)/def2-TZVP/SMD free energy (au): -2769.56807139
B3LYP-D3(BJ)/def2-TZVP/SMD free energy (quasi-harmonic) (au): -2769.55753301

Cartesian coordinates

ATOM	X	Y	Z
Fe	0.3498840000	-0.4676800000	0.8439300000
C	-1.1143950000	-2.5015960000	-2.7524730000
N	-2.0023940000	-1.4506870000	-2.6205460000
H	-2.8485910000	-1.3183440000	-3.1573710000
C	-1.5595480000	-0.6425840000	-1.6287070000
H	-2.0702730000	0.2468310000	-1.2957990000
N	-0.4310420000	-1.1175240000	-1.1189850000
C	-0.1454630000	-2.2809140000	-1.8114850000
H	0.7225260000	-2.8750950000	-1.5732050000
H	-1.2543800000	-3.2844840000	-3.4809910000
C	2.2383680000	-4.2611350000	1.4910660000
N	3.2449410000	-3.6047170000	0.8071650000
H	4.1491450000	-3.9888470000	0.5694660000
C	2.8181250000	-2.3500580000	0.5215540000
H	3.3960990000	-1.6089480000	-0.0095890000
N	1.5885980000	-2.1774760000	0.9875480000
C	1.2124620000	-3.3607480000	1.5966150000
H	0.2413300000	-3.4546730000	2.0579470000
H	2.3446870000	-5.2788440000	1.8319590000
C	1.5268730000	6.4272680000	3.3463200000
C	1.3987260000	5.4103800000	2.4129760000
C	0.6007510000	4.2837720000	2.6951950000
C	-0.0448190000	4.1353970000	3.9442600000
C	0.1210320000	5.1749260000	4.8709420000
C	0.8747120000	6.3083530000	4.5798570000
H	2.1323700000	7.3001200000	3.1248870000
H	1.9029280000	5.4712370000	1.4536370000
H	-0.3457020000	5.0791090000	5.8473860000
H	0.9705680000	7.0958140000	5.3213460000

C	-0.8263310000	2.9071180000	4.3648280000
C	-2.3403210000	3.1503830000	4.4486610000
H	-0.6318640000	2.0679090000	3.6952070000
H	-0.4619310000	2.6084080000	5.3559030000
H	-2.8501780000	2.2444720000	4.7862690000
H	-2.7460000000	3.4100670000	3.4670260000
H	-2.5764610000	3.9651590000	5.1417320000
C	0.5089240000	3.2715530000	1.6116990000
O	1.5217940000	2.7903040000	1.0780030000
N	-0.7916590000	2.9252380000	1.3475410000
C	-1.6701900000	3.6052290000	0.3857010000
C	-1.8998520000	5.0453260000	0.9203420000
C	-1.0228740000	3.6725810000	-1.0077820000
H	-2.6467740000	5.5162500000	0.2741680000
H	-0.9848320000	5.6397520000	0.8896740000
H	-2.2782860000	5.0343470000	1.9440230000
H	-0.8583130000	2.6672040000	-1.4018890000
H	-0.0628980000	4.1976220000	-0.9754520000
H	-1.6845320000	4.2132500000	-1.6912050000
F	-0.6235490000	1.2295260000	0.6014670000
C	-3.0093370000	2.8592150000	0.3609960000
H	-3.4365710000	2.7914860000	1.3643000000
H	-2.8792130000	1.8415730000	-0.0087830000
H	-3.7111880000	3.3893620000	-0.2896970000
O	1.5048800000	0.4505990000	2.5274850000
H	2.1830220000	-0.1369790000	2.8955500000
H	1.9401610000	1.2867470000	2.2736650000
O	1.8678130000	0.5394240000	-0.3691160000
H	1.7415890000	1.4668540000	-0.0348960000
H	1.4490180000	0.5085020000	-1.2457920000
N	-1.0586240000	-1.1925670000	1.9932920000
N	-1.9580000000	-0.4932430000	2.4184590000
N	-2.8292160000	0.1312770000	2.8402840000

$^5Im_2Wat_2N_3$_TS-3-F

Charge: 1
Multiplicity: 5
B3LYP-D3(BJ)/6-31G(d)-SDD SCF energy (au): -1629.52101210
B3LYP-D3(BJ)/6-31G(d)-SDD enthalpy (au): -1628.98378110
B3LYP-D3(BJ)/6-31G(d)-SDD free energy (au): -1629.09740210
B3LYP-D3(BJ)/def2-TZVP/SMD SCF energy (au): -2770.06182226
B3LYP-D3(BJ)/def2-TZVP/SMD enthalpy (au): -2769.52459126
B3LYP-D3(BJ)/def2-TZVP/SMD free energy (au): -2769.63821226
B3LYP-D3(BJ)/def2-TZVP/SMD free energy (quasi-harmonic) (au): -2769.62710116

Cartesian coordinates

ATOM	X	Y	Z
H	32.9101970000	26.2664420000	37.5700320000
C	33.2027260000	27.2466180000	37.2285820000
C	34.3730980000	27.7272590000	36.7093170000
N	32.3405470000	28.3250610000	37.2568730000
C	32.9872390000	29.4026490000	36.7583980000
N	34.2300160000	29.0753270000	36.4233400000

H	31.3739300000	28.3051220000	37.5508500000
H	35.2972960000	27.2079060000	36.5143630000
H	32.5536080000	30.3830880000	36.6445490000
H	39.4991130000	26.6891040000	35.8183770000
C	38.6655590000	27.2580570000	35.4378150000
C	37.9301690000	28.2826850000	35.9706120000
N	38.1214410000	27.0281990000	34.1889230000
C	37.0970060000	27.8946810000	34.0020540000
N	36.9561940000	28.6679780000	35.0690250000
H	38.4305980000	26.3303160000	33.5263080000
H	38.0343380000	28.7588580000	36.9332690000
H	36.4785870000	27.9493200000	33.1188300000
Fe	35.4641220000	30.2746120000	35.0709410000
O	34.0433790000	31.8422560000	35.4936090000
H	34.5545320000	32.6351420000	35.7233450000
H	33.4940540000	32.0800700000	34.6806880000
F	36.6856740000	31.4335340000	34.2334240000
N	33.3489030000	32.6558230000	31.1207820000
O	32.7082690000	32.3527470000	33.2932210000
C	34.5735760000	33.7884240000	32.8542340000
C	34.1827320000	34.9647410000	33.4901510000
C	35.1272860000	35.9361550000	33.8318610000
C	36.4806860000	35.7230210000	33.5383060000
C	36.8880790000	34.5396500000	32.9432260000
C	35.9548850000	33.5252010000	32.6030950000
C	33.4734080000	32.8491490000	32.4439370000
C	32.2632770000	31.9134910000	30.4207960000
C	32.2175190000	30.4609810000	30.9098800000
C	30.9228260000	32.6202060000	30.6751140000
C	32.6224470000	31.9606140000	28.9305820000
C	36.3706690000	32.2399760000	32.1627460000
C	37.7408400000	31.8705890000	31.7238510000
H	33.1286950000	35.1303060000	33.6918540000
H	34.8088790000	36.8572860000	34.3093980000
H	37.2159150000	36.4807780000	33.7907030000
H	37.9431690000	34.3716620000	32.7613400000
H	33.9676100000	33.2019450000	30.5354540000
H	35.5983260000	31.4956860000	31.9992610000
H	38.5234570000	32.4169050000	32.2547980000
H	37.9030280000	30.8004730000	31.8746500000
H	37.8608340000	32.0753760000	30.6481910000
H	31.4969670000	29.8982320000	30.3078330000
H	33.1990240000	29.9871500000	30.8091310000
H	31.9103260000	30.4055110000	31.9537280000
H	30.6907260000	32.6300720000	31.7427100000
H	30.9499130000	33.6520930000	30.3084640000
H	30.1196130000	32.0917810000	30.1515630000
H	33.5808340000	31.4639320000	28.7396660000
H	31.8540250000	31.4460510000	28.3475610000
H	32.6809890000	32.9935940000	28.5657560000
N	34.5727240000	29.5656480000	33.4279350000
N	33.5346450000	28.9383910000	33.5169780000
N	32.5451640000	28.3496280000	33.5774740000

O 36.3829260000 31.1829720000 36.9174660000
H 36.3843330000 30.6497800000 37.7279090000
H 37.3026280000 31.4119240000 36.7040980000

$^{5}Im_2Wat_2N_3$_TS-3-N_3

Charge: 1
Multiplicity: 5
B3LYP-D3(BJ)/6-31G(d)-SDD SCF energy (au): -1629.53760394
B3LYP-D3(BJ)/6-31G(d)-SDD enthalpy (au): -1628.99956994
B3LYP-D3(BJ)/6-31G(d)-SDD free energy (au): -1629.11023894
B3LYP-D3(BJ)/def2-TZVP/SMD SCF energy (au): -2770.07238075
B3LYP-D3(BJ)/def2-TZVP/SMD enthalpy (au): -2769.53434675
B3LYP-D3(BJ)/def2-TZVP/SMD free energy (au): -2769.64501575
B3LYP-D3(BJ)/def2-TZVP/SMD free energy (quasi-harmonic) (au): -2769.63561081

Cartesian coordinates

ATOM	X	Y	Z
H	33.0382110000	27.2832910000	38.5628570000
C	33.2678790000	27.9747910000	37.7679290000
C	34.4516150000	28.3717490000	37.2104670000
N	32.2866150000	28.6737400000	37.0904630000
C	32.8789960000	29.4556000000	36.1603410000
N	34.1972910000	29.2962420000	36.2115440000
H	31.2909150000	28.6032050000	37.2477630000
H	35.4572100000	28.0647860000	37.4474780000
H	32.3450750000	30.1036350000	35.4800590000
H	39.4683580000	26.7287410000	36.5189160000
C	38.6727130000	27.2039300000	35.9673070000
C	37.9091000000	28.3092890000	36.2261400000
N	38.2325550000	26.7248460000	34.7474730000
C	37.2374140000	27.5285070000	34.3074000000
N	37.0157050000	28.4963410000	35.1852560000
H	38.5957900000	25.9168180000	34.2609710000
H	37.9334700000	28.9847360000	37.0665580000
H	36.7172460000	27.4042010000	33.3705570000
Fe	35.6657880000	30.1354670000	34.8491350000
O	34.5290380000	31.9900700000	35.1023300000
H	35.0043470000	32.5524550000	34.4637040000
H	33.5944110000	31.9323740000	34.7479040000
F	36.9261390000	31.2453560000	34.0841530000
N	31.2539280000	31.5547360000	31.8700270000
O	32.1332800000	31.6378320000	33.9758550000
C	33.4511360000	32.5164460000	32.1956710000
C	33.7097690000	33.8257260000	32.5959930000
C	34.8997900000	34.4621650000	32.2293010000
C	35.8512920000	33.7650280000	31.4732650000
C	35.6121460000	32.4580940000	31.0803670000
C	34.4048760000	31.7884610000	31.4177890000
C	32.2193810000	31.8583560000	32.7467770000
C	29.9320740000	30.9310250000	32.1624410000
C	30.1411720000	29.5331680000	32.7611220000
C	29.1400920000	31.8340250000	33.1199430000
C	29.2119210000	30.8228120000	30.8128480000

```
C     34.1868940000   30.4356060000   31.0525600000
C     35.1539530000   29.6105900000   30.2735900000
H     32.9771740000   34.3498290000   33.2033060000
H     35.0828290000   35.4875220000   32.5337640000
H     36.7841830000   34.2468130000   31.1986480000
H     36.3663390000   31.9270950000   30.5116800000
H     31.4206170000   31.8414980000   30.9137890000
H     33.2110500000   30.0070590000   31.2502280000
H     36.1607140000   29.6625570000   30.7028010000
H     34.8423300000   28.5634360000   30.2558780000
H     35.2244840000   29.9531450000   29.2299250000
H     29.1694560000   29.0571680000   32.9273090000
H     30.7221780000   28.8968070000   32.0867880000
H     30.6675570000   29.5859190000   33.7155330000
H     29.6650130000   31.9487310000   34.0706840000
H     28.9890720000   32.8255420000   32.6806470000
H     28.1570310000   31.3931230000   33.3137240000
H     29.7721980000   30.1907710000   30.1139730000
H     28.2252800000   30.3735370000   30.9535690000
H     29.0680000000   31.8104850000   30.3593200000
N     34.8238530000   29.2685720000   33.2792450000
N     33.9088230000   28.4690200000   33.3149330000
N     33.0385650000   27.7134280000   33.3023810000
O     36.5948390000   31.0205610000   36.7327360000
H     35.8893250000   31.6535420000   36.9572970000
H     37.2214110000   31.5293510000   36.1813550000
```

^{5}Im$_2$Wat$_2$OH_11

```
Charge: 1
Multiplicity: 5
B3LYP-D3(BJ)/6-31G(d)-SDD SCF energy (au):              -1541.09343838
B3LYP-D3(BJ)/6-31G(d)-SDD enthalpy (au):                -1540.55497738
B3LYP-D3(BJ)/6-31G(d)-SDD free energy (au):             -1540.66585538
B3LYP-D3(BJ)/def2-TZVP/SMD SCF energy (au):             -2681.59541738
B3LYP-D3(BJ)/def2-TZVP/SMD enthalpy (au):               -2681.05695638
B3LYP-D3(BJ)/def2-TZVP/SMD free energy (au):            -2681.16783438
B3LYP-D3(BJ)/def2-TZVP/SMD free energy (quasi-harmonic) (au): -2681.15607500

Cartesian coordinates
ATOM    X               Y               Z
Fe    -0.0276010000   -0.7357270000    1.5877250000
C      1.0020500000    1.0737990000   -2.1606470000
N      2.1666950000    1.2810940000   -1.4404230000
H      2.9937290000    1.7585570000   -1.7707840000
C      1.9919480000    0.7872680000   -0.1940480000
H      2.7273910000    0.8276050000    0.5945770000
N      0.7771480000    0.2623970000   -0.0833250000
C      0.1459640000    0.4367020000   -1.3031450000
H     -0.8637840000    0.0876210000   -1.4519390000
H      0.9000660000    1.3834300000   -3.1887440000
C      1.1201100000   -4.7745200000    2.3808580000
N      2.0079680000   -4.5369200000    1.3490740000
H      2.6763570000   -5.1974760000    0.9775120000
```

C	1.8277040000	-3.2629670000	0.9198320000
H	2.3918190000	-2.8118140000	0.1174400000
N	0.8721200000	-2.6759010000	1.6232780000
C	0.4191600000	-3.6098700000	2.5421910000
H	-0.3684630000	-3.3543670000	3.2356360000
H	1.0725810000	-5.7240920000	2.8900580000
O	-1.8610820000	-1.4907700000	0.3306320000
H	-2.1876610000	-1.3748580000	1.2617800000
H	-1.8102660000	-2.4522470000	0.2062250000
C	1.8990850000	4.9478810000	-0.4302250000
C	0.9327690000	4.0418390000	-0.0042340000
C	0.5998090000	3.9335740000	1.3560610000
C	1.2533570000	4.7336940000	2.3219930000
C	2.2079090000	5.6473700000	1.8625650000
C	2.5331530000	5.7598530000	0.5099280000
H	2.1475510000	5.0267850000	-1.4845200000
H	0.4304950000	3.3968460000	-0.7162580000
H	2.7083270000	6.2845820000	2.5860930000
H	3.2831330000	6.4791930000	0.1948230000
C	0.9241910000	4.6861090000	3.7991740000
C	-0.4560990000	5.2777270000	4.1279470000
H	0.9684700000	3.6560410000	4.1610320000
H	1.6923720000	5.2493750000	4.3391870000
H	-0.6222700000	5.2846490000	5.2096390000
H	-1.2592930000	4.6870270000	3.6758750000
H	-0.5419830000	6.3066260000	3.7625830000
C	-0.3740290000	2.8816560000	1.7438140000
O	-0.1902030000	2.1007870000	2.6829570000
N	-1.4273510000	2.7010620000	0.8532340000
C	-2.5714200000	3.6384130000	0.5635080000
C	-2.0647600000	5.0734150000	0.3794540000
C	-3.2170360000	3.1416600000	-0.7363700000
H	-2.9373410000	5.6951790000	0.1585390000
H	-1.3646370000	5.1636160000	-0.4527340000
H	-1.5903130000	5.4693440000	1.2797120000
H	-3.5868390000	2.1196690000	-0.6286320000
H	-2.4933260000	3.1684920000	-1.5573420000
H	-4.0602720000	3.7880410000	-0.9966180000
F	-2.0058840000	1.4333990000	1.1091180000
C	-3.5633770000	3.5842750000	1.7376680000
H	-3.0939040000	3.9282540000	2.6646810000
H	-3.9433610000	2.5731450000	1.8922920000
H	-4.4109800000	4.2417360000	1.5228710000
O	-1.3834510000	-0.9707970000	2.8751450000
H	-1.6813300000	-0.1603030000	3.3143060000
O	1.6359650000	0.1795900000	2.7738800000
H	1.6718350000	-0.3131970000	3.6097490000
H	1.1688090000	1.0270740000	2.9697790000

$^{5}Im_2Wat_2OH\_13$

Charge: 1

Multiplicity: 5

B3LYP-D3(BJ)/6-31G(d)-SDD SCF energy (au): -1541.15440206

B3LYP-D3(BJ)/6-31G(d)-SDD enthalpy (au): -1540.61714106
B3LYP-D3(BJ)/6-31G(d)-SDD free energy (au): -1540.72822706
B3LYP-D3(BJ)/def2-TZVP/SMD SCF energy (au): -2681.66201061
B3LYP-D3(BJ)/def2-TZVP/SMD enthalpy (au): -2681.12474961
B3LYP-D3(BJ)/def2-TZVP/SMD free energy (au): -2681.23583561
B3LYP-D3(BJ)/def2-TZVP/SMD free energy (quasi-harmonic) (au): -2681.22380355

Cartesian coordinates

ATOM	X	Y	Z
Fe	0.1182290000	0.1861450000	-0.5069700000
C	1.7884360000	-0.2564820000	3.4773530000
N	2.8209040000	0.2866870000	2.7331900000
H	3.7357340000	0.5378870000	3.0820330000
C	2.3974190000	0.4417550000	1.4589430000
H	2.9778090000	0.8506800000	0.6457750000
N	1.1439160000	0.0203380000	1.3518340000
C	0.7478860000	-0.4165640000	2.6034770000
H	-0.2474190000	-0.7969680000	2.7707430000
O	1.5472480000	0.9700790000	-1.3352430000
H	1.2814850000	1.7079730000	-1.9206050000
H	1.8865710000	-0.4649340000	4.5309670000
C	1.2641540000	-3.6393790000	-2.1180680000
N	1.1892240000	-3.9542440000	-0.7725900000
H	1.4034810000	-4.8517890000	-0.3604950000
C	0.7825080000	-2.8541510000	-0.0993890000
H	0.6472150000	-2.8075700000	0.9697840000
N	0.5906090000	-1.8536290000	-0.9475220000
C	0.8898340000	-2.3275520000	-2.2116850000
H	0.8069980000	-1.6906410000	-3.0768520000
H	1.5698080000	-4.3590510000	-2.8606900000
O	-0.9908150000	0.1190630000	-2.3846290000
H	-0.6695800000	0.9975120000	-2.7625950000
H	-1.9435010000	0.2735820000	-2.2681450000
F	-1.0665860000	1.5236600000	0.0355630000
C	-1.0378680000	3.3634790000	-2.5483260000
O	-0.1403850000	2.5587900000	-2.9059150000
N	-0.7772240000	4.5997120000	-2.1037800000
H	-2.1202770000	3.8210740000	-0.1181250000
C	0.5458910000	5.2166870000	-1.8229640000
C	0.2499970000	6.6780350000	-1.4583820000
C	1.2033030000	4.5050580000	-0.6291000000
C	1.4378600000	5.1610190000	-3.0718430000
H	-0.2344280000	7.2020700000	-2.2898820000
H	-0.3975090000	6.7434310000	-0.5754530000
H	1.1823340000	7.2005510000	-1.2281300000
H	1.4052870000	3.4543890000	-0.8426330000
H	2.1528310000	4.9949590000	-0.3881610000
H	0.5562760000	4.5534240000	0.2531100000
H	2.3839060000	5.6721740000	-2.8661830000
H	1.6510520000	4.1315640000	-3.3626770000
H	0.9516250000	5.6656840000	-3.9130300000
C	-2.4794710000	2.9899110000	-2.6891920000
C	-2.8311130000	2.3646910000	-3.8878010000

C	-3.4781110000	3.2629640000	-1.6899860000
C	-4.1563110000	2.0276370000	-4.1694650000
H	-2.0527000000	2.1699030000	-4.6192840000
C	-4.8233230000	2.9254780000	-2.0248210000
C	-5.1521450000	2.3248360000	-3.2286150000
H	-4.4105880000	1.5597910000	-5.1148210000
H	-5.6051480000	3.1214350000	-1.2995390000
H	-6.1886140000	2.0790560000	-3.4401320000
C	-3.1623850000	3.7735660000	-0.4070130000
C	-4.1806450000	4.1098620000	0.6352630000
H	-4.6242890000	3.2050080000	1.0805110000
H	-3.7266650000	4.6778890000	1.4517780000
H	-5.0126300000	4.7014980000	0.2337400000
H	-1.5879770000	5.1654280000	-1.8884840000
O	-1.6745080000	-0.8360300000	0.6974670000
H	-2.3107310000	-1.3295560000	0.1562810000
H	-1.9799980000	0.0978290000	0.7173460000

$^{5}Im_2Wat_2OH\_FeF\_remove\text{-}F$

Charge: 1
Multiplicity: 5
B3LYP-D3(BJ)/6-31G(d)-SDD SCF energy (au): -805.005489630
B3LYP-D3(BJ)/6-31G(d)-SDD enthalpy (au): -804.776436630
B3LYP-D3(BJ)/6-31G(d)-SDD free energy (au): -804.846817630
B3LYP-D3(BJ)/def2-TZVP/SMD SCF energy (au): -1945.22035199
B3LYP-D3(BJ)/def2-TZVP/SMD enthalpy (au): -1944.99129899
B3LYP-D3(BJ)/def2-TZVP/SMD free energy (au): -1945.06167999
B3LYP-D3(BJ)/def2-TZVP/SMD free energy (quasi-harmonic) (au): -1945.057093360

Cartesian coordinates

ATOM	X	Y	Z
Fe	0.4671800000	-0.7918340000	0.8674320000
C	-1.7316720000	-0.4701070000	-2.7944250000
N	-2.4699210000	-1.4030480000	-2.0897160000
H	-3.3011800000	-1.8742700000	-2.4210650000
C	-1.8970580000	-1.5792020000	-0.8798070000
H	-2.2540620000	-2.2632690000	-0.1251680000
N	-0.8237360000	-0.8014350000	-0.7705960000
C	-0.7084230000	-0.1056610000	-1.9640230000
H	0.1002740000	0.5883270000	-2.1286490000
H	-1.9985880000	-0.1598430000	-3.7922020000
C	2.5698830000	-4.4744370000	-0.0781160000
N	2.8841840000	-4.2418630000	1.2466490000
H	3.4438820000	-4.8417230000	1.8375950000
C	2.3086500000	-3.0777250000	1.6267230000
H	2.3812660000	-2.6418690000	2.6123080000
N	1.6411970000	-2.5494100000	0.6075550000
C	1.7957600000	-3.4129640000	-0.4629540000
H	1.3405580000	-3.2104220000	-1.4206210000
H	2.9168980000	-5.3473970000	-0.6078920000
O	-1.1551080000	-1.8106760000	2.2266160000
H	-0.5301980000	-1.3828450000	2.8650180000
H	-1.9910510000	-1.3328750000	2.3552580000

O	0.9654550000	-0.2901780000	2.6036920000
H	1.0060540000	0.6083850000	2.9575210000
O	2.0252390000	0.3235330000	-0.3227430000
H	2.2849100000	1.1572400000	0.1041030000
H	2.8070220000	-0.2515760000	-0.2598520000

^{5}Im$_2$Wat$_2$OH_FeF_remove-OH
Charge: 1
Multiplicity: 5
B3LYP-D3(BJ)/6-31G(d)-SDD SCF energy (au): -829.053220256
B3LYP-D3(BJ)/6-31G(d)-SDD enthalpy (au): -828.835091256
B3LYP-D3(BJ)/6-31G(d)-SDD free energy (au): -828.903144256
B3LYP-D3(BJ)/def2-TZVP/SMD SCF energy (au): -1969.27415149
B3LYP-D3(BJ)/def2-TZVP/SMD enthalpy (au): -1969.05602249
B3LYP-D3(BJ)/def2-TZVP/SMD free energy (au): -1969.12407549
B3LYP-D3(BJ)/def2-TZVP/SMD free energy (quasi-harmonic) (au): -1969.119686234

Cartesian coordinates

ATOM	X	Y	Z
Fe	0.2660210000	-0.9982670000	0.7269780000
C	-1.3815070000	-0.6476530000	-3.2118810000
N	-2.5041050000	-0.8048300000	-2.4209680000
H	-3.4622560000	-0.8028860000	-2.7448810000
C	-2.1106720000	-0.9569030000	-1.1385190000
H	-2.7755780000	-1.1058050000	-0.3013020000
N	-0.7836520000	-0.9027570000	-1.0644460000
C	-0.3177600000	-0.7104910000	-2.3555090000
H	0.7373410000	-0.6415750000	-2.5721760000
H	-1.4434620000	-0.5112580000	-4.2798860000
C	2.2890340000	-4.7739340000	0.6988370000
N	3.3299130000	-3.8676900000	0.7748350000
H	4.3129870000	-4.0979410000	0.8342630000
C	2.8167910000	-2.6190410000	0.7625420000
H	3.3988140000	-1.7109140000	0.8043240000
N	1.4901880000	-2.6773740000	0.6842180000
C	1.1498620000	-4.0202620000	0.6429350000
H	0.1222880000	-4.3417930000	0.5677390000
H	2.4524460000	-5.8399080000	0.6903260000
O	-1.5865710000	-1.6905140000	1.8654270000
H	-1.5087690000	-2.4604670000	2.4523640000
H	-1.3832410000	-0.9112990000	2.4294330000
F	0.1281490000	0.2204940000	2.1368880000
O	2.0401090000	0.4009220000	0.4093930000
H	1.9727570000	1.0857050000	-0.2761110000
H	1.7038300000	0.8216780000	1.2318280000

^{6}Im$_2$Wat$_2$OH_FeF
Charge: 1
Multiplicity: 6
B3LYP-D3(BJ)/6-31G(d)-SDD SCF energy (au): -904.865739905
B3LYP-D3(BJ)/6-31G(d)-SDD enthalpy (au): -904.632340905
B3LYP-D3(BJ)/6-31G(d)-SDD free energy (au): -904.702620905
B3LYP-D3(BJ)/def2-TZVP/SMD SCF energy (au): -2045.12474063

B3LYP-D3(BJ)/def2-TZVP/SMD enthalpy (au): -2044.89134163
B3LYP-D3(BJ)/def2-TZVP/SMD free energy (au): -2044.96162163
B3LYP-D3(BJ)/def2-TZVP/SMD free energy (quasi-harmonic) (au): -2044.958718725

Cartesian coordinates

ATOM	X	Y	Z
Fe	0.3081710000	-0.7274540000	1.1870350000
C	-1.1073190000	-1.1579100000	-2.9452490000
N	-2.2190910000	-0.6842230000	-2.2726260000
H	-3.1193570000	-0.4782630000	-2.6847390000
C	-1.8986700000	-0.5309040000	-0.9691410000
H	-2.5639550000	-0.1501360000	-0.2101020000
N	-0.6359240000	-0.8907490000	-0.7689870000
C	-0.1288350000	-1.2801890000	-1.9973180000
H	0.8921460000	-1.6107470000	-2.1040270000
H	-1.1161720000	-1.3542180000	-4.0057400000
C	2.1299840000	-4.5070430000	-0.0193260000
N	3.1107850000	-3.9016680000	0.7459550000
H	4.0189320000	-4.2904120000	0.9625810000
C	2.6514560000	-2.7031200000	1.1648190000
H	3.1864150000	-2.0177640000	1.8039740000
N	1.4225410000	-2.5052060000	0.6988320000
C	1.0835860000	-3.6270400000	-0.0393740000
H	0.1220400000	-3.7148220000	-0.5193840000
H	2.2642240000	-5.4813030000	-0.4620000000
O	-1.0280210000	-2.1700660000	2.1681130000
H	-0.5344880000	-2.6997310000	2.8178530000
H	-1.6167720000	-1.5807040000	2.6746160000
F	-1.0301030000	0.4309660000	1.6497040000
O	1.5256550000	-0.4188460000	2.5606370000
H	1.1994660000	0.1959170000	3.2387460000
O	1.5834960000	0.6521930000	0.0504890000
H	1.0680520000	1.3727790000	-0.3514260000
H	2.1098340000	1.0429740000	0.7721180000

^{5}Im$_2$Wat$_2$OH_TS-1
Charge: 1
Multiplicity: 5
B3LYP-D3(BJ)/6-31G(d)-SDD SCF energy (au): -1541.07398927
B3LYP-D3(BJ)/6-31G(d)-SDD enthalpy (au): -1540.53838627
B3LYP-D3(BJ)/6-31G(d)-SDD free energy (au): -1540.64632327
B3LYP-D3(BJ)/def2-TZVP/SMD SCF energy (au): -2681.57376980
B3LYP-D3(BJ)/def2-TZVP/SMD enthalpy (au): -2681.03816680
B3LYP-D3(BJ)/def2-TZVP/SMD free energy (au): -2681.14610380
B3LYP-D3(BJ)/def2-TZVP/SMD free energy (quasi-harmonic) (au): -2681.13651553

Cartesian coordinates

ATOM	X	Y	Z
Fe	0.4933960000	-0.5962390000	1.0027650000
C	-1.3928300000	-0.9556550000	-2.8430270000
N	-2.2707080000	-1.6431120000	-2.0247920000
H	-3.1084350000	-2.1234870000	-2.3237110000
C	-1.8178380000	-1.5707930000	-0.7514840000

H	-2.2862050000	-2.0335940000	0.1036110000
N	-0.6945840000	-0.8667450000	-0.7161660000
C	-0.4136560000	-0.4778460000	-2.0141230000
H	0.4654720000	0.1046700000	-2.2400870000
H	-1.5443380000	-0.8718620000	-3.9075520000
C	2.2808400000	-4.3246870000	-0.3225990000
N	2.7228130000	-4.1973080000	0.9806620000
H	3.3152700000	-4.8507830000	1.4737580000
C	2.2224790000	-3.0479910000	1.4928130000
H	2.3917290000	-2.6743340000	2.4912650000
N	1.4801400000	-2.4346330000	0.5811170000
C	1.5077900000	-3.2196720000	-0.5569040000
H	0.9799580000	-2.9322740000	-1.4525970000
H	2.5514830000	-5.1639390000	-0.9435720000
O	-1.3463940000	-1.5073070000	2.2501760000
H	-0.5889520000	-1.4710890000	2.8812180000
H	-1.8075940000	-0.6670150000	2.4134560000
C	1.3917770000	6.8812220000	2.7649300000
C	1.3550160000	5.6573290000	2.1136660000
C	0.5808890000	4.5993470000	2.6288180000
C	-0.1280130000	4.7400090000	3.8430860000
C	-0.0571030000	5.9835280000	4.4850820000
C	0.6717310000	7.0444980000	3.9537760000
H	1.9791060000	7.6988500000	2.3599050000
H	1.9120170000	5.5000230000	1.1956220000
H	-0.5740430000	6.1113430000	5.4323330000
H	0.6959510000	7.9959340000	4.4764510000
C	-0.8769020000	3.6145580000	4.5290950000
C	-2.4041200000	3.7516750000	4.4463380000
H	-0.5789450000	2.6463170000	4.1195060000
H	-0.5782290000	3.6151980000	5.5846490000
H	-2.8916880000	2.9731220000	5.0422200000
H	-2.7446300000	3.6526110000	3.4117990000
H	-2.7383580000	4.7238930000	4.8238120000
C	0.5693070000	3.3575480000	1.8159530000
O	1.5952390000	2.8445720000	1.3747930000
N	-0.7349360000	2.8855950000	1.6244270000
C	-1.4892680000	3.2669120000	0.4125530000
C	-1.9002220000	4.7509680000	0.6124250000
C	-0.6616420000	3.1244580000	-0.8723900000
H	-2.5604460000	5.0229730000	-0.2167560000
H	-1.0355130000	5.4168310000	0.6015240000
H	-2.4399980000	4.8936690000	1.5507320000
H	-0.3491410000	2.0893980000	-1.0155440000
H	0.2272570000	3.7619870000	-0.8559400000
H	-1.2747270000	3.4215950000	-1.7289440000
F	-0.4522790000	1.1132090000	1.3985180000
C	-2.7541660000	2.3994850000	0.3578250000
H	-3.2972990000	2.4498320000	1.3060930000
H	-2.4973050000	1.3588940000	0.1519840000
H	-3.4104350000	2.7642420000	-0.4381340000
O	1.1802440000	-0.6934890000	2.7523150000
H	1.2301700000	0.1782260000	3.1743420000

O	1.9045890000	0.6175650000	-0.1183770000
H	1.8157390000	1.4848610000	0.3601560000
H	2.8068370000	0.3013900000	0.0499800000

$^5Im_2Wat_2OH$_TS-3-F
Charge: 1
Multiplicity: 5
B3LYP-D3(BJ)/6-31G(d)-SDD SCF energy (au): -1541.14373953
B3LYP-D3(BJ)/6-31G(d)-SDD enthalpy (au): -1540.60683953
B3LYP-D3(BJ)/6-31G(d)-SDD free energy (au): -1540.71279553
B3LYP-D3(BJ)/def2-TZVP/SMD SCF energy (au): -2681.64814221
B3LYP-D3(BJ)/def2-TZVP/SMD enthalpy (au): -2681.11124221
B3LYP-D3(BJ)/def2-TZVP/SMD free energy (au): -2681.21719821
B3LYP-D3(BJ)/def2-TZVP/SMD free energy (quasi-harmonic) (au): -2681.20954768

Cartesian coordinates

ATOM	X	Y	Z
Fe	-0.6000410000	0.9701060000	-0.9216350000
C	1.0928410000	1.1939180000	3.1117970000
N	1.4709980000	2.3828250000	2.5156760000
H	1.9990280000	3.1244660000	2.9538830000
C	1.0046210000	2.3893830000	1.2432180000
H	1.1387430000	3.1850190000	0.5260020000
N	0.3483340000	1.2651930000	0.9966100000
C	0.3934280000	0.5094280000	2.1540060000
H	-0.0824110000	-0.4573140000	2.2070120000
O	0.1714870000	2.5291320000	-1.6839430000
H	0.9668490000	2.3260530000	-2.1971290000
H	1.3461240000	0.9562050000	4.1329690000
C	2.3341240000	-1.4602660000	-3.0328570000
N	2.6401360000	-1.7703530000	-1.7204460000
H	3.3839230000	-2.3813810000	-1.4129460000
C	1.7660000000	-1.1185070000	-0.9159260000
H	1.7752420000	-1.1707830000	0.1620390000
N	0.9176810000	-0.4103760000	-1.6444530000
C	1.2613820000	-0.6124570000	-2.9708820000
H	0.7144880000	-0.1346310000	-3.7704340000
H	2.8925020000	-1.8629030000	-3.8631070000
O	-1.9133620000	-0.0982290000	-2.4162440000
H	-1.6288030000	0.2094670000	-3.3224770000
H	-2.7072620000	0.4328260000	-2.2263290000
F	-2.3058680000	1.8013660000	-0.5546140000
C	-1.7498940000	2.3145990000	-4.4569530000
O	-1.1069360000	1.2520430000	-4.5679770000
N	-1.2947570000	3.5145580000	-4.8480530000
H	-1.9509380000	4.2811400000	-4.7771100000
C	-0.0197540000	3.8009940000	-5.5612820000
C	-0.0059370000	5.3160160000	-5.7996610000
C	1.1786340000	3.3980020000	-4.6917660000
C	-0.0069920000	3.0473840000	-6.9003210000
H	-0.8616170000	5.6302860000	-6.4093520000
H	-0.0268720000	5.8649640000	-4.8510890000
H	0.9045730000	5.6003770000	-6.3338740000

H	1.1880480000	2.3193600000	-4.5226510000
H	2.1060470000	3.6780610000	-5.2018030000
H	1.1377390000	3.9080760000	-3.7257480000
H	0.9158010000	3.2733570000	-7.4442770000
H	-0.0590870000	1.9685140000	-6.7374110000
H	-0.8544570000	3.3509480000	-7.5240800000
C	-3.1737940000	2.2964570000	-3.9577560000
C	-4.0838330000	1.6054000000	-4.7568930000
C	-3.6108080000	2.9330690000	-2.7545670000
C	-5.4404130000	1.5688050000	-4.4272780000
H	-3.7262630000	1.1065420000	-5.6523370000
C	-4.9969610000	2.8845730000	-2.4522620000
C	-5.8945860000	2.2233440000	-3.2755040000
H	-6.1382960000	1.0387540000	-5.0674090000
H	-5.3551250000	3.3549840000	-1.5440910000
H	-6.9487810000	2.2014680000	-3.0179590000
C	-2.6691260000	3.5014770000	-1.8449410000
C	-3.0184680000	4.3799120000	-0.6999860000
H	-3.9173100000	4.0581320000	-0.1687550000
H	-2.1875310000	4.4136160000	0.0068350000
H	-3.1949560000	5.4044120000	-1.0641960000
H	-1.6160950000	3.4618640000	-2.1148400000
O	-1.4873050000	-0.8957330000	0.1541100000
H	-1.7616270000	-1.3238940000	-0.6781020000
H	-2.3013120000	-0.5059140000	0.5173060000

$^{5}Im_2Wat_2OH$_TS-3-OH

Charge: 1
Multiplicity: 5
B3LYP-D3(BJ)/6-31G(d)-SDD SCF energy (au): -1541.14678503
B3LYP-D3(BJ)/6-31G(d)-SDD enthalpy (au): -1540.61114203
B3LYP-D3(BJ)/6-31G(d)-SDD free energy (au): -1540.71889003
B3LYP-D3(BJ)/def2-TZVP/SMD SCF energy (au): -2681.65427957
B3LYP-D3(BJ)/def2-TZVP/SMD enthalpy (au): -2681.11863657
B3LYP-D3(BJ)/def2-TZVP/SMD free energy (au): -2681.22638457
B3LYP-D3(BJ)/def2-TZVP/SMD free energy (quasi-harmonic) (au): -2681.21638854

Cartesian coordinates

ATOM	X	Y	Z
Fe	0.2679990000	0.3922120000	-1.4093650000
C	1.2491170000	1.6557280000	2.6518910000
N	0.4751920000	2.6847350000	2.1448330000
H	0.2625590000	3.5535310000	2.6150610000
C	0.0421180000	2.3312940000	0.9134970000
H	-0.5713150000	2.9362550000	0.2642120000
N	0.5020380000	1.1237640000	0.6061960000
C	1.2588980000	0.6931720000	1.6802060000
H	1.7445720000	-0.2695390000	1.6761900000
O	1.8836330000	1.0435140000	-2.0940990000
H	2.2015700000	0.4913740000	-2.8287570000
H	1.7023590000	1.7037120000	3.6293650000
C	1.5444830000	-3.6744750000	-0.4467490000
N	2.7344250000	-2.9705780000	-0.4375810000

H	3.6541130000	-3.3624590000	-0.2877600000
C	2.4615230000	-1.6687250000	-0.6891710000
H	3.2002540000	-0.8843180000	-0.7523470000
N	1.1551350000	-1.5034030000	-0.8492130000
C	0.5709280000	-2.7477130000	-0.7042490000
H	-0.4938370000	-2.8803990000	-0.8113260000
H	1.5045490000	-4.7390730000	-0.2787690000
O	-0.2297600000	-0.4366630000	-3.2936230000
H	-0.2914350000	0.3409770000	-3.9339710000
H	-1.1116510000	-0.8428320000	-3.2529220000
F	-1.1737990000	1.6404140000	-1.5125720000
C	-0.7380050000	2.7084150000	-4.9260910000
O	-0.3553790000	1.5222470000	-5.0179130000
N	-1.4143940000	3.3148090000	-5.9193210000
H	-1.5881740000	4.3042660000	-5.8047860000
C	-1.8495340000	2.7016970000	-7.2022630000
C	-2.6175330000	3.7966770000	-7.9531060000
C	-0.6188860000	2.2642150000	-8.0119300000
C	-2.7757430000	1.5097600000	-6.9169820000
H	-3.4931010000	4.1288760000	-7.3831210000
H	-1.9772250000	4.6641520000	-8.1531940000
H	-2.9683230000	3.4126390000	-8.9148240000
H	-0.0488090000	1.5075880000	-7.4693650000
H	-0.9370810000	1.8424880000	-8.9709270000
H	0.0334500000	3.1204300000	-8.2142340000
H	-3.1376390000	1.0905980000	-7.8612670000
H	-2.2450260000	0.7276170000	-6.3710500000
H	-3.6426800000	1.8278840000	-6.3279970000
C	-0.4885700000	3.5638790000	-3.7222540000
C	-1.5667710000	4.2595080000	-3.1786590000
C	0.8113620000	3.6742840000	-3.1326500000
C	-1.4141540000	5.0630530000	-2.0472010000
H	-2.5500540000	4.1447890000	-3.6251090000
C	0.9388000000	4.5185830000	-1.9978780000
C	-0.1481590000	5.1948430000	-1.4652240000
H	-2.2700780000	5.5865810000	-1.6332430000
H	1.9150700000	4.6355500000	-1.5415070000
H	-0.0116520000	5.8381360000	-0.5999740000
C	1.9423370000	2.9992790000	-3.6610390000
C	3.3500950000	3.2673370000	-3.2441770000
H	3.4712340000	3.2208070000	-2.1574570000
H	4.0322950000	2.5376540000	-3.6879810000
H	3.6760670000	4.2657280000	-3.5738030000
H	1.7841360000	2.3700430000	-4.5285490000
O	-1.7748320000	-0.6953150000	-0.8744470000
H	-2.0235430000	0.2506570000	-1.0390850000
H	-1.8543900000	-0.8176600000	0.0852610000

$^{5}Im_2Wat_3$_11

Charge: 2
Multiplicity: 5
B3LYP-D3(BJ)/6-31G(d)-SDD SCF energy (au): -1541.41077592
B3LYP-D3(BJ)/6-31G(d)-SDD enthalpy (au): -1540.85962092

```
B3LYP-D3(BJ)/6-31G(d)-SDD free energy (au):             -1540.97374692
B3LYP-D3(BJ)/def2-TZVP/SMD SCF energy (au):             -2682.02920429
B3LYP-D3(BJ)/def2-TZVP/SMD enthalpy (au):               -2681.47804929
B3LYP-D3(BJ)/def2-TZVP/SMD free energy (au):            -2681.59217529
B3LYP-D3(BJ)/def2-TZVP/SMD free energy (quasi-harmonic) (au): -2681.58042037

Cartesian coordinates
ATOM    X               Y               Z
Fe     0.3443720000   -1.0430780000   -0.7976230000
C      1.6863430000    2.0969600000    1.8199650000
N      1.8557060000    2.6945560000    0.5861100000
H      2.2354250000    3.6190730000    0.4252850000
C      1.4561610000    1.8300630000   -0.3714510000
H      1.4829170000    2.0378410000   -1.4291740000
N      1.0340250000    0.6991590000    0.1877560000
C      1.1763540000    0.8548810000    1.5586010000
H      0.9223980000    0.0643400000    2.2480830000
O      1.8277010000   -1.0023850000   -2.3845030000
H      1.9254300000   -1.8699430000   -2.8145640000
H      1.4711340000   -0.4030230000   -3.0654890000
H      1.9449000000    2.5939700000    2.7419720000
C      1.8911490000   -4.0944350000    1.8280070000
N      3.0497900000   -3.4774340000    1.4002480000
H      3.9912180000   -3.7382940000    1.6673190000
C      2.7196020000   -2.4733330000    0.5603560000
H      3.4347950000   -1.8222400000    0.0801170000
N      1.3972230000   -2.4103370000    0.4230990000
C      0.8699900000   -3.4218820000    1.2141590000
H     -0.1944710000   -3.5872880000    1.2891450000
H      1.9009080000   -4.9305960000    2.5097890000
O     -0.4227930000   -2.7252600000   -2.0057780000
H     -1.2323880000   -2.4608860000   -2.4763360000
H     -0.6303920000   -3.5500760000   -1.5349770000
C     -5.1843820000    1.3509630000   -3.0936400000
C     -3.8344900000    1.1102460000   -3.3340670000
C     -2.8807530000    1.4411680000   -2.3622840000
C     -3.2569930000    1.9843460000   -1.1176830000
C     -4.6213330000    2.2232140000   -0.9076470000
C     -5.5746650000    1.9192320000   -1.8789020000
H     -5.9238660000    1.0985810000   -3.8462890000
H     -3.5176560000    0.6723190000   -4.2767550000
H     -4.9383300000    2.6544340000    0.0380320000
H     -6.6238130000    2.1171830000   -1.6842290000
C     -2.2547230000    2.3419870000   -0.0365620000
C     -1.6786420000    3.7624640000   -0.1622900000
H     -1.4231780000    1.6296710000   -0.0283110000
H     -2.7524410000    2.2531080000    0.9359260000
H     -1.0423800000    3.9917720000    0.6985950000
H     -1.0728400000    3.8648860000   -1.0682340000
H     -2.4765190000    4.5100370000   -0.2020300000
C     -1.4585450000    1.0737640000   -2.6134410000
O     -1.0066880000   -0.0047250000   -2.1617230000
N     -0.6761730000    1.9323390000   -3.2928740000
```

C	-0.8232450000	3.0952500000	-4.2534880000
C	-2.1317180000	3.8562240000	-4.0021210000
C	-0.7887580000	2.5227750000	-5.6767180000
H	-2.0886110000	4.7558790000	-4.6226000000
H	-3.0137690000	3.2901080000	-4.2957900000
H	-2.2397150000	4.1704970000	-2.9629680000
H	0.1401910000	1.9806180000	-5.8695030000
H	-1.6369730000	1.8527430000	-5.8484280000
H	-0.8562020000	3.3450950000	-6.3944610000
F	0.6262480000	1.3880390000	-3.4636380000
C	0.3660150000	4.0326270000	-3.9882490000
H	0.3504550000	4.4015400000	-2.9572990000
H	1.3240350000	3.5485530000	-4.1857160000
H	0.2830620000	4.8949750000	-4.6542980000
O	-1.5742760000	-1.1741980000	0.2962770000
H	-1.6995980000	-1.0198670000	1.2470440000
H	-2.1930600000	-0.5817730000	-0.1685250000

$^5Im_2Wat_3\_13$
Charge: 2
Multiplicity: 5
B3LYP-D3(BJ)/6-31G(d)-SDD SCF energy (au): -1541.47036790
B3LYP-D3(BJ)/6-31G(d)-SDD enthalpy (au): -1540.91940290
B3LYP-D3(BJ)/6-31G(d)-SDD free energy (au): -1541.03258790
B3LYP-D3(BJ)/def2-TZVP/SMD SCF energy (au): -2682.07872557
B3LYP-D3(BJ)/def2-TZVP/SMD enthalpy (au): -2681.52776057
B3LYP-D3(BJ)/def2-TZVP/SMD free energy (au): -2681.64094557
B3LYP-D3(BJ)/def2-TZVP/SMD free energy (quasi-harmonic) (au): -2681.62973867

Cartesian coordinates

ATOM	X	Y	Z
Fe	-0.0195520000	0.6318740000	-1.1762060000
C	2.2482340000	1.9190960000	2.3241040000
N	2.6668940000	2.7777370000	1.3271160000
H	3.2950190000	3.5618000000	1.4475260000
C	2.0900260000	2.3992290000	0.1641840000
H	2.2343790000	2.8987180000	-0.7813640000
N	1.3199570000	1.3359830000	0.3610600000
C	1.4094600000	1.0290960000	1.7097630000
H	0.8738200000	0.1963870000	2.1401220000
O	1.4311440000	1.0755260000	-2.7637560000
H	1.9423830000	0.2890460000	-3.0172110000
H	0.8960510000	1.3286460000	-3.5542750000
H	2.5754190000	2.0192640000	3.3471770000
C	1.0370210000	-3.5850850000	-1.2558200000
N	2.0252440000	-3.0833380000	-0.4307810000
H	2.7656460000	-3.6214160000	0.0012220000
C	1.8394910000	-1.7522720000	-0.2979830000
H	2.4685140000	-1.1074850000	0.2957060000
N	0.7745560000	-1.3689260000	-0.9990610000
C	0.2662180000	-2.5101690000	-1.6042100000
H	-0.5982180000	-2.4576380000	-2.2456550000
H	0.9772130000	-4.6313600000	-1.5116360000

O	-1.3426690000	-0.2005640000	-2.7518200000
H	-1.2409620000	0.4162310000	-3.5169680000
H	-2.2363570000	-0.0617710000	-2.3974570000
F	-0.8962680000	2.2587910000	-1.3743490000
C	-1.0456220000	2.6144120000	-5.0749270000
O	-0.4760150000	1.5926920000	-4.6303770000
N	-0.4430560000	3.6037430000	-5.7387070000
H	-1.0249570000	4.4033870000	-5.9591110000
C	1.0123550000	3.7699970000	-6.0557550000
C	1.0871420000	4.9700820000	-7.0090930000
C	1.7796580000	4.0745160000	-4.7603590000
C	1.5517430000	2.5138790000	-6.7524750000
H	0.5272680000	4.7811430000	-7.9318470000
H	0.6980550000	5.8806090000	-6.5379270000
H	2.1277200000	5.1604540000	-7.2832890000
H	1.7419700000	3.2383250000	-4.0586230000
H	2.8302110000	4.2695660000	-4.9970580000
H	1.3711780000	4.9623430000	-4.2671680000
H	2.5990180000	2.6785300000	-7.0216560000
H	1.4956990000	1.6337920000	-6.1097040000
H	0.9990710000	2.3084450000	-7.6746160000
C	-2.5200980000	2.8063830000	-4.8399730000
C	-2.9190560000	2.9724210000	-3.5236420000
C	-3.4900120000	2.9059650000	-5.9043240000
C	-4.2546990000	3.3027850000	-3.2262270000
H	-2.1881150000	2.8757140000	-2.7203920000
C	-4.8292770000	3.2898130000	-5.5574750000
C	-5.2045340000	3.4796450000	-4.2417000000
H	-4.5427570000	3.4471430000	-2.1892190000
H	-5.5625340000	3.3850750000	-6.3526600000
H	-6.2249560000	3.7546600000	-3.9976880000
C	-3.2747790000	2.5483200000	-7.2372310000
C	-2.1278310000	1.8591240000	-7.8614420000
H	-1.4202620000	1.4208360000	-7.1599690000
H	-2.5097510000	1.0768050000	-8.5311920000
H	-1.5910340000	2.5604070000	-8.5210860000
H	-4.1020400000	2.7618300000	-7.9134300000
O	-1.7390910000	0.0780910000	0.1520710000
H	-2.0054290000	0.8905100000	0.6163060000
H	-1.6517360000	-0.6163330000	0.8255330000

$^5Im_2Wat_3$_FeF_remove-F

Charge: 2
Multiplicity: 5
B3LYP-D3(BJ)/6-31G(d)-SDD SCF energy (au): -805.286859892
B3LYP-D3(BJ)/6-31G(d)-SDD enthalpy (au): -805.045067892
B3LYP-D3(BJ)/6-31G(d)-SDD free energy (au): -805.119312892
B3LYP-D3(BJ)/def2-TZVP/SMD SCF energy (au): -1945.64142437
B3LYP-D3(BJ)/def2-TZVP/SMD enthalpy (au): -1945.39963237
B3LYP-D3(BJ)/def2-TZVP/SMD free energy (au): -1945.47387737
B3LYP-D3(BJ)/def2-TZVP/SMD free energy (quasi-harmonic) (au): -1945.467435478

Cartesian coordinates

ATOM	X	Y	Z
Fe	0.7890410000	-0.5303620000	-0.6914240000
C	-0.0337380000	2.1328810000	2.4983000000
N	1.1741890000	2.6208260000	2.0395540000
H	1.6696650000	3.4207400000	2.4174870000
C	1.5863560000	1.8671670000	1.0062240000
H	2.5068580000	2.0249620000	0.4668960000
N	0.6923180000	0.9015230000	0.7668220000
C	-0.3262920000	1.0629130000	1.7023670000
H	-1.1776650000	0.4029140000	1.7354490000
O	2.7571850000	0.0539580000	-1.4272730000
H	3.5023550000	-0.5540640000	-1.2811570000
H	3.0645940000	0.7429000000	-2.0410570000
H	-0.5607100000	2.5803120000	3.3268940000
C	2.4379750000	-4.4711980000	-0.3617190000
N	2.3740280000	-4.1029360000	0.9652640000
H	2.6709670000	-4.6706660000	1.7508400000
C	1.8530420000	-2.8617240000	1.0486860000
H	1.7003280000	-2.3313270000	1.9771200000
N	1.5758000000	-2.4041320000	-0.1738540000
C	1.9404380000	-3.4081570000	-1.0645950000
H	1.8094740000	-3.2999890000	-2.1306940000
H	2.8178460000	-5.4304180000	-0.6783040000
O	0.1593960000	-0.5601900000	-2.6941730000
H	0.6416420000	-0.3183990000	-3.5029110000
H	-0.7079710000	-0.9159140000	-2.9534550000
O	-1.2427170000	-1.3149900000	-0.4002930000
H	-1.3753450000	-2.1993810000	-0.0182210000
H	-2.1057850000	-0.8662090000	-0.3945520000

^{6}Im$_2$Wat$_3$_FeF

Charge: 2
Multiplicity: 6
B3LYP-D3(BJ)/6-31G(d)-SDD SCF energy (au): -905.123838114
B3LYP-D3(BJ)/6-31G(d)-SDD enthalpy (au): -904.877612114
B3LYP-D3(BJ)/6-31G(d)-SDD free energy (au): -904.948797114
B3LYP-D3(BJ)/def2-TZVP/SMD SCF energy (au): -2045.52474341
B3LYP-D3(BJ)/def2-TZVP/SMD enthalpy (au): -2045.27851741
B3LYP-D3(BJ)/def2-TZVP/SMD free energy (au): -2045.34970241
B3LYP-D3(BJ)/def2-TZVP/SMD free energy (quasi-harmonic) (au): -2045.346362296

Cartesian coordinates

ATOM	X	Y	Z
Fe	0.6212690000	-0.3050430000	-0.9737230000
C	0.3150530000	1.6464850000	2.7941680000
N	1.4663810000	2.1992330000	2.2662810000
H	2.0171810000	2.9368360000	2.6924840000
C	1.7308850000	1.6192830000	1.0850200000
H	2.5690990000	1.8668170000	0.4533230000
N	0.7947100000	0.6996480000	0.8213240000
C	-0.1009960000	0.7139710000	1.8874440000
H	-0.9707820000	0.0779960000	1.9090040000
O	2.5754190000	0.3046490000	-1.5753870000

H	3.3070820000	-0.3281620000	-1.6897690000
H	2.5789180000	0.9104620000	-2.3409550000
H	-0.0975670000	1.9636130000	3.7397070000
C	2.5249380000	-4.0569090000	-0.1325750000
N	2.1454470000	-3.6290350000	1.1248620000
H	2.3265810000	-4.1194190000	1.9944320000
C	1.5013490000	-2.4553710000	1.0132020000
H	1.1117030000	-1.8909050000	1.8465650000
N	1.4362340000	-2.0983540000	-0.2756990000
C	2.0807730000	-3.0994720000	-0.9996670000
H	2.1855420000	-3.0536680000	-2.0722980000
H	3.0634160000	-4.9788280000	-0.2919570000
O	0.3896710000	-1.3907470000	-2.8796850000
H	-0.0058440000	-0.7983860000	-3.5464270000
H	0.0711480000	-2.2913210000	-3.0637610000
F	0.0124190000	1.0385550000	-1.9801310000
O	-1.3748210000	-0.8384740000	-0.4283010000
H	-1.7399400000	-1.7397420000	-0.4771870000
H	-2.0466250000	-0.2262760000	-0.7863420000

^{5}Im$_2$Wat$_3$_TS-1
Charge: 2
Multiplicity: 5
B3LYP-D3(BJ)/6-31G(d)-SDD SCF energy (au): -1541.39191887
B3LYP-D3(BJ)/6-31G(d)-SDD enthalpy (au): -1540.84375687
B3LYP-D3(BJ)/6-31G(d)-SDD free energy (au): -1540.95204187
B3LYP-D3(BJ)/def2-TZVP/SMD SCF energy (au): -2682.00860728
B3LYP-D3(BJ)/def2-TZVP/SMD enthalpy (au): -2681.46044528
B3LYP-D3(BJ)/def2-TZVP/SMD free energy (au): -2681.56873028
B3LYP-D3(BJ)/def2-TZVP/SMD free energy (quasi-harmonic) (au): -2681.55967541

Cartesian coordinates

ATOM	X	Y	Z
Fe	0.6111460000	-0.2947400000	-0.9807000000
C	0.1966610000	1.8541370000	2.7074650000
N	1.4817470000	2.1895660000	2.3253410000
H	2.0981070000	2.8263860000	2.8152270000
C	1.7816440000	1.5291410000	1.1886920000
H	2.7187440000	1.6096580000	0.6601810000
N	0.7474830000	0.7754060000	0.8200010000
C	-0.2530450000	0.9744170000	1.7619450000
H	-1.2082410000	0.4822980000	1.6762650000
O	2.6527180000	0.2794420000	-1.5466510000
H	3.3031760000	-0.4306830000	-1.6799230000
H	2.6483510000	0.8189640000	-2.3560110000
H	-0.2652490000	2.2606130000	3.5935950000
C	2.5943410000	-4.0788710000	-0.1991910000
N	2.2243330000	-3.6845270000	1.0694740000
H	2.4232000000	-4.1873840000	1.9259780000
C	1.5559020000	-2.5138310000	0.9806740000
H	1.1657730000	-1.9710440000	1.8286350000
N	1.4735830000	-2.1317910000	-0.2916090000
C	2.1233680000	-3.1054760000	-1.0383960000

H	2.2065320000	-3.0399080000	-2.1130860000
H	3.1415150000	-4.9902430000	-0.3845940000
O	0.2421540000	-1.2137760000	-2.9131810000
H	-0.5655230000	-0.6990750000	-3.1753960000
H	0.0136700000	-2.1541640000	-2.9994920000
C	-5.9345040000	2.1038370000	-3.0677890000
C	-4.6753010000	1.5491520000	-3.2111180000
C	-3.6406580000	1.9007710000	-2.3124660000
C	-3.8729230000	2.7895290000	-1.2282290000
C	-5.1624950000	3.3193000000	-1.1090740000
C	-6.1722190000	3.0032200000	-2.0161790000
H	-6.7301150000	1.8431200000	-3.7574760000
H	-4.4631600000	0.8510910000	-4.0144910000
H	-5.3819080000	3.9820880000	-0.2770410000
H	-7.1570530000	3.4441490000	-1.8971230000
C	-2.8510580000	3.1093260000	-0.1570500000
C	-2.2920340000	4.5372420000	-0.2466600000
H	-2.0237690000	2.3958380000	-0.1820300000
H	-3.3462690000	2.9842590000	0.8141220000
H	-1.6192130000	4.7350470000	0.5938350000
H	-1.7289260000	4.6747520000	-1.1740480000
H	-3.0917850000	5.2838610000	-0.2198080000
C	-2.3218000000	1.3144220000	-2.5990590000
O	-2.1251240000	0.1013640000	-2.7969490000
N	-1.3596410000	2.2954610000	-2.6114530000
C	-0.8763670000	2.9428310000	-3.8303850000
C	-2.0424150000	3.8896160000	-4.2732960000
C	-0.5759800000	1.9372320000	-4.9526740000
H	-1.6643950000	4.4733890000	-5.1174520000
H	-2.9171370000	3.3226880000	-4.5954160000
H	-2.3288250000	4.5707350000	-3.4702420000
H	0.2134450000	1.2457810000	-4.6484610000
H	-1.4640310000	1.3623580000	-5.2315150000
H	-0.2359280000	2.4820300000	-5.8377180000
F	0.1992730000	1.2718280000	-2.0658410000
C	0.3440730000	3.8010480000	-3.4742520000
H	0.1138680000	4.4841060000	-2.6524740000
H	1.1834800000	3.1701550000	-3.1784960000
H	0.6377410000	4.3910290000	-4.3464900000
O	-1.3981750000	-0.9308400000	-0.4246580000
H	-1.5186190000	-1.8643490000	-0.1835850000
H	-2.0020200000	-0.7382880000	-1.1787820000

^{5}Im$_2$Wat$_3$_TS-3-F
Charge: 2
Multiplicity: 5
B3LYP-D3(BJ)/6-31G(d)-SDD SCF energy (au): -1541.47459734
B3LYP-D3(BJ)/6-31G(d)-SDD enthalpy (au): -1540.92533834
B3LYP-D3(BJ)/6-31G(d)-SDD free energy (au): -1541.03668634
B3LYP-D3(BJ)/def2-TZVP/SMD SCF energy (au): -2682.08321435
B3LYP-D3(BJ)/def2-TZVP/SMD enthalpy (au): -2681.53395535
B3LYP-D3(BJ)/def2-TZVP/SMD free energy (au): -2681.64530335
B3LYP-D3(BJ)/def2-TZVP/SMD free energy (quasi-harmonic) (au): -2681.63477801

Cartesian coordinates

ATOM	X	Y	Z
Fe	-0.0850670000	0.5774010000	-1.0041760000
C	1.9834590000	1.3350250000	2.7798070000
N	2.6212190000	2.1927260000	1.9057990000
H	3.3503640000	2.8502020000	2.1497610000
C	2.1013570000	2.0076100000	0.6693610000
H	2.4144520000	2.5462980000	-0.2120780000
N	1.1607380000	1.0721570000	0.7024790000
C	1.0778240000	0.6467290000	2.0178230000
H	0.3778890000	-0.1159660000	2.3237290000
O	1.3863720000	1.5501050000	-2.4103280000
H	1.9607430000	0.8423890000	-2.7473610000
H	0.8465670000	1.8464850000	-3.1770410000
H	2.2240100000	1.2984840000	3.8308200000
C	1.5156060000	-3.3448110000	-2.0182230000
N	2.2872610000	-3.0185610000	-0.9193740000
H	3.0062050000	-3.5992890000	-0.5073410000
C	1.9182780000	-1.7932800000	-0.4849510000
H	2.3553140000	-1.2936550000	0.3655220000
N	0.9439820000	-1.3114970000	-1.2518930000
C	0.6841670000	-2.2763250000	-2.2151100000
H	-0.0767730000	-2.1187680000	-2.9621370000
H	1.6268740000	-4.2816840000	-2.5412700000
O	-1.2660900000	-0.0201360000	-2.8185010000
H	-1.2735090000	0.7918650000	-3.3714050000
H	-2.1511170000	-0.1067530000	-2.4275770000
F	-1.2139670000	2.0994260000	-0.8919070000
C	-1.0668030000	3.1375700000	-4.9264020000
O	-0.5425510000	2.2403770000	-4.2360300000
N	-0.4346520000	3.8936550000	-5.8241560000
H	-0.9884070000	4.6072980000	-6.2836920000
C	1.0097660000	3.8248840000	-6.2234490000
C	1.1673760000	4.8252270000	-7.3752440000
C	1.8857540000	4.2439890000	-5.0336070000
C	1.3434750000	2.4053570000	-6.7025820000
H	0.5365130000	4.5522450000	-8.2284020000
H	0.9164770000	5.8440540000	-7.0575170000
H	2.2050210000	4.8321090000	-7.7180040000
H	1.7859640000	3.5571010000	-4.1896310000
H	2.9361540000	4.2489740000	-5.3396240000
H	1.6301440000	5.2549280000	-4.6987590000
H	2.3830320000	2.3755830000	-7.0418190000
H	1.2190370000	1.6702690000	-5.9046490000
H	0.7049080000	2.1187680000	-7.5439870000
C	-2.5480130000	3.3725700000	-4.7830240000
C	-3.3673840000	3.0937280000	-5.8635840000
C	-3.1444400000	3.7034200000	-3.5172580000
C	-4.7692530000	3.1142200000	-5.7306920000
H	-2.9263840000	2.8237910000	-6.8180400000
C	-4.5664280000	3.6700740000	-3.4058690000
C	-5.3703240000	3.3902620000	-4.5000890000

H	-5.3865430000	2.8949780000	-6.5967660000
H	-5.0190830000	3.9110790000	-2.4486400000
H	-6.4504280000	3.3898790000	-4.4032820000
C	-2.4526500000	4.1861740000	-2.3920270000
C	-1.0612680000	4.5648730000	-2.2247400000
H	-0.4847340000	4.7106740000	-3.1351620000
H	-0.9723490000	5.4231930000	-1.5495970000
H	-0.6757460000	3.6855750000	-1.6485590000
H	-3.0629210000	4.3009440000	-1.4974240000
O	-1.8267950000	-0.4630420000	0.0293330000
H	-2.2386980000	0.2057750000	0.6021330000
H	-1.7130790000	-1.2631300000	0.5674340000

$^{5}Im_3OHN_3$_11
Charge: 0
Multiplicity: 5
B3LYP-D3(BJ)/6-31G(d)-SDD SCF energy (au): -1778.85087037
B3LYP-D3(BJ)/6-31G(d)-SDD enthalpy (au): -1778.27440437
B3LYP-D3(BJ)/6-31G(d)-SDD free energy (au): -1778.39363737
B3LYP-D3(BJ)/def2-TZVP/SMD SCF energy (au): -2919.40274584
B3LYP-D3(BJ)/def2-TZVP/SMD enthalpy (au): -2918.82627984
B3LYP-D3(BJ)/def2-TZVP/SMD free energy (au): -2918.94551284
B3LYP-D3(BJ)/def2-TZVP/SMD free energy (quasi-harmonic) (au): -2918.93436047

Cartesian coordinates

ATOM	X	Y	Z
Fe	1.1474490000	0.1577120000	0.7495890000
C	-0.1085660000	1.5685710000	-3.2527010000
N	0.9856850000	0.7827700000	-3.5667310000
H	1.2845420000	0.5206550000	-4.4939510000
C	1.5352070000	0.3377980000	-2.4067340000
H	2.3699180000	-0.3476150000	-2.3641740000
N	0.8572600000	0.8078320000	-1.3722130000
C	-0.1709130000	1.5743570000	-1.8858370000
H	-0.8865550000	2.0463890000	-1.2348240000
C	0.1314440000	4.4673660000	1.6427450000
N	-0.0850440000	3.7490180000	2.8065330000
H	-0.4297940000	4.1193630000	3.6795920000
C	0.2457390000	2.4506250000	2.5704100000
H	0.2110930000	1.6405420000	3.2857000000
N	0.6595500000	2.3039820000	1.3239050000
C	0.5951620000	3.5505670000	0.7348610000
H	0.8838540000	3.6966720000	-0.2950980000
H	-0.0596920000	5.5271010000	1.5765790000
H	-0.7343430000	2.0122780000	-4.0097840000
C	5.2863820000	1.5868290000	1.1116650000
N	5.5306030000	0.4059250000	0.4324810000
H	6.4361900000	0.0169770000	0.2157700000
C	4.3325770000	-0.1574060000	0.1194670000
H	4.2275820000	-1.0871400000	-0.4263430000
N	3.3413870000	0.6027240000	0.5563800000
C	3.9225500000	1.6905200000	1.1778350000
H	3.3118790000	2.4528340000	1.6351420000

H	6.0836460000	2.2154890000	1.4760820000
O	1.1327950000	-0.2242360000	2.6326290000
H	1.4776950000	-1.1216550000	2.7548060000
C	-1.3455630000	-1.6952980000	-3.5505220000
C	-1.2060360000	-1.7176190000	-2.1646390000
C	-2.1221720000	-1.0332290000	-1.3629160000
C	-3.2019080000	-0.3328750000	-1.9249500000
C	-3.3229360000	-0.3265170000	-3.3193370000
C	-2.4050850000	-0.9947760000	-4.1294220000
H	-0.6311620000	-2.2285500000	-4.1714320000
H	-0.3848000000	-2.2404650000	-1.6872950000
H	-4.1568000000	0.2059060000	-3.7715260000
H	-2.5265360000	-0.9797590000	-5.2096140000
C	-4.1983310000	0.4178270000	-1.0675300000
C	-3.7253360000	1.8327080000	-0.6902380000
H	-4.4010550000	-0.1486020000	-0.1510410000
H	-5.1497770000	0.4852020000	-1.6086640000
H	-3.5048200000	2.4201850000	-1.5892910000
H	-4.4987820000	2.3607260000	-0.1206330000
H	-2.8203640000	1.7837550000	-0.0786940000
C	-1.8869560000	-0.9864230000	0.1213880000
O	-1.2774540000	-0.0581470000	0.6577780000
N	-2.4825640000	-1.9719980000	0.8566090000
C	-2.1779090000	-2.5020720000	2.2129060000
C	-1.8671510000	-1.3298670000	3.1571080000
C	-0.9887900000	-3.4684270000	2.1134570000
H	-2.6489910000	-0.5652830000	3.0992590000
H	-1.8618980000	-1.7334050000	4.1768220000
H	-0.8935040000	-0.8756450000	2.9526090000
H	-1.2376180000	-4.3203970000	1.4730750000
H	-0.1136460000	-2.9644490000	1.6946770000
H	-0.7435630000	-3.8478080000	3.1115930000
F	-2.9568110000	-3.0177430000	0.0362120000
C	-3.4456780000	-3.2253380000	2.6965920000
H	-4.2915090000	-2.5312410000	2.7460660000
H	-3.7126090000	-4.0568150000	2.0409530000
H	-3.2639920000	-3.6209210000	3.7005580000
N	2.9213140000	-2.5341540000	-1.5694130000
N	2.1136950000	-2.2399600000	-0.7694420000
N	1.2815920000	-1.9216580000	0.0290150000

$^{5}Im_{3}OHN_{3}$_13

Charge: 0
Multiplicity: 5
B3LYP-D3(BJ)/6-31G(d)-SDD SCF energy (au): -1778.90731059
B3LYP-D3(BJ)/6-31G(d)-SDD enthalpy (au): -1778.33179559
B3LYP-D3(BJ)/6-31G(d)-SDD free energy (au): -1778.45313159
B3LYP-D3(BJ)/def2-TZVP/SMD SCF energy (au): -2919.47186337
B3LYP-D3(BJ)/def2-TZVP/SMD enthalpy (au): -2918.89634837
B3LYP-D3(BJ)/def2-TZVP/SMD free energy (au): -2919.01768437
B3LYP-D3(BJ)/def2-TZVP/SMD free energy (quasi-harmonic) (au): -2919.00419978

Cartesian coordinates

ATOM	X	Y	Z
H	33.1174460000	26.1231140000	36.2656960000
C	33.4014470000	27.1623930000	36.2130210000
C	34.6195600000	27.7876710000	36.2117300000
N	32.4672340000	28.1785010000	36.1226940000
C	33.1322760000	29.3595470000	36.0545710000
N	34.4380080000	29.1542230000	36.1166870000
H	31.4710440000	28.0663950000	36.0073710000
H	35.6085800000	27.3613640000	36.2616330000
H	32.6605470000	30.3203600000	35.9301120000
H	39.9575000000	27.2098870000	36.5324760000
C	39.1809160000	27.8001780000	36.0713890000
C	38.1436480000	28.5341240000	36.5870260000
N	39.0783350000	27.9402220000	34.6979920000
C	38.0049860000	28.7354730000	34.4368640000
N	37.4207640000	29.1039830000	35.5595690000
H	39.6990710000	27.5412550000	34.0096630000
H	37.8658590000	28.6939470000	37.6178990000
H	37.6779390000	29.0363620000	33.4527470000
H	36.3322630000	30.7889810000	40.9214290000
C	36.4577250000	30.9462610000	39.8615970000
C	35.8050530000	30.4406370000	38.7675730000
N	37.4182250000	31.8053320000	39.3522140000
C	37.3185150000	31.7934380000	37.9971720000
N	36.3547120000	30.9738200000	37.6230060000
H	38.0634710000	32.3638180000	39.8906810000
H	34.9839660000	29.7432910000	38.7127090000
H	37.9254130000	32.3619940000	37.3093690000
Fe	35.8255490000	30.8730370000	35.4616480000
O	34.4209040000	32.0422770000	35.7209470000
H	33.7061420000	31.9850330000	35.0557240000
F	37.3279620000	31.9187630000	35.1835520000
N	31.2702130000	31.2822540000	31.9702920000
O	32.1680220000	31.6925920000	34.0169040000
C	33.4745220000	32.2947210000	32.1049370000
C	34.0545930000	33.3802920000	32.7593370000
C	35.1479720000	34.0571350000	32.2215200000
C	35.6713830000	33.6451330000	30.9903600000
C	35.1260870000	32.5584370000	30.3294150000
C	34.0329990000	31.8169770000	30.8670690000
C	32.2710520000	31.7105760000	32.7785800000
C	30.0159580000	30.6400200000	32.4075490000
C	30.3214170000	29.4042100000	33.2717990000
C	29.1613030000	31.6504980000	33.1908570000
C	29.2886710000	30.2092590000	31.1271060000
C	33.6030640000	30.6300640000	30.2238210000
C	34.1694970000	30.1274910000	28.9325300000
H	33.6433940000	33.6917090000	33.7118350000
H	35.5876990000	34.8898440000	32.7604040000
H	36.5211490000	34.1664870000	30.5578670000
H	35.5715230000	32.2271730000	29.3976920000
H	31.4216670000	31.3731570000	30.9756040000
H	32.9633590000	29.9509770000	30.7723310000

H	35.1923670000	29.7382540000	29.0613410000
H	33.5623070000	29.3073310000	28.5371920000
H	34.2240830000	30.9083190000	28.1630400000
H	29.3867590000	28.8819380000	33.5093570000
H	30.9967680000	28.7216290000	32.7493040000
H	30.8100590000	29.7110730000	34.1970870000
H	29.7085100000	32.0018380000	34.0681310000
H	28.9153960000	32.5139610000	32.5630060000
H	28.2250570000	31.1833230000	33.5179980000
H	29.8856500000	29.4833990000	30.5624980000
H	28.3313990000	29.7414660000	31.3764490000
H	29.0841100000	31.0718460000	30.4810980000
N	35.5278690000	30.1720220000	33.5507220000
N	34.6187840000	29.4555590000	33.2246920000
N	33.7517150000	28.7656080000	32.8732890000

$^{5}Im_3OHN_3$_FeF_remove-F

Charge: 0
Multiplicity: 5
B3LYP-D3(BJ)/6-31G(d)-SDD SCF energy (au): -1042.77025016
B3LYP-D3(BJ)/6-31G(d)-SDD enthalpy (au): -1042.50234616
B3LYP-D3(BJ)/6-31G(d)-SDD free energy (au): -1042.58180416
B3LYP-D3(BJ)/def2-TZVP/SMD SCF energy (au): -2183.03131501
B3LYP-D3(BJ)/def2-TZVP/SMD enthalpy (au): -2182.76341101
B3LYP-D3(BJ)/def2-TZVP/SMD free energy (au): -2182.84286901
B3LYP-D3(BJ)/def2-TZVP/SMD free energy (quasi-harmonic) (au): -2182.83679385

Cartesian coordinates

ATOM	X	Y	Z
Fe	0.4786150000	-0.2373030000	-1.3442140000
C	0.1728390000	0.7685970000	2.9912350000
N	0.5388300000	1.9887100000	2.4524980000
H	0.8617440000	2.7948560000	2.9670210000
C	0.5261870000	1.8705670000	1.1006660000
H	0.8229560000	2.6527790000	0.4188160000
N	0.1493990000	0.6559760000	0.7438820000
C	-0.0661390000	-0.0452880000	1.9154750000
H	-0.3532260000	-1.0854230000	1.8932900000
C	-3.4891350000	-2.1023460000	-1.7825100000
N	-3.7250680000	-1.5583310000	-0.5310200000
H	-4.5857040000	-1.6173040000	-0.0076280000
C	-2.5957640000	-0.9014710000	-0.1457790000
H	-2.5025570000	-0.3720170000	0.7901300000
N	-1.6617140000	-1.0013460000	-1.0723080000
C	-2.2061370000	-1.7457970000	-2.1045220000
H	-1.6172140000	-1.9151700000	-2.9937830000
H	-4.2413210000	-2.6667570000	-2.3111990000
H	0.1260280000	0.6019680000	4.0559700000
C	1.6976610000	-4.1112060000	0.2999920000
N	2.7200060000	-3.2946860000	0.7512230000
H	3.5348770000	-3.5887280000	1.2690400000
C	2.4561820000	-2.0221060000	0.3521120000
H	3.0891770000	-1.1710250000	0.5743200000

N	1.3181480000	-1.9865670000	-0.3227650000
C	0.8350780000	-3.2791860000	-0.3636310000
H	-0.0866060000	-3.5080640000	-0.8766550000
H	1.6879970000	-5.1741890000	0.4829540000
O	0.2545450000	-0.7069910000	-3.1564140000
H	1.0541150000	-0.4509870000	-3.6411850000
N	3.6174610000	1.0932350000	0.4978500000
N	2.9100440000	1.0672150000	-0.4335620000
N	2.1632950000	1.0508320000	-1.3735880000

$^{5}Im_3OHN_3$_FeF_remove-N_3

Charge: 0
Multiplicity: 5
B3LYP-D3(BJ)/6-31G(d)-SDD SCF energy (au): -978.437199641
B3LYP-D3(BJ)/6-31G(d)-SDD enthalpy (au): -978.182543641
B3LYP-D3(BJ)/6-31G(d)-SDD free energy (au): -978.258219641
B3LYP-D3(BJ)/def2-TZVP/SMD SCF energy (au): -2118.66981038
B3LYP-D3(BJ)/def2-TZVP/SMD enthalpy (au): -2118.41515438
B3LYP-D3(BJ)/def2-TZVP/SMD free energy (au): -2118.49083038
B3LYP-D3(BJ)/def2-TZVP/SMD free energy (quasi-harmonic) (au): -2118.485611739

Cartesian coordinates

ATOM	X	Y	Z
Fe	-0.0906920000	-0.8289540000	-1.7987910000
C	0.6097590000	1.4010360000	3.5879920000
N	0.7788380000	1.7706090000	2.2659210000
H	0.5501720000	2.6632270000	1.8528490000
C	1.3218490000	0.7106000000	1.5987470000
H	1.4546750000	0.7263180000	0.5227980000
N	1.5108990000	-0.3117650000	2.4149210000
C	1.0734370000	0.1085930000	3.6543030000
H	1.1283080000	-0.5368270000	4.5205860000
C	-1.7658080000	-2.2133680000	1.9627750000
N	-1.7971650000	-0.8361660000	2.0962590000
H	-1.8318320000	-0.3233950000	2.9655390000
C	-1.4924420000	-0.2956520000	0.8892060000
H	-1.3878130000	0.7605760000	0.7049670000
N	-1.2930820000	-1.2440780000	-0.0086080000
C	-1.4638930000	-2.4477250000	0.6476770000
H	-1.3495320000	-3.3847650000	0.1244760000
H	-1.9681850000	-2.8741740000	2.7908350000
H	0.2280710000	2.0817530000	4.3337770000
C	2.7261190000	-4.0638230000	-1.3466190000
N	2.8200530000	-3.5475290000	-0.0648840000
H	3.4088540000	-3.8947610000	0.6772780000
C	1.9917660000	-2.4728300000	0.0254330000
H	1.8629900000	-1.8853430000	0.9289000000
N	1.3822540000	-2.2795510000	-1.1337410000
C	1.8269110000	-3.2632740000	-1.9979440000
H	1.4407270000	-3.3062780000	-3.0052280000
H	3.2918610000	-4.9267910000	-1.6608020000
O	-0.5760270000	-1.7617500000	-3.3407650000
H	-1.0783410000	-1.2859890000	-4.0166630000

F 0.4449270000 0.8567310000 -1.1933130000

$^{5}Im_3OHN_3$_FeF_remove-OH
Charge: 0
Multiplicity: 5
B3LYP-D3(BJ)/6-31G(d)-SDD SCF energy (au): -1066.82043566
B3LYP-D3(BJ)/6-31G(d)-SDD enthalpy (au): -1066.56367666
B3LYP-D3(BJ)/6-31G(d)-SDD free energy (au): -1066.64240966
B3LYP-D3(BJ)/def2-TZVP/SMD SCF energy (au): -2207.09170294
B3LYP-D3(BJ)/def2-TZVP/SMD enthalpy (au): -2206.83494394
B3LYP-D3(BJ)/def2-TZVP/SMD free energy (au): -2206.91367694
B3LYP-D3(BJ)/def2-TZVP/SMD free energy (quasi-harmonic) (au): -2206.90733428

Cartesian coordinates

ATOM	X	Y	Z
Fe	0.5163210000	-0.3038960000	-0.6386170000
C	1.3631560000	1.3021980000	3.3673890000
N	2.0365580000	2.1588470000	2.5133660000
H	2.6135920000	2.9416540000	2.7836200000
C	1.8268390000	1.7398550000	1.2419580000
H	2.2319750000	2.2158810000	0.3633800000
N	1.0520530000	0.6667940000	1.2347500000
C	0.7546490000	0.3841480000	2.5546310000
H	0.1218550000	-0.4495970000	2.8159610000
C	-2.4404630000	-2.6089680000	1.9915270000
N	-3.1799040000	-1.7081690000	1.2458710000
H	-4.1739640000	-1.5489320000	1.3186780000
C	-2.3340100000	-1.0730410000	0.3899770000
H	-2.6043050000	-0.3070430000	-0.3220610000
N	-1.0997170000	-1.5175270000	0.5483520000
C	-1.1513370000	-2.4750270000	1.5424570000
H	-0.2635720000	-3.0061400000	1.8534520000
H	-2.8893890000	-3.2403010000	2.7425130000
H	1.3808960000	1.4277090000	4.4385560000
C	1.9674220000	-4.4263160000	-0.9825670000
N	3.1598490000	-3.7407430000	-0.8316140000
H	4.0871020000	-4.1384510000	-0.8653380000
C	2.8789150000	-2.4258200000	-0.6518160000
H	3.6364890000	-1.6615380000	-0.5240030000
N	1.5665570000	-2.2362420000	-0.6741500000
C	0.9883250000	-3.4756970000	-0.8798010000
H	-0.0830290000	-3.5833650000	-0.9402580000
H	1.9326050000	-5.4912230000	-1.1495790000
F	-0.9395940000	0.4791500000	-1.4556530000
N	4.3834640000	0.4864590000	-0.6463030000
N	3.3106530000	0.5425320000	-1.1164500000
N	2.2111490000	0.5916690000	-1.5900200000

$^{6}Im_3OHN_3$_FeF
Charge: 0
Multiplicity: 6
B3LYP-D3(BJ)/6-31G(d)-SDD SCF energy (au): -1142.64169990
B3LYP-D3(BJ)/6-31G(d)-SDD enthalpy (au): -1142.36998490

B3LYP-D3(BJ)/6-31G(d)-SDD free energy (au): -1142.45093790
B3LYP-D3(BJ)/def2-TZVP/SMD SCF energy (au): -2282.94860223
B3LYP-D3(BJ)/def2-TZVP/SMD enthalpy (au): -2282.67688723
B3LYP-D3(BJ)/def2-TZVP/SMD free energy (au): -2282.75784023
B3LYP-D3(BJ)/def2-TZVP/SMD free energy (quasi-harmonic) (au): -2282.75221733

Cartesian coordinates

ATOM	X	Y	Z
Fe	0.1792580000	-0.3788940000	-0.9852090000
C	1.6232030000	0.9610590000	3.1197600000
N	2.0334410000	1.9670510000	2.2617710000
H	2.4898600000	2.8277700000	2.5255480000
C	1.7188820000	1.5919190000	0.9942740000
H	1.9075820000	2.1855090000	0.1127350000
N	1.1365870000	0.4084640000	0.9965210000
C	1.0645090000	0.0036840000	2.3131720000
H	0.6099590000	-0.9383450000	2.5804080000
C	-2.5219350000	-2.6651680000	1.7049750000
N	-3.1356020000	-1.4359720000	1.5294390000
H	-4.0354630000	-1.1617380000	1.8948610000
C	-2.3307090000	-0.6687490000	0.7508680000
H	-2.5324880000	0.3402950000	0.4256690000
N	-1.2404420000	-1.3406980000	0.4283660000
C	-1.3435360000	-2.5860820000	1.0104960000
H	-0.5736390000	-3.3293000000	0.8742060000
H	-2.9733290000	-3.4553420000	2.2841360000
H	1.7615790000	1.0251370000	4.1877280000
C	2.4232540000	-4.1704550000	-0.8860690000
N	3.1886200000	-3.4757000000	0.0369000000
H	4.0751420000	-3.7698550000	0.4190170000
C	2.5988650000	-2.2713120000	0.2495250000
H	3.0005740000	-1.5107230000	0.8979250000
N	1.4966700000	-2.1675660000	-0.4718150000
C	1.3760040000	-3.3434710000	-1.1910570000
H	0.5539200000	-3.4602140000	-1.8799810000
H	2.6946030000	-5.1566460000	-1.2285030000
O	-0.5006790000	-1.3752290000	-2.4142520000
H	-0.0523150000	-1.0931040000	-3.2282630000
F	-0.8870450000	1.1283880000	-0.8457680000
N	4.0347710000	0.0634010000	-1.1757250000
N	2.9448670000	0.2357140000	-1.5380080000
N	1.8159500000	0.4394620000	-1.9069840000

$^{5}Im_3OHN_3$_TS-1
Charge: 0
Multiplicity: 5
B3LYP-D3(BJ)/6-31G(d)-SDD SCF energy (au): -1778.82815305
B3LYP-D3(BJ)/6-31G(d)-SDD enthalpy (au): -1778.25406805
B3LYP-D3(BJ)/6-31G(d)-SDD free energy (au): -1778.37267705
B3LYP-D3(BJ)/def2-TZVP/SMD SCF energy (au): -2919.37921648
B3LYP-D3(BJ)/def2-TZVP/SMD enthalpy (au): -2918.80513148
B3LYP-D3(BJ)/def2-TZVP/SMD free energy (au): -2918.92374048
B3LYP-D3(BJ)/def2-TZVP/SMD free energy (quasi-harmonic) (au): -2918.91257943

Cartesian coordinates

ATOM	X	Y	Z
Fe	0.4401060000	-0.1494650000	-1.3728300000
C	-0.0360300000	0.5930410000	3.0832060000
N	0.4952530000	1.8101770000	2.6917790000
H	0.8631240000	2.5265380000	3.3003400000
C	0.5405220000	1.8288750000	1.3344680000
H	0.9361520000	2.6523580000	0.7629380000
N	0.0533670000	0.7069250000	0.8344790000
C	-0.3033590000	-0.0756620000	1.9180060000
H	-0.7028570000	-1.0672970000	1.7735550000
C	-3.4740300000	-2.0852920000	-1.8269860000
N	-3.8220260000	-1.2452900000	-0.7818640000
H	-4.7509590000	-1.1039970000	-0.4140830000
C	-2.6889130000	-0.6219390000	-0.3557880000
H	-2.6668310000	0.1093380000	0.4373770000
N	-1.6492370000	-1.0221010000	-1.0614020000
C	-2.1224170000	-1.9302380000	-1.9904280000
H	-1.4391240000	-2.3299250000	-2.7246500000
H	-4.2052500000	-2.6864470000	-2.3442940000
H	-0.1655240000	0.3301130000	4.1214030000
C	1.9283370000	-3.8718200000	0.4208060000
N	2.8005860000	-2.9629320000	0.9953960000
H	3.6106770000	-3.1838420000	1.5549460000
C	2.4061070000	-1.7127450000	0.6335520000
H	2.9238480000	-0.8077400000	0.9227610000
N	1.3309710000	-1.7792390000	-0.1322520000
C	1.0240350000	-3.1161520000	-0.2770490000
H	0.1909440000	-3.4273880000	-0.8869050000
H	2.0379800000	-4.9364780000	0.5547560000
O	0.5256070000	-1.2372110000	-2.8970360000
H	0.9444710000	-0.8048270000	-3.6551500000
C	0.8591010000	-0.0511500000	-6.1798300000
C	0.6767020000	1.1209080000	-5.4509490000
C	-0.5346380000	1.3838420000	-4.7964130000
C	-1.6070960000	0.4696810000	-4.8893470000
C	-1.4065380000	-0.6876570000	-5.6491500000
C	-0.1940850000	-0.9600650000	-6.2796960000
H	1.8105440000	-0.2498500000	-6.6639900000
H	1.4747520000	1.8508380000	-5.3681740000
H	-2.2251060000	-1.3976290000	-5.7368480000
H	-0.0720330000	-1.8807150000	-6.8435110000
C	-2.9697290000	0.7055270000	-4.2731050000
C	-3.7560600000	1.8262220000	-4.9709630000
H	-2.8623060000	0.9333250000	-3.2122520000
H	-3.5388260000	-0.2286040000	-4.3398830000
H	-4.7458150000	1.9472430000	-4.5145600000
H	-3.2330310000	2.7840400000	-4.8931250000
H	-3.8960610000	1.6044660000	-6.0350370000
C	-0.5852790000	2.7013580000	-4.0929250000
O	-0.0931000000	3.7117070000	-4.5913320000
N	-1.2925160000	2.7686050000	-2.8987520000

C	-1.1555260000	3.8819990000	-1.9481650000
C	-1.8440620000	5.0965680000	-2.6128320000
C	0.3088960000	4.1943680000	-1.6069800000
H	-1.8584990000	5.9232540000	-1.8933230000
H	-1.3065130000	5.4084860000	-3.5075790000
H	-2.8776400000	4.8534300000	-2.8806400000
H	0.8419450000	3.2781730000	-1.3369580000
H	0.8193450000	4.6327830000	-2.4664750000
H	0.3544650000	4.9055960000	-0.7734820000
F	-0.9091900000	1.3996200000	-2.0826340000
C	-1.9596730000	3.5053020000	-0.6926770000
H	-2.9824740000	3.2267310000	-0.9680530000
H	-1.5042450000	2.6679670000	-0.1648480000
H	-2.0033280000	4.3692110000	-0.0206030000
N	3.5475360000	1.4139860000	0.4219970000
N	2.8170820000	1.2782790000	-0.4775760000
N	2.0490820000	1.1585200000	-1.3934840000

[5]Im_3OHN_3_TS-3-F

Charge: 0
Multiplicity: 5
B3LYP-D3(BJ)/6-31G(d)-SDD SCF energy (au): -1778.89653373
B3LYP-D3(BJ)/6-31G(d)-SDD enthalpy (au): -1778.32085673
B3LYP-D3(BJ)/6-31G(d)-SDD free energy (au): -1778.43637273
B3LYP-D3(BJ)/def2-TZVP/SMD SCF energy (au): -2919.44846472
B3LYP-D3(BJ)/def2-TZVP/SMD enthalpy (au): -2918.87278772
B3LYP-D3(BJ)/def2-TZVP/SMD free energy (au): -2918.98830372
B3LYP-D3(BJ)/def2-TZVP/SMD free energy (quasi-harmonic) (au): -2918.97828499

Cartesian coordinates

ATOM	X	Y	Z
H	32.7208220000	25.9665630000	37.0550260000
C	33.0719900000	26.6787660000	36.3248840000
C	34.1100480000	27.5720190000	36.3298660000
N	32.4510630000	26.8432700000	35.0982070000
C	33.1266990000	27.7945100000	34.4061840000
N	34.1279440000	28.2611800000	35.1336820000
H	31.6995360000	26.2783770000	34.7317490000
H	34.8421810000	27.7711020000	37.0975330000
H	32.9072550000	28.1326220000	33.4045790000
H	38.3878370000	29.6747510000	38.7884860000
C	38.0164030000	29.3872660000	37.8172950000
C	37.0538850000	29.9203920000	37.0006560000
N	38.5144520000	28.2913580000	37.1322840000
C	37.8515680000	28.1972170000	35.9491430000
N	36.9630490000	29.1689480000	35.8473640000
H	39.2487580000	27.6747730000	37.4469480000
H	36.4227030000	30.7834860000	37.1419450000
H	38.0126130000	27.4424240000	35.1933850000
H	32.2080820000	33.2700690000	35.5477760000
C	33.1361460000	32.7592910000	35.3440620000
C	33.5814520000	31.4974140000	35.6476030000
N	34.1708500000	33.3635810000	34.6535630000

C	35.1745470000	32.4604770000	34.5440850000
N	34.8544720000	31.3200060000	35.1384490000
H	34.1211900000	34.1868160000	34.0585120000
H	33.0791870000	30.6993430000	36.1727990000
H	36.0887710000	32.6348500000	34.0012800000
Fe	35.6740330000	29.4227420000	34.0439370000
O	34.3765540000	29.7764590000	32.6377560000
H	34.1810350000	30.7238650000	32.6130040000
F	37.0236410000	30.6888730000	33.3090850000
N	34.3522970000	32.4687050000	30.8035240000
O	34.3481000000	34.3862680000	32.0322070000
C	36.4816940000	33.5085800000	31.4161200000
C	37.0023030000	34.8090380000	31.4523580000
C	38.3660650000	35.0613930000	31.3504300000
C	39.2502760000	33.9846130000	31.2187040000
C	38.7634610000	32.6883890000	31.2137640000
C	37.3800120000	32.3913210000	31.3203590000
C	34.9753960000	33.4800460000	31.4561730000
C	32.8948950000	32.3407160000	30.5519640000
C	32.4396560000	33.4821150000	29.6282870000
C	32.7152200000	30.9736640000	29.8766640000
C	32.1006670000	32.3682430000	31.8674090000
C	36.9777660000	31.0064420000	31.4293280000
C	37.8449390000	29.8608570000	31.0145460000
H	36.2965620000	35.6261590000	31.5501970000
H	38.7347660000	36.0825690000	31.3670710000
H	40.3192150000	34.1592450000	31.1335000000
H	39.4658670000	31.8665880000	31.1476270000
H	34.9374940000	31.8734310000	30.2346130000
H	35.9234800000	30.7619870000	31.4450180000
H	38.8634450000	29.9363180000	31.4041500000
H	37.4102270000	28.9296870000	31.3854780000
H	37.9053470000	29.8135460000	29.9165570000
H	31.3699500000	33.3890580000	29.4098660000
H	32.6173590000	34.4494170000	30.1053540000
H	32.9868200000	33.4523590000	28.6792650000
H	33.2543440000	30.9328180000	28.9210140000
H	33.0887800000	30.1788480000	30.5317150000
H	31.6556640000	30.7969880000	29.6670400000
H	32.2339470000	33.3195620000	32.3815990000
H	31.0366440000	32.2343470000	31.6423860000
H	32.4090050000	31.5556680000	32.5310410000
N	36.5048950000	27.6561830000	33.3388990000
N	35.7527700000	26.7528370000	33.0543360000
N	35.0461950000	25.8735460000	32.7861810000

$^{5}Im_3OHN_3$_TS-3-N_3

Charge: 0
Multiplicity: 5
B3LYP-D3(BJ)/6-31G(d)-SDD SCF energy (au): -1778.89107324
B3LYP-D3(BJ)/6-31G(d)-SDD enthalpy (au): -1778.31647324
B3LYP-D3(BJ)/6-31G(d)-SDD free energy (au): -1778.43443124
B3LYP-D3(BJ)/def2-TZVP/SMD SCF energy (au): -2919.45410297

B3LYP-D3(BJ)/def2-TZVP/SMD enthalpy (au): -2918.87950297
B3LYP-D3(BJ)/def2-TZVP/SMD free energy (au): -2918.99746097
B3LYP-D3(BJ)/def2-TZVP/SMD free energy (quasi-harmonic) (au): -2918.98560073

Cartesian coordinates

ATOM	X	Y	Z
H	31.2872020000	27.7496970000	37.0589890000
C	32.0662240000	28.4702530000	36.8648260000
C	33.3193780000	28.3544570000	36.3222270000
N	31.9169080000	29.8168030000	37.1523270000
C	33.0542830000	30.4571710000	36.7706010000
N	33.9263220000	29.5965120000	36.2725250000
H	31.0851960000	30.2634440000	37.5083580000
H	33.8140620000	27.4731900000	35.9447770000
H	33.2183630000	31.5238610000	36.8017480000
H	37.5410310000	25.5787100000	36.2178320000
C	37.2708790000	26.4945620000	35.7157560000
C	36.5402920000	27.5865460000	36.1075410000
N	37.6683190000	26.7685980000	34.4181780000
C	37.1737150000	27.9897700000	34.0785000000
N	36.4844540000	28.5016420000	35.0786790000
H	38.2140000000	26.1644360000	33.8221680000
H	36.0566740000	27.7849470000	37.0514620000
H	37.3445170000	28.4835130000	33.1351780000
H	38.1401750000	31.7216750000	39.5874320000
C	37.8890920000	31.5300100000	38.5559220000
C	36.7433340000	31.0873280000	37.9479070000
N	38.7966580000	31.7280050000	37.5278220000
C	38.1817350000	31.4081100000	36.3574130000
N	36.9453300000	31.0152630000	36.5888680000
H	39.7390540000	32.0746750000	37.6248500000
H	35.7901060000	30.8239560000	38.3802480000
H	38.6178740000	31.4703440000	35.3717720000
Fe	35.5984500000	30.6490670000	34.8735340000
O	34.7084890000	32.2504930000	35.2961610000
H	33.8545550000	32.3445050000	34.8401450000
F	37.1060380000	30.8718000000	33.7179920000
N	31.5029940000	31.1298980000	32.0471140000
O	31.9049100000	32.1006570000	34.0737540000
C	33.3579700000	32.6952810000	32.2891900000
C	33.3977390000	34.0592810000	32.5831150000
C	34.4806400000	34.8485390000	32.2052180000
C	35.5721600000	34.2494920000	31.5710380000
C	35.5632470000	32.8877540000	31.3048310000
C	34.4513470000	32.0747120000	31.6194110000
C	32.2024090000	31.9419200000	32.8833750000
C	30.3415580000	30.2972350000	32.4252850000
C	30.7388110000	29.3171050000	33.5408040000
C	29.1825480000	31.1997940000	32.8822250000
C	29.9424740000	29.5175960000	31.1663570000
C	34.4999630000	30.6522720000	31.3648000000
C	35.7472610000	29.9887490000	30.8637520000
H	32.5722840000	34.4905890000	33.1404340000

H	34.4877710000	35.9103490000	32.4323430000
H	36.4398780000	34.8439020000	31.2987460000
H	36.4317890000	32.4339860000	30.8429110000
H	31.7236190000	31.1954450000	31.0636690000
H	33.5684140000	30.1536420000	31.1261250000
H	36.5943800000	30.2745610000	31.4942060000
H	35.6316930000	28.8998490000	30.8930660000
H	35.9657960000	30.2648420000	29.8204510000
H	29.8631000000	28.7295700000	33.8412710000
H	31.5133810000	28.6250980000	33.1992580000
H	31.1161160000	29.8576480000	34.4090440000
H	29.4852360000	31.7827750000	33.7545380000
H	28.8935950000	31.8910050000	32.0822660000
H	28.3088200000	30.5921060000	33.1445170000
H	30.7651340000	28.8778420000	30.8261200000
H	29.0815790000	28.8764620000	31.3789370000
H	29.6626440000	30.1959450000	30.3501830000
N	34.4138760000	29.9011240000	33.2973090000
N	34.1631940000	28.7221150000	33.1710680000
N	33.9112620000	27.6050440000	33.0028660000

$^5Im_3OHN_3$_TS-3-OH

Charge: 0
Multiplicity: 5
B3LYP-D3(BJ)/6-31G(d)-SDD SCF energy (au): -1778.89402979
B3LYP-D3(BJ)/6-31G(d)-SDD enthalpy (au): -1778.31851379
B3LYP-D3(BJ)/6-31G(d)-SDD free energy (au): -1778.43335679
B3LYP-D3(BJ)/def2-TZVP/SMD SCF energy (au): -2919.45706218
B3LYP-D3(BJ)/def2-TZVP/SMD enthalpy (au): -2918.88154618
B3LYP-D3(BJ)/def2-TZVP/SMD free energy (au): -2918.99638918
B3LYP-D3(BJ)/def2-TZVP/SMD free energy (quasi-harmonic) (au): -2918.98622939

Cartesian coordinates

ATOM	X	Y	Z
H	-0.3883420000	-3.1885880000	-3.6500190000
C	-0.2069060000	-2.3467770000	-3.0008040000
C	0.6856820000	-2.1446960000	-1.9812760000
N	-0.9289360000	-1.1721610000	-3.0978730000
C	-0.4854410000	-0.3213350000	-2.1422090000
N	0.5016660000	-0.8789610000	-1.4606230000
H	-1.7715430000	-1.0334920000	-3.6366850000
H	1.4346320000	-2.8088410000	-1.5803040000
H	-0.8722270000	0.6683070000	-1.9575090000
H	5.4072800000	1.3189480000	-2.7297680000
C	4.7341970000	1.1359120000	-1.9072180000
C	3.5672450000	0.4269840000	-1.8127500000
N	4.9581240000	1.6532570000	-0.6426620000
C	3.9343790000	1.2748900000	0.1591480000
N	3.0897780000	0.5174350000	-0.5205610000
H	5.6845550000	2.3064330000	-0.3894640000
H	3.0289700000	-0.1247320000	-2.5676700000
H	3.7976080000	1.5460820000	1.1945260000
H	4.5468630000	-4.2570970000	1.3493850000

C	3.7082870000	-3.5806910000	1.2941920000
C	3.4203850000	-2.5269960000	0.4655700000
N	2.6368560000	-3.6631970000	2.1659970000
C	1.7574650000	-2.6769810000	1.8411920000
N	2.2031560000	-1.9804920000	0.8153860000
H	2.5375230000	-4.3192990000	2.9266050000
H	4.0019330000	-2.1151640000	-0.3453920000
H	0.8339200000	-2.4855270000	2.3626490000
Fe	1.2322700000	0.0743740000	0.3048650000
O	-0.3909060000	-0.3929070000	1.1710690000
H	-0.2182740000	-0.0179280000	2.0577660000
F	1.8443200000	0.6531580000	1.9887740000
N	-2.4987240000	1.6184410000	0.8600610000
O	-4.5100030000	1.9988390000	-0.1745070000
C	-3.4923920000	-0.1125170000	-0.5464820000
C	-4.1148130000	-0.2110480000	-1.7934980000
C	-4.1730130000	-1.4167130000	-2.4929400000
C	-3.5913010000	-2.5604620000	-1.9313470000
C	-2.9815290000	-2.4895670000	-0.6885490000
C	-2.9338350000	-1.2827270000	0.0589120000
C	-3.5454000000	1.2683900000	0.0747870000
C	-2.3415140000	2.9430780000	1.5008890000
C	-3.5609780000	3.2440980000	2.3871830000
C	-1.0825960000	2.8563920000	2.3747640000
C	-2.1696750000	4.0259160000	0.4223280000
C	-2.4142610000	-1.2945360000	1.3948910000
C	-1.9786580000	-2.5494720000	2.0802320000
H	-4.5765090000	0.6873090000	-2.1891000000
H	-4.6808420000	-1.4685930000	-3.4525630000
H	-3.6246510000	-3.5094490000	-2.4604310000
H	-2.5569430000	-3.3901300000	-0.2598630000
H	-1.6589960000	1.0376980000	0.8137310000
H	-2.6703460000	-0.4533560000	2.0248290000
H	-1.5224300000	-2.3175400000	3.0472420000
H	-2.8314370000	-3.2199520000	2.2706440000
H	-1.2498470000	-3.1054090000	1.4816140000
H	-3.4129210000	4.2008190000	2.9009290000
H	-4.4728080000	3.2960580000	1.7910840000
H	-3.6817710000	2.4631920000	3.1475320000
H	-0.9082040000	3.8177950000	2.8687850000
H	-1.2043190000	2.0953860000	3.1564790000
H	-0.2004350000	2.6068520000	1.7800780000
H	-2.0127580000	5.0053200000	0.8890930000
H	-1.3037640000	3.7911720000	-0.2025690000
H	-3.0628400000	4.0748960000	-0.2055600000
N	0.5510440000	1.8948300000	-0.5011820000
N	1.3491680000	2.7973950000	-0.5882340000
N	2.1148550000	3.6647160000	-0.6767520000

$^{5}Im_3Wat_2$_FeF_remove-F
Charge: 2
Multiplicity: 5
B3LYP-D3(BJ)/6-31G(d)-SDD SCF energy (au): -955.134744862

B3LYP-D3(BJ)/6-31G(d)-SDD enthalpy (au): -954.841582862
B3LYP-D3(BJ)/6-31G(d)-SDD free energy (au): -954.921788862
B3LYP-D3(BJ)/def2-TZVP/SMD SCF energy (au): -2095.53168490
B3LYP-D3(BJ)/def2-TZVP/SMD enthalpy (au): -2095.23852290
B3LYP-D3(BJ)/def2-TZVP/SMD free energy (au): -2095.31872890
B3LYP-D3(BJ)/def2-TZVP/SMD free energy (quasi-harmonic) (au): -2095.310899038

Cartesian coordinates

ATOM	X	Y	Z
Fe	0.1290200000	-0.2784260000	-0.6121820000
C	1.0039420000	1.6378360000	3.0852060000
N	2.0058800000	2.1637540000	2.2935170000
H	2.7107960000	2.8267370000	2.5946750000
C	1.8856260000	1.6597000000	1.0506370000
H	2.5483160000	1.9018680000	0.2350590000
N	0.8441220000	0.8281140000	0.9955520000
C	0.2863690000	0.8081440000	2.2700160000
H	-0.5787680000	0.2059870000	2.4981490000
C	-3.6470800000	-1.8027650000	0.7800250000
N	-3.8706050000	-0.4452620000	0.8972140000
H	-4.7225720000	-0.0135020000	1.2357620000
C	-2.7682110000	0.2122820000	0.4826840000
H	-2.6740420000	1.2883820000	0.4762470000
N	-1.8342380000	-0.6609520000	0.1008190000
C	-2.3783170000	-1.9260770000	0.2843040000
H	-1.8203570000	-2.8191290000	0.0490930000
O	1.8697030000	0.1616840000	-1.9464040000
H	2.5376480000	-0.5384620000	-2.0380240000
H	2.2494930000	0.9712640000	-2.3268220000
H	-4.3914530000	-2.5348910000	1.0523800000
H	0.8963850000	1.8974600000	4.1269830000
C	1.4541840000	-4.3636370000	-0.7621400000
N	1.8810510000	-3.9876270000	0.4940010000
H	2.3530360000	-4.5862780000	1.1613230000
C	1.5713020000	-2.6875560000	0.6862870000
H	1.7906580000	-2.1388450000	1.5899930000
N	0.9621600000	-2.2017290000	-0.3938960000
C	0.8833610000	-3.2457120000	-1.3077140000
H	0.4249510000	-3.1226640000	-2.2775460000
H	1.5927000000	-5.3629880000	-1.1449390000
O	-0.8444530000	0.1645010000	-2.4456590000
H	-0.4015110000	0.2139660000	-3.3095160000
H	-1.7861840000	-0.0295830000	-2.5889860000

$^{6}Im_3Wat_2\_FeF$
Charge: 2
Multiplicity: 6
B3LYP-D3(BJ)/6-31G(d)-SDD SCF energy (au): -1054.98309009
B3LYP-D3(BJ)/6-31G(d)-SDD enthalpy (au): -1054.68541309
B3LYP-D3(BJ)/6-31G(d)-SDD free energy (au): -1054.76279009
B3LYP-D3(BJ)/def2-TZVP/SMD SCF energy (au): -2195.42360882
B3LYP-D3(BJ)/def2-TZVP/SMD enthalpy (au): -2195.12593182
B3LYP-D3(BJ)/def2-TZVP/SMD free energy (au): -2195.20330882

B3LYP-D3(BJ)/def2-TZVP/SMD free energy (quasi-harmonic) (au): -2195.19898573

Cartesian coordinates

ATOM	X	Y	Z
Fe	0.0964840000	-0.0998000000	-0.7458960000
C	1.2103150000	0.9276020000	3.2585740000
N	1.4917950000	2.0837160000	2.5543800000
H	1.8152070000	2.9569540000	2.9554150000
C	1.2541210000	1.8686300000	1.2486300000
H	1.3550300000	2.6132690000	0.4740400000
N	0.8414700000	0.6101820000	1.0714020000
C	0.8017620000	0.0157980000	2.3267560000
H	0.4656830000	-0.9994640000	2.4656430000
C	-3.6404820000	-1.2953720000	0.9285800000
N	-3.8413150000	0.0372660000	0.6173760000
H	-4.6932940000	0.5611010000	0.7837580000
C	-2.7264430000	0.5278420000	0.0497420000
H	-2.5965450000	1.5434950000	-0.2929720000
N	-1.8051720000	-0.4394580000	-0.0217430000
C	-2.3668630000	-1.5842540000	0.5276370000
H	-1.8237460000	-2.5139880000	0.5845900000
O	1.8772600000	0.1242620000	-2.0382480000
H	2.7267780000	-0.3399820000	-1.9507540000
H	2.0588020000	1.0388710000	-2.3198080000
H	-4.4044270000	-1.9008070000	1.3914690000
H	1.3188670000	0.8640190000	4.3303400000
C	1.1694930000	-4.2624490000	-0.5687450000
N	2.1127880000	-3.7181120000	0.2807390000
H	2.8370840000	-4.2340980000	0.7675100000
C	1.9145170000	-2.3886570000	0.3567560000
H	2.5085790000	-1.7194990000	0.9600170000
N	0.8762790000	-2.0427860000	-0.4104070000
C	0.4057960000	-3.2132300000	-0.9977820000
H	-0.4223800000	-3.2033510000	-1.6874660000
H	1.1312080000	-5.3183670000	-0.7883580000
O	-0.6471930000	-0.9212450000	-2.6374660000
H	0.0109180000	-0.7626070000	-3.3392530000
H	-1.4706940000	-0.4924740000	-2.9325990000
F	-0.2212120000	1.5735220000	-1.3398050000

$^{5}Im_3WatN_3$_11

Charge: 1
Multiplicity: 5
B3LYP-D3(BJ)/6-31G(d)-SDD SCF energy (au): -1779.29954894
B3LYP-D3(BJ)/6-31G(d)-SDD enthalpy (au): -1778.70854694
B3LYP-D3(BJ)/6-31G(d)-SDD free energy (au): -1778.82893794
B3LYP-D3(BJ)/def2-TZVP/SMD SCF energy (au): -2919.88077577
B3LYP-D3(BJ)/def2-TZVP/SMD enthalpy (au): -2919.28977377
B3LYP-D3(BJ)/def2-TZVP/SMD free energy (au): -2919.41016477
B3LYP-D3(BJ)/def2-TZVP/SMD free energy (quasi-harmonic) (au): -2919.39838683

Cartesian coordinates

ATOM	X	Y	Z

Fe	0.0460890000	-1.1547180000	-0.8650850000
C	1.6100680000	2.1758070000	1.5047020000
N	2.0626770000	2.4800640000	0.2341950000
H	2.7129120000	3.2154240000	-0.0052420000
C	1.5369100000	1.5800600000	-0.6319580000
H	1.7287090000	1.5684650000	-1.6925870000
N	0.7668350000	0.7173350000	0.0156160000
C	0.8051920000	1.0780500000	1.3512820000
H	0.2476970000	0.5292630000	2.0945230000
C	-2.9869900000	-1.6872550000	2.2410430000
N	-3.7787890000	-1.3352490000	1.1635930000
H	-4.7861650000	-1.2565890000	1.1656430000
C	-2.9751450000	-1.1300150000	0.0928760000
H	-3.3216950000	-0.8284560000	-0.8818580000
N	-1.7082470000	-1.3283580000	0.4282980000
C	-1.7029170000	-1.6802060000	1.7673230000
H	-0.7825420000	-1.9196800000	2.2752080000
H	-3.4010080000	-1.9102060000	3.2116620000
H	1.8955720000	2.7521080000	2.3706500000
C	2.0582560000	-4.2759750000	1.3745220000
N	2.9292840000	-3.2187860000	1.5651860000
H	3.8553150000	-3.2755630000	1.9652410000
C	2.3538970000	-2.0984350000	1.0657760000
H	2.8329900000	-1.1325990000	1.0474920000
N	1.1572350000	-2.3808810000	0.5687500000
C	0.9625860000	-3.7398490000	0.7544600000
H	0.0582300000	-4.2222750000	0.4166760000
H	2.2962760000	-5.2801460000	1.6879080000
O	-0.5648790000	-2.9660690000	-2.0926380000
H	0.3239520000	-2.9294970000	-2.5066140000
H	-1.1533170000	-2.5698000000	-2.7572880000
C	-5.3873280000	1.8088860000	-3.1552480000
C	-4.0840960000	1.3719190000	-3.3807690000
C	-3.0977710000	1.5642510000	-2.4059780000
C	-3.4050410000	2.1502990000	-1.1637590000
C	-4.7206520000	2.5888890000	-0.9660710000
C	-5.7004100000	2.4324040000	-1.9460130000
H	-6.1486720000	1.6646460000	-3.9150730000
H	-3.8258340000	0.8860530000	-4.3176960000
H	-4.9777460000	3.0560490000	-0.0188400000
H	-6.7105610000	2.7849180000	-1.7610220000
C	-2.3793820000	2.3038810000	-0.0603420000
C	-1.5146070000	3.5696220000	-0.1662610000
H	-1.7226880000	1.4313310000	-0.0375730000
H	-2.9098110000	2.3166450000	0.8989700000
H	-0.8608220000	3.6491020000	0.7078540000
H	-0.8780760000	3.5348610000	-1.0547610000
H	-2.1314990000	4.4730090000	-0.2190210000
C	-1.7357490000	0.9973300000	-2.6554030000
O	-1.4607450000	-0.1465540000	-2.2572250000
N	-0.8139410000	1.7830630000	-3.2672500000
C	-0.8441150000	2.9328110000	-4.2351930000
C	-1.9806020000	3.9139580000	-3.9170450000

C	-0.9965240000	2.3589890000	-5.6511850000
H	-1.8280590000	4.7915540000	-4.5523190000
H	-2.9664850000	3.5087960000	-4.1381220000
H	-1.9601030000	4.2432540000	-2.8767780000
H	-0.2025970000	1.6409630000	-5.8705970000
H	-1.9650150000	1.8614390000	-5.7651720000
H	-0.9418570000	3.1695210000	-6.3840580000
F	0.3622810000	1.0532410000	-3.4938080000
C	0.4994540000	3.6654450000	-4.0735960000
H	0.6226640000	4.0211810000	-3.0451610000
H	1.3445860000	3.0261410000	-4.3330720000
H	0.5103160000	4.5331430000	-4.7384330000
N	3.7715500000	-0.7022220000	-1.5002780000
N	2.7094040000	-0.9985550000	-1.8554670000
N	1.5921920000	-1.2845650000	-2.2189700000

$^{5}Im_{3}WatN_{3}$_13

Charge: 1
Multiplicity: 5
B3LYP-D3(BJ)/6-31G(d)-SDD SCF energy (au): -1779.36491787
B3LYP-D3(BJ)/6-31G(d)-SDD enthalpy (au): -1778.77521587
B3LYP-D3(BJ)/6-31G(d)-SDD free energy (au): -1778.89570387
B3LYP-D3(BJ)/def2-TZVP/SMD SCF energy (au): -2919.95371781
B3LYP-D3(BJ)/def2-TZVP/SMD enthalpy (au): -2919.36401581
B3LYP-D3(BJ)/def2-TZVP/SMD free energy (au): -2919.48450381
B3LYP-D3(BJ)/def2-TZVP/SMD free energy (quasi-harmonic) (au): -2919.47132394

Cartesian coordinates

ATOM	X	Y	Z
H	33.1276900000	26.2897170000	37.3348880000
C	33.3807290000	27.2461090000	36.9056760000
C	34.5820460000	27.8430770000	36.6370280000
N	32.4198240000	28.1511260000	36.4988330000
C	33.0411730000	29.2451220000	36.0014270000
N	34.3574740000	29.0909330000	36.0785400000
H	31.4195510000	28.0100980000	36.5242260000
H	35.5810740000	27.4707830000	36.7969860000
H	32.5227880000	30.0892090000	35.5716530000
H	39.8346410000	26.9499830000	36.4325760000
C	39.0639100000	27.5121750000	35.9294520000
C	38.1370380000	28.4105220000	36.3836850000
N	38.8501890000	27.4155610000	34.5668930000
C	37.8241290000	28.2324060000	34.2361340000
N	37.3684440000	28.8463600000	35.3189270000
H	39.3755590000	26.8413660000	33.9220210000
H	37.9666850000	28.7739890000	37.3844070000
H	37.4418400000	28.3749520000	33.2373420000
H	36.4614910000	31.2095160000	40.5411610000
C	36.5581150000	31.2166640000	39.4669830000
C	35.9039430000	30.5293670000	38.4802370000
N	37.4663310000	32.0293010000	38.8127350000
C	37.3439150000	31.8209880000	37.4811580000
N	36.4056080000	30.9127030000	37.2508120000

H	38.1027080000	32.6815410000	39.2496960000
H	35.1142800000	29.7999400000	38.5615930000
H	37.9162140000	32.3194620000	36.7145320000
Fe	35.8777290000	30.4170610000	35.1854270000
O	34.4720730000	32.0148840000	35.3363580000
H	34.9522030000	32.6998460000	34.8363670000
H	33.6013080000	31.8940090000	34.8463880000
F	37.0734060000	31.6928840000	34.6502500000
N	31.3040420000	31.3068090000	31.9964170000
O	32.1893290000	31.5809800000	34.0775460000
C	33.3840360000	32.5210940000	32.2365680000
C	33.6670380000	33.7492890000	32.8379840000
C	34.7098640000	34.5562750000	32.3811070000
C	35.4912170000	34.1168950000	31.3026000000
C	35.2293940000	32.9006620000	30.6968720000
C	34.1746570000	32.0474020000	31.1367610000
C	32.2529560000	31.7485140000	32.8351670000
C	30.0409100000	30.6062940000	32.3483190000
C	30.3466310000	29.2915490000	33.0806650000
C	29.1692240000	31.5296620000	33.2137140000
C	29.3440280000	30.3079070000	31.0146690000
C	33.9807190000	30.7771160000	30.5438240000
C	34.8240970000	30.2340380000	29.4355960000
H	33.0425930000	34.0828660000	33.6622300000
H	34.9023230000	35.5167430000	32.8480880000
H	36.3078650000	34.7331030000	30.9388190000
H	35.8563980000	32.5718950000	29.8759800000
H	31.4260880000	31.5580090000	31.0238770000
H	33.2409800000	30.1115760000	30.9723940000
H	35.8571610000	30.0542420000	29.7713400000
H	34.4258260000	29.2827250000	29.0738260000
H	34.8868900000	30.9201190000	28.5806370000
H	29.4125080000	28.7442430000	33.2459860000
H	31.0247020000	28.6627110000	32.4979710000
H	30.8128260000	29.4864890000	34.0476220000
H	29.6831780000	31.7857880000	34.1430300000
H	28.9341580000	32.4547320000	32.6771190000
H	28.2273530000	31.0286030000	33.4604890000
H	29.9584650000	29.6531410000	30.3861160000
H	28.3908010000	29.8041620000	31.1964100000
H	29.1335900000	31.2314340000	30.4623200000
N	35.3071900000	29.7654520000	33.4090700000
N	34.4424400000	28.9544320000	33.1667140000
N	33.6180570000	28.1934950000	32.8939020000

^{5}Im$_3$WatN$_3$_FeF_remove-F

Charge: 1
Multiplicity: 5
B3LYP-D3(BJ)/6-31G(d)-SDD SCF energy (au): -1043.21464517
B3LYP-D3(BJ)/6-31G(d)-SDD enthalpy (au): -1042.93275317
B3LYP-D3(BJ)/6-31G(d)-SDD free energy (au): -1043.01460517
B3LYP-D3(BJ)/def2-TZVP/SMD SCF energy (au): -2183.51101043
B3LYP-D3(BJ)/def2-TZVP/SMD enthalpy (au): -2183.22911843

B3LYP-D3(BJ)/def2-TZVP/SMD free energy (au): -2183.31097043
B3LYP-D3(BJ)/def2-TZVP/SMD free energy (quasi-harmonic) (au): -2183.30341626

Cartesian coordinates

ATOM	X	Y	Z
Fe	0.3578050000	-0.7767130000	-0.5548850000
C	2.1106280000	0.9118400000	3.0684070000
N	2.0077290000	2.0259530000	2.2578220000
H	2.3768470000	2.9452750000	2.4602430000
C	1.3721970000	1.6674210000	1.1184430000
H	1.1777530000	2.3394440000	0.2963420000
N	1.0553000000	0.3791280000	1.1563230000
C	1.5156510000	-0.1044660000	2.3704860000
H	1.3923280000	-1.1414820000	2.6434750000
C	-3.4255840000	-2.3238330000	0.7826200000
N	-3.1887310000	-1.3356610000	1.7196040000
H	-3.7930280000	-1.0965000000	2.4943000000
C	-2.0157670000	-0.7343190000	1.4212210000
H	-1.5790400000	0.0709930000	1.9911600000
N	-1.4867430000	-1.2856760000	0.3349130000
C	-2.3594970000	-2.2832130000	-0.0727770000
H	-2.1503670000	-2.8880300000	-0.9412870000
H	-4.3062930000	-2.9456760000	0.8120270000
H	2.5848880000	0.9466360000	4.0365530000
C	2.7274450000	-4.3783200000	-0.2426410000
N	3.6697090000	-3.3735180000	-0.1332090000
H	4.6711100000	-3.5015100000	-0.0796750000
C	3.0285690000	-2.1820220000	-0.1341560000
H	3.5267790000	-1.2243270000	-0.0865110000
N	1.7180780000	-2.3732970000	-0.2411510000
C	1.5185910000	-3.7408420000	-0.3122580000
H	0.5293580000	-4.1599830000	-0.4117180000
H	2.9981450000	-5.4220640000	-0.2614040000
O	-0.4236710000	-1.7886900000	-2.4758550000
H	0.3507990000	-1.4411450000	-2.9611020000
H	-1.1704340000	-1.2838230000	-2.8404400000
N	3.4565680000	1.1528430000	-1.4469140000
N	2.3943950000	0.7626210000	-1.6744640000
N	1.2715220000	0.3604960000	-1.9111140000

$^5Im_3WatN_3$_FeF_remove-N_3

Charge: 1
Multiplicity: 5
B3LYP-D3(BJ)/6-31G(d)-SDD SCF energy (au): -978.881546340
B3LYP-D3(BJ)/6-31G(d)-SDD enthalpy (au): -978.613679340
B3LYP-D3(BJ)/6-31G(d)-SDD free energy (au): -978.687327340
B3LYP-D3(BJ)/def2-TZVP/SMD SCF energy (au): -2119.15587470
B3LYP-D3(BJ)/def2-TZVP/SMD enthalpy (au): -2118.88800770
B3LYP-D3(BJ)/def2-TZVP/SMD free energy (au): -2118.96165570
B3LYP-D3(BJ)/def2-TZVP/SMD free energy (quasi-harmonic) (au): -2118.956150360

Cartesian coordinates

ATOM	X	Y	Z

Fe	0.2353100000	-0.5913820000	-0.4129880000
C	2.0425790000	1.1506760000	3.1944440000
N	1.9141050000	2.2172810000	2.3264200000
H	2.1626880000	3.1769550000	2.5238030000
C	1.3868370000	1.7620290000	1.1641320000
H	1.1545110000	2.3729960000	0.3048940000
N	1.1738040000	0.4555110000	1.2424890000
C	1.5758760000	0.0620750000	2.5062710000
H	1.4867330000	-0.9631360000	2.8333140000
C	-2.9961530000	-2.7628090000	1.4671040000
N	-3.4688760000	-1.4657470000	1.4434050000
H	-4.3796360000	-1.1598920000	1.7574110000
C	-2.5082050000	-0.6708700000	0.9152660000
H	-2.6103120000	0.3939460000	0.7679360000
N	-1.4431180000	-1.3944090000	0.5996150000
C	-1.7352920000	-2.7030320000	0.9376240000
H	-1.0248750000	-3.4990590000	0.7753530000
H	-3.5843350000	-3.5832810000	1.8463630000
H	2.4366680000	1.2636610000	4.1921090000
C	2.4713140000	-4.2244680000	-0.9268760000
N	3.3566430000	-3.4780380000	-0.1725430000
H	4.2770970000	-3.7729720000	0.1246110000
C	2.7900390000	-2.2783250000	0.0901300000
H	3.2588750000	-1.4945110000	0.6652860000
N	1.5838840000	-2.2163050000	-0.4600040000
C	1.3726210000	-3.4278560000	-1.0995800000
H	0.4544110000	-3.6267670000	-1.6309290000
H	2.7011010000	-5.2235070000	-1.2621430000
O	-0.6320700000	-1.2364800000	-2.4825160000
H	0.0374100000	-1.3857240000	-3.1701810000
H	-0.7976260000	-0.2677980000	-2.5101750000
F	-0.1744980000	1.0127420000	-1.2610050000

$^{6}Im_3WatN_3$_FeF
Charge: 1
Multiplicity: 6
B3LYP-D3(BJ)/6-31G(d)-SDD SCF energy (au): -1143.07370529
B3LYP-D3(BJ)/6-31G(d)-SDD enthalpy (au): -1142.78776629
B3LYP-D3(BJ)/6-31G(d)-SDD free energy (au): -1142.86892629
B3LYP-D3(BJ)/def2-TZVP/SMD SCF energy (au): -2283.41282535
B3LYP-D3(BJ)/def2-TZVP/SMD enthalpy (au): -2283.12688635
B3LYP-D3(BJ)/def2-TZVP/SMD free energy (au): -2283.20804635
B3LYP-D3(BJ)/def2-TZVP/SMD free energy (quasi-harmonic) (au): -2283.20235306

Cartesian coordinates

ATOM	X	Y	Z
Fe	0.3290050000	-0.3119630000	-0.7178980000
C	1.5482200000	1.0664080000	3.2063450000
N	2.0335510000	2.0443290000	2.3571740000
H	2.5032910000	2.8951220000	2.6372630000
C	1.7659700000	1.6805340000	1.0847230000
H	2.0188970000	2.2588920000	0.2090400000
N	1.1330440000	0.5128880000	1.0761460000

C	0.9857780000	0.1194250000	2.3951820000
H	0.4847070000	-0.8001340000	2.6539250000
C	-2.4246610000	-2.7064160000	1.7400900000
N	-3.1192080000	-1.5386110000	1.4832760000
H	-4.0480330000	-1.3145910000	1.8143310000
C	-2.3540470000	-0.7480300000	0.6986650000
H	-2.6323710000	0.2293600000	0.3367920000
N	-1.1986590000	-1.3510380000	0.4389190000
C	-1.2311090000	-2.5755440000	1.0839390000
H	-0.4087940000	-3.2690580000	1.0163040000
H	-2.8366380000	-3.5012240000	2.3416770000
H	1.6445590000	1.1359960000	4.2783220000
C	2.3109660000	-4.1931670000	-0.9103930000
N	3.1207160000	-3.5248830000	-0.0116710000
H	3.9897160000	-3.8685900000	0.3742860000
C	2.5887080000	-2.3033260000	0.2167120000
H	3.0259070000	-1.5640810000	0.8686460000
N	1.4702700000	-2.1577850000	-0.4834060000
C	1.2864320000	-3.3326370000	-1.1958200000
H	0.4408650000	-3.4580180000	-1.8531920000
H	2.5340880000	-5.1906530000	-1.2545370000
O	-0.4569890000	-1.2242110000	-2.5994920000
H	0.1967650000	-0.8713600000	-3.2320510000
H	-1.2401490000	-0.6531480000	-2.7088450000
F	-0.9432870000	0.9570200000	-1.0554140000
N	4.0781960000	-0.1889670000	-1.6234650000
N	2.9667150000	0.0979140000	-1.7158390000
N	1.7983470000	0.4196880000	-1.8119130000

$^5Im_3WatN_3$_TS-1
Charge: 1
Multiplicity: 5
B3LYP-D3(BJ)/6-31G(d)-SDD SCF energy (au): -1779.27826697
B3LYP-D3(BJ)/6-31G(d)-SDD enthalpy (au): -1778.69037497
B3LYP-D3(BJ)/6-31G(d)-SDD free energy (au): -1778.80857397
B3LYP-D3(BJ)/def2-TZVP/SMD SCF energy (au): -2919.86033980
B3LYP-D3(BJ)/def2-TZVP/SMD enthalpy (au): -2919.27244780
B3LYP-D3(BJ)/def2-TZVP/SMD free energy (au): -2919.39064680
B3LYP-D3(BJ)/def2-TZVP/SMD free energy (quasi-harmonic) (au): -2919.37915083

Cartesian coordinates

ATOM	X	Y	Z
Fe	0.3118910000	-0.4076850000	-0.8764230000
C	1.5588250000	0.9756170000	3.1414590000
N	2.2915340000	1.7847110000	2.2945640000
H	2.9617040000	2.4875750000	2.5741310000
C	1.9635900000	1.4700160000	1.0172120000
H	2.3854100000	1.9418210000	0.1436830000
N	1.0588660000	0.5036020000	1.0016280000
C	0.7952220000	0.1863080000	2.3220170000
H	0.0795160000	-0.5778840000	2.5825600000
C	-2.5999300000	-2.5009630000	1.7116860000
N	-3.2296950000	-1.2891290000	1.4976400000

H	-4.1283110000	-1.0136700000	1.8692990000
C	-2.4460700000	-0.5392990000	0.6855070000
H	-2.6884990000	0.4557090000	0.3512760000
N	-1.3460670000	-1.2056370000	0.3703400000
C	-1.4287790000	-2.4333720000	1.0043970000
H	-0.6412070000	-3.1638070000	0.9044270000
H	-3.0332880000	-3.2742660000	2.3261920000
H	1.6440290000	1.0390780000	4.2149170000
C	2.9120410000	-3.8192870000	-1.1696270000
N	3.2298190000	-3.4022370000	0.1079850000
H	4.0307220000	-3.7023380000	0.6459400000
C	2.3475250000	-2.4431030000	0.4738120000
H	2.3802930000	-1.9182980000	1.4159840000
N	1.4626940000	-2.2401920000	-0.4932190000
C	1.8120070000	-3.0911660000	-1.5302650000
H	1.2708240000	-3.0864060000	-2.4637460000
H	3.4881060000	-4.5674220000	-1.6904820000
O	-0.7526810000	-1.4058690000	-2.4731460000
H	-1.5285260000	-0.7933370000	-2.6102030000
H	-1.1486020000	-2.2194720000	-2.1198660000
C	-5.8153100000	2.6216730000	-4.5465880000
C	-4.7138020000	1.9023240000	-4.1056380000
C	-4.0896050000	2.2314830000	-2.8877490000
C	-4.6028200000	3.2550180000	-2.0612330000
C	-5.7263950000	3.9514760000	-2.5257790000
C	-6.3160590000	3.6593760000	-3.7532430000
H	-6.2847300000	2.3764990000	-5.4937420000
H	-4.3088440000	1.0886580000	-4.6987590000
H	-6.1536440000	4.7300890000	-1.8993930000
H	-7.1797110000	4.2280110000	-4.0845600000
C	-4.0743960000	3.5789400000	-0.6769430000
C	-3.3062670000	4.9060210000	-0.5971130000
H	-3.4298640000	2.7749920000	-0.3121580000
H	-4.9361500000	3.6281030000	0.0008350000
H	-3.0559510000	5.1415130000	0.4425350000
H	-2.3743360000	4.8465710000	-1.1651200000
H	-3.8999700000	5.7345130000	-0.9976620000
C	-2.8831970000	1.4353810000	-2.5449690000
O	-2.8795880000	0.2042270000	-2.5846280000
N	-1.8150370000	2.2461540000	-2.1691560000
C	-0.8529470000	2.7596430000	-3.1754100000
C	-1.6187270000	3.8476480000	-3.9764550000
C	-0.3589940000	1.6679250000	-4.1355000000
H	-0.8921820000	4.3267480000	-4.6393620000
H	-2.4155170000	3.4214900000	-4.5881500000
H	-2.0471670000	4.6075580000	-3.3204390000
H	0.2286480000	0.9108060000	-3.6146520000
H	-1.1921010000	1.1905050000	-4.6621580000
H	0.2906690000	2.1277460000	-4.8868820000
F	-0.8909120000	1.2269600000	-1.1043460000
C	0.3098040000	3.4187020000	-2.4226940000
H	-0.0655540000	4.1158390000	-1.6674280000
H	0.9353880000	2.6621980000	-1.9477420000

H	0.9244350000	3.9747780000	-3.1370740000
N	4.0705440000	-0.3383140000	-1.4850870000
N	2.9835150000	-0.0030440000	-1.6814800000
N	1.8460170000	0.3693010000	-1.8807360000

^{5}Im$_3$WatN$_3$_TS-3-F

Charge: 1
Multiplicity: 5
B3LYP-D3(BJ)/6-31G(d)-SDD SCF energy (au): -1779.35280166
B3LYP-D3(BJ)/6-31G(d)-SDD enthalpy (au): -1778.76423366
B3LYP-D3(BJ)/6-31G(d)-SDD free energy (au): -1778.88297966
B3LYP-D3(BJ)/def2-TZVP/SMD SCF energy (au): -2919.94294440
B3LYP-D3(BJ)/def2-TZVP/SMD enthalpy (au): -2919.35437640
B3LYP-D3(BJ)/def2-TZVP/SMD free energy (au): -2919.47312240
B3LYP-D3(BJ)/def2-TZVP/SMD free energy (quasi-harmonic) (au): -2919.46087774

Cartesian coordinates

ATOM	X	Y	Z
H	32.9776240000	26.2852680000	37.5967610000
C	33.2479890000	27.2595430000	37.2216580000
C	34.4338620000	27.7777000000	36.7770440000
N	32.3314080000	28.2865970000	37.1100520000
C	32.9654410000	29.3691470000	36.6024160000
N	34.2468800000	29.0958560000	36.3973700000
H	31.3425730000	28.2247540000	37.3075640000
H	35.3996210000	27.3044660000	36.7007940000
H	32.4903240000	30.3146570000	36.3961960000
H	39.4340380000	26.6222620000	35.9365790000
C	38.6337480000	27.2185080000	35.5273680000
C	37.9473610000	28.2984390000	36.0152520000
N	38.0909760000	26.9682740000	34.2803470000
C	37.1124140000	27.8773510000	34.0514380000
N	37.0032030000	28.6950990000	35.0870570000
H	38.3712600000	26.2322670000	33.6472380000
H	38.0549430000	28.8103730000	36.9586160000
H	36.5036600000	27.9291680000	33.1615400000
H	37.4165060000	31.4194760000	40.1198340000
C	37.2479390000	31.4021420000	39.0545900000
C	36.3042760000	30.7809800000	38.2804740000
N	38.0524390000	32.0986170000	38.1696630000
C	37.5871730000	31.8858030000	36.9146040000
N	36.5282100000	31.0901590000	36.9522160000
H	38.8474190000	32.6716200000	38.4162450000
H	35.4870470000	30.1396180000	38.5701590000
H	38.0167930000	32.2925770000	36.0124810000
Fe	35.5628480000	30.3356070000	35.1212270000
O	34.1051960000	31.9031970000	35.5088250000
H	34.5952050000	32.7199020000	35.6947550000
H	33.5355950000	32.1011530000	34.7013180000
F	36.7446350000	31.4915230000	34.1780550000
N	33.3388470000	32.6632610000	31.1432390000
O	32.7115790000	32.3645500000	33.3209020000
C	34.5749330000	33.7993640000	32.8638450000

C	34.1894900000	34.9835440000	33.4900340000
C	35.1370550000	35.9568540000	33.8161590000
C	36.4873560000	35.7383460000	33.5130610000
C	36.8883470000	34.5477620000	32.9264230000
C	35.9521000000	33.5316600000	32.6056880000
C	33.4711100000	32.8581610000	32.4657100000
C	32.2548120000	31.9126460000	30.4508460000
C	32.2303960000	30.4581010000	30.9347330000
C	30.9091060000	32.6035370000	30.7204390000
C	32.5985890000	31.9697840000	28.9573280000
C	36.3654800000	32.2340770000	32.1844990000
C	37.7209940000	31.8783100000	31.6891030000
H	33.1367220000	35.1522940000	33.6960490000
H	34.8233410000	36.8837160000	34.2857350000
H	37.2255440000	36.4989280000	33.7488010000
H	37.9415320000	34.3771150000	32.7346370000
H	33.9581000000	33.2039760000	30.5537300000
H	35.5921380000	31.4865630000	32.0426890000
H	38.5191380000	32.4236790000	32.1981550000
H	37.8953170000	30.8079560000	31.8218080000
H	37.7999900000	32.0987970000	30.6130580000
H	31.5054500000	29.8900750000	30.3428870000
H	33.2145010000	29.9940850000	30.8159370000
H	31.9422610000	30.3948690000	31.9834680000
H	30.6870390000	32.6062640000	31.7901340000
H	30.9218200000	33.6373260000	30.3582280000
H	30.1070030000	32.0686120000	30.2017260000
H	33.5609150000	31.4851990000	28.7551940000
H	31.8308380000	31.4479810000	28.3798300000
H	32.6413530000	33.0048210000	28.5960990000
N	34.6577910000	29.6504580000	33.4484250000
N	33.6473940000	28.9800170000	33.5055120000
N	32.6811900000	28.3491280000	33.5387610000

$^{5}Im_3WatN_3$_TS-3-N_3

Charge: 1
Multiplicity: 5
B3LYP-D3(BJ)/6-31G(d)-SDD SCF energy (au): -1779.36187779
B3LYP-D3(BJ)/6-31G(d)-SDD enthalpy (au): -1778.77304579
B3LYP-D3(BJ)/6-31G(d)-SDD free energy (au): -1778.89114779
B3LYP-D3(BJ)/def2-TZVP/SMD SCF energy (au): -2919.95008180
B3LYP-D3(BJ)/def2-TZVP/SMD enthalpy (au): -2919.36124980
B3LYP-D3(BJ)/def2-TZVP/SMD free energy (au): -2919.47935180
B3LYP-D3(BJ)/def2-TZVP/SMD free energy (quasi-harmonic) (au): -2919.46759701

Cartesian coordinates

ATOM	X	Y	Z
H	32.8400230000	27.0610100000	38.2516600000
C	33.1280790000	27.8623910000	37.5900080000
C	34.3399720000	28.2459540000	37.0832270000
N	32.2119260000	28.7552200000	37.0690950000
C	32.8713880000	29.6337930000	36.2775980000
N	34.1682670000	29.3540090000	36.2714400000

H	31.2136450000	28.7409830000	37.2236780000
H	35.3126600000	27.8056660000	37.2329260000
H	32.3874170000	30.4171910000	35.7137220000
H	39.2839600000	26.4261650000	36.3124050000
C	38.5385970000	27.0217870000	35.8094230000
C	37.8209880000	28.1206280000	36.1992190000
N	38.1248420000	26.7494450000	34.5189950000
C	37.1880820000	27.6644320000	34.1715800000
N	36.9800180000	28.5066490000	35.1712760000
H	38.4647940000	26.0009270000	33.9314840000
H	37.8485090000	28.6605010000	37.1325470000
H	36.6965440000	27.7076720000	33.2123600000
H	37.3982120000	31.8465080000	39.9101660000
C	37.2928070000	31.6518710000	38.8545360000
C	36.3522770000	30.9648460000	38.1338340000
N	38.1959450000	32.1297540000	37.9223150000
C	37.7894760000	31.7305860000	36.6925120000
N	36.6764670000	31.0195040000	36.7923440000
H	39.0114460000	32.6926070000	38.1195730000
H	35.4702640000	30.4470580000	38.4762270000
H	38.2886900000	31.9578010000	35.7632200000
Fe	35.7011430000	30.2849110000	34.9691120000
O	34.5165260000	32.0946210000	35.1377510000
H	35.0287550000	32.6905260000	34.5636460000
H	33.6125930000	32.0387640000	34.7224330000
F	36.9352580000	31.2336210000	33.9747570000
N	31.2866370000	31.5123130000	31.8652840000
O	32.1131480000	31.7387700000	33.9820470000
C	33.4552410000	32.5446350000	32.1910900000
C	33.6669830000	33.8823370000	32.5181810000
C	34.8539830000	34.5253870000	32.1555100000
C	35.8512260000	33.8056540000	31.4855870000
C	35.6602140000	32.4689810000	31.1729870000
C	34.4554210000	31.7946740000	31.5011290000
C	32.2240720000	31.8893600000	32.7468410000
C	29.9729610000	30.8797380000	32.1697770000
C	30.1968430000	29.5369370000	32.8789830000
C	29.1354080000	31.8324180000	33.0365150000
C	29.2912740000	30.6521590000	30.8151710000
C	34.2797190000	30.4131820000	31.2136990000
C	35.2880630000	29.5779020000	30.4992860000
H	32.9000510000	34.4223970000	33.0660630000
H	35.0004000000	35.5726670000	32.3995270000
H	36.7832950000	34.2923500000	31.2162440000
H	36.4518760000	31.9207540000	30.6767230000
H	31.4643540000	31.7557960000	30.8992750000
H	33.2899320000	29.9921970000	31.3460730000
H	36.2837050000	29.6907000000	30.9409180000
H	35.0099480000	28.5216400000	30.5378480000
H	35.3619540000	29.8623330000	29.4383640000
H	29.2310410000	29.0549220000	33.0624970000
H	30.8045540000	28.8630920000	32.2678530000
H	30.7039330000	29.6755220000	33.8350940000

H	29.6342180000	32.0308450000	33.9876300000
H	28.9735340000	32.7842980000	32.5199170000
H	28.1577690000	31.3838370000	33.2405370000
H	29.8850110000	29.9839820000	30.1805380000
H	28.3118790000	30.1899120000	30.9649760000
H	29.1364640000	31.5989240000	30.2842120000
N	34.7754610000	29.4051090000	33.3851790000
N	33.9028070000	28.5643010000	33.4703230000
N	33.0731200000	27.7634030000	33.5117990000

^{5}Im$_3$WatOH_11

Charge: 1

Multiplicity: 5

B3LYP-D3(BJ)/6-31G(d)-SDD SCF energy (au): -1690.91838365

B3LYP-D3(BJ)/6-31G(d)-SDD enthalpy (au): -1690.32872265

B3LYP-D3(BJ)/6-31G(d)-SDD free energy (au): -1690.44564165

B3LYP-D3(BJ)/def2-TZVP/SMD SCF energy (au): -2831.47273599

B3LYP-D3(BJ)/def2-TZVP/SMD enthalpy (au): -2830.88307499

B3LYP-D3(BJ)/def2-TZVP/SMD free energy (au): -2830.99999399

B3LYP-D3(BJ)/def2-TZVP/SMD free energy (quasi-harmonic) (au): -2830.98779634

Cartesian coordinates

ATOM	X	Y	Z
Fe	0.0196270000	-0.4964380000	-0.5244470000
C	-0.0367070000	1.7868010000	3.3159830000
N	0.8731290000	2.4292590000	2.4969210000
H	1.3731470000	3.2793390000	2.7164550000
C	0.9588260000	1.7381750000	1.3311120000
H	1.5514440000	2.0212950000	0.4738340000
N	0.1561870000	0.6846030000	1.3649920000
C	-0.4729800000	0.7052500000	2.5959240000
H	-1.1979230000	-0.0464370000	2.8695150000
C	-3.2512380000	-2.5073610000	1.5168650000
N	-3.9329520000	-1.8809930000	0.4907890000
H	-4.9327120000	-1.8989800000	0.3438960000
C	-3.0340930000	-1.2477420000	-0.3013790000
H	-3.2725010000	-0.6925290000	-1.1990430000
N	-1.8071620000	-1.4343390000	0.1701380000
C	-1.9295980000	-2.2211620000	1.3027240000
H	-1.0623890000	-2.5301470000	1.8662810000
O	0.6232780000	1.0121640000	-1.4756850000
H	0.8799260000	0.7856830000	-2.3817960000
H	-3.7548630000	-3.0869770000	2.2742840000
H	-0.2814770000	2.1505860000	4.3015180000
C	3.0866300000	-3.4917850000	0.0372520000
N	3.0319790000	-2.9411470000	1.3047030000
H	3.6259150000	-3.1879100000	2.0840580000
C	2.0581050000	-1.9991660000	1.3159950000
H	1.8042870000	-1.3949320000	2.1736870000
N	1.4825570000	-1.9233440000	0.1245900000
C	2.1175910000	-2.8489130000	-0.6845310000
H	1.8359020000	-2.9606040000	-1.7199790000
H	3.7944830000	-4.2625620000	-0.2236520000

O	-0.2539310000	-1.7209550000	-2.4326460000
H	-0.8968220000	-1.0990400000	-2.8630760000
H	-0.7897020000	-2.4986020000	-2.2080490000
C	-5.5189670000	3.0020550000	-3.5032460000
C	-4.3750300000	2.2835230000	-3.8397450000
C	-3.3708140000	2.0746840000	-2.8868490000
C	-3.4893790000	2.5673980000	-1.5757710000
C	-4.6520660000	3.2836630000	-1.2620430000
C	-5.6538060000	3.5022100000	-2.2068290000
H	-6.2958950000	3.1685370000	-4.2426760000
H	-4.2534020000	1.8821230000	-4.8419340000
H	-4.7738570000	3.6841360000	-0.2602640000
H	-6.5407050000	4.0648140000	-1.9307740000
C	-2.3999010000	2.3481460000	-0.5427610000
C	-2.0201580000	3.6089830000	0.2453010000
H	-1.4960420000	1.9555720000	-1.0194600000
H	-2.7255450000	1.5750440000	0.1673700000
H	-1.2153530000	3.3780060000	0.9486980000
H	-1.6693650000	4.4020220000	-0.4243880000
H	-2.8599230000	4.0092150000	0.8221670000
C	-2.2283490000	1.1871270000	-3.2664080000
O	-2.2543320000	-0.0146880000	-2.9476440000
N	-1.2346580000	1.7073450000	-4.0138650000
C	-0.7392320000	3.0371480000	-4.4835770000
C	-1.5451680000	4.1863890000	-3.8668710000
C	-0.8600340000	3.0546030000	-6.0141060000
H	-1.0842980000	5.1153470000	-4.2156980000
H	-2.5894240000	4.1898460000	-4.1778550000
H	-1.4985320000	4.1736950000	-2.7760530000
H	-0.3242890000	2.2103720000	-6.4557880000
H	-1.9098000000	3.0043470000	-6.3205360000
H	-0.4287080000	3.9801340000	-6.4073240000
F	-0.3098040000	0.7189040000	-4.3820370000
C	0.7277580000	3.1643800000	-4.0329200000
H	0.8108850000	3.0057350000	-2.9542680000
H	1.3676820000	2.4424430000	-4.5444500000
H	1.0843310000	4.1682100000	-4.2809280000

^{5}Im$_3$WatOH_FeF_remove-F

Charge: 1
Multiplicity: 5
B3LYP-D3(BJ)/6-31G(d)-SDD SCF energy (au): -954.840129310
B3LYP-D3(BJ)/6-31G(d)-SDD enthalpy (au): -954.559932310
B3LYP-D3(BJ)/6-31G(d)-SDD free energy (au): -954.635646310
B3LYP-D3(BJ)/def2-TZVP/SMD SCF energy (au): -2095.10486805
B3LYP-D3(BJ)/def2-TZVP/SMD enthalpy (au): -2094.82467105
B3LYP-D3(BJ)/def2-TZVP/SMD free energy (au): -2094.90038505
B3LYP-D3(BJ)/def2-TZVP/SMD free energy (quasi-harmonic) (au): -2094.894789740

Cartesian coordinates

ATOM	X	Y	Z
Fe	0.0511950000	-0.2493030000	-0.5206620000
C	1.6383310000	0.7355820000	3.4676790000

N	2.1222220000	1.7381070000	2.6508510000
H	2.6967610000	2.5168640000	2.9432080000
C	1.6908900000	1.5048880000	1.3869820000
H	1.9167570000	2.1228270000	0.5305590000
N	0.9528220000	0.4039310000	1.3523700000
C	0.9134540000	-0.0871370000	2.6459580000
H	0.3736440000	-0.9899170000	2.8905610000
C	-3.9056140000	-0.3519560000	1.1870620000
N	-4.1354600000	-0.6791940000	-0.1367670000
H	-5.0390540000	-0.8457450000	-0.5582460000
C	-2.9474060000	-0.7242240000	-0.7806040000
H	-2.8142940000	-0.9380290000	-1.8306810000
N	-1.9627870000	-0.4439880000	0.0666650000
C	-2.5501040000	-0.2079410000	1.2982100000
H	-1.9583750000	0.0569150000	2.1605860000
O	0.4658120000	1.2712540000	-1.5806080000
H	-0.0277320000	2.0875760000	-1.4121280000
H	-4.7064050000	-0.2527990000	1.9026110000
H	1.8461740000	0.7035450000	4.5256780000
C	1.3861590000	-4.3260810000	-0.8737860000
N	2.3336930000	-3.8092330000	-0.0105260000
H	3.1223830000	-4.3116670000	0.3736250000
C	2.0343510000	-2.5110040000	0.2245420000
H	2.6065480000	-1.8609120000	0.8680850000
N	0.9428650000	-2.1672650000	-0.4469840000
C	0.5280260000	-3.2940320000	-1.1372140000
H	-0.3514230000	-3.2739220000	-1.7615290000
H	1.4113030000	-5.3508350000	-1.2090500000
O	-0.6729020000	-0.7277610000	-2.8054580000
H	-0.3088880000	0.2001390000	-2.7355250000
H	-0.0276830000	-1.2046270000	-3.3513410000

^{5}Im$_3$WatOH_FeF_remove-OH
Charge: 1
Multiplicity: 5
B3LYP-D3(BJ)/6-31G(d)-SDD SCF energy (au): -978.885373045
B3LYP-D3(BJ)/6-31G(d)-SDD enthalpy (au): -978.616364045
B3LYP-D3(BJ)/6-31G(d)-SDD free energy (au): -978.691752045
B3LYP-D3(BJ)/def2-TZVP/SMD SCF energy (au): -2119.15781167
B3LYP-D3(BJ)/def2-TZVP/SMD enthalpy (au): -2118.88880267
B3LYP-D3(BJ)/def2-TZVP/SMD free energy (au): -2118.96419067
B3LYP-D3(BJ)/def2-TZVP/SMD free energy (quasi-harmonic) (au): -2118.958198625

Cartesian coordinates

ATOM	X	Y	Z
Fe	-0.0869750000	-0.2235080000	-0.5234440000
C	1.4761480000	0.7775120000	3.4645860000
N	1.6527930000	1.9255050000	2.7179240000
H	2.0377230000	2.7983150000	3.0525260000
C	1.2213220000	1.6869710000	1.4558770000
H	1.2363540000	2.4008310000	0.6459280000
N	0.7765920000	0.4416400000	1.3554320000
C	0.9307860000	-0.1376120000	2.6029350000

H	0.6424660000	-1.1614550000	2.7902640000
C	-3.9462160000	-0.5917510000	1.3302410000
N	-4.2537120000	-0.6948020000	-0.0141150000
H	-5.1818010000	-0.7823030000	-0.4052530000
C	-3.1045080000	-0.6379690000	-0.7230240000
H	-3.0362890000	-0.6816010000	-1.7994510000
N	-2.0708990000	-0.5061220000	0.1016540000
C	-2.5850950000	-0.4743870000	1.3869870000
H	-1.9422570000	-0.3569850000	2.2453040000
H	-4.7047210000	-0.6078670000	2.0966860000
H	1.7464120000	0.7204190000	4.5072480000
C	1.3330640000	-4.2628440000	-0.9572650000
N	2.3494160000	-3.6865800000	-0.2193680000
H	3.2001970000	-4.1455620000	0.0770200000
C	2.0192920000	-2.3988480000	0.0310700000
H	2.6318430000	-1.7100720000	0.5925100000
N	0.8410610000	-2.1183100000	-0.5116650000
C	0.4021330000	-3.2758050000	-1.1322990000
H	-0.5462440000	-3.3120720000	-1.6461510000
H	1.3663860000	-5.2922700000	-1.2772010000
O	-0.8254950000	-0.4412240000	-2.7626970000
H	-0.2200790000	-0.9409750000	-3.3336450000
H	-0.4404300000	0.4668750000	-2.7199700000
F	0.4172390000	1.3433150000	-1.3921640000

$^{6}Im_3WatOH\_FeF$

Charge: 1
Multiplicity: 6
B3LYP-D3(BJ)/6-31G(d)-SDD SCF energy (au): -1054.69822175
B3LYP-D3(BJ)/6-31G(d)-SDD enthalpy (au): -1054.41368875
B3LYP-D3(BJ)/6-31G(d)-SDD free energy (au): -1054.49054475
B3LYP-D3(BJ)/def2-TZVP/SMD SCF energy (au): -2195.00741246
B3LYP-D3(BJ)/def2-TZVP/SMD enthalpy (au): -2194.72287946
B3LYP-D3(BJ)/def2-TZVP/SMD free energy (au): -2194.79973546
B3LYP-D3(BJ)/def2-TZVP/SMD free energy (quasi-harmonic) (au): -2194.79487071

Cartesian coordinates

ATOM	X	Y	Z
Fe	0.1804000000	0.0181660000	-0.7926600000
C	0.7325220000	1.4418660000	3.2800900000
N	1.9339540000	1.7394060000	2.6628430000
H	2.7109570000	2.2317900000	3.0822440000
C	1.8876320000	1.2815630000	1.3928180000
H	2.6783910000	1.3742370000	0.6649610000
N	0.7150090000	0.7000140000	1.1660070000
C	-0.0168340000	0.7958330000	2.3361200000
H	-1.0148930000	0.3964650000	2.4100640000
C	-3.4783380000	-1.4858480000	1.2072720000
N	-3.9041840000	-0.3764190000	0.5004190000
H	-4.8453190000	-0.0074630000	0.4837180000
C	-2.8479030000	0.1322250000	-0.1743610000
H	-2.8727620000	1.0132340000	-0.7963180000
N	-1.7648110000	-0.5976590000	0.0602930000

C	-2.1449250000	-1.6103630000	0.9240400000
H	-1.4384600000	-2.3456820000	1.2752890000
O	1.8929380000	0.3715800000	-1.4568400000
H	1.9195430000	1.2400790000	-1.8932880000
H	-4.1451960000	-2.0667010000	1.8247780000
H	0.5288930000	1.7126900000	4.3039760000
C	1.0204660000	-4.2686340000	-0.6399520000
N	2.2133150000	-3.6995220000	-0.2310370000
H	3.0695800000	-4.1973010000	-0.0286550000
C	2.0482900000	-2.3588360000	-0.1718280000
H	2.8253240000	-1.6551680000	0.0812850000
N	0.8075600000	-2.0406190000	-0.5125770000
C	0.1552190000	-3.2234390000	-0.8142120000
H	-0.8716830000	-3.2274710000	-1.1431470000
H	0.9052990000	-5.3331010000	-0.7693570000
O	-0.5115570000	-0.8093480000	-2.7678910000
H	0.3309110000	-0.9548430000	-3.2336980000
H	-0.8445330000	0.0480460000	-3.0941860000
F	-0.7058170000	1.4933430000	-1.4498580000

$^{5}Im_{3}$WatOH_TS-1

Charge: 1
Multiplicity: 5
B3LYP-D3(BJ)/6-31G(d)-SDD SCF energy (au): -1690.89186837
B3LYP-D3(BJ)/6-31G(d)-SDD enthalpy (au): -1690.30575237
B3LYP-D3(BJ)/6-31G(d)-SDD free energy (au): -1690.42189937
B3LYP-D3(BJ)/def2-TZVP/SMD SCF energy (au): -2831.44546097
B3LYP-D3(BJ)/def2-TZVP/SMD enthalpy (au): -2830.85934497
B3LYP-D3(BJ)/def2-TZVP/SMD free energy (au): -2830.97549197
B3LYP-D3(BJ)/def2-TZVP/SMD free energy (quasi-harmonic) (au): -2830.96342160

Cartesian coordinates

ATOM	X	Y	Z
Fe	0.3748020000	-0.6192920000	-0.9983780000
C	2.1921940000	1.8331830000	2.2649330000
N	2.9841950000	2.0577460000	1.1545780000
H	3.7647720000	2.6970100000	1.1014590000
C	2.5263580000	1.2793970000	0.1403610000
H	2.9331010000	1.2403510000	-0.8595980000
N	1.4837840000	0.5705090000	0.5457190000
C	1.2637330000	0.9063940000	1.8668940000
H	0.4600280000	0.4603770000	2.4331250000
C	-2.3305160000	-1.8775510000	2.2031530000
N	-2.8010650000	-0.6165850000	1.8854710000
H	-3.5737610000	-0.1418860000	2.3315420000
C	-2.0619930000	-0.1290630000	0.8602520000
H	-2.1953690000	0.8389800000	0.4053320000
N	-1.1387900000	-1.0135760000	0.5081930000
C	-1.2940390000	-2.1087000000	1.3386540000
H	-0.6536530000	-2.9719630000	1.2496370000
O	1.6152840000	-0.0893160000	-2.2852460000
H	1.7372800000	-0.7884660000	-2.9469880000
H	-2.7668980000	-2.4742820000	2.9885220000

H	2.3598310000	2.3384450000	3.2031690000
C	2.2418230000	-4.4789340000	-0.6958030000
N	2.8493270000	-3.8062680000	0.3487900000
H	3.5914030000	-4.1655200000	0.9331680000
C	2.2975010000	-2.5717520000	0.4380970000
H	2.5879790000	-1.8208950000	1.1562370000
N	1.3646240000	-2.4244910000	-0.4897450000
C	1.3209990000	-3.6054310000	-1.2086780000
H	0.6466620000	-3.7214420000	-2.0429180000
H	2.5189380000	-5.4844670000	-0.9703790000
O	-0.9336400000	-1.7531280000	-2.4214500000
H	-1.4344840000	-0.9884480000	-2.8055120000
H	-1.6087540000	-2.2486850000	-1.9305930000
C	-5.3670740000	2.9109510000	-5.1200120000
C	-4.2757970000	2.1923390000	-4.6489030000
C	-3.9805400000	2.1705960000	-3.2754150000
C	-4.8146030000	2.8206170000	-2.3444410000
C	-5.9164400000	3.5259830000	-2.8461270000
C	-6.1838400000	3.5908490000	-4.2114550000
H	-5.5856320000	2.9362240000	-6.1827490000
H	-3.6326550000	1.6490320000	-5.3340210000
H	-6.5846460000	4.0197180000	-2.1452030000
H	-7.0419110000	4.1533560000	-4.5667780000
C	-4.6314540000	2.7269770000	-0.8422530000
C	-4.0219920000	3.9831990000	-0.2036750000
H	-4.0172180000	1.8558540000	-0.5941940000
H	-5.6187210000	2.5467560000	-0.3987950000
H	-4.0229540000	3.9022810000	0.8892080000
H	-2.9896600000	4.1213640000	-0.5345350000
H	-4.5920350000	4.8784670000	-0.4735010000
C	-2.7564720000	1.4081840000	-2.8794600000
O	-2.6076950000	0.2302070000	-3.1858390000
N	-1.8890170000	2.1849760000	-2.1181510000
C	-0.9056660000	3.1043720000	-2.7470440000
C	-1.6991850000	4.2918110000	-3.3506850000
C	-0.0954100000	2.3976500000	-3.8433240000
H	-0.9667040000	5.0338380000	-3.6827780000
H	-2.2974700000	3.9898970000	-4.2109940000
H	-2.3563670000	4.7587460000	-2.6138380000
H	0.4868440000	1.5707480000	-3.4272100000
H	-0.7519850000	2.0162450000	-4.6333560000
H	0.5904140000	3.1189390000	-4.3002540000
F	-0.7902360000	0.9632170000	-1.3761280000
C	0.0053100000	3.6375850000	-1.6324060000
H	-0.5869460000	4.0919540000	-0.8315920000
H	0.6104390000	2.8348610000	-1.2118110000
H	0.6685740000	4.4027010000	-2.0478290000

N_3□
Charge: 0
Multiplicity: 2
B3LYP-D3(BJ)/6-31G(d)-SDD SCF energy (au): -164.135645456
B3LYP-D3(BJ)/6-31G(d)-SDD enthalpy (au): -164.123282456

B3LYP-D3(BJ)/6-31G(d)-SDD free energy (au): -164.145918456
B3LYP-D3(BJ)/def2-TZVP/SMD SCF energy (au): -164.204699031
B3LYP-D3(BJ)/def2-TZVP/SMD enthalpy (au): -164.192336031
B3LYP-D3(BJ)/def2-TZVP/SMD free energy (au): -164.214972031
B3LYP-D3(BJ)/def2-TZVP/SMD free energy (quasi-harmonic) (au): -164.214972031

Cartesian coordinates

ATOM	X	Y	Z
N	4.0850900000	-0.1948240000	-1.6230990000
N	2.9482470000	0.1105640000	-1.7169730000
N	1.8099210000	0.4128950000	-1.8111450000

N-centered radical
Charge: 0
Multiplicity: 2
B3LYP-D3(BJ)/6-31G(d)-SDD SCF energy (au): -636.215951469
B3LYP-D3(BJ)/6-31G(d)-SDD enthalpy (au): -635.915028469
B3LYP-D3(BJ)/6-31G(d)-SDD free energy (au): -635.974910469
B3LYP-D3(BJ)/def2-TZVP/SMD SCF energy (au): -636.478583618
B3LYP-D3(BJ)/def2-TZVP/SMD enthalpy (au): -636.177660618
B3LYP-D3(BJ)/def2-TZVP/SMD free energy (au): -636.237542618
B3LYP-D3(BJ)/def2-TZVP/SMD free energy (quasi-harmonic) (au): -636.235282149

Cartesian coordinates

ATOM	X	Y	Z
C	-3.4987470000	-3.4231720000	-5.9770130000
C	-2.7455640000	-2.2869970000	-5.7113140000
C	-3.2356070000	-1.2673920000	-4.8785970000
C	-4.5273010000	-1.3755720000	-4.3183830000
C	-5.2720290000	-2.5272760000	-4.6074520000
C	-4.7723250000	-3.5438690000	-5.4173450000
H	-3.1003650000	-4.2057460000	-6.6158920000
H	-1.7571300000	-2.1579640000	-6.1381000000
H	-6.2709650000	-2.6181760000	-4.1875660000
H	-5.3789150000	-4.4226330000	-5.6184690000
C	-5.1574150000	-0.3388280000	-3.4133790000
C	-4.8709110000	-0.6010130000	-1.9261290000
H	-6.2418050000	-0.3484730000	-3.5804520000
H	-4.8138550000	0.6651130000	-3.6794000000
H	-5.3532730000	0.1581480000	-1.2999200000
H	-5.2513670000	-1.5838850000	-1.6267410000
H	-3.7952010000	-0.5710750000	-1.7361790000
C	-2.3158450000	-0.1045980000	-4.6881210000
O	-1.2980900000	0.0376500000	-5.3802270000
N	-2.5216870000	0.7360210000	-3.6197140000
C	-2.3252810000	2.1794290000	-3.7112960000
C	-2.7598510000	2.7692710000	-5.0634520000
C	-0.8161750000	2.4391230000	-3.4654520000
H	-2.6080370000	3.8539410000	-5.0662180000
H	-2.1823780000	2.3353340000	-5.8824420000
H	-3.8241540000	2.5770550000	-5.2430490000
H	-0.5015130000	1.9901610000	-2.5186490000
H	-0.2257250000	2.0079560000	-4.2748460000

H	-0.6455130000	3.5207910000	-3.4164470000
C	-3.1350730000	2.8060080000	-2.5616460000
H	-4.2095770000	2.6539510000	-2.7078120000
H	-2.8521140000	2.3580280000	-1.6046380000
H	-2.9462440000	3.8838070000	-2.5176360000

OH□
Charge: 0
Multiplicity: 2
B3LYP-D3(BJ)/6-31G(d)-SDD SCF energy (au): -75.7217884823
B3LYP-D3(BJ)/6-31G(d)-SDD enthalpy (au): -75.7102224823
B3LYP-D3(BJ)/6-31G(d)-SDD free energy (au): -75.7304694823
B3LYP-D3(BJ)/def2-TZVP/SMD SCF energy (au): -75.7734433827
B3LYP-D3(BJ)/def2-TZVP/SMD enthalpy (au): -75.7618773827
B3LYP-D3(BJ)/def2-TZVP/SMD free energy (au): -75.7821243827
B3LYP-D3(BJ)/def2-TZVP/SMD free energy (quasi-harmonic) (au): -75.7821239004

Cartesian coordinates

ATOM	X	Y	Z
O	-0.4595260000	-1.2255800000	-2.5970370000
H	0.1993020000	-0.8699910000	-3.2345060000

^{5}TS-1'
Charge: 1
Multiplicity: 5
B3LYP-D3(BJ)/6-31G(d)-SDD SCF energy (au): -1693.77688517
B3LYP-D3(BJ)/6-31G(d)-SDD enthalpy (au): -1693.19952117
B3LYP-D3(BJ)/6-31G(d)-SDD free energy (au): -1693.31489017
B3LYP-D3(BJ)/def2-TZVP/SMD SCF energy (au): -2834.33807910
B3LYP-D3(BJ)/def2-TZVP/SMD enthalpy (au): -2833.76071510
B3LYP-D3(BJ)/def2-TZVP/SMD free energy (au): -2833.87608410
B3LYP-D3(BJ)/def2-TZVP/SMD free energy (quasi-harmonic) (au): -2833.86476393

Cartesian coordinates

ATOM	X	Y	Z
Fe	0.0380170000	-0.6593910000	-0.8120440000
C	-0.8973410000	-1.7926800000	3.3080600000
N	-1.4780580000	-0.5417560000	3.2073140000
H	-1.9369190000	-0.0388540000	3.9544800000
C	-1.3224110000	-0.0996510000	1.9385680000
H	-1.6756800000	0.8499310000	1.5700770000
N	-0.6694020000	-1.0032160000	1.2184470000
C	-0.3971780000	-2.0649130000	2.0642200000
H	0.1321080000	-2.9367710000	1.7146140000
H	-0.8966960000	-2.3541580000	4.2288830000
C	1.9448700000	-4.5886280000	-1.0212690000
N	2.9345220000	-3.8207140000	-0.4377740000
H	3.8668540000	-4.1328410000	-0.2027430000
C	2.4519200000	-2.5680910000	-0.2573410000
H	3.0090130000	-1.7546150000	0.1801840000
N	1.2014910000	-2.4965450000	-0.6902910000
C	0.8716670000	-3.7515480000	-1.1728490000
H	-0.1033700000	-3.9472950000	-1.5915530000

H	2.0938290000	-5.6276010000	-1.2695950000
O	-1.6133130000	-1.8123750000	-1.5531760000
H	-2.0931680000	-1.0938230000	-2.0689000000
H	-2.1882650000	-2.0123400000	-0.7957600000
C	-3.7360990000	3.4139350000	-5.7544710000
C	-3.0110970000	2.5910940000	-4.8983520000
C	-3.5194250000	2.2841570000	-3.6291820000
C	-4.7799320000	2.7474070000	-3.2106190000
C	-5.4866040000	3.5752360000	-4.0917640000
C	-4.9709140000	3.9204360000	-5.3396480000
H	-3.3459350000	3.6522810000	-6.7387600000
H	-2.0571790000	2.1785850000	-5.2113390000
H	-6.4657610000	3.9414880000	-3.7943910000
H	-5.5417070000	4.5671880000	-5.9989410000
C	-5.4004920000	2.3502750000	-1.8866960000
C	-5.2219110000	3.3858610000	-0.7653060000
H	-4.9978180000	1.3852300000	-1.5606590000
H	-6.4720260000	2.1911090000	-2.0545540000
H	-5.8089760000	3.1013480000	0.1143680000
H	-4.1728610000	3.4557490000	-0.4644210000
H	-5.5544770000	4.3786700000	-1.0867810000
C	-2.7184410000	1.3844210000	-2.7290260000
O	-2.7985420000	0.1608360000	-2.8306820000
N	-2.0762620000	2.0839530000	-1.7469240000
C	-1.1115890000	3.2043430000	-1.8221020000
C	-1.8931160000	4.4942330000	-2.1899500000
C	-0.0016390000	2.9472590000	-2.8466410000
H	-1.1878510000	5.3275230000	-2.1122290000
H	-2.2826860000	4.4644090000	-3.2061740000
H	-2.7172270000	4.6717670000	-1.4972620000
H	0.5512810000	2.0369640000	-2.6203280000
H	-0.4086780000	2.8657730000	-3.8577540000
H	0.7034120000	3.7845470000	-2.8361110000
F	-1.2892960000	0.8328620000	-0.7558350000
C	-0.5219490000	3.4068160000	-0.4125310000
H	-1.3201890000	3.4881080000	0.3317990000
H	0.1433480000	2.5879110000	-0.1380420000
H	0.0478610000	4.3404720000	-0.4069190000
C	2.5737640000	0.8880660000	-1.0463880000
O	2.6953170000	0.6437160000	-2.2626320000
O	1.5919900000	0.4647110000	-0.3076640000
C	3.5936610000	1.7505030000	-0.3370940000
H	3.1332620000	2.7161720000	-0.0974270000
H	3.8889390000	1.2927310000	0.6117740000
H	4.4656680000	1.9167680000	-0.9706240000
O	0.4742390000	-0.4788880000	-2.8945180000
H	0.5640780000	-1.3450710000	-3.3237120000
H	1.4119750000	-0.0812360000	-2.8135270000

^{3}TS-1'

Charge: 1

Multiplicity: 3

B3LYP-D3(BJ)/6-31G(d)-SDD SCF energy (au): -1693.75025590

B3LYP-D3(BJ)/6-31G(d)-SDD enthalpy (au): -1693.17163990
B3LYP-D3(BJ)/6-31G(d)-SDD free energy (au): -1693.28401490
B3LYP-D3(BJ)/def2-TZVP/SMD SCF energy (au): -2834.31268992
B3LYP-D3(BJ)/def2-TZVP/SMD enthalpy (au): -2833.73407392
B3LYP-D3(BJ)/def2-TZVP/SMD free energy (au): -2833.84644892
B3LYP-D3(BJ)/def2-TZVP/SMD free energy (quasi-harmonic) (au): -2833.83569902

Cartesian coordinates

ATOM	X	Y	Z
Fe	0.3716990000	-0.6737230000	-0.9888760000
C	-2.2649440000	-1.9603890000	2.0349940000
N	-2.5001390000	-0.6003050000	1.9699500000
H	-3.1494840000	-0.0824170000	2.5455210000
C	-1.7085720000	-0.0711640000	1.0084620000
H	-1.6790460000	0.9700040000	0.7310530000
N	-0.9760330000	-1.0291580000	0.4560760000
C	-1.3113190000	-2.2143710000	1.0865150000
H	-0.8372110000	-3.1455920000	0.8199100000
H	-2.7803520000	-2.6022940000	2.7315690000
C	2.2833480000	-4.5840100000	-0.7194940000
N	2.7933360000	-3.9194420000	0.3794380000
H	3.4745530000	-4.2859390000	1.0302530000
C	2.2405100000	-2.6828730000	0.4259730000
H	2.4551880000	-1.9345710000	1.1732740000
N	1.4000830000	-2.5256370000	-0.5858200000
C	1.4176680000	-3.7029940000	-1.3122210000
H	0.8146570000	-3.8212410000	-2.1996410000
H	2.5783040000	-5.5904160000	-0.9717230000
O	-0.8060030000	-1.6298120000	-2.3830200000
H	-1.2738620000	-0.8743970000	-2.8449110000
H	-1.5207360000	-2.1122680000	-1.9347860000
C	-5.3703060000	2.8998160000	-5.0052250000
C	-4.2403630000	2.1955800000	-4.6107050000
C	-3.8774810000	2.1427320000	-3.2539320000
C	-4.6803600000	2.7415350000	-2.2615980000
C	-5.8222400000	3.4320900000	-2.6878460000
C	-6.1570130000	3.5308260000	-4.0363710000
H	-5.6417850000	2.9524560000	-6.0545810000
H	-3.6174610000	1.6912000000	-5.3426890000
H	-6.4683480000	3.8862610000	-1.9411090000
H	-7.0447060000	4.0816990000	-4.3320490000
C	-4.4250710000	2.5996010000	-0.7732000000
C	-3.8446650000	3.8582880000	-0.1124530000
H	-3.7625920000	1.7494510000	-0.5828800000
H	-5.3838400000	2.3593160000	-0.2967270000
H	-3.7975010000	3.7378190000	0.9757940000
H	-2.8336630000	4.0549140000	-0.4781020000
H	-4.4626520000	4.7372090000	-0.3239130000
C	-2.6193880000	1.4124100000	-2.9333520000
O	-2.4327330000	0.2523380000	-3.2924490000
N	-1.7489910000	2.1934030000	-2.1751980000
C	-0.8092530000	3.1450420000	-2.8288730000
C	-1.6539100000	4.3475410000	-3.3217800000

C	-0.0763720000	2.4947520000	-4.0096670000
H	-0.9541920000	5.0991590000	-3.6993870000
H	-2.3323950000	4.0726270000	-4.1299290000
H	-2.2350640000	4.7914330000	-2.5107660000
H	0.4754980000	1.6089710000	-3.6895180000
H	-0.7757510000	2.2083200000	-4.8026610000
H	0.6334270000	3.2129310000	-4.4319570000
F	-0.6935920000	1.0003840000	-1.4568230000
C	0.1738370000	3.6402950000	-1.7591480000
H	-0.3634270000	3.9647860000	-0.8624470000
H	0.8902190000	2.8653860000	-1.4887400000
H	0.7306640000	4.4946460000	-2.1553370000
C	2.3711800000	1.1409520000	0.1079440000
O	2.8392590000	1.3866230000	-1.0259120000
O	1.4352610000	0.2819210000	0.3564540000
C	2.8886930000	1.9014080000	1.3089570000
H	2.1762680000	2.6996400000	1.5495240000
H	2.9569690000	1.2478720000	2.1823950000
H	3.8569350000	2.3523950000	1.0871740000
O	1.5839200000	-0.1990700000	-2.5166850000
H	2.1310590000	-0.9588900000	-2.7741300000
H	2.2008910000	0.4867820000	-2.0410930000

^{5}TS-1

Charge: 2
Multiplicity: 5
B3LYP-D3(BJ)/6-31G(d)-SDD SCF energy (au): -1691.22827201
B3LYP-D3(BJ)/6-31G(d)-SDD enthalpy (au): -1690.62899401
B3LYP-D3(BJ)/6-31G(d)-SDD free energy (au): -1690.74560901
B3LYP-D3(BJ)/def2-TZVP/SMD SCF energy (au): -2831.89376700
B3LYP-D3(BJ)/def2-TZVP/SMD enthalpy (au): -2831.29448900
B3LYP-D3(BJ)/def2-TZVP/SMD free energy (au): -2831.41110400
B3LYP-D3(BJ)/def2-TZVP/SMD free energy (quasi-harmonic) (au): -2831.39943799

Cartesian coordinates

ATOM	X	Y	Z
Fe	0.3653060000	-0.3631130000	-0.7281460000
C	1.1320730000	1.3526830000	3.1568150000
N	2.1041450000	1.9540980000	2.3807180000
H	2.7876600000	2.6271670000	2.7045120000
C	1.9892980000	1.4996740000	1.1150750000
H	2.6260290000	1.7940040000	0.2957600000
N	0.9850150000	0.6308650000	1.0370620000
C	0.4401240000	0.5316980000	2.3091650000
H	-0.4007900000	-0.1120230000	2.5125430000
C	-3.0231650000	-2.4910060000	0.8748660000
N	-3.5335500000	-1.2087300000	0.8186960000
H	-4.4811940000	-0.9392520000	1.0506640000
C	-2.5616820000	-0.3766030000	0.3868880000
H	-2.6930180000	0.6849500000	0.2492350000
N	-1.4431730000	-1.0596910000	0.1656450000
C	-1.7212900000	-2.3852520000	0.4680380000
H	-0.9693450000	-3.1531810000	0.3750130000

O	2.2607130000	0.2999700000	-1.7173040000
H	2.8424330000	-0.4309390000	-1.9865930000
H	2.0432350000	0.7978810000	-2.5231880000
H	-3.6165110000	-3.3340670000	1.1927330000
H	1.0245140000	1.5614710000	4.2097650000
C	2.7447980000	-4.0196890000	-0.5899790000
N	2.7415810000	-3.6448910000	0.7373210000
H	3.2125870000	-4.1314180000	1.4900490000
C	2.0112610000	-2.5135250000	0.8641360000
H	1.8515350000	-1.9961480000	1.7979480000
N	1.5379250000	-2.1410320000	-0.3206610000
C	1.9935660000	-3.0769110000	-1.2394720000
H	1.7503010000	-3.0065990000	-2.2889870000
H	3.2651740000	-4.8971690000	-0.9413320000
O	-0.2273290000	-1.2678110000	-2.5808560000
H	-1.0007880000	-0.6800630000	-2.8313250000
H	-0.6014870000	-2.1607050000	-2.4945610000
C	-6.0296960000	2.2351680000	-3.7045310000
C	-4.7731640000	1.6619940000	-3.6094490000
C	-3.8790040000	2.0619960000	-2.5901110000
C	-4.2593030000	3.0295230000	-1.6241360000
C	-5.5441980000	3.5747760000	-1.7417510000
C	-6.4113990000	3.2049090000	-2.7671610000
H	-6.7106720000	1.9341520000	-4.4935210000
H	-4.4532430000	0.9065980000	-4.3194650000
H	-5.8729070000	4.3005010000	-1.0032580000
H	-7.3938220000	3.6624120000	-2.8309160000
C	-3.4169930000	3.4520850000	-0.4356320000
C	-2.8818050000	4.8885050000	-0.5439510000
H	-2.5704520000	2.7760080000	-0.2932890000
H	-4.0477180000	3.3802860000	0.4597480000
H	-2.3611710000	5.1726320000	0.3757810000
H	-2.1775470000	4.9781560000	-1.3757230000
H	-3.6922740000	5.6053880000	-0.7071750000
C	-2.5534780000	1.4107210000	-2.6372300000
O	-2.4019360000	0.2036040000	-2.8364740000
N	-1.5324470000	2.3414400000	-2.4486050000
C	-0.8222550000	2.9216660000	-3.5962530000
C	-1.8530990000	3.8722010000	-4.2840130000
C	-0.3563950000	1.8670290000	-4.6129210000
H	-1.3111640000	4.4119810000	-5.0659860000
H	-2.6710180000	3.3143340000	-4.7428360000
H	-2.2653400000	4.5954220000	-3.5788080000
H	0.3402500000	1.1609010000	-4.1540760000
H	-1.1942210000	1.3000700000	-5.0274900000
H	0.1534390000	2.3695090000	-5.4395860000
F	-0.1863890000	1.2863110000	-1.6808400000
C	0.3430280000	3.7629420000	-3.0597360000
H	-0.0117110000	4.4915410000	-2.3259610000
H	1.0923060000	3.1307150000	-2.5792700000
H	0.8151920000	4.3016910000	-3.8857640000

^{3}TS-1

Charge: 2
Multiplicity: 3
B3LYP-D3(BJ)/6-31G(d)-SDD SCF energy (au): -1691.20095744
B3LYP-D3(BJ)/6-31G(d)-SDD enthalpy (au): -1690.60052244
B3LYP-D3(BJ)/6-31G(d)-SDD free energy (au): -1690.71334744
B3LYP-D3(BJ)/def2-TZVP/SMD SCF energy (au): -2831.86896007
B3LYP-D3(BJ)/def2-TZVP/SMD enthalpy (au): -2831.26852507
B3LYP-D3(BJ)/def2-TZVP/SMD free energy (au): -2831.38135007
B3LYP-D3(BJ)/def2-TZVP/SMD free energy (quasi-harmonic) (au): -2831.37042963

Cartesian coordinates

ATOM	X	Y	Z
Fe	0.3726390000	-0.4260220000	-0.6901880000
C	1.5961460000	1.1984960000	2.9627970000
N	2.2619490000	1.9986320000	2.0548450000
H	2.8641640000	2.7795540000	2.2840380000
C	1.9742760000	1.5729270000	0.8075100000
H	2.3579860000	2.0154900000	-0.0970900000
N	1.1509920000	0.5291150000	0.8698020000
C	0.9027200000	0.2879330000	2.2139600000
H	0.2485780000	-0.5069080000	2.5343680000
C	-2.6166150000	-2.3720990000	1.4875150000
N	-3.1466090000	-1.1085550000	1.3241090000
H	-4.0520460000	-0.8002760000	1.6549950000
C	-2.2657040000	-0.3528100000	0.6332340000
H	-2.4180260000	0.6781180000	0.3595210000
N	-1.1873500000	-1.0760840000	0.3482840000
C	-1.3919580000	-2.3423020000	0.8766280000
H	-0.6542620000	-3.1219830000	0.7761980000
O	1.9532040000	0.2606320000	-1.8496260000
H	2.5826350000	-0.4793620000	-1.9260300000
H	1.6451830000	0.4489180000	-2.7543970000
H	-3.1439430000	-3.1573430000	2.0062660000
H	1.6716670000	1.3486700000	4.0284110000
C	2.5490950000	-4.2350050000	-0.7439630000
N	2.9354450000	-3.7241250000	0.4774690000
H	3.5591060000	-4.1745460000	1.1359250000
C	2.3522090000	-2.5145680000	0.6424440000
H	2.4848510000	-1.8932780000	1.5149890000
N	1.5999730000	-2.2212730000	-0.4141020000
C	1.7181050000	-3.2928910000	-1.2890610000
H	1.2188310000	-3.3036010000	-2.2459050000
H	2.8936810000	-5.1914900000	-1.1055130000
O	-0.4509460000	-1.2530330000	-2.3616640000
H	-1.2217230000	-0.6338340000	-2.5760750000
H	-0.8743530000	-2.1067760000	-2.1675560000
C	-5.8274920000	2.4521340000	-4.4184280000
C	-4.6626010000	1.7978800000	-4.0520700000
C	-4.0041010000	2.1366200000	-2.8495280000
C	-4.5419580000	3.1081350000	-1.9699030000
C	-5.7283230000	3.7383850000	-2.3648060000
C	-6.3544550000	3.4365050000	-3.5721440000
H	-6.3278270000	2.1997780000	-5.3473070000

H	-4.2352270000	1.0290200000	-4.6873800000
H	-6.1784000000	4.4701820000	-1.6999280000
H	-7.2676550000	3.9546970000	-3.8477610000
C	-3.9816600000	3.4292470000	-0.5978620000
C	-3.3342600000	4.8178460000	-0.4922760000
H	-3.2519850000	2.6737120000	-0.2932390000
H	-4.8131350000	3.3738000000	0.1157100000
H	-3.0675640000	5.0374110000	0.5462850000
H	-2.4233540000	4.8690210000	-1.0952120000
H	-4.0151790000	5.6024960000	-0.8364600000
C	-2.7293350000	1.4319280000	-2.6108810000
O	-2.5926700000	0.2141580000	-2.7463130000
N	-1.7164010000	2.3236950000	-2.2422100000
C	-0.8540700000	2.9337290000	-3.2767860000
C	-1.7133450000	4.0300540000	-3.9710380000
C	-0.3858840000	1.9198900000	-4.3311820000
H	-1.0549270000	4.5606760000	-4.6652250000
H	-2.5423040000	3.6003240000	-4.5344810000
H	-2.1080570000	4.7453290000	-3.2483110000
H	0.1366760000	1.0768790000	-3.8694000000
H	-1.2224940000	1.5120590000	-4.9056610000
H	0.2960930000	2.4099010000	-5.0314930000
F	-0.5894300000	1.2236090000	-1.3409820000
C	0.3237470000	3.6134250000	-2.5651370000
H	-0.0379040000	4.2731870000	-1.7718340000
H	0.9937550000	2.8741390000	-2.1225830000
H	0.8871710000	4.2116370000	-3.2864640000

^{5}TS-2'

Charge: 1
Multiplicity: 5
B3LYP-D3(BJ)/6-31G(d)-SDD SCF energy (au): -1693.81375146
B3LYP-D3(BJ)/6-31G(d)-SDD enthalpy (au): -1693.24029146
B3LYP-D3(BJ)/6-31G(d)-SDD free energy (au): -1693.35562146
B3LYP-D3(BJ)/def2-TZVP/SMD SCF energy (au): -2834.37665990
B3LYP-D3(BJ)/def2-TZVP/SMD enthalpy (au): -2833.80319990
B3LYP-D3(BJ)/def2-TZVP/SMD free energy (au): -2833.91852990
B3LYP-D3(BJ)/def2-TZVP/SMD free energy (quasi-harmonic) (au): -2833.90629244

Cartesian coordinates

ATOM	X	Y	Z
Fe	-0.1453580000	-0.1141520000	-0.1035820000
C	-2.8354440000	-3.1324240000	1.5263070000
N	-3.2083120000	-1.9679860000	2.1744760000
H	-3.9113530000	-1.8897220000	2.8966770000
C	-2.4674570000	-0.9492730000	1.6872330000
H	-2.5322950000	0.0798600000	2.0040740000
N	-1.6360870000	-1.4048080000	0.7566440000
C	-1.8540370000	-2.7664730000	0.6472450000
H	-1.2970250000	-3.3691420000	-0.0517440000
H	-3.2927420000	-4.0843190000	1.7450320000
C	1.2785020000	-3.4689130000	-2.4663060000
N	2.1406740000	-3.4727480000	-1.3840520000

H	2.9299630000	-4.0910380000	-1.2559370000
C	1.7665270000	-2.4894400000	-0.5353420000
H	2.2586000000	-2.2494280000	0.3938250000
N	0.7037440000	-1.8606910000	-1.0185670000
C	0.3886680000	-2.4578730000	-2.2259800000
H	-0.4359280000	-2.1003300000	-2.8210980000
H	1.3819420000	-4.1618960000	-3.2861340000
O	-1.4189720000	0.0004600000	-1.8856450000
H	-1.2152550000	0.9025220000	-2.2765740000
H	-2.3124690000	0.0902890000	-1.5132670000
F	-1.1127310000	1.2995900000	0.5478490000
C	-1.3261250000	3.4714080000	-2.5637790000
O	-0.6261730000	2.4188970000	-2.6092500000
N	-0.8199000000	4.6749330000	-2.2776560000
H	-3.1667890000	5.4968250000	-0.7424570000
C	0.3669090000	5.0081840000	-1.4705490000
C	0.3908570000	6.5397710000	-1.3387840000
C	0.2827470000	4.3558020000	-0.0815930000
C	1.6261240000	4.5627030000	-2.2470460000
H	0.4227420000	7.0167290000	-2.3231980000
H	-0.4935110000	6.9017580000	-0.8026250000
H	1.2748410000	6.8477270000	-0.7731460000
H	0.1025780000	3.2824530000	-0.1397810000
H	1.2138090000	4.5164500000	0.4706240000
H	-0.5374420000	4.7973450000	0.4954390000
H	2.5117850000	4.9161710000	-1.7092250000
H	1.6897000000	3.4790920000	-2.3429630000
H	1.6295890000	4.9975140000	-3.2505920000
C	-2.7728670000	3.4215450000	-2.9321830000
C	-3.2483290000	2.3147830000	-3.6470970000
C	-3.6475430000	4.4776190000	-2.5977040000
C	-4.5847060000	2.2456000000	-4.0297010000
H	-2.5555510000	1.5322370000	-3.9361620000
C	-4.9849840000	4.4002430000	-3.0022500000
C	-5.4534710000	3.2898380000	-3.7031790000
H	-4.9439340000	1.3929310000	-4.5973890000
H	-5.6664880000	5.2080820000	-2.7557980000
H	-6.4963520000	3.2438740000	-4.0020910000
C	-3.1187000000	5.6479670000	-1.8278000000
C	-3.5471950000	7.0347110000	-2.2593000000
H	-4.6120530000	7.1983490000	-2.0437820000
H	-2.9871790000	7.8033310000	-1.7180050000
H	-3.3970350000	7.1802590000	-3.3332210000
H	-1.9109500000	5.5064410000	-2.0423210000
C	1.9660950000	0.7274170000	1.7309870000
O	2.3418140000	1.6578180000	1.0067380000
O	1.1664110000	-0.2348430000	1.3254450000
C	2.4071460000	0.6191640000	3.1704370000
H	1.5411290000	0.7852500000	3.8211830000
H	2.7799750000	-0.3884970000	3.3775480000
H	3.1758910000	1.3608010000	3.3900080000
O	1.1714370000	1.0345420000	-1.2223640000
H	1.7260300000	1.4397040000	-0.4786620000

H 0.6900830000 1.7513320000 -1.7041810000

³TS-2'

Charge: 1
Multiplicity: 3
B3LYP-D3(BJ)/6-31G(d)-SDD SCF energy (au): -1693.78985435
B3LYP-D3(BJ)/6-31G(d)-SDD enthalpy (au): -1693.21719735
B3LYP-D3(BJ)/6-31G(d)-SDD free energy (au): -1693.33048435
B3LYP-D3(BJ)/def2-TZVP/SMD SCF energy (au): -2834.35668777
B3LYP-D3(BJ)/def2-TZVP/SMD enthalpy (au): -2833.78403077
B3LYP-D3(BJ)/def2-TZVP/SMD free energy (au): -2833.89731777
B3LYP-D3(BJ)/def2-TZVP/SMD free energy (quasi-harmonic) (au): -2833.88591142

Cartesian coordinates

ATOM	X	Y	Z
Fe	-0.2685590000	-0.1846270000	-0.0116610000
C	-2.8721200000	-3.0376350000	1.5242460000
N	-2.2511550000	-2.5377310000	2.6558320000
H	-2.4433260000	-2.8142820000	3.6090020000
C	-1.3439020000	-1.6115250000	2.2816460000
H	-0.6922140000	-1.0554050000	2.9361680000
N	-1.3606580000	-1.4983540000	0.9603220000
C	-2.3060930000	-2.3790610000	0.4685190000
H	-2.4911780000	-2.4743920000	-0.5894720000
H	-3.6365110000	-3.7965540000	1.5738180000
C	1.2383330000	-3.2315800000	-2.4941040000
N	2.1049530000	-3.2630100000	-1.4160780000
H	2.9168090000	-3.8581340000	-1.3244580000
C	1.7002090000	-2.3487070000	-0.5084670000
H	2.1815310000	-2.1320500000	0.4320930000
N	0.6114530000	-1.7403520000	-0.9587680000
C	0.3082750000	-2.2740160000	-2.1972140000
H	-0.5374550000	-1.9191220000	-2.7632810000
H	1.3632520000	-3.8727960000	-3.3520230000
O	-1.8059950000	-0.0941700000	-1.7113370000
H	-1.5446920000	0.7740400000	-2.1249390000
H	-2.6006840000	0.1351400000	-1.2002520000
F	-1.2070850000	1.1445570000	0.6851780000
C	-1.4600000000	3.4106140000	-2.5411630000
O	-0.8567790000	2.3022450000	-2.4506460000
N	-0.8787510000	4.5827600000	-2.2687840000
H	-3.3000300000	5.7472560000	-1.0969550000
C	0.2501250000	4.8700830000	-1.3669660000
C	0.3584270000	6.4012900000	-1.2734040000
C	0.0067740000	4.2722220000	0.0280670000
C	1.5407990000	4.3290960000	-2.0215100000
H	0.4968520000	6.8450990000	-2.2641860000
H	-0.5405120000	6.8317710000	-0.8183960000
H	1.2136270000	6.6722660000	-0.6477970000
H	-0.2349550000	3.2101440000	-0.0092690000
H	0.8977370000	4.3931800000	0.6517660000
H	-0.8276250000	4.7863270000	0.5182320000
H	2.3953070000	4.6065320000	-1.3960380000

H	1.5287320000	3.2446950000	-2.1261870000
H	1.6751270000	4.7675700000	-3.0145120000
C	-2.8591710000	3.4582540000	-3.0620340000
C	-3.3657460000	2.3383990000	-3.7334940000
C	-3.6560400000	4.6141660000	-2.9133260000
C	-4.6535570000	2.3563010000	-4.2602350000
H	-2.7327070000	1.4687490000	-3.8675250000
C	-4.9431450000	4.6224810000	-3.4622420000
C	-5.4425030000	3.5008490000	-4.1225870000
H	-5.0356620000	1.4896270000	-4.7904710000
H	-5.5635630000	5.5068690000	-3.3586370000
H	-6.4471870000	3.5223940000	-4.5339350000
C	-3.1048030000	5.7947860000	-2.1753290000
C	-3.3332160000	7.1709600000	-2.7646330000
H	-4.3940690000	7.4499550000	-2.7030250000
H	-2.7676780000	7.9302630000	-2.2159550000
H	-3.0391840000	7.2051070000	-3.8179550000
H	-1.9030340000	5.5257910000	-2.2213910000
C	1.9304260000	0.7091960000	1.8101710000
O	2.1829670000	1.6354560000	0.9985970000
O	1.1449110000	-0.2823960000	1.5663810000
C	2.5612980000	0.7527980000	3.1852020000
H	1.8217750000	1.1370120000	3.8983820000
H	2.8456600000	-0.2514940000	3.5106310000
H	3.4269130000	1.4168410000	3.1916070000
O	0.8952150000	0.9651770000	-0.9959180000
H	1.4884370000	1.3870170000	-0.2120370000
H	0.3691850000	1.6483860000	-1.4895520000

^{5}TS-2

Charge: 1
Multiplicity: 5
B3LYP-D3(BJ)/6-31G(d)-SDD SCF energy (au): -1690.94148217
B3LYP-D3(BJ)/6-31G(d)-SDD enthalpy (au): -1690.36062717
B3LYP-D3(BJ)/6-31G(d)-SDD free energy (au): -1690.46996717
B3LYP-D3(BJ)/def2-TZVP/SMD SCF energy (au): -2831.49602977
B3LYP-D3(BJ)/def2-TZVP/SMD enthalpy (au): -2830.91517477
B3LYP-D3(BJ)/def2-TZVP/SMD free energy (au): -2831.02451477
B3LYP-D3(BJ)/def2-TZVP/SMD free energy (quasi-harmonic) (au): -2831.01585260

Cartesian coordinates

ATOM	X	Y	Z
Fe	0.1277060000	0.3699270000	-0.4839610000
C	0.8407880000	-0.0826320000	3.7897910000
N	1.9977410000	0.5069900000	3.3116660000
H	2.7888880000	0.7993020000	3.8686870000
C	1.8852260000	0.6474620000	1.9703570000
H	2.6267920000	1.0789120000	1.3146630000
N	0.7145390000	0.1730400000	1.5690490000
C	0.0490570000	-0.2828020000	2.6907770000
H	-0.9402620000	-0.7071580000	2.6196120000
C	-3.6885050000	-1.9344750000	0.0873620000
N	-4.0696380000	-0.6063660000	0.1540450000

H	-5.0149550000	-0.2548920000	0.2134200000
C	-2.9612140000	0.1634350000	0.0596200000
H	-2.9549710000	1.2406740000	0.0559210000
N	-1.8839130000	-0.6033680000	-0.0616910000
C	-2.3256860000	-1.9153310000	-0.0473840000
H	-1.6359620000	-2.7387010000	-0.1439820000
O	1.7637740000	1.0371640000	-1.0260470000
H	1.6826430000	1.8316080000	-1.5797950000
H	-4.4011240000	-2.7424490000	0.1406800000
H	0.6921040000	-0.2909340000	4.8375590000
C	1.6985300000	-3.4629500000	-1.8436900000
N	1.7830550000	-3.6571510000	-0.4768220000
H	2.1882800000	-4.4594150000	-0.0152730000
C	1.2542500000	-2.5717920000	0.1384800000
H	1.1969170000	-2.4502990000	1.2089580000
N	0.8292770000	-1.7006900000	-0.7642170000
C	1.1041720000	-2.2413190000	-2.0066960000
H	0.8573440000	-1.7016710000	-2.9063320000
H	2.0658380000	-4.1896510000	-2.5508590000
O	-0.5896980000	0.2168430000	-2.5099910000
H	-0.5273210000	1.1649380000	-2.8525990000
H	-1.5424230000	0.0466100000	-2.4257000000
F	-0.8295020000	1.9343620000	-0.1915360000
C	-1.3184450000	3.4750290000	-2.5492890000
O	-0.4429070000	2.7697370000	-3.1042780000
N	-1.1215860000	4.7516760000	-2.1808490000
H	-3.7746220000	5.5785120000	-0.6623480000
C	0.1518740000	5.4254880000	-1.8839710000
C	-0.2040530000	6.8552070000	-1.4466330000
C	0.9266670000	4.7093580000	-0.7678830000
C	0.9764130000	5.4804960000	-3.1883880000
H	-0.7892970000	7.3629410000	-2.2192560000
H	-0.7850800000	6.8496180000	-0.5170440000
H	0.7100600000	7.4289710000	-1.2682470000
H	1.1939910000	3.6919610000	-1.0543940000
H	1.8474140000	5.2605910000	-0.5481750000
H	0.3308380000	4.6508330000	0.1472420000
H	1.8880010000	6.0597280000	-3.0053920000
H	1.2454570000	4.4774470000	-3.5211130000
H	0.4054020000	5.9721390000	-3.9815270000
C	-2.7189330000	2.9639690000	-2.3534930000
C	-3.1718360000	1.9007080000	-3.1435260000
C	-3.5775470000	3.5454080000	-1.4020400000
C	-4.4537840000	1.3833160000	-2.9685750000
H	-2.5263420000	1.5147790000	-3.9259310000
C	-4.8649750000	3.0196340000	-1.2344390000
C	-5.2990180000	1.9388120000	-2.0035890000
H	-4.8024980000	0.5709710000	-3.5989890000
H	-5.5335340000	3.4739160000	-0.5078090000
H	-6.3082370000	1.5557930000	-1.8772050000
C	-3.0999220000	4.7182360000	-0.6002930000
C	-2.6126080000	4.4225370000	0.8068530000
H	-1.8673990000	3.6231310000	0.7947120000

H	-2.1844350000	5.3163230000	1.2722290000
H	-3.4565860000	4.0985960000	1.4333260000
H	-2.1165200000	5.0495520000	-1.2953840000

^{3}TS-2

Charge: 1
Multiplicity: 3
B3LYP-D3(BJ)/6-31G(d)-SDD SCF energy (au): -1690.92552005
B3LYP-D3(BJ)/6-31G(d)-SDD enthalpy (au): -1690.34233105
B3LYP-D3(BJ)/6-31G(d)-SDD free energy (au): -1690.45304105
B3LYP-D3(BJ)/def2-TZVP/SMD SCF energy (au): -2831.47118872
B3LYP-D3(BJ)/def2-TZVP/SMD enthalpy (au): -2830.88799972
B3LYP-D3(BJ)/def2-TZVP/SMD free energy (au): -2830.99870972
B3LYP-D3(BJ)/def2-TZVP/SMD free energy (quasi-harmonic) (au): -2830.98821467

Cartesian coordinates

ATOM	X	Y	Z
Fe	-0.1724940000	0.2513850000	-0.5693510000
C	0.8357680000	0.3247600000	3.7134860000
N	1.8244350000	1.0810420000	3.1095960000
H	2.5528730000	1.5944100000	3.5860660000
C	1.6404060000	1.0320400000	1.7677130000
H	2.2471720000	1.5335320000	1.0291030000
N	0.5887510000	0.2798020000	1.4838730000
C	0.0731290000	-0.1645690000	2.6861780000
H	-0.8041880000	-0.7925590000	2.7229780000
C	-3.5317270000	-2.1591400000	0.4694090000
N	-4.0566400000	-0.8845300000	0.3561610000
H	-5.0267110000	-0.6264020000	0.4718940000
C	-3.0558260000	-0.0281520000	0.0535150000
H	-3.1622230000	1.0347680000	-0.0926860000
N	-1.9110310000	-0.6976010000	-0.0346300000
C	-2.1921210000	-2.0275590000	0.2216760000
H	-1.4201170000	-2.7789500000	0.1939840000
O	1.3255050000	1.0561590000	-1.1478460000
H	1.0393740000	1.7120630000	-1.8140730000
H	-4.1421640000	-3.0162640000	0.7056560000
H	0.7694380000	0.2139950000	4.7844050000
C	1.6731130000	-3.3494820000	-1.9387840000
N	2.0522710000	-3.3498610000	-0.6090920000
H	2.6725120000	-4.0146850000	-0.1684760000
C	1.4772540000	-2.2852030000	-0.0014240000
H	1.6118720000	-2.0267820000	1.0363380000
N	0.7431050000	-1.6125620000	-0.8756180000
C	0.8592150000	-2.2606490000	-2.0918000000
H	0.3611350000	-1.8771370000	-2.9666450000
H	2.0138680000	-4.0998820000	-2.6343070000
O	-0.8891340000	0.0843670000	-2.6580360000
H	-0.7064870000	1.0142940000	-2.9818190000
H	-1.8568280000	0.0436780000	-2.5929480000
F	-1.0860910000	1.8139000000	-0.3061220000
C	-1.2507920000	3.4174590000	-2.5554870000
O	-0.4068650000	2.6651060000	-3.1031250000

N	-0.9771630000	4.6512400000	-2.1065820000
H	-3.6304310000	5.6004240000	-0.7036330000
C	0.3139520000	5.2371360000	-1.7150170000
C	0.0257280000	6.6982500000	-1.3340310000
C	0.9341590000	4.4934080000	-0.5217070000
C	1.2521570000	5.2076390000	-2.9410240000
H	-0.4435830000	7.2315540000	-2.1661530000
H	-0.6410850000	6.7539270000	-0.4655960000
H	0.9587090000	7.2073230000	-1.0747430000
H	1.1348270000	3.4473210000	-0.7536450000
H	1.8769840000	4.9774220000	-0.2428940000
H	0.2644910000	4.5231190000	0.3431450000
H	2.1753410000	5.7401610000	-2.6880700000
H	1.4926020000	4.1840880000	-3.2287580000
H	0.7842660000	5.7092920000	-3.7932700000
C	-2.6928740000	3.0050790000	-2.4476730000
C	-3.1880810000	2.0193940000	-3.3093510000
C	-3.5380200000	3.5748160000	-1.4779600000
C	-4.4934280000	1.5495050000	-3.1745390000
H	-2.5523800000	1.6497650000	-4.1073600000
C	-4.8462740000	3.0928970000	-1.3461300000
C	-5.3189540000	2.0758060000	-2.1769010000
H	-4.8734140000	0.7968160000	-3.8588010000
H	-5.5013220000	3.5321310000	-0.5982330000
H	-6.3410420000	1.7227070000	-2.0719520000
C	-3.0267960000	4.6905240000	-0.6163270000
C	-2.6602250000	4.3357090000	0.8126400000
H	-1.9744210000	3.4854660000	0.8303640000
H	-2.2050280000	5.1882560000	1.3271470000
H	-3.5662440000	4.0605420000	1.3723630000
H	-1.9842750000	4.9700180000	-1.2429970000

^{5}TS-3'

Charge: 1
Multiplicity: 5
B3LYP-D3(BJ)/6-31G(d)-SDD SCF energy (au): -1693.84972847
B3LYP-D3(BJ)/6-31G(d)-SDD enthalpy (au): -1693.27195547
B3LYP-D3(BJ)/6-31G(d)-SDD free energy (au): -1693.38574947
B3LYP-D3(BJ)/def2-TZVP/SMD SCF energy (au): -2834.41477160
B3LYP-D3(BJ)/def2-TZVP/SMD enthalpy (au): -2833.83699860
B3LYP-D3(BJ)/def2-TZVP/SMD free energy (au): -2833.95079260
B3LYP-D3(BJ)/def2-TZVP/SMD free energy (quasi-harmonic) (au): -2833.94062613

Cartesian coordinates

ATOM	X	Y	Z
Fe	-0.6607560000	0.5403190000	-1.0337070000
C	-2.3643250000	-2.9390010000	0.9697140000
N	-3.1491910000	-1.8894820000	1.4142350000
H	-3.9020410000	-1.9546110000	2.0851480000
C	-2.7318490000	-0.7566440000	0.8036870000
H	-3.1550740000	0.2244540000	0.9526050000
N	-1.7195970000	-1.0281780000	-0.0081920000
C	-1.4788480000	-2.3859650000	0.0855650000

H	-0.6976920000	-2.8570210000	-0.4899310000
H	-2.5090440000	-3.9507110000	1.3140760000
C	2.1840740000	-2.5138040000	-2.3702270000
N	2.8050010000	-1.9934630000	-1.2490080000
H	3.6874680000	-2.2996640000	-0.8630090000
C	2.0298540000	-0.9997660000	-0.7542050000
H	2.2498680000	-0.4189670000	0.1279370000
N	0.9447130000	-0.8569820000	-1.5029450000
C	1.0266820000	-1.7970840000	-2.5159300000
H	0.2472820000	-1.8806050000	-3.2565000000
H	2.6142170000	-3.3195160000	-2.9439240000
O	-1.5716050000	-0.1954140000	-2.8520550000
H	-1.4080450000	0.4032000000	-3.6471620000
H	-2.5290100000	-0.1503990000	-2.6930290000
F	-2.0936460000	1.6868480000	-0.7027840000
C	-1.7635950000	2.5541820000	-4.5586550000
O	-1.1445390000	1.5045270000	-4.8242080000
N	-1.3368320000	3.7771490000	-4.9160660000
H	-1.9759610000	4.5407450000	-4.7371430000
C	-0.1350030000	4.0986800000	-5.7324250000
C	-0.0812320000	5.6291330000	-5.8146150000
C	1.1309520000	3.5657530000	-5.0460690000
C	-0.2945980000	3.4889080000	-7.1339490000
H	-0.9831240000	6.0330940000	-6.2902620000
H	0.0223800000	6.0752160000	-4.8187810000
H	0.7778200000	5.9398940000	-6.4152300000
H	1.1110900000	2.4752470000	-5.0006060000
H	2.0108900000	3.8759210000	-5.6184930000
H	1.2168120000	3.9670230000	-4.0316820000
H	0.5752660000	3.7385440000	-7.7501310000
H	-0.3771370000	2.4012140000	-7.0736580000
H	-1.1884270000	3.8838860000	-7.6283300000
C	-3.1020410000	2.5032400000	-3.8763460000
C	-4.0795450000	1.7324350000	-4.5023060000
C	-3.3941280000	3.1878390000	-2.6532850000
C	-5.3782080000	1.6708500000	-3.9891080000
H	-3.8247700000	1.1987420000	-5.4129090000
C	-4.7285060000	3.1207730000	-2.1687620000
C	-5.7001350000	2.3850520000	-2.8269060000
H	-6.1351020000	1.0838100000	-4.4994040000
H	-4.9808030000	3.6365050000	-1.2495890000
H	-6.7113210000	2.3519360000	-2.4331220000
C	-2.3553670000	3.7794610000	-1.8945890000
C	-2.4965360000	4.5338840000	-0.6247460000
H	-3.3507890000	4.2120800000	-0.0249240000
H	-1.5825370000	4.4195810000	-0.0355430000
H	-2.6181350000	5.6094230000	-0.8320160000
H	-1.3570010000	3.7301340000	-2.3106610000
C	0.7103900000	2.3450400000	0.8431770000
O	0.7933900000	3.2577040000	-0.0098300000
O	0.4238040000	1.1138160000	0.5640150000
C	0.9402340000	2.6560990000	2.3045100000
H	-0.0247740000	2.6333760000	2.8244270000

H	1.5720740000	1.8893290000	2.7615910000
H	1.3890260000	3.6429700000	2.4247210000
O	0.2722780000	2.0684080000	-2.1965970000
H	0.5687110000	2.6501370000	-1.3966680000
H	1.0682660000	1.7838820000	-2.6720480000

^{3}TS-3'

Charge: 1
Multiplicity: 3
B3LYP-D3(BJ)/6-31G(d)-SDD SCF energy (au): -1693.81918759
B3LYP-D3(BJ)/6-31G(d)-SDD enthalpy (au): -1693.24074959
B3LYP-D3(BJ)/6-31G(d)-SDD free energy (au): -1693.35265159
B3LYP-D3(BJ)/def2-TZVP/SMD SCF energy (au): -2834.38717027
B3LYP-D3(BJ)/def2-TZVP/SMD enthalpy (au): -2833.80873227
B3LYP-D3(BJ)/def2-TZVP/SMD free energy (au): -2833.92063427
B3LYP-D3(BJ)/def2-TZVP/SMD free energy (quasi-harmonic) (au): -2833.91087168

Cartesian coordinates

ATOM	X	Y	Z
Fe	-0.5337290000	0.4741070000	-1.1181160000
C	-2.4177410000	-2.8962510000	0.9101520000
N	-3.0939730000	-1.7991090000	1.4129170000
H	-3.8090920000	-1.8189930000	2.1263970000
C	-2.6258700000	-0.6903480000	0.7914700000
H	-2.9651990000	0.3158220000	0.9804360000
N	-1.6865410000	-1.0215030000	-0.0807800000
C	-1.5449110000	-2.3944240000	-0.0169310000
H	-0.8354990000	-2.9145230000	-0.6414650000
H	-2.6195050000	-3.9003290000	1.2482730000
C	2.1582460000	-2.5043060000	-2.2338980000
N	2.7310380000	-2.0071560000	-1.0768900000
H	3.5867720000	-2.3338640000	-0.6499500000
C	1.9551110000	-1.0048180000	-0.6067580000
H	2.1351700000	-0.4319100000	0.2886950000
N	0.9114520000	-0.8378240000	-1.4108430000
C	1.0227040000	-1.7675420000	-2.4324150000
H	0.2762970000	-1.8287500000	-3.2075760000
H	2.6008810000	-3.3123090000	-2.7944210000
O	-1.4985220000	-0.2189390000	-2.8952590000
H	-1.3557070000	0.3936350000	-3.6779270000
H	-2.4390790000	-0.1226340000	-2.6716700000
F	-1.9129950000	1.6338180000	-0.8149180000
C	-1.7689780000	2.5553600000	-4.5818390000
O	-1.1524440000	1.5171310000	-4.8830720000
N	-1.3565460000	3.7929150000	-4.9076240000
H	-2.0022260000	4.5446290000	-4.7034210000
C	-0.1778170000	4.1460010000	-5.7455950000
C	-0.1366990000	5.6784400000	-5.7865940000
C	1.1081260000	3.6029960000	-5.1068800000
C	-0.3704810000	3.5738090000	-7.1587580000
H	-1.0527170000	6.0891670000	-6.2284970000
H	-0.0108540000	6.0974730000	-4.7816440000
H	0.7050520000	6.0118150000	-6.3994660000

H	1.0933140000	2.5120180000	-5.0853700000
H	1.9703650000	3.9321520000	-5.6955640000
H	1.2188500000	3.9781320000	-4.0852050000
H	0.4808940000	3.8470250000	-7.7905140000
H	-0.4431390000	2.4841720000	-7.1265320000
H	-1.2798790000	3.9750680000	-7.6187010000
C	-3.0989510000	2.4816260000	-3.8798250000
C	-4.1114720000	1.7940600000	-4.5459410000
C	-3.3482290000	3.0710700000	-2.6035320000
C	-5.3996760000	1.7273430000	-4.0082490000
H	-3.8920380000	1.3285670000	-5.5018620000
C	-4.6683000000	2.9996700000	-2.0900080000
C	-5.6765050000	2.3483550000	-2.7836810000
H	-6.1840300000	1.2049540000	-4.5466610000
H	-4.8839200000	3.4431450000	-1.1247450000
H	-6.6792150000	2.3086650000	-2.3696510000
C	-2.2720630000	3.5782480000	-1.8227660000
C	-2.3944590000	4.3307950000	-0.5500700000
H	-3.1983640000	3.9578290000	0.0895220000
H	-1.4469650000	4.2845030000	-0.0096240000
H	-2.6059760000	5.3907350000	-0.7642950000
H	-1.2884650000	3.5569740000	-2.2742460000
C	0.6778640000	2.3608230000	0.7634850000
O	0.7884410000	3.2493620000	-0.1158310000
O	0.4261730000	1.1209180000	0.5218310000
C	0.8313800000	2.7369550000	2.2218620000
H	-0.1681270000	2.8395130000	2.6619280000
H	1.3511440000	1.9480790000	2.7716150000
H	1.3580730000	3.6871970000	2.3231130000
O	0.4178110000	1.9923840000	-2.2957140000
H	0.6574970000	2.5885040000	-1.4889300000
H	1.2459080000	1.7327440000	-2.7278370000

[5]TS-3-OH
Charge: 1
Multiplicity: 5
B3LYP-D3(BJ)/6-31G(d)-SDD SCF energy (au): -1690.97145734
B3LYP-D3(BJ)/6-31G(d)-SDD enthalpy (au): -1690.38479934
B3LYP-D3(BJ)/6-31G(d)-SDD free energy (au): -1690.50114534
B3LYP-D3(BJ)/def2-TZVP/SMD SCF energy (au): -2831.53192102
B3LYP-D3(BJ)/def2-TZVP/SMD enthalpy (au): -2830.94526302
B3LYP-D3(BJ)/def2-TZVP/SMD free energy (au): -2831.06160902
B3LYP-D3(BJ)/def2-TZVP/SMD free energy (quasi-harmonic) (au): -2831.04842568

Cartesian coordinates

ATOM	X	Y	Z
Fe	-0.0357060000	0.6259070000	-1.1433790000
C	0.5411030000	1.3783600000	3.1572830000
N	1.2998640000	2.3454820000	2.5223460000
H	1.7769210000	3.1146000000	2.9714520000
C	1.2789830000	2.0955700000	1.1903010000
H	1.7671790000	2.6910080000	0.4339880000
N	0.5493630000	1.0197300000	0.9424870000

C	0.0771110000	0.5641600000	2.1586080000
H	-0.5571730000	-0.3068200000	2.2219170000
C	-3.1241020000	-2.0355100000	0.7206580000
N	-3.6864550000	-0.7714910000	0.7208130000
H	-4.5847660000	-0.5211470000	1.1098850000
C	-2.8303700000	0.0754890000	0.0984230000
H	-2.9839800000	1.1337720000	-0.0467090000
N	-1.7488520000	-0.5805880000	-0.2986980000
C	-1.9191250000	-1.8983100000	0.0833370000
H	-1.1740820000	-2.6463520000	-0.1383760000
O	1.5613480000	1.4262830000	-1.7890870000
H	2.2276090000	0.7473320000	-1.9867290000
H	-3.6240750000	-2.8877060000	1.1535850000
H	0.4076170000	1.3637820000	4.2275480000
C	1.8185300000	-3.4038890000	-1.4553040000
N	2.3028630000	-2.9939380000	-0.2265660000
H	2.9156580000	-3.5243320000	0.3769800000
C	1.8280430000	-1.7473560000	0.0207570000
H	2.0415360000	-1.1819920000	0.9144080000
N	1.0646150000	-1.3421060000	-0.9817490000
C	1.0508250000	-2.3654590000	-1.9126090000
H	0.4916960000	-2.2628780000	-2.8289300000
H	2.0635430000	-4.3658350000	-1.8773390000
O	-0.7132030000	-0.1505230000	-3.0225950000
H	-0.5486320000	0.5316640000	-3.7454160000
H	-1.6781030000	-0.1985820000	-2.9261700000
F	-1.2231940000	2.0370700000	-1.1297490000
C	-0.8282290000	2.7218870000	-5.0237580000
O	-0.5126330000	1.5121000000	-5.0205710000
N	-1.4596150000	3.2835570000	-6.0742500000
H	-1.5712870000	4.2877890000	-6.0448590000
C	-1.9079830000	2.5942690000	-7.3111640000
C	-2.6087760000	3.6628960000	-8.1595280000
C	-0.6917780000	2.0314930000	-8.0637060000
C	-2.8983080000	1.4762350000	-6.9505560000
H	-3.4725100000	4.0840260000	-7.6318030000
H	-1.9217120000	4.4780590000	-8.4165170000
H	-2.9669140000	3.2218330000	-9.0938030000
H	-0.1685900000	1.2937450000	-7.4523390000
H	-1.0186770000	1.5509240000	-8.9917740000
H	0.0065180000	2.8349220000	-8.3215720000
H	-3.2682360000	1.0017560000	-7.8652610000
H	-2.4152570000	0.7153260000	-6.3347820000
H	-3.7558640000	1.8840370000	-6.4043530000
C	-0.5406700000	3.6550260000	-3.8915630000
C	-1.5660210000	4.4966620000	-3.4560870000
C	0.7542000000	3.7281370000	-3.2887490000
C	-1.3604360000	5.4168970000	-2.4291470000
H	-2.5490510000	4.4106610000	-3.9103530000
C	0.9408200000	4.7045070000	-2.2770220000
C	-0.0915820000	5.5234890000	-1.8497900000
H	-2.1767070000	6.0469690000	-2.0908210000
H	1.9194540000	4.8011740000	-1.8205980000

H	0.0861900000	6.2505960000	-1.0626020000
C	1.8243640000	2.8724490000	-3.6728880000
C	3.2554520000	3.1206170000	-3.3256850000
H	3.3948500000	3.2901870000	-2.2532740000
H	3.8786440000	2.2698850000	-3.6158470000
H	3.6429390000	4.0059060000	-3.8524940000
H	1.6145610000	2.1196340000	-4.4214260000

[5]TS-3

Charge: 1
Multiplicity: 5
B3LYP-D3(BJ)/6-31G(d)-SDD SCF energy (au): -1690.96849496
B3LYP-D3(BJ)/6-31G(d)-SDD enthalpy (au): -1690.38134196
B3LYP-D3(BJ)/6-31G(d)-SDD free energy (au): -1690.49561096
B3LYP-D3(BJ)/def2-TZVP/SMD SCF energy (au): -2831.52617049
B3LYP-D3(BJ)/def2-TZVP/SMD enthalpy (au): -2830.93901749
B3LYP-D3(BJ)/def2-TZVP/SMD free energy (au): -2831.05328649
B3LYP-D3(BJ)/def2-TZVP/SMD free energy (quasi-harmonic) (au): -2831.04225653

Cartesian coordinates

ATOM	X	Y	Z
Fe	-0.6871620000	0.7391400000	-1.0785410000
C	0.8973180000	1.5048730000	2.9791100000
N	1.2791790000	2.6092450000	2.2380460000
H	1.7574920000	3.4252480000	2.5929410000
C	0.8915340000	2.4136550000	0.9526030000
H	1.0450470000	3.0961090000	0.1296750000
N	0.2840250000	1.2428730000	0.8368110000
C	0.2776070000	0.6656500000	2.0913700000
H	-0.1662620000	-0.3035880000	2.2589250000
C	-2.4215880000	-2.6918650000	1.1645100000
N	-3.2898600000	-1.6502240000	1.4390220000
H	-4.0990820000	-1.6955610000	2.0421880000
C	-2.8746900000	-0.5593180000	0.7482530000
H	-3.3499010000	0.4088250000	0.7679530000
N	-1.7877940000	-0.8483360000	0.0494420000
C	-1.4943380000	-2.1753280000	0.2984930000
H	-0.6490210000	-2.6567910000	-0.1676380000
O	0.2228690000	2.1804140000	-1.9370180000
H	0.8421680000	1.8776140000	-2.6167020000
H	-2.5475690000	-3.6734240000	1.5936640000
H	1.0972880000	1.4249910000	4.0361240000
C	2.0970960000	-2.4442510000	-2.3480190000
N	2.8394520000	-1.8020580000	-1.3741810000
H	3.7839560000	-2.0322390000	-1.0992640000
C	2.0833600000	-0.8001280000	-0.8590280000
H	2.4096160000	-0.1331670000	-0.0765730000
N	0.9000150000	-0.7689290000	-1.4502220000
C	0.8945760000	-1.7895880000	-2.3845820000
H	0.0284360000	-1.9626710000	-3.0030560000
H	2.4857630000	-3.2793070000	-2.9092830000
O	-1.6568980000	-0.2082690000	-2.8003740000
H	-1.4532810000	0.3270060000	-3.6243320000

H	-2.6144420000	-0.1207460000	-2.6677850000
F	-2.2136900000	1.8499900000	-0.6640580000
C	-1.7660400000	2.4651650000	-4.5444870000
O	-1.1146270000	1.4383210000	-4.8156900000
N	-1.3459480000	3.7112970000	-4.8231700000
H	-2.0107370000	4.4530710000	-4.6480880000
C	-0.1053790000	4.0918700000	-5.5484930000
C	-0.0669090000	5.6251070000	-5.5346370000
C	1.1284370000	3.5308980000	-4.8276670000
C	-0.1818540000	3.5677880000	-6.9911480000
H	-0.9505410000	6.0497680000	-6.0268410000
H	-0.0162970000	6.0083620000	-4.5089380000
H	0.8155640000	5.9808400000	-6.0733390000
H	1.1403530000	2.4405400000	-4.8813080000
H	2.0336400000	3.9139880000	-5.3102860000
H	1.1298140000	3.8317280000	-3.7764890000
H	0.7209700000	3.8532650000	-7.5410930000
H	-0.2624120000	2.4781310000	-6.9982430000
H	-1.0481210000	3.9892350000	-7.5124550000
C	-3.1477700000	2.3655220000	-3.9585660000
C	-4.0743930000	1.6340500000	-4.7023570000
C	-3.5346180000	2.9871490000	-2.7336060000
C	-5.4081930000	1.5513140000	-4.2982300000
H	-3.7470110000	1.1488680000	-5.6168170000
C	-4.9007240000	2.9052740000	-2.3616740000
C	-5.8194800000	2.2081340000	-3.1309500000
H	-6.1228000000	0.9938330000	-4.8953020000
H	-5.2257990000	3.3710580000	-1.4383560000
H	-6.8583480000	2.1599700000	-2.8188430000
C	-2.5489360000	3.5375090000	-1.8572450000
C	-2.8402200000	4.4296490000	-0.7030080000
H	-3.7459310000	4.1482140000	-0.1601480000
H	-2.0006460000	4.4162900000	-0.0042640000
H	-2.9708780000	5.4655670000	-1.0524030000
H	-1.5119210000	3.4794100000	-2.1745900000

^{3}TS-3

Charge: 1
Multiplicity: 3
B3LYP-D3(BJ)/6-31G(d)-SDD SCF energy (au): -1690.94345980
B3LYP-D3(BJ)/6-31G(d)-SDD enthalpy (au): -1690.35548580
B3LYP-D3(BJ)/6-31G(d)-SDD free energy (au): -1690.46779380
B3LYP-D3(BJ)/def2-TZVP/SMD SCF energy (au): -2831.50402869
B3LYP-D3(BJ)/def2-TZVP/SMD enthalpy (au): -2830.91605469
B3LYP-D3(BJ)/def2-TZVP/SMD free energy (au): -2831.02836269
B3LYP-D3(BJ)/def2-TZVP/SMD free energy (quasi-harmonic) (au): -2831.01784689

Cartesian coordinates

ATOM	X	Y	Z
Fe	-0.7006130000	0.5611590000	-1.1149670000
C	0.6711990000	1.8952590000	2.9877220000
N	1.0095660000	2.9323860000	2.1380810000
H	1.3967950000	3.8232560000	2.4153430000

C	0.7333040000	2.5414490000	0.8646490000
H	0.8861100000	3.1392100000	-0.0216260000
N	0.2393910000	1.3156050000	0.8578860000
C	0.1935580000	0.9008560000	2.1729050000
H	-0.1778030000	-0.0779500000	2.4384570000
C	-2.2718530000	-2.7480630000	1.0973560000
N	-3.1584950000	-1.7238770000	1.3786440000
H	-3.9682170000	-1.7876060000	1.9793250000
C	-2.7657500000	-0.6213110000	0.6980470000
H	-3.2598030000	0.3364550000	0.7201090000
N	-1.6722170000	-0.8874150000	-0.0021920000
C	-1.3531880000	-2.2113310000	0.2368610000
H	-0.5018950000	-2.6770100000	-0.2322410000
O	0.1272690000	1.9237430000	-2.0305390000
H	0.6623640000	1.5749670000	-2.7593600000
H	-2.3794610000	-3.7349400000	1.5187860000
H	0.8009120000	1.9626270000	4.0566450000
C	2.0209250000	-2.3373700000	-2.4162590000
N	2.7703850000	-1.6381320000	-1.4875080000
H	3.7343310000	-1.8185080000	-1.2452260000
C	1.9944170000	-0.6603340000	-0.9607040000
H	2.3096500000	0.0432390000	-0.2072110000
N	0.7865750000	-0.7047820000	-1.5047460000
C	0.7866420000	-1.7455550000	-2.4193070000
H	-0.0978690000	-1.9680870000	-2.9942730000
H	2.4262880000	-3.1650170000	-2.9763470000
O	-1.8433960000	-0.3844910000	-2.9302470000
H	-1.6084720000	0.1690760000	-3.7211280000
H	-2.7627010000	-0.1437390000	-2.7362080000
F	-2.1656310000	1.6820240000	-0.7382780000
C	-1.7668370000	2.4246490000	-4.5361810000
O	-1.1804380000	1.3846090000	-4.8814650000
N	-1.2805950000	3.6640170000	-4.7414270000
H	-1.9049510000	4.4281540000	-4.5200360000
C	-0.0386830000	4.0184400000	-5.4761750000
C	0.0886490000	5.5434170000	-5.3728940000
C	1.1782520000	3.3471820000	-4.8236120000
C	-0.1769080000	3.5878420000	-6.9450830000
H	-0.7798020000	6.0459260000	-5.8163750000
H	0.1833580000	5.8607450000	-4.3279100000
H	0.9779100000	5.8812980000	-5.9119920000
H	1.1251090000	2.2629000000	-4.9398480000
H	2.0925460000	3.7044970000	-5.3088100000
H	1.2248620000	3.5876810000	-3.7577730000
H	0.7280800000	3.8548520000	-7.5007750000
H	-0.3207320000	2.5069490000	-7.0142160000
H	-1.0290270000	4.0881500000	-7.4177200000
C	-3.1448280000	2.3688890000	-3.9319930000
C	-4.1233680000	1.7381840000	-4.7015280000
C	-3.4777110000	2.9304100000	-2.6648510000
C	-5.4517160000	1.6988170000	-4.2745910000
H	-3.8382320000	1.2940480000	-5.6501780000
C	-4.8354260000	2.8881380000	-2.2640310000

C	-5.8055100000	2.2924520000	-3.0567460000
H	-6.2056940000	1.2196520000	-4.8909570000
H	-5.1175930000	3.3075450000	-1.3048960000
H	-6.8389380000	2.2752780000	-2.7241250000
C	-2.4463520000	3.3878860000	-1.7784080000
C	-2.6822270000	4.2368120000	-0.5781090000
H	-3.5726290000	3.9463150000	-0.0148380000
H	-1.8193920000	4.1814750000	0.0897150000
H	-2.8092000000	5.2869190000	-0.8823910000
H	-1.4257740000	3.3348840000	-2.1427980000

benzyl radical
Charge: 0
Multiplicity: 2
B3LYP-D3(BJ)/6-31G(d)-SDD SCF energy (au): -636.239341674
B3LYP-D3(BJ)/6-31G(d)-SDD enthalpy (au): -635.938361674
B3LYP-D3(BJ)/6-31G(d)-SDD free energy (au): -635.998826674
B3LYP-D3(BJ)/def2-TZVP/SMD SCF energy (au): -636.509902367
B3LYP-D3(BJ)/def2-TZVP/SMD enthalpy (au): -636.208922367
B3LYP-D3(BJ)/def2-TZVP/SMD free energy (au): -636.269387367
B3LYP-D3(BJ)/def2-TZVP/SMD free energy (quasi-harmonic) (au): -636.267443693

Cartesian coordinates

ATOM	X	Y	Z
C	-0.0183400000	0.2415300000	-3.6542560000
O	-0.6218130000	0.0465660000	-2.5976030000
N	-0.2011640000	-0.5294420000	-4.7684740000
H	0.2116950000	-0.1935130000	-5.6265490000
C	-1.1344330000	-1.6696330000	-4.8758600000
C	-0.9812620000	-2.2192990000	-6.2998290000
C	-0.7503950000	-2.7514810000	-3.8535510000
C	-2.5792960000	-1.1942350000	-4.6449780000
H	-1.2433410000	-1.4601750000	-7.0475780000
H	0.0471560000	-2.5514860000	-6.4847350000
H	-1.6461070000	-3.0759130000	-6.4459790000
H	-0.8210470000	-2.3573010000	-2.8384790000
H	-1.4224660000	-3.6116710000	-3.9489590000
H	0.2758630000	-3.0932590000	-4.0273560000
H	-3.2747410000	-2.0356880000	-4.7411690000
H	-2.6786600000	-0.7655400000	-3.6460250000
H	-2.8552090000	-0.4332560000	-5.3837770000
C	0.9982210000	1.3363440000	-3.8279580000
C	2.1761030000	1.0563240000	-4.5212120000
C	0.7863920000	2.6467810000	-3.2735270000
C	3.1690430000	2.0225150000	-4.7042510000
H	2.3355030000	0.0503170000	-4.8992710000
C	1.8118080000	3.6126580000	-3.4954290000
C	2.9715810000	3.3091970000	-4.1889610000
H	4.0830690000	1.7724470000	-5.2340220000
H	1.6773970000	4.6139130000	-3.1004220000
H	3.7325110000	4.0727320000	-4.3258870000
C	-0.3852870000	2.9917540000	-2.5611530000
C	-0.6479340000	4.3567830000	-2.0066660000

H	-0.6140680000	5.1357650000	-2.7825860000
H	-1.6329400000	4.4018390000	-1.5343500000
H	0.0951410000	4.6437410000	-1.2470110000
H	-1.1141880000	2.2137100000	-2.3790770000

benzyl radical_Arg
Charge: 1
Multiplicity: 2
B3LYP-D3(BJ)/6-31G(d)-SDD SCF energy (au): -881.348122470
B3LYP-D3(BJ)/6-31G(d)-SDD enthalpy (au): -880.919631470
B3LYP-D3(BJ)/6-31G(d)-SDD free energy (au): -881.001373470
B3LYP-D3(BJ)/def2-TZVP/SMD SCF energy (au): -881.780057450
B3LYP-D3(BJ)/def2-TZVP/SMD enthalpy (au): -881.351566450
B3LYP-D3(BJ)/def2-TZVP/SMD free energy (au): -881.433308450
B3LYP-D3(BJ)/def2-TZVP/SMD free energy (quasi-harmonic) (au): -881.425923980

Cartesian coordinates

ATOM	X	Y	Z
C	-5.6633590000	-0.8097060000	-4.3164370000
C	-4.4326890000	-0.1684470000	-4.3448790000
C	-3.3117700000	-0.7761480000	-4.9482540000
C	-3.4261260000	-2.0633970000	-5.5287470000
C	-4.6755190000	-2.6909000000	-5.4701180000
C	-5.7818260000	-2.0816310000	-4.8806130000
H	-6.5212270000	-0.3245650000	-3.8626110000
H	-4.3261850000	0.8251160000	-3.9226350000
H	-4.7774410000	-3.6849680000	-5.8971220000
H	-6.7355940000	-2.6002130000	-4.8614840000
C	-2.2935020000	-2.7922130000	-6.2166810000
C	-2.2968440000	-2.5334710000	-7.7339450000
H	-2.4135070000	-3.8671870000	-6.0421300000
H	-1.3297770000	-2.5024020000	-5.7937130000
H	-1.4433190000	-3.0253920000	-8.2153690000
H	-3.2101950000	-2.9230330000	-8.1944660000
H	-2.2634630000	-1.4600280000	-7.9504790000
C	-2.0297670000	-0.0486250000	-4.8720460000
O	-1.0714250000	-0.1967610000	-5.6620930000
N	-1.9353170000	0.9053850000	-3.8904120000
C	-1.2639790000	0.6453170000	-2.6120680000
C	-1.6258680000	-0.7468800000	-2.0613360000
C	-1.6945860000	1.7500120000	-1.6392870000
H	-1.1447950000	-0.8903130000	-1.0892060000
H	-2.7068170000	-0.8457460000	-1.9269770000
H	-1.2864160000	-1.5431100000	-2.7322700000
H	-1.4601080000	2.7375360000	-2.0473250000
H	-2.7721220000	1.7012610000	-1.4542990000
H	-1.1767830000	1.6332850000	-0.6821370000
C	0.2603200000	0.7293550000	-2.8754400000
H	0.5868250000	-0.0520110000	-3.5666710000
H	0.5278570000	1.7101390000	-3.2818270000
H	0.7867170000	0.5970110000	-1.9249590000
H	2.8362310000	3.0654020000	-8.1724410000
C	3.2275850000	2.0452470000	-8.2658140000

N	2.1752240000	1.0871530000	-8.5922790000
C	1.2076680000	0.7158030000	-7.7402360000
N	1.1308180000	1.2475480000	-6.5178770000
N	0.3055060000	-0.2069190000	-8.1013060000
H	3.9635040000	2.0314390000	-9.0698660000
H	3.7305900000	1.7537180000	-7.3388740000
H	2.0972160000	0.7746050000	-9.5495020000
H	1.6320080000	2.0914200000	-6.2871750000
H	0.3778830000	0.9165360000	-5.9031750000
H	0.4653520000	-0.8068450000	-8.8966860000
H	-0.4116110000	-0.4732010000	-7.4260360000

benzyl radical_water
Charge: 0
Multiplicity: 2
B3LYP-D3(BJ)/6-31G(d)-SDD SCF energy (au): -712.642754030
B3LYP-D3(BJ)/6-31G(d)-SDD enthalpy (au): -712.313877030
B3LYP-D3(BJ)/6-31G(d)-SDD free energy (au): -712.381467030
B3LYP-D3(BJ)/def2-TZVP/SMD SCF energy (au): -712.958136430
B3LYP-D3(BJ)/def2-TZVP/SMD enthalpy (au): -712.629259430
B3LYP-D3(BJ)/def2-TZVP/SMD free energy (au): -712.696849430
B3LYP-D3(BJ)/def2-TZVP/SMD free energy (quasi-harmonic) (au): -712.693335400

Cartesian coordinates

ATOM	X	Y	Z
N	33.8843920000	34.8698210000	34.2929100000
O	35.1005740000	36.1612670000	32.8754590000
C	35.8116800000	33.8830540000	33.1964570000
C	35.2549500000	32.6081780000	33.4060460000
C	36.0147940000	31.4579170000	33.2293950000
C	37.3540220000	31.5770200000	32.8548450000
C	37.9126040000	32.8384130000	32.6572020000
C	37.1670840000	34.0138710000	32.8123600000
C	34.9473790000	35.0697350000	33.4367240000
C	33.9216970000	35.3146410000	35.6896000000
C	35.0657180000	36.2934310000	35.9978020000
C	32.5602360000	35.9664260000	35.9914770000
C	34.0867930000	34.0177750000	36.5190120000
C	37.8482590000	35.3386410000	32.5488890000
C	37.6922580000	35.7895160000	31.0867740000
H	34.2106430000	32.5379170000	33.6897460000
H	35.5679220000	30.4800660000	33.3806260000
H	37.9645570000	30.6888770000	32.7176400000
H	38.9587480000	32.9226290000	32.3736880000
H	37.4465710000	36.1153620000	33.2034760000
H	38.9141960000	35.2281810000	32.7831030000
H	38.2297030000	36.7290360000	30.9161510000
H	38.0934790000	35.0363360000	30.3991820000
H	36.6367430000	35.9489750000	30.8545120000
H	35.0403010000	36.5603460000	37.0595070000
H	36.0428610000	35.8447660000	35.7840700000
H	34.9490390000	37.2058080000	35.4091650000
H	32.4509940000	36.8838550000	35.4070400000

```
H     31.7450780000   35.2799870000   35.7430370000
H     32.5001120000   36.2142690000   37.0574390000
H     35.0421760000   33.5303320000   36.3013300000
H     34.0573140000   34.2720290000   37.5839690000
H     33.2769470000   33.3146550000   36.3033990000
O     33.4831690000   38.3233320000   33.7487720000
H     33.9601660000   37.5713820000   33.3413700000
H     34.1080390000   39.0595490000   33.6762130000
```

water

Charge: 0
Multiplicity: 1

```
B3LYP-D3(BJ)/6-31G(d)-SDD SCF energy (au):                -76.4075979388
B3LYP-D3(BJ)/6-31G(d)-SDD enthalpy (au):                  -76.3826819388
B3LYP-D3(BJ)/6-31G(d)-SDD free energy (au):               -76.4047809388
B3LYP-D3(BJ)/def2-TZVP/SMD SCF energy (au):               -76.4696566367
B3LYP-D3(BJ)/def2-TZVP/SMD enthalpy (au):                 -76.4447406367
B3LYP-D3(BJ)/def2-TZVP/SMD free energy (au):              -76.4668396367
B3LYP-D3(BJ)/def2-TZVP/SMD free energy (quasi-harmonic) (au): -76.4668396979
```

Cartesian coordinates

```
ATOM    X                Y                Z
O     -0.3884090000   -0.2449700000    0.0000000000
H      0.5789960000   -0.1974570000    0.0000000000
H     -0.6665480000    0.6828060000    0.0000000000
```

$His_2AspWat_2$_FeF_theozyme

Charge: 2
Multiplicity: 5

```
B3LYP-D3(BJ)/6-31G(d)-SDD SCF energy (au):                -3260.75679370
B3LYP-D3(BJ)/6-31G(d)-SDD enthalpy (au):                  -3259.48349470
B3LYP-D3(BJ)/6-31G(d)-SDD free energy (au):               -3259.68033270
B3LYP-D3(BJ)/def2-TZVP/SMD SCF energy (au):               -4402.21860609
B3LYP-D3(BJ)/def2-TZVP/SMD enthalpy (au):                 -4400.94530709
B3LYP-D3(BJ)/def2-TZVP/SMD free energy (au):              -4401.14214509
B3LYP-D3(BJ)/def2-TZVP/SMD free energy (quasi-harmonic) (au): -4401.11949639
```

Cartesian coordinates

```
ATOM    X                Y                Z
H     42.5038290000   47.0446510000   28.5015760000
C     43.4097510000   46.4756090000   28.4865960000
H     43.4937590000   45.9793960000   27.5146630000
C     44.5125520000   47.5439690000   28.6682810000
H     45.5153780000   47.1039310000   28.6482410000
H     44.4681730000   48.2433070000   27.8227030000
C     44.2771730000   48.3108750000   29.9870720000
H     43.2127580000   48.2452540000   30.2413430000
H     44.8190650000   47.8397210000   30.8171090000
C     44.6367980000   49.8021590000   29.9383540000
H     45.7173310000   49.9619200000   29.9367560000
H     44.2668820000   50.2585040000   29.0115860000
N     44.0991390000   50.5429770000   31.0895400000
H     44.7397990000   50.8577090000   31.8040370000
```

C	42.7966790000	50.7988960000	31.2943320000
N	41.8903610000	50.5790820000	30.3374100000
H	40.9025070000	50.7178520000	30.5823600000
H	42.1633620000	50.4678390000	29.3731180000
N	42.3835440000	51.3025300000	32.4699820000
H	42.8832280000	51.0260170000	33.3071460000
H	41.3671930000	51.4025400000	32.5685710000
H	43.5181060000	45.7284070000	29.2446310000
H	45.3297990000	43.5663220000	33.0145030000
C	45.7855520000	43.8763940000	33.9322950000
H	46.8683800000	43.9190670000	33.7730810000
C	45.2568460000	45.3079000000	34.2582520000
H	45.7951300000	45.7278610000	35.1113620000
H	45.4484120000	45.9586740000	33.3940780000
C	43.8029580000	45.3070980000	34.6111580000
N	42.8128840000	44.8159050000	33.7676890000
C	41.6155920000	44.9236530000	34.3851350000
H	40.6758380000	44.5869170000	33.9785270000
N	41.7823940000	45.4637900000	35.5852850000
H	42.9631160000	44.3836600000	32.8672010000
C	43.1359880000	45.7069640000	35.7409550000
H	43.5408130000	46.1269020000	36.6468150000
C	45.4329520000	42.9857360000	35.1195990000
O	44.4716150000	42.2142480000	35.1154890000
N	46.2069390000	43.2279150000	36.2061310000
H	46.9799770000	43.8770580000	36.1211260000
C	45.8521890000	42.9774280000	37.5861110000
H	45.1576370000	42.1351800000	37.6359040000
C	47.1638900000	42.6567900000	38.3667570000
H	46.9265780000	42.6349240000	39.4411780000
C	47.7720240000	41.3280450000	37.9493450000
H	48.7098110000	41.1701190000	38.4877020000
H	47.0913300000	40.5017840000	38.1784360000
H	47.9825950000	41.3233260000	36.8763270000
O	48.1206330000	43.6716630000	38.0865810000
H	47.8133010000	44.5139420000	38.4615870000
C	45.2489260000	44.2640110000	38.1964770000
O	45.5430570000	45.3679180000	37.7344770000
N	44.4717130000	44.1101440000	39.3020370000
H	44.3711190000	43.1830550000	39.6971190000
C	44.0650740000	45.2523440000	40.1274560000
H	44.0968570000	46.1290670000	39.4806700000
C	42.6970750000	45.0881860000	40.7775310000
H	42.6080530000	44.1377400000	41.3209850000
H	42.5936980000	45.8561690000	41.5527550000
C	41.4711030000	45.2098360000	39.8923470000
O	41.6486140000	45.2199470000	38.6180170000
O	40.3544130000	45.2838100000	40.4615150000
H	44.7755570000	45.4080890000	40.9409840000
H	36.1387690000	40.7413690000	41.5246690000
C	37.1406330000	40.3189580000	41.4362380000
H	37.0060690000	39.2318320000	41.4754870000
C	37.7543780000	40.6575730000	40.0625020000

H	38.6631060000	40.0592080000	39.9084240000
H	37.0585600000	40.3867610000	39.2647300000
C	38.1499990000	42.1017650000	39.8306860000
O	37.8146550000	42.7154190000	38.7859580000
N	38.9126050000	42.6869990000	40.7555370000
H	39.2836730000	43.6196570000	40.6079760000
H	39.2251680000	42.2398070000	41.6075050000
H	37.7806810000	40.5614190000	42.2840840000
H	42.7026240000	40.1710440000	33.7692100000
C	41.7036340000	40.3127830000	33.3572800000
H	41.5048180000	39.4419230000	32.7232670000
C	40.6429290000	40.3579750000	34.4755690000
H	39.6411900000	40.4264140000	34.0387110000
H	40.6702300000	39.4080720000	35.0267010000
C	40.7932440000	41.4886480000	35.4453280000
N	41.9747370000	41.7558540000	36.1245610000
H	42.9098890000	41.4365600000	35.8750100000
C	41.7749360000	42.8026240000	36.9454250000
H	42.5464860000	43.2664930000	37.5387510000
N	40.5182760000	43.2360760000	36.8527670000
C	39.8964270000	42.4152910000	35.9180600000
H	38.8549960000	42.5315850000	35.6623170000
H	41.6373040000	41.1894040000	32.7130610000
Fe	40.3114430000	45.3845840000	37.1392470000
O	38.7018410000	45.1706390000	38.4116430000
H	38.3781660000	44.2240110000	38.5453840000
H	39.1263420000	45.4188810000	39.2798420000
O	38.8681680000	45.3127660000	35.5752920000
H	38.0752000000	44.7600790000	35.8483230000
H	38.5615310000	46.2297980000	35.4060560000
C	40.5392630000	48.7974970000	34.3590820000
C	41.1586230000	48.1495590000	33.2917920000
C	42.5480390000	48.0451980000	33.2165310000
C	43.3351400000	48.6251530000	34.2220690000
C	42.7419940000	49.3125460000	35.2691700000
C	41.3227540000	49.4347420000	35.3784800000
H	40.5344800000	47.7077340000	32.5204380000
H	43.0097580000	47.5075950000	32.3977780000
H	44.4175500000	48.5324090000	34.1861970000
H	43.3647090000	49.7593920000	36.0365560000
C	40.7425190000	50.1889380000	36.4238740000
H	39.0454010000	48.0864240000	36.2431510000
H	39.6669140000	50.3209810000	36.4261080000
C	41.5159950000	50.8100070000	37.5405680000
H	42.2595670000	51.5357960000	37.1805690000
H	42.0662470000	50.0517370000	38.1160260000
H	40.8527770000	51.3352540000	38.2311060000
C	39.0371670000	48.7984500000	34.3523650000
O	38.4154890000	49.1826930000	33.3452460000
N	38.4261820000	48.2974220000	35.4546120000
C	36.9710520000	48.4505800000	35.7702000000
C	36.5847820000	49.9373310000	35.7365480000
C	36.1334780000	47.6498390000	34.7619570000

C	36.7808320000	47.8899270000	37.1855680000
H	37.1711650000	50.5059000000	36.4670680000
H	36.7438650000	50.3625620000	34.7435380000
H	35.5272720000	50.0510910000	35.9940480000
H	35.0706280000	47.7483690000	35.0037730000
H	36.2955880000	48.0085690000	33.7441980000
H	36.3870410000	46.5832070000	34.8009300000
H	35.7321990000	47.9908920000	37.4798280000
H	37.0548590000	46.8324630000	37.2491630000
H	37.3904820000	48.4355320000	37.9141710000
F	40.1901060000	47.2136770000	37.2906480000
O	36.9490280000	43.6063540000	36.3120050000
H	37.1090850000	43.1359880000	37.1602340000
H	36.0037840000	43.8197300000	36.2957480000
O	40.6766660000	43.5603320000	42.7641170000
H	40.6519180000	43.7558270000	43.7129760000
H	40.4776980000	44.4041350000	42.3177260000
O	39.6441080000	51.0622760000	31.8821780000
H	38.9753150000	51.7567890000	31.7786750000
H	39.1875180000	50.3107780000	32.3543050000

$His_2AspWat_2$_Fe_theozyme

Charge: 2
Multiplicity: 5
B3LYP-D3(BJ)/6-31G(d)-SDD SCF energy (au): -3260.68853372
B3LYP-D3(BJ)/6-31G(d)-SDD enthalpy (au): -3259.41609572
B3LYP-D3(BJ)/6-31G(d)-SDD free energy (au): -3259.61069672
B3LYP-D3(BJ)/def2-TZVP/SMD SCF energy (au): -4402.15419091
B3LYP-D3(BJ)/def2-TZVP/SMD enthalpy (au): -4400.88175291
B3LYP-D3(BJ)/def2-TZVP/SMD free energy (au): -4401.07635391
B3LYP-D3(BJ)/def2-TZVP/SMD free energy (quasi-harmonic) (au): -4401.05406419

Cartesian coordinates

ATOM	X	Y	Z
H	42.5036580000	47.0445370000	28.5014600000
C	43.4099960000	46.4760150000	28.4870150000
H	43.4454140000	45.9517690000	27.5255990000
C	44.5598170000	47.5010810000	28.5632380000
H	45.5366920000	47.0060850000	28.5009590000
H	44.4957770000	48.1669730000	27.6922100000
C	44.4825130000	48.3403050000	29.8479020000
H	43.4426740000	48.6470600000	30.0157530000
H	44.7724300000	47.7313960000	30.7142950000
C	45.3645930000	49.5934430000	29.8041490000
H	46.4047180000	49.3257320000	29.6030410000
H	45.0604140000	50.2542890000	28.9824620000
N	45.3764890000	50.3489030000	31.0677770000
H	46.2408290000	50.3738640000	31.5902320000
C	44.3647020000	51.0720290000	31.5707300000
N	43.2520230000	51.3025090000	30.8661560000
H	42.4904080000	51.7978060000	31.3365560000
H	43.2353180000	51.1944260000	29.8632920000
N	44.4629670000	51.5913550000	32.8051390000

H	45.0123090000	51.0932680000	33.4925460000
H	43.6318780000	52.0874830000	33.1496280000
H	43.5178380000	45.7281210000	29.2446000000
H	45.3308130000	43.5670830000	33.0134060000
C	45.7849840000	43.8750270000	33.9320090000
H	46.8685770000	43.9109920000	33.7699320000
C	45.2783230000	45.3084310000	34.2609800000
H	45.8177600000	45.7131370000	35.1211810000
H	45.5042690000	45.9518510000	33.4012410000
C	43.8204550000	45.3659210000	34.5928380000
N	42.8117650000	45.0566340000	33.6883630000
C	41.6137060000	45.2161570000	34.3049520000
H	40.6603690000	45.0197140000	33.8380180000
N	41.7937340000	45.6157660000	35.5558120000
H	42.9424420000	44.7178040000	32.7457140000
C	43.1614620000	45.7098090000	35.7472220000
H	43.5797810000	46.0040100000	36.6951180000
C	45.4463510000	42.9765450000	35.1152710000
O	44.5219880000	42.1642420000	35.1004160000
N	46.2009290000	43.2496240000	36.2093370000
H	46.9540990000	43.9212830000	36.1285080000
C	45.8498930000	42.9843170000	37.5871480000
H	45.1451370000	42.1501340000	37.6261880000
C	47.1577210000	42.6385160000	38.3631110000
H	46.9176140000	42.5987400000	39.4365210000
C	47.7544720000	41.3125800000	37.9217810000
H	48.6909050000	41.1369100000	38.4570900000
H	47.0668090000	40.4881910000	38.1360630000
H	47.9646770000	41.3258770000	36.8487420000
O	48.1238340000	43.6503830000	38.1038450000
H	47.8091110000	44.4916950000	38.4751040000
C	45.2678270000	44.2715970000	38.2135390000
O	45.6077650000	45.3775080000	37.7810260000
N	44.4634120000	44.1177470000	39.2952610000
H	44.3087360000	43.1847550000	39.6575290000
C	44.0640040000	45.2539410000	40.1299920000
H	44.1008310000	46.1337730000	39.4881470000
C	42.6872830000	45.1046110000	40.7663250000
H	42.5620030000	44.1358300000	41.2669130000
H	42.6097860000	45.8352190000	41.5802710000
C	41.4709030000	45.3206790000	39.8763330000
O	41.6550840000	45.5055380000	38.6249080000
O	40.3451380000	45.3129170000	40.4473220000
H	44.7764580000	45.4078360000	40.9404650000
H	36.1386980000	40.7413310000	41.5253400000
C	37.1400150000	40.3200700000	41.4359780000
H	37.0002510000	39.2332230000	41.4832750000
C	37.7727030000	40.6417910000	40.0692120000
H	38.6725550000	40.0256750000	39.9329450000
H	37.0826940000	40.3805480000	39.2636250000
C	38.1924630000	42.0794820000	39.8283110000
O	37.8646150000	42.6726390000	38.7781440000
N	38.9632940000	42.6567250000	40.7620650000

H	39.3541130000	43.5796230000	40.6132840000
H	39.2473570000	42.2084530000	41.6218380000
H	37.7810690000	40.5604590000	42.2841310000
H	42.7023980000	40.1715690000	33.7695640000
C	41.7039960000	40.3109990000	33.3550050000
H	41.5182300000	39.4431700000	32.7122880000
C	40.6347830000	40.3346850000	34.4655150000
H	39.6353060000	40.4118050000	34.0254210000
H	40.6590340000	39.3753230000	35.0002650000
C	40.7974440000	41.4512990000	35.4478410000
N	42.0148400000	41.7416950000	36.0428070000
H	42.9355000000	41.4162300000	35.7557310000
C	41.8540660000	42.7884880000	36.8736000000
H	42.6550450000	43.2665860000	37.4148220000
N	40.5871100000	43.1895550000	36.8723510000
C	39.9143870000	42.3568090000	35.9887330000
H	38.8495150000	42.4471210000	35.8347390000
H	41.6373820000	41.1898020000	32.7136170000
Fe	40.2827760000	45.2481020000	37.1241640000
O	38.6023580000	45.1418450000	38.4624060000
H	38.2988970000	44.1670260000	38.5628270000
H	39.0910990000	45.3476400000	39.3080880000
O	39.1199130000	46.4776060000	35.8729680000
H	38.2001970000	46.6552450000	36.2444050000
H	39.5869330000	47.3303370000	35.8453110000
C	41.9331000000	49.7042660000	35.0330150000
C	41.7613140000	49.2595280000	33.7142550000
C	42.7894850000	48.5981420000	33.0538450000
C	44.0171620000	48.4397350000	33.6970170000
C	44.1907440000	48.8905070000	35.0036540000
C	43.1496430000	49.5043780000	35.7154350000
H	40.8137860000	49.4371850000	33.2141350000
H	42.6407440000	48.2305310000	32.0457280000
H	44.8435080000	47.9656640000	33.1783500000
H	45.1439650000	48.7383870000	35.5037720000
C	43.3885110000	49.9120050000	37.1539020000
H	42.5870780000	50.5651100000	37.5074890000
H	44.3135090000	50.5000300000	37.2021010000
C	43.5190340000	48.7016650000	38.0982420000
H	44.3572660000	48.0571160000	37.8122870000
H	42.6102980000	48.0943930000	38.0944640000
H	43.6982190000	49.0475600000	39.1217010000
C	40.7886720000	50.4923240000	35.5872370000
O	40.3857500000	51.5203900000	35.0326470000
N	40.2029850000	50.0577730000	36.7483270000
C	38.8407690000	50.4159140000	37.2613390000
C	38.8886480000	51.9104390000	37.6211880000
C	37.7801730000	50.1216230000	36.1915200000
C	38.5896700000	49.5897910000	38.5315470000
H	39.6698130000	52.1034980000	38.3625200000
H	39.0659290000	52.5314980000	36.7435340000
H	37.9251170000	52.1897010000	38.0576440000
H	36.7904020000	50.3833410000	36.5783730000

H	37.9560120000	50.7108560000	35.2885520000
H	37.7670460000	49.0586310000	35.9305800000
H	37.6896360000	49.9790330000	39.0160770000
H	38.4259010000	48.5333870000	38.3063160000
H	39.4206130000	49.6780890000	39.2370140000
F	40.4817590000	48.6706330000	36.9356440000
O	36.8451960000	46.6448780000	37.1273710000
H	37.2424190000	46.1278030000	37.8686010000
H	36.3298030000	47.3690540000	37.5100370000
O	40.6806900000	43.6709350000	42.7885140000
H	40.5735330000	43.9400010000	43.7129580000
H	40.4689860000	44.4623580000	42.2576660000
O	41.8682620000	52.6100150000	32.9837380000
H	41.6555060000	53.5566070000	33.0044480000
H	41.3004580000	52.1938230000	33.6802040000

His$_2$AspWatOH_FeF_theozyme

Charge: 1
Multiplicity: 5
B3LYP-D3(BJ)/6-31G(d)-SDD SCF energy (au): -3260.39205093
B3LYP-D3(BJ)/6-31G(d)-SDD enthalpy (au): -3259.13184093
B3LYP-D3(BJ)/6-31G(d)-SDD free energy (au): -3259.33195893
B3LYP-D3(BJ)/def2-TZVP/SMD SCF energy (au): -4401.75413527
B3LYP-D3(BJ)/def2-TZVP/SMD enthalpy (au): -4400.49392527
B3LYP-D3(BJ)/def2-TZVP/SMD free energy (au): -4400.69404327
B3LYP-D3(BJ)/def2-TZVP/SMD free energy (quasi-harmonic) (au): -4400.66830534

Cartesian coordinates

ATOM	X	Y	Z
H	42.5038250000	47.0446350000	28.5015430000
C	43.4097330000	46.4756580000	28.4866620000
H	43.4782840000	45.9734340000	27.5158730000
C	44.5359900000	47.5207590000	28.6438600000
H	45.5274190000	47.0602990000	28.5541360000
H	44.4604370000	48.2541580000	27.8291100000
C	44.4031840000	48.2384010000	29.9965050000
H	43.3415490000	48.4301100000	30.1986280000
H	44.7518660000	47.5786750000	30.8004780000
C	45.1536070000	49.5733150000	30.0867960000
H	46.2363770000	49.4256610000	30.0659980000
H	44.9253110000	50.2053330000	29.2196020000
N	44.8540590000	50.3239250000	31.3126630000
H	45.4877190000	50.2288630000	32.0932870000
C	43.6735580000	50.9120060000	31.5810770000
N	42.7593190000	51.0915730000	30.6252840000
H	41.8283210000	51.4205730000	30.9200650000
H	42.9610860000	50.9028570000	29.6570250000
N	43.4149640000	51.3770480000	32.8144570000
H	43.8251040000	50.8822530000	33.6007530000
H	42.4481180000	51.6964890000	32.9539320000
H	43.5181230000	45.7283700000	29.2446050000
H	45.3298900000	43.5663100000	33.0144610000
C	45.7854340000	43.8763900000	33.9323340000

H	46.8701340000	43.9110900000	33.7773710000
C	45.2630990000	45.2989370000	34.2819540000
H	45.8062780000	45.7064570000	35.1390680000
H	45.4640160000	45.9641820000	33.4304640000
C	43.8110900000	45.3097600000	34.6349840000
N	42.8062980000	44.9236710000	33.7536590000
C	41.6084890000	45.0553140000	34.3781800000
H	40.6435480000	44.8134200000	33.9598200000
N	41.7947890000	45.5009130000	35.6084720000
H	42.9400980000	44.5574630000	32.8227920000
C	43.1530580000	45.6636270000	35.7861890000
H	43.5614800000	46.0233660000	36.7166070000
C	45.4310110000	42.9423280000	35.0871890000
O	44.5537290000	42.0814900000	35.0130230000
N	46.1287880000	43.2233580000	36.2169270000
H	46.8047200000	43.9752130000	36.1861910000
C	45.7349960000	42.8984850000	37.5715560000
H	44.9357080000	42.1544490000	37.5392280000
C	46.9640180000	42.3438430000	38.3486390000
H	46.6656700000	42.2232090000	39.4027090000
C	47.4361040000	41.0098950000	37.7948960000
H	48.3278900000	40.6827260000	38.3360710000
H	46.6578570000	40.2474820000	37.9041360000
H	47.6879220000	41.1079900000	36.7352730000
O	48.0458260000	43.2578220000	38.2357380000
H	47.7573450000	44.1271850000	38.5629550000
C	45.2989910000	44.2060530000	38.2657660000
O	45.8059480000	45.2783170000	37.9164730000
N	44.4507090000	44.0989020000	39.3109430000
H	44.0796150000	43.1907310000	39.5578900000
C	44.0650720000	45.2522590000	40.1274620000
H	44.0968190000	46.1280810000	39.4776870000
C	42.6960710000	45.0936390000	40.7719280000
H	42.6218640000	44.1551740000	41.3375650000
H	42.5873190000	45.8771160000	41.5308240000
C	41.4680590000	45.1917310000	39.8707390000
O	41.6243960000	45.2555900000	38.6200980000
O	40.3546700000	45.2009360000	40.4861720000
H	44.7755670000	45.4081540000	40.9409840000
H	36.1387560000	40.7413420000	41.5246710000
C	37.1406500000	40.3190270000	41.4362150000
H	37.0013490000	39.2316140000	41.4837970000
C	37.7603000000	40.6443650000	40.0642460000
H	38.6743720000	40.0508800000	39.9262510000
H	37.0701020000	40.3591120000	39.2665910000
C	38.1372020000	42.0941800000	39.8204870000
O	37.7361290000	42.7107570000	38.8094860000
N	38.9469520000	42.6720600000	40.7122840000
H	39.3195080000	43.6030910000	40.5404170000
H	39.2950250000	42.2232280000	41.5489670000
H	37.7806830000	40.5613950000	42.2840980000
H	42.7025920000	40.1709240000	33.7692310000
C	41.7038120000	40.3129750000	33.3573220000

H	41.4986720000	39.4397450000	32.7272260000
C	40.6568720000	40.3677440000	34.4876460000
H	39.6500650000	40.4339440000	34.0613180000
H	40.6908140000	39.4170010000	35.0389750000
C	40.8104460000	41.5036750000	35.4517910000
N	42.0018850000	41.8070140000	36.1017430000
H	42.9383110000	41.5207400000	35.8198970000
C	41.7724930000	42.8457470000	36.9377420000
H	42.5367570000	43.3471390000	37.5084960000
N	40.5038310000	43.2285500000	36.8832110000
C	39.8945070000	42.3919630000	35.9593230000
H	38.8478740000	42.4859910000	35.7173820000
H	41.6371760000	41.1893510000	32.7129960000
Fe	40.1224680000	45.4757470000	37.0128900000
O	38.6447600000	45.2559610000	38.4742720000
H	38.3097450000	44.3208960000	38.5391050000
H	39.1534640000	45.4003870000	39.3204340000
O	38.9344050000	45.3304270000	35.6094690000
H	38.2065310000	44.6902440000	35.7773760000
C	41.3257420000	49.0342180000	34.5013200000
C	42.0228000000	48.4160730000	33.4654140000
C	43.4018660000	48.2165600000	33.5362680000
C	44.1092510000	48.6879390000	34.6525470000
C	43.4396860000	49.3296100000	35.6824040000
C	42.0230360000	49.5170500000	35.6584910000
H	41.4622320000	48.0745820000	32.6004710000
H	43.9148520000	47.6902050000	32.7413340000
H	45.1815460000	48.5230800000	34.7306890000
H	43.9980070000	49.6752470000	36.5453460000
C	41.3614500000	50.1659600000	36.7252720000
H	39.5006180000	48.1574040000	36.0104200000
H	40.2894130000	50.2970430000	36.6538370000
C	42.0291160000	50.5944580000	37.9904270000
H	42.9247040000	51.2057810000	37.8134890000
H	42.3482920000	49.7203650000	38.5782260000
H	41.3459950000	51.1762520000	38.6139320000
C	39.8406950000	49.1803080000	34.3001570000
O	39.4204570000	49.7103470000	33.2392610000
N	39.0513660000	48.6936770000	35.2530110000
C	37.5655160000	48.6906420000	35.2245350000
C	37.0444570000	50.1290810000	35.0813150000
C	37.0814790000	47.8026330000	34.0670490000
C	37.1107140000	48.1006810000	36.5659090000
H	37.4036950000	50.7526000000	35.9083030000
H	37.3662910000	50.5721630000	34.1372420000
H	35.9497820000	50.1253290000	35.1110280000
H	35.9866710000	47.7525010000	34.0703270000
H	37.4071070000	48.2088960000	33.1060860000
H	37.4859860000	46.7939170000	34.1909790000
H	36.0166140000	48.0979860000	36.6088870000
H	37.4733490000	47.0772430000	36.6827620000
H	37.4845750000	48.6952980000	37.4067670000
F	40.2736530000	47.3209060000	37.2713850000

O 36.8794060000 43.4540010000 36.2725970000
H 37.0783350000 43.0243520000 37.1303500000
H 36.1029700000 44.0066710000 36.4483790000
O 40.6747390000 43.5666720000 42.7711670000
H 40.4974450000 43.9129990000 43.6579800000
H 40.5122800000 44.3197380000 42.1634630000
O 40.7138030000 51.7535550000 32.2880190000
H 40.0854710000 52.4866460000 32.3666710000
H 40.2257620000 50.9291170000 32.6194380000

His$_2$AspWatOH_Fe_theozyme

Charge: 1
Multiplicity: 5
B3LYP-D3(BJ)/6-31G(d)-SDD SCF energy (au): -3260.30710059
B3LYP-D3(BJ)/6-31G(d)-SDD enthalpy (au): -3259.04719559
B3LYP-D3(BJ)/6-31G(d)-SDD free energy (au): -3259.23969659
B3LYP-D3(BJ)/def2-TZVP/SMD SCF energy (au): -4401.67722663
B3LYP-D3(BJ)/def2-TZVP/SMD enthalpy (au): -4400.41732163
B3LYP-D3(BJ)/def2-TZVP/SMD free energy (au): -4400.60982263
B3LYP-D3(BJ)/def2-TZVP/SMD free energy (quasi-harmonic) (au): -4400.58935104

Cartesian coordinates

ATOM	X	Y	Z
H	42.5036320000	47.0445310000	28.5015590000
C	43.4100850000	46.4759930000	28.4868640000
H	43.4693630000	45.9692820000	27.5177580000
C	44.5457800000	47.5129010000	28.6212690000
H	45.5301960000	47.0436100000	28.5036890000
H	44.4548160000	48.2455640000	27.8074490000
C	44.4779750000	48.2418490000	29.9751770000
H	43.4289350000	48.4377710000	30.2332080000
H	44.8747430000	47.5970410000	30.7695980000
C	45.2473040000	49.5687140000	29.9790720000
H	46.3011840000	49.4016880000	29.7424630000
H	44.8667920000	50.2365010000	29.1951030000
N	45.2225140000	50.2729970000	31.2686940000
H	46.0799230000	50.3171930000	31.8002360000
C	44.1717960000	50.9245390000	31.7927520000
N	43.0327330000	51.0725050000	31.1135420000
H	42.2273430000	51.4588160000	31.6202840000
H	42.9630140000	50.8413650000	30.1354540000
N	44.2684340000	51.4746520000	33.0173010000
H	44.8348690000	50.9823430000	33.6994080000
H	43.3856590000	51.8556860000	33.3819250000
H	43.5177820000	45.7281480000	29.2446500000
H	45.3308450000	43.5670550000	33.0133770000
C	45.7849360000	43.8750640000	33.9320460000
H	46.8713170000	43.8528880000	33.7746160000
C	45.3500350000	45.3272910000	34.2446200000
H	45.9364180000	45.7376850000	35.0719100000
H	45.5733040000	45.9499590000	33.3668250000
C	43.9122710000	45.4369890000	34.6231760000
N	42.8656860000	45.2031970000	33.7390230000

C	41.6929710000	45.3984650000	34.4048110000
H	40.7008950000	45.2886970000	33.9913900000
N	41.9354090000	45.7365890000	35.6577580000
H	42.9526030000	44.8875860000	32.7842310000
C	43.3041000000	45.7623510000	35.8105900000
H	43.7643800000	46.0222860000	36.7508150000
C	45.4203630000	42.9475780000	35.0859350000
O	44.6050060000	42.0306380000	34.9872950000
N	46.0675830000	43.2701690000	36.2326170000
H	46.6958430000	44.0627090000	36.2271580000
C	45.6898720000	42.8826420000	37.5750770000
H	44.8575310000	42.1773220000	37.5191540000
C	46.9000410000	42.2308320000	38.3048600000
H	46.6022060000	42.0648710000	39.3536360000
C	47.2973270000	40.9079320000	37.6711220000
H	48.1787500000	40.5078660000	38.1794220000
H	46.4837620000	40.1794320000	37.7491680000
H	47.5409710000	41.0535270000	36.6149230000
O	48.0248180000	43.0929080000	38.2323640000
H	47.7655720000	43.9712870000	38.5610280000
C	45.3263390000	44.1735770000	38.3284020000
O	45.9350490000	45.2192000000	38.0620990000
N	44.4150340000	44.0909130000	39.3174680000
H	43.9430260000	43.2136360000	39.4932140000
C	44.0640170000	45.2538890000	40.1299790000
H	44.1199450000	46.1263380000	39.4784380000
C	42.6820890000	45.1442360000	40.7640730000
H	42.5542390000	44.1940280000	41.2981730000
H	42.6150810000	45.9032240000	41.5528900000
C	41.4548930000	45.3501180000	39.8706180000
O	41.6180080000	45.5908660000	38.6390190000
O	40.3381680000	45.2805980000	40.4700940000
H	44.7764550000	45.4078750000	40.9404710000
H	36.1386980000	40.7413340000	41.5253420000
C	37.1400160000	40.3200660000	41.4359730000
H	36.9920850000	39.2336870000	41.5006050000
C	37.7815560000	40.6080350000	40.0685310000
H	38.6803650000	39.9854830000	39.9567980000
H	37.0965800000	40.3259480000	39.2655360000
C	38.2033610000	42.0397130000	39.7842480000
O	37.8741030000	42.5968670000	38.7229580000
N	38.9811430000	42.6414070000	40.7019050000
H	39.3707310000	43.5598780000	40.5176120000
H	39.2613460000	42.2253490000	41.5785720000
H	37.7810680000	40.5604610000	42.2841320000
H	42.7023990000	40.1715690000	33.7695640000
C	41.7039930000	40.3110030000	33.3550100000
H	41.4997820000	39.4368090000	32.7261590000
C	40.6490280000	40.3790540000	34.4803190000
H	39.6449330000	40.4421370000	34.0475070000
H	40.6774120000	39.4368210000	35.0463440000
C	40.8152900000	41.5327120000	35.4219840000
N	42.0349240000	41.8525220000	36.0009870000

H	42.9561100000	41.5351300000	35.7049080000
C	41.8511160000	42.9201690000	36.8119000000
H	42.6472690000	43.4343790000	37.3252830000
N	40.5833780000	43.3021900000	36.8110080000
C	39.9243150000	42.4386680000	35.9525990000
H	38.8606810000	42.5249640000	35.7887680000
H	41.6373830000	41.1898020000	32.7136140000
Fe	40.1780920000	45.4524670000	37.0166470000
O	38.5538480000	45.1688390000	38.4780470000
H	38.3158840000	44.1932390000	38.5163520000
H	39.0446640000	45.3492370000	39.3205140000
O	39.1123280000	46.1285220000	35.6151020000
H	38.1675410000	45.9385250000	35.7791700000
C	42.2220410000	49.2576720000	35.4384900000
C	42.3482880000	48.7401830000	34.1399810000
C	43.5915030000	48.3781360000	33.6421260000
C	44.7323550000	48.5836090000	34.4239840000
C	44.6073510000	49.0990930000	35.7101350000
C	43.3527390000	49.4213720000	36.2578220000
H	41.4535740000	48.6113400000	33.5399830000
H	43.6709240000	47.9419200000	32.6538330000
H	45.7142670000	48.3137600000	34.0435520000
H	45.4931560000	49.2342090000	36.3253460000
C	43.2990530000	49.9049600000	37.6907290000
H	42.3210370000	50.3357060000	37.9181100000
H	44.0345300000	50.7095020000	37.8183300000
C	43.6117280000	48.7747830000	38.6895650000
H	44.6063090000	48.3520730000	38.5078930000
H	42.8799680000	47.9683280000	38.6053030000
H	43.5908190000	49.1598660000	39.7148380000
C	40.8545800000	49.7613010000	35.7902340000
O	40.3022230000	50.6209340000	35.0759960000
N	40.2334650000	49.3154710000	36.8986180000
C	38.8093040000	49.5417680000	37.3146360000
C	38.6798270000	51.0347370000	37.6562160000
C	37.8768920000	49.1284880000	36.1670210000
C	38.5376220000	48.6946520000	38.5632610000
H	39.3408690000	51.3030250000	38.4870720000
H	38.9154970000	51.6598860000	36.7934540000
H	37.6477680000	51.2377430000	37.9588100000
H	36.8390240000	49.1924860000	36.5151550000
H	37.9872790000	49.7984080000	35.3120200000
H	38.1124970000	48.1056550000	35.8548730000
H	37.5443130000	48.9630650000	38.9357170000
H	38.5424410000	47.6278570000	38.3375430000
H	39.2631900000	48.8938850000	39.3569990000
F	40.8306270000	48.1763140000	37.4458150000
O	36.4678940000	45.9610060000	36.8003140000
H	37.0523080000	45.7527020000	37.5583400000
H	36.3562110000	46.9215850000	36.8441800000
O	40.6310260000	43.7006730000	42.7945960000
H	40.3909040000	44.0810000000	43.6520750000
H	40.4649870000	44.4142680000	42.1417640000

O	41.5576090000	52.0734010000	33.2388760000
H	41.1357940000	52.9407000000	33.3372820000
H	41.0926200000	51.4733300000	33.8921120000

His_3Wat_2_FeF_theozyme

Charge: 3
Multiplicity: 5
B3LYP-D3(BJ)/6-31G(d)-SDD SCF energy (au): -2898.45553725
B3LYP-D3(BJ)/6-31G(d)-SDD enthalpy (au): -2897.25744725
B3LYP-D3(BJ)/6-31G(d)-SDD free energy (au): -2897.43442025
B3LYP-D3(BJ)/def2-TZVP/SMD SCF energy (au): -4039.91226972
B3LYP-D3(BJ)/def2-TZVP/SMD enthalpy (au): -4038.71417972
B3LYP-D3(BJ)/def2-TZVP/SMD free energy (au): -4038.89115272
B3LYP-D3(BJ)/def2-TZVP/SMD free energy (quasi-harmonic) (au): -4038.87283447

Cartesian coordinates

ATOM	X	Y	Z
H	50.4463900000	18.1352380000	40.5491810000
C	51.0759990000	18.9330260000	40.8839960000
H	50.6793990000	19.4297890000	41.7447000000
C	51.2268060000	19.9765890000	39.7422130000
C	51.3938080000	21.3859220000	40.2225600000
C	50.7252910000	22.5383650000	39.8978060000
N	52.3265160000	21.7784080000	41.1741030000
C	52.2148750000	23.1082850000	41.3826300000
N	51.2454170000	23.6075940000	40.6225390000
H	52.0449570000	18.5043540000	41.1613940000
H	52.0632360000	19.7050500000	39.0853190000
H	50.3335870000	19.9718220000	39.1098730000
H	52.9875030000	21.1649000000	41.6329450000
H	49.9453510000	22.6712910000	39.1643040000
H	52.8441750000	23.6665490000	42.0579120000
H	46.2257260000	28.1487280000	45.7837450000
C	46.7069880000	27.8000540000	44.8939800000
H	46.0294720000	27.2078360000	44.3150500000
C	48.0774520000	27.1375970000	45.0362480000
C	48.7832830000	27.1098890000	43.7082210000
C	49.7704090000	26.2690460000	43.1705780000
N	48.5586180000	28.0414110000	42.7306680000
C	49.4045690000	27.8026920000	41.6883330000
N	50.1298670000	26.6976190000	41.9245370000
H	46.8269930000	28.7333850000	44.3333340000
H	48.6932250000	27.6954030000	45.7549750000
H	48.0056230000	26.1168100000	45.4250050000
H	47.9491800000	28.8715240000	42.7990610000
H	50.1980900000	25.3802400000	43.6127960000
H	49.3264820000	28.2953520000	40.7352770000
H	43.0995510000	29.3205140000	42.0312280000
C	43.5559960000	29.7580370000	41.1680110000
H	42.8881030000	30.5217770000	40.7643040000
C	44.9349550000	30.3581750000	41.5682110000
H	45.6776160000	29.5548370000	41.5244240000
H	45.2328650000	31.0914430000	40.8144740000

C	45.0353460000	30.9708710000	42.9660780000
H	44.3826570000	31.8435430000	43.0748090000
H	44.7124670000	30.2395840000	43.7207120000
C	46.4775970000	31.3372850000	43.2961980000
O	47.4475760000	30.5946590000	43.0363490000
N	46.6627300000	32.5220360000	43.9040750000
H	45.8890050000	33.1205260000	44.1578440000
H	47.5887910000	32.7823470000	44.2166110000
C	43.9419700000	28.7781530000	40.0441780000
O	44.3516380000	29.2020820000	38.9683910000
N	44.0007740000	27.4422170000	40.3729160000
C	44.2630090000	26.4159910000	39.3470010000
H	43.3155570000	26.1085120000	38.9562240000
C	45.0449730000	25.2032480000	39.9015770000
C	46.5031050000	25.2235640000	39.5799410000
C	47.6309190000	25.4513010000	40.3236610000
N	46.9749050000	24.9755200000	38.2978240000
C	48.3235540000	25.0454580000	38.2978200000
N	48.7599060000	25.3394960000	39.5184930000
H	43.4312070000	27.1454000000	41.1567250000
H	44.8030100000	26.9216520000	38.5449490000
H	44.9235230000	25.1424400000	40.9875400000
H	44.6057350000	24.2830280000	39.4975810000
H	46.4097120000	24.7187380000	37.4983530000
H	47.7032290000	25.6526480000	41.3803070000
H	48.9276470000	24.8007980000	37.4370970000
H	48.4060690000	38.5789460000	37.9572130000
C	49.2070030000	38.8929080000	37.3210340000
H	48.8580890000	39.8190460000	36.8497580000
C	50.5176970000	39.1785140000	38.0627950000
H	51.2460890000	39.5807670000	37.3507670000
H	50.3626040000	39.9656870000	38.8123350000
C	51.1449330000	37.9539560000	38.7421530000
H	51.2389170000	37.1302650000	38.0195850000
H	52.1625050000	38.1928470000	39.0753930000
C	50.3371460000	37.4893350000	39.9569150000
H	50.2557880000	38.2948180000	40.6919180000
H	49.3119660000	37.2115690000	39.6949760000
N	50.9543800000	36.3483390000	40.6547600000
H	51.7253180000	36.5654870000	41.2735070000
C	50.7842720000	35.0629880000	40.3385900000
N	49.9446760000	34.6850670000	39.3643200000
H	49.6167650000	35.3469260000	38.6771290000
H	49.8170880000	33.6919250000	39.1911390000
N	51.4373980000	34.1046360000	41.0175320000
H	51.9119910000	34.3230750000	41.8804840000
H	51.2566730000	33.1309840000	40.7652470000
H	49.3592450000	38.1981040000	36.5217270000
Fe	50.7998110000	25.5813340000	40.0577790000
O	53.2174970000	26.2620060000	40.5173470000
H	52.7351630000	26.9697590000	40.0233840000
N	49.9162300000	29.7303970000	38.9139520000
C	51.9202780000	30.1171590000	40.1810520000

C	53.0630550000	30.0633930000	39.3934460000
C	54.2810850000	29.6560430000	39.9474200000
C	54.3678610000	29.2851950000	41.3008300000
C	53.2368040000	29.3259840000	42.0954640000
C	51.9880520000	29.7569980000	41.5636050000
C	50.6264640000	30.6286430000	39.5904110000
C	48.5785990000	29.9392980000	38.2755270000
C	48.0821820000	28.5464030000	37.8660070000
C	47.5990010000	30.5466400000	39.2887590000
C	48.7408570000	30.8413010000	37.0454140000
C	50.8356470000	29.8484270000	42.3784950000
C	50.8021090000	29.6045040000	43.8377180000
O	50.3174730000	31.8208490000	39.8023440000
F	51.2402090000	27.3623250000	39.3184040000
H	53.0089770000	30.3393210000	38.3453710000
H	55.1703620000	29.6293070000	39.3250100000
H	55.3217350000	28.9806350000	41.7188850000
H	53.3042710000	29.0511580000	43.1428430000
H	49.9591370000	30.3266480000	41.9507230000
H	50.3411570000	28.7960290000	38.8496390000
H	50.8544380000	28.5224600000	44.0567130000
H	51.6625810000	30.0579500000	44.3431460000
H	49.8769070000	29.9901780000	44.2676250000
H	48.7724900000	28.0659150000	37.1636250000
H	47.9619360000	27.8950970000	38.7382090000
H	47.1052610000	28.6384780000	37.3843510000
H	47.8526610000	31.5798740000	39.5331090000
H	46.5849540000	30.5084660000	38.8812420000
H	47.5956270000	29.9707170000	40.2197790000
H	49.1455280000	31.8177640000	37.3275300000
H	49.4124000000	30.3863920000	36.3104270000
H	47.7682750000	30.9984930000	36.5695620000
O	51.6037600000	24.9209500000	38.0390990000
H	52.5307660000	24.5837190000	38.2343950000
H	51.7128180000	25.7501680000	37.5445670000
O	53.9865800000	24.1764040000	38.8816230000
H	54.0786240000	24.8957000000	39.5380270000
H	54.8015060000	24.1568730000	38.3558670000
O	49.8684580000	23.0756720000	36.7451260000
H	50.6937410000	23.5187430000	37.0138630000
H	50.0935180000	22.5190770000	35.9842810000
H	53.3593820000	26.6189650000	41.4065430000

His_3Wat_2_Fe_theozyme
Charge: 3
Multiplicity: 5
B3LYP-D3(BJ)/6-31G(d)-SDD SCF energy (au): -2898.39635455
B3LYP-D3(BJ)/6-31G(d)-SDD enthalpy (au): -2897.19775655
B3LYP-D3(BJ)/6-31G(d)-SDD free energy (au): -2897.38135555
B3LYP-D3(BJ)/def2-TZVP/SMD SCF energy (au): -4039.85390262
B3LYP-D3(BJ)/def2-TZVP/SMD enthalpy (au): -4038.65530462
B3LYP-D3(BJ)/def2-TZVP/SMD free energy (au): -4038.83890362
B3LYP-D3(BJ)/def2-TZVP/SMD free energy (quasi-harmonic) (au): -4038.81484307

Cartesian coordinates

ATOM	X	Y	Z
H	50.4463830000	18.1352940000	40.5491870000
C	51.0760190000	18.9328850000	40.8839940000
H	50.6794050000	19.4298360000	41.7446950000
C	51.1468560000	19.9004970000	39.6716580000
C	51.5196870000	21.3088880000	39.9899240000
C	50.7500390000	22.4445440000	40.0672880000
N	52.8003480000	21.7331930000	40.3115630000
C	52.7824880000	23.0605590000	40.5651060000
N	51.5436950000	23.5274390000	40.4311700000
H	52.0596990000	18.5285220000	41.1399980000
H	51.8260650000	19.4951440000	38.9114120000
H	50.1589970000	19.9444170000	39.2009690000
H	53.6206190000	21.1396560000	40.3495780000
H	49.6856250000	22.5533060000	39.9208130000
H	53.6627840000	23.6481460000	40.7737830000
H	46.2257400000	28.1487440000	45.7837720000
C	46.7069460000	27.8000210000	44.8939860000
H	46.0294650000	27.2078320000	44.3150560000
C	48.0552520000	27.1151870000	45.1427630000
C	48.8412130000	27.0212590000	43.8711560000
C	49.5991030000	26.0391080000	43.2834810000
N	48.8915790000	28.0816640000	42.9765550000
C	49.6312050000	27.7414650000	41.9104000000
N	50.0875660000	26.5018740000	42.0627540000
H	46.8535470000	28.7246650000	44.3171920000
H	48.6287960000	27.6865600000	45.8842500000
H	47.9239290000	26.1120480000	45.5588380000
H	48.4193520000	28.9710830000	43.0737760000
H	49.8144900000	25.0432780000	43.6421800000
H	49.7845070000	28.3743750000	41.0531020000
H	43.0996680000	29.3204490000	42.0312650000
C	43.5558780000	29.7581050000	41.1679350000
H	42.7437900000	30.0450530000	40.4856890000
C	44.4028340000	30.9713570000	41.5502280000
H	45.1867900000	30.6605020000	42.2495120000
H	44.9242530000	31.3449900000	40.6660790000
C	43.5902320000	32.1029040000	42.1734480000
H	42.7950210000	32.4290470000	41.4890240000
H	43.0839460000	31.7694830000	43.0893870000
C	44.4713020000	33.3094870000	42.5054420000
O	45.6753110000	33.3436930000	42.2549070000
N	43.8257650000	34.3451220000	43.0967130000
H	42.8349960000	34.3315300000	43.2898950000
H	44.3485080000	35.1775270000	43.3319740000
C	44.4153550000	28.6917190000	40.4337890000
O	45.5050600000	28.9528010000	39.9226290000
N	43.8825450000	27.4295730000	40.3585890000
C	44.2630890000	26.4160490000	39.3468240000
H	43.3154910000	26.1084650000	38.9563840000
C	45.0994750000	25.2256200000	39.8757860000

C	46.5706140000	25.3148250000	39.6032180000
C	47.6717700000	25.3420000000	40.4195100000
N	47.0938720000	25.3107690000	38.3158050000
C	48.4442240000	25.3370280000	38.3826440000
N	48.8347120000	25.3508720000	39.6529440000
H	42.9562640000	27.3135100000	40.7510780000
H	44.7981500000	26.9437780000	38.5528710000
H	44.9485810000	25.1260940000	40.9539580000
H	44.7170850000	24.3009820000	39.4239450000
H	46.5547860000	25.2882600000	37.4598510000
H	47.7058600000	25.3563430000	41.4977990000
H	49.0961190000	25.3088250000	37.5227220000
H	48.4060880000	38.5788630000	37.9572150000
C	49.2070090000	38.8930950000	37.3209780000
H	48.8387400000	39.8101190000	36.8455320000
C	50.5217700000	39.2098510000	38.0453890000
H	51.1639920000	39.7847560000	37.3699570000
H	50.3325900000	39.8611620000	38.9083530000
C	51.3072030000	37.9702960000	38.4887830000
H	51.4139990000	37.2848030000	37.6359610000
H	52.3241180000	38.2519040000	38.7877550000
C	50.6446990000	37.2312720000	39.6550460000
H	50.7048880000	37.8181150000	40.5751460000
H	49.5797890000	37.0498070000	39.4810940000
N	51.2852140000	35.9347600000	39.9324940000
H	52.1644920000	35.9663270000	40.4332010000
C	51.0255340000	34.8102310000	39.2586250000
N	50.0274080000	34.7456920000	38.3674910000
H	49.6083850000	35.5818670000	37.9893040000
H	49.8128530000	33.8523820000	37.9368550000
N	51.7527280000	33.7020880000	39.4775770000
H	52.3302740000	33.6224360000	40.3013010000
H	51.4580580000	32.8470920000	39.0062920000
H	49.3592250000	38.1980050000	36.5217850000
Fe	50.8358130000	25.4791760000	40.4354460000
O	52.8978780000	26.0967430000	41.7226970000
H	52.5713930000	26.9943620000	41.9199050000
N	48.9888840000	29.8556680000	38.1480110000
C	50.7206570000	30.3475220000	39.8028810000
C	51.7540040000	29.4265620000	39.5997490000
C	52.6597820000	29.1559130000	40.6268860000
C	52.5072240000	29.7863190000	41.8655070000
C	51.4536870000	30.6824770000	42.0683820000
C	50.5494410000	30.9954110000	41.0438790000
C	49.8830870000	30.7535790000	38.6262510000
C	47.8423600000	30.0913180000	37.2056820000
C	47.1357910000	28.7452500000	36.9969450000
C	46.8862450000	31.1213650000	37.8220820000
C	48.4434930000	30.5737300000	35.8757940000
C	49.3959430000	31.9439720000	41.2930260000
C	48.1439760000	31.2376000000	41.8439720000
O	50.0745040000	31.8451240000	38.0678850000
F	48.7137270000	28.8436190000	39.0934270000

H	51.8581060000	28.9368670000	38.6372160000
H	53.4939000000	28.4866700000	40.4367970000
H	53.2200520000	29.6072070000	42.6660680000
H	51.3406940000	31.1668330000	43.0348730000
H	49.7088120000	32.7179520000	42.0021720000
H	49.1285110000	32.4611330000	40.3679560000
H	47.8335230000	30.4056360000	41.1998720000
H	48.3467020000	30.8646990000	42.8582530000
H	47.3066170000	31.9377780000	41.9237820000
H	47.8393850000	27.9857950000	36.6391880000
H	46.6562140000	28.4099590000	37.9174900000
H	46.3676450000	28.8870900000	36.2313290000
H	47.3991320000	32.0694500000	38.0078070000
H	46.0669230000	31.3145680000	37.1224750000
H	46.4579790000	30.7391370000	38.7520090000
H	48.9434700000	31.5363910000	35.9780010000
H	49.1588850000	29.8443680000	35.4833380000
H	47.6302670000	30.6794210000	35.1518800000
O	51.8674280000	26.1424870000	38.7826480000
H	52.8553690000	26.1452030000	38.9297490000
H	51.6962850000	25.4844720000	38.0571460000
O	54.4258360000	25.9141980000	39.4854440000
H	54.3486470000	26.0918830000	40.4410670000
H	55.2562660000	26.3062270000	39.1724910000
O	51.1461530000	24.1318340000	37.1385940000
H	51.5508500000	23.9020590000	36.2860230000
H	51.1367630000	23.3134120000	37.6649160000
H	52.8764310000	25.6308160000	42.5761030000

His_3WatOH_FeF_theozyme

Charge: 2
Multiplicity: 5
B3LYP-D3(BJ)/6-31G(d)-SDD SCF energy (au): -2898.20021760
B3LYP-D3(BJ)/6-31G(d)-SDD enthalpy (au): -2897.01463660
B3LYP-D3(BJ)/6-31G(d)-SDD free energy (au): -2897.19253760
B3LYP-D3(BJ)/def2-TZVP/SMD SCF energy (au): -4039.45699808
B3LYP-D3(BJ)/def2-TZVP/SMD enthalpy (au): -4038.27141708
B3LYP-D3(BJ)/def2-TZVP/SMD free energy (au): -4038.44931808
B3LYP-D3(BJ)/def2-TZVP/SMD free energy (quasi-harmonic) (au): -4038.42895048

Cartesian coordinates

ATOM	X	Y	Z
H	50.4463900000	18.1352380000	40.5491810000
C	51.0759990000	18.9330260000	40.8839960000
H	50.6793990000	19.4297890000	41.7447000000
C	51.2173580000	19.9841330000	39.7461770000
C	51.3113050000	21.3978930000	40.2320760000
C	50.6127030000	22.5253390000	39.8872000000
N	52.2073070000	21.8232950000	41.2082960000
C	52.0506630000	23.1491050000	41.4062350000
N	51.0828310000	23.6091300000	40.6184640000
H	52.0473820000	18.5084080000	41.1587450000
H	52.0836850000	19.7486280000	39.1142950000

H	50.3450370000	19.9424660000	39.0866760000
H	52.8850920000	21.2384990000	41.6784090000
H	49.8500460000	22.6354990000	39.1333600000
H	52.6482800000	23.7510210000	42.0719080000
H	46.2257260000	28.1487280000	45.7837450000
C	46.7069880000	27.8000540000	44.8939800000
H	46.0294720000	27.2078360000	44.3150500000
C	48.0971960000	27.1728470000	45.0320360000
C	48.7729580000	27.1125830000	43.6893910000
C	49.5607600000	26.1707020000	43.0682680000
N	48.6629150000	28.1478570000	42.7667580000
C	49.3510490000	27.8188380000	41.6584150000
N	49.9105930000	26.6235630000	41.8043520000
H	46.8099210000	28.7305830000	44.3217610000
H	48.7037440000	27.7745040000	45.7220510000
H	48.0532580000	26.1643150000	45.4547100000
H	48.1542430000	29.0280610000	42.8876680000
H	49.8895350000	25.2086270000	43.4311100000
H	49.4112400000	28.4340890000	40.7794010000
H	43.0995510000	29.3205140000	42.0312280000
C	43.5559960000	29.7580370000	41.1680110000
H	42.8859430000	30.5232500000	40.7692940000
C	44.9220370000	30.3615220000	41.5804850000
H	45.6590570000	29.5531370000	41.6245640000
H	45.2595060000	31.0445690000	40.7974380000
C	44.9481950000	31.0677430000	42.9357500000
H	44.2939710000	31.9469240000	42.9458460000
H	44.5797770000	30.3906760000	43.7197830000
C	46.3723620000	31.4536100000	43.3210380000
O	47.3518010000	30.7292010000	43.0908720000
N	46.5142050000	32.6418130000	43.9467600000
H	45.7218300000	33.2207290000	44.1840560000
H	47.4239560000	32.9041510000	44.3020860000
C	43.9008500000	28.7760010000	40.0334240000
O	44.1942690000	29.1925700000	38.9178730000
N	44.0291990000	27.4472400000	40.3718300000
C	44.2630090000	26.4159910000	39.3470010000
H	43.3155570000	26.1085120000	38.9562240000
C	45.0362300000	25.1919190000	39.8912230000
C	46.4958890000	25.1809360000	39.5722880000
C	47.6196740000	25.3376330000	40.3387940000
N	46.9757240000	24.9601480000	38.2873460000
C	48.3285090000	24.9859230000	38.3104260000
N	48.7538750000	25.2146110000	39.5461870000
H	43.5647080000	27.1424320000	41.2183480000
H	44.8035250000	26.9115700000	38.5386030000
H	44.9185770000	25.1258630000	40.9778000000
H	44.5774660000	24.2815900000	39.4850890000
H	46.4153100000	24.7676240000	37.4679900000
H	47.6851440000	25.5136500000	41.4001540000
H	48.9392730000	24.7690150000	37.4466420000
H	48.4060690000	38.5789460000	37.9572130000
C	49.2070030000	38.8929080000	37.3210340000

H	48.8723190000	39.8278260000	36.8572450000
C	50.5192500000	39.1431750000	38.0761160000
H	51.2545950000	39.5585890000	37.3784570000
H	50.3692310000	39.9092570000	38.8486000000
C	51.1291740000	37.8895460000	38.7194570000
H	51.2026080000	37.0849430000	37.9736230000
H	52.1550800000	38.1000380000	39.0464920000
C	50.3295960000	37.4011600000	39.9320980000
H	50.2871650000	38.1825240000	40.6964830000
H	49.2916410000	37.1688030000	39.6760700000
N	50.9120890000	36.2145410000	40.5729330000
H	51.7062690000	36.3691400000	41.1798400000
C	50.6836890000	34.9464530000	40.2004870000
N	49.8271900000	34.6587060000	39.2097260000
H	49.5757900000	35.3582130000	38.5285430000
H	49.6694810000	33.6750300000	38.9918410000
N	51.2850570000	33.9328300000	40.8335450000
H	51.7362500000	34.0731570000	41.7247530000
H	51.0498400000	32.9798560000	40.5228470000
H	49.3592450000	38.1981040000	36.5217270000
Fe	50.8367090000	25.6701770000	40.1435230000
O	52.5804900000	25.9489910000	40.8358240000
H	52.7629990000	26.8978730000	40.9529690000
N	49.6702060000	29.7416700000	38.7467500000
C	51.6329070000	30.1567980000	40.0565950000
C	52.6446580000	29.5957930000	39.2867670000
C	53.8061620000	29.0922030000	39.8824530000
C	53.9374690000	29.1350900000	41.2778080000
C	52.9290530000	29.6772130000	42.0619950000
C	51.7423170000	30.2171760000	41.4873820000
C	50.3914290000	30.6673520000	39.3864890000
C	48.3329470000	29.9233290000	38.1208580000
C	47.8178130000	28.5113830000	37.8043800000
C	47.3728280000	30.5925810000	39.1140080000
C	48.4714160000	30.7502340000	36.8358170000
C	50.6999060000	30.7269610000	42.3006280000
C	50.7052650000	30.6896280000	43.7959610000
O	50.0678750000	31.8751610000	39.4829650000
F	50.7238740000	27.2220380000	39.0634460000
H	52.5267040000	29.5502760000	38.2085770000
H	54.5986360000	28.6785510000	39.2671800000
H	54.8354800000	28.7483350000	41.7507720000
H	53.0463310000	29.6962300000	43.1398910000
H	49.8153220000	31.1310560000	41.8206350000
H	50.0525990000	28.7908490000	38.7563000000
H	50.9598370000	29.6922340000	44.1798510000
H	51.4381320000	31.3846870000	44.2336240000
H	49.7158970000	30.9521810000	44.1774640000
H	48.4897770000	27.9872620000	37.1159630000
H	47.7256560000	27.9139690000	38.7180280000
H	46.8274380000	28.5803910000	37.3461910000
H	47.6374240000	31.6363180000	39.2918270000
H	46.3499520000	30.5343730000	38.7318550000

H	47.3997440000	30.0691080000	40.0748140000
H	48.8901470000	31.7364320000	37.0552510000
H	49.1274520000	30.2478340000	36.1174170000
H	47.4903170000	30.8869010000	36.3698600000
O	51.6126800000	24.9517760000	38.1516190000
H	52.5697820000	24.7054630000	38.3799760000
H	51.6361000000	25.8471240000	37.7673120000
O	53.9584960000	24.4407810000	39.1581430000
H	53.6845110000	25.0481450000	39.9003620000
H	54.7768010000	24.8012990000	38.7835760000
O	50.0151320000	23.1417540000	36.6870860000
H	50.7709310000	23.6189460000	37.0798090000
H	50.3618100000	22.6958860000	35.9001190000

His_3WatOH_Fe_theozyme
Charge: 2
Multiplicity: 5
B3LYP-D3(BJ)/6-31G(d)-SDD SCF energy (au): -2898.12129405
B3LYP-D3(BJ)/6-31G(d)-SDD enthalpy (au): -2896.93719805
B3LYP-D3(BJ)/6-31G(d)-SDD free energy (au): -2897.11655105
B3LYP-D3(BJ)/def2-TZVP/SMD SCF energy (au): -4039.37969258
B3LYP-D3(BJ)/def2-TZVP/SMD enthalpy (au): -4038.19559658
B3LYP-D3(BJ)/def2-TZVP/SMD free energy (au): -4038.37494958
B3LYP-D3(BJ)/def2-TZVP/SMD free energy (quasi-harmonic) (au): -4038.35223353

Cartesian coordinates

ATOM	X	Y	Z
H	50.4463980000	18.1352480000	40.5491820000
C	51.0759960000	18.9330110000	40.8839900000
H	50.6794020000	19.4297940000	41.7447040000
C	51.1394800000	19.9463450000	39.7088270000
C	51.4659100000	21.3642670000	40.0669470000
C	50.6541790000	22.4691000000	40.2035930000
N	52.7437000000	21.8478860000	40.3103420000
C	52.6729370000	23.1802530000	40.5634670000
N	51.4146130000	23.5897050000	40.5019670000
H	52.0634470000	18.5358710000	41.1366140000
H	51.8370720000	19.5867470000	38.9412840000
H	50.1545770000	19.9738640000	39.2286780000
H	53.5928480000	21.2984460000	40.3043970000
H	49.5795710000	22.5286220000	40.1113950000
H	53.5322620000	23.8198510000	40.7017470000
H	46.2257140000	28.1487440000	45.7837420000
C	46.7070450000	27.7999780000	44.8940520000
H	46.0294340000	27.2078880000	44.3149890000
C	47.9600900000	26.9755720000	45.2785370000
C	48.9336300000	26.7934890000	44.1569860000
C	49.1519670000	25.7935480000	43.2387430000
N	49.8453700000	27.7825510000	43.8123330000
C	50.5546890000	27.3698580000	42.7363810000
N	50.1517690000	26.1687020000	42.3500010000
H	46.9849530000	28.6771070000	44.2996990000
H	48.4744040000	27.4652270000	46.1155330000

H	47.6545730000	25.9915280000	45.6486220000
H	49.9703170000	28.6741180000	44.2731070000
H	48.6643480000	24.8324710000	43.1667140000
H	51.3166510000	27.9422910000	42.2392300000
H	43.0995770000	29.3205290000	42.0312600000
C	43.5559650000	29.7579950000	41.1679510000
H	42.7356390000	30.0598120000	40.5002590000
C	44.3955470000	30.9720560000	41.5597900000
H	45.1824990000	30.6570700000	42.2499840000
H	44.9077370000	31.3602180000	40.6760620000
C	43.5639770000	32.0778020000	42.2053080000
H	42.7490970000	32.3895120000	41.5374330000
H	43.0801890000	31.7161670000	43.1238060000
C	44.4082680000	33.3024990000	42.5584570000
O	45.6318060000	33.3264130000	42.4553190000
N	43.6989280000	34.3767860000	42.9983950000
H	42.6960430000	34.3584890000	43.1083070000
H	44.2000980000	35.2042890000	43.2894240000
C	44.3984680000	28.7193850000	40.3838990000
O	45.4292660000	29.0257530000	39.7910080000
N	43.8999350000	27.4412750000	40.3510540000
C	44.2630290000	26.4159850000	39.3469960000
H	43.3155310000	26.1085250000	38.9562350000
C	45.0286330000	25.2019510000	39.9295330000
C	46.4946700000	25.1879390000	39.6455810000
C	47.5887600000	25.6029220000	40.3581510000
N	47.0182840000	24.6713900000	38.4666680000
C	48.3676590000	24.7749860000	38.5014990000
N	48.7470710000	25.3335550000	39.6450510000
H	43.0100970000	27.2975610000	40.8114590000
H	44.8367160000	26.9149440000	38.5637390000
H	44.8772600000	25.1782290000	41.0124680000
H	44.5840270000	24.2786260000	39.5372330000
H	46.4833000000	24.2648110000	37.7110750000
H	47.6235980000	26.0766520000	41.3254300000
H	49.0178810000	24.4153720000	37.7187250000
H	48.4060500000	38.5789320000	37.9572110000
C	49.2070490000	38.8929260000	37.3210370000
H	48.8644770000	39.8227310000	36.8518660000
C	50.5103860000	39.1712160000	38.0807030000
H	51.2397350000	39.6044040000	37.3873410000
H	50.3352270000	39.9346370000	38.8505360000
C	51.1553390000	37.9416330000	38.7342900000
H	51.3161220000	37.1585180000	37.9793460000
H	52.1492920000	38.2074510000	39.1153430000
C	50.3309410000	37.3821020000	39.8999240000
H	50.1480560000	38.1623830000	40.6440440000
H	49.3462380000	37.0267890000	39.5812940000
N	51.0045760000	36.2818410000	40.6049100000
H	51.7031430000	36.5411660000	41.2890090000
C	51.0158330000	35.0045750000	40.2022480000
N	50.3227630000	34.6104850000	39.1261790000
H	50.0024450000	35.2779940000	38.4421270000

H	50.3136500000	33.6191240000	38.8959700000
N	51.7148020000	34.0858560000	40.8832050000
H	51.9931170000	34.2517740000	41.8392020000
H	51.6751450000	33.1211780000	40.5449620000
H	49.3592180000	38.1980880000	36.5217260000
Fe	50.7680370000	25.6520110000	40.2696990000
O	51.9737540000	27.1785270000	40.2240820000
H	51.7315900000	27.7138870000	39.4515440000
N	49.8223320000	29.8431580000	39.5790070000
C	51.1273750000	30.6980780000	41.4777280000
C	52.3738150000	30.0792990000	41.6351090000
C	52.9752350000	30.0332370000	42.8913520000
C	52.3317170000	30.6135750000	43.9871160000
C	51.0970910000	31.2425970000	43.8183100000
C	50.4741940000	31.3069130000	42.5626580000
C	50.5746250000	30.8427040000	40.0914490000
C	48.9426340000	29.8593140000	38.3628350000
C	48.3380740000	28.4586980000	38.1998340000
C	47.8423370000	30.9119180000	38.5547970000
C	49.8425250000	30.1644470000	37.1541630000
C	49.1205730000	31.9631260000	42.4234790000
C	47.9731340000	31.0302490000	42.8396070000
O	50.8699110000	31.8389750000	39.4035880000
F	49.3609830000	28.9630200000	40.5682470000
H	52.8474900000	29.5964330000	40.7881990000
H	53.9363870000	29.5444120000	43.0131110000
H	52.7960670000	30.5866820000	44.9684870000
H	50.6064020000	31.7085540000	44.6698270000
H	49.0874420000	32.8645800000	43.0462780000
H	48.9563910000	32.2976050000	41.3932650000
H	47.9428460000	30.1408770000	42.2025610000
H	48.1037910000	30.7043510000	43.8785480000
H	47.0277780000	31.5700830000	42.7594310000
H	49.1166540000	27.6913100000	38.1769120000
H	47.6174580000	28.2331830000	38.9858300000
H	47.8074590000	28.4395450000	37.2430420000
H	48.2709270000	31.9074810000	38.7048600000
H	47.2130360000	30.9429570000	37.6599640000
H	47.1990830000	30.6515090000	39.3985330000
H	50.2982210000	31.1519390000	37.2174890000
H	50.6358960000	29.4150270000	37.0655510000
H	49.2281680000	30.1178390000	36.2501340000
O	51.4477690000	25.4333170000	38.0881110000
H	52.4212790000	25.4476130000	38.2703600000
H	51.2651510000	24.5665280000	37.6616570000
O	53.8935780000	25.6707860000	39.2594550000
H	53.3319470000	26.3701310000	39.7188240000
H	54.6619470000	26.1302670000	38.8886260000
O	50.6763820000	22.9559720000	37.1300440000
H	51.1487710000	22.5275880000	36.3990540000
H	50.8374580000	22.4097070000	37.9215180000

12-A_theozyme

Charge: 1
Multiplicity: 5
B3LYP-D3(BJ)/6-31G(d)-SDD SCF energy (au): -3132.02019251
B3LYP-D3(BJ)/6-31G(d)-SDD enthalpy (au): -3130.75165351
B3LYP-D3(BJ)/6-31G(d)-SDD free energy (au): -3130.96752751
B3LYP-D3(BJ)/def2-TZVP/SMD SCF energy (au): -4273.31794966
B3LYP-D3(BJ)/def2-TZVP/SMD enthalpy (au): -4272.04941066
B3LYP-D3(BJ)/def2-TZVP/SMD free energy (au): -4272.26528466
B3LYP-D3(BJ)/def2-TZVP/SMD free energy (quasi-harmonic) (au): -4272.23042515

Cartesian coordinates

ATOM	X	Y	Z
H	36.6176320000	24.2867520000	40.6866460000
C	36.2409030000	24.5417870000	39.6919670000
H	37.0603690000	24.9555310000	39.0977930000
C	35.1530830000	25.6387090000	39.8376860000
C	34.8540810000	26.4613860000	38.6137860000
C	35.3921710000	27.6429320000	38.1552420000
N	33.8399240000	26.1777390000	37.7072800000
C	33.7941660000	27.1644850000	36.7739600000
N	34.7291110000	28.0713380000	37.0162090000
H	35.8669220000	23.6420700000	39.1953140000
H	34.2222680000	25.1972630000	40.2158660000
H	35.4821650000	26.3415140000	40.6115750000
H	33.2148930000	25.3837820000	37.7420980000
H	36.1900720000	28.2303640000	38.5841860000
H	33.0560830000	27.2068670000	35.9870260000
H	29.7025460000	22.7110720000	37.5034010000
C	29.2400330000	23.2259100000	36.6470680000
H	28.9461840000	24.2328180000	36.9439750000
C	28.0795660000	22.5380320000	35.9473760000
C	27.6692710000	23.3702340000	34.7205710000
O	28.2803640000	24.4010160000	34.3660840000
O	26.6569620000	22.9448210000	34.0751320000
H	30.0531650000	23.3689370000	35.9271930000
H	28.3431640000	21.5320730000	35.6003510000
H	27.2024990000	22.4242520000	36.5942300000
H	25.6136270000	30.4503390000	42.5541610000
C	26.4050450000	30.7305890000	43.2551910000
H	26.1176250000	30.4443670000	44.2716060000
C	26.9041320000	32.1650410000	43.0765190000
C	27.8470050000	32.3214570000	41.8692950000
O	27.9549520000	31.3815500000	41.0286270000
O	28.4960920000	33.4057700000	41.7731560000
H	27.2333400000	30.0559060000	43.0022690000
H	26.0806360000	32.8793530000	42.9504880000
H	27.4593320000	32.5055660000	43.9586160000
H	41.2540500000	32.9343430000	33.7554340000
C	40.4492010000	32.4364000000	34.3021300000
H	40.7812270000	32.2475160000	35.3275560000
C	39.9620170000	31.1484880000	33.6123080000
C	38.7859560000	30.4867170000	34.2859730000
C	37.4755220000	30.8604020000	34.4965930000

N	38.8697810000	29.2118940000	34.8325250000
C	37.6522790000	28.8666880000	35.3323780000
N	36.7867700000	29.8474480000	35.1453620000
H	39.6217060000	33.1531550000	34.3690500000
H	39.6904210000	31.3802960000	32.5751230000
H	40.7855460000	30.4259470000	33.5507910000
H	39.6954370000	28.6293300000	34.8297580000
H	36.9642120000	31.7794130000	34.2527110000
H	37.4428710000	27.9296290000	35.8269200000
H	39.3343920000	34.2947870000	39.1395590000
C	38.6576600000	33.8739940000	39.8917060000
H	39.0647710000	34.1441680000	40.8703130000
C	38.5867600000	32.3248090000	39.8050950000
C	37.3324930000	31.7739200000	39.2127610000
C	37.0484590000	31.2501680000	37.9792780000
N	36.1337290000	31.6885440000	39.9115760000
C	35.1938640000	31.1292350000	39.1007840000
N	35.7244200000	30.8532450000	37.9214470000
H	37.6736890000	34.3395800000	39.7832610000
H	39.4294350000	31.9458920000	39.2183440000
H	38.7132380000	31.9006190000	40.8097050000
H	35.9837200000	31.9824330000	40.8666910000
H	37.7022380000	31.1489460000	37.1281330000
H	34.1582360000	30.9880800000	39.3858940000
O	29.8417420000	32.4804310000	39.4953330000
H	29.7707330000	33.1859880000	40.1733750000
H	29.1153730000	31.8971810000	39.8727050000
H	33.8251030000	43.3258380000	32.3960350000
C	32.7408300000	43.4797130000	32.3962030000
H	32.3584030000	43.2660970000	33.4029550000
C	32.0150470000	42.6263230000	31.3488040000
C	31.9474840000	41.1179360000	31.6422350000
C	33.3109740000	40.4075670000	31.6293880000
N	33.1930940000	38.9586980000	31.4043770000
C	33.1780260000	37.9754880000	32.3186150000
N	33.2796350000	38.2179420000	33.6308290000
N	33.0812700000	36.7016960000	31.9168260000
H	32.5734640000	44.5434240000	32.2003970000
H	30.9830580000	42.9840070000	31.2555510000
H	32.4765980000	42.7756510000	30.3629830000
H	31.4465670000	40.9397420000	32.6036010000
H	31.3168830000	40.6464510000	30.8771920000
H	33.9303710000	40.7962660000	30.8139750000
H	33.8801040000	40.5695120000	32.5488260000
H	32.9295560000	38.6851160000	30.4668160000
H	33.0598340000	39.1269650000	34.0076720000
H	33.2956760000	37.4049480000	34.2559090000
H	33.2561910000	36.4412640000	30.9581460000
H	33.0247250000	35.9660040000	32.6274630000
Fe	34.7380400000	30.1732650000	36.0735690000
N	32.7670480000	35.4079770000	36.6089630000
O	33.0876830000	35.4882290000	34.3904610000
C	31.7453520000	33.6649590000	35.2617480000

C	30.7934540000	33.4706420000	36.2832090000
C	29.9387930000	32.3755790000	36.2742080000
C	30.0333020000	31.4573040000	35.2261790000
C	30.9948240000	31.6257860000	34.2273440000
C	31.8748310000	32.7170850000	34.2191360000
C	32.6034610000	34.8683720000	35.3634290000
C	33.8989180000	35.1037930000	37.4857760000
C	35.1792210000	34.7742050000	36.6995560000
C	34.0924140000	36.3164780000	38.4071110000
C	33.4713640000	33.8696830000	38.3204740000
C	32.8998640000	32.7930590000	33.1121350000
C	32.2803730000	33.3044900000	31.8008120000
F	34.6615310000	31.9327640000	35.3872580000
H	30.7158430000	34.1936440000	37.0863690000
H	29.2374030000	32.2483840000	37.0904980000
H	29.3657300000	30.5994130000	35.2022620000
H	31.0907550000	30.8857590000	33.4367820000
H	33.7395730000	33.4159350000	33.4157040000
H	33.3025470000	31.7862910000	32.9639590000
H	33.0448640000	33.4006690000	31.0198500000
H	31.5132420000	32.6165090000	31.4295380000
H	31.7928130000	34.2761040000	31.9414240000
H	36.0024000000	34.6267430000	37.4078070000
H	35.0590460000	33.8508680000	36.1240670000
H	35.4477000000	35.5974910000	36.0287330000
H	34.3898240000	37.1983460000	37.8295700000
H	33.1675500000	36.5488960000	38.9433960000
H	34.8794410000	36.1070890000	39.1391010000
H	33.3700760000	32.9880280000	37.6867200000
H	34.2580920000	33.6768100000	39.0554300000
H	32.5321710000	34.0471720000	38.8506910000
O	33.8605220000	29.2691750000	34.2921440000
H	34.4946780000	28.8130570000	33.7209420000
H	32.9982390000	28.7708040000	34.2572110000
O	32.6593030000	30.2938220000	36.8233310000
H	32.2599600000	30.9625190000	36.2441460000
H	32.4136790000	30.5636890000	37.7532980000
O	31.4542590000	28.1476200000	34.1567820000
H	31.0433370000	27.3002650000	34.4817710000
H	30.8568370000	28.8479440000	34.4558600000
O	31.9985190000	30.9705330000	39.3516510000
H	31.2230950000	31.6217470000	39.4280790000
H	31.6751660000	30.1751720000	39.8029570000
O	30.2874290000	26.0233710000	35.3283050000
H	29.6607700000	25.3920860000	34.8856740000
H	29.7400330000	26.4106440000	36.0283930000

TS-2-A_theozyme
Charge: 1
Multiplicity: 5
B3LYP-D3(BJ)/6-31G(d)-SDD SCF energy (au): -3131.99502982
B3LYP-D3(BJ)/6-31G(d)-SDD enthalpy (au): -3130.73011182
B3LYP-D3(BJ)/6-31G(d)-SDD free energy (au): -3130.94853882

B3LYP-D3(BJ)/def2-TZVP/SMD SCF energy (au): -4273.31274133
B3LYP-D3(BJ)/def2-TZVP/SMD enthalpy (au): -4272.04782333
B3LYP-D3(BJ)/def2-TZVP/SMD free energy (au): -4272.26625033
B3LYP-D3(BJ)/def2-TZVP/SMD free energy (quasi-harmonic) (au): -4272.22883951

Cartesian coordinates

ATOM	X	Y	Z
H	36.6175660000	24.2867220000	40.6866880000
C	36.2408890000	24.5419910000	39.6917670000
H	37.0604330000	24.9554790000	39.0978540000
C	35.1618170000	25.6479650000	39.8214130000
C	34.9235550000	26.4526410000	38.5766730000
C	35.3973080000	27.6800280000	38.1807320000
N	34.1041120000	26.0486450000	37.5306340000
C	34.0931930000	27.0087790000	36.5749880000
N	34.8774740000	28.0183670000	36.9385130000
H	35.8649530000	23.6418980000	39.1973060000
H	34.2148760000	25.2211050000	40.1755380000
H	35.4798490000	26.3553580000	40.5949670000
H	33.5565750000	25.1988830000	37.5021050000
H	36.0579710000	28.3504790000	38.7078850000
H	33.4838930000	26.9526910000	35.6818400000
H	29.7028830000	22.7111790000	37.5032770000
C	29.2395430000	23.2255600000	36.6474040000
H	28.9461860000	24.2329230000	36.9437490000
C	28.0802600000	22.5419620000	35.9425570000
C	27.6724570000	23.3619780000	34.6964840000
O	28.2684840000	24.4190310000	34.3754130000
O	26.7079170000	22.8844650000	34.0302810000
H	30.0518550000	23.3682230000	35.9232390000
H	28.3348570000	21.5296190000	35.6082060000
H	27.1988250000	22.4426500000	36.5861830000
H	25.6135410000	30.4504430000	42.5542670000
C	26.4055960000	30.7302760000	43.2550480000
H	26.1172760000	30.4445490000	44.2715920000
C	26.8898180000	32.1702510000	43.0832190000
C	27.8035480000	32.3287930000	41.8630500000
O	27.8648280000	31.4159200000	40.9907890000
O	28.4957350000	33.3873150000	41.7638270000
H	27.2373510000	30.0596670000	43.0037280000
H	26.0582310000	32.8780670000	42.9740300000
H	27.4584090000	32.5096030000	43.9570440000
H	41.2540250000	32.9342630000	33.7553020000
C	40.4491780000	32.4364960000	34.3023150000
H	40.7812680000	32.2475690000	35.3275380000
C	39.9875970000	31.1384020000	33.6147690000
C	38.8004100000	30.4711940000	34.2642080000
C	37.5902800000	30.9277440000	34.7341700000
N	38.7406290000	29.0933340000	34.4457240000
C	37.5438590000	28.7697960000	34.9985520000
N	36.8249810000	29.8628140000	35.1901770000
H	39.6173360000	33.1486940000	34.3593480000
H	39.7426910000	31.3543490000	32.5662660000

H	40.8192110000	30.4227970000	33.5868290000
H	39.4648590000	28.4383440000	34.1848000000
H	37.2054210000	31.9330350000	34.7813040000
H	37.2364290000	27.7656520000	35.2478240000
H	39.3343460000	34.2947510000	39.1394480000
C	38.6576290000	33.8740690000	39.8920130000
H	39.0648550000	34.1441840000	40.8702140000
C	38.5331830000	32.3316130000	39.8152130000
C	37.2884850000	31.8339960000	39.1575540000
C	37.0779710000	31.0434370000	38.0610550000
N	36.0143740000	32.1105460000	39.6382530000
C	35.0982660000	31.5060020000	38.8431540000
N	35.7179770000	30.8454020000	37.8739620000
H	37.6874100000	34.3699390000	39.7861320000
H	39.3889970000	31.9029820000	39.2848560000
H	38.5770610000	31.9192090000	40.8323130000
H	35.7973060000	32.6746260000	40.4483100000
H	37.8065820000	30.6196590000	37.3907220000
H	34.0277450000	31.5515270000	39.0089010000
O	29.8097870000	32.4063410000	39.4389750000
H	29.8023200000	33.1243310000	40.1031510000
H	29.0560150000	31.8685600000	39.8176050000
H	33.8251850000	43.3260790000	32.3959540000
C	32.7408580000	43.4791760000	32.3963020000
H	32.3583450000	43.2663120000	33.4029670000
C	32.0162570000	42.5945600000	31.3569180000
C	31.3803800000	41.3201010000	31.9371750000
C	32.3737500000	40.2725470000	32.4519250000
N	31.6324710000	39.1883420000	33.1094630000
C	32.0325670000	37.9159700000	33.2749930000
N	33.2488670000	37.5080410000	32.8915190000
N	31.2120080000	37.0128920000	33.8241900000
H	32.5726240000	44.5407890000	32.1944700000
H	31.2022800000	43.1663400000	30.8982550000
H	32.6935130000	42.3300000000	30.5348990000
H	30.7208470000	41.6131990000	32.7679380000
H	30.7443310000	40.8442530000	31.1811000000
H	32.9365720000	39.8475850000	31.6136290000
H	33.0887890000	40.7225180000	33.1532510000
H	30.7634220000	39.4489620000	33.5560280000
H	33.9930260000	38.1724970000	32.7478180000
H	33.4667740000	36.5124240000	33.0463560000
H	30.2172050000	37.1791680000	33.8604060000
H	31.5834850000	36.0465240000	33.9124680000
Fe	34.8100700000	30.0636820000	36.0848860000
N	33.5200490000	32.9171400000	32.7265850000
O	32.8888630000	34.9121040000	33.7033280000
C	31.6880400000	32.9082770000	34.2512220000
C	31.1903650000	33.4765170000	35.4275750000
C	30.3317470000	32.7658080000	36.2672230000
C	29.9726680000	31.4583530000	35.9287880000
C	30.4613780000	30.8762720000	34.7559280000
C	31.3070850000	31.5901760000	33.8880630000

C	32.7567660000	33.6593820000	33.5013160000
C	34.7318310000	33.3319600000	32.0196640000
C	35.7129670000	34.0139710000	32.9937570000
C	35.3790610000	32.0393980000	31.4919600000
C	34.3748830000	34.2545800000	30.8414050000
C	31.8452490000	30.9752670000	32.6483520000
C	31.2148640000	31.4222420000	31.3334410000
F	34.6910820000	31.7124280000	35.2993700000
H	31.5335540000	34.4670450000	35.7081020000
H	29.9778850000	33.1864400000	37.2024500000
H	29.3228850000	30.8921260000	36.5899600000
H	30.1948150000	29.8545710000	34.4972440000
H	32.9894370000	31.7182080000	32.6215010000
H	31.8891580000	29.8840160000	32.7126130000
H	31.7672950000	31.0189440000	30.4766150000
H	30.1751170000	31.0744170000	31.2524870000
H	31.2090970000	32.5134970000	31.2470770000
H	36.6928670000	34.1367960000	32.5166680000
H	35.3499170000	34.9943800000	33.3078860000
H	35.8140700000	33.3941210000	33.8881020000
H	35.6596320000	31.3864890000	32.3259780000
H	34.6865050000	31.4957130000	30.8396610000
H	36.2803330000	32.2704640000	30.9143530000
H	33.8612860000	35.1534280000	31.1933950000
H	35.2789010000	34.5586820000	30.3010510000
H	33.7123130000	33.7382830000	30.1387490000
O	33.9486550000	29.2042950000	34.3119310000
H	33.7356880000	29.9649330000	33.7390670000
H	33.1277200000	28.6410650000	34.3444250000
O	32.8116840000	30.1276450000	36.8328110000
H	32.2316960000	30.5709020000	36.1849700000
H	32.5034080000	30.4590760000	37.7307990000
O	31.9312730000	27.4309220000	34.2050960000
H	31.1100500000	27.0850640000	34.6730700000
H	31.9216350000	27.0032140000	33.3356580000
O	32.0267500000	31.0152120000	39.2161710000
H	31.1929110000	31.5938520000	39.2836290000
H	31.8447050000	30.2635620000	39.8012410000
O	29.7918160000	26.3798420000	35.3936070000
H	29.3429070000	25.5647490000	35.0252820000
H	29.0555210000	26.9987420000	35.5117070000

TS-6_theozyme

Charge: 1
Multiplicity: 5
B3LYP-D3(BJ)/6-31G(d)-SDD SCF energy (au): -3132.00372344
B3LYP-D3(BJ)/6-31G(d)-SDD enthalpy (au): -3130.73977144
B3LYP-D3(BJ)/6-31G(d)-SDD free energy (au): -3130.95690644
B3LYP-D3(BJ)/def2-TZVP/SMD SCF energy (au): -4273.31285587
B3LYP-D3(BJ)/def2-TZVP/SMD enthalpy (au): -4272.04890387
B3LYP-D3(BJ)/def2-TZVP/SMD free energy (au): -4272.26603887
B3LYP-D3(BJ)/def2-TZVP/SMD free energy (quasi-harmonic) (au): -4272.23053643

Cartesian coordinates

ATOM	X	Y	Z
H	36.6176230000	24.2867560000	40.6866550000
C	36.2409240000	24.5417860000	39.6919380000
H	37.0603580000	24.9555310000	39.0978090000
C	35.1335020000	25.6187170000	39.8176060000
C	34.8526960000	26.4160480000	38.5747350000
C	35.3870720000	27.6014140000	38.1250200000
N	33.9059580000	26.0843540000	37.6132770000
C	33.8889580000	27.0437500000	36.6576860000
N	34.7855570000	27.9853720000	36.9356750000
H	35.8814830000	23.6349430000	39.1966870000
H	34.2040790000	25.1635980000	40.1819030000
H	35.4362830000	26.3365590000	40.5884010000
H	33.2783240000	25.2899400000	37.6180490000
H	36.1446870000	28.2157210000	38.5878090000
H	33.1998410000	27.0223540000	35.8269910000
H	29.7025730000	22.7110700000	37.5033840000
C	29.2400130000	23.2259190000	36.6470920000
H	28.9461660000	24.2328070000	36.9439730000
C	28.2219370000	22.6546690000	35.6906950000
C	28.0881040000	23.5773370000	34.4465520000
O	28.8810100000	24.5967950000	34.2487680000
O	27.2019670000	23.2940740000	33.6400950000
H	30.1362700000	23.4331930000	36.0458820000
H	28.5095830000	21.6655300000	35.3159320000
H	27.2198950000	22.5477680000	36.1178560000
H	25.6136260000	30.4503430000	42.5541740000
C	26.4050520000	30.7305820000	43.2551680000
H	26.1176190000	30.4443690000	44.2716140000
C	26.8985150000	32.1675620000	43.0781490000
C	27.8238410000	32.3194320000	41.8650260000
O	27.8879910000	31.4102260000	40.9892550000
O	28.5253410000	33.3728350000	41.7719190000
H	27.2342110000	30.0558660000	43.0047670000
H	26.0714180000	32.8789460000	42.9583630000
H	27.4614960000	32.5090420000	43.9547530000
H	41.2540120000	32.9343590000	33.7554210000
C	40.4492780000	32.4363820000	34.3021410000
H	40.7811990000	32.2475180000	35.3275580000
C	39.9500440000	31.1484890000	33.6151170000
C	38.7763930000	30.4744160000	34.2882690000
C	37.4529190000	30.8164080000	34.4687790000
N	38.8791080000	29.2065910000	34.8521670000
C	37.6636890000	28.8337140000	35.3308110000
N	36.7763470000	29.7913460000	35.1156370000
H	39.6246430000	33.1565420000	34.3712540000
H	39.6692920000	31.3836220000	32.5814030000
H	40.7720040000	30.4252430000	33.5427930000
H	39.7175560000	28.6423860000	34.8694820000
H	36.9238550000	31.7183630000	34.2029470000
H	37.4665930000	27.8969660000	35.8305950000
H	39.3343860000	34.2947850000	39.1395590000

C	38.6576720000	33.8739960000	39.8917060000
H	39.0647670000	34.1441690000	40.8703150000
C	38.5555640000	32.3272700000	39.7821870000
C	37.3095650000	31.7912660000	39.1481470000
C	37.0586010000	31.2252730000	37.9244490000
N	36.0902280000	31.7274430000	39.8136370000
C	35.1731370000	31.1390090000	39.0012340000
N	35.7347570000	30.8216540000	37.8445900000
H	37.6804680000	34.3557210000	39.7863970000
H	39.4095460000	31.9380220000	39.2190970000
H	38.6418900000	31.8891640000	40.7851610000
H	35.9142260000	32.0469670000	40.7561540000
H	37.7355030000	31.0962410000	37.0956600000
H	34.1366360000	30.9891940000	39.2775320000
O	29.8604480000	32.4048480000	39.4456930000
H	29.8423280000	33.1124760000	40.1212520000
H	29.0936450000	31.8734020000	39.8045830000
H	33.8251060000	43.3258300000	32.3960420000
C	32.7408190000	43.4797310000	32.3961920000
H	32.3584110000	43.2660890000	33.4029580000
C	32.0082650000	42.6361890000	31.3446280000
C	31.8793280000	41.1339720000	31.6504070000
C	33.2151060000	40.3727080000	31.6838600000
N	33.0573530000	38.9299870000	31.4408210000
C	32.9093680000	37.9513560000	32.3483960000
N	32.8993420000	38.1953630000	33.6644230000
N	32.7916130000	36.6818670000	31.9387370000
H	32.5773480000	44.5449000000	32.2044440000
H	30.9910030000	43.0271860000	31.2266430000
H	32.4933690000	42.7586310000	30.3664830000
H	31.3439670000	40.9857580000	32.5984180000
H	31.2535640000	40.6779860000	30.8723560000
H	33.8792350000	40.7429320000	30.8959020000
H	33.7539790000	40.5067640000	32.6260730000
H	32.8654090000	38.6682000000	30.4827940000
H	32.6840220000	39.1156560000	34.0159660000
H	32.8132600000	37.3857400000	34.2885330000
H	33.0406210000	36.4106410000	30.9996160000
H	32.6453440000	35.9503470000	32.6400950000
Fe	34.7281940000	30.0541860000	36.0302360000
N	32.2557040000	35.2366090000	36.6234900000
O	32.5563640000	35.4791830000	34.4101840000
C	31.5192680000	33.4280490000	35.1883900000
C	30.5740740000	33.0463530000	36.1636210000
C	29.9514760000	31.8050610000	36.1070530000
C	30.2887320000	30.9167480000	35.0796740000
C	31.2456680000	31.2836290000	34.1313130000
C	31.8759390000	32.5335750000	34.1516470000
C	32.1632540000	34.7503290000	35.3475460000
C	33.4357060000	35.0127580000	37.4654030000
C	34.7096600000	34.7270870000	36.6537750000
C	33.5941640000	36.2612000000	38.3470750000
C	33.0936470000	33.7875590000	38.3478990000

C	32.9017700000	32.8283240000	33.0830030000
C	32.2365430000	33.2742930000	31.7700580000
F	34.6951190000	31.7553150000	35.2843310000
H	30.3229730000	33.7266050000	36.9688400000
H	29.2315340000	31.5378990000	36.8725120000
H	29.8586490000	29.9211760000	35.0295930000
H	31.5418320000	30.5633300000	33.3765210000
H	33.6102700000	33.5821240000	33.4275140000
H	33.4785860000	31.9145090000	32.9140540000
H	32.9911860000	33.5179060000	31.0112710000
H	31.6009170000	32.4815340000	31.3618740000
H	31.5930440000	34.1478580000	31.9257250000
H	35.5605270000	34.6412680000	37.3389500000
H	34.6314070000	33.7845860000	36.1021090000
H	34.9183540000	35.5424240000	35.9527200000
H	33.8314370000	37.1389770000	37.7364340000
H	32.6731050000	36.4631430000	38.9013910000
H	34.4103930000	36.1116800000	39.0616270000
H	33.0107220000	32.8835250000	37.7430300000
H	33.9073440000	33.6507780000	39.0670050000
H	32.1592720000	33.9381280000	38.8933930000
O	33.7418310000	29.1035980000	34.3701980000
H	34.3553110000	28.6038360000	33.8116980000
H	32.8574190000	28.5731980000	34.4022670000
O	32.7802080000	30.1667180000	36.8930920000
H	32.1323390000	30.5084980000	36.2528890000
H	32.4658930000	30.4442420000	37.8026270000
O	31.5103500000	27.7989930000	34.3368150000
H	31.1251040000	26.9002580000	34.8574940000
H	31.2750680000	27.6392270000	33.4096990000
O	31.9860650000	30.8377730000	39.3496180000
H	31.2097450000	31.4881370000	39.4023910000
H	31.6581570000	30.0365590000	39.7871390000
O	30.6690410000	25.7680100000	35.4991500000
H	29.7047490000	25.0334130000	34.8928510000
H	30.1831120000	26.1321390000	36.2566010000

XIV. ^{1}H and ^{13}C NMR Spectra of Compounds

^{1}H NMR Spectrum of 1 (400 MHz, CDCl$_3$)

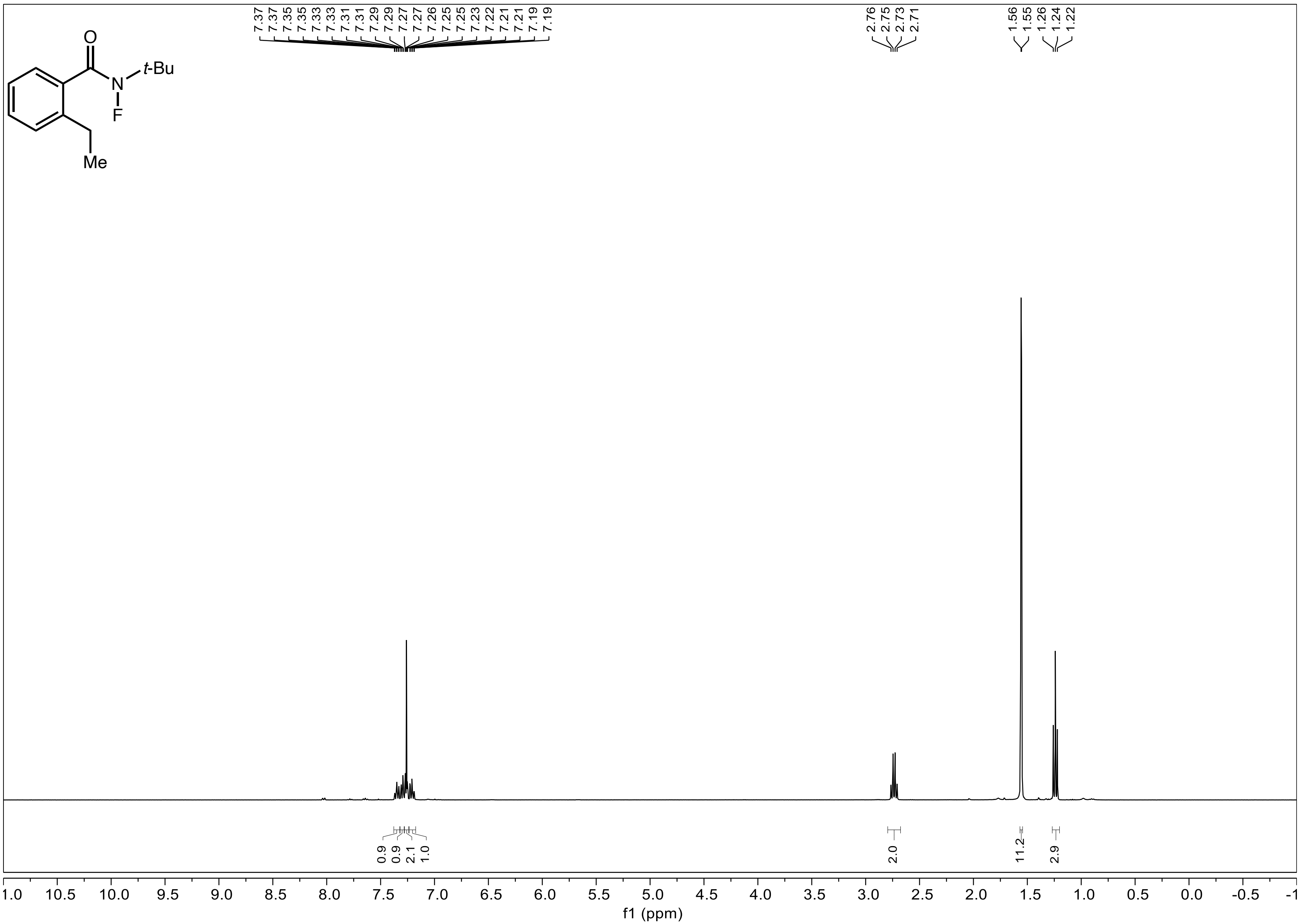

^{13}C NMR Spectrum of 1 (101 MHz, $CDCl_3$)

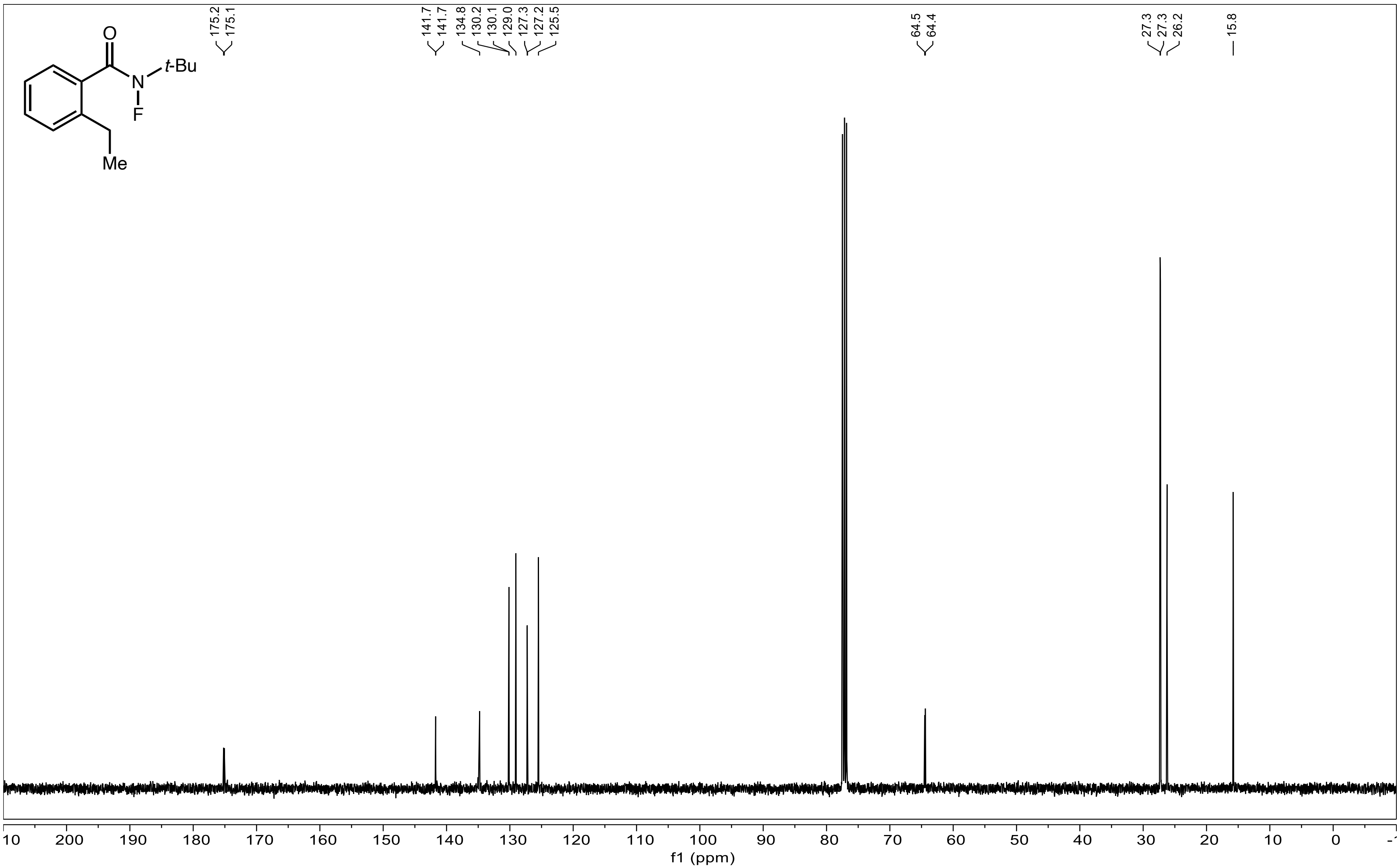

^{19}F NMR Spectrum of 1 (376 MHz, $CDCl_3$)

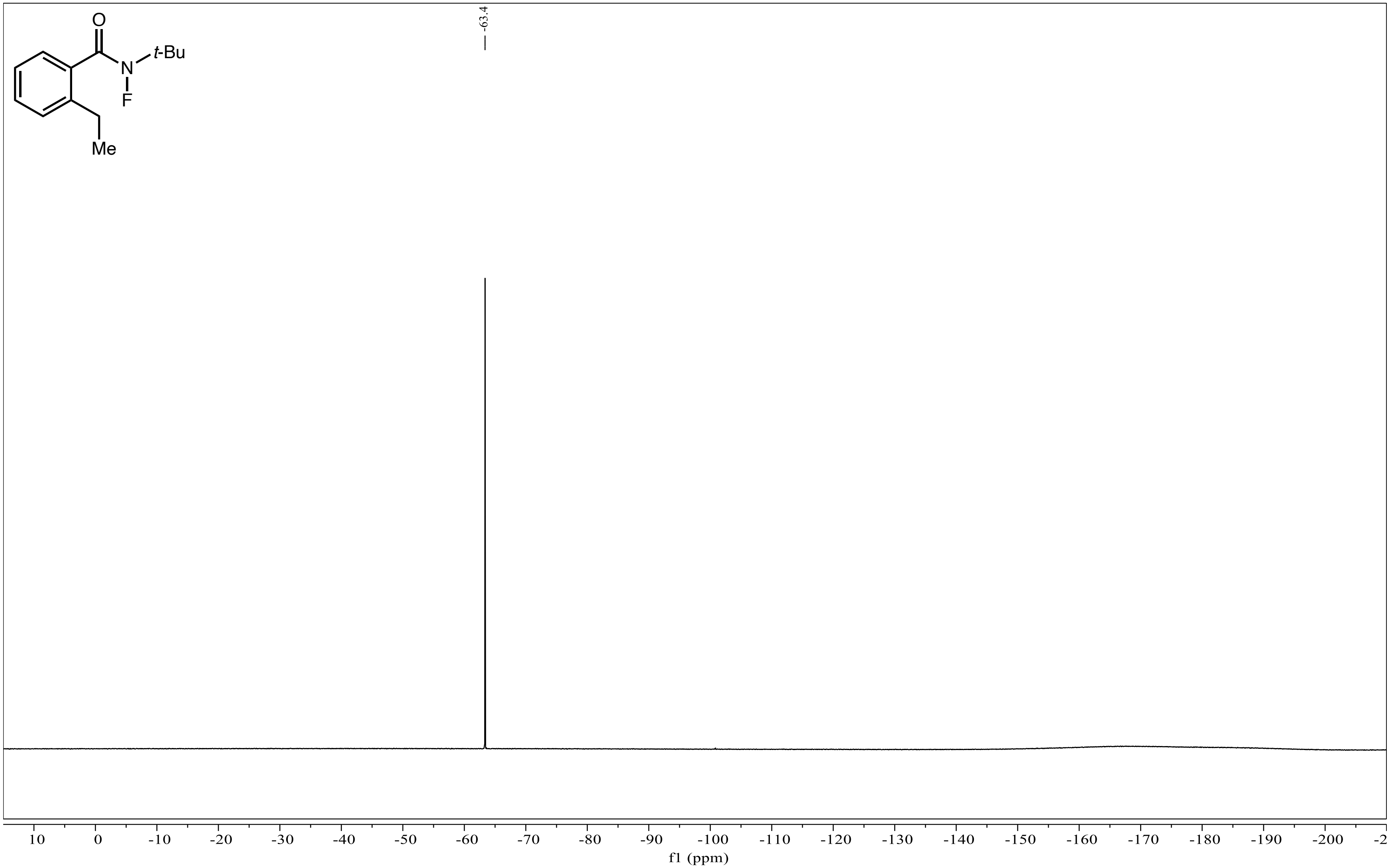

^{1}H NMR Spectrum of 2 (400 MHz, $CDCl_3$)

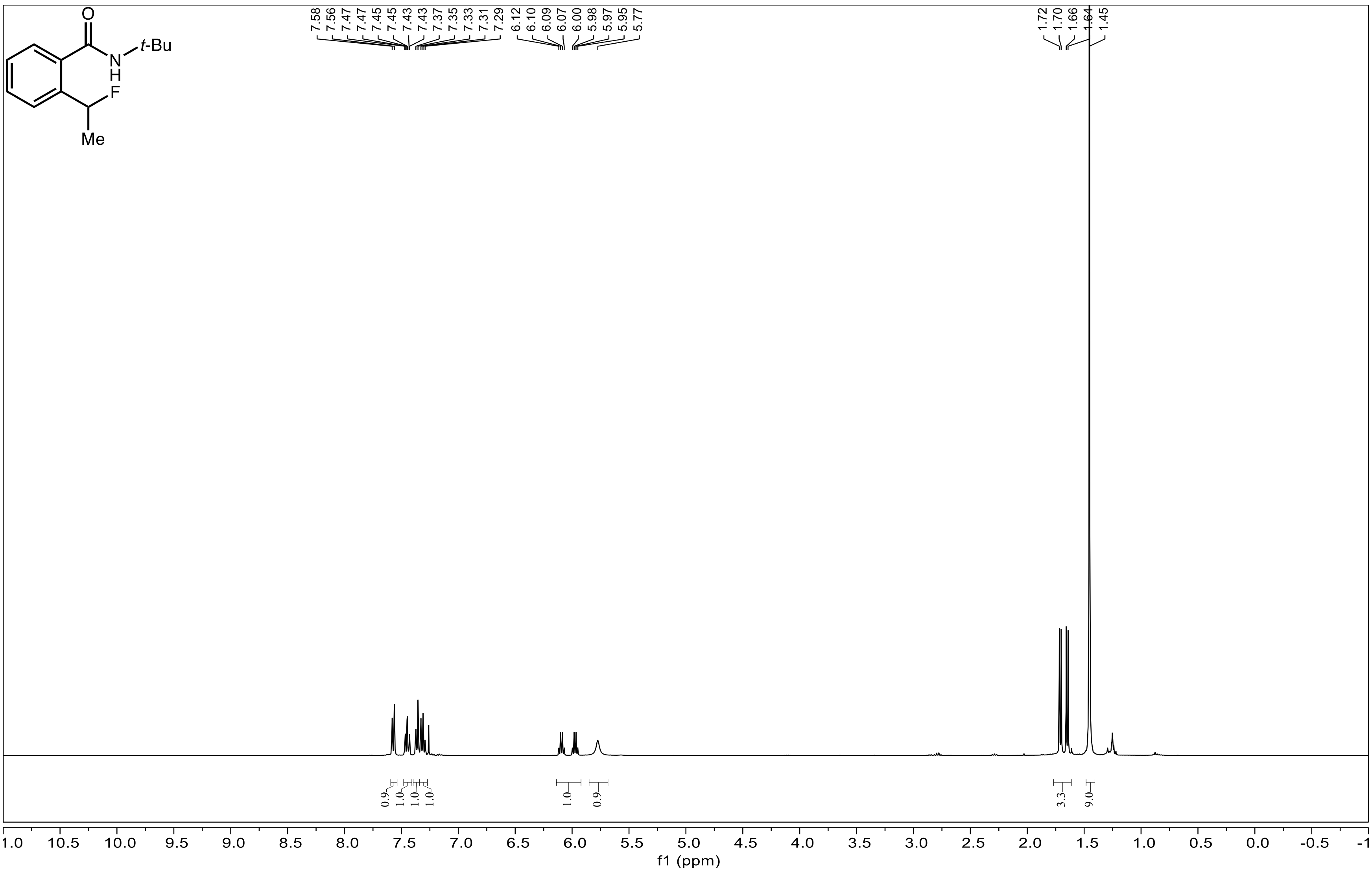

^{13}C NMR Spectrum of 2 (101 MHz, CDCl₃)

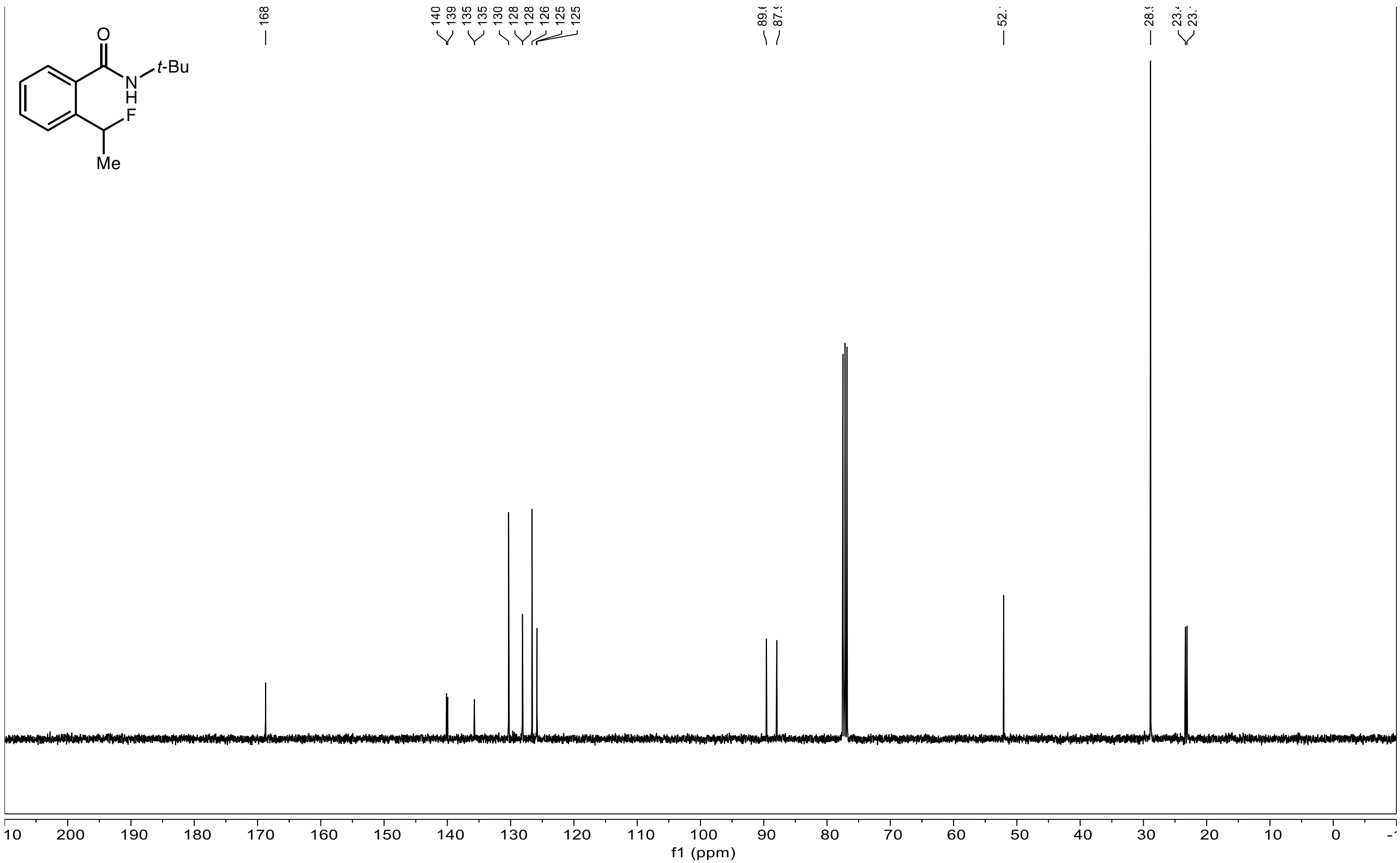

^{19}F NMR Spectrum of 2 (376 MHz, $CDCl_3$)

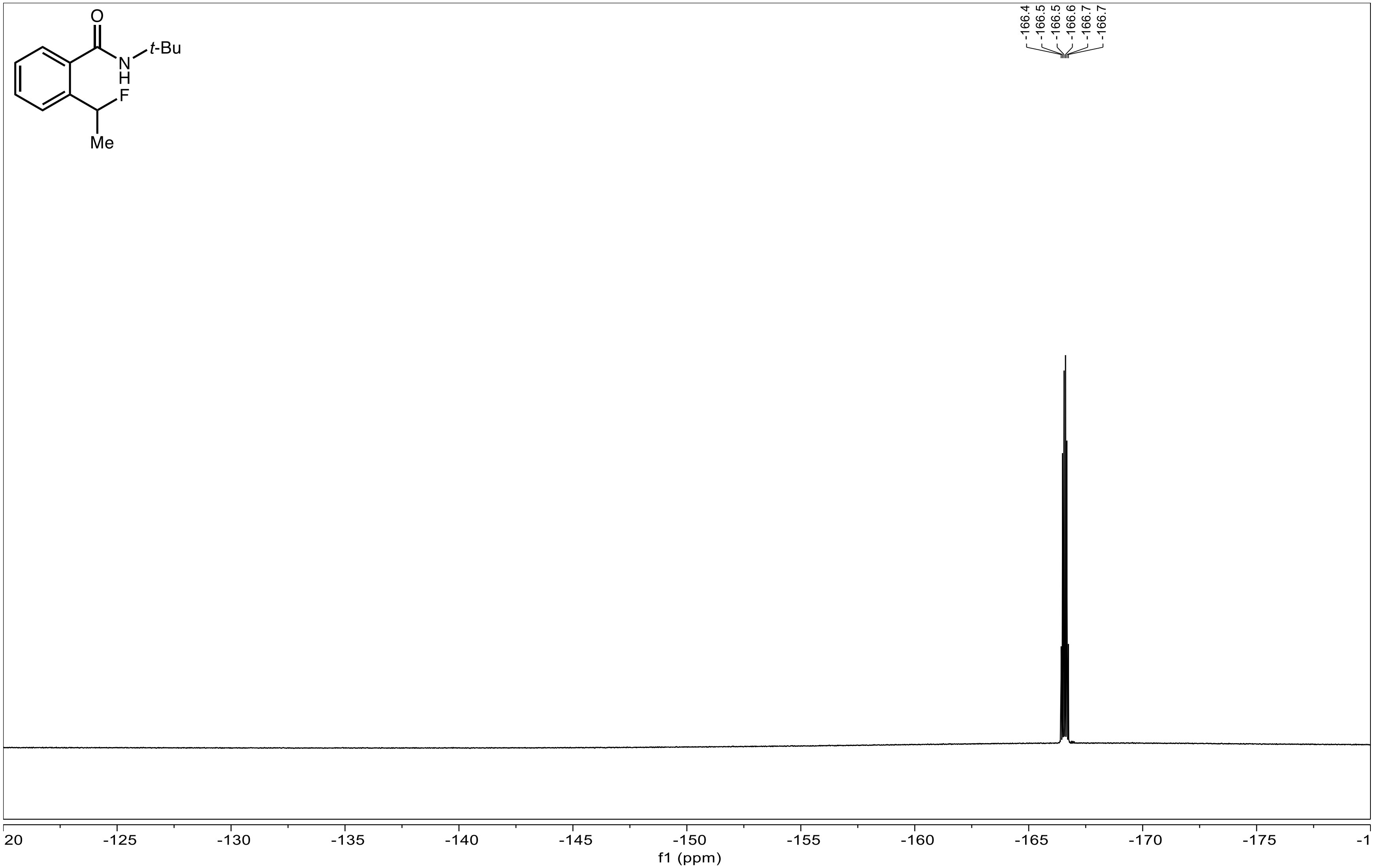

^{1}H NMR Spectrum of 3 (400 MHz, $CDCl_3$)

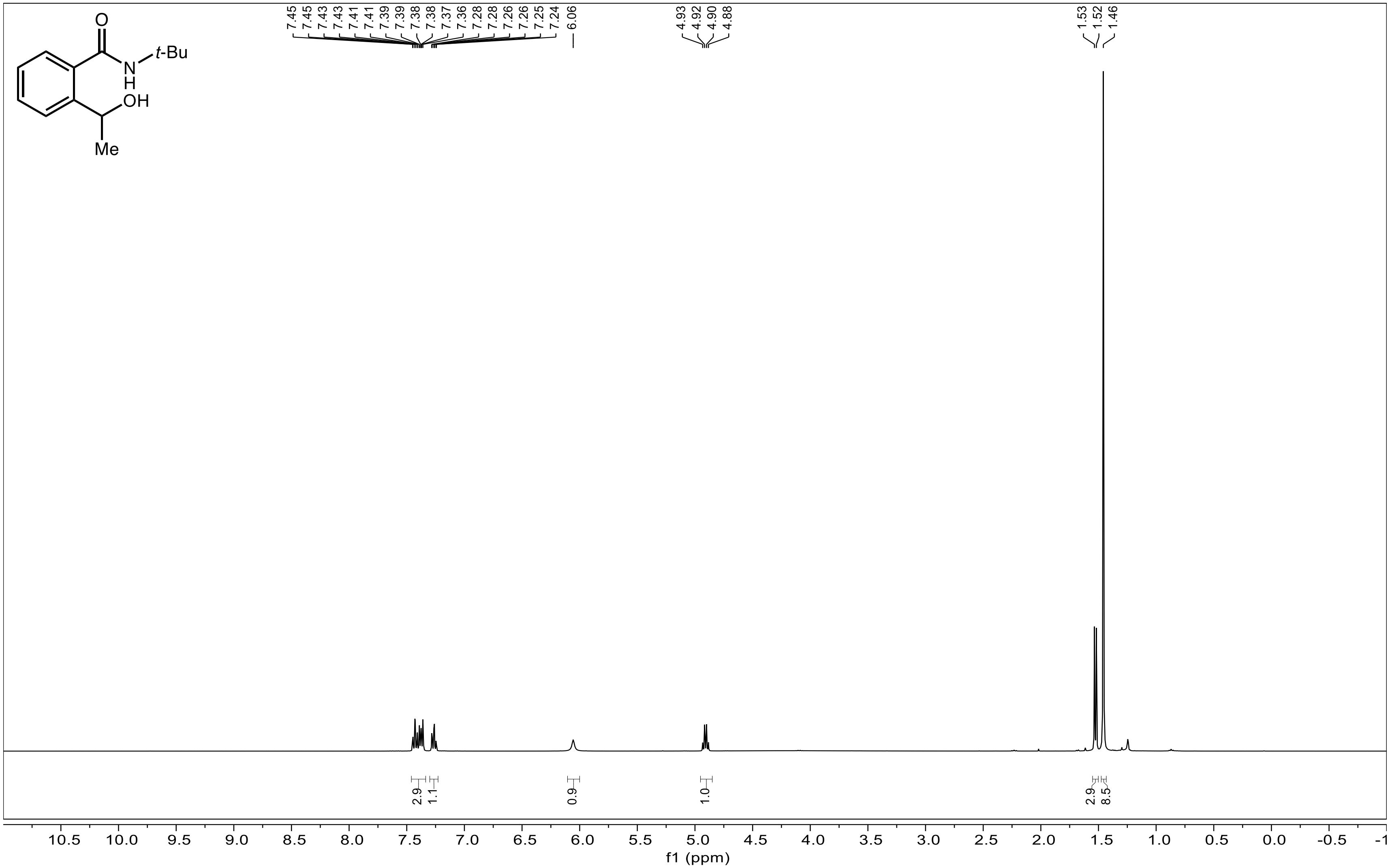

^{13}C NMR Spectrum of 3 (101 MHz, $CDCl_3$)

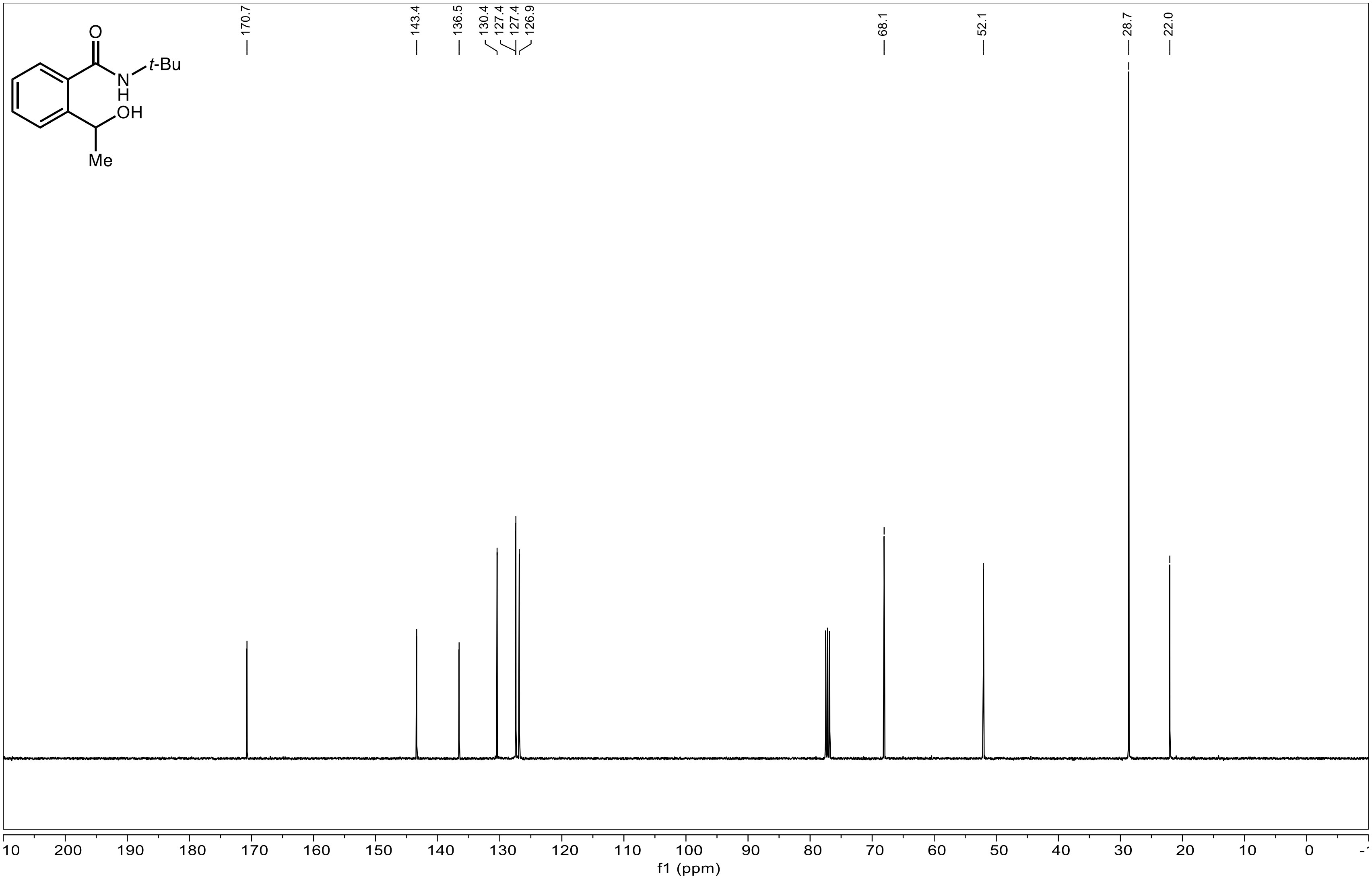

^{1}H NMR Spectrum of 4 (400 MHz, $CDCl_3$)

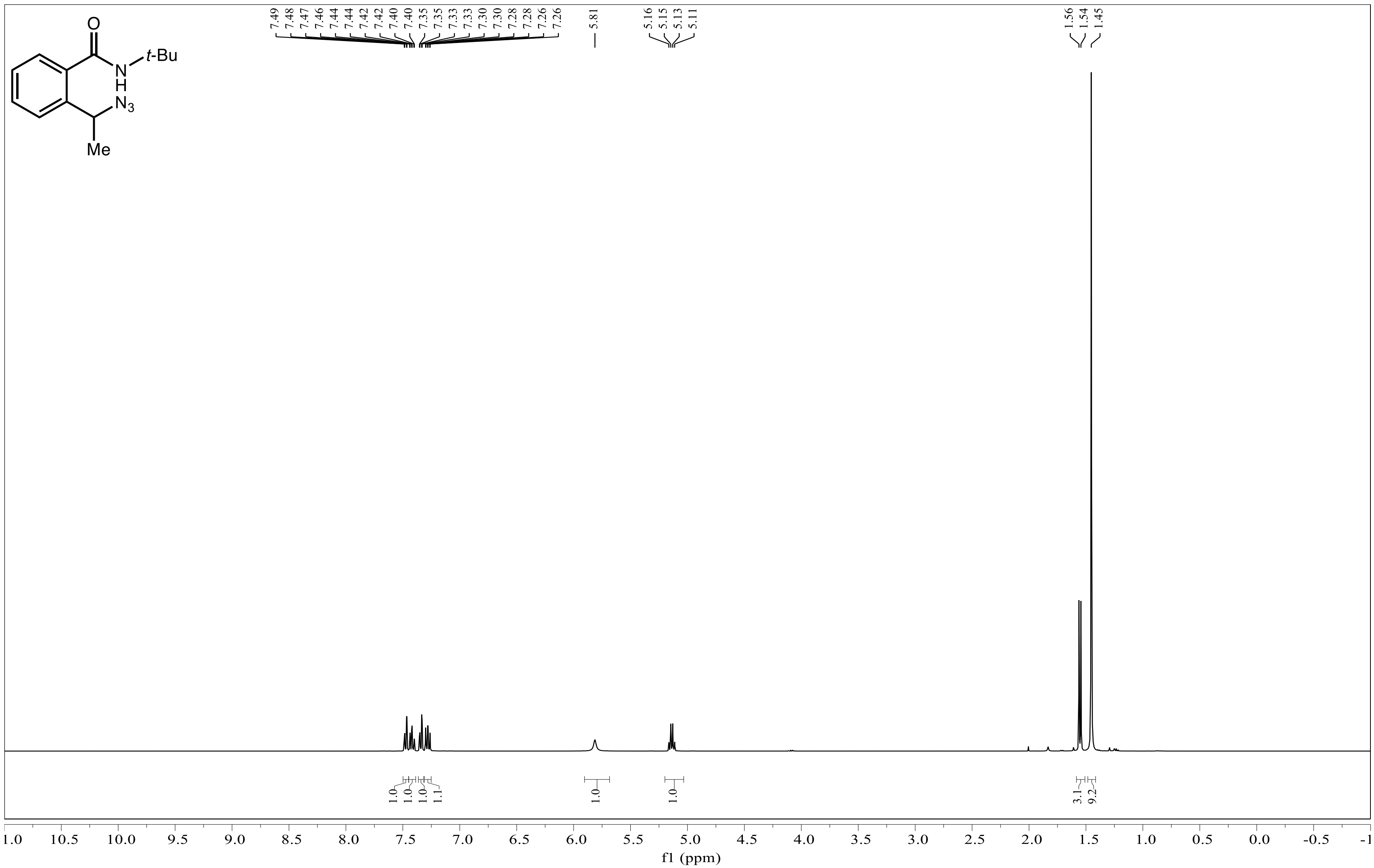

^{13}C NMR Spectrum of 4 (101 MHz, $CDCl_3$)

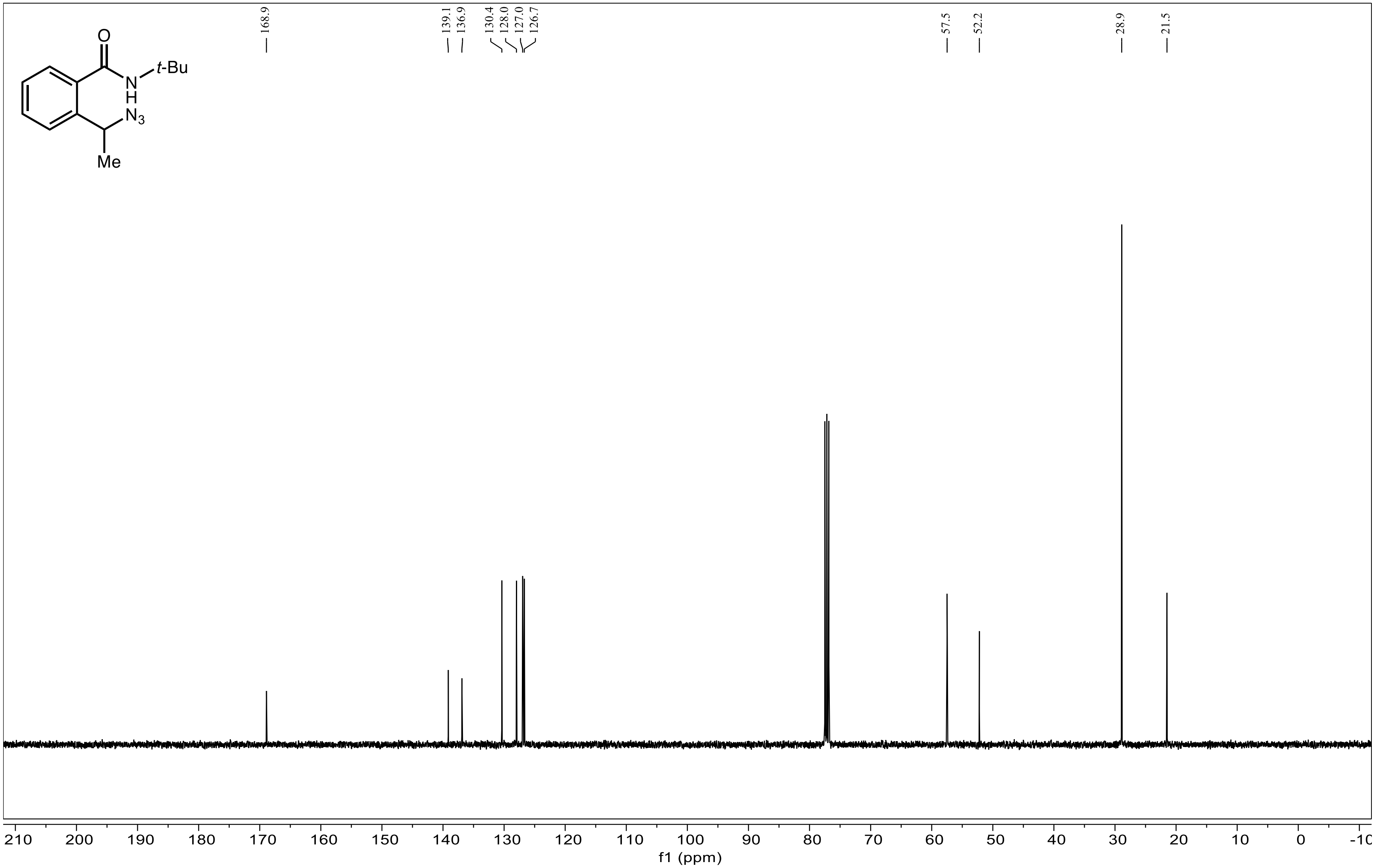

^{1}H NMR Spectrum of ethyl 2-(1-azidoethyl)benzoate (400 MHz, $CDCl_3$)

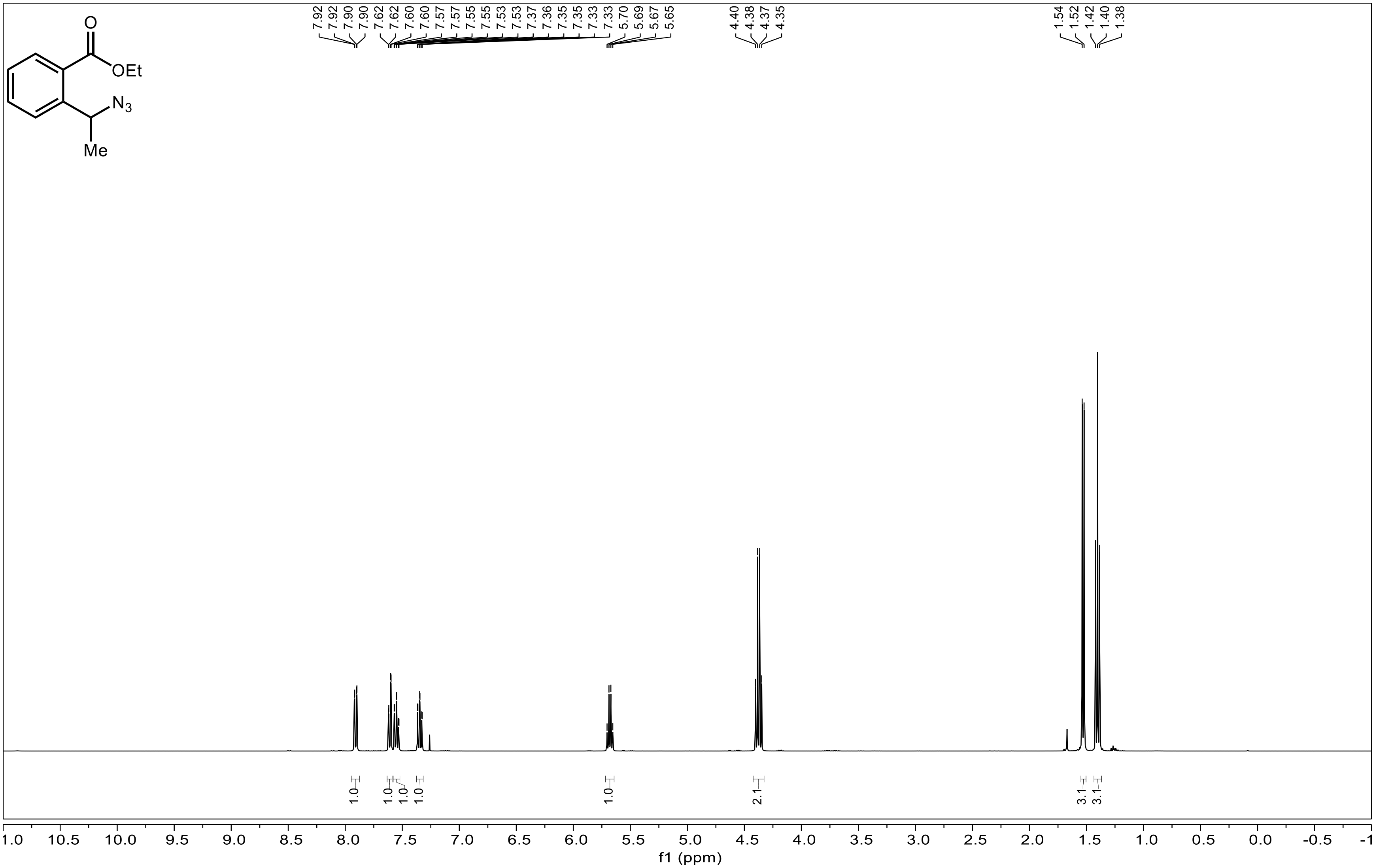

^{13}C NMR Spectrum of ethyl 2-(1-azidoethyl)benzoate (101 MHz, $CDCl_3$)

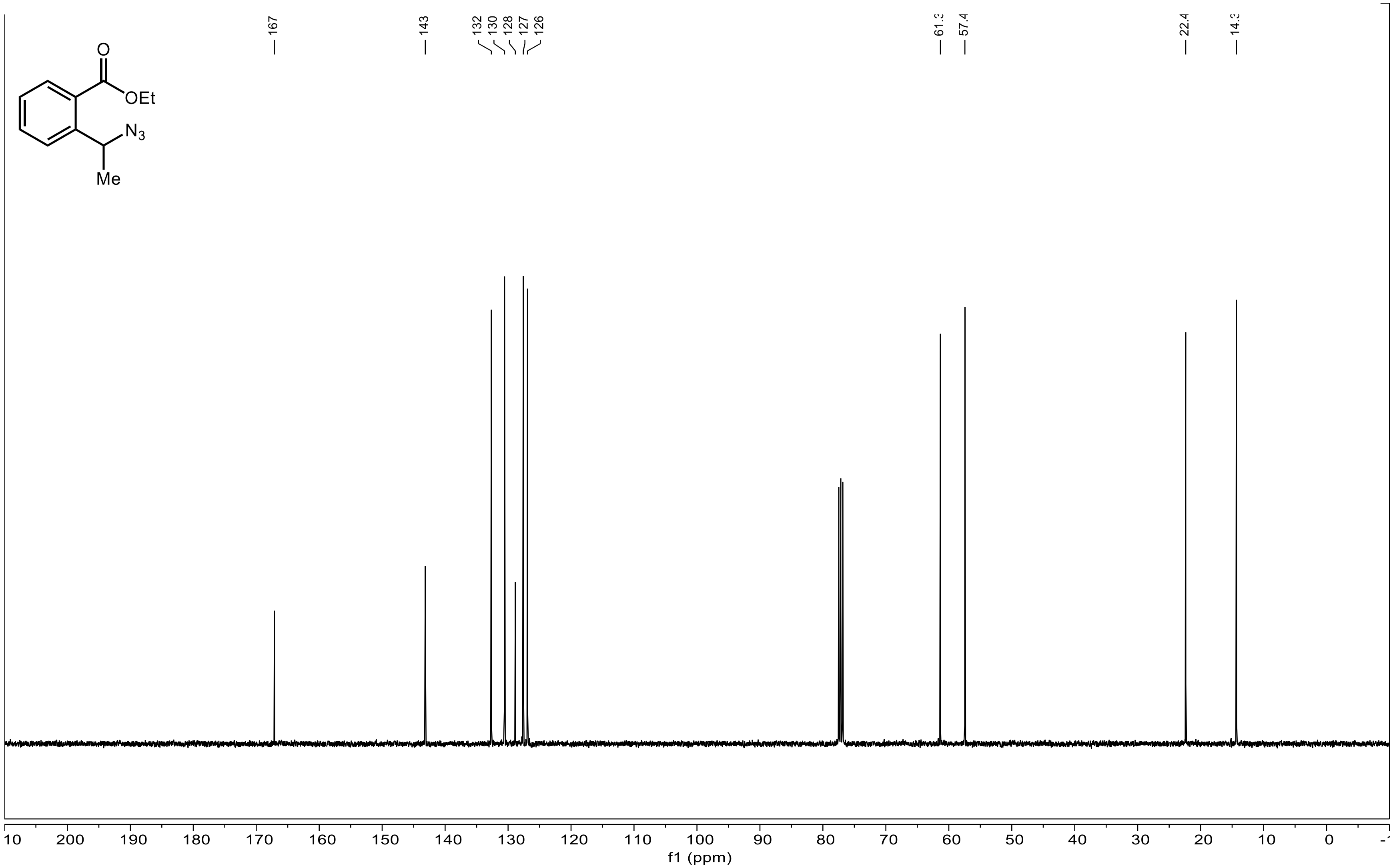

Supplement: Supplementary file 1 [file media-1.pdf]
